# Micro-ribonucleic acids and extracellular vesicles repertoire in the spent culture media is altered in women undergoing *In Vitro* Fertilization

**DOI:** 10.1038/s41598-017-13683-8

**Published:** 2017-10-19

**Authors:** Masood Abu-Halima, Sebastian Häusler, Christina Backes, Tobias Fehlmann, Claudia Staib, Sigrun Nestel, Irina Nazarenko, Eckart Meese, Andreas Keller

**Affiliations:** 1 Institute of Human Genetics, Saarland University, 66421 Homburg/Saar, Germany; 2 Department of Obstetrics and Gynaecology, University of Würzburg, School of Medicine, 97080 Würzburg, Germany; 3 Chair for Clinical Bioinformatics, Saarland University, 66041 Saarbrücken, Germany; 4 Institute of Anatomy and Cell Biology, University of Freiburg, 79085 Freiburg, Germany; 5 AG Exosomes and Tumor biology, 79085 Freiburg, Germany

## Abstract

MicroRNAs (miRNAs) are class of small RNA molecules with major impact on gene regulation. We analyzed the potential of miRNAs secreted from pre-implantation embryos into the embryonic culture media as biomarkers to predict successful pregnancy. Using microarray analysis, we profiled the miRNome of the 56 spent culture media (SCM) after embryos transfer and found a total of 621 miRNAs in the SCM. On average, we detected 163 miRNAs in SCM of samples with failed pregnancies, but only 149 SCM miRNAs of embryos leading to pregnancies. MiR-634 predicted an embryo transfer leading to a positive pregnancy with an accuracy of 71% and a sensitivity of 85%. Among the 621 miRNAs, 102 (16.4%) showed a differential expression between positive and negative outcome of pregnancy with miR-29c-3p as the most significantly differentially expressed miRNA. The number of extracellular vehicles was lower in SCM with positive outcomes (3.8 × 10^9^/mL EVs), as compared to a negative outcome (7.35 × 10^9^/mL EVs) possibly explaining the reduced number of miRNAs in the SCM associated with failed pregnancies. The analysis of the miRNome in the SCM of couples undergoing fertility treatment lays the ground towards development of biomarkers to predict successful pregnancy and towards understanding the role of embryonic miRNAs found in the SCM.

## Introduction

Worldwide, about 72.4 million couples suffer from subfertility. To overcome undesired childlessness, about 40 million turns to Assisted Reproduction Techniques (ART) like *In Vitro F*ertilization (IVF) and/or Intracytoplasmic Sperm Injection (ICSI)^1^. Unfortunately, only about 30% of all IVF-cycles manage to establish pregnancies^2^. Unsuccessful treatment approaches cause considerable physical and psychological stress to the corresponding patients. The chances that a specific IVF-cycle (either after fresh stimulation/egg retrieval or after cryopreservation of two-pronuclear zygotes or embryos in earlier treatments) will induce a pregnancy depends on the fitness of the transferred embryo^3^. On average, no more than 2-3 out of about 9 embryos obtained in one treatment cycle are transferred^4^. Otherwise, an excess of multiparous pregnancies would have to be expected^4^. Considering the medical risks of multiparous pregnancies including preterm delivery, increased rate of surgically induced deliveries or twin-to-twin transfusion syndrome^1,5^, the restriction appears reasonable and is also legally enforced in countries like Germany. However, a lack of diagnostics that accurately predict the reproductive competence of a given embryo often results in the need of multiple stimulations. Bühler *et al*., concluded that 10 IVF stimulations would be needed to achieve an 80% probability of pregnancy in the average IVF patient in Germany^2^. To reduce the average number of potentially harmful treatment cycles, new diagnostic tools are needed to identify the most capable embryo derived from the retrieved oocytes^6^. Beyond image-based approaches, molecular markers such as nucleic acids play an increasing role for reliable diagnosis. Among nucleic acids, small non-coding RNAs, so-called microRNAs (miRNAs), show a substantial diagnostic potential. Many diseases as well as physiological conditions were already found to be associated with specific miRNA expression patterns^7^. After more than one decade of biomarker discovery, miRNA patterns have specifically become of interest for analyzing male and/or female partners undergoing ART^8–16^. Recently, it has been shown that RNAs including miRNAs are selectively secreted from pre-implantation embryos into the embryonic culture media and may have a potential as biomarkers of embryo development^17–19^. While previous studies demonstrated that miRNAs are secreted and may be correlated to successful pregnancy there are no studies that offer a comprehensive analysis covering all 2,549 known human miRNAs. Since single techniques for measuring miRNAs, including next generation sequencing and microarrays, have an inherent bias in miRNA profiling^20^, we combined two techniques with microarray screening in an initial analysis and RT-qPCR in an evaluation analysis. Due to the low amount of miRNA per embryonic culture media, high-throughput sequencing is not possible unless by pooling samples. With this study, we aimed to identify miRNA expression patterns (miRNomes) in the SCM following embryo transfer and to assess whether specific miRNAs may serve as biomarker for embryo quality and for successful pregnancy. We also explore the number and size distribution of extracellular vesicles^21^ in the SCM following embryonic transfer.

## Materials and Methods

### Study Population and Sample Collection

The study was approved by the Institutional Review Board (Nr. 160/15) of the University Hospital of Saarland and written informed consent was obtained from all participants before inclusion. The methods in this study were carried out in accordance with the approved guidelines by University Hospital of Saarland and University Hospital of Würzburg and all experimental protocols were approved by the ethics committee. All the females included in the study underwent same controlled ovarian stimulation and transvaginal ultrasound-guided oocyte retrieval was performed according to standard procedures of the University hospital of Würzburg, Germany. After fertilization with conventional ICSI procedure, embryos were transferred into the wells of the EmbryoSlide^**®**^ culture dish (Unisense FertiliTech, Aarhus, Denmark) and placed into the EmbryoScope^**®**^ time-lapse incubator (Unisense FertiliTech, Aarhus, Denmark) until transfer under the conditions of 6.0% CO_2_, 7.0% O_2_, and 37.0 °C in 25 µL individual wells containing droplets of Continuous Single Culture^®^ Medium (CSCM) (Irvine Scientific – USA). The SCM was collected individually on Days 3 and Day 5 from all embryos that displayed two pronuclei and completed the first division, and stored individually at −80 °C until the analysis was performed. In total, 64 subfertile females underwent single-embryo transfer cycle were included in the study. Seventeen-microliter (µL) SCM from 56 females/single-embryo transfers were used for the miRNA profiling to investigate whether the miRNA(s) correlates with pregnancy outcome and 9 µL SCM from 8 females/single-embryo transfers were used for EVs concentration measurements as a small ‘Proof-of-Concept’ to investigate whether the EVs concentration correlates with pregnancy outcome. In addition, two negative controls were cultured in the same conditions but without embryos i.e. media that were not exposed to blastocysts were included to provide an estimation of a background distribution of miRNAs.

### Total RNA, including miRNAs isolation

Total RNA, including miRNAs from 56 SCM was isolated using miRNeasy Micro Kit on the QIAcube™ robot (Qiagen, Germany) according to the manufacturer’s instructions. The *Caenorhabditis elegans* (*C. elegans*) miR-39 mimic was added to each isolation as an internal spike-in control (Qiagen, Germany). DNase I treatment (Thermo Fisher Scientific, USA) was carried out and excluded as perversely described^22^. The concentration of the isolated total RNA, including miRNAs was quantified using NanoDrop Spectrophotometer (Thermo Fisher Scientific, USA) (Supplemental Table 1 **)**. The RNA integrity was assessed using Agilent 2100 Bioanalyzer RNA 6000 Pico Kit (Agilent Technologies, USA) (The RIN values are provided in Supplemental Fig. 1 **)**.

### MiRNA microarray and array measurement procedure

MiRNA expression profiling analysis for the 56 SCM was established using SurePrint™ 8 × 60 K Human v21 miRNA microarrays (Agilent Technologies, USA). These microarrays contain ~20 replicates for each probe complement to each of the 2549 mature miRNAs of miRBase v21. These probes act in concert to measure the miRNA of interest, and the data are combined later during software analysis. All probes are randomly distributed on the array, and cross hybridization is prevented by the addition of a G residue and a hairpin at the 50-end of the probe. All procedures were carried out according to the manufacturer’s recommendations and as described previously^23^. In brief, a total of 100 ng total RNA from each SCM was dephosphorylated by incubation with calf intestinal phosphatase (CIP) at 37 °C for 30 minutes and denatured with the use of 100% Dimethyl sulfoxide (DMSO) at 100 °C for 7 minutes. Samples were labeled with pCp-Cy3 with the use of T4 ligase at 16 °C incubation for 2 hours. Each labeled RNA sample was then hybridized onto an individual sub-array of the 8 × 60 K format, with each array containing probes for 2549 miRNAs according to miRBase v21. Hybridizations were performed in SureHyb chambers (Agilent Technologies, USA) at 55 °C for 20 hours with rotation. Arrays were then washed, dried and scanned at a resolution of 3 μm double-pass using Agilent G2565C scanner. Data were acquired using Agilent AGW Feature Extraction software version 10.10.11 (Agilent Technologies, USA). In addition to the SCM samples that have been collected following embryo transfer, we also included pure culture media (i.e. media that were not exposed to blastocysts) as negative control (CSC-Medium, Irvine Scientific, USA) to highlight the potential false positive (background noise) miRNA signals.

### Reverse Transcription and RT-qPCR of miRNAs

RT-qPCR analysis was performed to evaluate the results obtained in the initial screening microarray experiments. Out of 56 SCM samples that were used for the microarray analysis, 28 samples were used for the RT-qPCR analysis. These 28 samples yielded a sufficient RNA quantity allowing RT-qPCR analysis using *mi*Script miRNA PCR system (Qiagen, Germany). All steps were curried our according to the manufacturer’s recommendations. In brief, 30 ng RNA was converted into cDNA using *mi*Script II RT Kit. The resulted cDNA was then diluted 1:5 and 1 µL of cDNA was mixed with 10 µL 2X *mi*Script SYBR Green mix, 2 µL 10X *mi*Script Universal Primer, 2 µL 10X *mi*Script Primer Assay for 8 selected miRNAs namely hsa-miR-29c-3p, hsa-miR-566, hsa-miR-22-5p, hsa-miR-6812-5p, hsa-miR-30c-5p, hsa-let-7c-5p and hsa-miR-6076 in a total volume of 20 µL. Hsa-miR-16-2 and Ce_miR-39_1 *mi*Script Primer Assays were used as an endogenous control and as an internal spike-in control, respectively^24^ for normalization analysis. Reactions were run on a StepOnePlus™ Real-Time PCR System (Applied Biosystems, USA) with the following thermal cycling parameters: initial activation step 95 °C for 15 minutes followed by 40 cycles at 94 °C for 15 seconds (denaturation), 55 °C for 30 seconds (annealing), and 70 °C for 30 seconds (extension). Melt curve analysis was carried out to check if the assays have produced single, specific products.

### Extracellular vesicles (EV) in the SCM

Based on the fact that extracellular miRNAs contained within extracellular vesicles (EV), we tested EVs concentration in the SCM by Nanoparticle Tracking Analysis (NTA) using the ZetaView PMX110 (Particle Metrix, Germany) and its corresponding software ZetaView software 8.02.30.02. Eight SCM samples were used as a small ‘Proof-of-Concept’. Samples were centrifuged at 5000 g, 4 °C for 15 minutes to remove cell debris. For each sample, 9 µL was used for a 1:111 dilution using 0.1X Phosphate Buffer Saline (PBS) in order to make up the required volume of 1-mL. Measurements parameters like minimum brightness ‘20’, minimum size ‘5’, and maximum size ‘500’, camera sensitivity varied from ‘49–68’, shutter ‘100’, video acquisition ‘moderate’ were used. The size distribution measurements were recorded at 11 positions and 5 cycles. The Zeta potential measurements were recorded at ‘2’ positions and ‘5’ cycles. The temperature ranged from ‘21 to 22 °C’. The EV fractions were divided into three groups, below 30 nm, 30-200 nm (corresponding approximately size of exosomes) and larger than 200 nm (corresponding approximately size of microvesicles). In addition to the relative size distribution, we counted the absolute number of EVs in the SCM. As a negative control, culture media not exposed to blastocysts was measured. Additionally, dynamic light scattering DLS was approached to detect the presence of EVs beyond the NTA detection limit, smaller 20 nm and larger 1 µm. The size distribution was calculated according to the signal intensity.

### Statistical Analysis

We used the freely available R statistical environment (version 3.2.4. for MacOS) to analyze the microarray row data. MiRNAs were considered as detected according to the detection flag provided by Agilent’s detection flag. Specifically, we extracted the “gIsGeneDetected” column from the raw data file and denote the respective binary values as present calls. To account the global variations between microarrays, quantile normalization was carried out (*preprocessCore* package) and not detected miRNAs were subsequently eliminated. To find miRNAs that are more frequently present in the one or the other group (only the binary signal ‘present’ versus ‘absent’), Fishers Exact test was applied to the 2 × 2 contingency table, containing present calls for each miRNA in both groups. For comparing groups, hypothesis tests were performed. Since not all miRNAs were normally distributed, we calculated the significant values not only for the parametric t-test, but also for non-parametric Wilcoxon-Mann Whitney test. If not mentioned explicitly, *P-*values were subjected to adjustment for multiple testing applying the Benjamini-Hochberg approach.

### MiRNA Target Prediction and Pathway Analysis

To investigate downstream effects of miRNAs, we employed six web-based computational tools that we have previously implemented and published. For the analysis of miRNA sets, we used the microRNA enrichment analysis and annotation tool miEAA^25^ (available at http://www.ccb.uni-saarland.de/mieaa_tool/), which relies on the GeneTrail framework^26,27^ (available at http://genetrail2.bioinf.uni-sb.de/). Effects of single miRNAs on pathways have been determined by the miRNA Pathway Dictionary^28^ (available at https://mpd.bioinf.uni-sb.de/). The complex pathway analysis has been done using miRTargetLink^29^ (available at https://ccb-web.cs.uni-saarland.de/mirtargetlink). The distribution of miRNAs across different tissues and body fluids has been checked using the Human miRNA Tissue Atlas^23,30^ (available at https://ccb-web.cs.uni-saarland.de/tissueatlas/). If not mentioned explicitly, all tools have been used with standard parameters.

## Results

### Present and absent miRNAs in the SCM following embryo transfer

**Figure 1:**
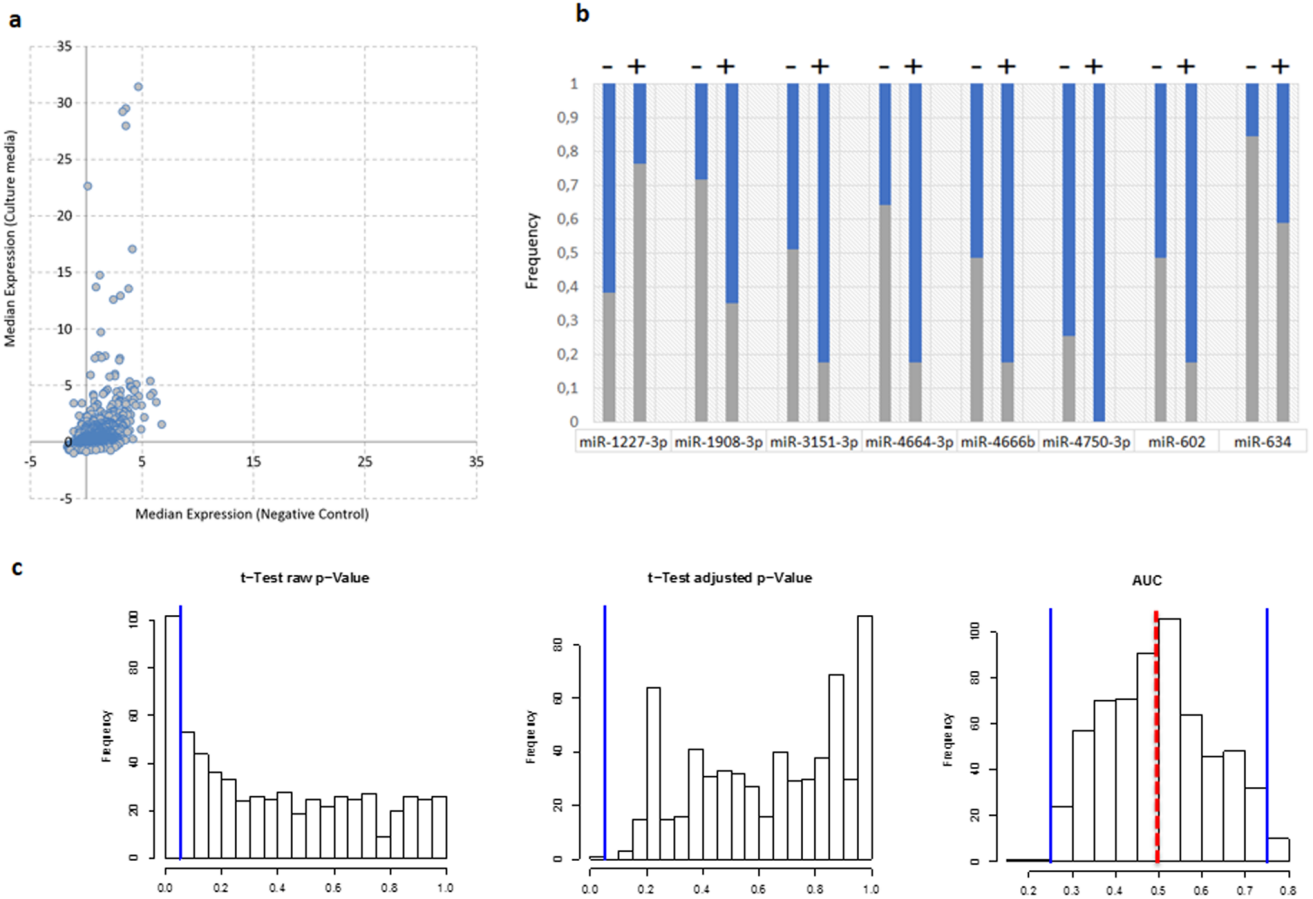
(**a**) Raw median expression intensity for all the miRNAs in pure culture media negative control versus culture media exposed to blastocysts. (**b**) Percentage of the detected miRNAs [Silver] versus not detected miRNAs [Blue] in negative samples (left bar each) and positive samples (right bar each). All results were significant according to fishers test *P-*value (alpha level of 0.05) prior to adjustment for multiple testing. (**c**) Histograms of raw *P-*values, adjusted *P-*values and the AUC values for the comparison of 621 miRNAs in the SCM where oocytes did not lead to pregnancy versus oocytes leading to pregnancy. Blue lines in the first two histograms denote the 0.05 alpha level, in the right histogram AUC of 0.25 to 0.75. Red dotted line represents miRNAs with AUC of 0.5.

We profiled the miRNA repertoire of the 56 spent culture media (SCM) after embryos transfer. While embryos lead to a positive pregnancy transfer in 17 females, no pregnancy occurred for the remaining 39 females. A total number of 621 different human miRNAs was found in the SCM (see Supplemental Table 2), the largest fraction of which showed a rather low expression slightly above background. Since we previously reported miRNA signals even in the pure water^31^, we profiled pure culture media not exposed to blastocysts using the same microarray technology. We found 28 miRNAs in the first control culture media and 12 miRNAs in the second control culture media (Supplemental Table 3). The number of miRNAs in control culture media was significantly (z-score of 2.7, two-tailed *P*-value of 0.007) below the number in culture media exposed blastocysts. Likewise, we found a difference between the median raw intensity of the controls versus the median of the raw intensity of the culture media samples, which were exposed to blastocysts (Fig. 1a). The number of detected miRNAs was within the range of miRNome complexity that we found in human organs or body fluids^30^. Almost all of the identified miRNAs in both control pure culture media are presumed miRNAs that have not been discovered until most recently and deposited in later miRBase versions. We and others demonstrated that the recently in miRBase deposited miRNAs are mostly not true miRNAs^32,33^. We next asked whether the complexity of the miRNome differs between samples leading to pregnancy (positive samples) compared to samples not leading to pregnancy (negative samples). In patients with a negative pregnancy we detected on average 163 miRNAs, but only 149 miRNAs in samples from a positive pregnancy. To identify specific miRNAs that differ between both groups, we calculated 2 × 2 contingency tables for each miRNA, containing the number of positive and negative samples with the miRNA being present or absent. Significance values were obtained by Fishers Exact test. The best separation was obtained for miR-634 which predicted with an accuracy of 71% at a sensitivity of 85% whether an embryo transfer would lead to a positive pregnancy. The results for eight markers with *P-*values below 0.05 prior to adjustment are presented in Fig. 1b. In line with our abovementioned finding that the SCM from embryos leading to a pregnancy have a decreased repertoire of miRNAs, seven of the eight miRNAs presented in Fig. 1b occur significantly less frequent in such samples.

### MiRNA abundance in the SCM following embryo transfer

**Figure 2:**
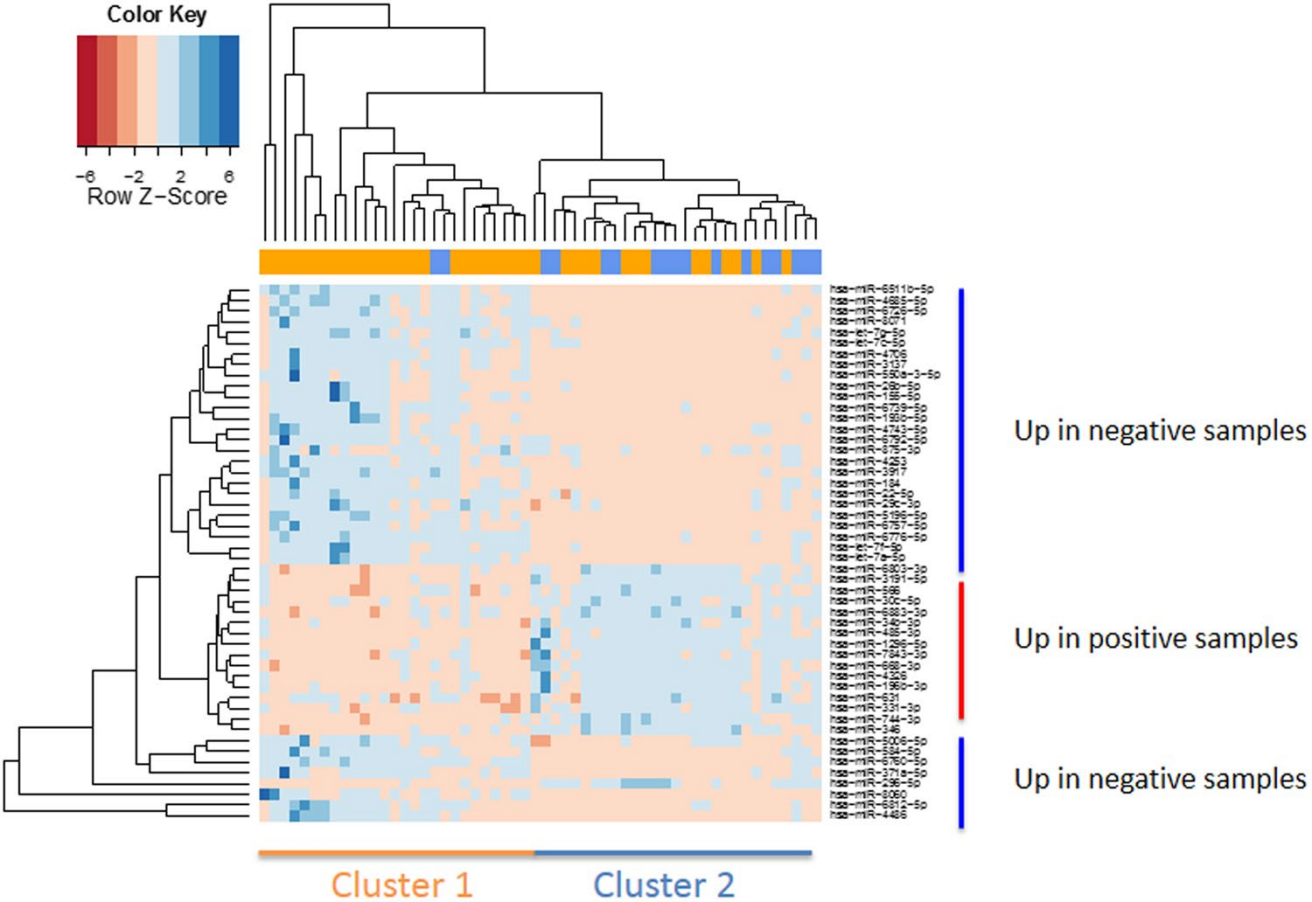
Heatmap of miRNA abundances in culture media (spent culture media (SCM)) with embryos leading to a successful pregnancy as indicated in blue and versus miRNA abundances in media from failed pregnancies as indicated in orange. As indicated on top of the heatmap the left cluster largely consists of cases with a failed pregnancies (25 out of 27), while the right cluster consists of a mixture of negative and positive outcomes of pregnancies. On the right-hand side, there are three clusters of miRNAs indicated, with two clusters containing up-regulated SCM miRNAs from cases with failed pregnancies and one cluster with up-regulated SCM miRNAs from embryos leading to pregnancies.

By correlating the miRNA expression intensity with a positive pregnancy using an un-paired two-tailed t-test, we found 102 of 621 miRNAs (16.4%) that were significant at an alpha level of 0.05. Of these 102, 70 (68.6%) had significantly lower expression intensity in the positive samples. Following adjustment for multiple testing, miR-29c-3p remained significant with a raw- and adjusted *P-*value of 3.1 × 10^−5^ and 0.019, respectively, and an AUC value of 0.83. Expression values for all miRNAs are presented in Supplemental Table 2. Histograms showing the distribution of raw- and adjusted *P-*values as well as the area under the curve (AUC) values are shown in Fig. 1c. A heatmap providing a graphical representation of the clustering of the most significantly dysregulated miRNAs is given in Fig. 2.

### Relative quantitative of selected miRNAs by Real time PCR

For evaluation of seven selected miRNAs, we used RT-qPCR. In detail, we analyzed the abundance of let-7c-5p, miR-22-5p, miR-29c-3p, miR-30c-5p, miR-566, miR-6076 and miR-6812-5p. MiR-16 was used as an endogenous control and spiked-in *C. elegans* miR-39 as normalization control. By comparing Ct values without endogenous control and ∆Ct values with both the spike-in and the endogenous controls, we observed generally concordant results. Discordant results between RT-qPCR and microarrays were only observed for miR-30c-5p. In this case RT-qPCR showed a decreased expression in samples associated with pregnancy, independent on whether raw Ct values or both ∆Ct values were used, while microarrays showed an increased expression. Since significance values do not take into account whether miRNAs are up- or down-regulated, we correlated the AUC values of miRNAs obtained by microarrays to those obtained by RT-qPCR. In general, the RT-qPCR results correlated with microarray data, and the pattern of a lower miRNA expression in samples leading to pregnancy was confirmed. This correlation analysis also showed the influence of the choice of the normalization method. The least positive correlation was observed for ∆Ct values compared to the endogenous control. In this case microarrays and RT-qPCR correlated with a Pearson Correlation Coefficient of 0.37. The raw Ct values correlated with correlation coefficient of 0.41. The highest correlation coefficient of 0.58 was obtained for the measurements normalized using the spike-ins.

### The role of miRNAs in the SCM

To improve our understanding of the potential role of the previously mentioned miRNAs, we employed different web-based computational tools that we previously published as described in the Materials and Methods section.

**Figure 3:**
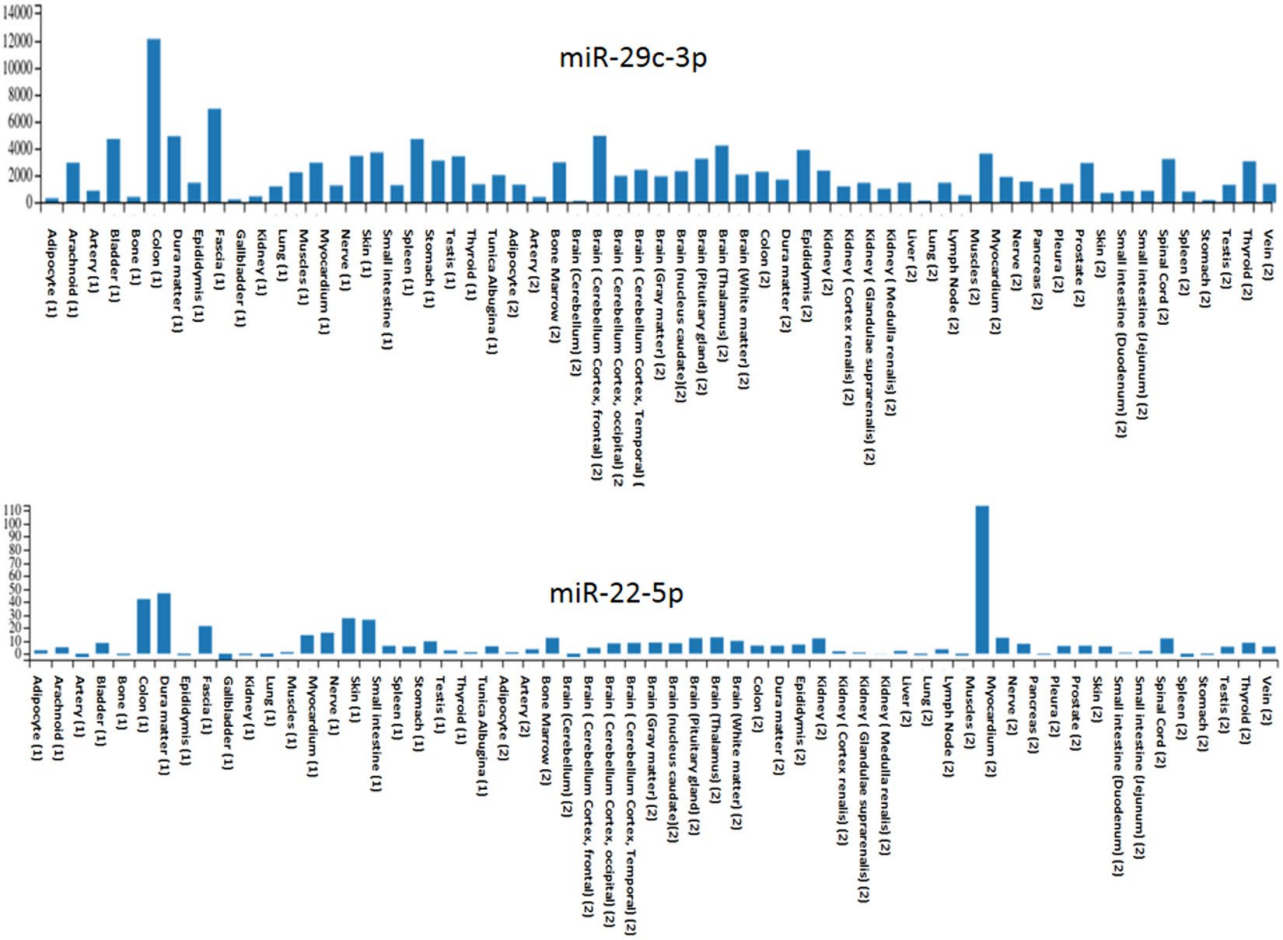
Distribution of miRNAs from SCM in tissues. The plot shows the distribution across many human tissues for 2 of the significantly dysregulated 10 miRNAs. The remaining three miRNAs were not found in the respective tissues.

By using our recently developed human miRNA tissue atlas, we asked whether the miRNAs identified in the SCM are tissue specific or broadly expressed across different tissues. Notably, all samples from the tissue organ atlas, which currently holds 982 complete miRNomes have been measured using the same microarray technology. Of the 10 miRNAs that were most abundant in the SCM, miR-668-3p, miR-6757-5p, and miR-6812-5p were not contained in the tissue atlas (Supplemental Fig. 2). The majority of other miRNAs identified in the SCM was found in a wide variety of different tissues. We also observed SCM miRNAs that were rather tissue specific, like miR-22-5p (Fig. 3), which is most abundant in muscles. Other examples include let-7f-5p and let-7a-5p, which are found in the SCM and which are mostly present in different areas of the brain. We conclude that miRNAs found in the SCM showed a high heterogeneity with respect to the tissues of origin.

Since spermatozoa miRNAs play a crucial function in the control of the transcriptomic homeostasis in fertilized eggs, zygotes and two-cell embryos^34^, we computed the overlap between spermatozoa miRNAs and miRNAs in the embryonic culture media. Out of 101 miRNAs that were previously reported being highly expressed in human spermatozoa, 83 (82.2%) were also discovered in the SCM (see Supplemental Table 4). These data possibly indicate that miRNAs found in the SCM might be attributed to spermatozoa.

**Figure 4:**
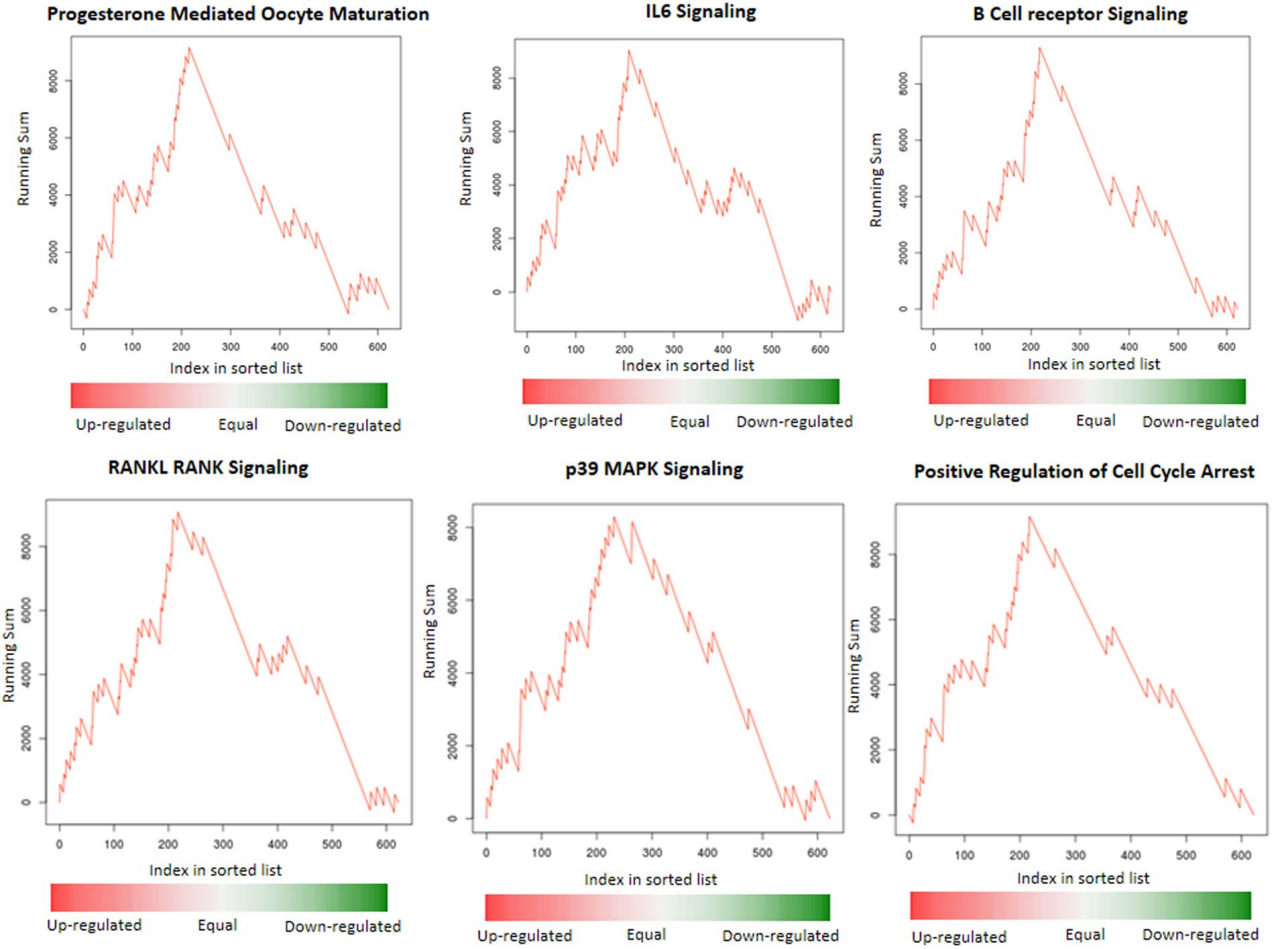
Pathway enrichment analysis: the plots show the running sum of a miRNA set enrichment analysis. MiRNAs are sorted with respect to a decreasing fold-change. The sorted list is processed from left to right. For each miRNA that is found in a pathway the running sum is increased, otherwise it is decreased. The strongly increasing curves in the beginning indicate up-regulated miRNAs while the decreasing curves at the end indicate down-regulated miRNAs.

To obtain a first inside into regulatory effects of miRNAs, we analyzed the 621 SCM miRNAs found in the SCM using our software miEAA that calculates whether miRNAs belong to a pathway, gene ontology, organ or any other functional category. We compared miRNAs that are most significantly up-regulated in samples not leading to pregnancy with miRNAs samples up-regulated in samples associated with an established pregnancy. In total, we investigated 13,962 categories. Following adjustment for multiple testing, 777 of them (5.6%), remained significant. Decreasing the alpha level to 0.01 still left 29 pathways and categories. The complete list together with the involved miRNAs is presented in Supplemental Table 5. Figure 4 highlights prominent examples including miRNAs that are most significantly up-regulated in samples that were not associated with a pregnancy and that are significantly enriched on the progesterone mediated oocyte maturation pathway. Other miRNAs not associated with pregnancy are enriched in the IL6 signaling, B cell receptor signaling, positive regulation of cell cycle arrest, p39 signaling and the RANKL RANK signaling cascade.

### Pathway analysis

**Figure 5:**
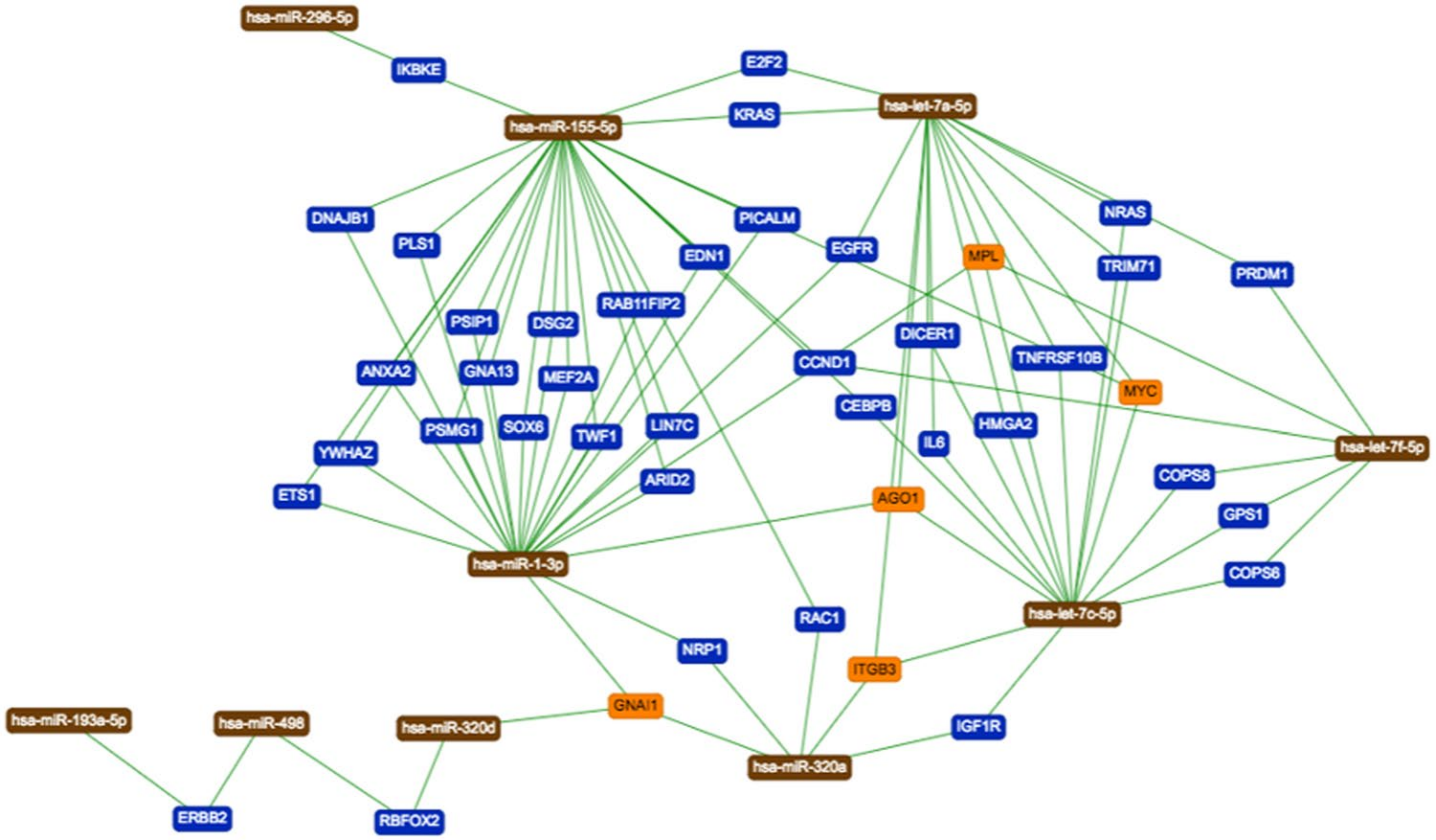
Network of dys-regulated miRNAs and target genes. MiRNAs are shown as brown nodes, genes targeted by at two miRNAs as blue and genes targeted by at least three miRNAs as orange nodes.

To gain a more complete insight in the regulation of miRNAs we performed a network analysis. First, we selected those genes that are up- or down-regulated in one of the two groups. As criterion we selected a t-test *P*-value threshold 0 f 0.05 and additionally required that the miRNAs were altered in the expression in one of both groups by at least 20%. For these miRNAs we constructed the target gene network which is presented in Fig. 5. In this network, miRNAs are represented as brown nodes, genes targeted by two miRNAs in blue and by three or more miRNAs in orange. Genes only targeted by a single miRNA were not represented. Our analysis highlighted four central genes: ITGB3, GNAI1, AGO1 and MPL. These genes were targeted by six miRNAs: hsa-let-7a-5p, hsa-miR-1-3p, hsa-miR-320d, hsa-miR-320a, hsa-let-7c-5p and hsa-let-7f-5p. Importantly, all these miRNAs were up-regulated in the group of women with negative outcome. We also asked on whether the genes in Fig. 5 are enriched for specific gene ontologies. The most significant category from gene ontologies biological processes was the positive regulation of epithelial cell proliferation (adjusted *P-*value of 9.5 × 10^−7^). The most significant pathway from the NCI pathway interaction database was the SHP2 signaling cascade (adjusted *P-*value of 6.1 × 10^−7^). With respects to pathways from Reactome, three categories achieved the most significant *P-*value of adjusted *P-*value of 6 × 10^−6^: GRB2 events in ERBB2 signaling, SHC1 events in ERBB2 signaling and Signal transduction by L1. These categories are down-regulated via an up-regulation of the miRNAs in women not leading to positive pregnancy after IVF.

### The potential role of microvesicles in the SCM

**Figure 6:**
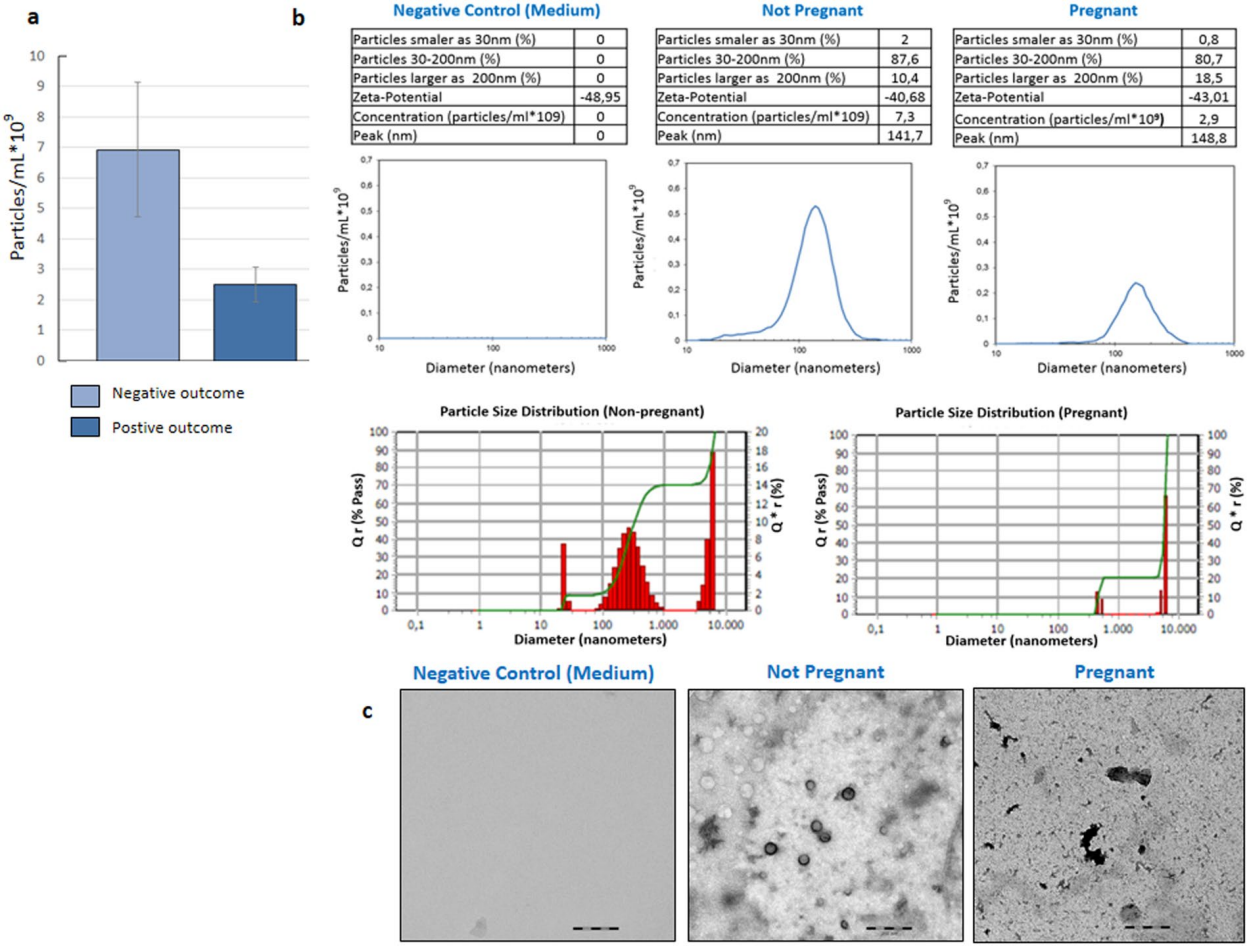
Results of three measurements of Nanoparticle Tracking Analysis. (**a**) Number of counted particles from embryos leading to successful pregnancies and from failed pregnancies. (**b**) Mean and standard deviation of particles in samples from pregnancies, from failed pregnancies and from negative controls culture media. (**c**) Electron microscopy images of the negative control, pregnant and non-pregnant groups.

A possible explanation for the decreased repertoire of miRNAs in the SCM of samples leading to pregnancy may be an altered number of extracellular vehicles (EVs) secreted by blastocysts. To get first insights into the role of EV population, e.g. exosomes or microvesicles, we performed a small Proof-of-Concept study on eight SCM samples. We observed a small difference in the overall size distribution between of EVs of samples leading to pregnancy and EVs of negative samples with fold changes of 0.74 and 1.24, respectively. The total number of EVs was decreased by 1.93 fold in the SCM of female with positive outcome. For these positive outcomes, 3.8 × 10^9^ EVs per mL were detected, while in the SCM of females with negative outcome, 7.35 × 10^9^ EVs were detected (Fig. 6a **)**. In pure culture media i.e. media not exposed to blastocysts, EVs were not detected. Representative results for the positive outcome, the negative outcome and the control samples are provided in Fig. 6b. The observed results correlate with the overall decreased miRNA repertoire in the SCM of samples leading to a pregnancy. Figure 6c shows a representative electron microscopy image of EVs in the SCM that were exposed to blastocysts.

## Discussion

As of now, very few studies have been conducted on SCM suggesting that the embryo quality and development are correlated with the miRNA expression^17,18^. In the present study, we used miRNA microarray and RT-qPCR to identify the miRNAs, which are contained in the SCM of couples undergoing fertility treatment. A total of 621 miRNAs were identified in 56 samples. Out of these miRNAs, 149 were detected in each female with pregnancies and 163 miRNAs in females with negative outcome. The SCM from embryos leading to a pregnancy following implantation have a decreased repertoire of miRNAs. One single miR-634 was correlated with a successful outcome with an accuracy of 71% and a sensitivity of 85%. Based on the miRNA expression intensity, miR-29c-3p was correlated with a positive outcome with an AUC value of 0.83. MiR-29c-3p is a multifunctional miRNA implicated in several processes including extracellular remodeling and angiogenesis^35^. Dysregulation of miR-29c-3p was previously shown in the endometriotic tissues^35^. Among the most significant miRNAs in our study, we found miR-30c, which is derived from trophectoderm cells and which was previously described to be significant in blastocyst culture medium as candidate for human embryo reproductive competence assessment^36^. Notable, among the highest expressed miRNAs were miRNAs that had been added to later versions of the miRBase. In detail, hsa-miR-1246 had been added in version 11 and hsa-miR-6089 to version 19. As we recently indicated miRNAs that have been added to later miRBase versions likely do not represent true miRNAs^32^. Likewise, miR-6757-5p and miR-6812-5p that were not included in our miRNA tissue Atlas that contains 982 complete miRNomes, may represent miRNAs that are erroneously annotated in miRBase. As for miRNA miR-668-3p, that was also not included in the miRNA tissue Atlas but was added to an earlier version of miRBase, this miRNA was significantly dysregulated in the myocardial regenerative process of 1- to 7-day-old neonatal mouse^37^.

Our miRNA profiling is consistent with previously reported data showing that sperm miRNAs are important for fertilization and preimplantation embryos^34^. We found a considerable overlap of 101 shared miRNAs between the SCM and human spermatozoa^10^. Further investigations are needed to prove the importance of these miRNAs in the development of preimplantation embryos. As for the downstream effects of the identified miRNAs, our results identified pathways that may be relevant to the development such as progesterone-mediated oocyte maturation. For the latter pathway, we observe a statistically significant accumulation of miRNAs that are highly abundant in the SCM samples not leading to pregnancy. These miRNAs were also enriched in processes like IL6 signaling, B cell receptor signaling, and the RANKL RANK signaling cascade. Growing evidence supports the involvement of hepatoma-derived growth factor (HDGF) in the early embryo development. The effect can largely enhance the vascular endothelial growth factor (VEGF), which plays a major role in the angiogenesis process^38^. Similarly, Cyclin T2 (CCNT2), is involved in muscle differentiation^39^ and plays an essential role in the early embryogenesis, development processes^40^, and in the early steps of spermatogenesis^41^. A pathway analysis has shown clear involvement of the miRNAs in biochemical pathways. A potential flaw of respective analyses is a bias towards heavily studied genes for which more miRNAs targeting those genes are known. Thus, it is important to discuss findings taking a potential bias into account. One important factor is that the pathways and categories that we describe in the results are correlated to fertility related processes. The maybe most stinking finding is the regulation of epithelial cell proliferation^42,43^. Therefore, an understanding of the miRNAs related pathways central to epithelial cell proliferation, differentiation and/or implantation have the potential to alleviate many problems associated with successful pregnancy and/or improve the outcome of IVF. Finally, we would like to caution about two issues. First, despite promising initial results with miRNA biomarkers, there are still no circulating miRNA biomarker established in clinical routine testing. One likely reason is the lack of specificity of single markers, as described by Haider and co-workers^44^. While combinations of markers can add the specificity to the miRNA-disease patterns^7^, the results of miRNA profiles vary dependent on the used technology^24^ and sample handling^20^. As indicated above the value of miRNA biomarker can further be compromised by erroneously annotated miRNAs in miRBase^32^ calling for carefully curated databases such as miRGeneDB^45^. These and other challenges influencing the translational process of miRNAs have been recently summarized^46^. Second, we measured a very large number of features in a comparably small cohort of samples (p > n problem) with a single miRNA (miR-29c-3p) remaining significant following adjustment for multiple testing. This miRNA was described as a biomarker for different cancers including head-and-neck cancer^47^. Nonetheless, the respective single miRNA has no sufficient predictive power. In addition, as addressed above, single miRNAs are usually not specific for a health condition. This requires statistical learning approaches to build specific combinations of miRNAs. In order to avoid over-fitting, larger cohorts are required to develop miRNA biomarkers that can be used with a high predictive power in a clinical setting.

Our analysis indicates a possible link between the decreased miRNA repertoire and the decreased EVs in the SCM of samples leading to pregnancy. However, a comprehensive analysis of the miRNAs and the EVs in SCM samples is rather challenging. Although many methods are available to isolate and characterize differentially sized microvesicles^48–52^, the available isolation methods do not necessarily provide good quality and quantity EVs for ECM samples^48–52^. The protocols are most often used for serum/plasma^52–54^, urine^48,55^, and/or CSF^56^ samples. The microvesicle content in these liquids is likely much higher, as they are in constant contact with myriads of body cells, compared to the ECM, where the microvesicles can be released only from the embryo itself.

In conclusion, we identified miRNAs contained in the SCM of couples undergoing fertility treatment. The SCM from embryos leading to a pregnancy following implantation have a decreased repertoire of miRNAs.

## Electronic supplementary material

Supplementay files
