# Supplementary material for: Micro-ribonucleic acids and extracellular vesicles repertoire in the spent culture media is altered in women undergoing In Vitro Fertilization
Source: Sci Rep. 2017 Oct 19;7:13525. doi: 10.1038/s41598-017-13683-8 (PMC5648749; doi:10.1038/s41598-017-13683-8)
Supplement: Supplementary file 1 — Supplementay files [file 41598_2017_13683_MOESM1_ESM.pdf]

Micro-ribonucleic acids and extracellular vesicles repertoire in the spent culture media is altered in women undergoing *In Vitro Fertilization*

Masood Abu-Halima[1,*], Sebastian Häusler[2,*], Christina Backes[3], Tobias Fehlmann[3], Claudia Staib[2], Sigrun Nestel[4], Irina Nazarenko[5], Eckart Meese[1], Andreas Keller[3]

[1] Department of Human Genetics, Saarland University, 66421 Homburg /Saar, Germany

[2] Department of Obstetrics and Gynaecology, University of Würzburg, School of Medicine, 97080 Würzburg, Germany

[3] Chair for Clinical Bioinformatics, Saarland University, 66041 Saarbruecken, Germany

[4] Global Head of Fertility Technologies, Merck KGaA, 64293 Darmstadt, Germany

[4] Institute of Anatomy and Cell Biology, University of Freiburg, 79085 Freiburg, Germany

[5] AG Exosomes and Tumor biology, 79085 Freiburg, Germany

* Both authors contributed equally and are listed in alphabetic order

Corresponding Author

Dr. rer. nat. Masood Abu-Halima

Institute of Human Genetics

Saarland University Medical Center

Kirrberger Straße 60

66421 Homburg/Saar, Germany

Tel.: +49 6841 16- 26289

Fax: +49 6841 16- 26251

E-Mail: masood@daad-alumni.de

Supplementary Figure 1

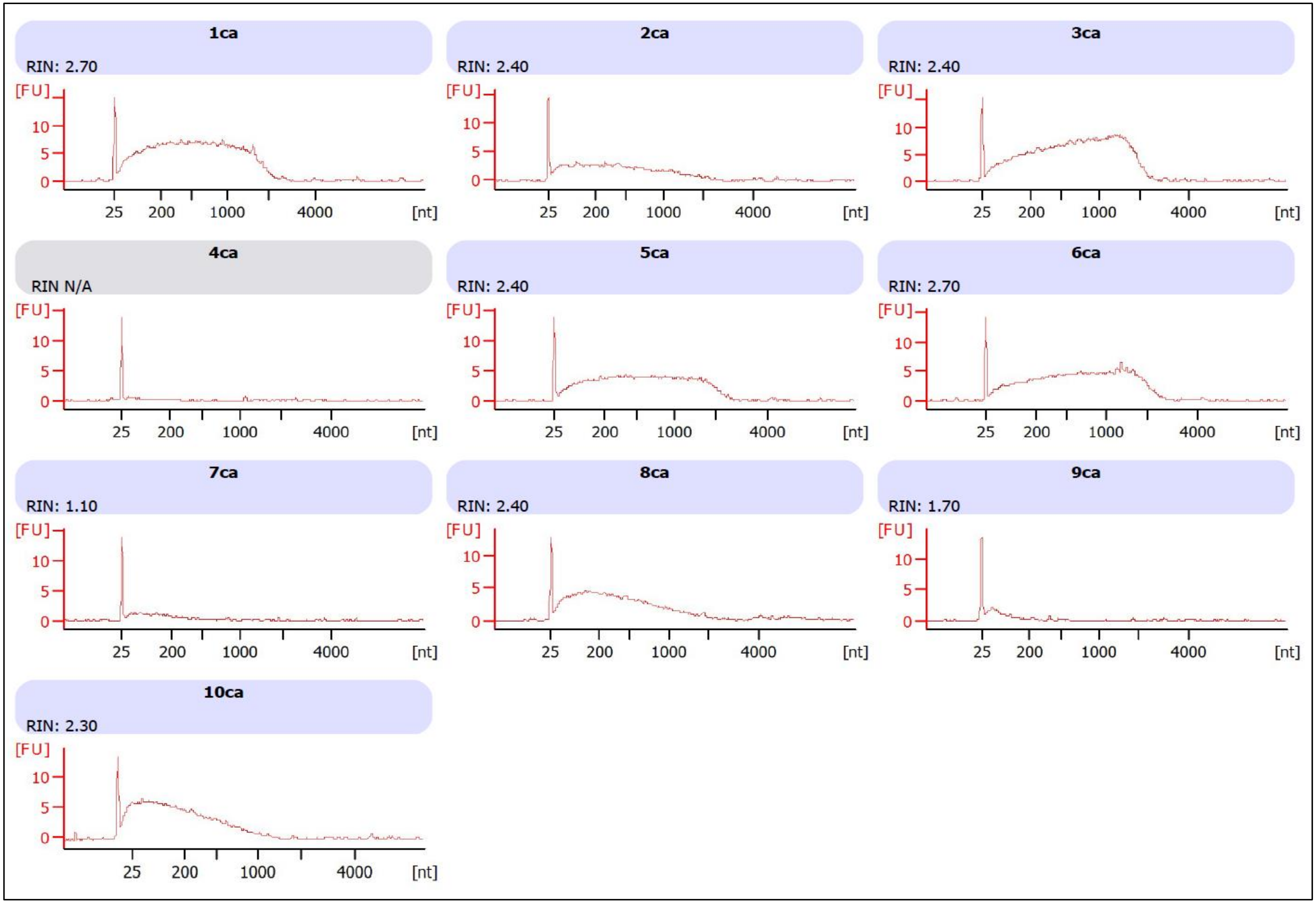

Supplementary Figure 2

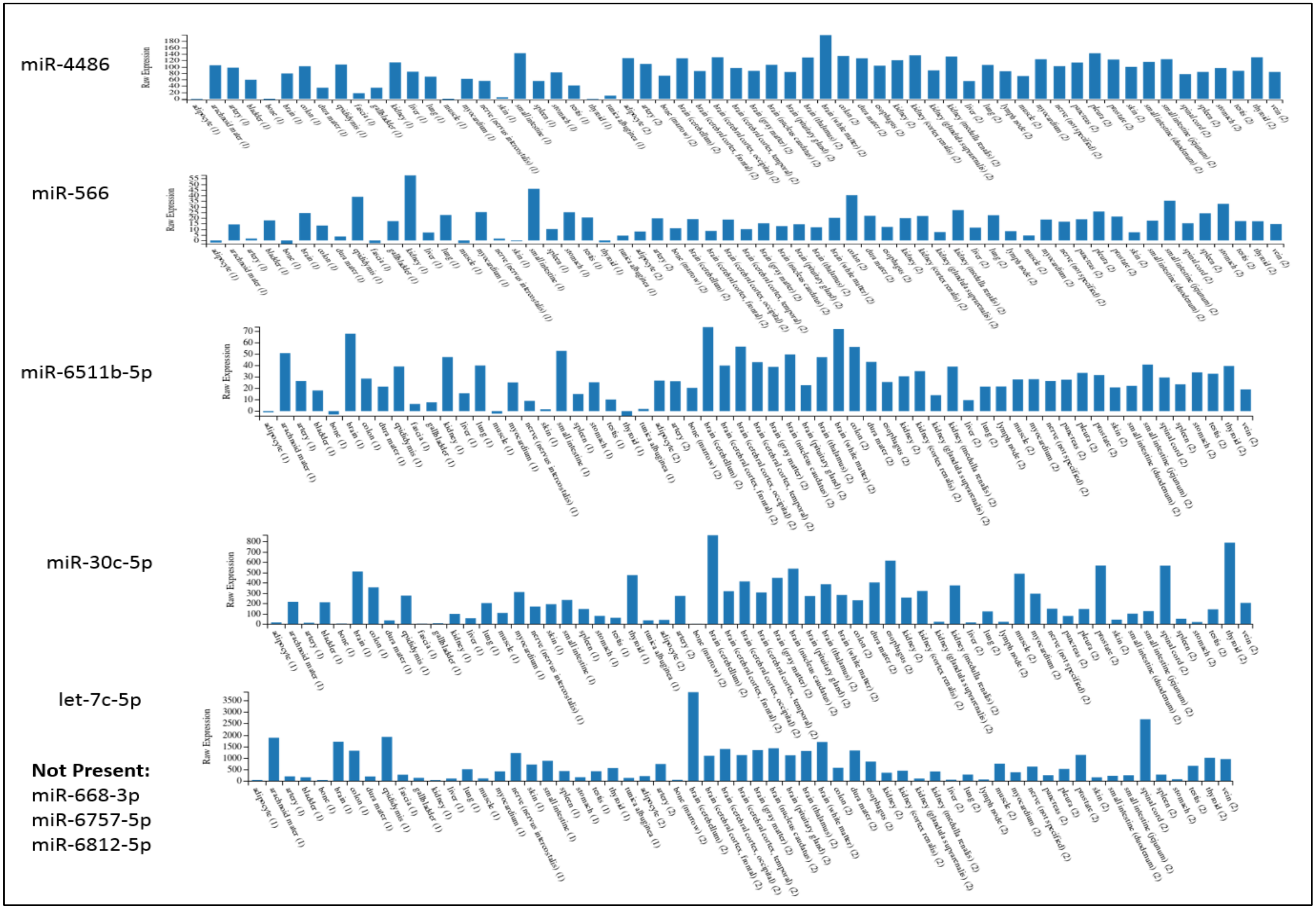

Supplemental Table 1: RNA concentrations and A260:A280 ratio observed in all embryonic culture media samples. Measured by Nanodrop spectrophotometer

Sample ID	Nucleic Acid Conc.	Unit	260/280	Sample Type
108/2	30.3	ng/µl	1.7	RNA
108/3	23.8	ng/µl	1.75	RNA
113/4	19.3	ng/µl	1.78	RNA
114/3	28.4	ng/µl	1.66	RNA
117/1	17.6	ng/µl	1.71	RNA
117/2	16.8	ng/µl	1.7	RNA
117/3	21.3	ng/µl	1.83	RNA
117/5	20.6	ng/µl	1.75	RNA
131/2	19	ng/µl	1.77	RNA
132/1	20.6	ng/µl	1.77	RNA
132/3	22.1	ng/µl	1.67	RNA
133/2	19.9	ng/µl	1.72	RNA
133/4	15.9	ng/µl	1.85	RNA
138/1	14.3	ng/µl	1.86	RNA
147/1	18.7	ng/µl	1.81	RNA
147/3	21.3	ng/µl	1.8	RNA
149/1	25.3	ng/µl	1.89	RNA
149/2	20.4	ng/µl	1.96	RNA
158/4	19.3	ng/µl	1.71	RNA
158/6	18.1	ng/µl	1.65	RNA
172/9	23	ng/µl	1.71	RNA
181/0	13.5	ng/µl	1.89	RNA
181/3	22.4	ng/µl	2.03	RNA
183/6	14	ng/µl	1.96	RNA
189/3	20.8	ng/µl	1.73	RNA
189/4	24.5	ng/µl	1.68	RNA
190/1	15.3	ng/µl	1.8	RNA
191/2	25.3	ng/µl	1.67	RNA
191/3	12.3	ng/µl	2.03	RNA
205/1	26.6	ng/µl	1.67	RNA
205/4	22.1	ng/µl	1.74	RNA
214/1	25.8	ng/µl	1.69	RNA
219/1	24.9	ng/µl	1.65	RNA
219/2	20.4	ng/µl	1.87	RNA

223/5	22.6	ng/µl	1.83	RNA
224/1	19.5	ng/µl	1.96	RNA
230/10	24.7	ng/µl	1.67	RNA
230/6	25	ng/µl	1.67	RNA
234/3	24.6	ng/µl	1.66	RNA
235/1	24.9	ng/µl	1.78	RNA
235/4	20.5	ng/µl	1.69	RNA
244/2	22.4	ng/µl	1.78	RNA
256/1	18.1	ng/µl	1.83	RNA
258/6	17.1	ng/µl	1.85	RNA
27//1	23.8	ng/µl	1.77	RNA
27//2	26.7	ng/µl	1.71	RNA
27//5	18.7	ng/µl	1.81	RNA
273/2	17.4	ng/µl	1.82	RNA
274/1	19.1	ng/µl	1.79	RNA
277/2	20	ng/µl	1.83	RNA
290/5	15.3	ng/µl	1.86	RNA
291/1	20.7	ng/µl	1.81	RNA
291/2	17.3	ng/µl	1.85	RNA
304/1	19.3	ng/µl	1.82	RNA
88/2	21.5	ng/µl	1.85	RNA
88/6	14.8	ng/µl	1.9	RNA

Supplemental Table 2: MiRNA abundance in embryonic culture media

miRNA	Median Group 1	Median Group 2	STDV Group 1	STDV Group 2	Fold change	Log(2) fold change	Wilcoxon Mann Whitney Test raw p-Value	Wilcoxon Mann Whitney Test adjusted p-Value	t-Test raw p-Value	t-Test adjusted p-Value	AUC
hsa-miR-29c-3p	2.13	1.86	0.51	0.17	1.15	0.20	0.0001	0.0736	0.000031	0.0193	0.17
hsa-miR-22-5p	1.80	1.35	0.38	0.24	1.33	0.41	0.0067	0.1644	0.0008	0.1192	0.27
hsa-miR-4486	3.17	2.59	1.95	0.67	1.23	0.30	0.0028	0.1269	0.0007	0.1192	0.25
hsa-miR-566	1.48	1.85	0.38	0.25	0.80	-0.32	0.0038	0.1304	0.0007	0.1192	0.75
hsa-let-7a-5p	1.71	1.20	1.08	0.46	1.43	0.51	0.0158	0.1644	0.0054	0.1911	0.29
hsa-let-7c-5p	1.53	0.93	0.53	0.43	1.65	0.72	0.0079	0.1644	0.0034	0.1911	0.27
hsa-let-7f-5p	1.87	1.16	1.32	0.50	1.61	0.69	0.0200	0.1652	0.0039	0.1911	0.30
hsa-miR-30c-5p	1.63	1.99	0.31	0.20	0.82	-0.29	0.0106	0.1644	0.0031	0.1911	0.72

hsa-miR-4253	1.97	1.44	0.57	0.42	1.37	0.45	0.0035	0.1269	0.0034	0.1911	0.25
hsa-miR-485-3p	1.65	2.21	0.39	0.64	0.75	-0.42	0.0010	0.1269	0.0058	0.1911	0.78
hsa-miR-584-5p	2.50	2.09	0.90	0.60	1.19	0.26	0.0161	0.1644	0.0057	0.1911	0.30
hsa-miR-6511b-5p	1.51	1.10	0.53	0.36	1.37	0.46	0.0114	0.1644	0.0026	0.1911	0.29
hsa-miR-668-3p	1.40	1.95	0.40	0.36	0.72	-0.48	0.0010	0.1269	0.0019	0.1911	0.78
hsa-miR-6726-5p	1.22	0.83	0.58	0.43	1.47	0.55	0.0068	0.1644	0.0047	0.1911	0.27
hsa-miR-6757-5p	1.67	1.42	0.87	0.50	1.17	0.23	0.0093	0.1644	0.0026	0.1911	0.28
hsa-miR-6760-5p	2.65	1.17	1.28	0.91	2.27	1.18	0.0136	0.1644	0.0056	0.1911	0.29
hsa-miR-6812-5p	4.52	3.56	2.35	1.12	1.27	0.34	0.0114	0.1644	0.0028	0.1911	0.29
hsa-miR-6883-3p	1.58	1.98	0.33	0.26	0.80	-0.32	0.0075	0.1644	0.0046	0.1911	0.72
hsa-miR-875-3p	1.04	0.91	0.25	0.15	1.14	0.19	0.0167	0.1644	0.0049	0.1911	0.30
hsa-miR-4685-5p	1.38	0.94	0.72	0.45	1.47	0.56	0.0433	0.2258	0.0066	0.2034	0.33
hsa-miR-3191-5p	1.49	1.89	0.43	0.36	0.79	-0.35	0.0093	0.1644	0.0079	0.2065	0.72
hsa-miR-4326	1.47	1.86	0.40	0.49	0.79	-0.34	0.0034	0.1269	0.0071	0.2065	0.75
hsa-miR-631	2.01	2.24	0.36	0.29	0.90	-0.16	0.0126	0.1644	0.0079	0.2065	0.71
hsa-miR-8060	1.80	1.01	4.04	0.47	1.79	0.84	0.0028	0.1269	0.0080	0.2065	0.25
hsa-let-7g-5p	1.51	1.31	0.56	0.32	1.15	0.20	0.0560	0.2432	0.0109	0.2068	0.34
hsa-miR-1296-5p	1.62	2.07	0.60	0.37	0.78	-0.35	0.0022	0.1269	0.0098	0.2068	0.76
hsa-miR-155-5p	1.15	0.91	0.70	0.28	1.26	0.33	0.0114	0.1644	0.0124	0.2068	0.29
hsa-miR-184	1.69	1.43	0.59	0.24	1.18	0.24	0.1175	0.3333	0.0120	0.2068	0.37
hsa-miR-196b-3p	1.26	1.73	0.40	0.63	0.73	-0.46	0.0020	0.1269	0.0104	0.2068	0.76
hsa-miR-26b-5p	1.43	1.31	0.70	0.23	1.09	0.13	0.0537	0.2416	0.0112	0.2068	0.34
hsa-miR-3137	1.39	1.03	0.90	0.56	1.36	0.44	0.0159	0.1644	0.0140	0.2068	0.30
hsa-miR-331-3p	1.94	2.20	0.32	0.23	0.88	-0.18	0.0253	0.1871	0.0155	0.2068	0.69
hsa-miR-346	1.74	2.25	0.50	0.36	0.77	-0.37	0.0178	0.1649	0.0135	0.2068	0.70
hsa-miR-34b-3p	1.79	2.08	0.40	0.50	0.86	-0.22	0.0129	0.1644	0.0119	0.2068	0.71
hsa-miR-371a-5p	2.08	1.52	1.88	0.48	1.37	0.45	0.0034	0.1269	0.0157	0.2068	0.26
hsa-miR-3917	1.84	1.66	0.47	0.35	1.11	0.15	0.0301	0.1988	0.0155	0.2068	0.32
hsa-miR-4706	1.52	1.22	0.74	0.41	1.25	0.32	0.0237	0.1796	0.0145	0.2068	0.31
hsa-miR-4743-5p	0.97	0.77	0.49	0.30	1.26	0.33	0.0129	0.1644	0.0143	0.2068	0.29
hsa-miR-5006-5p	2.87	2.31	0.78	0.66	1.24	0.31	0.0210	0.1652	0.0135	0.2068	0.30
hsa-miR-5196-5p	1.87	1.45	0.85	0.50	1.29	0.37	0.0678	0.2598	0.0127	0.2068	0.34

hsa-miR-550a-3-5p	1.28	0.99	0.76	0.31	1.29	0.37	0.0139	0.1644	0.0134	0.2068	0.29
hsa-miR-6739-5p	1.36	1.11	0.99	0.49	1.23	0.30	0.0469	0.2327	0.0122	0.2068	0.33
hsa-miR-6776-5p	2.45	1.35	0.98	0.74	1.81	0.86	0.0259	0.1871	0.0148	0.2068	0.31
hsa-miR-6792-5p	1.05	0.75	0.68	0.29	1.39	0.48	0.0290	0.1960	0.0146	0.2068	0.31
hsa-miR-6803-3p	1.59	1.96	0.43	0.43	0.81	-0.30	0.0151	0.1644	0.0125	0.2068	0.70
hsa-miR-744-3p	1.82	2.21	0.54	0.45	0.83	-0.28	0.0178	0.1649	0.0141	0.2068	0.70
hsa-miR-8071	1.65	1.16	0.63	0.52	1.43	0.51	0.0143	0.1644	0.0151	0.2068	0.29
hsa-miR-1236-3p	1.71	2.04	0.33	0.34	0.84	-0.25	0.0205	0.1652	0.0186	0.2086	0.70
hsa-miR-193a-5p	1.41	0.97	1.08	0.61	1.45	0.54	0.0195	0.1652	0.0180	0.2086	0.30
hsa-miR-193b-5p	0.93	0.68	0.67	0.45	1.37	0.45	0.0136	0.1644	0.0166	0.2086	0.29
hsa-miR-2276-3p	1.78	1.39	0.71	0.41	1.28	0.35	0.0460	0.2320	0.0183	0.2086	0.33
hsa-miR-296-5p	2.88	4.08	0.98	0.90	0.71	-0.50	0.0069	0.1644	0.0174	0.2086	0.73
hsa-miR-30d-5p	2.46	2.20	0.32	0.27	1.12	0.16	0.0210	0.1652	0.0186	0.2086	0.30
hsa-miR-4306	0.90	0.69	0.47	0.30	1.31	0.39	0.0560	0.2432	0.0192	0.2086	0.34
hsa-miR-451a	2.41	2.34	1.25	0.43	1.03	0.04	0.1127	0.3287	0.0181	0.2086	0.37
hsa-miR-6892-5p	1.59	1.18	1.13	0.54	1.34	0.43	0.0340	0.2085	0.0191	0.2086	0.32
hsa-miR-7843-3p	1.63	1.81	0.33	0.36	0.90	-0.15	0.0068	0.1644	0.0172	0.2086	0.73
hsa-miR-6076	13.07	7.58	22.58	13.86	1.72	0.79	0.0119	0.1644	0.0208	0.2224	0.29
hsa-miR-6778-3p	1.48	1.98	0.49	0.44	0.75	-0.42	0.0195	0.1652	0.0212	0.2233	0.70
hsa-miR-1-3p	1.15	0.85	1.39	0.64	1.36	0.44	0.0301	0.1988	0.0250	0.2350	0.32
hsa-miR-3591-3p	1.87	2.30	0.42	0.70	0.82	-0.29	0.0042	0.1337	0.0248	0.2350	0.74
hsa-miR-4324	1.71	1.82	0.29	0.19	0.94	-0.08	0.0452	0.2320	0.0248	0.2350	0.67
hsa-miR-454-5p	2.22	2.41	0.33	0.39	0.92	-0.12	0.0210	0.1652	0.0245	0.2350	0.70
hsa-miR-498	2.34	1.81	0.58	0.48	1.29	0.37	0.0422	0.2258	0.0233	0.2350	0.33
hsa-miR-6754-5p	1.03	0.67	0.53	0.27	1.54	0.62	0.0762	0.2789	0.0240	0.2350	0.35
hsa-miR-6852-3p	1.38	1.53	0.34	0.28	0.91	-0.14	0.0158	0.1644	0.0239	0.2350	0.71
hsa-miR-3937	2.78	1.87	0.92	0.86	1.49	0.57	0.0290	0.1960	0.0262	0.2432	0.31
hsa-miR-3124-5p	1.53	1.38	0.81	0.55	1.11	0.15	0.0975	0.3185	0.0283	0.2439	0.36
hsa-miR-3622b-5p	0.91	0.57	0.56	0.38	1.60	0.68	0.0387	0.2227	0.0275	0.2439	0.32
hsa-miR-4446-5p	1.46	1.75	0.38	0.80	0.83	-0.26	0.0006	0.1269	0.0279	0.2439	0.78
hsa-miR-4669	2.00	1.57	0.99	0.68	1.28	0.35	0.0664	0.2598	0.0276	0.2439	0.34
hsa-miR-6873-5p	1.07	0.97	0.35	0.19	1.10	0.14	0.1607	0.4056	0.0269	0.2439	0.38

hsa-miR-320d	1.15	0.90	0.63	0.39	1.28	0.35	0.1128	0.3287	0.0291	0.2467	0.37
hsa-miR-4298	3.49	2.75	1.63	1.20	1.27	0.34	0.0452	0.2320	0.0294	0.2467	0.33
hsa-miR-1185-1-3p	1.72	1.53	0.64	0.40	1.13	0.17	0.1049	0.3275	0.0328	0.2469	0.36
hsa-miR-3156-5p	1.54	1.29	0.60	0.38	1.19	0.25	0.0922	0.3045	0.0316	0.2469	0.36
hsa-miR-4257	2.51	1.75	1.34	0.85	1.44	0.53	0.0500	0.2365	0.0315	0.2469	0.33
hsa-miR-4433a-5p	4.01	3.17	1.53	1.16	1.26	0.34	0.0422	0.2258	0.0319	0.2469	0.33
hsa-miR-4646-5p	0.83	0.66	0.73	0.31	1.26	0.33	0.1011	0.3249	0.0330	0.2469	0.36
hsa-miR-4713-3p	1.81	1.77	1.13	0.44	1.02	0.03	0.2422	0.5204	0.0321	0.2469	0.40
hsa-miR-4714-5p	2.32	2.14	0.35	0.27	1.08	0.11	0.0332	0.2085	0.0323	0.2469	0.32
hsa-miR-6509-3p	1.91	2.38	0.54	0.44	0.80	-0.32	0.0340	0.2085	0.0324	0.2469	0.68
hsa-miR-877-5p	1.03	0.89	0.48	0.27	1.17	0.22	0.0521	0.2379	0.0300	0.2469	0.33
hsa-miR-4738-3p	2.41	1.78	0.74	0.62	1.35	0.43	0.0532	0.2410	0.0339	0.2503	0.34
hsa-miR-1226-3p	1.18	1.31	0.29	0.22	0.90	-0.15	0.0315	0.2019	0.0346	0.2518	0.68
hsa-miR-320a	1.83	1.49	0.65	0.51	1.23	0.29	0.0543	0.2425	0.0351	0.2518	0.34
hsa-miR-6789-3p	1.42	1.76	0.48	0.30	0.81	-0.31	0.0165	0.1644	0.0353	0.2518	0.70
hsa-miR-1914-5p	1.45	1.85	0.48	0.42	0.78	-0.35	0.0215	0.1670	0.0363	0.2559	0.70
hsa-miR-6130	1.02	0.85	0.46	0.31	1.20	0.26	0.0493	0.2355	0.0369	0.2577	0.33
hsa-miR-4290	2.16	2.42	0.47	1.04	0.89	-0.17	0.0047	0.1337	0.0391	0.2692	0.74
hsa-miR-6741-5p	2.42	1.56	0.87	0.85	1.55	0.63	0.0165	0.1644	0.0399	0.2692	0.30
hsa-miR-6829-5p	1.51	1.26	0.67	0.50	1.20	0.26	0.0489	0.2355	0.0398	0.2692	0.33
hsa-miR-6086	1.24	0.94	1.17	0.57	1.32	0.40	0.1030	0.3263	0.0416	0.2777	0.36
hsa-miR-6887-3p	1.91	2.29	0.42	0.36	0.83	-0.26	0.0510	0.2365	0.0431	0.2845	0.67
hsa-miR-4271	3.37	3.12	2.81	1.12	1.08	0.11	0.1168	0.3333	0.0455	0.2943	0.37
hsa-miR-4514	1.01	0.71	0.93	0.40	1.42	0.50	0.0609	0.2471	0.0455	0.2943	0.34
hsa-miR-3685	1.81	1.93	0.34	0.30	0.94	-0.09	0.0678	0.2598	0.0469	0.2974	0.66
hsa-miR-4758-3p	3.94	3.37	1.31	0.65	1.17	0.22	0.2025	0.4574	0.0465	0.2974	0.39
hsa-miR-1287-5p	2.70	2.37	1.19	0.64	1.14	0.18	0.1011	0.3249	0.0499	0.3038	0.36
hsa-miR-146a-5p	1.28	1.17	0.31	0.16	1.09	0.13	0.1169	0.3333	0.0486	0.3038	0.37
hsa-miR-550a-5p	2.94	2.66	0.96	0.62	1.11	0.15	0.0922	0.3045	0.0497	0.3038	0.36
hsa-miR-6879-3p	1.33	1.65	0.40	0.50	0.81	-0.31	0.0396	0.2235	0.0497	0.3038	0.67
hsa-miR-6740-5p	1.75	1.30	2.41	0.97	1.34	0.43	0.0460	0.2320	0.0522	0.3105	0.33
hsa-miR-6751-3p	1.71	2.14	0.38	0.37	0.80	-0.32	0.0253	0.1871	0.0525	0.3105	0.69

hsa-miR-6856-3p	1.50	1.55	0.24	0.32	0.97	-0.04	0.1088	0.3275	0.0525	0.3105	0.64
hsa-let-7g-3p	1.13	1.35	0.31	0.32	0.83	-0.26	0.0472	0.2327	0.0537	0.3141	0.67
hsa-miR-466	1.53	1.78	0.32	0.26	0.86	-0.22	0.1053	0.3275	0.0541	0.3141	0.64
hsa-miR-107	1.58	1.56	0.70	0.23	1.01	0.02	0.1756	0.4228	0.0549	0.3158	0.38
hsa-miR-103a-3p	1.34	1.16	0.47	0.28	1.16	0.22	0.0638	0.2525	0.0560	0.3188	0.34
hsa-miR-6867-5p	1.00	0.88	0.81	0.60	1.13	0.18	0.1175	0.3333	0.0565	0.3191	0.37
hsa-miR-4688	1.52	1.33	0.52	0.26	1.14	0.19	0.1728	0.4228	0.0593	0.3257	0.38
hsa-miR-6762-3p	1.39	1.59	0.36	0.31	0.88	-0.19	0.0433	0.2258	0.0590	0.3257	0.67
hsa-miR-6873-3p	1.75	1.87	0.29	0.26	0.93	-0.10	0.0422	0.2258	0.0586	0.3257	0.67
hsa-miR-623	1.10	0.88	0.49	0.41	1.26	0.33	0.0746	0.2762	0.0617	0.3361	0.35
hsa-miR-106b-5p	0.99	0.87	0.84	0.28	1.14	0.19	0.0777	0.2789	0.0666	0.3544	0.35
hsa-miR-1250-5p	1.11	1.01	0.37	0.20	1.10	0.14	0.0678	0.2598	0.0708	0.3544	0.34
hsa-miR-1295a	0.82	0.74	0.59	0.15	1.10	0.14	0.1189	0.3342	0.0671	0.3544	0.37
hsa-miR-142-3p	2.07	1.71	2.85	0.38	1.21	0.28	0.1645	0.4071	0.0724	0.3544	0.38
hsa-miR-16-5p	2.23	2.10	2.64	0.46	1.06	0.08	0.1785	0.4263	0.0717	0.3544	0.39
hsa-miR-20a-5p	1.25	1.20	3.65	0.54	1.04	0.05	0.1964	0.4517	0.0673	0.3544	0.39
hsa-miR-20b-3p	1.00	1.07	0.18	0.35	0.93	-0.10	0.0906	0.3045	0.0724	0.3544	0.64
hsa-miR-2277-3p	1.26	1.46	0.51	0.40	0.86	-0.22	0.0132	0.1644	0.0693	0.3544	0.71
hsa-miR-3151-3p	3.03	3.39	0.59	0.71	0.90	-0.16	0.0413	0.2258	0.0681	0.3544	0.67
hsa-miR-4632-5p	2.32	1.45	1.04	0.88	1.60	0.68	0.0691	0.2617	0.0686	0.3544	0.35
hsa-miR-6793-5p	1.45	0.99	1.30	0.63	1.46	0.55	0.0906	0.3045	0.0694	0.3544	0.36
hsa-miR-6798-5p	1.23	1.15	0.51	0.30	1.07	0.10	0.2881	0.5641	0.0725	0.3544	0.41
hsa-miR-7-2-3p	1.55	1.97	0.65	0.32	0.79	-0.34	0.0023	0.1269	0.0723	0.3544	0.76
hsa-miR-4758-5p	1.35	1.16	0.38	0.36	1.16	0.22	0.0431	0.2258	0.0740	0.3558	0.33
hsa-miR-758-3p	1.74	1.89	0.34	0.26	0.92	-0.12	0.1011	0.3249	0.0738	0.3558	0.64
hsa-miR-8064	1.40	1.21	1.81	0.48	1.16	0.21	0.0237	0.1796	0.0745	0.3558	0.31
hsa-miR-582-3p	1.21	1.29	0.26	0.49	0.94	-0.09	0.0609	0.2471	0.0755	0.3580	0.66
hsa-miR-218-1-3p	0.88	1.14	0.26	0.38	0.77	-0.38	0.0733	0.2743	0.0767	0.3608	0.65
hsa-miR-6860	2.32	1.44	0.87	0.88	1.61	0.69	0.1088	0.3275	0.0781	0.3637	0.36
hsa-miR-92b-3p	2.99	2.77	0.73	0.51	1.08	0.11	0.2255	0.4932	0.0785	0.3637	0.40
hsa-miR-4699-3p	1.93	2.21	0.44	0.34	0.87	-0.19	0.0339	0.2085	0.0791	0.3640	0.68
hsa-miR-6893-5p	3.22	2.55	1.05	1.03	1.26	0.34	0.0577	0.2455	0.0810	0.3697	0.34

hsa-miR-30b-5p	1.89	2.04	0.32	0.26	0.93	-0.11	0.0589	0.2471	0.0818	0.3709	0.66
hsa-miR-19a-3p	1.62	1.58	1.63	0.26	1.02	0.03	0.5446	0.8014	0.0856	0.3776	0.45
hsa-miR-4651	1.03	0.87	1.65	0.28	1.19	0.25	0.0159	0.1644	0.0863	0.3776	0.30
hsa-miR-4800-5p	1.01	0.89	0.61	0.45	1.14	0.19	0.1015	0.3249	0.0862	0.3776	0.36
hsa-miR-7846-3p	1.25	1.17	0.50	0.31	1.07	0.10	0.2080	0.4663	0.0847	0.3776	0.39
hsa-miR-937-5p	2.58	1.79	0.97	0.89	1.44	0.53	0.0601	0.2471	0.0847	0.3776	0.34
hsa-miR-18b-5p	1.29	1.13	0.25	0.20	1.14	0.19	0.0777	0.2789	0.0896	0.3887	0.35
hsa-miR-3173-5p	1.78	1.95	0.23	0.64	0.91	-0.13	0.3315	0.6108	0.0906	0.3887	0.58
hsa-miR-629-3p	1.29	1.63	0.44	0.43	0.79	-0.34	0.0922	0.3045	0.0907	0.3887	0.64
hsa-miR-29a-3p	1.98	1.92	1.35	0.28	1.03	0.05	0.4705	0.7415	0.0920	0.3913	0.44
hsa-miR-181a-2-3p	1.35	1.49	0.24	0.24	0.91	-0.14	0.0371	0.2172	0.0935	0.3925	0.68
hsa-miR-29b-3p	2.30	2.16	0.70	0.26	1.06	0.09	0.2850	0.5618	0.0933	0.3925	0.41
hsa-miR-3652	1.41	1.02	1.23	0.54	1.38	0.47	0.0808	0.2859	0.0943	0.3932	0.35
hsa-miR-136-3p	1.52	1.64	0.24	0.24	0.93	-0.11	0.1128	0.3287	0.0972	0.3938	0.63
hsa-miR-3129-3p	1.64	1.82	0.33	0.25	0.90	-0.16	0.0577	0.2455	0.0977	0.3938	0.66
hsa-miR-3682-3p	2.75	2.26	0.84	0.91	1.22	0.29	0.1068	0.3275	0.0972	0.3938	0.36
hsa-miR-4493	1.70	1.81	0.25	0.43	0.94	-0.10	0.0776	0.2789	0.0971	0.3938	0.65
hsa-miR-6514-3p	1.47	1.65	0.40	0.76	0.89	-0.17	0.0287	0.1960	0.0965	0.3938	0.68
hsa-miR-365a-5p	0.81	0.61	0.43	0.36	1.33	0.41	0.0396	0.2235	0.0984	0.3943	0.33
hsa-miR-6503-3p	2.51	2.15	0.47	0.42	1.17	0.22	0.1088	0.3275	0.1012	0.4027	0.36
hsa-miR-6786-3p	2.04	2.26	0.46	0.35	0.90	-0.15	0.0379	0.2199	0.1023	0.4046	0.68
hsa-miR-6831-5p	1.65	1.13	0.85	0.70	1.46	0.55	0.1756	0.4228	0.1048	0.4120	0.38
hsa-miR-1976	1.85	1.95	0.24	0.21	0.95	-0.08	0.1254	0.3461	0.1086	0.4131	0.63
hsa-miR-3619-3p	1.14	1.26	0.30	0.22	0.90	-0.15	0.0560	0.2432	0.1081	0.4131	0.66
hsa-miR-4428	1.15	1.16	0.70	0.37	0.99	-0.01	0.3765	0.6605	0.1072	0.4131	0.42
hsa-miR-525-5p	1.81	1.70	0.33	0.21	1.07	0.09	0.1814	0.4316	0.1091	0.4131	0.39
hsa-miR-6775-5p	2.03	1.41	0.90	0.75	1.45	0.53	0.1566	0.4018	0.1079	0.4131	0.38
hsa-miR-6780b-3p	1.65	1.73	0.25	0.42	0.95	-0.07	0.1756	0.4228	0.1067	0.4131	0.62
hsa-miR-4499	1.55	1.36	1.36	0.71	1.14	0.19	0.2616	0.5451	0.1100	0.4139	0.40
hsa-miR-6756-5p	3.06	2.62	1.21	0.97	1.17	0.22	0.1489	0.3853	0.1142	0.4246	0.38
hsa-miR-6875-3p	1.26	1.49	0.34	0.29	0.84	-0.25	0.1030	0.3263	0.1142	0.4246	0.64
hsa-miR-611	0.90	1.08	0.25	0.61	0.84	-0.26	0.0510	0.2365	0.1156	0.4272	0.67

hsa-miR-425-3p	4.33	3.65	1.66	1.32	1.19	0.25	0.1645	0.4071	0.1211	0.4348	0.38
hsa-miR-4534	1.76	1.19	0.85	0.77	1.48	0.56	0.0577	0.2455	0.1206	0.4348	0.34
hsa-miR-6752-5p	2.35	2.00	0.74	0.71	1.17	0.23	0.0922	0.3045	0.1198	0.4348	0.36
hsa-miR-6894-3p	2.25	2.61	0.54	1.06	0.86	-0.22	0.1299	0.3552	0.1202	0.4348	0.63
hsa-miR-764	1.69	2.02	0.36	1.55	0.84	-0.26	0.0035	0.1269	0.1193	0.4348	0.75
hsa-let-7i-5p	1.75	1.60	0.49	0.51	1.09	0.13	0.0777	0.2789	0.1245	0.4379	0.35
hsa-miR-1238-5p	2.30	2.20	0.28	0.23	1.05	0.07	0.1068	0.3275	0.1252	0.4379	0.36
hsa-miR-1284	1.29	1.50	0.39	0.66	0.86	-0.22	0.0185	0.1652	0.1269	0.4379	0.70
hsa-miR-199b-5p	1.95	1.78	0.23	0.24	1.09	0.13	0.0747	0.2762	0.1246	0.4379	0.35
hsa-miR-6090	21.41	39.18	19.71	17.61	0.55	-0.87	0.0684	0.2606	0.1258	0.4379	0.65
hsa-miR-6515-5p	1.27	0.97	0.58	0.44	1.31	0.39	0.1832	0.4343	0.1259	0.4379	0.39
hsa-miR-6822-3p	1.19	1.26	0.29	0.36	0.95	-0.08	0.2123	0.4741	0.1269	0.4379	0.61
hsa-miR-1268b	2.52	2.41	1.69	1.05	1.04	0.06	0.3924	0.6731	0.1339	0.4482	0.43
hsa-miR-4655-5p	2.19	1.58	5.96	0.54	1.38	0.47	0.0169	0.1644	0.1321	0.4482	0.30
hsa-miR-513b-3p	1.48	1.45	0.28	0.18	1.02	0.03	0.2931	0.5688	0.1318	0.4482	0.41
hsa-miR-6716-3p	1.96	2.03	1.13	3.74	0.97	-0.05	0.0609	0.2471	0.1331	0.4482	0.66
hsa-miR-6796-5p	0.95	0.80	0.75	0.26	1.19	0.25	0.0609	0.2471	0.1342	0.4482	0.34
hsa-miR-7975	6.38	8.26	29.72	5.81	0.77	-0.37	0.7619	0.9296	0.1335	0.4482	0.47
hsa-miR-6728-3p	2.47	2.66	0.42	0.47	0.93	-0.11	0.1210	0.3386	0.1357	0.4507	0.63
hsa-miR-324-3p	2.78	2.54	0.77	0.51	1.09	0.13	0.0186	0.1652	0.1373	0.4511	0.30
hsa-miR-760	1.12	0.86	1.42	0.64	1.31	0.39	0.0922	0.3045	0.1373	0.4511	0.36
hsa-miR-1292-3p	1.15	1.55	0.43	0.46	0.74	-0.44	0.1107	0.3275	0.1402	0.4548	0.64
hsa-miR-19b-3p	1.91	1.91	4.25	0.70	1.00	0.00	0.9432	0.9762	0.1397	0.4548	0.51
hsa-miR-4687-5p	2.66	3.31	0.79	0.63	0.80	-0.31	0.1933	0.4496	0.1412	0.4548	0.61
hsa-miR-5010-3p	3.42	3.89	0.80	0.94	0.88	-0.19	0.1592	0.4052	0.1414	0.4548	0.62
hsa-miR-4708-5p	1.17	1.54	0.46	0.41	0.76	-0.39	0.0626	0.2523	0.1421	0.4548	0.66
hsa-miR-3154	0.97	0.78	2.94	0.46	1.24	0.31	0.0823	0.2872	0.1445	0.4598	0.35
hsa-miR-520d-3p	1.84	1.48	0.32	0.43	1.25	0.32	0.0609	0.2471	0.1451	0.4598	0.34
hsa-miR-765	1.16	0.98	0.60	0.49	1.18	0.23	0.2800	0.5611	0.1468	0.4626	0.41
hsa-miR-6804-3p	1.82	2.21	0.52	0.45	0.83	-0.28	0.2155	0.4780	0.1479	0.4639	0.61
hsa-miR-555	1.30	1.40	0.23	0.41	0.93	-0.11	0.0515	0.2367	0.1494	0.4663	0.67
hsa-miR-3667-5p	1.27	1.14	0.56	0.33	1.11	0.16	0.3576	0.6437	0.1517	0.4710	0.42

hsa-miR-3141	1.83	1.41	1.40	0.89	1.30	0.38	0.3404	0.6199	0.1542	0.4765	0.42
hsa-miR-24-3p	1.45	1.35	0.36	0.24	1.07	0.10	0.1995	0.4538	0.1587	0.4767	0.39
hsa-miR-5571-5p	3.09	3.60	0.79	0.85	0.86	-0.22	0.1756	0.4228	0.1587	0.4767	0.62
hsa-miR-5787	4.33	8.05	3.28	4.50	0.54	-0.89	0.1755	0.4228	0.1569	0.4767	0.62
hsa-miR-6769b-5p	1.70	1.37	1.05	0.76	1.24	0.31	0.2189	0.4837	0.1578	0.4767	0.40
hsa-miR-6877-5p	0.85	0.69	1.12	0.29	1.24	0.31	0.1232	0.3416	0.1597	0.4767	0.37
hsa-miR-6887-5p	1.19	1.09	0.45	0.34	1.09	0.12	0.1995	0.4538	0.1596	0.4767	0.39
hsa-miR-8089	1.68	1.08	0.65	0.73	1.56	0.64	0.0905	0.3045	0.1576	0.4767	0.36
hsa-miR-6507-3p	3.31	3.94	0.75	1.63	0.84	-0.25	0.5505	0.8063	0.1614	0.4795	0.55
hsa-miR-150-3p	1.46	1.27	0.88	0.67	1.15	0.20	0.2494	0.5323	0.1637	0.4796	0.40
hsa-miR-3189-5p	0.94	0.99	0.44	0.18	0.95	-0.07	0.7149	0.9097	0.1637	0.4796	0.47
hsa-miR-6831-3p	1.95	2.24	0.37	0.30	0.87	-0.21	0.2616	0.5451	0.1630	0.4796	0.60
hsa-miR-1236-5p	1.03	0.72	1.68	0.80	1.44	0.52	0.1321	0.3583	0.1739	0.4952	0.37
hsa-miR-1246	35.13	21.41	43.91	39.53	1.64	0.71	0.0810	0.2859	0.1737	0.4952	0.35
hsa-miR-17-5p	1.01	1.05	1.25	0.30	0.96	-0.06	0.7913	0.9360	0.1714	0.4952	0.52
hsa-miR-4695-5p	1.33	1.07	0.72	0.67	1.25	0.32	0.1355	0.3626	0.1725	0.4952	0.37
hsa-miR-574-3p	2.19	2.38	0.78	1.58	0.92	-0.12	0.0638	0.2525	0.1719	0.4952	0.66
hsa-miR-605-5p	1.68	1.98	0.38	1.42	0.85	-0.23	0.0088	0.1644	0.1721	0.4952	0.72
hsa-miR-33b-3p	6.22	5.15	10.90	6.71	1.21	0.27	0.2808	0.5611	0.1748	0.4957	0.41
hsa-miR-4485-5p	2.81	2.56	1.42	0.45	1.10	0.13	0.2770	0.5611	0.1775	0.5009	0.41
hsa-miR-3927-5p	1.98	2.06	0.25	0.73	0.96	-0.06	0.1148	0.3315	0.1786	0.5019	0.63
hsa-miR-6088	7.73	9.75	3.97	6.64	0.79	-0.34	0.1867	0.4405	0.1803	0.5044	0.61
hsa-miR-1290	1.96	1.61	4.00	2.28	1.22	0.28	0.0678	0.2598	0.1835	0.5093	0.34
hsa-miR-4451	0.98	0.79	0.48	0.42	1.24	0.31	0.1403	0.3708	0.1837	0.5093	0.37
hsa-miR-6510-5p	2.26	1.98	1.24	1.02	1.14	0.19	0.1592	0.4052	0.1867	0.5117	0.38
hsa-miR-6867-3p	2.05	2.51	0.88	0.55	0.82	-0.29	0.0210	0.1652	0.1869	0.5117	0.70
hsa-miR-7107-5p	8.40	7.44	20.66	3.08	1.13	0.17	0.3872	0.6721	0.1871	0.5117	0.43
hsa-miR-4433b-3p	0.87	0.68	0.76	0.81	1.29	0.36	0.0823	0.2872	0.1883	0.5128	0.35
hsa-miR-7977	3.67	5.10	14.22	4.65	0.72	-0.48	0.5804	0.8347	0.1901	0.5156	0.45
hsa-miR-1237-3p	3.15	3.29	0.62	0.89	0.96	-0.06	0.2222	0.4876	0.1946	0.5198	0.60
hsa-miR-4512	1.69	2.05	0.85	0.72	0.82	-0.29	0.0182	0.1652	0.1950	0.5198	0.70
hsa-miR-6779-5p	1.16	1.00	0.34	0.34	1.16	0.22	0.1148	0.3315	0.1928	0.5198	0.37

hsa-miR-6885-3p	2.72	2.89	0.36	0.40	0.94	-0.09	0.1903	0.4442	0.1940	0.5198	0.61
hsa-miR-876-3p	1.41	1.20	3.21	0.29	1.17	0.23	0.0346	0.2085	0.1965	0.5215	0.32
hsa-miR-181a-5p	0.93	0.92	0.39	0.23	1.01	0.02	0.3634	0.6467	0.2004	0.5252	0.42
hsa-miR-516a-3p	1.35	1.40	0.21	0.30	0.96	-0.06	0.4023	0.6807	0.1992	0.5252	0.57
hsa-miR-937-3p	1.71	1.73	0.37	0.32	0.99	-0.02	0.3359	0.6172	0.2004	0.5252	0.58
hsa-miR-599	1.36	1.31	0.24	0.14	1.04	0.05	0.3449	0.6245	0.2025	0.5280	0.42
hsa-miR-6892-3p	2.99	3.40	0.73	0.67	0.88	-0.19	0.3728	0.6579	0.2032	0.5280	0.58
hsa-miR-6085	3.75	4.01	2.16	1.54	0.94	-0.09	0.4174	0.6969	0.2077	0.5373	0.43
hsa-miR-451b	1.69	1.81	0.29	2.58	0.94	-0.10	0.0311	0.2010	0.2095	0.5375	0.68
hsa-miR-4698	2.83	2.69	0.71	0.38	1.05	0.07	0.4760	0.7445	0.2093	0.5375	0.44
hsa-miR-4723-3p	2.90	3.29	0.77	0.64	0.88	-0.18	0.2155	0.4780	0.2118	0.5388	0.61
hsa-miR-4756-3p	1.43	1.55	0.31	0.86	0.92	-0.12	0.2800	0.5611	0.2158	0.5388	0.59
hsa-miR-491-3p	1.80	1.91	0.26	0.24	0.94	-0.08	0.1087	0.3275	0.2157	0.5388	0.64
hsa-miR-6812-3p	3.47	3.22	0.86	0.67	1.08	0.11	0.2026	0.4574	0.2127	0.5388	0.39
hsa-miR-6846-3p	1.83	1.89	0.61	0.86	0.97	-0.04	0.0975	0.3185	0.2119	0.5388	0.64
hsa-miR-933	2.87	2.60	1.07	0.82	1.10	0.14	0.2972	0.5713	0.2160	0.5388	0.41
hsa-miR-98-3p	3.53	3.86	0.76	1.25	0.91	-0.13	0.3403	0.6199	0.2158	0.5388	0.58
hsa-miR-8072	2.18	1.70	0.82	1.00	1.29	0.36	0.0489	0.2355	0.2196	0.5454	0.33
hsa-miR-20b-5p	1.28	1.26	1.01	0.33	1.02	0.03	0.9007	0.9655	0.2214	0.5477	0.49
hsa-miR-3960	35.13	46.96	17.96	20.65	0.75	-0.42	0.1963	0.4517	0.2234	0.5504	0.61
hsa-miR-4322	1.20	1.07	0.43	0.41	1.12	0.16	0.1785	0.4263	0.2244	0.5509	0.39
hsa-miR-6862-3p	1.61	1.67	0.34	0.30	0.96	-0.06	0.1088	0.3275	0.2259	0.5524	0.64
hsa-miR-1255b-2-3p	1.90	2.02	0.29	0.29	0.94	-0.09	0.1645	0.4071	0.2281	0.5554	0.62
hsa-miR-3679-5p	2.55	2.38	5.59	1.93	1.07	0.10	0.2654	0.5493	0.2322	0.5632	0.40
hsa-miR-8485	2.43	2.54	0.53	1.14	0.96	-0.06	0.2770	0.5611	0.2334	0.5640	0.59
hsa-miR-3679-3p	2.87	2.94	0.60	0.78	0.98	-0.03	0.3634	0.6467	0.2360	0.5658	0.58
hsa-miR-4730	1.88	1.98	0.35	2.91	0.95	-0.08	0.2222	0.4876	0.2351	0.5658	0.60
hsa-miR-1539	4.63	4.08	1.32	1.18	1.13	0.18	0.2809	0.5611	0.2381	0.5687	0.41
hsa-miR-6790-5p	1.42	1.08	0.79	0.86	1.32	0.40	0.1607	0.4056	0.2415	0.5746	0.38
hsa-miR-125b-1-3p	0.94	0.95	0.21	0.36	0.99	-0.01	0.3729	0.6579	0.2439	0.5758	0.58
hsa-miR-6087	14.49	23.76	9.94	10.12	0.61	-0.71	0.2760	0.5611	0.2444	0.5758	0.59

hsa-miR-671-5p	6.48	4.84	3.76	3.04	1.34	0.42	0.3139	0.5944	0.2448	0.5758	0.41
hsa-miR-127-5p	0.78	0.76	0.18	0.34	1.03	0.05	0.7082	0.9068	0.2464	0.5765	0.53
hsa-miR-483-5p	1.53	1.13	0.92	0.81	1.36	0.44	0.3635	0.6467	0.2478	0.5765	0.42
hsa-miR-6890-3p	3.11	3.21	0.52	0.54	0.97	-0.04	0.2504	0.5324	0.2478	0.5765	0.60
hsa-miR-802	1.43	1.44	0.26	0.45	0.99	-0.02	0.3874	0.6721	0.2490	0.5771	0.57
hsa-miR-3180-5p	3.20	2.88	0.76	0.64	1.11	0.15	0.2971	0.5713	0.2511	0.5797	0.41
hsa-miR-563	3.04	3.22	0.73	0.94	0.95	-0.08	0.2395	0.5165	0.2526	0.5810	0.60
hsa-miR-122-3p	2.11	2.21	0.34	0.36	0.96	-0.07	0.2850	0.5618	0.2548	0.5818	0.59
hsa-miR-6124	3.31	2.72	3.00	1.74	1.22	0.29	0.4871	0.7524	0.2541	0.5818	0.44
hsa-miR-6821-5p	12.69	21.41	8.99	7.63	0.59	-0.75	0.2834	0.5618	0.2559	0.5821	0.59
hsa-miR-500b-5p	1.34	1.37	0.29	0.28	0.98	-0.03	0.2800	0.5611	0.2593	0.5871	0.59
hsa-miR-6779-3p	2.41	2.57	0.61	0.34	0.94	-0.09	0.1107	0.3275	0.2609	0.5871	0.64
hsa-miR-6884-3p	2.63	3.10	0.58	0.63	0.85	-0.24	0.2578	0.5446	0.2606	0.5871	0.60
hsa-miR-4433a-3p	1.92	1.18	1.50	1.54	1.62	0.70	0.1439	0.3756	0.2653	0.5947	0.38
hsa-miR-483-3p	3.25	3.55	1.55	1.21	0.92	-0.13	0.0922	0.3045	0.2676	0.5977	0.64
hsa-miR-185-3p	1.49	1.58	0.20	0.36	0.95	-0.08	0.2360	0.5125	0.2700	0.6010	0.60
hsa-miR-574-5p	2.50	3.06	0.85	1.47	0.82	-0.29	0.4023	0.6807	0.2728	0.6050	0.57
hsa-miR-124-3p	1.40	1.30	0.98	0.37	1.07	0.10	0.4175	0.6969	0.2762	0.6095	0.43
hsa-miR-4650-5p	1.73	1.79	0.32	0.36	0.97	-0.05	0.3495	0.6309	0.2768	0.6095	0.58
hsa-miR-6748-3p	1.48	1.79	0.88	0.46	0.83	-0.27	0.0174	0.1649	0.2781	0.6102	0.70
hsa-miR-129-2-3p	3.09	3.24	0.66	0.90	0.95	-0.07	0.5039	0.7720	0.2809	0.6125	0.56
hsa-miR-21-5p	1.86	1.54	1.95	1.16	1.21	0.28	0.4579	0.7337	0.2813	0.6125	0.44
hsa-miR-2392	1.60	1.18	0.89	1.06	1.36	0.44	0.1107	0.3275	0.2840	0.6125	0.36
hsa-miR-6722-3p	0.92	0.76	0.59	0.63	1.21	0.27	0.1049	0.3275	0.2840	0.6125	0.36
hsa-miR-93-5p	1.07	0.99	0.50	0.30	1.09	0.12	0.3765	0.6605	0.2829	0.6125	0.42
hsa-miR-365a-3p	3.77	3.64	0.80	0.81	1.04	0.05	0.3314	0.6108	0.2864	0.6154	0.42
hsa-miR-6866-3p	2.05	2.18	0.61	0.54	0.94	-0.09	0.1232	0.3416	0.2922	0.6256	0.63
hsa-miR-663a	1.16	1.56	0.44	0.76	0.75	-0.42	0.5446	0.8014	0.2955	0.6307	0.55
hsa-miR-15b-5p	1.77	1.88	0.91	0.24	0.94	-0.09	0.6241	0.8651	0.2974	0.6326	0.54
hsa-miR-3613-3p	3.25	3.56	0.71	0.73	0.91	-0.13	0.3013	0.5758	0.3056	0.6478	0.59
hsa-miR-613	1.55	1.54	0.30	0.98	1.01	0.01	0.9929	0.9945	0.3067	0.6478	0.50
hsa-miR-181c-3p	1.11	1.09	0.27	0.10	1.02	0.03	0.5505	0.8063	0.3095	0.6515	0.45

hsa-miR-1227-3p	2.37	2.12	0.44	0.58	1.12	0.16	0.0733	0.2743	0.3148	0.6605	0.35
hsa-miR-548d-3p	1.60	1.52	0.19	0.24	1.05	0.07	0.1263	0.3469	0.3164	0.6616	0.37
hsa-miR-26a-5p	1.06	1.00	0.39	0.27	1.06	0.08	0.5097	0.7720	0.3175	0.6617	0.44
hsa-miR-6734-5p	1.08	0.76	0.64	0.89	1.43	0.51	0.0472	0.2327	0.3202	0.6628	0.33
hsa-miR-6834-3p	2.91	2.99	0.74	0.63	0.97	-0.04	0.2616	0.5451	0.3198	0.6628	0.60
hsa-miR-6819-3p	6.01	5.77	4.58	2.93	1.04	0.06	0.7824	0.9353	0.3219	0.6642	0.48
hsa-miR-1-5p	2.08	2.07	0.32	0.40	1.00	0.00	0.6053	0.8582	0.3261	0.6669	0.54
hsa-miR-105-5p	1.28	1.35	0.24	0.21	0.94	-0.08	0.2931	0.5688	0.3294	0.6669	0.59
hsa-miR-1268a	2.90	3.07	1.48	1.00	0.94	-0.08	0.8237	0.9438	0.3286	0.6669	0.48
hsa-miR-4327	1.63	1.39	0.79	0.68	1.17	0.23	0.3315	0.6108	0.3308	0.6669	0.42
hsa-miR-490-5p	0.99	1.05	0.22	0.50	0.94	-0.09	0.5625	0.8200	0.3302	0.6669	0.55
hsa-miR-6728-5p	1.65	1.47	0.63	0.60	1.12	0.17	0.4705	0.7415	0.3295	0.6669	0.44
hsa-miR-8069	26.13	35.13	52.28	59.82	0.74	-0.43	0.4586	0.7337	0.3273	0.6669	0.56
hsa-miR-18a-5p	1.06	1.10	0.39	0.17	0.96	-0.05	0.7149	0.9097	0.3339	0.6688	0.53
hsa-miR-3188	1.31	1.06	0.70	0.89	1.23	0.30	0.0807	0.2859	0.3328	0.6688	0.35
hsa-miR-634	2.51	2.75	0.49	0.95	0.91	-0.13	0.7282	0.9110	0.3358	0.6706	0.53
hsa-miR-4307	1.84	1.68	0.41	1.40	1.10	0.13	0.8739	0.9605	0.3408	0.6739	0.49
hsa-miR-6730-3p	2.43	2.48	0.43	0.32	0.98	-0.03	0.3874	0.6721	0.3398	0.6739	0.57
hsa-miR-6794-5p	1.57	1.38	0.72	0.62	1.13	0.18	0.4435	0.7248	0.3394	0.6739	0.43
hsa-miR-3195	1.97	1.70	1.33	32.25	1.16	0.21	0.9578	0.9799	0.3424	0.6751	0.49
hsa-miR-1299	1.78	1.27	3.73	1.66	1.40	0.49	0.1892	0.4434	0.3458	0.6795	0.39
hsa-miR-1238-3p	6.01	5.59	2.64	2.22	1.08	0.11	0.5096	0.7720	0.3477	0.6811	0.44
hsa-miR-494-3p	2.04	1.76	15.27	16.15	1.16	0.21	0.9289	0.9703	0.3494	0.6822	0.51
hsa-miR-4728-3p	3.18	3.51	0.64	0.75	0.91	-0.14	0.2654	0.5493	0.3568	0.6860	0.60
hsa-miR-4745-5p	1.49	1.87	0.58	0.61	0.80	-0.32	0.4596	0.7337	0.3540	0.6860	0.56
hsa-miR-5195-3p	1.93	1.60	0.58	0.44	1.21	0.27	0.4760	0.7445	0.3549	0.6860	0.44
hsa-miR-6737-3p	8.72	8.99	4.31	9.13	0.97	-0.04	0.8237	0.9438	0.3558	0.6860	0.52
hsa-miR-6765-3p	2.77	3.11	0.69	0.52	0.89	-0.17	0.1415	0.3708	0.3549	0.6860	0.63
hsa-miR-6749-5p	8.05	8.72	3.26	4.00	0.92	-0.12	0.4756	0.7445	0.3591	0.6883	0.56
hsa-miR-374a-3p	1.55	1.36	0.29	0.33	1.14	0.18	0.3449	0.6245	0.3609	0.6897	0.42
hsa-miR-1185-2-3p	1.19	1.21	0.41	0.25	0.99	-0.02	0.6560	0.8875	0.3700	0.6927	0.46
hsa-miR-188-5p	2.38	1.94	1.19	0.81	1.23	0.30	0.6689	0.8952	0.3703	0.6927	0.46

hsa-miR-335-3p	2.19	2.18	1.19	2.40	1.01	0.01	0.6689	0.8952	0.3715	0.6927	0.54
hsa-miR-3940-5p	1.81	1.56	1.59	1.39	1.15	0.21	0.4579	0.7337	0.3688	0.6927	0.44
hsa-miR-4443	2.60	1.87	1.00	16.69	1.39	0.48	0.2850	0.5618	0.3709	0.6927	0.41
hsa-miR-4497	1.22	1.76	9.52	9.72	0.69	-0.53	0.9148	0.9703	0.3687	0.6927	0.51
hsa-miR-4700-5p	0.89	0.83	0.45	0.25	1.08	0.11	0.7082	0.9068	0.3660	0.6927	0.47
hsa-miR-6743-3p	3.11	3.47	0.86	0.63	0.90	-0.16	0.1617	0.4066	0.3681	0.6927	0.62
hsa-miR-6796-3p	2.82	2.87	0.56	0.47	0.98	-0.02	0.4174	0.6969	0.3730	0.6936	0.57
hsa-miR-4297	1.33	1.51	1.11	0.56	0.88	-0.18	0.0025	0.1269	0.3778	0.7003	0.76
hsa-miR-718	1.26	1.48	0.61	0.75	0.85	-0.23	0.4871	0.7524	0.3799	0.7020	0.56
hsa-miR-6754-3p	1.24	1.57	0.95	0.48	0.79	-0.34	0.0265	0.1892	0.3818	0.7035	0.69
hsa-miR-149-5p	4.63	5.00	1.33	1.49	0.93	-0.11	0.5684	0.8229	0.3869	0.7053	0.55
hsa-miR-602	3.15	3.46	0.84	0.69	0.91	-0.13	0.3972	0.6759	0.3873	0.7053	0.57
hsa-miR-6869-5p	5.71	9.98	6.74	6.99	0.57	-0.81	0.2888	0.5641	0.3865	0.7053	0.59
hsa-miR-939-5p	2.10	1.60	0.51	0.58	1.31	0.39	0.3227	0.6035	0.3854	0.7053	0.42
hsa-miR-4707-5p	1.32	1.05	0.84	0.96	1.26	0.33	0.1645	0.4071	0.3897	0.7075	0.38
hsa-miR-6800-3p	6.22	5.77	2.76	2.14	1.08	0.11	0.5211	0.7836	0.3913	0.7084	0.44
hsa-miR-6855-3p	3.07	3.67	0.76	0.98	0.84	-0.26	0.6367	0.8729	0.4008	0.7235	0.54
hsa-miR-4728-5p	1.70	1.36	0.54	0.57	1.25	0.32	0.2494	0.5323	0.4050	0.7291	0.40
hsa-miR-223-3p	3.85	3.62	1.12	0.93	1.06	0.09	0.4595	0.7337	0.4069	0.7303	0.44
hsa-miR-548d-5p	1.88	1.67	0.36	0.43	1.13	0.18	0.2058	0.4630	0.4083	0.7304	0.39
hsa-miR-576-5p	2.24	2.12	0.33	0.46	1.06	0.08	0.7824	0.9353	0.4093	0.7304	0.52
hsa-miR-520g-3p	1.75	1.75	0.32	0.51	1.00	0.00	0.8600	0.9554	0.4162	0.7385	0.52
hsa-miR-7114-5p	1.18	1.07	0.51	0.57	1.10	0.14	0.1344	0.3614	0.4158	0.7385	0.37
hsa-miR-4739	1.12	1.20	0.53	0.46	0.93	-0.10	0.2616	0.5451	0.4219	0.7420	0.60
hsa-miR-505-3p	2.08	2.16	0.25	0.28	0.97	-0.05	0.1873	0.4405	0.4221	0.7420	0.61
hsa-miR-6790-3p	2.57	2.54	0.39	0.33	1.01	0.01	0.3874	0.6721	0.4197	0.7420	0.43
hsa-miR-6889-3p	5.77	5.65	1.63	1.84	1.02	0.03	0.5684	0.8229	0.4230	0.7420	0.55
hsa-miR-4713-5p	1.49	1.89	1.02	0.31	0.79	-0.34	0.0108	0.1644	0.4244	0.7425	0.72
hsa-miR-1913	1.11	1.32	0.48	0.33	0.84	-0.26	0.2881	0.5641	0.4268	0.7435	0.59
hsa-miR-4665-3p	8.05	7.44	5.24	3.83	1.08	0.11	0.6239	0.8651	0.4275	0.7435	0.46
hsa-miR-186-3p	1.63	1.64	0.22	0.18	0.99	-0.01	0.8600	0.9554	0.4323	0.7438	0.48
hsa-miR-7106-5p	1.40	1.13	0.60	0.95	1.24	0.31	0.0259	0.1871	0.4324	0.7438	0.31

hsa-miR-7641	2.81	2.95	17.76	18.91	0.95	-0.07	0.9218	0.9703	0.4298	0.7438	0.49
hsa-miR-7845-5p	1.85	1.15	0.87	1.51	1.61	0.68	0.0479	0.2344	0.4321	0.7438	0.33
hsa-miR-1470	2.99	2.75	0.64	0.48	1.09	0.12	0.3314	0.6108	0.4373	0.7499	0.42
hsa-miR-4286	2.49	2.37	0.97	0.70	1.05	0.07	0.6495	0.8826	0.4383	0.7499	0.46
hsa-miR-154-5p	1.86	1.72	0.62	0.40	1.08	0.11	0.3013	0.5758	0.4429	0.7510	0.41
hsa-miR-197-3p	3.29	4.13	2.18	0.92	0.80	-0.33	0.0196	0.1652	0.4475	0.7510	0.70
hsa-miR-4530	3.35	5.20	4.36	4.45	0.64	-0.63	0.6302	0.8661	0.4430	0.7510	0.54
hsa-miR-4640-3p	2.65	2.70	0.56	0.53	0.98	-0.03	0.4870	0.7524	0.4475	0.7510	0.56
hsa-miR-521	1.90	1.98	0.35	0.33	0.96	-0.06	0.2290	0.4990	0.4456	0.7510	0.60
hsa-miR-6069	6.48	6.87	3.26	2.12	0.94	-0.08	0.8237	0.9438	0.4455	0.7510	0.48
hsa-miR-6774-5p	1.12	1.11	0.38	0.24	1.01	0.02	0.7109	0.9084	0.4421	0.7510	0.47
hsa-miR-26b-3p	1.85	1.92	0.28	0.34	0.97	-0.05	0.4705	0.7415	0.4496	0.7526	0.56
hsa-miR-7108-5p	2.48	2.32	1.73	1.66	1.07	0.09	0.3682	0.6532	0.4527	0.7558	0.42
hsa-miR-129-1-3p	3.07	3.12	0.64	0.75	0.98	-0.02	0.6304	0.8661	0.4581	0.7618	0.54
hsa-miR-6736-3p	1.68	1.90	0.90	0.47	0.88	-0.18	0.1321	0.3583	0.4588	0.7618	0.63
hsa-miR-640	1.53	1.86	0.59	0.35	0.82	-0.29	0.1502	0.3870	0.4615	0.7642	0.62
hsa-miR-615-3p	1.52	1.58	0.75	0.86	0.96	-0.06	0.2810	0.5611	0.4655	0.7689	0.59
hsa-miR-4689	1.13	1.13	0.76	0.46	1.00	0.00	0.8866	0.9605	0.4688	0.7722	0.49
hsa-miR-4511	1.31	1.38	0.48	0.48	0.95	-0.08	0.5328	0.7916	0.4713	0.7743	0.55
hsa-miR-2116-3p	3.89	3.49	1.44	1.38	1.12	0.16	0.5212	0.7836	0.4727	0.7745	0.44
hsa-miR-4261	1.06	1.20	0.34	0.21	0.89	-0.17	0.1391	0.3708	0.4765	0.7766	0.63
hsa-miR-6127	19.22	17.08	15.36	14.45	1.13	0.17	0.2707	0.5585	0.4761	0.7766	0.41
hsa-miR-1207-5p	4.06	3.23	2.11	1.96	1.26	0.33	0.3922	0.6731	0.4780	0.7771	0.43
hsa-miR-4701-5p	3.72	3.35	1.14	0.98	1.11	0.15	0.4814	0.7494	0.4844	0.7853	0.44
hsa-let-7f-1-3p	4.36	4.91	1.49	1.59	0.89	-0.17	0.5268	0.7902	0.4872	0.7878	0.55
hsa-miR-10a-5p	1.98	1.89	0.34	0.28	1.05	0.07	0.6431	0.8759	0.4899	0.7903	0.54
hsa-miR-1225-5p	6.66	10.43	6.07	4.90	0.64	-0.65	0.3095	0.5882	0.4941	0.7913	0.59
hsa-miR-6766-3p	3.06	3.65	1.01	0.86	0.84	-0.26	0.4815	0.7494	0.4936	0.7913	0.56
hsa-miR-762	2.38	3.00	2.82	2.77	0.79	-0.34	0.6241	0.8651	0.4944	0.7913	0.54
hsa-miR-197-5p	11.65	12.01	10.84	12.42	0.97	-0.04	0.8936	0.9618	0.4991	0.7948	0.51
hsa-miR-6777-5p	1.24	1.13	0.71	3.64	1.09	0.13	0.5040	0.7720	0.4988	0.7948	0.44
hsa-miR-4666b	3.07	3.04	0.73	0.75	1.01	0.01	0.6115	0.8611	0.5011	0.7959	0.54

hsa-miR-337-3p	2.50	2.64	1.85	1.68	0.95	-0.08	0.0363	0.2144	0.5033	0.7974	0.68
hsa-miR-6880-3p	4.18	4.49	1.14	1.10	0.93	-0.10	0.4488	0.7297	0.5052	0.7983	0.56
hsa-miR-6832-3p	1.94	1.99	0.33	0.30	0.98	-0.03	0.4175	0.6969	0.5093	0.8028	0.57
hsa-miR-6848-3p	3.49	3.80	0.94	1.09	0.92	-0.12	0.6304	0.8661	0.5114	0.8040	0.54
hsa-miR-4505	4.44	5.65	3.77	3.75	0.79	-0.35	0.3972	0.6759	0.5154	0.8082	0.57
hsa-miR-6068	2.41	2.73	1.96	2.18	0.88	-0.18	0.6115	0.8611	0.5192	0.8121	0.54
hsa-miR-6841-3p	2.55	2.52	0.44	0.60	1.01	0.02	0.9149	0.9703	0.5228	0.8157	0.51
hsa-miR-1267	2.82	2.70	0.69	0.66	1.05	0.07	0.6177	0.8621	0.5277	0.8173	0.46
hsa-miR-4540	1.43	1.43	0.25	0.23	1.00	-0.01	0.7282	0.9110	0.5271	0.8173	0.47
hsa-miR-6727-5p	2.10	2.27	1.15	1.33	0.92	-0.11	0.6431	0.8759	0.5266	0.8173	0.54
hsa-miR-3162-5p	11.65	10.43	8.15	6.55	1.12	0.16	0.6175	0.8621	0.5334	0.8190	0.46
hsa-miR-4740-3p	1.50	1.48	0.29	0.27	1.01	0.02	0.7892	0.9353	0.5314	0.8190	0.52
hsa-miR-4787-3p	4.96	4.67	1.77	1.60	1.06	0.09	0.5445	0.8014	0.5323	0.8190	0.45
hsa-miR-638	4.30	6.01	4.32	4.56	0.71	-0.48	0.4330	0.7170	0.5349	0.8190	0.57
hsa-miR-645	1.24	1.26	0.17	0.25	0.99	-0.01	0.8878	0.9605	0.5361	0.8190	0.51
hsa-miR-6731-3p	3.89	3.89	1.19	0.93	1.00	0.00	0.6950	0.9021	0.5381	0.8190	0.47
hsa-miR-6886-3p	1.48	1.94	2.85	1.93	0.76	-0.40	0.0079	0.1644	0.5374	0.8190	0.73
hsa-miR-4284	6.22	5.85	4.56	6.18	1.06	0.09	0.4433	0.7248	0.5404	0.8204	0.43
hsa-miR-2861	5.65	9.75	7.62	7.17	0.58	-0.79	0.4329	0.7170	0.5477	0.8215	0.57
hsa-miR-4769-3p	3.29	3.36	0.88	0.67	0.98	-0.03	0.7016	0.9021	0.5474	0.8215	0.47
hsa-miR-6724-5p	4.40	4.67	1.73	1.81	0.94	-0.08	0.4542	0.7337	0.5438	0.8215	0.44
hsa-miR-6756-3p	2.98	2.99	0.56	0.74	1.00	0.00	0.7241	0.9110	0.5470	0.8215	0.53
hsa-miR-7110-5p	3.69	3.31	2.33	2.29	1.11	0.15	0.5685	0.8229	0.5431	0.8215	0.45
hsa-miR-548i	1.18	1.12	0.20	0.28	1.05	0.07	0.2578	0.5446	0.5497	0.8226	0.40
hsa-miR-3928-5p	1.06	1.12	1.18	1.13	0.95	-0.08	0.0922	0.3045	0.5566	0.8269	0.64
hsa-miR-4793-5p	1.69	1.49	0.34	0.50	1.13	0.17	0.5841	0.8357	0.5563	0.8269	0.45
hsa-miR-581	1.31	1.36	0.22	0.27	0.96	-0.05	0.7484	0.9185	0.5546	0.8269	0.53
hsa-miR-4721	2.19	2.29	1.08	1.31	0.96	-0.07	0.8656	0.9564	0.5584	0.8276	0.52
hsa-miR-4310	4.30	4.30	1.34	1.20	1.00	0.00	0.5867	0.8357	0.5626	0.8299	0.45
hsa-miR-6776-3p	2.60	2.76	0.44	0.40	0.94	-0.09	0.4382	0.7218	0.5613	0.8299	0.57
hsa-miR-7152-5p	1.97	2.30	0.83	0.45	0.86	-0.22	0.1728	0.4228	0.5643	0.8304	0.62
hsa-miR-195-3p	1.15	1.25	0.66	0.25	0.92	-0.13	0.0346	0.2085	0.5704	0.8375	0.68

hsa-miR-6878-3p	2.02	2.19	0.96	0.32	0.92	-0.12	0.0136	0.1644	0.5719	0.8376	0.71
hsa-miR-181b-5p	1.19	0.92	0.51	0.57	1.29	0.37	0.4060	0.6851	0.5759	0.8415	0.43
hsa-miR-487a-5p	1.18	1.22	0.43	0.25	0.97	-0.04	0.2396	0.5165	0.5799	0.8453	0.60
hsa-miR-6759-3p	4.24	4.13	1.59	1.13	1.03	0.04	0.8585	0.9554	0.5815	0.8456	0.48
hsa-miR-6865-3p	5.35	5.52	2.10	1.87	0.97	-0.05	0.5328	0.7916	0.5851	0.8485	0.55
hsa-miR-6891-5p	2.72	2.80	1.67	1.57	0.97	-0.05	0.7349	0.9110	0.5861	0.8485	0.47
hsa-miR-134-5p	1.22	1.13	0.51	0.45	1.08	0.11	0.5328	0.7916	0.5893	0.8491	0.45
hsa-miR-148b-3p	1.40	1.43	0.22	0.29	0.98	-0.03	0.6053	0.8582	0.5890	0.8491	0.54
hsa-miR-1227-5p	1.98	1.97	1.15	1.24	1.00	0.01	0.4435	0.7248	0.6197	0.8503	0.43
hsa-miR-1228-3p	8.26	8.26	3.23	3.65	1.00	0.00	0.8585	0.9554	0.6131	0.8503	0.52
hsa-miR-1260a	2.38	2.85	5.28	1.23	0.83	-0.26	0.1415	0.3708	0.6189	0.8503	0.63
hsa-miR-1281	6.87	7.17	3.06	2.99	0.96	-0.06	0.5039	0.7720	0.5998	0.8503	0.56
hsa-miR-15a-5p	1.92	1.81	0.59	0.40	1.06	0.08	0.7484	0.9185	0.6195	0.8503	0.47
hsa-miR-1908-3p	3.34	3.21	1.07	0.90	1.04	0.06	0.7687	0.9306	0.6097	0.8503	0.47
hsa-miR-1909-5p	1.14	1.32	1.29	0.57	0.87	-0.21	0.0108	0.1644	0.6079	0.8503	0.72
hsa-miR-211-5p	2.15	2.59	1.43	0.41	0.83	-0.26	0.0129	0.1644	0.6013	0.8503	0.71
hsa-miR-29c-5p	2.05	2.12	0.32	0.63	0.97	-0.05	0.7892	0.9353	0.5969	0.8503	0.52
hsa-miR-3605-3p	1.54	1.61	1.05	0.36	0.95	-0.07	0.5625	0.8200	0.6203	0.8503	0.55
hsa-miR-4323	2.84	2.73	0.41	0.30	1.04	0.06	0.6624	0.8942	0.6039	0.8503	0.46
hsa-miR-4787-5p	4.49	3.70	1.75	1.55	1.21	0.28	0.7282	0.9110	0.5942	0.8503	0.47
hsa-miR-4788	2.03	2.22	2.10	2.06	0.91	-0.13	0.7687	0.9306	0.5984	0.8503	0.53
hsa-miR-5100	3.75	4.24	9.36	13.49	0.89	-0.17	0.3226	0.6035	0.6159	0.8503	0.58
hsa-miR-520a-3p	1.90	1.75	0.33	0.69	1.09	0.12	0.8796	0.9605	0.6113	0.8503	0.49
hsa-miR-5703	1.80	2.53	3.39	2.54	0.71	-0.49	0.9858	0.9945	0.6005	0.8503	0.50
hsa-miR-625-3p	2.49	2.54	0.46	0.37	0.98	-0.03	0.7016	0.9021	0.5955	0.8503	0.53
hsa-miR-6763-3p	4.36	4.20	0.99	0.99	1.04	0.05	0.7349	0.9110	0.6186	0.8503	0.53
hsa-miR-6795-3p	3.00	2.99	0.93	1.23	1.01	0.01	0.8237	0.9438	0.6172	0.8503	0.52
hsa-miR-7111-3p	2.92	3.01	0.76	0.95	0.97	-0.05	0.8656	0.9564	0.5987	0.8503	0.52
hsa-miR-7847-3p	5.94	5.52	23.14	15.23	1.08	0.11	0.4382	0.7218	0.6047	0.8503	0.43
hsa-miR-885-5p	1.98	2.49	21.27	4.63	0.80	-0.33	0.0014	0.1269	0.6092	0.8503	0.76
hsa-miR-1910-5p	1.24	1.40	1.61	0.91	0.89	-0.16	0.0510	0.2365	0.6243	0.8536	0.67
hsa-miR-4749-3p	4.56	4.67	1.23	1.24	0.98	-0.03	0.7349	0.9110	0.6254	0.8536	0.53

hsa-miR-6792-3p	3.05	3.00	0.62	0.59	1.02	0.02	0.8099	0.9436	0.6365	0.8631	0.48
hsa-miR-7704	2.93	3.94	3.24	3.61	0.74	-0.43	0.7484	0.9185	0.6359	0.8631	0.53
hsa-miR-92a-3p	1.09	1.21	0.75	0.33	0.90	-0.15	0.3960	0.6759	0.6355	0.8631	0.57
hsa-miR-4725-5p	5.52	5.00	1.88	1.58	1.10	0.14	0.7015	0.9021	0.6429	0.8698	0.47
hsa-miR-1538	1.41	1.48	0.58	0.28	0.95	-0.07	0.1344	0.3614	0.6465	0.8701	0.63
hsa-miR-6763-5p	3.62	3.27	1.41	1.29	1.11	0.15	0.7016	0.9021	0.6473	0.8701	0.47
hsa-miR-6797-3p	6.48	6.94	2.22	2.58	0.93	-0.10	0.6818	0.8972	0.6453	0.8701	0.54
hsa-miR-6851-3p	4.71	4.27	1.40	1.44	1.10	0.14	0.6178	0.8621	0.6499	0.8717	0.46
hsa-miR-1914-3p	1.82	1.78	0.50	0.62	1.02	0.03	0.6819	0.8972	0.6517	0.8722	0.46
hsa-miR-181d-5p	1.52	1.30	0.78	0.73	1.17	0.23	0.3635	0.6467	0.6643	0.8740	0.42
hsa-miR-1825	5.15	4.87	1.77	1.65	1.06	0.08	0.8168	0.9438	0.6570	0.8740	0.48
hsa-miR-3675-3p	2.75	3.15	0.80	0.80	0.88	-0.19	0.8307	0.9482	0.6637	0.8740	0.52
hsa-miR-449b-3p	1.73	2.13	4.37	0.58	0.81	-0.30	0.0311	0.2010	0.6626	0.8740	0.68
hsa-miR-6165	8.40	8.40	11.62	13.22	1.00	0.00	0.9290	0.9703	0.6642	0.8740	0.51
hsa-miR-6745	0.99	0.82	0.27	0.43	1.21	0.28	0.1415	0.3708	0.6625	0.8740	0.37
hsa-miR-6757-3p	3.09	3.49	0.84	0.89	0.89	-0.17	0.6884	0.8982	0.6635	0.8740	0.53
hsa-miR-6891-3p	2.62	2.59	0.43	0.37	1.01	0.02	0.6884	0.8982	0.6600	0.8740	0.53
hsa-miR-4299	1.22	1.22	0.94	0.88	1.00	0.00	0.3227	0.6035	0.6673	0.8746	0.42
hsa-miR-4466	3.35	4.63	3.12	3.13	0.72	-0.47	0.5867	0.8357	0.6690	0.8746	0.55
hsa-miR-4687-3p	3.91	4.36	3.45	3.99	0.90	-0.16	0.7619	0.9296	0.6677	0.8746	0.53
hsa-miR-299-5p	2.41	2.56	1.07	0.34	0.94	-0.09	0.3923	0.6731	0.6714	0.8759	0.57
hsa-miR-5708	1.30	1.34	1.41	1.33	0.97	-0.05	0.3304	0.6108	0.6774	0.8819	0.58
hsa-miR-23a-3p	1.72	1.75	0.39	0.25	0.98	-0.03	0.5154	0.7788	0.6824	0.8821	0.56
hsa-miR-4313	9.19	9.47	11.23	15.98	0.97	-0.04	0.7016	0.9021	0.6838	0.8821	0.53
hsa-miR-4664-3p	3.34	3.47	0.98	1.28	0.96	-0.06	0.9716	0.9859	0.6810	0.8821	0.50
hsa-miR-572	1.46	1.57	0.87	0.86	0.93	-0.10	0.6689	0.8952	0.6846	0.8821	0.54
hsa-miR-6132	1.00	1.74	1.50	1.26	0.57	-0.80	0.5387	0.7984	0.6806	0.8821	0.55
hsa-miR-4463	1.04	0.98	0.72	0.74	1.06	0.09	0.8099	0.9436	0.6940	0.8849	0.48
hsa-miR-4516	11.17	14.49	10.87	9.24	0.77	-0.37	0.3915	0.6731	0.6929	0.8849	0.57
hsa-miR-5584-3p	1.89	1.87	1.08	0.37	1.01	0.01	0.8446	0.9536	0.6927	0.8849	0.52
hsa-miR-595	1.62	1.47	2.09	1.08	1.10	0.14	0.4488	0.7297	0.6939	0.8849	0.44
hsa-miR-651-5p	1.18	1.06	0.22	0.29	1.12	0.16	0.2962	0.5713	0.6911	0.8849	0.41

hsa-miR-3617-3p	2.18	2.52	9.22	2.46	0.86	-0.21	0.0290	0.1960	0.7011	0.8877	0.69
hsa-miR-5739	4.20	3.62	1.28	1.60	1.16	0.22	0.9290	0.9703	0.7019	0.8877	0.51
hsa-miR-6511b-3p	1.83	2.00	1.40	0.29	0.92	-0.12	0.3183	0.6008	0.7000	0.8877	0.59
hsa-miR-6752-3p	4.79	4.49	1.27	1.09	1.07	0.10	0.6177	0.8621	0.6984	0.8877	0.46
hsa-miR-7150	4.13	6.10	2.61	2.81	0.68	-0.56	0.7824	0.9353	0.7061	0.8912	0.52
hsa-miR-4646-3p	2.91	2.87	0.68	0.62	1.02	0.02	0.6304	0.8661	0.7181	0.8973	0.54
hsa-miR-4732-3p	1.65	1.82	2.20	0.41	0.91	-0.14	0.0284	0.1960	0.7200	0.8973	0.69
hsa-miR-6760-3p	3.51	3.46	1.09	1.21	1.02	0.02	0.8168	0.9438	0.7210	0.8973	0.52
hsa-miR-6784-3p	2.75	2.90	0.49	0.48	0.95	-0.07	0.7349	0.9110	0.7207	0.8973	0.53
hsa-miR-6861-3p	3.23	3.03	0.58	0.76	1.06	0.09	0.3403	0.6199	0.7183	0.8973	0.42
hsa-miR-7108-3p	2.93	3.07	0.66	0.58	0.95	-0.07	0.7961	0.9381	0.7127	0.8973	0.52
hsa-miR-99b-3p	0.88	0.80	0.22	0.39	1.10	0.14	0.5097	0.7720	0.7204	0.8973	0.44
hsa-miR-142-5p	1.11	1.14	0.42	0.20	0.97	-0.04	0.4542	0.7337	0.7241	0.8993	0.56
hsa-let-7d-3p	1.91	2.12	11.04	3.18	0.90	-0.15	0.0047	0.1337	0.7294	0.9008	0.74
hsa-miR-1587	2.29	2.08	1.19	1.71	1.10	0.14	0.8796	0.9605	0.7296	0.9008	0.49
hsa-miR-487b-5p	1.32	1.62	3.98	0.59	0.82	-0.29	0.0006	0.1269	0.7282	0.9008	0.78
hsa-miR-4645-3p	1.43	1.20	0.43	0.92	1.19	0.25	0.4278	0.7122	0.7324	0.9024	0.43
hsa-miR-1229-3p	2.47	2.45	0.55	0.48	1.01	0.01	0.6115	0.8611	0.7408	0.9025	0.46
hsa-miR-1249-3p	3.36	4.33	2.81	2.20	0.78	-0.37	0.4705	0.7415	0.7422	0.9025	0.56
hsa-miR-1275	2.30	2.87	2.32	2.39	0.80	-0.32	0.9078	0.9703	0.7387	0.9025	0.49
hsa-miR-1304-3p	6.48	6.48	2.30	2.22	1.00	0.00	0.7891	0.9353	0.7404	0.9025	0.52
hsa-miR-328-5p	1.05	1.11	1.01	0.89	0.94	-0.08	0.7824	0.9353	0.7372	0.9025	0.52
hsa-miR-3614-5p	3.02	3.02	0.72	0.74	1.00	0.00	0.7416	0.9156	0.7354	0.9025	0.53
hsa-miR-4281	17.96	19.22	11.61	9.42	0.93	-0.10	0.6806	0.8972	0.7464	0.9025	0.54
hsa-miR-449c-5p	1.03	1.03	0.18	0.29	1.00	0.00	0.7551	0.9249	0.7485	0.9025	0.47
hsa-miR-6800-5p	12.01	11.65	10.59	10.67	1.03	0.04	0.7415	0.9156	0.7483	0.9025	0.53
hsa-miR-6824-3p	4.06	4.04	1.04	1.15	1.00	0.01	0.6559	0.8875	0.7470	0.9025	0.46
hsa-miR-6879-5p	15.27	9.19	18.85	30.78	1.66	0.73	0.1486	0.3853	0.7427	0.9025	0.38
hsa-miR-636	2.61	2.67	0.50	0.48	0.98	-0.04	0.9574	0.9799	0.7630	0.9175	0.51
hsa-miR-6850-5p	3.47	3.27	1.72	1.82	1.06	0.09	0.5867	0.8357	0.7638	0.9175	0.45
hsa-miR-204-5p	2.24	2.42	3.47	0.63	0.93	-0.11	0.0404	0.2242	0.7701	0.9214	0.67
hsa-miR-544a	1.74	1.70	0.27	0.27	1.02	0.04	0.8586	0.9554	0.7692	0.9214	0.52

hsa-miR-6777-3p	3.12	2.84	0.86	1.14	1.10	0.14	0.1963	0.4517	0.7794	0.9308	0.39
hsa-miR-3656	5.15	5.71	3.73	3.22	0.90	-0.15	0.5745	0.8297	0.7831	0.9316	0.55
hsa-miR-486-5p	1.62	1.58	0.28	0.28	1.02	0.03	0.8739	0.9605	0.7826	0.9316	0.51
hsa-miR-1229-5p	4.44	4.96	2.73	2.33	0.90	-0.16	0.9361	0.9721	0.7899	0.9379	0.49
hsa-miR-130b-5p	1.67	1.89	0.69	0.23	0.88	-0.18	0.0560	0.2432	0.8011	0.9453	0.66
hsa-miR-4484	3.34	3.22	0.71	0.59	1.04	0.05	0.7824	0.9353	0.8029	0.9453	0.48
hsa-miR-4515	5.52	5.40	3.03	3.74	1.02	0.03	0.9929	0.9945	0.8023	0.9453	0.50
hsa-miR-6125	26.13	17.96	21.15	24.04	1.45	0.54	0.8934	0.9618	0.7986	0.9453	0.51
hsa-miR-6126	1.50	1.54	0.62	0.81	0.97	-0.04	0.9859	0.9945	0.8066	0.9453	0.50
hsa-miR-630	2.21	3.22	4.39	3.39	0.69	-0.54	0.6754	0.8962	0.8068	0.9453	0.54
hsa-miR-6508-5p	6.22	5.65	2.78	2.42	1.10	0.14	0.8937	0.9618	0.8043	0.9453	0.49
hsa-miR-148a-3p	1.57	1.60	0.30	0.22	0.98	-0.03	0.9297	0.9703	0.8152	0.9513	0.49
hsa-miR-4649-3p	9.19	7.73	4.72	4.79	1.19	0.25	0.7755	0.9353	0.8165	0.9513	0.48
hsa-miR-6075	0.83	0.80	0.52	0.52	1.03	0.05	0.6719	0.8962	0.8164	0.9513	0.46
hsa-miR-548ai	2.97	3.15	0.64	0.87	0.94	-0.09	0.5806	0.8347	0.8221	0.9543	0.45
hsa-miR-6858-3p	5.59	4.91	2.03	2.67	1.14	0.19	0.7892	0.9353	0.8222	0.9543	0.48
hsa-miR-4459	39.18	39.18	25.33	21.20	1.00	0.00	0.7875	0.9353	0.8333	0.9655	0.52
hsa-miR-3622b-3p	1.09	1.36	1.93	0.65	0.80	-0.32	0.0047	0.1337	0.8443	0.9674	0.74
hsa-miR-422a	0.66	0.59	0.16	0.38	1.13	0.17	0.2731	0.5611	0.8434	0.9674	0.41
hsa-miR-4436b-5p	3.47	3.67	1.37	1.21	0.95	-0.08	0.8866	0.9605	0.8402	0.9674	0.49
hsa-miR-4734	1.29	1.33	0.35	0.45	0.97	-0.05	0.8446	0.9536	0.8420	0.9674	0.48
hsa-miR-4750-3p	2.82	2.87	0.53	0.46	0.99	-0.02	0.7687	0.9306	0.8427	0.9674	0.53
hsa-miR-4780	1.40	1.55	1.67	0.46	0.90	-0.15	0.0404	0.2242	0.8443	0.9674	0.67
hsa-miR-4455	1.74	1.46	0.32	0.58	1.19	0.25	0.1995	0.4538	0.8462	0.9677	0.39
hsa-miR-1260b	1.85	2.28	1.77	0.52	0.81	-0.31	0.0638	0.2525	0.8562	0.9685	0.66
hsa-miR-25-3p	1.77	1.80	0.31	0.20	0.98	-0.02	0.8516	0.9554	0.8524	0.9685	0.52
hsa-miR-320c	1.26	1.22	1.39	1.26	1.04	0.05	0.9574	0.9799	0.8557	0.9685	0.49
hsa-miR-4700-3p	2.63	2.60	0.53	0.66	1.01	0.02	0.8307	0.9482	0.8539	0.9685	0.48
hsa-miR-4731-3p	2.99	3.12	0.63	0.55	0.96	-0.06	0.7349	0.9110	0.8509	0.9685	0.53
hsa-miR-6847-3p	1.46	1.50	0.95	0.54	0.97	-0.05	0.9574	0.9799	0.8489	0.9685	0.51
hsa-miR-4695-3p	1.29	1.65	3.23	1.74	0.78	-0.35	0.0167	0.1644	0.8585	0.9693	0.70
hsa-miR-1273g-3p	2.40	2.73	1.44	1.28	0.88	-0.19	0.4704	0.7415	0.8703	0.9707	0.56

hsa-miR-135a-3p	1.75	1.82	0.32	0.39	0.96	-0.06	0.8866	0.9605	0.8738	0.9707	0.51
hsa-miR-4746-3p	1.17	1.06	0.55	0.47	1.10	0.13	0.8049	0.9436	0.8717	0.9707	0.52
hsa-miR-6089	70.83	70.83	47.79	44.72	1.00	0.00	0.9926	0.9945	0.8725	0.9707	0.50
hsa-miR-6515-3p	6.75	6.66	2.26	1.88	1.01	0.02	0.8655	0.9564	0.8630	0.9707	0.48
hsa-miR-6775-3p	2.94	3.00	0.57	0.41	0.98	-0.03	0.9361	0.9721	0.8715	0.9707	0.51
hsa-miR-6785-5p	2.56	2.70	1.71	2.02	0.95	-0.08	0.9432	0.9762	0.8649	0.9707	0.49
hsa-miR-6786-5p	1.29	1.34	0.65	0.61	0.97	-0.05	1.0000	1.0000	0.8692	0.9707	0.50
hsa-miR-6825-3p	1.38	1.95	2.52	0.35	0.71	-0.49	0.0086	0.1644	0.8716	0.9707	0.72
hsa-miR-18b-3p	2.30	2.27	0.29	0.20	1.01	0.02	0.8866	0.9605	0.8908	0.9762	0.51
hsa-miR-3620-3p	2.95	2.98	0.67	0.49	0.99	-0.01	0.9645	0.9819	0.8871	0.9762	0.50
hsa-miR-3646	2.58	2.55	0.43	0.26	1.01	0.02	0.9219	0.9703	0.8883	0.9762	0.51
hsa-miR-4312	3.03	2.99	0.77	0.76	1.01	0.02	0.8866	0.9605	0.8811	0.9762	0.51
hsa-miR-642b-3p	2.51	2.52	0.67	0.81	1.00	0.00	0.8237	0.9438	0.8924	0.9762	0.48
hsa-miR-6511a-3p	1.46	1.70	6.04	3.74	0.86	-0.22	0.0123	0.1644	0.8928	0.9762	0.71
hsa-miR-6787-3p	1.97	1.96	0.95	0.48	1.01	0.01	0.6431	0.8759	0.8855	0.9762	0.54
hsa-miR-6857-3p	2.76	2.61	0.49	0.40	1.06	0.08	0.7349	0.9110	0.8867	0.9762	0.47
hsa-miR-766-3p	4.01	4.06	1.01	0.87	0.99	-0.02	0.8237	0.9438	0.8925	0.9762	0.52
hsa-miR-6785-3p	3.22	3.18	0.95	0.94	1.01	0.02	0.7687	0.9306	0.8966	0.9785	0.53
hsa-miR-6877-3p	2.84	2.84	0.79	0.52	1.00	0.00	0.6753	0.8962	0.8991	0.9795	0.54
hsa-miR-130a-5p	1.39	1.33	0.52	0.47	1.04	0.06	0.8446	0.9536	0.9032	0.9796	0.48
hsa-miR-3074-5p	1.29	1.30	0.40	0.30	0.99	-0.01	0.8866	0.9605	0.9016	0.9796	0.51
hsa-miR-3940-3p	2.61	2.64	0.39	0.32	0.99	-0.02	0.8168	0.9438	0.9039	0.9796	0.48
hsa-miR-26a-2-3p	2.06	1.90	0.30	0.40	1.08	0.11	0.9017	0.9655	0.9097	0.9825	0.49
hsa-miR-7974	3.34	2.82	0.79	1.18	1.18	0.24	0.5328	0.7916	0.9097	0.9825	0.45
hsa-let-7b-3p	4.40	4.60	4.53	2.41	0.96	-0.06	0.7282	0.9110	0.9372	0.9834	0.53
hsa-let-7e-3p	1.54	1.82	1.82	0.41	0.84	-0.25	0.0355	0.2119	0.9623	0.9834	0.68
hsa-miR-1202	13.07	12.41	13.10	12.96	1.05	0.07	0.9929	0.9945	0.9450	0.9834	0.50
hsa-miR-1224-5p	1.96	2.43	1.44	1.54	0.81	-0.31	0.9574	0.9799	0.9319	0.9834	0.49
hsa-miR-1234-3p	7.58	7.17	3.41	2.57	1.06	0.08	0.9290	0.9703	0.9508	0.9834	0.51
hsa-miR-1915-3p	6.56	6.56	4.30	4.85	1.00	0.00	0.9219	0.9703	0.9503	0.9834	0.49
hsa-miR-3150b-5p	2.53	2.51	0.55	0.47	1.01	0.01	0.8516	0.9554	0.9448	0.9834	0.48
hsa-miR-3162-3p	8.54	8.26	6.98	7.10	1.03	0.05	0.9219	0.9703	0.9419	0.9834	0.51

hsa-miR-328-3p	2.21	2.73	7.17	2.57	0.81	-0.30	0.0028	0.1269	0.9638	0.9834	0.75
hsa-miR-3663-3p	1.67	1.47	0.92	0.92	1.14	0.18	0.9503	0.9799	0.9133	0.9834	0.51
hsa-miR-3665	3.91	4.87	4.69	4.52	0.80	-0.32	0.8376	0.9527	0.9519	0.9834	0.52
hsa-miR-371b-5p	2.08	1.89	2.35	2.49	1.10	0.14	0.9219	0.9703	0.9688	0.9834	0.51
hsa-miR-4270	2.93	2.71	1.52	1.67	1.08	0.11	0.8099	0.9436	0.9531	0.9834	0.48
hsa-miR-4274	2.86	2.82	0.51	0.43	1.01	0.02	0.8099	0.9436	0.9260	0.9834	0.48
hsa-miR-4442	2.31	2.19	0.75	1.00	1.05	0.08	0.8030	0.9436	0.9516	0.9834	0.48
hsa-miR-4456	1.44	1.31	0.34	0.41	1.10	0.14	0.6848	0.8982	0.9707	0.9834	0.46
hsa-miR-4465	3.15	3.06	3.79	3.50	1.03	0.04	0.6950	0.9021	0.9303	0.9834	0.47
hsa-miR-4481	1.08	0.87	0.63	1.16	1.24	0.31	0.1107	0.3275	0.9288	0.9834	0.36
hsa-miR-4507	2.61	2.84	1.49	1.99	0.92	-0.12	0.6689	0.8952	0.9644	0.9834	0.46
hsa-miR-4652-3p	3.24	3.16	0.98	1.14	1.02	0.04	0.6884	0.8982	0.9574	0.9834	0.47
hsa-miR-4668-5p	1.27	1.06	0.30	0.60	1.19	0.25	0.1189	0.3342	0.9488	0.9834	0.37
hsa-miR-4741	1.57	1.59	0.88	0.98	0.99	-0.02	0.9290	0.9703	0.9234	0.9834	0.49
hsa-miR-4754	0.73	0.64	0.25	0.47	1.13	0.18	0.3098	0.5882	0.9702	0.9834	0.41
hsa-miR-4763-3p	2.50	3.11	1.86	1.93	0.80	-0.31	0.9645	0.9819	0.9624	0.9834	0.50
hsa-miR-4767	2.55	2.53	0.49	0.38	1.01	0.01	0.9716	0.9859	0.9408	0.9834	0.50
hsa-miR-504-5p	1.43	1.50	0.24	0.18	0.95	-0.07	0.5097	0.7720	0.9404	0.9834	0.56
hsa-miR-642a-3p	7.29	6.56	3.26	3.19	1.11	0.15	0.9148	0.9703	0.9477	0.9834	0.51
hsa-miR-664b-3p	2.45	2.54	0.83	0.41	0.97	-0.05	0.5097	0.7720	0.9400	0.9834	0.56
hsa-miR-6732-3p	2.77	2.87	0.60	0.48	0.97	-0.05	0.9645	0.9819	0.9379	0.9834	0.50
hsa-miR-6789-5p	0.93	1.11	0.61	0.64	0.84	-0.25	0.8446	0.9536	0.9277	0.9834	0.48
hsa-miR-6791-5p	2.65	2.54	2.82	3.36	1.05	0.06	0.6754	0.8962	0.9702	0.9834	0.46
hsa-miR-6798-3p	3.02	3.12	0.44	0.42	0.97	-0.05	0.9007	0.9655	0.9555	0.9834	0.51
hsa-miR-6813-3p	5.65	5.71	1.73	1.69	0.99	-0.02	0.9361	0.9721	0.9544	0.9834	0.51
hsa-miR-6826-5p	1.43	1.12	0.39	1.19	1.28	0.35	0.0143	0.1644	0.9305	0.9834	0.29
hsa-miR-7109-3p	1.41	1.79	2.66	0.57	0.79	-0.35	0.0084	0.1644	0.9685	0.9834	0.72
hsa-miR-7114-3p	3.04	3.18	0.66	0.60	0.96	-0.07	0.9929	0.9945	0.9299	0.9834	0.50
hsa-miR-767-3p	2.74	2.69	0.48	0.44	1.02	0.03	0.8585	0.9554	0.9625	0.9834	0.52
hsa-miR-940	10.43	9.98	8.29	8.92	1.04	0.06	0.9502	0.9799	0.9320	0.9834	0.49
hsa-miR-4634	1.41	1.42	0.68	0.60	0.99	-0.01	0.8586	0.9554	0.9756	0.9862	0.52
hsa-miR-5001-5p	2.49	2.59	1.76	2.05	0.96	-0.06	0.6819	0.8972	0.9775	0.9862	0.46

hsa-miR-6870-3p	3.36	3.28	0.62	0.65	1.02	0.04	0.7349	0.9110	0.9783	0.9862	0.47
hsa-miR-6803-5p	3.50	3.28	2.18	2.67	1.07	0.09	0.7349	0.9110	0.9819	0.9882	0.47
hsa-miR-3196	1.72	1.68	0.64	0.81	1.03	0.04	0.8376	0.9527	0.9862	0.9910	0.48
hsa-miR-23c	3.74	3.50	1.10	1.30	1.07	0.10	0.7961	0.9381	0.9939	0.9971	0.48
hsa-miR-191-3p	5.15	5.15	2.00	1.93	1.00	0.00	0.9787	0.9914	0.9988	0.9994	0.50
hsa-miR-3190-5p	3.25	3.49	0.80	0.89	0.93	-0.10	0.8726	0.9605	0.9994	0.9994	0.49

Supplemental Table 3: MiRNA detected in the negative embryonic culture media (media not exposed to embryo).

**miRNA**	**Negative Control (1)**	**Negative Control (2)**
hsa-miR-548aj-3p	1	0
hsa-miR-933	1	1
hsa-miR-1246	1	0
hsa-miR-3162-3p	1	1
hsa-miR-6089	1	1
hsa-miR-93-3p	1	1
hsa-miR-892b	1	0
hsa-miR-6731-3p	1	0
hsa-miR-6737-3p	1	1
hsa-miR-6813-3p	1	0
hsa-miR-6797-3p	1	0
hsa-miR-4426	1	0
hsa-miR-940	1	1
hsa-miR-1825	1	1
hsa-miR-4274	1	1
hsa-miR-4769-3p	1	1
hsa-miR-542-3p	1	0
hsa-miR-556-5p	1	0
hsa-miR-576-3p	1	0
hsa-miR-1281	1	1
hsa-miR-1238-3p	1	1
hsa-miR-625-3p	1	0
hsa-miR-26a-2-3p	1	0
hsa-miR-6508-5p	1	0

hsa-miR-6889-3p	1	0
hsa-miR-4281	1	1
hsa-miR-6085	1	0
hsa-miR-4649-3p	1	0

Supplemental Table 4: Embryonic culture media and spermatozoa shared miRNAs

**miRNAs**			
hsa-miR-15b-5p	hsa-miR-1290	hsa-miR-623	hsa-miR-3196
hsa-miR-3154	hsa-miR-4286	hsa-miR-3180-5p	hsa-miR-4327
hsa-miR-3917	hsa-miR-762	hsa-miR-4271	hsa-miR-4281
hsa-miR-4299	hsa-miR-1249-3p	hsa-miR-191-3p	hsa-miR-1260b
hsa-miR-939-5p	hsa-miR-1825	hsa-miR-324-3p	hsa-miR-3665
hsa-miR-4298	hsa-miR-4313	hsa-miR-718	hsa-miR-320a
hsa-miR-3679-5p	hsa-let-7a-5p	hsa-miR-3162-5p	hsa-miR-1225-5p
hsa-miR-188-5p	hsa-miR-1281	hsa-miR-630	hsa-miR-371a-5p
hsa-miR-15a-5p	hsa-miR-574-5p	hsa-miR-129-2-3p	hsa-miR-483-5p
hsa-miR-3195	hsa-miR-2861	hsa-miR-4257	hsa-miR-150-3p
hsa-let-7c-5p	hsa-miR-1246	hsa-miR-642b-3p	hsa-miR-19b-3p
hsa-miR-4284	hsa-miR-671-5p	hsa-miR-1202	hsa-miR-3937
hsa-miR-16-5p	hsa-miR-766-3p	hsa-miR-1914-3p	hsa-miR-494-3p
hsa-miR-638	hsa-miR-1275	hsa-miR-765	hsa-miR-1260a
hsa-miR-320d	hsa-miR-3656	hsa-miR-1207-5p	hsa-miR-3667-5p
hsa-miR-1268a	hsa-miR-1234-3p	hsa-miR-4270	hsa-miR-1915-3p
hsa-miR-21-5p	hsa-miR-1238-3p	hsa-miR-134-5p	hsa-miR-3663-3p
hsa-miR-572	hsa-miR-1299	hsa-miR-320c	hsa-miR-3188
hsa-let-7f-5p	hsa-miR-940	hsa-miR-3652	hsa-miR-2276-3p
hsa-miR-1224-5p	hsa-miR-149-5p	hsa-miR-1228-3p	hsa-miR-135a-3p
hsa-miR-3141	hsa-miR-33b-3p	hsa-miR-498	

Supplemental Table 5: MiRNA enrichment Analysis

Category	Subcategory	Enrichment	p-value	#miRNAs	miRNAs/precursors
Immune cells	CD19 expressed	enriched	0.000100819	118	hsa-miR-29c-3p; hsa-let-7c-5p; hsa-miR-155-5p; hsa-let-7a-5p; hsa-miR-584-5p; hsa-miR-324-3p; hsa-let-7f-5p; hsa-miR-30d-5p; hsa-miR-4298; hsa-miR-4257; hsa-miR-26b-5p; hsa-miR-320a; hsa-let-7g-5p; hsa-miR-4306; hsa-miR-103a-3p; hsa-miR-1290; hsa-miR-106b-5p; hsa-miR-18b-5p; hsa-let-7i-5p; hsa-miR-1246; hsa-miR-550a-5p; hsa-miR-760; hsa-miR-451a; hsa-miR-320d; hsa-miR-4271; hsa-miR-146a-5p; hsa-miR-142-3p; hsa-miR-425-3p; hsa-miR-107; hsa-miR-16-5p; hsa-miR-20a-5p; hsa-miR-24-3p; hsa-miR-150-3p; hsa-miR-3679-5p; hsa-miR-33b-3p; hsa-miR-1539; hsa-miR-29b-3p; hsa-miR-933; hsa-miR-3180-5p; hsa-miR-671-5p; hsa-miR-939-5p; hsa-miR-4299; hsa-miR-365a-3p; hsa-miR-181a-5p; hsa-miR-181d-5p; hsa-miR-93-5p; hsa-miR-1207-5p; hsa-miR-181b-5p; hsa-miR-4284; hsa-miR-21-5p; hsa-miR-223-3p; hsa-miR-29a-3p; hsa-miR-26a-5p; hsa-miR-1238-3p; hsa-miR-2116-3p; hsa-miR-19a-3p; hsa-miR-4310; hsa-miR-3162-5p; hsa-miR-4286; hsa-miR-4323; hsa-miR-1914-3p; hsa-miR-15a-5p; hsa-miR-23c; hsa-miR-1825; hsa-miR-1268a; hsa-miR-642b-3p; hsa-miR-3196; hsa-miR-20b-5p; hsa-miR-1275; hsa-miR-1915-3p; hsa-miR-148a-3p; hsa-miR-940; hsa-miR-3195; hsa-miR-320c; hsa-miR-191-3p; hsa-miR-1202; hsa-miR-3663-3p; hsa-miR-19b-3p; hsa-miR-494-3p; hsa-miR-1234-3p; hsa-miR-486-5p; hsa-miR-1228-3p; hsa-miR-25-3p; hsa-miR-3665; hsa-miR-766-3p; hsa-miR-17-5p; hsa-miR-29c-5p; hsa-miR-634; hsa-let-7b-3p; hsa-miR-18a-5p; hsa-miR-4313; hsa-miR-4281; hsa-miR-572; hsa-miR-15b-5p; hsa-miR-762; hsa-miR-148b-3p; hsa-miR-3656; hsa-miR-149-5p; hsa-let-7f-1-3p; hsa-miR-23a-3p; hsa-miR-129-2-3p; hsa-miR-1281; hsa-miR-1249-3p; hsa-miR-142-5p; hsa-miR-638; hsa-miR-2861; hsa-miR-574-5p; hsa-miR-92a-3p; hsa-miR-1225-5p; hsa-miR-1237-3p; hsa-miR-505-3p; hsa-miR-1260a; hsa-miR-574-3p; hsa-miR-1260b; hsa-miR-30b-5p; hsa-miR-331-3p; hsa-miR-197-3p; hsa-miR-30c-5p

Immune cells	CD56 expressed	enriched	0.000100819	147	hsa-miR-29c-3p; hsa-miR-22-5p; hsa-let-7c-5p; hsa-miR-155-5p; hsa-let-7a-5p; hsa-miR-584-5p; hsa-miR-324-3p; hsa-let-7f-5p; hsa-miR-30d-5p; hsa-miR-4298; hsa-miR-4257; hsa-miR-26b-5p; hsa-miR-320a; hsa-let-7g-5p; hsa-miR-4306; hsa-miR-103a-3p; hsa-miR-1290; hsa-miR-1227-3p; hsa-miR-199b-5p; hsa-miR-106b-5p; hsa-miR-18b-5p; hsa-let-7i-5p; hsa-miR-3652; hsa-miR-3188; hsa-miR-1246; hsa-miR-3156-5p; hsa-miR-550a-5p; hsa-miR-451a; hsa-miR-320d; hsa-miR-4271; hsa-miR-146a-5p; hsa-miR-142-3p; hsa-miR-425-3p; hsa-miR-107; hsa-miR-16-5p; hsa-miR-20a-5p; hsa-miR-24-3p; hsa-miR-92b-3p; hsa-miR-150-3p; hsa-miR-3679-5p; hsa-miR-33b-3p; hsa-miR-1539; hsa-miR-29b-3p; hsa-miR-933; hsa-miR-3180-5p; hsa-miR-671-5p; hsa-miR-939-5p; hsa-miR-4299; hsa-miR-365a-3p; hsa-miR-4327; hsa-miR-3141; hsa-miR-181a-5p; hsa-miR-181d-5p; hsa-miR-93-5p; hsa-miR-1207-5p; hsa-miR-181b-5p; hsa-miR-4284; hsa-miR-21-5p; hsa-miR-223-3p; hsa-miR-29a-3p; hsa-miR-26a-5p; hsa-miR-1238-3p; hsa-miR-2116-3p; hsa-miR-134-5p; hsa-miR-19a-3p; hsa-miR-4310; hsa-miR-1229-3p; hsa-miR-3162-5p; hsa-miR-1267; hsa-miR-4286; hsa-miR-4323; hsa-miR-188-5p; hsa-miR-1914-3p; hsa-miR-15a-5p; hsa-miR-23c; hsa-miR-4270; hsa-miR-1825; hsa-miR-1268a; hsa-miR-642b-3p; hsa-miR-3196; hsa-miR-20b-5p; hsa-miR-1275; hsa-miR-1915-3p; hsa-miR-148a-3p; hsa-miR-940; hsa-miR-3195; hsa-miR-320c; hsa-miR-1224-5p; hsa-miR-191-3p; hsa-miR-1202; hsa-miR-3663-3p; hsa-miR-19b-3p; hsa-miR-494-3p; hsa-miR-1234-3p; hsa-miR-135a-3p; hsa-miR-4312; hsa-miR-18b-3p; hsa-miR-1228-3p; hsa-miR-25-3p; hsa-miR-3665; hsa-miR-3675-3p; hsa-miR-766-3p; hsa-miR-17-5p; hsa-miR-29c-5p; hsa-miR-3614-5p; hsa-miR-634; hsa-let-7b-3p; hsa-miR-18a-5p; hsa-miR-4313; hsa-miR-4281; hsa-miR-630; hsa-miR-572; hsa-miR-10a-5p; hsa-miR-129-1-3p; hsa-miR-15b-5p; hsa-miR-762; hsa-miR-148b-3p; hsa-miR-3656; hsa-miR-149-5p; hsa-let-7f-1-3p; hsa-miR-23a-3p; hsa-miR-129-2-3p; hsa-miR-1281; hsa-miR-26b-3p; hsa-miR-1249-3p; hsa-miR-142-5p; hsa-miR-638; hsa-miR-2861; hsa-miR-574-5p; hsa-miR-602; hsa-miR-92a-3p; hsa-miR-3679-3p; hsa-miR-1225-5p; hsa-miR-3613-3p; hsa-miR-563; hsa-miR-1237-3p; hsa-miR-505-3p; hsa-miR-1260a; hsa-miR-4261; hsa-miR-574-3p; hsa-miR-1260b; hsa-miR-30b-5p; hsa-miR-181a-2-3p; hsa-miR-331-3p; hsa-miR-197-3p; hsa-miR-30c-5p; hsa-miR-296-5p

Immune cells	CD15 expressed	enriched	0.000159956	156	hsa-miR-29c-3p; hsa-miR-22-5p; hsa-let-7c-5p; hsa-miR-155-5p; hsa-miR-193b-5p; hsa-let-7a-5p; hsa-miR-324-3p; hsa-let-7f-5p; hsa-miR-193a-5p; hsa-miR-30d-5p; hsa-miR-3937; hsa-miR-2276-3p; hsa-miR-4298; hsa-miR-4257; hsa-miR-26b-5p; hsa-miR-320a; hsa-let-7g-5p; hsa-miR-4306; hsa-miR-103a-3p; hsa-miR-1290; hsa-miR-1227-3p; hsa-miR-199b-5p; hsa-miR-106b-5p; hsa-miR-18b-5p; hsa-let-7i-5p; hsa-miR-3652; hsa-miR-3188; hsa-miR-1246; hsa-miR-3156-5p; hsa-miR-550a-5p; hsa-miR-760; hsa-miR-451a; hsa-miR-320d; hsa-miR-4271; hsa-miR-146a-5p; hsa-miR-142-3p; hsa-miR-425-3p; hsa-miR-107; hsa-miR-16-5p; hsa-miR-20a-5p; hsa-miR-24-3p; hsa-miR-548d-5p; hsa-miR-92b-3p; hsa-miR-150-3p; hsa-miR-3679-5p; hsa-miR-33b-3p; hsa-miR-1539; hsa-miR-29b-3p; hsa-miR-933; hsa-miR-3180-5p; hsa-miR-671-5p; hsa-miR-939-5p; hsa-miR-4299; hsa-miR-365a-3p; hsa-miR-4327; hsa-miR-3141; hsa-miR-181a-5p; hsa-miR-483-5p; hsa-miR-181d-5p; hsa-miR-93-5p; hsa-miR-1207-5p; hsa-miR-181b-5p; hsa-miR-4284; hsa-miR-21-5p; hsa-miR-223-3p; hsa-miR-29a-3p; hsa-miR-26a-5p; hsa-miR-1238-3p; hsa-miR-2116-3p; hsa-miR-134-5p; hsa-miR-19a-3p; hsa-miR-181c-3p; hsa-miR-4310; hsa-miR-3162-5p; hsa-miR-4286; hsa-miR-4323; hsa-miR-188-5p; hsa-miR-1914-3p; hsa-miR-15a-5p; hsa-miR-23c; hsa-miR-4274; hsa-miR-4270; hsa-miR-1825; hsa-miR-1268a; hsa-miR-642b-3p; hsa-miR-3196; hsa-miR-20b-5p; hsa-miR-1275; hsa-miR-1915-3p; hsa-miR-148a-3p; hsa-miR-940; hsa-miR-3195; hsa-miR-320c; hsa-miR-1224-5p; hsa-miR-191-3p; hsa-miR-1202; hsa-miR-3663-3p; hsa-miR-19b-3p; hsa-miR-494-3p; hsa-miR-1234-3p; hsa-miR-135a-3p; hsa-miR-4312; hsa-miR-18b-3p; hsa-miR-486-5p; hsa-miR-1228-3p; hsa-miR-25-3p; hsa-miR-3665; hsa-miR-766-3p; hsa-miR-17-5p; hsa-miR-29c-5p; hsa-miR-3614-5p; hsa-miR-634; hsa-let-7b-3p; hsa-miR-18a-5p; hsa-miR-4313; hsa-miR-4281; hsa-miR-630; hsa-miR-572; hsa-miR-15b-5p; hsa-miR-762; hsa-miR-148b-3p; hsa-miR-3656; hsa-miR-149-5p; hsa-miR-663a; hsa-let-7f-1-3p; hsa-miR-23a-3p; hsa-miR-129-2-3p; hsa-miR-1281; hsa-miR-718; hsa-miR-26b-3p; hsa-miR-1249-3p; hsa-miR-142-5p; hsa-miR-638; hsa-miR-2861; hsa-miR-574-5p; hsa-miR-602; hsa-miR-92a-3p; hsa-miR-3679-3p; hsa-miR-1225-5p; hsa-miR-563; hsa-miR-1237-3p; hsa-miR-505-3p; hsa-miR-1260a; hsa-miR-4261; hsa-miR-629-3p; hsa-miR-574-3p; hsa-miR-1260b; hsa-miR-582-3p; hsa-miR-30b-5p; hsa-miR-331-3p; hsa-miR-197-3p; hsa-miR-744-3p; hsa-miR-30c-5p; hsa-miR-296-5p; hsa-let-7d-3p; hsa-miR-328-3p
Diseases (published studies about miRNA profiles from peripheral blood)	Alzheimers Disease upregulated	depleted	0.000210286	38	hsa-miR-550a-3-5p; hsa-miR-30d-5p; hsa-miR-550a-5p; hsa-miR-3180-5p; hsa-miR-26a-5p; hsa-miR-1908-3p; hsa-miR-371b-5p; hsa-miR-3074-5p; hsa-miR-766-3p; hsa-miR-29c-5p; hsa-miR-576-5p; hsa-let-7b-3p; hsa-miR-6797-3p; hsa-miR-10a-5p; hsa-miR-3605-3p; hsa-let-7f-1-3p; hsa-miR-664b-3p; hsa-miR-26b-3p; hsa-miR-937-3p; hsa-miR-3173-5p; hsa-miR-505-3p; hsa-miR-5010-3p; hsa-miR-1260a; hsa-miR-6866-3p; hsa-miR-758-3p; hsa-miR-1260b; hsa-miR-30b-5p; hsa-miR-130b-5p; hsa-miR-1910-5p; hsa-miR-6762-3p; hsa-miR-181a-2-3p; hsa-miR-6754-3p; hsa-miR-4512; hsa-miR-30c-5p; hsa-let-7d-3p; hsa-miR-4326; hsa-miR-328-3p; hsa-miR-1296-5p
Diseases (miRWalk)	Carcinoma Hepatocellular	enriched	0.000245173	22	hsa-miR-29c-3p; hsa-let-7c-5p; hsa-let-7a-5p; hsa-miR-30d-5p; hsa-miR-1-3p; hsa-let-7g-5p; hsa-miR-199b-5p; hsa-miR-106b-5p; hsa-miR-16-5p; hsa-miR-29b-3p; hsa-miR-181a-5p; hsa-miR-181d-5p; hsa-miR-93-5p; hsa-miR-181b-5p; hsa-miR-124-3p; hsa-miR-21-5p; hsa-miR-223-3p; hsa-miR-29a-3p; hsa-miR-26a-5p; hsa-miR-520a-3p; hsa-miR-18a-5p; hsa-miR-23a-3p

Diseases (published studies about miRNA profiles from peripheral blood)	psoriasis down regulated	enriched	0.000253203	32	hsa-miR-29c-3p; hsa-miR-22-5p; hsa-let-7c-5p; hsa-miR-155-5p; hsa-miR-584-5p; hsa-miR-324-3p; hsa-miR-26b-5p; hsa-let-7g-5p; hsa-miR-520d-3p; hsa-miR-106b-5p; hsa-let-7i-5p; hsa-miR-320d; hsa-miR-146a-5p; hsa-miR-548d-3p; hsa-miR-1299; hsa-miR-20a-5p; hsa-miR-29b-3p; hsa-miR-181a-5p; hsa-miR-93-5p; hsa-miR-181b-5p; hsa-miR-595; hsa-miR-21-5p; hsa-miR-29a-3p; hsa-miR-26a-5p; hsa-miR-20b-5p; hsa-miR-320c; hsa-miR-25-3p; hsa-miR-23a-3p; hsa-miR-142-5p; hsa-miR-521; hsa-miR-181a-2-3p; hsa-miR-331-3p
Immune cells	CD3 expressed	enriched	0.000925534	121	hsa-miR-29c-3p; hsa-let-7c-5p; hsa-miR-155-5p; hsa-let-7a-5p; hsa-miR-324-3p; hsa-let-7f-5p; hsa-miR-30d-5p; hsa-miR-4257; hsa-miR-26b-5p; hsa-miR-320a; hsa-let-7g-5p; hsa-miR-4306; hsa-miR-103a-3p; hsa-miR-1227-3p; hsa-miR-106b-5p; hsa-miR-18b-5p; hsa-let-7i-5p; hsa-miR-1246; hsa-miR-550a-5p; hsa-miR-451a; hsa-miR-320d; hsa-miR-146a-5p; hsa-miR-142-3p; hsa-miR-425-3p; hsa-miR-107; hsa-miR-16-5p; hsa-miR-20a-5p; hsa-miR-24-3p; hsa-miR-150-3p; hsa-miR-3679-5p; hsa-miR-33b-3p; hsa-miR-1539; hsa-miR-29b-3p; hsa-miR-933; hsa-miR-3180-5p; hsa-miR-939-5p; hsa-miR-4299; hsa-miR-365a-3p; hsa-miR-181a-5p; hsa-miR-181d-5p; hsa-miR-93-5p; hsa-miR-1207-5p; hsa-miR-181b-5p; hsa-miR-4284; hsa-miR-21-5p; hsa-miR-223-3p; hsa-miR-29a-3p; hsa-miR-26a-5p; hsa-miR-1238-3p; hsa-miR-2116-3p; hsa-miR-19a-3p; hsa-miR-4310; hsa-miR-3162-5p; hsa-miR-4286; hsa-miR-4323; hsa-miR-1914-3p; hsa-miR-15a-5p; hsa-miR-23c; hsa-miR-4270; hsa-miR-1825; hsa-miR-1268a; hsa-miR-642b-3p; hsa-miR-3196; hsa-miR-20b-5p; hsa-miR-1275; hsa-miR-1915-3p; hsa-miR-148a-3p; hsa-miR-940; hsa-miR-3195; hsa-miR-320c; hsa-miR-191-3p; hsa-miR-1202; hsa-miR-19b-3p; hsa-miR-494-3p; hsa-miR-1234-3p; hsa-miR-4312; hsa-miR-486-5p; hsa-miR-1228-3p; hsa-miR-25-3p; hsa-miR-3665; hsa-miR-766-3p; hsa-miR-17-5p; hsa-miR-29c-5p; hsa-miR-3614-5p; hsa-miR-634; hsa-let-7b-3p; hsa-miR-18a-5p; hsa-miR-4313; hsa-miR-4281; hsa-miR-572; hsa-miR-129-1-3p; hsa-miR-15b-5p; hsa-miR-762; hsa-miR-148b-3p; hsa-miR-3656; hsa-miR-149-5p; hsa-let-7f-1-3p; hsa-miR-23a-3p; hsa-miR-129-2-3p; hsa-miR-1281; hsa-miR-26b-3p; hsa-miR-1249-3p; hsa-miR-142-5p; hsa-miR-638; hsa-miR-2861; hsa-miR-574-5p; hsa-miR-92a-3p; hsa-miR-3679-3p; hsa-miR-1225-5p; hsa-miR-563; hsa-miR-1237-3p; hsa-miR-505-3p; hsa-miR-1260a; hsa-miR-4261; hsa-miR-574-3p; hsa-miR-1260b; hsa-miR-30b-5p; hsa-miR-331-3p; hsa-miR-197-3p; hsa-miR-30c-5p; hsa-miR-296-5p
Pathways (miRWalk)	P00046 Oxidative stress response	enriched	0.00209572	44	hsa-miR-29c-3p; hsa-let-7c-5p; hsa-miR-155-5p; hsa-let-7a-5p; hsa-miR-324-3p; hsa-let-7f-5p; hsa-miR-1-3p; hsa-miR-877-5p; hsa-miR-26b-5p; hsa-miR-320a; hsa-let-7g-5p; hsa-miR-103a-3p; hsa-miR-106b-5p; hsa-miR-760; hsa-miR-451a; hsa-miR-146a-5p; hsa-miR-16-5p; hsa-miR-20a-5p; hsa-miR-24-3p; hsa-miR-29b-3p; hsa-miR-365a-3p; hsa-miR-181a-5p; hsa-miR-181d-5p; hsa-miR-93-5p; hsa-miR-181b-5p; hsa-miR-124-3p; hsa-miR-21-5p; hsa-miR-223-3p; hsa-miR-29a-3p; hsa-miR-26a-5p; hsa-miR-15a-5p; hsa-miR-449c-5p; hsa-miR-148a-3p; hsa-miR-19b-3p; hsa-miR-17-5p; hsa-miR-630; hsa-miR-10a-5p; hsa-miR-15b-5p; hsa-miR-149-5p; hsa-miR-92a-3p; hsa-miR-615-3p; hsa-miR-204-5p; hsa-miR-34b-3p; hsa-miR-296-5p
Pathways (miRWalk)	WP2018 RANKL RANK Signaling Pathway	enriched	0.00209572	44	hsa-miR-29c-3p; hsa-let-7c-5p; hsa-miR-155-5p; hsa-let-7a-5p; hsa-let-7f-5p; hsa-miR-30d-5p; hsa-miR-1-3p; hsa-miR-877-5p; hsa-miR-26b-5p; hsa-miR-320a; hsa-miR-103a-3p; hsa-miR-106b-5p; hsa-miR-451a; hsa-miR-184; hsa-miR-146a-5p; hsa-miR-142-3p; hsa-miR-16-5p; hsa-miR-20a-5p; hsa-miR-24-3p; hsa-miR-92b-3p; hsa-miR-29b-3p; hsa-miR-181a-5p; hsa-miR-483-5p; hsa-miR-93-5p; hsa-miR-181b-5p; hsa-miR-124-3p; hsa-miR-21-5p; hsa-miR-223-3p; hsa-miR-29a-3p; hsa-miR-99b-3p; hsa-miR-1914-3p; hsa-miR-15a-5p; hsa-

					miR-766-3p; hsa-miR-17-5p; hsa-miR-18a-5p; hsa-miR-10a-5p; hsa-miR-15b-5p; hsa-miR-149-5p; hsa-miR-92a-3p; hsa-miR-615-3p; hsa-miR-331-3p; hsa-miR-744-3p; hsa-miR-30c-5p; hsa-miR-1296-5p
Pathways (miRWalk)	WP364 IL 6 signaling pathway	enriched	0.00209572	44	hsa-let-7c-5p; hsa-miR-155-5p; hsa-let-7a-5p; hsa-miR-324-3p; hsa-let-7f-5p; hsa-miR-30d-5p; hsa-miR-1-3p; hsa-miR-877-5p; hsa-miR-26b-5p; hsa-miR-320a; hsa-let-7g-5p; hsa-miR-103a-3p; hsa-miR-106b-5p; hsa-miR-451a; hsa-miR-146a-5p; hsa-miR-142-3p; hsa-miR-16-5p; hsa-miR-20a-5p; hsa-miR-24-3p; hsa-miR-92b-3p; hsa-miR-671-5p; hsa-miR-365a-3p; hsa-miR-181a-5p; hsa-miR-483-5p; hsa-miR-93-5p; hsa-miR-181b-5p; hsa-miR-124-3p; hsa-miR-21-5p; hsa-miR-29a-3p; hsa-miR-26a-5p; hsa-miR-20b-5p; hsa-miR-766-3p; hsa-miR-17-5p; hsa-miR-15b-5p; hsa-miR-663a; hsa-miR-23a-3p; hsa-miR-92a-3p; hsa-miR-615-3p; hsa-miR-30b-5p; hsa-miR-130b-5p; hsa-miR-337-3p; hsa-miR-1226-3p; hsa-miR-346; hsa-miR-30c-5p
Pathways (miRWalk)	hsa04662 B cell receptor signaling pathway	enriched	0.00209572	43	hsa-miR-29c-3p; hsa-let-7c-5p; hsa-miR-155-5p; hsa-let-7a-5p; hsa-miR-324-3p; hsa-miR-1-3p; hsa-miR-877-5p; hsa-miR-26b-5p; hsa-miR-320a; hsa-let-7g-5p; hsa-miR-106b-5p; hsa-miR-451a; hsa-miR-184; hsa-miR-146a-5p; hsa-miR-142-3p; hsa-miR-16-5p; hsa-miR-20a-5p; hsa-miR-24-3p; hsa-miR-92b-3p; hsa-miR-29b-3p; hsa-miR-181a-5p; hsa-miR-483-5p; hsa-miR-181d-5p; hsa-miR-93-5p; hsa-miR-124-3p; hsa-miR-21-5p; hsa-miR-223-3p; hsa-miR-29a-3p; hsa-miR-26a-5p; hsa-miR-99b-3p; hsa-miR-15a-5p; hsa-miR-766-3p; hsa-miR-17-5p; hsa-miR-15b-5p; hsa-miR-148b-3p; hsa-miR-149-5p; hsa-miR-92a-3p; hsa-miR-615-3p; hsa-miR-1260b; hsa-miR-331-3p; hsa-miR-346; hsa-miR-30c-5p; hsa-miR-1296-5p
Diseases (miRWalk)	Carcinoma	enriched	0.00210311	42	hsa-miR-29c-3p; hsa-let-7c-5p; hsa-let-7a-5p; hsa-miR-584-5p; hsa-let-7f-5p; hsa-miR-193a-5p; hsa-miR-30d-5p; hsa-miR-1-3p; hsa-miR-26b-5p; hsa-let-7g-5p; hsa-miR-199b-5p; hsa-miR-106b-5p; hsa-miR-451a; hsa-miR-184; hsa-miR-146a-5p; hsa-miR-107; hsa-miR-16-5p; hsa-miR-24-3p; hsa-miR-29b-3p; hsa-miR-181a-5p; hsa-miR-181d-5p; hsa-miR-93-5p; hsa-miR-181b-5p; hsa-miR-124-3p; hsa-miR-21-5p; hsa-miR-223-3p; hsa-miR-29a-3p; hsa-miR-26a-5p; hsa-miR-19a-3p; hsa-miR-15a-5p; hsa-miR-520a-3p; hsa-miR-19b-3p; hsa-miR-17-5p; hsa-miR-18a-5p; hsa-miR-630; hsa-miR-23a-3p; hsa-miR-516a-3p; hsa-miR-204-5p; hsa-miR-1226-3p; hsa-miR-197-3p; hsa-miR-346; hsa-miR-296-5p
Diseases (miRWalk)	Polycythemia	enriched	0.00210311	3	hsa-let-7c-5p; hsa-let-7a-5p; hsa-let-7f-5p
Diseases (miRWalk)	Primary Myelofibrosis	enriched	0.00210311	3	hsa-let-7c-5p; hsa-let-7a-5p; hsa-let-7f-5p
Diseases (miRWalk)	Thrombocythemia Essential	enriched	0.00210311	3	hsa-let-7c-5p; hsa-let-7a-5p; hsa-let-7f-5p
Diseases (miRWalk)	Thrombocytosis	enriched	0.00210311	3	hsa-let-7c-5p; hsa-let-7a-5p; hsa-let-7f-5p

Immune cells	CD14 expressed	enriched	0.00287092	131	hsa-miR-29c-3p; hsa-miR-22-5p; hsa-let-7c-5p; hsa-miR-155-5p; hsa-let-7a-5p; hsa-miR-324-3p; hsa-let-7f-5p; hsa-miR-30d-5p; hsa-miR-4257; hsa-miR-26b-5p; hsa-miR-320a; hsa-let-7g-5p; hsa-miR-4306; hsa-miR-103a-3p; hsa-miR-1290; hsa-miR-1227-3p; hsa-miR-199b-5p; hsa-miR-106b-5p; hsa-miR-18b-5p; hsa-let-7i-5p; hsa-miR-1246; hsa-miR-550a-5p; hsa-miR-451a; hsa-miR-320d; hsa-miR-146a-5p; hsa-miR-142-3p; hsa-miR-425-3p; hsa-miR-107; hsa-miR-16-5p; hsa-miR-20a-5p; hsa-miR-24-3p; hsa-miR-92b-3p; hsa-miR-3679-5p; hsa-miR-33b-3p; hsa-miR-1539; hsa-miR-29b-3p; hsa-miR-933; hsa-miR-3180-5p; hsa-miR-939-5p; hsa-miR-4299; hsa-miR-365a-3p; hsa-miR-181a-5p; hsa-miR-181d-5p; hsa-miR-93-5p; hsa-miR-1207-5p; hsa-miR-181b-5p; hsa-miR-4284; hsa-miR-21-5p; hsa-miR-223-3p; hsa-miR-29a-3p; hsa-miR-26a-5p; hsa-miR-1238-3p; hsa-miR-2116-3p; hsa-miR-19a-3p; hsa-miR-4310; hsa-miR-1229-3p; hsa-miR-3162-5p; hsa-miR-4286; hsa-miR-4323; hsa-miR-188-5p; hsa-miR-1914-3p; hsa-miR-15a-5p; hsa-miR-23c; hsa-miR-1825; hsa-miR-1268a; hsa-miR-642b-3p; hsa-miR-3196; hsa-miR-20b-5p; hsa-miR-1275; hsa-miR-1915-3p; hsa-miR-148a-3p; hsa-miR-940; hsa-miR-3195; hsa-miR-320c; hsa-miR-191-3p; hsa-miR-1202; hsa-miR-19b-3p; hsa-miR-494-3p; hsa-miR-1234-3p; hsa-miR-3646; hsa-miR-4312; hsa-miR-18b-3p; hsa-miR-1228-3p; hsa-miR-25-3p; hsa-miR-3665; hsa-miR-766-3p; hsa-miR-17-5p; hsa-miR-29c-5p; hsa-miR-3614-5p; hsa-miR-634; hsa-let-7b-3p; hsa-miR-18a-5p; hsa-miR-4313; hsa-miR-4281; hsa-miR-572; hsa-miR-129-1-3p; hsa-miR-15b-5p; hsa-miR-762; hsa-miR-148b-3p; hsa-miR-3656; hsa-miR-149-5p; hsa-let-7f-1-3p; hsa-miR-23a-3p; hsa-miR-129-2-3p; hsa-miR-1281; hsa-miR-26b-3p; hsa-miR-1249-3p; hsa-miR-142-5p; hsa-miR-638; hsa-miR-2861; hsa-miR-574-5p; hsa-miR-602; hsa-miR-92a-3p; hsa-miR-3679-3p; hsa-miR-1225-5p; hsa-miR-3613-3p; hsa-miR-563; hsa-miR-1237-3p; hsa-miR-505-3p; hsa-miR-1260a; hsa-miR-4261; hsa-miR-574-3p; hsa-miR-1260b; hsa-miR-582-3p; hsa-miR-30b-5p; hsa-miR-331-3p; hsa-miR-197-3p; hsa-miR-30c-5p; hsa-miR-296-5p; hsa-let-7d-3p; hsa-miR-328-3p
Diseases (miRWalk)	Fibrosis	enriched	0.0029063	12	hsa-miR-155-5p; hsa-let-7f-5p; hsa-miR-1-3p; hsa-let-7g-5p; hsa-miR-199b-5p; hsa-let-7i-5p; hsa-miR-16-5p; hsa-miR-29b-3p; hsa-miR-21-5p; hsa-miR-29a-3p; hsa-miR-26a-5p; hsa-miR-18a-5p
Gene Ontology (miRWalk)	GO0071158 positive regulation of cell cycle arrest	enriched	0.0041957	35	hsa-let-7c-5p; hsa-miR-155-5p; hsa-let-7a-5p; hsa-miR-324-3p; hsa-let-7f-5p; hsa-miR-30d-5p; hsa-miR-1-3p; hsa-miR-26b-5p; hsa-miR-320a; hsa-let-7g-5p; hsa-miR-103a-3p; hsa-miR-106b-5p; hsa-miR-760; hsa-miR-146a-5p; hsa-miR-16-5p; hsa-miR-20a-5p; hsa-miR-24-3p; hsa-miR-92b-3p; hsa-miR-671-5p; hsa-miR-365a-3p; hsa-miR-181a-5p; hsa-miR-93-5p; hsa-miR-181b-5p; hsa-miR-124-3p; hsa-miR-21-5p; hsa-miR-26a-5p; hsa-miR-99b-3p; hsa-miR-15a-5p; hsa-miR-25-3p; hsa-miR-17-5p; hsa-miR-504-5p; hsa-miR-92a-3p; hsa-miR-615-3p; hsa-miR-331-3p; hsa-miR-605-5p
Organs (miRWalk)	Stem Cells	enriched	0.0046131	26	hsa-miR-155-5p; hsa-let-7a-5p; hsa-miR-1-3p; hsa-miR-26b-5p; hsa-miR-320a; hsa-miR-199b-5p; hsa-miR-142-3p; hsa-miR-16-5p; hsa-miR-20a-5p; hsa-miR-24-3p; hsa-miR-92b-3p; hsa-miR-29b-3p; hsa-miR-181a-5p; hsa-miR-181d-5p; hsa-miR-181b-5p; hsa-miR-124-3p; hsa-miR-21-5p; hsa-miR-223-3p; hsa-miR-29a-3p; hsa-miR-26a-5p; hsa-miR-15a-5p; hsa-miR-148a-3p; hsa-miR-19b-3p; hsa-miR-17-5p; hsa-miR-92a-3p; hsa-miR-34b-3p

Pathways (miRWalk)	WP400 p38 MAPK Signaling Pathway	enriched	0.00582307	42	hsa-miR-29c-3p; hsa-let-7c-5p; hsa-miR-155-5p; hsa-let-7a-5p; hsa-let-7f-5p; hsa-miR-1-3p; hsa-miR-877-5p; hsa-miR-26b-5p; hsa-miR-320a; hsa-let-7g-5p; hsa-miR-103a-3p; hsa-miR-106b-5p; hsa-miR-451a; hsa-miR-146a-5p; hsa-miR-142-3p; hsa-miR-16-5p; hsa-miR-20a-5p; hsa-miR-24-3p; hsa-miR-92b-3p; hsa-miR-29b-3p; hsa-miR-181a-5p; hsa-miR-181d-5p; hsa-miR-93-5p; hsa-miR-124-3p; hsa-miR-21-5p; hsa-miR-29a-3p; hsa-miR-26a-5p; hsa-miR-19a-3p; hsa-miR-1229-3p; hsa-miR-15a-5p; hsa-miR-449c-5p; hsa-miR-148a-3p; hsa-miR-19b-3p; hsa-miR-17-5p; hsa-miR-10a-5p; hsa-miR-15b-5p; hsa-miR-615-3p; hsa-miR-30b-5p; hsa-miR-204-5p; hsa-miR-197-3p; hsa-miR-34b-3p; hsa-miR-30c-5p
Pathways (miRWalk)	hsa00350 Tyrosine metabolism	enriched	0.00582307	14	hsa-miR-155-5p; hsa-let-7a-5p; hsa-miR-1-3p; hsa-miR-26b-5p; hsa-miR-320a; hsa-miR-106b-5p; hsa-miR-451a; hsa-miR-16-5p; hsa-miR-24-3p; hsa-miR-181a-5p; hsa-miR-93-5p; hsa-miR-124-3p; hsa-miR-148b-3p; hsa-miR-92a-3p
Pathways (miRWalk)	hsa00630 Glyoxylate and dicarboxylate metabolism	enriched	0.00582307	16	hsa-miR-29c-3p; hsa-miR-155-5p; hsa-let-7a-5p; hsa-miR-30d-5p; hsa-miR-1-3p; hsa-miR-877-5p; hsa-miR-26b-5p; hsa-miR-320a; hsa-miR-106b-5p; hsa-miR-425-3p; hsa-miR-16-5p; hsa-miR-92b-3p; hsa-miR-93-5p; hsa-miR-940; hsa-miR-15b-5p; hsa-miR-615-3p
Pathways (miRWalk)	hsa05221 Acute myeloid leukemia	enriched	0.00582307	52	hsa-miR-29c-3p; hsa-let-7c-5p; hsa-miR-155-5p; hsa-let-7a-5p; hsa-miR-324-3p; hsa-let-7f-5p; hsa-miR-193a-5p; hsa-miR-1-3p; hsa-miR-877-5p; hsa-miR-26b-5p; hsa-miR-320a; hsa-let-7g-5p; hsa-miR-103a-3p; hsa-miR-106b-5p; hsa-miR-451a; hsa-miR-184; hsa-miR-146a-5p; hsa-miR-16-5p; hsa-miR-20a-5p; hsa-miR-24-3p; hsa-miR-92b-3p; hsa-miR-365a-3p; hsa-miR-181a-5p; hsa-miR-483-5p; hsa-miR-181d-5p; hsa-miR-93-5p; hsa-miR-181b-5p; hsa-miR-124-3p; hsa-miR-21-5p; hsa-miR-223-3p; hsa-miR-29a-3p; hsa-miR-26a-5p; hsa-miR-19a-3p; hsa-miR-1229-3p; hsa-miR-15a-5p; hsa-miR-449c-5p; hsa-miR-20b-5p; hsa-miR-766-3p; hsa-miR-17-5p; hsa-miR-15b-5p; hsa-miR-148b-3p; hsa-miR-149-5p; hsa-miR-92a-3p; hsa-miR-615-3p; hsa-miR-337-3p; hsa-miR-1226-3p; hsa-miR-331-3p; hsa-miR-346; hsa-miR-34b-3p; hsa-miR-30c-5p; hsa-miR-296-5p; hsa-miR-1296-5p
Pathways (miRWalk)	P00053 T cell activation	enriched	0.0066867	44	hsa-miR-29c-3p; hsa-let-7c-5p; hsa-miR-155-5p; hsa-let-7a-5p; hsa-miR-324-3p; hsa-miR-1-3p; hsa-miR-877-5p; hsa-miR-26b-5p; hsa-miR-320a; hsa-let-7g-5p; hsa-miR-106b-5p; hsa-miR-451a; hsa-miR-184; hsa-miR-146a-5p; hsa-miR-142-3p; hsa-miR-16-5p; hsa-miR-20a-5p; hsa-miR-24-3p; hsa-miR-92b-3p; hsa-miR-29b-3p; hsa-miR-181a-5p; hsa-miR-483-5p; hsa-miR-181d-5p; hsa-miR-93-5p; hsa-miR-124-3p; hsa-miR-21-5p; hsa-miR-223-3p; hsa-miR-29a-3p; hsa-miR-1914-3p; hsa-miR-15a-5p; hsa-miR-766-3p; hsa-miR-17-5p; hsa-miR-10a-5p; hsa-miR-15b-5p; hsa-miR-148b-3p; hsa-miR-149-5p; hsa-miR-92a-3p; hsa-miR-615-3p; hsa-miR-331-3p; hsa-miR-346; hsa-miR-744-3p; hsa-miR-34b-3p; hsa-miR-30c-5p; hsa-miR-1296-5p
Pathways (miRWalk)	P00010 B cell activation	enriched	0.00675359	39	hsa-miR-29c-3p; hsa-let-7c-5p; hsa-miR-155-5p; hsa-let-7a-5p; hsa-miR-324-3p; hsa-miR-1-3p; hsa-miR-877-5p; hsa-miR-26b-5p; hsa-miR-320a; hsa-miR-106b-5p; hsa-miR-184; hsa-miR-142-3p; hsa-miR-16-5p; hsa-miR-20a-5p; hsa-miR-24-3p; hsa-miR-92b-3p; hsa-miR-29b-3p; hsa-miR-181a-5p; hsa-miR-483-5p; hsa-miR-181d-5p; hsa-miR-93-5p; hsa-miR-124-3p; hsa-miR-21-5p; hsa-miR-223-3p; hsa-miR-26a-5p; hsa-miR-99b-3p; hsa-miR-15a-5p; hsa-miR-766-3p; hsa-miR-17-5p; hsa-miR-10a-5p; hsa-miR-15b-5p; hsa-miR-148b-3p; hsa-miR-149-5p; hsa-miR-92a-3p; hsa-miR-615-3p; hsa-miR-1226-3p; hsa-miR-331-3p; hsa-miR-346; hsa-miR-30c-5p

Pathways (miRWalk)	P00048 PI3 kinase pathway	enriched	0.00675359	57	hsa-miR-29c-3p; hsa-let-7c-5p; hsa-miR-155-5p; hsa-let-7a-5p; hsa-miR-324-3p; hsa-let-7f-5p; hsa-miR-30d-5p; hsa-miR-1-3p; hsa-miR-877-5p; hsa-miR-26b-5p; hsa-miR-320a; hsa-let-7g-5p; hsa-miR-103a-3p; hsa-miR-106b-5p; hsa-miR-451a; hsa-miR-184; hsa-miR-142-3p; hsa-miR-107; hsa-miR-16-5p; hsa-miR-20a-5p; hsa-miR-24-3p; hsa-miR-92b-3p; hsa-miR-29b-3p; hsa-miR-671-5p; hsa-miR-365a-3p; hsa-miR-181a-5p; hsa-miR-181d-5p; hsa-miR-93-5p; hsa-miR-181b-5p; hsa-miR-124-3p; hsa-miR-21-5p; hsa-miR-223-3p; hsa-miR-29a-3p; hsa-miR-26a-5p; hsa-miR-19a-3p; hsa-miR-1229-3p; hsa-miR-15a-5p; hsa-miR-148a-3p; hsa-miR-19b-3p; hsa-miR-494-3p; hsa-miR-17-5p; hsa-miR-18a-5p; hsa-miR-10a-5p; hsa-miR-15b-5p; hsa-miR-148b-3p; hsa-miR-149-5p; hsa-miR-23a-3p; hsa-miR-504-5p; hsa-miR-92a-3p; hsa-miR-615-3p; hsa-miR-331-3p; hsa-miR-197-3p; hsa-miR-346; hsa-miR-34b-3p; hsa-miR-30c-5p; hsa-miR-296-5p; hsa-miR-1296-5p
Pathways (miRWalk)	hsa04621 NOD like receptor signaling pathway	enriched	0.00900702	44	hsa-miR-155-5p; hsa-let-7a-5p; hsa-miR-324-3p; hsa-let-7f-5p; hsa-miR-30d-5p; hsa-miR-1-3p; hsa-miR-877-5p; hsa-miR-26b-5p; hsa-miR-320a; hsa-miR-103a-3p; hsa-miR-106b-5p; hsa-miR-760; hsa-miR-146a-5p; hsa-miR-16-5p; hsa-miR-20a-5p; hsa-miR-24-3p; hsa-miR-92b-3p; hsa-miR-365a-3p; hsa-miR-181a-5p; hsa-miR-483-5p; hsa-miR-181d-5p; hsa-miR-93-5p; hsa-miR-181b-5p; hsa-miR-124-3p; hsa-miR-21-5p; hsa-miR-223-3p; hsa-miR-29a-3p; hsa-miR-26a-5p; hsa-miR-15a-5p; hsa-miR-148a-3p; hsa-miR-25-3p; hsa-miR-766-3p; hsa-miR-17-5p; hsa-miR-10a-5p; hsa-miR-15b-5p; hsa-miR-148b-3p; hsa-miR-149-5p; hsa-miR-23a-3p; hsa-miR-92a-3p; hsa-miR-615-3p; hsa-miR-204-5p; hsa-miR-1226-3p; hsa-miR-346; hsa-miR-30c-5p
Pathways (miRWalk)	hsa04914 Progesterone mediated oocyte maturation	enriched	0.00900702	58	hsa-miR-29c-3p; hsa-let-7c-5p; hsa-miR-155-5p; hsa-let-7a-5p; hsa-miR-324-3p; hsa-let-7f-5p; hsa-miR-30d-5p; hsa-miR-1-3p; hsa-miR-877-5p; hsa-miR-26b-5p; hsa-miR-320a; hsa-let-7g-5p; hsa-miR-103a-3p; hsa-miR-1227-3p; hsa-miR-106b-5p; hsa-let-7i-5p; hsa-miR-760; hsa-miR-451a; hsa-miR-184; hsa-miR-146a-5p; hsa-miR-16-5p; hsa-miR-20a-5p; hsa-miR-24-3p; hsa-miR-92b-3p; hsa-miR-365a-3p; hsa-miR-181a-5p; hsa-miR-483-5p; hsa-miR-181d-5p; hsa-miR-93-5p; hsa-miR-181b-5p; hsa-miR-124-3p; hsa-miR-21-5p; hsa-miR-223-3p; hsa-miR-29a-3p; hsa-miR-1229-3p; hsa-miR-15a-5p; hsa-miR-148a-3p; hsa-miR-19b-3p; hsa-miR-25-3p; hsa-miR-766-3p; hsa-miR-17-5p; hsa-miR-18a-5p; hsa-miR-10a-5p; hsa-miR-15b-5p; hsa-miR-148b-3p; hsa-miR-149-5p; hsa-miR-663a; hsa-miR-26b-3p; hsa-miR-92a-3p; hsa-miR-615-3p; hsa-miR-204-5p; hsa-miR-1226-3p; hsa-miR-331-3p; hsa-miR-197-3p; hsa-miR-346; hsa-miR-30c-5p; hsa-miR-1296-5p; hsa-miR-885-5p
Pathways (miRWalk)	P00029 Huntington disease	enriched	0.010216	61	hsa-miR-29c-3p; hsa-let-7c-5p; hsa-miR-155-5p; hsa-let-7a-5p; hsa-miR-324-3p; hsa-let-7f-5p; hsa-miR-193a-5p; hsa-miR-30d-5p; hsa-miR-1-3p; hsa-miR-877-5p; hsa-miR-26b-5p; hsa-miR-320a; hsa-miR-103a-3p; hsa-miR-1227-3p; hsa-miR-106b-5p; hsa-miR-451a; hsa-miR-184; hsa-miR-1295a; hsa-miR-142-3p; hsa-miR-425-3p; hsa-miR-107; hsa-miR-16-5p; hsa-miR-20a-5p; hsa-miR-24-3p; hsa-miR-92b-3p; hsa-miR-29b-3p; hsa-miR-365a-3p; hsa-miR-181a-5p; hsa-miR-181d-5p; hsa-miR-93-5p; hsa-miR-181b-5p; hsa-miR-124-3p; hsa-miR-21-5p; hsa-miR-29a-3p; hsa-miR-26a-5p; hsa-miR-99b-3p; hsa-miR-1229-3p; hsa-miR-15a-5p; hsa-miR-148a-3p; hsa-miR-320c; hsa-miR-19b-3p; hsa-miR-25-3p; hsa-miR-766-3p; hsa-miR-17-5p; hsa-miR-10a-5p; hsa-miR-15b-5p; hsa-miR-148b-3p; hsa-miR-149-5p; hsa-miR-504-5p; hsa-miR-92a-3p; hsa-miR-615-3p; hsa-miR-505-3p; hsa-miR-30b-5p; hsa-miR-130b-5p; hsa-miR-1226-3p; hsa-miR-331-3p; hsa-miR-197-3p; hsa-miR-30c-5p; hsa-miR-605-5p; hsa-miR-296-5p; hsa-miR-1296-5p

Diseases (miRWalk)	Lung Neoplasms	enriched	0.0102659	22	hsa-miR-29c-3p; hsa-let-7a-5p; hsa-let-7f-5p; hsa-miR-1-3p; hsa-let-7g-5p; hsa-miR-451a; hsa-miR-107; hsa-miR-16-5p; hsa-miR-20a-5p; hsa-miR-29b-3p; hsa-miR-93-5p; hsa-miR-21-5p; hsa-miR-29a-3p; hsa-miR-134-5p; hsa-miR-19a-3p; hsa-miR-188-5p; hsa-miR-15a-5p; hsa-miR-449c-5p; hsa-let-7b-3p; hsa-let-7e-3p; hsa-miR-197-3p; hsa-miR-30c-5p
Gene Ontology (miRWalk)	GO0007249 i kappab kinase nf kappab cascade	enriched	0.0113644	35	hsa-miR-29c-3p; hsa-let-7c-5p; hsa-miR-155-5p; hsa-let-7a-5p; hsa-miR-324-3p; hsa-let-7f-5p; hsa-miR-30d-5p; hsa-miR-1-3p; hsa-miR-877-5p; hsa-miR-26b-5p; hsa-miR-320a; hsa-miR-103a-3p; hsa-let-7i-5p; hsa-miR-760; hsa-miR-1287-5p; hsa-miR-146a-5p; hsa-miR-16-5p; hsa-miR-24-3p; hsa-miR-29b-3p; hsa-miR-181a-5p; hsa-miR-93-5p; hsa-miR-181b-5p; hsa-miR-124-3p; hsa-miR-21-5p; hsa-miR-223-3p; hsa-miR-26a-5p; hsa-miR-15a-5p; hsa-miR-17-5p; hsa-miR-18a-5p; hsa-miR-10a-5p; hsa-miR-15b-5p; hsa-miR-615-3p; hsa-miR-30b-5p; hsa-miR-346; hsa-miR-30c-5p
Organs (miRWalk)	Bodily Secretions	enriched	0.012673	19	hsa-let-7f-5p; hsa-let-7g-5p; hsa-let-7i-5p; hsa-miR-184; hsa-miR-146a-5p; hsa-miR-107; hsa-miR-16-5p; hsa-miR-20a-5p; hsa-miR-29b-3p; hsa-miR-181a-5p; hsa-miR-124-3p; hsa-miR-21-5p; hsa-miR-29a-3p; hsa-miR-26a-5p; hsa-miR-19a-3p; hsa-miR-15a-5p; hsa-miR-19b-3p; hsa-miR-17-5p; hsa-miR-346
Organs (miRWalk)	Liver	enriched	0.012673	23	hsa-let-7c-5p; hsa-miR-155-5p; hsa-miR-1-3p; hsa-miR-26b-5p; hsa-miR-320a; hsa-miR-103a-3p; hsa-miR-107; hsa-miR-422a; hsa-miR-29b-3p; hsa-miR-181a-5p; hsa-miR-181d-5p; hsa-miR-181b-5p; hsa-miR-124-3p; hsa-miR-21-5p; hsa-miR-223-3p; hsa-miR-29a-3p; hsa-miR-26a-5p; hsa-miR-15a-5p; hsa-miR-18a-5p; hsa-miR-758-3p; hsa-miR-483-3p; hsa-miR-631; hsa-miR-296-5p
Organs (miRWalk)	Lung	enriched	0.012673	30	hsa-miR-29c-3p; hsa-let-7c-5p; hsa-miR-155-5p; hsa-let-7a-5p; hsa-let-7f-5p; hsa-miR-1-3p; hsa-let-7g-5p; hsa-let-7i-5p; hsa-miR-451a; hsa-miR-107; hsa-miR-16-5p; hsa-miR-20a-5p; hsa-miR-29b-3p; hsa-miR-93-5p; hsa-miR-21-5p; hsa-miR-223-3p; hsa-miR-29a-3p; hsa-miR-26a-5p; hsa-miR-134-5p; hsa-miR-19a-3p; hsa-miR-188-5p; hsa-miR-15a-5p; hsa-miR-449c-5p; hsa-let-7b-3p; hsa-miR-574-5p; hsa-miR-483-3p; hsa-miR-204-5p; hsa-let-7e-3p; hsa-miR-197-3p; hsa-miR-30c-5p
Organs (miRWalk)	Serum	enriched	0.012673	8	hsa-miR-29c-3p; hsa-miR-1-3p; hsa-miR-422a; hsa-miR-29b-3p; hsa-miR-181a-5p; hsa-miR-21-5p; hsa-miR-29a-3p; hsa-miR-26a-5p
Pathways (miRWalk)	hsa00982 Drug metabolism cytochrome P450	enriched	0.0127199	15	hsa-miR-155-5p; hsa-miR-1-3p; hsa-miR-26b-5p; hsa-miR-320a; hsa-miR-103a-3p; hsa-miR-106b-5p; hsa-miR-142-3p; hsa-miR-107; hsa-miR-16-5p; hsa-miR-92b-3p; hsa-miR-181a-5p; hsa-miR-124-3p; hsa-miR-148b-3p; hsa-miR-92a-3p; hsa-miR-197-3p
Gene Ontology (miRWalk)	GO0006513 protein monoubiquitination	enriched	0.0129657	27	hsa-miR-29c-3p; hsa-let-7c-5p; hsa-miR-155-5p; hsa-let-7a-5p; hsa-miR-324-3p; hsa-miR-877-5p; hsa-miR-26b-5p; hsa-miR-320a; hsa-miR-103a-3p; hsa-miR-106b-5p; hsa-miR-142-3p; hsa-miR-20a-5p; hsa-miR-24-3p; hsa-miR-365a-3p; hsa-miR-181a-5p; hsa-miR-181d-5p; hsa-miR-93-5p; hsa-miR-181b-5p; hsa-miR-124-3p; hsa-miR-21-5p; hsa-miR-29a-3p; hsa-miR-17-5p; hsa-miR-10a-5p; hsa-miR-148b-3p; hsa-miR-149-5p; hsa-miR-92a-3p; hsa-miR-331-3p

Pathways (miRWalk)	P00034 Integrin signalling pathway	enriched	0.0132117	62	hsa-miR-29c-3p; hsa-let-7c-5p; hsa-miR-155-5p; hsa-miR-193b-5p; hsa-let-7a-5p; hsa-miR-324-3p; hsa-miR-1-3p; hsa-miR-877-5p; hsa-miR-26b-5p; hsa-miR-320a; hsa-let-7g-5p; hsa-miR-103a-3p; hsa-miR-1227-3p; hsa-miR-199b-5p; hsa-miR-106b-5p; hsa-let-7i-5p; hsa-miR-760; hsa-miR-146a-5p; hsa-miR-1295a; hsa-miR-142-3p; hsa-miR-107; hsa-miR-16-5p; hsa-miR-20a-5p; hsa-miR-24-3p; hsa-miR-92b-3p; hsa-miR-29b-3p; hsa-miR-181a-5p; hsa-miR-483-5p; hsa-miR-181d-5p; hsa-miR-93-5p; hsa-miR-124-3p; hsa-miR-21-5p; hsa-miR-223-3p; hsa-miR-29a-3p; hsa-miR-26a-5p; hsa-miR-99b-3p; hsa-miR-1229-3p; hsa-miR-15a-5p; hsa-miR-148a-3p; hsa-miR-320c; hsa-miR-19b-3p; hsa-miR-25-3p; hsa-miR-17-5p; hsa-miR-10a-5p; hsa-miR-129-1-3p; hsa-miR-15b-5p; hsa-miR-148b-3p; hsa-miR-149-5p; hsa-miR-23a-3p; hsa-miR-92a-3p; hsa-miR-615-3p; hsa-miR-505-3p; hsa-miR-1260b; hsa-miR-30b-5p; hsa-miR-130b-5p; hsa-miR-204-5p; hsa-miR-337-3p; hsa-miR-1226-3p; hsa-miR-331-3p; hsa-miR-197-3p; hsa-miR-346; hsa-miR-30c-5p
Pathways (miRWalk)	P00054 Toll receptor signaling pathway	enriched	0.0132117	38	hsa-miR-29c-3p; hsa-let-7c-5p; hsa-miR-155-5p; hsa-let-7a-5p; hsa-miR-30d-5p; hsa-miR-1-3p; hsa-miR-877-5p; hsa-miR-26b-5p; hsa-miR-320a; hsa-miR-103a-3p; hsa-miR-106b-5p; hsa-let-7i-5p; hsa-miR-146a-5p; hsa-miR-16-5p; hsa-miR-20a-5p; hsa-miR-24-3p; hsa-miR-181a-5p; hsa-miR-483-5p; hsa-miR-93-5p; hsa-miR-124-3p; hsa-miR-21-5p; hsa-miR-223-3p; hsa-miR-29a-3p; hsa-miR-26a-5p; hsa-miR-19a-3p; hsa-miR-15a-5p; hsa-miR-186-3p; hsa-miR-19b-3p; hsa-miR-17-5p; hsa-miR-10a-5p; hsa-miR-149-5p; hsa-miR-92a-3p; hsa-miR-105-5p; hsa-miR-1226-3p; hsa-miR-346; hsa-miR-30c-5p; hsa-miR-296-5p; hsa-miR-328-3p
Pathways (miRWalk)	WP205 IL 7 signaling pathway	enriched	0.0132117	39	hsa-let-7c-5p; hsa-miR-155-5p; hsa-let-7a-5p; hsa-miR-324-3p; hsa-let-7f-5p; hsa-miR-1-3p; hsa-miR-26b-5p; hsa-miR-320a; hsa-let-7g-5p; hsa-miR-106b-5p; hsa-miR-451a; hsa-miR-146a-5p; hsa-miR-16-5p; hsa-miR-20a-5p; hsa-miR-24-3p; hsa-miR-92b-3p; hsa-miR-365a-3p; hsa-miR-181a-5p; hsa-miR-483-5p; hsa-miR-93-5p; hsa-miR-124-3p; hsa-miR-21-5p; hsa-miR-29a-3p; hsa-miR-26a-5p; hsa-miR-19a-3p; hsa-miR-1229-3p; hsa-miR-15a-5p; hsa-miR-449c-5p; hsa-miR-20b-5p; hsa-miR-766-3p; hsa-miR-17-5p; hsa-miR-15b-5p; hsa-miR-92a-3p; hsa-miR-615-3p; hsa-miR-1260b; hsa-miR-337-3p; hsa-miR-34b-3p; hsa-miR-30c-5p; hsa-miR-296-5p
Pathways (miRWalk)	hsa00670 One carbon pool by folate	enriched	0.0132117	18	hsa-miR-29c-3p; hsa-miR-155-5p; hsa-let-7a-5p; hsa-miR-30d-5p; hsa-miR-1-3p; hsa-miR-877-5p; hsa-miR-26b-5p; hsa-miR-320a; hsa-miR-103a-3p; hsa-miR-106b-5p; hsa-miR-16-5p; hsa-miR-24-3p; hsa-miR-124-3p; hsa-miR-940; hsa-miR-10a-5p; hsa-miR-15b-5p; hsa-miR-92a-3p; hsa-miR-615-3p
Pathways (miRWalk)	hsa05223 Non small cell lung cancer	enriched	0.0132117	59	hsa-miR-29c-3p; hsa-let-7c-5p; hsa-miR-155-5p; hsa-let-7a-5p; hsa-let-7f-5p; hsa-miR-30d-5p; hsa-miR-1-3p; hsa-miR-26b-5p; hsa-miR-320a; hsa-let-7g-5p; hsa-miR-103a-3p; hsa-miR-199b-5p; hsa-miR-106b-5p; hsa-miR-451a; hsa-miR-184; hsa-miR-146a-5p; hsa-miR-548d-3p; hsa-miR-107; hsa-miR-16-5p; hsa-miR-20a-5p; hsa-miR-24-3p; hsa-miR-92b-3p; hsa-miR-29b-3p; hsa-miR-365a-3p; hsa-miR-181a-5p; hsa-miR-483-5p; hsa-miR-181d-5p; hsa-miR-93-5p; hsa-miR-181b-5p; hsa-miR-124-3p; hsa-miR-21-5p; hsa-miR-223-3p; hsa-miR-29a-3p; hsa-miR-26a-5p; hsa-miR-19a-3p; hsa-miR-1914-3p; hsa-miR-15a-5p; hsa-miR-19b-3p; hsa-miR-25-3p; hsa-miR-766-3p; hsa-miR-17-5p; hsa-miR-10a-5p; hsa-miR-15b-5p; hsa-miR-148b-3p; hsa-miR-149-5p; hsa-miR-23a-3p; hsa-miR-504-5p; hsa-miR-92a-3p; hsa-miR-615-3p; hsa-miR-505-3p; hsa-miR-331-3p; hsa-miR-197-3p; hsa-miR-346; hsa-miR-744-3p; hsa-miR-34b-3p; hsa-miR-30c-5p; hsa-miR-605-5p; hsa-miR-296-5p; hsa-miR-1296-5p

Pathways (miRWalk)	WP28 Selenium Metabolism and Selenoproteins	enriched	0.0132306	26	hsa-miR-29c-3p; hsa-miR-155-5p; hsa-let-7a-5p; hsa-miR-1-3p; hsa-miR-26b-5p; hsa-miR-320a; hsa-miR-106b-5p; hsa-miR-146a-5p; hsa-miR-16-5p; hsa-miR-20a-5p; hsa-miR-92b-3p; hsa-miR-29b-3p; hsa-miR-671-5p; hsa-miR-181a-5p; hsa-miR-93-5p; hsa-miR-124-3p; hsa-miR-21-5p; hsa-miR-223-3p; hsa-miR-15a-5p; hsa-miR-149-5p; hsa-miR-504-5p; hsa-miR-92a-3p; hsa-miR-615-3p; hsa-miR-1260b; hsa-miR-331-3p; hsa-miR-346
Pathways (miRWalk)	WP404 Nucleotide Metabolism	enriched	0.0132306	26	hsa-miR-29c-3p; hsa-miR-155-5p; hsa-let-7a-5p; hsa-miR-30d-5p; hsa-miR-1-3p; hsa-miR-26b-5p; hsa-miR-320a; hsa-let-7g-5p; hsa-let-7i-5p; hsa-miR-142-3p; hsa-miR-107; hsa-miR-16-5p; hsa-miR-20a-5p; hsa-miR-24-3p; hsa-miR-29b-3p; hsa-miR-124-3p; hsa-miR-29a-3p; hsa-miR-26a-5p; hsa-miR-1229-3p; hsa-miR-19b-3p; hsa-miR-10a-5p; hsa-miR-15b-5p; hsa-miR-92a-3p; hsa-miR-615-3p; hsa-miR-197-3p; hsa-miR-30c-5p
Pathways (miRWalk)	P04393 Ras Pathway	enriched	0.0144428	48	hsa-miR-29c-3p; hsa-let-7c-5p; hsa-miR-155-5p; hsa-let-7a-5p; hsa-miR-324-3p; hsa-miR-30d-5p; hsa-miR-1-3p; hsa-miR-877-5p; hsa-miR-26b-5p; hsa-miR-320a; hsa-let-7g-5p; hsa-miR-106b-5p; hsa-miR-451a; hsa-miR-146a-5p; hsa-miR-142-3p; hsa-miR-16-5p; hsa-miR-20a-5p; hsa-miR-24-3p; hsa-miR-92b-3p; hsa-miR-29b-3p; hsa-miR-181a-5p; hsa-miR-483-5p; hsa-miR-181d-5p; hsa-miR-93-5p; hsa-miR-124-3p; hsa-miR-21-5p; hsa-miR-223-3p; hsa-miR-29a-3p; hsa-miR-26a-5p; hsa-miR-1229-3p; hsa-miR-15a-5p; hsa-miR-20b-5p; hsa-miR-148a-3p; hsa-miR-320c; hsa-miR-19b-3p; hsa-miR-766-3p; hsa-miR-17-5p; hsa-miR-10a-5p; hsa-miR-15b-5p; hsa-miR-148b-3p; hsa-miR-149-5p; hsa-miR-92a-3p; hsa-miR-615-3p; hsa-miR-30b-5p; hsa-miR-337-3p; hsa-miR-331-3p; hsa-miR-30c-5p; hsa-miR-1296-5p
Pathways (miRWalk)	WP1991 SRF and miRs in Smooth Muscle Differentiation and Proliferation	enriched	0.0144428	26	hsa-let-7c-5p; hsa-miR-155-5p; hsa-let-7a-5p; hsa-miR-324-3p; hsa-let-7f-5p; hsa-miR-1-3p; hsa-miR-877-5p; hsa-miR-26b-5p; hsa-miR-320a; hsa-miR-103a-3p; hsa-miR-106b-5p; hsa-miR-107; hsa-miR-16-5p; hsa-miR-20a-5p; hsa-miR-124-3p; hsa-miR-21-5p; hsa-miR-223-3p; hsa-miR-26a-5p; hsa-miR-15a-5p; hsa-miR-19b-3p; hsa-miR-25-3p; hsa-miR-17-5p; hsa-miR-92a-3p; hsa-miR-615-3p; hsa-miR-30b-5p; hsa-miR-331-3p
Pathways (miRWalk)	WP2037 Prolactin Signaling Pathway	enriched	0.0144428	63	hsa-miR-29c-3p; hsa-let-7c-5p; hsa-miR-155-5p; hsa-let-7a-5p; hsa-miR-324-3p; hsa-let-7f-5p; hsa-miR-30d-5p; hsa-miR-1-3p; hsa-miR-877-5p; hsa-miR-26b-5p; hsa-miR-320a; hsa-let-7g-5p; hsa-miR-103a-3p; hsa-miR-199b-5p; hsa-miR-106b-5p; hsa-let-7i-5p; hsa-miR-760; hsa-miR-451a; hsa-miR-146a-5p; hsa-miR-548d-3p; hsa-miR-142-3p; hsa-miR-16-5p; hsa-miR-20a-5p; hsa-miR-24-3p; hsa-miR-92b-3p; hsa-miR-29b-3p; hsa-miR-181a-5p; hsa-miR-483-5p; hsa-miR-181d-5p; hsa-miR-93-5p; hsa-miR-181b-5p; hsa-miR-124-3p; hsa-miR-21-5p; hsa-miR-29a-3p; hsa-miR-26a-5p; hsa-miR-19a-3p; hsa-miR-1229-3p; hsa-miR-15a-5p; hsa-miR-449c-5p; hsa-miR-20b-5p; hsa-miR-148a-3p; hsa-miR-19b-3p; hsa-miR-25-3p; hsa-miR-766-3p; hsa-miR-17-5p; hsa-miR-18a-5p; hsa-miR-10a-5p; hsa-miR-15b-5p; hsa-miR-148b-3p; hsa-miR-149-5p; hsa-miR-23a-3p; hsa-miR-92a-3p; hsa-miR-615-3p; hsa-miR-1260b; hsa-miR-30b-5p; hsa-miR-130b-5p; hsa-miR-204-5p; hsa-miR-337-3p; hsa-miR-1226-3p; hsa-miR-331-3p; hsa-miR-197-3p; hsa-miR-34b-3p; hsa-miR-30c-5p

Pathways (miRWalk)	WP231 TNF alpha Signaling Pathway	enriched	0.0144428	53	hsa-miR-29c-3p; hsa-let-7c-5p; hsa-miR-155-5p; hsa-let-7a-5p; hsa-miR-324-3p; hsa-let-7f-5p; hsa-miR-30d-5p; hsa-miR-1-3p; hsa-miR-877-5p; hsa-miR-26b-5p; hsa-miR-320a; hsa-let-7g-5p; hsa-miR-103a-3p; hsa-miR-106b-5p; hsa-miR-760; hsa-miR-451a; hsa-miR-146a-5p; hsa-miR-142-3p; hsa-miR-16-5p; hsa-miR-20a-5p; hsa-miR-24-3p; hsa-miR-92b-3p; hsa-miR-365a-3p; hsa-miR-181a-5p; hsa-miR-483-5p; hsa-miR-93-5p; hsa-miR-124-3p; hsa-miR-21-5p; hsa-miR-223-3p; hsa-miR-29a-3p; hsa-miR-26a-5p; hsa-miR-1229-3p; hsa-miR-15a-5p; hsa-miR-940; hsa-miR-25-3p; hsa-miR-766-3p; hsa-miR-17-5p; hsa-miR-10a-5p; hsa-miR-15b-5p; hsa-miR-148b-3p; hsa-miR-149-5p; hsa-miR-504-5p; hsa-miR-92a-3p; hsa-miR-937-3p; hsa-miR-615-3p; hsa-miR-204-5p; hsa-miR-337-3p; hsa-miR-1226-3p; hsa-miR-331-3p; hsa-miR-197-3p; hsa-miR-346; hsa-miR-30c-5p; hsa-miR-296-5p
Pathways (miRWalk)	WP244 Alpha 6 Beta 4 signaling pathway	enriched	0.0144428	35	hsa-miR-29c-3p; hsa-let-7c-5p; hsa-miR-155-5p; hsa-let-7a-5p; hsa-miR-1-3p; hsa-miR-26b-5p; hsa-miR-320a; hsa-miR-199b-5p; hsa-miR-760; hsa-miR-451a; hsa-miR-142-3p; hsa-miR-16-5p; hsa-miR-24-3p; hsa-miR-92b-3p; hsa-miR-181a-5p; hsa-miR-483-5p; hsa-miR-181d-5p; hsa-miR-93-5p; hsa-miR-181b-5p; hsa-miR-124-3p; hsa-miR-21-5p; hsa-miR-29a-3p; hsa-miR-148a-3p; hsa-miR-320c; hsa-miR-766-3p; hsa-miR-10a-5p; hsa-miR-15b-5p; hsa-miR-148b-3p; hsa-miR-23a-3p; hsa-miR-92a-3p; hsa-miR-615-3p; hsa-miR-505-3p; hsa-miR-30b-5p; hsa-miR-130b-5p; hsa-miR-30c-5p
Pathways (miRWalk)	WP286 IL 3 Signaling Pathway	enriched	0.0144428	55	hsa-miR-29c-3p; hsa-let-7c-5p; hsa-miR-155-5p; hsa-miR-193b-5p; hsa-let-7a-5p; hsa-miR-324-3p; hsa-let-7f-5p; hsa-miR-1-3p; hsa-miR-877-5p; hsa-miR-26b-5p; hsa-miR-320a; hsa-let-7g-5p; hsa-miR-103a-3p; hsa-miR-106b-5p; hsa-miR-451a; hsa-miR-146a-5p; hsa-miR-107; hsa-miR-16-5p; hsa-miR-20a-5p; hsa-miR-24-3p; hsa-miR-92b-3p; hsa-miR-29b-3p; hsa-miR-365a-3p; hsa-miR-181a-5p; hsa-miR-483-5p; hsa-miR-181d-5p; hsa-miR-93-5p; hsa-miR-181b-5p; hsa-miR-124-3p; hsa-miR-21-5p; hsa-miR-29a-3p; hsa-miR-99b-3p; hsa-miR-1229-3p; hsa-miR-15a-5p; hsa-miR-20b-5p; hsa-miR-148a-3p; hsa-miR-25-3p; hsa-miR-766-3p; hsa-miR-17-5p; hsa-miR-630; hsa-miR-10a-5p; hsa-miR-15b-5p; hsa-miR-149-5p; hsa-miR-23a-3p; hsa-miR-92a-3p; hsa-miR-615-3p; hsa-miR-30b-5p; hsa-miR-130b-5p; hsa-miR-1910-5p; hsa-miR-204-5p; hsa-miR-337-3p; hsa-miR-331-3p; hsa-miR-30c-5p; hsa-miR-296-5p; hsa-miR-328-3p
Pathways (miRWalk)	WP306 Focal Adhesion	enriched	0.0144428	74	hsa-miR-29c-3p; hsa-let-7c-5p; hsa-miR-155-5p; hsa-let-7a-5p; hsa-miR-584-5p; hsa-miR-324-3p; hsa-let-7f-5p; hsa-miR-1-3p; hsa-miR-877-5p; hsa-miR-26b-5p; hsa-miR-320a; hsa-let-7g-5p; hsa-miR-103a-3p; hsa-miR-1227-3p; hsa-miR-199b-5p; hsa-miR-106b-5p; hsa-miR-760; hsa-miR-451a; hsa-miR-184; hsa-miR-146a-5p; hsa-miR-1295a; hsa-miR-548d-3p; hsa-miR-142-3p; hsa-miR-107; hsa-miR-16-5p; hsa-miR-20a-5p; hsa-miR-24-3p; hsa-miR-92b-3p; hsa-miR-29b-3p; hsa-miR-365a-3p; hsa-miR-181a-5p; hsa-miR-181d-5p; hsa-miR-93-5p; hsa-miR-181b-5p; hsa-miR-124-3p; hsa-miR-21-5p; hsa-miR-223-3p; hsa-miR-29a-3p; hsa-miR-26a-5p; hsa-miR-99b-3p; hsa-miR-19a-3p; hsa-miR-1229-3p; hsa-miR-15a-5p; hsa-miR-148a-3p; hsa-miR-320c; hsa-miR-19b-3p; hsa-miR-494-3p; hsa-miR-25-3p; hsa-miR-766-3p; hsa-miR-17-5p; hsa-miR-18a-5p; hsa-miR-630; hsa-miR-10a-5p; hsa-miR-129-1-3p; hsa-miR-15b-5p; hsa-miR-148b-3p; hsa-miR-149-5p; hsa-miR-23a-3p; hsa-miR-92a-3p; hsa-miR-299-5p; hsa-miR-615-3p; hsa-miR-1260b; hsa-miR-30b-5p; hsa-miR-130b-5p; hsa-miR-1910-5p; hsa-miR-204-5p; hsa-miR-337-3p; hsa-miR-1226-3p; hsa-miR-331-3p; hsa-miR-197-3p; hsa-miR-34b-3p; hsa-miR-30c-5p; hsa-miR-296-5p; hsa-miR-1296-5p

Pathways (miRWalk)	WP455 GPCRs Class A Rhodopsin like	enriched	0.0144428	19	hsa-miR-155-5p; hsa-let-7a-5p; hsa-miR-1-3p; hsa-miR-877-5p; hsa-miR-26b-5p; hsa-miR-320a; hsa-miR-106b-5p; hsa-miR-146a-5p; hsa-miR-142-3p; hsa-miR-107; hsa-miR-16-5p; hsa-miR-181a-5p; hsa-miR-124-3p; hsa-miR-21-5p; hsa-miR-148a-3p; hsa-miR-10a-5p; hsa-miR-148b-3p; hsa-miR-504-5p; hsa-miR-802
Pathways (miRWalk)	WP558 Complement and Coagulation Cascades	enriched	0.0144428	17	hsa-miR-29c-3p; hsa-miR-155-5p; hsa-let-7a-5p; hsa-miR-1-3p; hsa-miR-26b-5p; hsa-miR-142-3p; hsa-miR-16-5p; hsa-miR-29b-3p; hsa-miR-181a-5p; hsa-miR-93-5p; hsa-miR-124-3p; hsa-miR-21-5p; hsa-miR-29a-3p; hsa-miR-19b-3p; hsa-miR-92a-3p; hsa-miR-204-5p; hsa-miR-30c-5p
Pathways (miRWalk)	WP712 Estrogen signaling pathway	enriched	0.0144428	45	hsa-miR-29c-3p; hsa-miR-155-5p; hsa-let-7a-5p; hsa-miR-324-3p; hsa-let-7f-5p; hsa-miR-1-3p; hsa-miR-26b-5p; hsa-miR-320a; hsa-miR-103a-3p; hsa-miR-106b-5p; hsa-miR-18b-5p; hsa-miR-451a; hsa-miR-146a-5p; hsa-miR-16-5p; hsa-miR-20a-5p; hsa-miR-24-3p; hsa-miR-92b-3p; hsa-miR-29b-3p; hsa-miR-365a-3p; hsa-miR-181a-5p; hsa-miR-181d-5p; hsa-miR-93-5p; hsa-miR-181b-5p; hsa-miR-124-3p; hsa-miR-21-5p; hsa-miR-29a-3p; hsa-miR-26a-5p; hsa-miR-19a-3p; hsa-miR-15a-5p; hsa-miR-20b-5p; hsa-miR-148a-3p; hsa-miR-19b-3p; hsa-miR-766-3p; hsa-miR-17-5p; hsa-miR-18a-5p; hsa-miR-630; hsa-miR-15b-5p; hsa-miR-148b-3p; hsa-miR-149-5p; hsa-miR-92a-3p; hsa-miR-615-3p; hsa-miR-204-5p; hsa-miR-331-3p; hsa-miR-30c-5p; hsa-miR-296-5p
Pathways (miRWalk)	WP75 Toll Like Receptor signaling	enriched	0.0144428	52	hsa-miR-29c-3p; hsa-miR-155-5p; hsa-let-7a-5p; hsa-let-7f-5p; hsa-miR-30d-5p; hsa-miR-1-3p; hsa-miR-877-5p; hsa-miR-26b-5p; hsa-miR-320a; hsa-miR-103a-3p; hsa-miR-106b-5p; hsa-let-7i-5p; hsa-miR-451a; hsa-miR-184; hsa-miR-146a-5p; hsa-miR-142-3p; hsa-miR-16-5p; hsa-miR-20a-5p; hsa-miR-24-3p; hsa-miR-92b-3p; hsa-miR-29b-3p; hsa-miR-365a-3p; hsa-miR-181a-5p; hsa-miR-483-5p; hsa-miR-93-5p; hsa-miR-124-3p; hsa-miR-21-5p; hsa-miR-223-3p; hsa-miR-29a-3p; hsa-miR-26a-5p; hsa-miR-19a-3p; hsa-miR-15a-5p; hsa-miR-19b-3p; hsa-miR-486-5p; hsa-miR-766-3p; hsa-miR-17-5p; hsa-miR-10a-5p; hsa-miR-15b-5p; hsa-miR-148b-3p; hsa-miR-149-5p; hsa-miR-92a-3p; hsa-miR-299-5p; hsa-miR-105-5p; hsa-miR-615-3p; hsa-miR-204-5p; hsa-miR-1226-3p; hsa-miR-331-3p; hsa-miR-346; hsa-miR-30c-5p; hsa-miR-296-5p; hsa-miR-328-3p; hsa-miR-1296-5p
Pathways (miRWalk)	hsa00980 Metabolism of xenobiotics by cytochrome P450	enriched	0.0144428	14	hsa-miR-155-5p; hsa-miR-1-3p; hsa-miR-26b-5p; hsa-miR-103a-3p; hsa-miR-106b-5p; hsa-miR-142-3p; hsa-miR-107; hsa-miR-16-5p; hsa-miR-92b-3p; hsa-miR-181a-5p; hsa-miR-124-3p; hsa-miR-148b-3p; hsa-miR-92a-3p; hsa-miR-197-3p
Pathways (miRWalk)	hsa03022 Basal transcription factors	enriched	0.0144428	23	hsa-let-7c-5p; hsa-miR-155-5p; hsa-let-7a-5p; hsa-miR-324-3p; hsa-let-7f-5p; hsa-miR-1-3p; hsa-miR-877-5p; hsa-miR-26b-5p; hsa-miR-106b-5p; hsa-miR-16-5p; hsa-miR-24-3p; hsa-miR-92b-3p; hsa-miR-181a-5p; hsa-miR-93-5p; hsa-miR-181b-5p; hsa-miR-21-5p; hsa-miR-1229-3p; hsa-miR-15a-5p; hsa-miR-19b-3p; hsa-miR-766-3p; hsa-miR-17-5p; hsa-miR-615-3p; hsa-miR-197-3p

Pathways (miRWalk)	hsa04530 Tight junction	enriched	0.0144428	64	hsa-miR-29c-3p; hsa-let-7c-5p; hsa-miR-155-5p; hsa-let-7a-5p; hsa-miR-324-3p; hsa-miR-30d-5p; hsa-miR-1-3p; hsa-miR-877-5p; hsa-miR-26b-5p; hsa-miR-320a; hsa-let-7g-5p; hsa-miR-103a-3p; hsa-miR-1227-3p; hsa-miR-106b-5p; hsa-miR-760; hsa-miR-451a; hsa-miR-184; hsa-miR-1295a; hsa-miR-142-3p; hsa-miR-107; hsa-miR-16-5p; hsa-miR-20a-5p; hsa-miR-24-3p; hsa-miR-92b-3p; hsa-miR-29b-3p; hsa-miR-181a-5p; hsa-miR-181d-5p; hsa-miR-93-5p; hsa-miR-181b-5p; hsa-miR-124-3p; hsa-miR-595; hsa-miR-21-5p; hsa-miR-223-3p; hsa-miR-29a-3p; hsa-miR-26a-5p; hsa-miR-99b-3p; hsa-miR-19a-3p; hsa-miR-1229-3p; hsa-miR-15a-5p; hsa-miR-148a-3p; hsa-miR-940; hsa-miR-320c; hsa-miR-19b-3p; hsa-miR-494-3p; hsa-miR-25-3p; hsa-miR-766-3p; hsa-miR-17-5p; hsa-miR-18a-5p; hsa-miR-10a-5p; hsa-miR-15b-5p; hsa-miR-148b-3p; hsa-miR-149-5p; hsa-miR-23a-3p; hsa-miR-92a-3p; hsa-miR-615-3p; hsa-miR-505-3p; hsa-miR-483-3p; hsa-miR-337-3p; hsa-miR-1226-3p; hsa-miR-331-3p; hsa-miR-197-3p; hsa-miR-346; hsa-miR-34b-3p; hsa-miR-1296-5p
Pathways (miRWalk)	hsa04610 Complement and coagulation cascades	enriched	0.0144428	19	hsa-miR-29c-3p; hsa-miR-155-5p; hsa-let-7a-5p; hsa-miR-1-3p; hsa-miR-26b-5p; hsa-miR-146a-5p; hsa-miR-142-3p; hsa-miR-16-5p; hsa-miR-29b-3p; hsa-miR-181a-5p; hsa-miR-93-5p; hsa-miR-124-3p; hsa-miR-21-5p; hsa-miR-29a-3p; hsa-miR-19b-3p; hsa-miR-10a-5p; hsa-miR-92a-3p; hsa-miR-204-5p; hsa-miR-30c-5p
Pathways (miRWalk)	hsa04620 Toll like receptor signaling pathway	enriched	0.0144428	52	hsa-miR-29c-3p; hsa-miR-155-5p; hsa-let-7a-5p; hsa-let-7f-5p; hsa-miR-30d-5p; hsa-miR-1-3p; hsa-miR-877-5p; hsa-miR-26b-5p; hsa-miR-320a; hsa-miR-103a-3p; hsa-miR-106b-5p; hsa-let-7i-5p; hsa-miR-451a; hsa-miR-184; hsa-miR-146a-5p; hsa-miR-142-3p; hsa-miR-16-5p; hsa-miR-20a-5p; hsa-miR-24-3p; hsa-miR-92b-3p; hsa-miR-29b-3p; hsa-miR-365a-3p; hsa-miR-181a-5p; hsa-miR-483-5p; hsa-miR-93-5p; hsa-miR-124-3p; hsa-miR-21-5p; hsa-miR-223-3p; hsa-miR-29a-3p; hsa-miR-26a-5p; hsa-miR-19a-3p; hsa-miR-15a-5p; hsa-miR-19b-3p; hsa-miR-486-5p; hsa-miR-766-3p; hsa-miR-17-5p; hsa-miR-10a-5p; hsa-miR-15b-5p; hsa-miR-148b-3p; hsa-miR-149-5p; hsa-miR-92a-3p; hsa-miR-299-5p; hsa-miR-105-5p; hsa-miR-615-3p; hsa-miR-204-5p; hsa-miR-1226-3p; hsa-miR-331-3p; hsa-miR-346; hsa-miR-30c-5p; hsa-miR-296-5p; hsa-miR-328-3p; hsa-miR-1296-5p
Pathways (miRWalk)	hsa04912 GnRH signaling pathway	enriched	0.0144428	46	hsa-miR-29c-3p; hsa-let-7c-5p; hsa-miR-155-5p; hsa-let-7a-5p; hsa-miR-324-3p; hsa-miR-1-3p; hsa-miR-877-5p; hsa-miR-26b-5p; hsa-miR-320a; hsa-let-7g-5p; hsa-miR-106b-5p; hsa-miR-451a; hsa-miR-146a-5p; hsa-miR-142-3p; hsa-miR-16-5p; hsa-miR-20a-5p; hsa-miR-24-3p; hsa-miR-92b-3p; hsa-miR-29b-3p; hsa-miR-365a-3p; hsa-miR-181a-5p; hsa-miR-483-5p; hsa-miR-181d-5p; hsa-miR-93-5p; hsa-miR-124-3p; hsa-miR-21-5p; hsa-miR-29a-3p; hsa-miR-26a-5p; hsa-miR-15a-5p; hsa-miR-449c-5p; hsa-miR-148a-3p; hsa-miR-19b-3p; hsa-miR-766-3p; hsa-miR-17-5p; hsa-miR-10a-5p; hsa-miR-15b-5p; hsa-miR-148b-3p; hsa-miR-149-5p; hsa-miR-92a-3p; hsa-miR-615-3p; hsa-miR-505-3p; hsa-miR-1226-3p; hsa-miR-331-3p; hsa-miR-197-3p; hsa-miR-30c-5p; hsa-miR-1296-5p
Pathways (miRWalk)	hsa05130 Pathogenic Escherichia coli infection	enriched	0.0144428	48	hsa-miR-29c-3p; hsa-let-7c-5p; hsa-miR-155-5p; hsa-let-7a-5p; hsa-miR-584-5p; hsa-miR-324-3p; hsa-let-7f-5p; hsa-miR-1-3p; hsa-miR-877-5p; hsa-miR-26b-5p; hsa-miR-320a; hsa-miR-1227-3p; hsa-miR-106b-5p; hsa-let-7i-5p; hsa-miR-760; hsa-miR-146a-5p; hsa-miR-1295a; hsa-miR-142-3p; hsa-miR-107; hsa-miR-16-5p; hsa-miR-20a-5p; hsa-miR-92b-3p; hsa-miR-29b-3p; hsa-miR-365a-3p; hsa-miR-93-5p; hsa-miR-124-3p; hsa-miR-21-5p; hsa-miR-29a-3p; hsa-miR-99b-3p; hsa-miR-1229-3p; hsa-miR-940; hsa-miR-320c; hsa-miR-25-3p; hsa-miR-17-5p; hsa-miR-18a-5p; hsa-miR-10a-5p; hsa-miR-148b-3p; hsa-miR-149-5p; hsa-miR-92a-3p; hsa-miR-615-3p; hsa-miR-505-3p; hsa-miR-1910-5p; hsa-miR-204-5p;

					hsa-miR-1226-3p; hsa-miR-331-3p; hsa-miR-197-3p; hsa-miR-30c-5p; hsa-miR-328-3p
Pathways (miRWalk)	hsa05212 Pancreatic cancer	enriched	0.0144428	64	hsa-miR-29c-3p; hsa-let-7c-5p; hsa-miR-155-5p; hsa-let-7a-5p; hsa-let-7f-5p; hsa-miR-30d-5p; hsa-miR-1-3p; hsa-miR-877-5p; hsa-miR-26b-5p; hsa-miR-320a; hsa-let-7g-5p; hsa-miR-103a-3p; hsa-miR-199b-5p; hsa-miR-106b-5p; hsa-miR-451a; hsa-miR-184; hsa-miR-146a-5p; hsa-miR-548d-3p; hsa-miR-142-3p; hsa-miR-107; hsa-miR-16-5p; hsa-miR-20a-5p; hsa-miR-24-3p; hsa-miR-92b-3p; hsa-miR-29b-3p; hsa-miR-365a-3p; hsa-miR-181a-5p; hsa-miR-483-5p; hsa-miR-93-5p; hsa-miR-181b-5p; hsa-miR-124-3p; hsa-miR-21-5p; hsa-miR-223-3p; hsa-miR-29a-3p; hsa-miR-26a-5p; hsa-miR-134-5p; hsa-miR-19a-3p; hsa-miR-15a-5p; hsa-miR-20b-5p; hsa-miR-19b-3p; hsa-miR-520g-3p; hsa-miR-25-3p; hsa-miR-766-3p; hsa-miR-17-5p; hsa-miR-18a-5p; hsa-miR-10a-5p; hsa-miR-15b-5p; hsa-miR-148b-3p; hsa-miR-149-5p; hsa-miR-504-5p; hsa-miR-92a-3p; hsa-miR-615-3p; hsa-miR-483-3p; hsa-miR-204-5p; hsa-miR-337-3p; hsa-miR-331-3p; hsa-miR-1914-5p; hsa-miR-197-3p; hsa-miR-346; hsa-miR-34b-3p; hsa-miR-30c-5p; hsa-miR-605-5p; hsa-miR-296-5p; hsa-miR-1296-5p
Organs (miRWalk)	Hepatocytes	enriched	0.0160018	10	hsa-let-7c-5p; hsa-let-7g-5p; hsa-miR-103a-3p; hsa-miR-107; hsa-miR-422a; hsa-miR-181a-5p; hsa-miR-181b-5p; hsa-miR-21-5p; hsa-miR-223-3p; hsa-miR-296-5p
Pathways (miRWalk)	WP24 Peptide GPCRs	enriched	0.0166725	14	hsa-miR-155-5p; hsa-miR-1-3p; hsa-miR-877-5p; hsa-miR-26b-5p; hsa-miR-320a; hsa-miR-106b-5p; hsa-miR-146a-5p; hsa-miR-142-3p; hsa-miR-181a-5p; hsa-miR-124-3p; hsa-miR-21-5p; hsa-miR-148a-3p; hsa-miR-148b-3p; hsa-miR-802
Pathways (miRWalk)	WP314 FAS pathway and Stress induction of HSP regulation	enriched	0.0167227	45	hsa-miR-29c-3p; hsa-miR-155-5p; hsa-let-7a-5p; hsa-miR-30d-5p; hsa-miR-1-3p; hsa-miR-877-5p; hsa-miR-26b-5p; hsa-miR-320a; hsa-let-7g-5p; hsa-miR-103a-3p; hsa-miR-106b-5p; hsa-miR-451a; hsa-miR-146a-5p; hsa-miR-16-5p; hsa-miR-20a-5p; hsa-miR-24-3p; hsa-miR-29b-3p; hsa-miR-365a-3p; hsa-miR-181a-5p; hsa-miR-181d-5p; hsa-miR-93-5p; hsa-miR-181b-5p; hsa-miR-124-3p; hsa-miR-21-5p; hsa-miR-223-3p; hsa-miR-29a-3p; hsa-miR-26a-5p; hsa-miR-1229-3p; hsa-miR-15a-5p; hsa-miR-148a-3p; hsa-miR-320c; hsa-miR-17-5p; hsa-miR-18a-5p; hsa-miR-630; hsa-miR-10a-5p; hsa-miR-15b-5p; hsa-miR-149-5p; hsa-miR-504-5p; hsa-miR-92a-3p; hsa-miR-615-3p; hsa-miR-30b-5p; hsa-miR-204-5p; hsa-miR-331-3p; hsa-miR-346; hsa-miR-296-5p
Gene Ontology (miRWalk)	GO0005845 mrna cap binding complex	enriched	0.0173585	16	hsa-let-7c-5p; hsa-miR-155-5p; hsa-let-7a-5p; hsa-miR-30d-5p; hsa-miR-1-3p; hsa-miR-26b-5p; hsa-miR-103a-3p; hsa-miR-107; hsa-miR-16-5p; hsa-miR-24-3p; hsa-miR-671-5p; hsa-miR-181b-5p; hsa-miR-124-3p; hsa-miR-21-5p; hsa-miR-19b-3p; hsa-miR-331-3p
Gene Ontology (miRWalk)	GO0007254 jnk cascade	enriched	0.0173585	41	hsa-let-7c-5p; hsa-miR-155-5p; hsa-miR-193b-5p; hsa-let-7a-5p; hsa-miR-324-3p; hsa-let-7f-5p; hsa-miR-30d-5p; hsa-miR-1-3p; hsa-miR-877-5p; hsa-miR-26b-5p; hsa-miR-320a; hsa-miR-103a-3p; hsa-miR-106b-5p; hsa-let-7i-5p; hsa-miR-760; hsa-miR-1287-5p; hsa-miR-146a-5p; hsa-miR-107; hsa-miR-16-5p; hsa-miR-20a-5p; hsa-miR-24-3p; hsa-miR-181a-5p; hsa-miR-93-5p; hsa-miR-181b-5p; hsa-miR-124-3p; hsa-miR-21-5p; hsa-miR-29a-3p; hsa-miR-26a-5p; hsa-miR-15a-5p; hsa-miR-148a-3p; hsa-miR-17-5p; hsa-miR-18a-5p; hsa-miR-

					10a-5p; hsa-miR-15b-5p; hsa-miR-92a-3p; hsa-miR-615-3p; hsa-miR-30b-5p; hsa-let-7e-3p; hsa-miR-1226-3p; hsa-miR-30c-5p; hsa-miR-328-3p
Gene Ontology (miRWalk)	GO0017017 map kinase tyrosine serine threonine phosphatase activity	enriched	0.0173585	10	hsa-let-7c-5p; hsa-miR-26b-5p; hsa-miR-320a; hsa-miR-760; hsa-miR-16-5p; hsa-miR-29b-3p; hsa-miR-181a-5p; hsa-miR-93-5p; hsa-miR-124-3p; hsa-miR-21-5p
Gene Ontology (miRWalk)	GO0042474 middle ear morphogenesis	enriched	0.0173585	29	hsa-let-7c-5p; hsa-miR-155-5p; hsa-let-7a-5p; hsa-miR-324-3p; hsa-let-7f-5p; hsa-miR-1-3p; hsa-miR-26b-5p; hsa-miR-320a; hsa-let-7g-5p; hsa-miR-103a-3p; hsa-miR-106b-5p; hsa-miR-451a; hsa-miR-142-3p; hsa-miR-107; hsa-miR-20a-5p; hsa-miR-24-3p; hsa-miR-181a-5p; hsa-miR-93-5p; hsa-miR-181b-5p; hsa-miR-124-3p; hsa-miR-21-5p; hsa-miR-26a-5p; hsa-miR-449c-5p; hsa-miR-148a-3p; hsa-miR-17-5p; hsa-miR-10a-5p; hsa-miR-148b-3p; hsa-miR-92a-3p; hsa-miR-34b-3p
Gene Ontology (miRWalk)	GO0051607 defense response to virus	enriched	0.0173585	39	hsa-miR-29c-3p; hsa-let-7c-5p; hsa-miR-155-5p; hsa-let-7a-5p; hsa-miR-1-3p; hsa-miR-26b-5p; hsa-miR-320a; hsa-miR-103a-3p; hsa-miR-106b-5p; hsa-miR-451a; hsa-miR-146a-5p; hsa-miR-142-3p; hsa-miR-107; hsa-miR-16-5p; hsa-miR-20a-5p; hsa-miR-92b-3p; hsa-miR-29b-3p; hsa-miR-154-5p; hsa-miR-365a-3p; hsa-miR-181a-5p; hsa-miR-181d-5p; hsa-miR-93-5p; hsa-miR-181b-5p; hsa-miR-124-3p; hsa-miR-21-5p; hsa-miR-29a-3p; hsa-miR-26a-5p; hsa-miR-15a-5p; hsa-miR-148a-3p; hsa-miR-486-5p; hsa-miR-25-3p; hsa-miR-17-5p; hsa-miR-18a-5p; hsa-miR-630; hsa-miR-10a-5p; hsa-miR-15b-5p; hsa-miR-92a-3p; hsa-miR-204-5p; hsa-miR-296-5p
Gene Ontology (miRWalk)	GO2000774 positive regulation of cellular senescence	enriched	0.0173585	19	hsa-let-7c-5p; hsa-miR-155-5p; hsa-let-7a-5p; hsa-miR-324-3p; hsa-let-7f-5p; hsa-miR-877-5p; hsa-miR-320a; hsa-let-7g-5p; hsa-miR-760; hsa-miR-16-5p; hsa-miR-24-3p; hsa-miR-671-5p; hsa-miR-181a-5p; hsa-miR-181b-5p; hsa-miR-124-3p; hsa-miR-26a-5p; hsa-miR-615-3p; hsa-miR-331-3p; hsa-miR-30c-5p
Pathways (miRWalk)	P00013 Cell cycle	enriched	0.0182412	34	hsa-miR-155-5p; hsa-let-7a-5p; hsa-miR-324-3p; hsa-let-7f-5p; hsa-miR-1-3p; hsa-miR-877-5p; hsa-miR-26b-5p; hsa-miR-320a; hsa-miR-103a-3p; hsa-miR-106b-5p; hsa-miR-760; hsa-miR-142-3p; hsa-miR-107; hsa-miR-16-5p; hsa-miR-20a-5p; hsa-miR-24-3p; hsa-miR-92b-3p; hsa-miR-365a-3p; hsa-miR-93-5p; hsa-miR-124-3p; hsa-miR-26a-5p; hsa-miR-19a-3p; hsa-miR-15a-5p; hsa-miR-940; hsa-miR-19b-3p; hsa-miR-17-5p; hsa-miR-10a-5p; hsa-miR-15b-5p; hsa-miR-92a-3p; hsa-miR-615-3p; hsa-miR-331-3p; hsa-miR-1236-3p; hsa-miR-34b-3p; hsa-miR-296-5p
Pathways (miRWalk)	P00032 Insulin IGF pathway mitogen activated protein kinase kinase MAP kinase cascade	enriched	0.0182412	30	hsa-miR-155-5p; hsa-let-7a-5p; hsa-miR-1-3p; hsa-miR-877-5p; hsa-miR-26b-5p; hsa-miR-320a; hsa-miR-103a-3p; hsa-miR-16-5p; hsa-miR-92b-3p; hsa-miR-29b-3p; hsa-miR-181a-5p; hsa-miR-483-5p; hsa-miR-93-5p; hsa-miR-181b-5p; hsa-miR-124-3p; hsa-miR-21-5p; hsa-miR-223-3p; hsa-miR-29a-3p; hsa-miR-26a-5p; hsa-miR-19a-3p; hsa-miR-148a-3p; hsa-miR-766-3p; hsa-miR-18a-5p; hsa-miR-15b-5p; hsa-miR-92a-3p; hsa-miR-615-3p; hsa-miR-1226-3p; hsa-miR-331-3p; hsa-miR-30c-5p; hsa-miR-1296-5p
Pathways (miRWalk)	P02743 Formyltetrahydroformate biosynthesis	enriched	0.0182412	9	hsa-miR-29c-3p; hsa-miR-155-5p; hsa-miR-30d-5p; hsa-miR-1-3p; hsa-miR-26b-5p; hsa-miR-106b-5p; hsa-miR-16-5p; hsa-miR-10a-5p; hsa-miR-615-3p

Pathways (miRWalk)	hsa04664 Fc epsilon RI signaling pathway	enriched	0.0182412	45	hsa-miR-29c-3p; hsa-let-7c-5p; hsa-miR-155-5p; hsa-let-7a-5p; hsa-let-7f-5p; hsa-miR-1-3p; hsa-miR-877-5p; hsa-miR-26b-5p; hsa-miR-320a; hsa-let-7g-5p; hsa-miR-106b-5p; hsa-let-7i-5p; hsa-miR-451a; hsa-miR-184; hsa-miR-142-3p; hsa-miR-16-5p; hsa-miR-20a-5p; hsa-miR-24-3p; hsa-miR-92b-3p; hsa-miR-181a-5p; hsa-miR-483-5p; hsa-miR-181d-5p; hsa-miR-93-5p; hsa-miR-124-3p; hsa-miR-21-5p; hsa-miR-29a-3p; hsa-miR-99b-3p; hsa-miR-1914-3p; hsa-miR-15a-5p; hsa-miR-449c-5p; hsa-miR-766-3p; hsa-miR-17-5p; hsa-miR-10a-5p; hsa-miR-15b-5p; hsa-miR-148b-3p; hsa-miR-149-5p; hsa-miR-92a-3p; hsa-miR-615-3p; hsa-miR-505-3p; hsa-miR-331-3p; hsa-miR-454-5p; hsa-miR-346; hsa-miR-744-3p; hsa-miR-30c-5p; hsa-miR-1296-5p
Pathways (miRWalk)	P00052 TGF beta signaling pathway	enriched	0.0184553	53	hsa-miR-29c-3p; hsa-let-7c-5p; hsa-miR-155-5p; hsa-let-7a-5p; hsa-miR-324-3p; hsa-miR-30d-5p; hsa-miR-1-3p; hsa-miR-877-5p; hsa-miR-26b-5p; hsa-miR-320a; hsa-let-7g-5p; hsa-miR-103a-3p; hsa-miR-106b-5p; hsa-miR-760; hsa-miR-146a-5p; hsa-miR-142-3p; hsa-miR-16-5p; hsa-miR-20a-5p; hsa-miR-24-3p; hsa-miR-92b-3p; hsa-miR-29b-3p; hsa-miR-181a-5p; hsa-miR-483-5p; hsa-miR-181d-5p; hsa-miR-93-5p; hsa-miR-181b-5p; hsa-miR-124-3p; hsa-miR-21-5p; hsa-miR-29a-3p; hsa-miR-26a-5p; hsa-miR-19a-3p; hsa-miR-15a-5p; hsa-miR-20b-5p; hsa-miR-148a-3p; hsa-miR-19b-3p; hsa-miR-25-3p; hsa-miR-766-3p; hsa-miR-17-5p; hsa-miR-18a-5p; hsa-miR-10a-5p; hsa-miR-15b-5p; hsa-miR-148b-3p; hsa-miR-149-5p; hsa-miR-663a; hsa-miR-92a-3p; hsa-miR-615-3p; hsa-miR-483-3p; hsa-miR-30b-5p; hsa-miR-130b-5p; hsa-miR-204-5p; hsa-miR-1226-3p; hsa-miR-197-3p; hsa-miR-30c-5p
Pathways (miRWalk)	WP2034 Leptin signaling pathway	enriched	0.0184553	64	hsa-miR-29c-3p; hsa-let-7c-5p; hsa-miR-155-5p; hsa-let-7a-5p; hsa-miR-324-3p; hsa-let-7f-5p; hsa-miR-1-3p; hsa-miR-877-5p; hsa-miR-26b-5p; hsa-miR-320a; hsa-let-7g-5p; hsa-miR-103a-3p; hsa-miR-199b-5p; hsa-miR-106b-5p; hsa-miR-18b-5p; hsa-miR-451a; hsa-miR-146a-5p; hsa-miR-548d-3p; hsa-miR-142-3p; hsa-miR-16-5p; hsa-miR-20a-5p; hsa-miR-24-3p; hsa-miR-92b-3p; hsa-miR-29b-3p; hsa-miR-365a-3p; hsa-miR-181a-5p; hsa-miR-483-5p; hsa-miR-181d-5p; hsa-miR-93-5p; hsa-miR-181b-5p; hsa-miR-124-3p; hsa-miR-21-5p; hsa-miR-223-3p; hsa-miR-29a-3p; hsa-miR-26a-5p; hsa-miR-19a-3p; hsa-miR-1229-3p; hsa-miR-1914-3p; hsa-miR-15a-5p; hsa-miR-20b-5p; hsa-miR-148a-3p; hsa-miR-320c; hsa-miR-19b-3p; hsa-miR-766-3p; hsa-miR-17-5p; hsa-miR-18a-5p; hsa-miR-10a-5p; hsa-miR-15b-5p; hsa-miR-148b-3p; hsa-miR-149-5p; hsa-miR-23a-3p; hsa-miR-504-5p; hsa-miR-92a-3p; hsa-miR-615-3p; hsa-miR-1260b; hsa-miR-30b-5p; hsa-miR-130b-5p; hsa-miR-204-5p; hsa-miR-337-3p; hsa-miR-331-3p; hsa-miR-744-3p; hsa-miR-34b-3p; hsa-miR-30c-5p; hsa-miR-296-5p
Pathways (miRWalk)	WP23 B Cell Receptor Signaling Pathway	enriched	0.0184553	63	hsa-miR-29c-3p; hsa-let-7c-5p; hsa-miR-155-5p; hsa-miR-193b-5p; hsa-let-7a-5p; hsa-miR-324-3p; hsa-let-7f-5p; hsa-miR-1-3p; hsa-miR-877-5p; hsa-miR-26b-5p; hsa-miR-320a; hsa-let-7g-5p; hsa-miR-103a-3p; hsa-miR-106b-5p; hsa-miR-451a; hsa-miR-184; hsa-miR-146a-5p; hsa-miR-142-3p; hsa-miR-107; hsa-miR-16-5p; hsa-miR-20a-5p; hsa-miR-24-3p; hsa-miR-92b-3p; hsa-miR-29b-3p; hsa-miR-939-5p; hsa-miR-181a-5p; hsa-miR-181d-5p; hsa-miR-93-5p; hsa-miR-181b-5p; hsa-miR-124-3p; hsa-miR-21-5p; hsa-miR-223-3p; hsa-miR-29a-3p; hsa-miR-26a-5p; hsa-miR-99b-3p; hsa-miR-1229-3p; hsa-miR-1914-3p; hsa-miR-15a-5p; hsa-miR-449c-5p; hsa-miR-940; hsa-miR-19b-3p; hsa-miR-25-3p; hsa-miR-766-3p; hsa-miR-17-5p; hsa-miR-10a-5p; hsa-miR-129-1-3p; hsa-miR-15b-5p; hsa-miR-148b-3p; hsa-miR-149-5p; hsa-miR-23a-3p; hsa-miR-92a-3p; hsa-miR-615-3p; hsa-miR-1260b; hsa-miR-30b-5p; hsa-miR-130b-5p; hsa-miR-204-5p; hsa-miR-1226-3p; hsa-miR-331-3p; hsa-miR-197-3p; hsa-miR-346; hsa-miR-744-3p; hsa-miR-34b-3p; hsa-miR-30c-5p

Pathways (miRWalk)	WP666 Hypothetical Network for Drug Addiction	enriched	0.0184553	18	hsa-miR-1-3p; hsa-miR-877-5p; hsa-miR-26b-5p; hsa-miR-320a; hsa-miR-103a-3p; hsa-miR-16-5p; hsa-miR-92b-3p; hsa-miR-365a-3p; hsa-miR-181a-5p; hsa-miR-483-5p; hsa-miR-181b-5p; hsa-miR-124-3p; hsa-miR-21-5p; hsa-miR-766-3p; hsa-miR-15b-5p; hsa-miR-504-5p; hsa-miR-331-3p; hsa-miR-30c-5p
Pathways (miRWalk)	hsa04062 Chemokine signaling pathway	enriched	0.0184553	64	hsa-miR-29c-3p; hsa-let-7c-5p; hsa-miR-155-5p; hsa-miR-193b-5p; hsa-let-7a-5p; hsa-miR-584-5p; hsa-miR-324-3p; hsa-miR-30d-5p; hsa-miR-1-3p; hsa-miR-877-5p; hsa-miR-26b-5p; hsa-miR-320a; hsa-let-7g-5p; hsa-miR-103a-3p; hsa-miR-106b-5p; hsa-miR-451a; hsa-miR-184; hsa-miR-146a-5p; hsa-miR-142-3p; hsa-miR-107; hsa-miR-16-5p; hsa-miR-20a-5p; hsa-miR-24-3p; hsa-miR-92b-3p; hsa-miR-29b-3p; hsa-miR-365a-3p; hsa-miR-181a-5p; hsa-miR-483-5p; hsa-miR-181d-5p; hsa-miR-93-5p; hsa-miR-124-3p; hsa-miR-21-5p; hsa-miR-223-3p; hsa-miR-29a-3p; hsa-miR-26a-5p; hsa-miR-19a-3p; hsa-miR-1229-3p; hsa-miR-15a-5p; hsa-miR-20b-5p; hsa-miR-148a-3p; hsa-miR-320c; hsa-miR-19b-3p; hsa-miR-25-3p; hsa-miR-766-3p; hsa-miR-17-5p; hsa-miR-10a-5p; hsa-miR-129-1-3p; hsa-miR-15b-5p; hsa-miR-148b-3p; hsa-miR-149-5p; hsa-miR-23a-3p; hsa-miR-92a-3p; hsa-miR-615-3p; hsa-miR-483-3p; hsa-miR-1260b; hsa-miR-30b-5p; hsa-miR-204-5p; hsa-miR-337-3p; hsa-miR-331-3p; hsa-miR-197-3p; hsa-miR-346; hsa-miR-744-3p; hsa-miR-30c-5p; hsa-miR-1296-5p
Pathways (miRWalk)	hsa04650 Natural killer cell mediated cytotoxicity	enriched	0.0184553	54	hsa-let-7c-5p; hsa-miR-155-5p; hsa-let-7a-5p; hsa-miR-324-3p; hsa-miR-30d-5p; hsa-miR-1-3p; hsa-miR-877-5p; hsa-miR-26b-5p; hsa-miR-320a; hsa-let-7g-5p; hsa-miR-103a-3p; hsa-miR-106b-5p; hsa-miR-184; hsa-miR-146a-5p; hsa-miR-142-3p; hsa-miR-16-5p; hsa-miR-20a-5p; hsa-miR-24-3p; hsa-miR-92b-3p; hsa-miR-29b-3p; hsa-miR-939-5p; hsa-miR-181a-5p; hsa-miR-483-5p; hsa-miR-181d-5p; hsa-miR-93-5p; hsa-miR-181b-5p; hsa-miR-124-3p; hsa-miR-21-5p; hsa-miR-29a-3p; hsa-miR-26a-5p; hsa-miR-99b-3p; hsa-miR-1914-3p; hsa-miR-15a-5p; hsa-miR-148a-3p; hsa-miR-940; hsa-miR-766-3p; hsa-miR-17-5p; hsa-miR-15b-5p; hsa-miR-148b-3p; hsa-miR-149-5p; hsa-miR-23a-3p; hsa-miR-504-5p; hsa-miR-92a-3p; hsa-miR-615-3p; hsa-miR-505-3p; hsa-miR-30b-5p; hsa-miR-130b-5p; hsa-miR-331-3p; hsa-miR-197-3p; hsa-miR-346; hsa-miR-744-3p; hsa-miR-34b-3p; hsa-miR-30c-5p; hsa-miR-1296-5p
Pathways (miRWalk)	hsa04660 T cell receptor signaling pathway	enriched	0.0184553	50	hsa-miR-29c-3p; hsa-let-7c-5p; hsa-miR-155-5p; hsa-let-7a-5p; hsa-miR-324-3p; hsa-miR-1-3p; hsa-miR-877-5p; hsa-miR-26b-5p; hsa-miR-320a; hsa-let-7g-5p; hsa-miR-103a-3p; hsa-miR-106b-5p; hsa-miR-451a; hsa-miR-184; hsa-miR-146a-5p; hsa-miR-16-5p; hsa-miR-20a-5p; hsa-miR-24-3p; hsa-miR-92b-3p; hsa-miR-29b-3p; hsa-miR-181a-5p; hsa-miR-483-5p; hsa-miR-181d-5p; hsa-miR-93-5p; hsa-miR-124-3p; hsa-miR-21-5p; hsa-miR-223-3p; hsa-miR-29a-3p; hsa-miR-26a-5p; hsa-miR-1229-3p; hsa-miR-1914-3p; hsa-miR-15a-5p; hsa-miR-320c; hsa-miR-25-3p; hsa-miR-766-3p; hsa-miR-17-5p; hsa-miR-10a-5p; hsa-miR-15b-5p; hsa-miR-148b-3p; hsa-miR-149-5p; hsa-miR-92a-3p; hsa-miR-615-3p; hsa-miR-204-5p; hsa-miR-331-3p; hsa-miR-454-5p; hsa-miR-346; hsa-miR-744-3p; hsa-miR-34b-3p; hsa-miR-30c-5p; hsa-miR-1296-5p

Pathways (miRWalk)	hsa05219 Bladder cancer	enriched	0.0184553	62	hsa-miR-29c-3p; hsa-let-7c-5p; hsa-miR-155-5p; hsa-let-7a-5p; hsa-let-7f-5p; hsa-miR-30d-5p; hsa-miR-1-3p; hsa-miR-26b-5p; hsa-miR-320a; hsa-let-7g-5p; hsa-miR-103a-3p; hsa-miR-199b-5p; hsa-miR-106b-5p; hsa-miR-18b-5p; hsa-miR-451a; hsa-miR-146a-5p; hsa-miR-548d-3p; hsa-miR-107; hsa-miR-16-5p; hsa-miR-20a-5p; hsa-miR-24-3p; hsa-miR-92b-3p; hsa-miR-29b-3p; hsa-miR-365a-3p; hsa-miR-181a-5p; hsa-miR-483-5p; hsa-miR-181d-5p; hsa-miR-93-5p; hsa-miR-181b-5p; hsa-miR-124-3p; hsa-miR-21-5p; hsa-miR-223-3p; hsa-miR-26a-5p; hsa-miR-134-5p; hsa-miR-19a-3p; hsa-miR-1229-3p; hsa-miR-15a-5p; hsa-miR-449c-5p; hsa-miR-520a-3p; hsa-miR-20b-5p; hsa-miR-148a-3p; hsa-miR-19b-3p; hsa-miR-520g-3p; hsa-miR-25-3p; hsa-miR-766-3p; hsa-miR-17-5p; hsa-miR-572; hsa-miR-10a-5p; hsa-miR-15b-5p; hsa-miR-148b-3p; hsa-miR-149-5p; hsa-miR-504-5p; hsa-miR-92a-3p; hsa-miR-299-5p; hsa-miR-615-3p; hsa-miR-1260b; hsa-miR-204-5p; hsa-miR-331-3p; hsa-miR-34b-3p; hsa-miR-30c-5p; hsa-miR-605-5p; hsa-miR-296-5p
Pathways (miRWalk)	P00018 EGF receptor signaling pathway	enriched	0.0185505	58	hsa-miR-29c-3p; hsa-let-7c-5p; hsa-miR-155-5p; hsa-let-7a-5p; hsa-miR-324-3p; hsa-let-7f-5p; hsa-miR-1-3p; hsa-miR-877-5p; hsa-miR-26b-5p; hsa-miR-320a; hsa-let-7g-5p; hsa-miR-103a-3p; hsa-miR-199b-5p; hsa-miR-106b-5p; hsa-let-7i-5p; hsa-miR-451a; hsa-miR-184; hsa-miR-146a-5p; hsa-miR-548d-3p; hsa-miR-142-3p; hsa-miR-16-5p; hsa-miR-20a-5p; hsa-miR-24-3p; hsa-miR-92b-3p; hsa-miR-365a-3p; hsa-miR-181a-5p; hsa-miR-483-5p; hsa-miR-181d-5p; hsa-miR-93-5p; hsa-miR-124-3p; hsa-miR-21-5p; hsa-miR-26a-5p; hsa-miR-19a-3p; hsa-miR-1229-3p; hsa-miR-1914-3p; hsa-miR-15a-5p; hsa-miR-20b-5p; hsa-miR-148a-3p; hsa-miR-19b-3p; hsa-miR-766-3p; hsa-miR-17-5p; hsa-miR-10a-5p; hsa-miR-15b-5p; hsa-miR-148b-3p; hsa-miR-149-5p; hsa-miR-92a-3p; hsa-miR-615-3p; hsa-miR-505-3p; hsa-miR-30b-5p; hsa-miR-1910-5p; hsa-miR-337-3p; hsa-miR-331-3p; hsa-miR-1914-5p; hsa-miR-197-3p; hsa-miR-744-3p; hsa-miR-30c-5p; hsa-miR-328-3p; hsa-miR-1296-5p
Pathways (miRWalk)	WP1544 MicroRNAs in cardiomyocyte hypertrophy	enriched	0.0185505	48	hsa-miR-155-5p; hsa-let-7a-5p; hsa-miR-584-5p; hsa-miR-30d-5p; hsa-miR-1-3p; hsa-miR-877-5p; hsa-miR-26b-5p; hsa-miR-320a; hsa-miR-103a-3p; hsa-miR-760; hsa-miR-451a; hsa-miR-184; hsa-miR-146a-5p; hsa-miR-142-3p; hsa-miR-16-5p; hsa-miR-20a-5p; hsa-miR-24-3p; hsa-miR-92b-3p; hsa-miR-29b-3p; hsa-miR-181a-5p; hsa-miR-483-5p; hsa-miR-93-5p; hsa-miR-181b-5p; hsa-miR-124-3p; hsa-miR-21-5p; hsa-miR-223-3p; hsa-miR-29a-3p; hsa-miR-26a-5p; hsa-miR-15a-5p; hsa-miR-20b-5p; hsa-miR-940; hsa-miR-320c; hsa-miR-766-3p; hsa-miR-17-5p; hsa-miR-10a-5p; hsa-miR-15b-5p; hsa-miR-148b-3p; hsa-miR-149-5p; hsa-miR-92a-3p; hsa-miR-615-3p; hsa-miR-204-5p; hsa-miR-337-3p; hsa-miR-1226-3p; hsa-miR-331-3p; hsa-miR-346; hsa-miR-30c-5p; hsa-miR-296-5p; hsa-miR-1296-5p
Pathways (miRWalk)	WP185 Integrin mediated cell adhesion	enriched	0.0185505	48	hsa-miR-29c-3p; hsa-let-7c-5p; hsa-miR-155-5p; hsa-let-7a-5p; hsa-miR-584-5p; hsa-miR-1-3p; hsa-miR-26b-5p; hsa-miR-320a; hsa-miR-103a-3p; hsa-miR-106b-5p; hsa-miR-760; hsa-miR-451a; hsa-miR-184; hsa-miR-146a-5p; hsa-miR-142-3p; hsa-miR-425-3p; hsa-miR-16-5p; hsa-miR-20a-5p; hsa-miR-24-3p; hsa-miR-92b-3p; hsa-miR-29b-3p; hsa-miR-181a-5p; hsa-miR-181d-5p; hsa-miR-93-5p; hsa-miR-124-3p; hsa-miR-21-5p; hsa-miR-29a-3p; hsa-miR-26a-5p; hsa-miR-1229-3p; hsa-miR-15a-5p; hsa-miR-25-3p; hsa-miR-766-3p; hsa-miR-17-5p; hsa-miR-10a-5p; hsa-miR-129-1-3p; hsa-miR-15b-5p; hsa-miR-148b-3p; hsa-miR-149-5p; hsa-miR-92a-3p; hsa-miR-615-3p; hsa-miR-1260b; hsa-miR-30b-5p; hsa-miR-130b-5p; hsa-miR-204-5p; hsa-miR-337-3p; hsa-miR-331-3p; hsa-miR-30c-5p; hsa-miR-1296-5p

Pathways (miRWalk)	WP1984 Integrated Breast Cancer Pathway	enriched	0.0185505	58	hsa-miR-29c-3p; hsa-miR-155-5p; hsa-let-7a-5p; hsa-let-7f-5p; hsa-miR-30d-5p; hsa-miR-1-3p; hsa-miR-26b-5p; hsa-miR-320a; hsa-let-7g-5p; hsa-miR-103a-3p; hsa-miR-106b-5p; hsa-miR-18b-5p; hsa-let-7i-5p; hsa-miR-146a-5p; hsa-miR-107; hsa-miR-16-5p; hsa-miR-20a-5p; hsa-miR-24-3p; hsa-miR-92b-3p; hsa-miR-29b-3p; hsa-miR-671-5p; hsa-miR-365a-3p; hsa-miR-181a-5p; hsa-miR-181d-5p; hsa-miR-93-5p; hsa-miR-181b-5p; hsa-miR-124-3p; hsa-miR-21-5p; hsa-miR-223-3p; hsa-miR-29a-3p; hsa-miR-26a-5p; hsa-miR-134-5p; hsa-miR-19a-3p; hsa-miR-1229-3p; hsa-miR-15a-5p; hsa-miR-20b-5p; hsa-miR-148a-3p; hsa-miR-19b-3p; hsa-miR-520g-3p; hsa-miR-25-3p; hsa-miR-766-3p; hsa-miR-17-5p; hsa-miR-18a-5p; hsa-miR-10a-5p; hsa-miR-15b-5p; hsa-miR-148b-3p; hsa-miR-149-5p; hsa-miR-23a-3p; hsa-miR-504-5p; hsa-miR-92a-3p; hsa-miR-615-3p; hsa-miR-130b-5p; hsa-miR-337-3p; hsa-miR-331-3p; hsa-miR-34b-3p; hsa-miR-30c-5p; hsa-miR-296-5p; hsa-miR-1296-5p
Conserved (in at least 5 species) (miRBase)	Conserved	enriched	0.0187356	94	hsa-miR-29c-3p; hsa-miR-22-5p; hsa-let-7c-5p; hsa-miR-155-5p; hsa-miR-193b-5p; hsa-let-7a-5p; hsa-let-7f-5p; hsa-miR-193a-5p; hsa-miR-30d-5p; hsa-miR-1-3p; hsa-miR-365a-5p; hsa-miR-877-5p; hsa-miR-26b-5p; hsa-miR-320a; hsa-let-7g-5p; hsa-miR-103a-3p; hsa-miR-199b-5p; hsa-miR-106b-5p; hsa-miR-18b-5p; hsa-let-7i-5p; hsa-miR-760; hsa-miR-451a; hsa-miR-184; hsa-miR-146a-5p; hsa-miR-142-3p; hsa-miR-107; hsa-miR-16-5p; hsa-miR-20a-5p; hsa-miR-24-3p; hsa-miR-92b-3p; hsa-miR-29b-3p; hsa-miR-154-5p; hsa-miR-671-5p; hsa-miR-365a-3p; hsa-miR-599; hsa-miR-181a-5p; hsa-miR-181d-5p; hsa-miR-93-5p; hsa-miR-181b-5p; hsa-miR-124-3p; hsa-miR-21-5p; hsa-miR-223-3p; hsa-miR-29a-3p; hsa-miR-26a-5p; hsa-miR-134-5p; hsa-miR-19a-3p; hsa-miR-188-5p; hsa-miR-15a-5p; hsa-miR-20b-5p; hsa-miR-148a-3p; hsa-miR-19b-3p; hsa-miR-494-3p; hsa-miR-486-5p; hsa-miR-520g-3p; hsa-miR-544a; hsa-miR-25-3p; hsa-miR-17-5p; hsa-miR-18a-5p; hsa-miR-10a-5p; hsa-miR-129-1-3p; hsa-miR-15b-5p; hsa-miR-148b-3p; hsa-miR-149-5p; hsa-miR-490-5p; hsa-let-7f-1-3p; hsa-miR-23a-3p; hsa-miR-504-5p; hsa-miR-129-2-3p; hsa-miR-26b-3p; hsa-miR-1249-3p; hsa-miR-142-5p; hsa-miR-92a-3p; hsa-miR-299-5p; hsa-miR-802; hsa-miR-125b-1-3p; hsa-miR-105-5p; hsa-miR-615-3p; hsa-miR-505-3p; hsa-miR-491-3p; hsa-miR-758-3p; hsa-miR-574-3p; hsa-miR-30b-5p; hsa-miR-204-5p; hsa-let-7e-3p; hsa-miR-331-3p; hsa-miR-197-3p; hsa-miR-346; hsa-miR-30c-5p; hsa-miR-296-5p; hsa-let-7d-3p; hsa-miR-328-3p; hsa-miR-1296-5p; hsa-miR-885-5p; hsa-miR-485-3p
Pathways (miRWalk)	P00016 Cytoskeletal regulation by Rho GTPase	enriched	0.019353	50	hsa-miR-29c-3p; hsa-let-7c-5p; hsa-miR-155-5p; hsa-let-7a-5p; hsa-miR-584-5p; hsa-miR-324-3p; hsa-miR-1-3p; hsa-miR-877-5p; hsa-miR-26b-5p; hsa-miR-320a; hsa-miR-1227-3p; hsa-miR-106b-5p; hsa-miR-146a-5p; hsa-miR-1295a; hsa-miR-142-3p; hsa-miR-107; hsa-miR-16-5p; hsa-miR-20a-5p; hsa-miR-24-3p; hsa-miR-92b-3p; hsa-miR-29b-3p; hsa-miR-365a-3p; hsa-miR-93-5p; hsa-miR-124-3p; hsa-miR-21-5p; hsa-miR-223-3p; hsa-miR-29a-3p; hsa-miR-26a-5p; hsa-miR-99b-3p; hsa-miR-1229-3p; hsa-miR-320c; hsa-miR-19b-3p; hsa-miR-25-3p; hsa-miR-766-3p; hsa-miR-17-5p; hsa-miR-18a-5p; hsa-miR-10a-5p; hsa-miR-148b-3p; hsa-miR-149-5p; hsa-miR-23a-3p; hsa-miR-92a-3p; hsa-miR-615-3p; hsa-miR-505-3p; hsa-miR-1226-3p; hsa-miR-331-3p; hsa-miR-197-3p; hsa-miR-346; hsa-miR-30c-5p; hsa-miR-328-3p; hsa-miR-1296-5p
Pathways (miRWalk)	P00022 General transcription by RNA polymerase I	enriched	0.019353	14	hsa-miR-155-5p; hsa-miR-30d-5p; hsa-miR-1-3p; hsa-miR-760; hsa-miR-16-5p; hsa-miR-24-3p; hsa-miR-365a-3p; hsa-miR-374a-3p; hsa-miR-93-5p; hsa-miR-124-3p; hsa-miR-26a-5p; hsa-miR-10a-5p; hsa-miR-615-3p; hsa-miR-328-3p

Gene Ontology (miRWalk)	GO0000975 regulatory region dna binding	enriched	0.0195967	18	hsa-let-7c-5p; hsa-miR-155-5p; hsa-let-7a-5p; hsa-let-7f-5p; hsa-miR-30d-5p; hsa-miR-26b-5p; hsa-let-7g-5p; hsa-miR-106b-5p; hsa-miR-760; hsa-miR-146a-5p; hsa-miR-16-5p; hsa-miR-20a-5p; hsa-miR-93-5p; hsa-miR-21-5p; hsa-miR-26a-5p; hsa-miR-15a-5p; hsa-miR-17-5p; hsa-miR-92a-3p
Gene Ontology (miRWalk)	GO0001047 core promoter binding	enriched	0.0195967	35	hsa-let-7c-5p; hsa-miR-155-5p; hsa-let-7a-5p; hsa-let-7f-5p; hsa-miR-30d-5p; hsa-miR-26b-5p; hsa-miR-320a; hsa-let-7g-5p; hsa-miR-103a-3p; hsa-miR-106b-5p; hsa-miR-760; hsa-miR-16-5p; hsa-miR-20a-5p; hsa-miR-24-3p; hsa-miR-92b-3p; hsa-miR-671-5p; hsa-miR-181a-5p; hsa-miR-181d-5p; hsa-miR-93-5p; hsa-miR-181b-5p; hsa-miR-124-3p; hsa-miR-21-5p; hsa-miR-223-3p; hsa-miR-26a-5p; hsa-miR-1229-3p; hsa-miR-25-3p; hsa-miR-17-5p; hsa-miR-10a-5p; hsa-miR-149-5p; hsa-miR-92a-3p; hsa-miR-615-3p; hsa-miR-130b-5p; hsa-miR-331-3p; hsa-miR-34b-3p; hsa-miR-30c-5p
Gene Ontology (miRWalk)	GO0001078 rna polymerase ii binding in negative regulation of transcription	enriched	0.0195967	29	hsa-miR-29c-3p; hsa-let-7c-5p; hsa-miR-155-5p; hsa-let-7a-5p; hsa-miR-324-3p; hsa-let-7f-5p; hsa-miR-1-3p; hsa-miR-26b-5p; hsa-miR-320a; hsa-let-7g-5p; hsa-miR-103a-3p; hsa-miR-199b-5p; hsa-miR-760; hsa-miR-16-5p; hsa-miR-20a-5p; hsa-miR-181a-5p; hsa-miR-181b-5p; hsa-miR-124-3p; hsa-miR-21-5p; hsa-miR-223-3p; hsa-miR-26a-5p; hsa-miR-25-3p; hsa-miR-630; hsa-miR-10a-5p; hsa-miR-148b-3p; hsa-miR-23a-3p; hsa-miR-92a-3p; hsa-miR-1260b; hsa-miR-204-5p
Gene Ontology (miRWalk)	GO0003680 at dna binding	enriched	0.0195967	21	hsa-let-7c-5p; hsa-let-7a-5p; hsa-miR-324-3p; hsa-let-7f-5p; hsa-miR-1-3p; hsa-miR-877-5p; hsa-miR-26b-5p; hsa-miR-320a; hsa-let-7g-5p; hsa-miR-1227-3p; hsa-miR-760; hsa-miR-16-5p; hsa-miR-671-5p; hsa-miR-124-3p; hsa-miR-21-5p; hsa-miR-223-3p; hsa-miR-26a-5p; hsa-miR-17-5p; hsa-miR-92a-3p; hsa-miR-331-3p; hsa-miR-30c-5p
Gene Ontology (miRWalk)	GO0004488 methylenetetrahydrofolate dehydrogenase nadpposi activity	enriched	0.0195967	9	hsa-miR-29c-3p; hsa-miR-155-5p; hsa-let-7a-5p; hsa-miR-30d-5p; hsa-miR-1-3p; hsa-miR-877-5p; hsa-miR-106b-5p; hsa-miR-16-5p; hsa-miR-615-3p
Gene Ontology (miRWalk)	GO0004896 cytokine receptor activity	enriched	0.0195967	9	hsa-let-7c-5p; hsa-miR-155-5p; hsa-let-7a-5p; hsa-miR-324-3p; hsa-let-7f-5p; hsa-miR-877-5p; hsa-miR-26b-5p; hsa-miR-148b-3p; hsa-miR-346
Gene Ontology (miRWalk)	GO0007040 lysosome organization	enriched	0.0195967	16	hsa-let-7c-5p; hsa-miR-155-5p; hsa-miR-324-3p; hsa-miR-1-3p; hsa-miR-877-5p; hsa-miR-26b-5p; hsa-miR-760; hsa-miR-16-5p; hsa-miR-20a-5p; hsa-miR-181a-5p; hsa-miR-93-5p; hsa-miR-124-3p; hsa-miR-26a-5p; hsa-miR-19a-3p; hsa-miR-615-3p; hsa-miR-331-3p
Gene Ontology (miRWalk)	GO0008544 epidermis development	enriched	0.0195967	29	hsa-miR-29c-3p; hsa-let-7c-5p; hsa-miR-155-5p; hsa-let-7a-5p; hsa-let-7f-5p; hsa-miR-1-3p; hsa-miR-498; hsa-miR-26b-5p; hsa-miR-320a; hsa-let-7g-5p; hsa-miR-199b-5p; hsa-miR-142-3p; hsa-miR-16-5p; hsa-miR-20a-5p; hsa-miR-24-3p; hsa-miR-671-5p; hsa-miR-181a-5p; hsa-miR-181d-5p; hsa-miR-124-3p; hsa-miR-21-5p; hsa-miR-26a-5p; hsa-miR-18a-5p; hsa-miR-15b-5p; hsa-miR-148b-3p; hsa-miR-149-5p; hsa-miR-615-3p; hsa-miR-1226-3p; hsa-miR-197-3p; hsa-miR-211-5p
Gene Ontology (miRWalk)	GO0010629 negative regulation of gene expression	enriched	0.0195967	48	hsa-miR-29c-3p; hsa-let-7c-5p; hsa-miR-155-5p; hsa-let-7a-5p; hsa-let-7f-5p; hsa-miR-1-3p; hsa-miR-26b-5p; hsa-miR-320a; hsa-miR-103a-3p; hsa-miR-106b-5p; hsa-miR-18b-5p; hsa-miR-760; hsa-miR-451a; hsa-miR-184; hsa-miR-146a-5p; hsa-miR-107; hsa-miR-16-5p; hsa-miR-20a-5p; hsa-miR-24-3p; hsa-miR-92b-3p; hsa-miR-29b-3p; hsa-miR-671-5p; hsa-miR-181a-5p; hsa-miR-93-5p; hsa-miR-181b-5p; hsa-miR-124-3p; hsa-miR-21-5p; hsa-miR-223-3p; hsa-miR-29a-3p; hsa-miR-26a-5p; hsa-miR-19a-3p; hsa-miR-1229-3p; hsa-miR-520a-3p; hsa-miR-20b-5p; hsa-miR-19b-3p; hsa-miR-17-5p; hsa-miR-18a-5p; hsa-miR-572; hsa-miR-15b-5p; hsa-miR-148b-3p; hsa-miR-92a-3p; hsa-miR-

					299-5p; hsa-miR-615-3p; hsa-miR-1260b; hsa-miR-204-5p; hsa-miR-331-3p; hsa-miR-1914-5p; hsa-miR-328-3p
Gene Ontology (miRWalk)	GO0021675 nerve development	enriched	0.0195967	12	hsa-miR-29c-3p; hsa-let-7c-5p; hsa-let-7a-5p; hsa-miR-1-3p; hsa-miR-26b-5p; hsa-miR-103a-3p; hsa-miR-107; hsa-miR-16-5p; hsa-miR-154-5p; hsa-miR-124-3p; hsa-miR-29a-3p; hsa-miR-18a-5p
Gene Ontology (miRWalk)	GO0021889 olfactory bulb interneuron differentiation	enriched	0.0195967	13	hsa-miR-29c-3p; hsa-let-7c-5p; hsa-let-7a-5p; hsa-miR-26b-5p; hsa-miR-320a; hsa-miR-103a-3p; hsa-miR-146a-5p; hsa-miR-107; hsa-miR-16-5p; hsa-miR-154-5p; hsa-miR-29a-3p; hsa-miR-19a-3p; hsa-miR-18a-5p
Gene Ontology (miRWalk)	GO0031054 pre mirna processing	enriched	0.0195967	15	hsa-miR-29c-3p; hsa-let-7c-5p; hsa-let-7a-5p; hsa-miR-30d-5p; hsa-miR-26b-5p; hsa-miR-103a-3p; hsa-miR-107; hsa-miR-16-5p; hsa-miR-154-5p; hsa-miR-671-5p; hsa-miR-124-3p; hsa-miR-21-5p; hsa-miR-29a-3p; hsa-miR-18a-5p; hsa-miR-15b-5p
Gene Ontology (miRWalk)	GO0032049 cardiolipin biosynthetic process	enriched	0.0195967	4	hsa-let-7c-5p; hsa-miR-30d-5p; hsa-miR-1-3p; hsa-miR-26b-5p
Gene Ontology (miRWalk)	GO0032868 response to insulin stimulus	enriched	0.0195967	45	hsa-miR-29c-3p; hsa-let-7c-5p; hsa-miR-155-5p; hsa-let-7a-5p; hsa-miR-30d-5p; hsa-miR-1-3p; hsa-miR-877-5p; hsa-miR-26b-5p; hsa-miR-320a; hsa-miR-103a-3p; hsa-miR-106b-5p; hsa-let-7i-5p; hsa-miR-451a; hsa-miR-184; hsa-miR-146a-5p; hsa-miR-142-3p; hsa-miR-16-5p; hsa-miR-20a-5p; hsa-miR-29b-3p; hsa-miR-671-5p; hsa-miR-365a-3p; hsa-miR-181a-5p; hsa-miR-181d-5p; hsa-miR-93-5p; hsa-miR-181b-5p; hsa-miR-124-3p; hsa-miR-21-5p; hsa-miR-29a-3p; hsa-miR-26a-5p; hsa-miR-1229-3p; hsa-miR-188-5p; hsa-miR-15a-5p; hsa-miR-148a-3p; hsa-miR-19b-3p; hsa-miR-17-5p; hsa-miR-18a-5p; hsa-miR-630; hsa-miR-10a-5p; hsa-miR-15b-5p; hsa-miR-148b-3p; hsa-miR-92a-3p; hsa-miR-204-5p; hsa-miR-331-3p; hsa-miR-30c-5p; hsa-miR-296-5p
Gene Ontology (miRWalk)	GO0043392 negative regulation of dna binding	enriched	0.0195967	25	hsa-miR-29c-3p; hsa-let-7c-5p; hsa-miR-155-5p; hsa-let-7a-5p; hsa-miR-324-3p; hsa-let-7f-5p; hsa-miR-1-3p; hsa-miR-26b-5p; hsa-miR-320a; hsa-let-7g-5p; hsa-miR-103a-3p; hsa-miR-760; hsa-miR-16-5p; hsa-miR-24-3p; hsa-miR-29b-3p; hsa-miR-93-5p; hsa-miR-124-3p; hsa-miR-21-5p; hsa-miR-26a-5p; hsa-miR-15a-5p; hsa-miR-19b-3p; hsa-miR-148b-3p; hsa-miR-149-5p; hsa-miR-92a-3p; hsa-miR-331-3p
Gene Ontology (miRWalk)	GO0043552 positive regulation of phosphatidylinositol 3 kinase activity	enriched	0.0195967	21	hsa-miR-29c-3p; hsa-let-7c-5p; hsa-miR-155-5p; hsa-let-7a-5p; hsa-miR-1-3p; hsa-miR-26b-5p; hsa-miR-320a; hsa-miR-103a-3p; hsa-miR-106b-5p; hsa-miR-146a-5p; hsa-miR-142-3p; hsa-miR-16-5p; hsa-miR-24-3p; hsa-miR-29b-3p; hsa-miR-21-5p; hsa-miR-29a-3p; hsa-miR-19a-3p; hsa-miR-148a-3p; hsa-miR-148b-3p; hsa-miR-92a-3p; hsa-miR-30c-5p
Gene Ontology (miRWalk)	GO0045599 negative regulation of fat cell differentiation	enriched	0.0195967	27	hsa-let-7c-5p; hsa-miR-155-5p; hsa-let-7a-5p; hsa-miR-324-3p; hsa-miR-1-3p; hsa-miR-26b-5p; hsa-miR-320a; hsa-miR-103a-3p; hsa-miR-106b-5p; hsa-miR-107; hsa-miR-16-5p; hsa-miR-20a-5p; hsa-miR-24-3p; hsa-miR-365a-3p; hsa-miR-181a-5p; hsa-miR-181b-5p; hsa-miR-124-3p; hsa-miR-21-5p; hsa-miR-223-3p; hsa-miR-26a-5p; hsa-miR-15a-5p; hsa-miR-940; hsa-miR-320c; hsa-miR-18a-5p; hsa-miR-92a-3p; hsa-miR-331-3p; hsa-miR-34b-3p

Gene Ontology (miRWalk)	GO0045671 negative regulation of osteoclast differentiation	enriched	0.0195967	15	hsa-miR-155-5p; hsa-let-7a-5p; hsa-miR-1-3p; hsa-miR-877-5p; hsa-miR-26b-5p; hsa-miR-320a; hsa-let-7i-5p; hsa-miR-146a-5p; hsa-miR-16-5p; hsa-miR-92b-3p; hsa-miR-181a-5p; hsa-miR-124-3p; hsa-miR-21-5p; hsa-miR-1226-3p; hsa-miR-331-3p
Gene Ontology (miRWalk)	GO0048762 mesenchymal cell differentiation	enriched	0.0195967	15	hsa-let-7c-5p; hsa-miR-155-5p; hsa-let-7a-5p; hsa-let-7f-5p; hsa-miR-1-3p; hsa-miR-26b-5p; hsa-let-7g-5p; hsa-miR-760; hsa-miR-142-3p; hsa-miR-16-5p; hsa-miR-181b-5p; hsa-miR-124-3p; hsa-miR-26a-5p; hsa-miR-10a-5p; hsa-miR-204-5p
Gene Ontology (miRWalk)	GO0050998 nitric oxide synthase binding	enriched	0.0195967	12	hsa-let-7c-5p; hsa-miR-155-5p; hsa-let-7a-5p; hsa-miR-1-3p; hsa-miR-26b-5p; hsa-miR-320a; hsa-miR-103a-3p; hsa-miR-1295a; hsa-miR-16-5p; hsa-miR-124-3p; hsa-miR-21-5p; hsa-miR-92a-3p
Pathways (miRWalk)	P00031 Inflammation mediated by chemokine and cytokine signaling pathway	enriched	0.0196912	69	hsa-miR-29c-3p; hsa-let-7c-5p; hsa-miR-155-5p; hsa-let-7a-5p; hsa-miR-584-5p; hsa-miR-324-3p; hsa-miR-1-3p; hsa-miR-877-5p; hsa-miR-26b-5p; hsa-miR-320a; hsa-let-7g-5p; hsa-miR-103a-3p; hsa-miR-1227-3p; hsa-miR-106b-5p; hsa-miR-451a; hsa-miR-184; hsa-miR-146a-5p; hsa-miR-1295a; hsa-miR-142-3p; hsa-miR-107; hsa-miR-16-5p; hsa-miR-20a-5p; hsa-miR-24-3p; hsa-miR-92b-3p; hsa-miR-29b-3p; hsa-miR-671-5p; hsa-miR-365a-3p; hsa-miR-181a-5p; hsa-miR-483-5p; hsa-miR-181d-5p; hsa-miR-93-5p; hsa-miR-181b-5p; hsa-miR-124-3p; hsa-miR-21-5p; hsa-miR-29a-3p; hsa-miR-26a-5p; hsa-miR-99b-3p; hsa-miR-19a-3p; hsa-miR-1229-3p; hsa-miR-1914-3p; hsa-miR-15a-5p; hsa-miR-449c-5p; hsa-miR-20b-5p; hsa-miR-148a-3p; hsa-miR-320c; hsa-miR-19b-3p; hsa-miR-494-3p; hsa-miR-25-3p; hsa-miR-766-3p; hsa-miR-17-5p; hsa-miR-18a-5p; hsa-miR-10a-5p; hsa-miR-15b-5p; hsa-miR-148b-3p; hsa-miR-149-5p; hsa-miR-663a; hsa-miR-23a-3p; hsa-miR-92a-3p; hsa-miR-615-3p; hsa-miR-505-3p; hsa-miR-30b-5p; hsa-miR-204-5p; hsa-miR-337-3p; hsa-miR-1226-3p; hsa-miR-331-3p; hsa-miR-197-3p; hsa-miR-744-3p; hsa-miR-30c-5p; hsa-miR-1296-5p
Pathways (miRWalk)	WP382 MAPK signaling pathway	enriched	0.0196912	69	hsa-miR-29c-3p; hsa-let-7c-5p; hsa-miR-155-5p; hsa-miR-193b-5p; hsa-let-7a-5p; hsa-miR-324-3p; hsa-let-7f-5p; hsa-miR-30d-5p; hsa-miR-1-3p; hsa-miR-877-5p; hsa-miR-26b-5p; hsa-miR-320a; hsa-let-7g-5p; hsa-miR-103a-3p; hsa-miR-106b-5p; hsa-miR-760; hsa-miR-451a; hsa-miR-184; hsa-miR-146a-5p; hsa-miR-142-3p; hsa-miR-107; hsa-miR-16-5p; hsa-miR-20a-5p; hsa-miR-24-3p; hsa-miR-92b-3p; hsa-miR-29b-3p; hsa-miR-365a-3p; hsa-miR-181a-5p; hsa-miR-483-5p; hsa-miR-93-5p; hsa-miR-181b-5p; hsa-miR-124-3p; hsa-miR-21-5p; hsa-miR-223-3p; hsa-miR-29a-3p; hsa-miR-26a-5p; hsa-miR-99b-3p; hsa-miR-19a-3p; hsa-miR-1229-3p; hsa-miR-15a-5p; hsa-miR-449c-5p; hsa-miR-186-3p; hsa-miR-148a-3p; hsa-miR-19b-3p; hsa-miR-25-3p; hsa-miR-766-3p; hsa-miR-17-5p; hsa-miR-18a-5p; hsa-miR-10a-5p; hsa-miR-129-1-3p; hsa-miR-15b-5p; hsa-miR-148b-3p; hsa-miR-149-5p; hsa-miR-663a; hsa-miR-23a-3p; hsa-miR-504-5p; hsa-miR-92a-3p; hsa-miR-615-3p; hsa-miR-1260b; hsa-miR-30b-5p; hsa-miR-130b-5p; hsa-miR-204-5p; hsa-miR-337-3p; hsa-miR-331-3p; hsa-miR-197-3p; hsa-miR-34b-3p; hsa-miR-30c-5p; hsa-miR-605-5p; hsa-miR-1296-5p
Pathways (miRWalk)	WP408 Oxidative Stress	enriched	0.0196912	30	hsa-miR-155-5p; hsa-let-7a-5p; hsa-let-7f-5p; hsa-miR-1-3p; hsa-miR-877-5p; hsa-miR-26b-5p; hsa-miR-320a; hsa-miR-106b-5p; hsa-miR-146a-5p; hsa-miR-16-5p; hsa-miR-24-3p; hsa-miR-92b-3p; hsa-miR-29b-3p; hsa-miR-671-5p; hsa-miR-181a-5p; hsa-miR-181b-5p; hsa-miR-124-3p; hsa-miR-21-5p; hsa-miR-223-3p; hsa-miR-15a-5p; hsa-miR-15b-5p; hsa-miR-148b-3p; hsa-miR-149-5p; hsa-miR-663a; hsa-miR-504-5p; hsa-miR-142-5p; hsa-miR-92a-3p; hsa-miR-30b-5p; hsa-miR-1226-3p; hsa-miR-197-3p

Gene Ontology (miRWalk)	GO0031996 thioesterase binding	enriched	0.0198127	17	hsa-miR-29c-3p; hsa-miR-155-5p; hsa-miR-1-3p; hsa-miR-26b-5p; hsa-miR-320a; hsa-miR-103a-3p; hsa-miR-106b-5p; hsa-miR-146a-5p; hsa-miR-142-3p; hsa-miR-16-5p; hsa-miR-29b-3p; hsa-miR-181a-5p; hsa-miR-4284; hsa-miR-29a-3p; hsa-miR-92a-3p; hsa-miR-30c-5p; hsa-miR-605-5p
Gene Ontology (miRWalk)	GO0031069 hair follicle morphogenesis	enriched	0.0200775	26	hsa-miR-29c-3p; hsa-let-7c-5p; hsa-miR-155-5p; hsa-let-7a-5p; hsa-miR-1-3p; hsa-miR-26b-5p; hsa-miR-320a; hsa-miR-103a-3p; hsa-miR-106b-5p; hsa-miR-107; hsa-miR-16-5p; hsa-miR-20a-5p; hsa-miR-24-3p; hsa-miR-29b-3p; hsa-miR-154-5p; hsa-miR-93-5p; hsa-miR-124-3p; hsa-miR-21-5p; hsa-miR-29a-3p; hsa-miR-17-5p; hsa-miR-18a-5p; hsa-miR-148b-3p; hsa-miR-92a-3p; hsa-miR-204-5p; hsa-miR-1226-3p; hsa-miR-331-3p
Diseases (published studies about miRNA profiles from peripheral blood)	lung cancer upregulated	depleted	0.0201694	56	hsa-miR-193b-5p; hsa-miR-324-3p; hsa-miR-193a-5p; hsa-miR-30d-5p; hsa-miR-498; hsa-miR-877-5p; hsa-miR-1290; hsa-miR-1227-3p; hsa-miR-199b-5p; hsa-miR-142-3p; hsa-miR-425-3p; hsa-miR-92b-3p; hsa-miR-548i; hsa-miR-765; hsa-miR-154-5p; hsa-miR-939-5p; hsa-miR-365a-3p; hsa-miR-1207-5p; hsa-miR-223-3p; hsa-miR-29a-3p; hsa-miR-1914-3p; hsa-miR-1275; hsa-miR-19b-3p; hsa-miR-135a-3p; hsa-miR-766-3p; hsa-miR-29c-5p; hsa-miR-576-5p; hsa-miR-129-1-3p; hsa-miR-23a-3p; hsa-miR-1281; hsa-miR-26b-3p; hsa-miR-1249-3p; hsa-miR-125b-1-3p; hsa-miR-1225-5p; hsa-miR-1260a; hsa-miR-629-3p; hsa-miR-483-3p; hsa-miR-574-3p; hsa-miR-582-3p; hsa-miR-130b-5p; hsa-miR-204-5p; hsa-miR-181a-2-3p; hsa-miR-337-3p; hsa-let-7e-3p; hsa-miR-1226-3p; hsa-miR-331-3p; hsa-miR-1236-3p; hsa-miR-197-3p; hsa-miR-346; hsa-miR-744-3p; hsa-miR-1909-5p; hsa-miR-605-5p; hsa-let-7d-3p; hsa-miR-328-3p; hsa-miR-885-5p; hsa-miR-668-3p
Diseases (published studies about miRNA profiles from peripheral blood)	pancreatic cancer ducatal downregulated	enriched	0.0201694	23	hsa-miR-371a-5p; hsa-miR-155-5p; hsa-miR-584-5p; hsa-miR-324-3p; hsa-miR-320a; hsa-miR-106b-5p; hsa-miR-320d; hsa-miR-146a-5p; hsa-miR-1295a; hsa-miR-548d-3p; hsa-miR-92b-3p; hsa-miR-422a; hsa-miR-29b-3p; hsa-miR-181a-5p; hsa-miR-181b-5p; hsa-miR-29a-3p; hsa-miR-148a-3p; hsa-miR-320c; hsa-miR-636; hsa-miR-1538; hsa-miR-197-3p; hsa-let-7d-3p; hsa-miR-566
Diseases (published studies about miRNA profiles from peripheral blood)	pancreatitis downregulated	enriched	0.0201694	18	hsa-miR-155-5p; hsa-miR-584-5p; hsa-miR-324-3p; hsa-miR-320a; hsa-miR-520d-3p; hsa-miR-320d; hsa-miR-146a-5p; hsa-miR-92b-3p; hsa-miR-150-3p; hsa-miR-422a; hsa-miR-374a-3p; hsa-miR-181a-5p; hsa-miR-181b-5p; hsa-miR-148a-3p; hsa-miR-320c; hsa-miR-197-3p; hsa-miR-566; hsa-miR-485-3p
Gene Ontology (miRWalk)	GO0000080 g1 phase of mitotic cell cycle	enriched	0.0202217	57	hsa-miR-29c-3p; hsa-let-7c-5p; hsa-miR-155-5p; hsa-let-7a-5p; hsa-miR-324-3p; hsa-let-7f-5p; hsa-miR-1-3p; hsa-miR-877-5p; hsa-miR-26b-5p; hsa-miR-320a; hsa-let-7g-5p; hsa-miR-103a-3p; hsa-miR-1227-3p; hsa-miR-106b-5p; hsa-miR-760; hsa-miR-1287-5p; hsa-miR-146a-5p; hsa-miR-142-3p; hsa-miR-107; hsa-miR-16-5p; hsa-miR-20a-5p; hsa-miR-24-3p; hsa-miR-92b-3p; hsa-miR-29b-3p; hsa-miR-671-5p; hsa-miR-365a-3p; hsa-miR-181a-5p; hsa-miR-93-5p; hsa-miR-181b-5p; hsa-miR-124-3p; hsa-miR-21-5p; hsa-miR-223-3p; hsa-miR-29a-3p; hsa-miR-26a-5p; hsa-miR-19a-3p; hsa-miR-1229-3p; hsa-miR-15a-5p; hsa-miR-520a-3p; hsa-miR-20b-5p; hsa-miR-25-3p; hsa-miR-766-3p; hsa-miR-17-5p; hsa-miR-18a-5p; hsa-miR-572; hsa-miR-10a-5p; hsa-miR-15b-5p; hsa-miR-149-5p; hsa-miR-92a-3p; hsa-miR-299-5p; hsa-miR-615-3p; hsa-miR-1260b; hsa-miR-30b-5p; hsa-miR-331-3p; hsa-miR-197-3p; hsa-miR-34b-3p; hsa-miR-30c-5p; hsa-miR-296-5p

Gene Ontology (miRWalk)	GO0002224 toll like receptor signaling pathway	enriched	0.0202217	53	hsa-miR-29c-3p; hsa-let-7c-5p; hsa-miR-155-5p; hsa-let-7a-5p; hsa-miR-324-3p; hsa-let-7f-5p; hsa-miR-30d-5p; hsa-miR-1-3p; hsa-miR-877-5p; hsa-miR-26b-5p; hsa-miR-320a; hsa-let-7g-5p; hsa-miR-103a-3p; hsa-miR-106b-5p; hsa-let-7i-5p; hsa-miR-760; hsa-miR-1287-5p; hsa-miR-146a-5p; hsa-miR-16-5p; hsa-miR-20a-5p; hsa-miR-24-3p; hsa-miR-92b-3p; hsa-miR-29b-3p; hsa-miR-181a-5p; hsa-miR-483-5p; hsa-miR-93-5p; hsa-miR-181b-5p; hsa-miR-124-3p; hsa-miR-21-5p; hsa-miR-223-3p; hsa-miR-29a-3p; hsa-miR-26a-5p; hsa-miR-19a-3p; hsa-miR-1229-3p; hsa-miR-15a-5p; hsa-miR-148a-3p; hsa-miR-19b-3p; hsa-miR-766-3p; hsa-miR-17-5p; hsa-miR-18a-5p; hsa-miR-10a-5p; hsa-miR-15b-5p; hsa-miR-149-5p; hsa-miR-663a; hsa-miR-92a-3p; hsa-miR-105-5p; hsa-miR-615-3p; hsa-miR-30b-5p; hsa-miR-1226-3p; hsa-miR-346; hsa-miR-30c-5p; hsa-miR-328-3p; hsa-miR-1296-5p
Gene Ontology (miRWalk)	GO0002756 myd88 independent toll like receptor signaling pathway	enriched	0.0202217	51	hsa-miR-29c-3p; hsa-let-7c-5p; hsa-miR-155-5p; hsa-let-7a-5p; hsa-miR-324-3p; hsa-let-7f-5p; hsa-miR-30d-5p; hsa-miR-1-3p; hsa-miR-877-5p; hsa-miR-26b-5p; hsa-miR-320a; hsa-miR-103a-3p; hsa-miR-106b-5p; hsa-miR-760; hsa-miR-1287-5p; hsa-miR-146a-5p; hsa-miR-142-3p; hsa-miR-16-5p; hsa-miR-20a-5p; hsa-miR-24-3p; hsa-miR-92b-3p; hsa-miR-29b-3p; hsa-miR-181a-5p; hsa-miR-483-5p; hsa-miR-93-5p; hsa-miR-181b-5p; hsa-miR-124-3p; hsa-miR-21-5p; hsa-miR-223-3p; hsa-miR-29a-3p; hsa-miR-26a-5p; hsa-miR-19a-3p; hsa-miR-1229-3p; hsa-miR-15a-5p; hsa-miR-148a-3p; hsa-miR-19b-3p; hsa-miR-766-3p; hsa-miR-17-5p; hsa-miR-18a-5p; hsa-miR-10a-5p; hsa-miR-15b-5p; hsa-miR-149-5p; hsa-miR-663a; hsa-miR-92a-3p; hsa-miR-615-3p; hsa-miR-30b-5p; hsa-miR-1226-3p; hsa-miR-30c-5p; hsa-miR-296-5p; hsa-miR-328-3p; hsa-miR-1296-5p
Gene Ontology (miRWalk)	GO0006184 gtp catabolic process	enriched	0.0202217	46	hsa-miR-29c-3p; hsa-let-7c-5p; hsa-miR-155-5p; hsa-let-7a-5p; hsa-miR-324-3p; hsa-miR-30d-5p; hsa-miR-1-3p; hsa-miR-877-5p; hsa-miR-26b-5p; hsa-miR-320a; hsa-let-7g-5p; hsa-miR-103a-3p; hsa-miR-106b-5p; hsa-miR-760; hsa-miR-451a; hsa-miR-146a-5p; hsa-miR-142-3p; hsa-miR-16-5p; hsa-miR-20a-5p; hsa-miR-24-3p; hsa-miR-92b-3p; hsa-miR-29b-3p; hsa-miR-181a-5p; hsa-miR-93-5p; hsa-miR-124-3p; hsa-miR-21-5p; hsa-miR-223-3p; hsa-miR-29a-3p; hsa-miR-26a-5p; hsa-miR-19a-3p; hsa-miR-15a-5p; hsa-miR-148a-3p; hsa-miR-19b-3p; hsa-miR-17-5p; hsa-miR-18a-5p; hsa-miR-10a-5p; hsa-miR-15b-5p; hsa-miR-148b-3p; hsa-miR-149-5p; hsa-miR-23a-3p; hsa-miR-92a-3p; hsa-miR-615-3p; hsa-miR-30b-5p; hsa-miR-331-3p; hsa-miR-197-3p; hsa-miR-30c-5p
Gene Ontology (miRWalk)	GO0034130 toll like receptor 1 signaling pathway	enriched	0.0202217	53	hsa-miR-29c-3p; hsa-let-7c-5p; hsa-miR-155-5p; hsa-let-7a-5p; hsa-miR-324-3p; hsa-let-7f-5p; hsa-miR-30d-5p; hsa-miR-1-3p; hsa-miR-877-5p; hsa-miR-26b-5p; hsa-miR-320a; hsa-let-7g-5p; hsa-miR-103a-3p; hsa-miR-106b-5p; hsa-let-7i-5p; hsa-miR-760; hsa-miR-1287-5p; hsa-miR-146a-5p; hsa-miR-16-5p; hsa-miR-20a-5p; hsa-miR-24-3p; hsa-miR-92b-3p; hsa-miR-29b-3p; hsa-miR-181a-5p; hsa-miR-483-5p; hsa-miR-93-5p; hsa-miR-181b-5p; hsa-miR-124-3p; hsa-miR-21-5p; hsa-miR-223-3p; hsa-miR-29a-3p; hsa-miR-26a-5p; hsa-miR-19a-3p; hsa-miR-1229-3p; hsa-miR-15a-5p; hsa-miR-148a-3p; hsa-miR-19b-3p; hsa-miR-766-3p; hsa-miR-17-5p; hsa-miR-18a-5p; hsa-miR-10a-5p; hsa-miR-15b-5p; hsa-miR-149-5p; hsa-miR-663a; hsa-miR-92a-3p; hsa-miR-105-5p; hsa-miR-615-3p; hsa-miR-30b-5p; hsa-miR-1226-3p; hsa-miR-346; hsa-miR-30c-5p; hsa-miR-328-3p; hsa-miR-1296-5p

Gene Ontology (miRWalk)	GO0034134 toll like receptor 2 signaling pathway	enriched	0.0202217	53	hsa-miR-29c-3p; hsa-let-7c-5p; hsa-miR-155-5p; hsa-let-7a-5p; hsa-miR-324-3p; hsa-let-7f-5p; hsa-miR-30d-5p; hsa-miR-1-3p; hsa-miR-877-5p; hsa-miR-26b-5p; hsa-miR-320a; hsa-let-7g-5p; hsa-miR-103a-3p; hsa-miR-106b-5p; hsa-let-7i-5p; hsa-miR-760; hsa-miR-1287-5p; hsa-miR-146a-5p; hsa-miR-16-5p; hsa-miR-20a-5p; hsa-miR-24-3p; hsa-miR-92b-3p; hsa-miR-29b-3p; hsa-miR-181a-5p; hsa-miR-483-5p; hsa-miR-93-5p; hsa-miR-181b-5p; hsa-miR-124-3p; hsa-miR-21-5p; hsa-miR-223-3p; hsa-miR-29a-3p; hsa-miR-26a-5p; hsa-miR-19a-3p; hsa-miR-1229-3p; hsa-miR-15a-5p; hsa-miR-148a-3p; hsa-miR-19b-3p; hsa-miR-766-3p; hsa-miR-17-5p; hsa-miR-18a-5p; hsa-miR-10a-5p; hsa-miR-15b-5p; hsa-miR-149-5p; hsa-miR-663a; hsa-miR-92a-3p; hsa-miR-105-5p; hsa-miR-615-3p; hsa-miR-30b-5p; hsa-miR-1226-3p; hsa-miR-346; hsa-miR-30c-5p; hsa-miR-328-3p; hsa-miR-1296-5p
Gene Ontology (miRWalk)	GO0034138 toll like receptor 3 signaling pathway	enriched	0.0202217	51	hsa-miR-29c-3p; hsa-let-7c-5p; hsa-miR-155-5p; hsa-let-7a-5p; hsa-miR-324-3p; hsa-let-7f-5p; hsa-miR-30d-5p; hsa-miR-1-3p; hsa-miR-877-5p; hsa-miR-26b-5p; hsa-miR-320a; hsa-miR-103a-3p; hsa-miR-106b-5p; hsa-let-7i-5p; hsa-miR-760; hsa-miR-1287-5p; hsa-miR-146a-5p; hsa-miR-16-5p; hsa-miR-20a-5p; hsa-miR-24-3p; hsa-miR-92b-3p; hsa-miR-29b-3p; hsa-miR-181a-5p; hsa-miR-483-5p; hsa-miR-93-5p; hsa-miR-181b-5p; hsa-miR-124-3p; hsa-miR-21-5p; hsa-miR-223-3p; hsa-miR-29a-3p; hsa-miR-26a-5p; hsa-miR-19a-3p; hsa-miR-1229-3p; hsa-miR-15a-5p; hsa-miR-148a-3p; hsa-miR-19b-3p; hsa-miR-766-3p; hsa-miR-17-5p; hsa-miR-18a-5p; hsa-miR-10a-5p; hsa-miR-15b-5p; hsa-miR-149-5p; hsa-miR-663a; hsa-miR-92a-3p; hsa-miR-615-3p; hsa-miR-30b-5p; hsa-miR-1226-3p; hsa-miR-30c-5p; hsa-miR-296-5p; hsa-miR-328-3p; hsa-miR-1296-5p
Gene Ontology (miRWalk)	GO0034142 toll like receptor 4 signaling pathway	enriched	0.0202217	55	hsa-miR-29c-3p; hsa-let-7c-5p; hsa-miR-155-5p; hsa-let-7a-5p; hsa-miR-324-3p; hsa-let-7f-5p; hsa-miR-30d-5p; hsa-miR-1-3p; hsa-miR-877-5p; hsa-miR-26b-5p; hsa-miR-320a; hsa-let-7g-5p; hsa-miR-103a-3p; hsa-miR-106b-5p; hsa-let-7i-5p; hsa-miR-760; hsa-miR-1287-5p; hsa-miR-146a-5p; hsa-miR-142-3p; hsa-miR-16-5p; hsa-miR-20a-5p; hsa-miR-24-3p; hsa-miR-92b-3p; hsa-miR-29b-3p; hsa-miR-181a-5p; hsa-miR-483-5p; hsa-miR-93-5p; hsa-miR-181b-5p; hsa-miR-124-3p; hsa-miR-21-5p; hsa-miR-223-3p; hsa-miR-29a-3p; hsa-miR-26a-5p; hsa-miR-19a-3p; hsa-miR-1229-3p; hsa-miR-15a-5p; hsa-miR-148a-3p; hsa-miR-19b-3p; hsa-miR-766-3p; hsa-miR-17-5p; hsa-miR-18a-5p; hsa-miR-10a-5p; hsa-miR-15b-5p; hsa-miR-149-5p; hsa-miR-663a; hsa-miR-92a-3p; hsa-miR-105-5p; hsa-miR-615-3p; hsa-miR-30b-5p; hsa-miR-1226-3p; hsa-miR-346; hsa-miR-30c-5p; hsa-miR-296-5p; hsa-miR-328-3p; hsa-miR-1296-5p
Gene Ontology (miRWalk)	GO0035666 trif dependent toll like receptor signaling pathway	enriched	0.0202217	51	hsa-miR-29c-3p; hsa-let-7c-5p; hsa-miR-155-5p; hsa-let-7a-5p; hsa-miR-324-3p; hsa-let-7f-5p; hsa-miR-30d-5p; hsa-miR-1-3p; hsa-miR-877-5p; hsa-miR-26b-5p; hsa-miR-320a; hsa-miR-103a-3p; hsa-miR-106b-5p; hsa-let-7i-5p; hsa-miR-760; hsa-miR-1287-5p; hsa-miR-146a-5p; hsa-miR-16-5p; hsa-miR-20a-5p; hsa-miR-24-3p; hsa-miR-92b-3p; hsa-miR-29b-3p; hsa-miR-181a-5p; hsa-miR-483-5p; hsa-miR-93-5p; hsa-miR-181b-5p; hsa-miR-124-3p; hsa-miR-21-5p; hsa-miR-223-3p; hsa-miR-29a-3p; hsa-miR-26a-5p; hsa-miR-19a-3p; hsa-miR-1229-3p; hsa-miR-15a-5p; hsa-miR-148a-3p; hsa-miR-19b-3p; hsa-miR-766-3p; hsa-miR-17-5p; hsa-miR-18a-5p; hsa-miR-10a-5p; hsa-miR-15b-5p; hsa-miR-149-5p; hsa-miR-663a; hsa-miR-92a-3p; hsa-miR-615-3p; hsa-miR-30b-5p; hsa-miR-1226-3p; hsa-miR-30c-5p; hsa-miR-296-5p; hsa-miR-328-3p; hsa-miR-1296-5p
Gene Ontology (miRWalk)	GO0042742 defense response to bacterium	enriched	0.0202217	22	hsa-miR-155-5p; hsa-let-7a-5p; hsa-miR-324-3p; hsa-miR-1-3p; hsa-miR-26b-5p; hsa-miR-320a; hsa-miR-103a-3p; hsa-let-7i-5p; hsa-miR-146a-5p; hsa-miR-16-5p; hsa-miR-92b-3p; hsa-miR-29b-3p; hsa-miR-93-5p; hsa-miR-124-3p; hsa-

					miR-21-5p; hsa-miR-26a-5p; hsa-miR-99b-3p; hsa-miR-15a-5p; hsa-miR-15b-5p; hsa-miR-92a-3p; hsa-miR-615-3p; hsa-miR-331-3p
Gene Ontology (miRWalk)	GO0060575 intestinal epithelial cell differentiation	enriched	0.0202217	8	hsa-miR-1-3p; hsa-miR-103a-3p; hsa-miR-16-5p; hsa-miR-20a-5p; hsa-miR-92b-3p; hsa-miR-181a-5p; hsa-miR-181b-5p; hsa-miR-124-3p
Pathways (miRWalk)	WP474 Endochondral Ossification	enriched	0.0202775	50	hsa-miR-29c-3p; hsa-let-7c-5p; hsa-miR-155-5p; hsa-let-7a-5p; hsa-miR-324-3p; hsa-miR-30d-5p; hsa-miR-1-3p; hsa-miR-877-5p; hsa-miR-26b-5p; hsa-miR-320a; hsa-miR-103a-3p; hsa-miR-106b-5p; hsa-miR-18b-5p; hsa-miR-451a; hsa-miR-146a-5p; hsa-miR-107; hsa-miR-16-5p; hsa-miR-20a-5p; hsa-miR-24-3p; hsa-miR-92b-3p; hsa-miR-29b-3p; hsa-miR-365a-3p; hsa-miR-181a-5p; hsa-miR-93-5p; hsa-miR-181b-5p; hsa-miR-124-3p; hsa-miR-21-5p; hsa-miR-223-3p; hsa-miR-134-5p; hsa-miR-1229-3p; hsa-miR-15a-5p; hsa-miR-20b-5p; hsa-miR-19b-3p; hsa-miR-520g-3p; hsa-miR-25-3p; hsa-miR-17-5p; hsa-miR-18a-5p; hsa-miR-10a-5p; hsa-miR-15b-5p; hsa-miR-148b-3p; hsa-miR-504-5p; hsa-miR-92a-3p; hsa-miR-299-5p; hsa-miR-615-3p; hsa-miR-30b-5p; hsa-miR-204-5p; hsa-miR-1226-3p; hsa-miR-331-3p; hsa-miR-34b-3p; hsa-miR-30c-5p
Pathways (miRWalk)	hsa04510 Focal adhesion	enriched	0.0202775	83	hsa-miR-29c-3p; hsa-let-7c-5p; hsa-miR-155-5p; hsa-miR-193b-5p; hsa-let-7a-5p; hsa-miR-584-5p; hsa-miR-324-3p; hsa-let-7f-5p; hsa-miR-1-3p; hsa-miR-877-5p; hsa-miR-26b-5p; hsa-miR-320a; hsa-let-7g-5p; hsa-miR-103a-3p; hsa-miR-1227-3p; hsa-miR-199b-5p; hsa-miR-106b-5p; hsa-miR-760; hsa-miR-451a; hsa-miR-184; hsa-miR-146a-5p; hsa-miR-1295a; hsa-miR-548d-3p; hsa-miR-142-3p; hsa-miR-107; hsa-miR-16-5p; hsa-miR-20a-5p; hsa-miR-24-3p; hsa-miR-92b-3p; hsa-miR-29b-3p; hsa-miR-365a-3p; hsa-miR-181a-5p; hsa-miR-483-5p; hsa-miR-181d-5p; hsa-miR-93-5p; hsa-miR-181b-5p; hsa-miR-124-3p; hsa-miR-21-5p; hsa-miR-223-3p; hsa-miR-29a-3p; hsa-miR-26a-5p; hsa-miR-99b-3p; hsa-miR-134-5p; hsa-miR-19a-3p; hsa-miR-1229-3p; hsa-miR-15a-5p; hsa-miR-20b-5p; hsa-miR-148a-3p; hsa-miR-940; hsa-miR-320c; hsa-miR-19b-3p; hsa-miR-494-3p; hsa-miR-520g-3p; hsa-miR-25-3p; hsa-miR-766-3p; hsa-miR-17-5p; hsa-miR-18a-5p; hsa-miR-630; hsa-miR-10a-5p; hsa-miR-129-1-3p; hsa-miR-15b-5p; hsa-miR-148b-3p; hsa-miR-149-5p; hsa-miR-23a-3p; hsa-miR-504-5p; hsa-miR-92a-3p; hsa-miR-299-5p; hsa-miR-615-3p; hsa-miR-505-3p; hsa-miR-1260b; hsa-miR-30b-5p; hsa-miR-130b-5p; hsa-miR-204-5p; hsa-miR-337-3p; hsa-miR-1226-3p; hsa-miR-331-3p; hsa-miR-197-3p; hsa-miR-346; hsa-miR-34b-3p; hsa-miR-30c-5p; hsa-miR-296-5p; hsa-miR-328-3p; hsa-miR-1296-5p
Pathways (miRWalk)	hsa04810 Regulation of actin cytoskeleton	enriched	0.0202775	66	hsa-miR-29c-3p; hsa-let-7c-5p; hsa-miR-155-5p; hsa-miR-193b-5p; hsa-let-7a-5p; hsa-miR-584-5p; hsa-miR-324-3p; hsa-miR-1-3p; hsa-miR-877-5p; hsa-miR-26b-5p; hsa-miR-320a; hsa-let-7g-5p; hsa-miR-103a-3p; hsa-miR-1227-3p; hsa-miR-106b-5p; hsa-miR-18b-5p; hsa-let-7i-5p; hsa-miR-146a-5p; hsa-miR-1295a; hsa-miR-142-3p; hsa-miR-107; hsa-miR-16-5p; hsa-miR-20a-5p; hsa-miR-24-3p; hsa-miR-92b-3p; hsa-miR-29b-3p; hsa-miR-181a-5p; hsa-miR-483-5p; hsa-miR-181d-5p; hsa-miR-93-5p; hsa-miR-181b-5p; hsa-miR-124-3p; hsa-miR-21-5p; hsa-miR-29a-3p; hsa-miR-26a-5p; hsa-miR-99b-3p; hsa-miR-1229-3p; hsa-miR-148a-3p; hsa-miR-940; hsa-miR-320c; hsa-miR-1224-5p; hsa-miR-19b-3p; hsa-miR-25-3p; hsa-miR-766-3p; hsa-miR-17-5p; hsa-miR-18a-5p; hsa-miR-10a-5p; hsa-miR-129-1-3p; hsa-miR-15b-5p; hsa-miR-148b-3p; hsa-miR-149-5p; hsa-miR-23a-3p; hsa-miR-92a-3p; hsa-miR-615-3p; hsa-miR-505-3p; hsa-miR-1260b; hsa-miR-30b-5p; hsa-miR-130b-5p; hsa-miR-204-5p; hsa-miR-1226-3p;

					hsa-miR-331-3p; hsa-miR-197-3p; hsa-miR-346; hsa-miR-30c-5p; hsa-miR-328-3p; hsa-miR-1296-5p
Gene Ontology (miRWalk)	GO0070848 response to growth factor stimulus	enriched	0.020816	24	hsa-let-7c-5p; hsa-let-7a-5p; hsa-let-7f-5p; hsa-miR-1-3p; hsa-miR-26b-5p; hsa-miR-320a; hsa-let-7g-5p; hsa-miR-451a; hsa-miR-146a-5p; hsa-miR-16-5p; hsa-miR-20a-5p; hsa-miR-24-3p; hsa-miR-92b-3p; hsa-miR-181a-5p; hsa-miR-181b-5p; hsa-miR-124-3p; hsa-miR-21-5p; hsa-miR-26a-5p; hsa-miR-449c-5p; hsa-miR-940; hsa-miR-17-5p; hsa-miR-504-5p; hsa-miR-30b-5p; hsa-miR-34b-3p
Gene Ontology (miRWalk)	GO0043353 enucleate erythrocyte differentiation	enriched	0.0208319	17	hsa-let-7a-5p; hsa-let-7f-5p; hsa-miR-1-3p; hsa-miR-26b-5p; hsa-miR-103a-3p; hsa-miR-106b-5p; hsa-miR-16-5p; hsa-miR-20a-5p; hsa-miR-29b-3p; hsa-miR-93-5p; hsa-miR-124-3p; hsa-miR-21-5p; hsa-miR-223-3p; hsa-miR-26a-5p; hsa-miR-17-5p; hsa-miR-149-5p; hsa-miR-1260b
Pathways (miRWalk)	WP69 TCR Signaling Pathway	enriched	0.0211281	53	hsa-miR-29c-3p; hsa-let-7c-5p; hsa-miR-155-5p; hsa-miR-193b-5p; hsa-let-7a-5p; hsa-miR-1-3p; hsa-miR-877-5p; hsa-miR-26b-5p; hsa-miR-320a; hsa-miR-103a-3p; hsa-miR-106b-5p; hsa-miR-451a; hsa-miR-184; hsa-miR-146a-5p; hsa-miR-107; hsa-miR-16-5p; hsa-miR-20a-5p; hsa-miR-24-3p; hsa-miR-92b-3p; hsa-miR-29b-3p; hsa-miR-365a-3p; hsa-miR-181a-5p; hsa-miR-483-5p; hsa-miR-181d-5p; hsa-miR-93-5p; hsa-miR-124-3p; hsa-miR-21-5p; hsa-miR-223-3p; hsa-miR-29a-3p; hsa-miR-26a-5p; hsa-miR-1914-3p; hsa-miR-15a-5p; hsa-miR-19b-3p; hsa-miR-766-3p; hsa-miR-17-5p; hsa-miR-10a-5p; hsa-miR-129-1-3p; hsa-miR-15b-5p; hsa-miR-149-5p; hsa-miR-3605-3p; hsa-miR-23a-3p; hsa-miR-504-5p; hsa-miR-92a-3p; hsa-miR-615-3p; hsa-miR-1260b; hsa-miR-30b-5p; hsa-miR-130b-5p; hsa-miR-204-5p; hsa-miR-331-3p; hsa-miR-346; hsa-miR-744-3p; hsa-miR-34b-3p; hsa-miR-30c-5p
Pathways (miRWalk)	WP2035 FSH signaling pathway	enriched	0.0212595	29	hsa-miR-155-5p; hsa-miR-324-3p; hsa-let-7f-5p; hsa-miR-1-3p; hsa-miR-877-5p; hsa-miR-26b-5p; hsa-miR-320a; hsa-miR-103a-3p; hsa-miR-451a; hsa-miR-16-5p; hsa-miR-24-3p; hsa-miR-92b-3p; hsa-miR-365a-3p; hsa-miR-483-5p; hsa-miR-181b-5p; hsa-miR-124-3p; hsa-miR-21-5p; hsa-miR-223-3p; hsa-miR-19a-3p; hsa-miR-15a-5p; hsa-miR-19b-3p; hsa-miR-766-3p; hsa-miR-17-5p; hsa-miR-15b-5p; hsa-miR-92a-3p; hsa-miR-505-3p; hsa-miR-1260b; hsa-miR-331-3p; hsa-miR-1296-5p
Pathways (miRWalk)	WP2118 Arrhythmogenic right ventricular cardiomyopathy	enriched	0.0212595	38	hsa-let-7c-5p; hsa-miR-155-5p; hsa-let-7a-5p; hsa-miR-324-3p; hsa-let-7f-5p; hsa-miR-1-3p; hsa-miR-877-5p; hsa-miR-26b-5p; hsa-miR-320a; hsa-miR-103a-3p; hsa-miR-1227-3p; hsa-miR-1295a; hsa-miR-142-3p; hsa-miR-16-5p; hsa-miR-24-3p; hsa-miR-92b-3p; hsa-miR-671-5p; hsa-miR-93-5p; hsa-miR-124-3p; hsa-miR-21-5p; hsa-miR-29a-3p; hsa-miR-26a-5p; hsa-miR-99b-3p; hsa-miR-1229-3p; hsa-miR-25-3p; hsa-miR-17-5p; hsa-miR-10a-5p; hsa-miR-15b-5p; hsa-miR-148b-3p; hsa-miR-149-5p; hsa-miR-23a-3p; hsa-miR-92a-3p; hsa-miR-615-3p; hsa-miR-204-5p; hsa-miR-1226-3p; hsa-miR-331-3p; hsa-miR-197-3p; hsa-miR-30c-5p

Pathways (miRWalk)	WP272 Blood Clotting Cascade	enriched	0.0212595	11	hsa-miR-29c-3p; hsa-miR-155-5p; hsa-miR-1-3p; hsa-miR-26b-5p; hsa-miR-16-5p; hsa-miR-29b-3p; hsa-miR-124-3p; hsa-miR-21-5p; hsa-miR-29a-3p; hsa-miR-204-5p; hsa-miR-30c-5p
Pathways (miRWalk)	WP49 IL 2 Signaling pathway	enriched	0.0212595	55	hsa-miR-29c-3p; hsa-let-7c-5p; hsa-miR-155-5p; hsa-let-7a-5p; hsa-miR-324-3p; hsa-let-7f-5p; hsa-miR-1-3p; hsa-miR-877-5p; hsa-miR-26b-5p; hsa-miR-320a; hsa-let-7g-5p; hsa-miR-103a-3p; hsa-miR-106b-5p; hsa-miR-451a; hsa-miR-146a-5p; hsa-miR-16-5p; hsa-miR-20a-5p; hsa-miR-24-3p; hsa-miR-92b-3p; hsa-miR-29b-3p; hsa-miR-365a-3p; hsa-miR-181a-5p; hsa-miR-483-5p; hsa-miR-181d-5p; hsa-miR-93-5p; hsa-miR-181b-5p; hsa-miR-124-3p; hsa-miR-21-5p; hsa-miR-29a-3p; hsa-miR-26a-5p; hsa-miR-99b-3p; hsa-miR-1229-3p; hsa-miR-15a-5p; hsa-miR-449c-5p; hsa-miR-20b-5p; hsa-miR-148a-3p; hsa-miR-19b-3p; hsa-miR-766-3p; hsa-miR-17-5p; hsa-miR-630; hsa-miR-15b-5p; hsa-miR-23a-3p; hsa-miR-92a-3p; hsa-miR-615-3p; hsa-miR-1260b; hsa-miR-30b-5p; hsa-miR-130b-5p; hsa-miR-204-5p; hsa-miR-337-3p; hsa-miR-331-3p; hsa-miR-197-3p; hsa-miR-34b-3p; hsa-miR-30c-5p; hsa-miR-296-5p; hsa-miR-1296-5p
Pathways (miRWalk)	hsa04012 ErbB signaling pathway	enriched	0.0212595	61	hsa-miR-29c-3p; hsa-let-7c-5p; hsa-miR-155-5p; hsa-miR-193b-5p; hsa-let-7a-5p; hsa-miR-324-3p; hsa-let-7f-5p; hsa-miR-1-3p; hsa-miR-877-5p; hsa-miR-26b-5p; hsa-miR-320a; hsa-let-7g-5p; hsa-miR-103a-3p; hsa-miR-199b-5p; hsa-miR-106b-5p; hsa-miR-451a; hsa-miR-184; hsa-miR-146a-5p; hsa-miR-548d-3p; hsa-miR-107; hsa-miR-16-5p; hsa-miR-20a-5p; hsa-miR-24-3p; hsa-miR-92b-3p; hsa-miR-181a-5p; hsa-miR-483-5p; hsa-miR-181d-5p; hsa-miR-93-5p; hsa-miR-181b-5p; hsa-miR-124-3p; hsa-miR-21-5p; hsa-miR-29a-3p; hsa-miR-26a-5p; hsa-miR-19a-3p; hsa-miR-1229-3p; hsa-miR-1914-3p; hsa-miR-15a-5p; hsa-miR-449c-5p; hsa-miR-520a-3p; hsa-miR-20b-5p; hsa-miR-766-3p; hsa-miR-17-5p; hsa-miR-572; hsa-miR-10a-5p; hsa-miR-129-1-3p; hsa-miR-15b-5p; hsa-miR-148b-3p; hsa-miR-149-5p; hsa-miR-92a-3p; hsa-miR-299-5p; hsa-miR-615-3p; hsa-miR-505-3p; hsa-miR-1260b; hsa-miR-30b-5p; hsa-miR-331-3p; hsa-miR-346; hsa-miR-744-3p; hsa-miR-34b-3p; hsa-miR-30c-5p; hsa-miR-296-5p; hsa-miR-1296-5p
Pathways (miRWalk)	hsa04350 TGF beta signaling pathway	enriched	0.0212595	54	hsa-let-7c-5p; hsa-miR-155-5p; hsa-let-7a-5p; hsa-miR-584-5p; hsa-miR-324-3p; hsa-let-7f-5p; hsa-miR-30d-5p; hsa-miR-1-3p; hsa-miR-877-5p; hsa-miR-26b-5p; hsa-miR-320a; hsa-let-7g-5p; hsa-miR-103a-3p; hsa-miR-106b-5p; hsa-miR-451a; hsa-miR-146a-5p; hsa-miR-142-3p; hsa-miR-16-5p; hsa-miR-20a-5p; hsa-miR-24-3p; hsa-miR-92b-3p; hsa-miR-29b-3p; hsa-miR-483-5p; hsa-miR-181d-5p; hsa-miR-93-5p; hsa-miR-181b-5p; hsa-miR-124-3p; hsa-miR-21-5p; hsa-miR-29a-3p; hsa-miR-26a-5p; hsa-miR-19a-3p; hsa-miR-15a-5p; hsa-miR-449c-5p; hsa-miR-148a-3p; hsa-miR-320c; hsa-miR-19b-3p; hsa-miR-25-3p; hsa-miR-766-3p; hsa-miR-17-5p; hsa-miR-18a-5p; hsa-miR-10a-5p; hsa-miR-15b-5p; hsa-miR-148b-3p; hsa-miR-149-5p; hsa-miR-92a-3p; hsa-miR-615-3p; hsa-miR-483-3p; hsa-miR-30b-5p; hsa-miR-130b-5p; hsa-miR-204-5p; hsa-miR-197-3p; hsa-miR-34b-3p; hsa-miR-30c-5p; hsa-miR-1296-5p
Pathways (miRWalk)	hsa04370 VEGF signaling pathway	enriched	0.0212595	51	hsa-miR-29c-3p; hsa-let-7c-5p; hsa-miR-155-5p; hsa-let-7a-5p; hsa-miR-324-3p; hsa-miR-1-3p; hsa-miR-877-5p; hsa-miR-26b-5p; hsa-miR-320a; hsa-let-7g-5p; hsa-miR-106b-5p; hsa-miR-451a; hsa-miR-184; hsa-miR-142-3p; hsa-miR-107; hsa-miR-16-5p; hsa-miR-20a-5p; hsa-miR-24-3p; hsa-miR-92b-3p; hsa-miR-29b-3p; hsa-miR-181a-5p; hsa-miR-483-5p; hsa-miR-181d-5p; hsa-miR-93-5p; hsa-miR-124-3p; hsa-miR-21-5p; hsa-miR-29a-3p; hsa-miR-26a-5p; hsa-miR-134-5p; hsa-miR-1229-3p; hsa-miR-1914-3p; hsa-miR-15a-5p; hsa-miR-449c-5p; hsa-miR-20b-5p; hsa-miR-520g-3p; hsa-miR-766-3p; hsa-miR-17-5p; hsa-miR-10a-5p; hsa-miR-15b-5p; hsa-miR-148b-3p; hsa-miR-149-5p; hsa-miR-504-

					5p; hsa-miR-92a-3p; hsa-miR-615-3p; hsa-miR-505-3p; hsa-miR-331-3p; hsa-miR-346; hsa-miR-744-3p; hsa-miR-34b-3p; hsa-miR-30c-5p; hsa-miR-1296-5p
Pathways (miRWalk)	hsa05410 Hypertrophic cardiomyopathy HCM	enriched	0.0212595	40	hsa-miR-29c-3p; hsa-let-7c-5p; hsa-miR-155-5p; hsa-let-7a-5p; hsa-let-7f-5p; hsa-miR-1-3p; hsa-miR-877-5p; hsa-miR-26b-5p; hsa-miR-320a; hsa-miR-103a-3p; hsa-miR-1227-3p; hsa-miR-1295a; hsa-miR-142-3p; hsa-miR-16-5p; hsa-miR-24-3p; hsa-miR-29b-3p; hsa-miR-671-5p; hsa-miR-365a-3p; hsa-miR-93-5p; hsa-miR-124-3p; hsa-miR-21-5p; hsa-miR-29a-3p; hsa-miR-26a-5p; hsa-miR-99b-3p; hsa-miR-1229-3p; hsa-miR-940; hsa-miR-19b-3p; hsa-miR-25-3p; hsa-miR-17-5p; hsa-miR-10a-5p; hsa-miR-15b-5p; hsa-miR-148b-3p; hsa-miR-149-5p; hsa-miR-92a-3p; hsa-miR-615-3p; hsa-miR-204-5p; hsa-miR-1226-3p; hsa-miR-331-3p; hsa-miR-197-3p; hsa-miR-30c-5p
Pathways (miRWalk)	hsa05412 Arrhythmogenic right ventricular cardiomyopathy ARVC	enriched	0.0212595	38	hsa-let-7c-5p; hsa-miR-155-5p; hsa-let-7a-5p; hsa-miR-324-3p; hsa-let-7f-5p; hsa-miR-1-3p; hsa-miR-877-5p; hsa-miR-26b-5p; hsa-miR-320a; hsa-miR-103a-3p; hsa-miR-1227-3p; hsa-miR-1295a; hsa-miR-142-3p; hsa-miR-16-5p; hsa-miR-24-3p; hsa-miR-92b-3p; hsa-miR-671-5p; hsa-miR-93-5p; hsa-miR-124-3p; hsa-miR-21-5p; hsa-miR-29a-3p; hsa-miR-26a-5p; hsa-miR-99b-3p; hsa-miR-1229-3p; hsa-miR-25-3p; hsa-miR-17-5p; hsa-miR-10a-5p; hsa-miR-15b-5p; hsa-miR-148b-3p; hsa-miR-149-5p; hsa-miR-23a-3p; hsa-miR-92a-3p; hsa-miR-615-3p; hsa-miR-204-5p; hsa-miR-1226-3p; hsa-miR-331-3p; hsa-miR-197-3p; hsa-miR-30c-5p
Gene Ontology (miRWalk)	GO0051045 negative regulation of membrane protein ectodomain proteolysis	enriched	0.0224383	8	hsa-miR-1-3p; hsa-miR-26b-5p; hsa-miR-103a-3p; hsa-miR-18b-5p; hsa-miR-16-5p; hsa-miR-181b-5p; hsa-miR-124-3p; hsa-miR-21-5p
Gene Ontology (miRWalk)	GO0051205 protein insertion into membrane	enriched	0.0224383	8	hsa-miR-155-5p; hsa-let-7a-5p; hsa-miR-1-3p; hsa-miR-26b-5p; hsa-miR-146a-5p; hsa-miR-16-5p; hsa-miR-671-5p; hsa-miR-21-5p
Gene Ontology (miRWalk)	GO0000981 sequence specific dna binding rna polymerase ii transcription factor activity	enriched	0.0224879	23	hsa-let-7c-5p; hsa-miR-155-5p; hsa-let-7f-5p; hsa-miR-1-3p; hsa-miR-877-5p; hsa-miR-26b-5p; hsa-miR-320a; hsa-miR-103a-3p; hsa-miR-16-5p; hsa-miR-20a-5p; hsa-miR-92b-3p; hsa-miR-181a-5p; hsa-miR-93-5p; hsa-miR-181b-5p; hsa-miR-124-3p; hsa-miR-21-5p; hsa-miR-223-3p; hsa-miR-19b-3p; hsa-miR-17-5p; hsa-miR-10a-5p; hsa-miR-148b-3p; hsa-miR-92a-3p; hsa-miR-30c-5p
Gene Ontology (miRWalk)	GO0003676 nucleic acid binding	enriched	0.0224879	60	hsa-miR-29c-3p; hsa-let-7c-5p; hsa-miR-155-5p; hsa-miR-193b-5p; hsa-let-7a-5p; hsa-miR-324-3p; hsa-let-7f-5p; hsa-miR-193a-5p; hsa-miR-30d-5p; hsa-miR-1-3p; hsa-miR-877-5p; hsa-miR-26b-5p; hsa-miR-320a; hsa-miR-103a-3p; hsa-miR-106b-5p; hsa-miR-18b-5p; hsa-miR-760; hsa-miR-142-3p; hsa-miR-425-3p; hsa-miR-107; hsa-miR-16-5p; hsa-miR-20a-5p; hsa-miR-24-3p; hsa-miR-92b-3p; hsa-miR-29b-3p; hsa-miR-671-5p; hsa-miR-365a-3p; hsa-miR-181a-5p; hsa-miR-93-5p; hsa-miR-181b-5p; hsa-miR-124-3p; hsa-miR-21-5p; hsa-miR-29a-3p; hsa-miR-26a-5p; hsa-miR-99b-3p; hsa-miR-19a-3p; hsa-miR-1229-3p; hsa-miR-1914-3p; hsa-miR-148a-3p; hsa-miR-19b-3p; hsa-miR-25-3p; hsa-miR-17-5p; hsa-miR-18a-5p; hsa-miR-10a-5p; hsa-miR-15b-5p; hsa-miR-148b-3p; hsa-

					miR-149-5p; hsa-miR-504-5p; hsa-miR-92a-3p; hsa-miR-615-3p; hsa-miR-505-3p; hsa-miR-574-3p; hsa-miR-1260b; hsa-miR-30b-5p; hsa-miR-130b-5p; hsa-miR-1226-3p; hsa-miR-331-3p; hsa-miR-197-3p; hsa-miR-346; hsa-miR-30c-5p
Gene Ontology (miRWalk)	GO0005791 rough endoplasmic reticulum	enriched	0.0224879	17	hsa-miR-155-5p; hsa-miR-1-3p; hsa-miR-877-5p; hsa-miR-26b-5p; hsa-miR-103a-3p; hsa-miR-451a; hsa-miR-146a-5p; hsa-miR-16-5p; hsa-miR-20a-5p; hsa-miR-181a-5p; hsa-miR-93-5p; hsa-miR-124-3p; hsa-miR-99b-3p; hsa-miR-19a-3p; hsa-miR-148b-3p; hsa-miR-92a-3p; hsa-miR-204-5p
Gene Ontology (miRWalk)	GO0007492 endoderm development	enriched	0.0224879	24	hsa-miR-29c-3p; hsa-miR-155-5p; hsa-miR-324-3p; hsa-let-7f-5p; hsa-miR-1-3p; hsa-miR-320a; hsa-miR-18b-5p; hsa-miR-146a-5p; hsa-miR-142-3p; hsa-miR-16-5p; hsa-miR-20a-5p; hsa-miR-24-3p; hsa-miR-181a-5p; hsa-miR-93-5p; hsa-miR-124-3p; hsa-miR-21-5p; hsa-miR-26a-5p; hsa-miR-19a-3p; hsa-miR-19b-3p; hsa-miR-17-5p; hsa-miR-18a-5p; hsa-miR-10a-5p; hsa-miR-92a-3p; hsa-miR-483-3p
Gene Ontology (miRWalk)	GO0010224 response to uv b	enriched	0.0224879	25	hsa-miR-29c-3p; hsa-miR-155-5p; hsa-let-7a-5p; hsa-miR-1-3p; hsa-miR-26b-5p; hsa-miR-320a; hsa-miR-103a-3p; hsa-miR-451a; hsa-miR-16-5p; hsa-miR-20a-5p; hsa-miR-29b-3p; hsa-miR-365a-3p; hsa-miR-181a-5p; hsa-miR-181d-5p; hsa-miR-181b-5p; hsa-miR-124-3p; hsa-miR-21-5p; hsa-miR-29a-3p; hsa-miR-15a-5p; hsa-miR-148a-3p; hsa-miR-17-5p; hsa-miR-630; hsa-miR-15b-5p; hsa-miR-204-5p; hsa-miR-296-5p
Gene Ontology (miRWalk)	GO0012506 vesicle membrane	enriched	0.0224879	14	hsa-miR-155-5p; hsa-let-7a-5p; hsa-miR-324-3p; hsa-miR-1-3p; hsa-miR-16-5p; hsa-miR-24-3p; hsa-miR-92b-3p; hsa-miR-93-5p; hsa-miR-124-3p; hsa-miR-21-5p; hsa-miR-223-3p; hsa-miR-26a-5p; hsa-miR-148b-3p; hsa-miR-328-3p
Gene Ontology (miRWalk)	GO0016442 rna induced silencing complex	enriched	0.0224879	23	hsa-miR-29c-3p; hsa-let-7c-5p; hsa-let-7a-5p; hsa-miR-30d-5p; hsa-miR-1-3p; hsa-miR-26b-5p; hsa-miR-320a; hsa-miR-103a-3p; hsa-miR-107; hsa-miR-16-5p; hsa-miR-24-3p; hsa-miR-154-5p; hsa-miR-671-5p; hsa-miR-93-5p; hsa-miR-124-3p; hsa-miR-21-5p; hsa-miR-29a-3p; hsa-miR-19b-3p; hsa-miR-18a-5p; hsa-miR-148b-3p; hsa-miR-92a-3p; hsa-miR-505-3p; hsa-miR-331-3p
Gene Ontology (miRWalk)	GO0031012 extracellular matrix	enriched	0.0224879	36	hsa-miR-29c-3p; hsa-miR-155-5p; hsa-let-7a-5p; hsa-miR-1-3p; hsa-miR-26b-5p; hsa-miR-320a; hsa-let-7g-5p; hsa-miR-103a-3p; hsa-miR-106b-5p; hsa-miR-18b-5p; hsa-miR-760; hsa-miR-146a-5p; hsa-miR-16-5p; hsa-miR-20a-5p; hsa-miR-29b-3p; hsa-miR-671-5p; hsa-miR-181a-5p; hsa-miR-181d-5p; hsa-miR-93-5p; hsa-miR-181b-5p; hsa-miR-124-3p; hsa-miR-21-5p; hsa-miR-29a-3p; hsa-miR-26a-5p; hsa-miR-17-5p; hsa-miR-10a-5p; hsa-miR-148b-3p; hsa-miR-663a; hsa-miR-26b-3p; hsa-miR-92a-3p; hsa-miR-615-3p; hsa-miR-1260b; hsa-miR-30b-5p; hsa-miR-204-5p; hsa-miR-197-3p; hsa-miR-30c-5p
Gene Ontology (miRWalk)	GO0031424 keratinization	enriched	0.0224879	8	hsa-miR-29c-3p; hsa-miR-155-5p; hsa-miR-1-3p; hsa-miR-26b-5p; hsa-miR-106b-5p; hsa-miR-29b-3p; hsa-miR-124-3p; hsa-miR-29a-3p

Gene Ontology (miRWalk)	GO0032091 negative regulation of protein binding	enriched	0.0224879	32	hsa-miR-29c-3p; hsa-let-7c-5p; hsa-miR-155-5p; hsa-let-7a-5p; hsa-let-7f-5p; hsa-miR-1-3p; hsa-miR-877-5p; hsa-miR-26b-5p; hsa-miR-320a; hsa-let-7g-5p; hsa-miR-103a-3p; hsa-miR-451a; hsa-miR-146a-5p; hsa-miR-16-5p; hsa-miR-20a-5p; hsa-miR-24-3p; hsa-miR-92b-3p; hsa-miR-29b-3p; hsa-miR-365a-3p; hsa-miR-124-3p; hsa-miR-21-5p; hsa-miR-26a-5p; hsa-miR-449c-5p; hsa-miR-17-5p; hsa-miR-10a-5p; hsa-miR-148b-3p; hsa-miR-504-5p; hsa-miR-92a-3p; hsa-miR-615-3p; hsa-miR-34b-3p; hsa-miR-296-5p; hsa-miR-328-3p
Gene Ontology (miRWalk)	GO0042060 wound healing	enriched	0.0224879	34	hsa-miR-29c-3p; hsa-let-7c-5p; hsa-miR-155-5p; hsa-let-7a-5p; hsa-miR-30d-5p; hsa-miR-1-3p; hsa-miR-26b-5p; hsa-miR-320a; hsa-let-7g-5p; hsa-miR-103a-3p; hsa-miR-199b-5p; hsa-miR-106b-5p; hsa-miR-548d-3p; hsa-miR-142-3p; hsa-miR-16-5p; hsa-miR-20a-5p; hsa-miR-24-3p; hsa-miR-29b-3p; hsa-miR-181a-5p; hsa-miR-124-3p; hsa-miR-21-5p; hsa-miR-223-3p; hsa-miR-26a-5p; hsa-miR-17-5p; hsa-miR-18a-5p; hsa-miR-148b-3p; hsa-miR-92a-3p; hsa-miR-615-3p; hsa-miR-30b-5p; hsa-miR-204-5p; hsa-miR-331-3p; hsa-miR-197-3p; hsa-miR-346; hsa-miR-30c-5p
Gene Ontology (miRWalk)	GO0045646 regulation of erythrocyte differentiation	enriched	0.0224879	14	hsa-miR-29c-3p; hsa-miR-155-5p; hsa-let-7a-5p; hsa-miR-26b-5p; hsa-miR-103a-3p; hsa-miR-107; hsa-miR-16-5p; hsa-miR-29b-3p; hsa-miR-124-3p; hsa-miR-21-5p; hsa-miR-29a-3p; hsa-miR-26a-5p; hsa-miR-615-3p; hsa-miR-34b-3p
Gene Ontology (miRWalk)	GO0045727 positive regulation of translation	enriched	0.0224879	40	hsa-miR-155-5p; hsa-let-7a-5p; hsa-miR-324-3p; hsa-miR-1-3p; hsa-miR-877-5p; hsa-miR-26b-5p; hsa-miR-320a; hsa-miR-103a-3p; hsa-miR-199b-5p; hsa-miR-760; hsa-miR-1295a; hsa-miR-548d-3p; hsa-miR-16-5p; hsa-miR-20a-5p; hsa-miR-24-3p; hsa-miR-92b-3p; hsa-miR-29b-3p; hsa-miR-365a-3p; hsa-miR-181a-5p; hsa-miR-181d-5p; hsa-miR-93-5p; hsa-miR-181b-5p; hsa-miR-124-3p; hsa-miR-21-5p; hsa-miR-29a-3p; hsa-miR-26a-5p; hsa-miR-25-3p; hsa-miR-17-5p; hsa-miR-10a-5p; hsa-miR-149-5p; hsa-miR-23a-3p; hsa-miR-129-2-3p; hsa-miR-92a-3p; hsa-miR-615-3p; hsa-miR-629-3p; hsa-miR-30b-5p; hsa-miR-130b-5p; hsa-miR-204-5p; hsa-miR-331-3p; hsa-miR-30c-5p
Gene Ontology (miRWalk)	GO0045869 negative regulation of retroviral genome replication	enriched	0.0224879	7	hsa-let-7c-5p; hsa-let-7a-5p; hsa-let-7f-5p; hsa-miR-26b-5p; hsa-let-7g-5p; hsa-miR-760; hsa-miR-26a-5p
Gene Ontology (miRWalk)	GO0046854 phosphatidylinositol phosphorylation	enriched	0.0224879	25	hsa-let-7c-5p; hsa-miR-155-5p; hsa-let-7a-5p; hsa-miR-324-3p; hsa-miR-1-3p; hsa-miR-877-5p; hsa-miR-26b-5p; hsa-miR-320a; hsa-miR-103a-3p; hsa-miR-184; hsa-miR-16-5p; hsa-miR-92b-3p; hsa-miR-671-5p; hsa-miR-365a-3p; hsa-miR-181a-5p; hsa-miR-93-5p; hsa-miR-21-5p; hsa-miR-29a-3p; hsa-miR-17-5p; hsa-miR-10a-5p; hsa-miR-15b-5p; hsa-miR-148b-3p; hsa-miR-92a-3p; hsa-miR-615-3p; hsa-miR-346
Gene Ontology (miRWalk)	GO0051000 positive regulation of nitric oxide synthase activity	enriched	0.0224879	24	hsa-miR-155-5p; hsa-let-7a-5p; hsa-miR-1-3p; hsa-miR-26b-5p; hsa-let-7g-5p; hsa-miR-199b-5p; hsa-miR-18b-5p; hsa-miR-451a; hsa-miR-142-3p; hsa-miR-107; hsa-miR-16-5p; hsa-miR-20a-5p; hsa-miR-29b-3p; hsa-miR-181a-5p; hsa-miR-124-3p; hsa-miR-223-3p; hsa-miR-26a-5p; hsa-miR-19a-3p; hsa-miR-20b-5p; hsa-miR-19b-3p; hsa-miR-18a-5p; hsa-miR-148b-3p; hsa-miR-92a-3p; hsa-miR-615-3p
Gene Ontology (miRWalk)	GO0070534 protein k63 linked ubiquitination	enriched	0.0224879	24	hsa-miR-155-5p; hsa-let-7a-5p; hsa-miR-324-3p; hsa-miR-877-5p; hsa-miR-26b-5p; hsa-miR-320a; hsa-let-7g-5p; hsa-miR-106b-5p; hsa-miR-18b-5p; hsa-miR-146a-5p; hsa-miR-16-5p; hsa-miR-24-3p; hsa-miR-92b-3p; hsa-miR-93-5p; hsa-miR-124-3p; hsa-miR-29a-3p; hsa-miR-26a-5p; hsa-miR-19a-3p; hsa-miR-17-5p; hsa-miR-18a-5p; hsa-miR-149-5p; hsa-miR-615-3p; hsa-miR-1226-3p; hsa-miR-30c-5p
Gene Ontology (miRWalk)	GO0097361 cia complex	enriched	0.0224879	6	hsa-miR-29c-3p; hsa-miR-155-5p; hsa-miR-26b-5p; hsa-miR-16-5p; hsa-miR-24-3p; hsa-miR-92b-3p

Gene Ontology (miRWalk)	GO2000145 regulation of cell motility	enriched	0.0224879	26	hsa-miR-29c-3p; hsa-miR-155-5p; hsa-let-7a-5p; hsa-miR-584-5p; hsa-miR-26b-5p; hsa-miR-103a-3p; hsa-miR-146a-5p; hsa-miR-425-3p; hsa-miR-107; hsa-miR-16-5p; hsa-miR-24-3p; hsa-miR-29b-3p; hsa-miR-181a-5p; hsa-miR-93-5p; hsa-miR-124-3p; hsa-miR-21-5p; hsa-miR-29a-3p; hsa-miR-26a-5p; hsa-miR-99b-3p; hsa-miR-15a-5p; hsa-miR-17-5p; hsa-miR-148b-3p; hsa-miR-149-5p; hsa-miR-92a-3p; hsa-miR-615-3p; hsa-miR-34b-3p
Pathways (miRWalk)	hsa05213 Endometrial cancer	enriched	0.02279	61	hsa-miR-29c-3p; hsa-let-7c-5p; hsa-miR-155-5p; hsa-let-7a-5p; hsa-let-7f-5p; hsa-miR-30d-5p; hsa-miR-1-3p; hsa-miR-26b-5p; hsa-miR-320a; hsa-let-7g-5p; hsa-miR-103a-3p; hsa-miR-199b-5p; hsa-miR-106b-5p; hsa-miR-451a; hsa-miR-184; hsa-miR-146a-5p; hsa-miR-548d-3p; hsa-miR-107; hsa-miR-16-5p; hsa-miR-20a-5p; hsa-miR-24-3p; hsa-miR-92b-3p; hsa-miR-29b-3p; hsa-miR-365a-3p; hsa-miR-181a-5p; hsa-miR-483-5p; hsa-miR-181d-5p; hsa-miR-93-5p; hsa-miR-181b-5p; hsa-miR-124-3p; hsa-miR-21-5p; hsa-miR-29a-3p; hsa-miR-26a-5p; hsa-miR-19a-3p; hsa-miR-15a-5p; hsa-miR-449c-5p; hsa-miR-148a-3p; hsa-miR-320c; hsa-miR-19b-3p; hsa-miR-494-3p; hsa-miR-25-3p; hsa-miR-766-3p; hsa-miR-17-5p; hsa-miR-18a-5p; hsa-miR-10a-5p; hsa-miR-15b-5p; hsa-miR-148b-3p; hsa-miR-149-5p; hsa-miR-23a-3p; hsa-miR-504-5p; hsa-miR-92a-3p; hsa-miR-615-3p; hsa-miR-1226-3p; hsa-miR-331-3p; hsa-miR-197-3p; hsa-miR-346; hsa-miR-34b-3p; hsa-miR-30c-5p; hsa-miR-605-5p; hsa-miR-296-5p; hsa-miR-1296-5p
Pathways (miRWalk)	P00035 Interferon gamma signaling pathway	enriched	0.0228868	31	hsa-miR-155-5p; hsa-let-7a-5p; hsa-miR-877-5p; hsa-miR-26b-5p; hsa-miR-320a; hsa-miR-103a-3p; hsa-miR-106b-5p; hsa-miR-146a-5p; hsa-miR-16-5p; hsa-miR-20a-5p; hsa-miR-24-3p; hsa-miR-92b-3p; hsa-miR-29b-3p; hsa-miR-181a-5p; hsa-miR-483-5p; hsa-miR-93-5p; hsa-miR-181b-5p; hsa-miR-124-3p; hsa-miR-21-5p; hsa-miR-15a-5p; hsa-miR-19b-3p; hsa-miR-766-3p; hsa-miR-17-5p; hsa-miR-10a-5p; hsa-miR-15b-5p; hsa-miR-23a-3p; hsa-miR-92a-3p; hsa-miR-615-3p; hsa-miR-130b-5p; hsa-miR-30c-5p; hsa-miR-1296-5p
Pathways (miRWalk)	WP2012 miRs in Muscle Cell Differentiation	enriched	0.0228868	32	hsa-let-7c-5p; hsa-miR-155-5p; hsa-let-7a-5p; hsa-miR-324-3p; hsa-let-7f-5p; hsa-miR-30d-5p; hsa-miR-1-3p; hsa-miR-877-5p; hsa-miR-26b-5p; hsa-miR-320a; hsa-miR-103a-3p; hsa-miR-16-5p; hsa-miR-20a-5p; hsa-miR-92b-3p; hsa-miR-365a-3p; hsa-miR-93-5p; hsa-miR-124-3p; hsa-miR-21-5p; hsa-miR-223-3p; hsa-miR-26a-5p; hsa-miR-19b-3p; hsa-miR-25-3p; hsa-miR-17-5p; hsa-miR-148b-3p; hsa-miR-92a-3p; hsa-miR-615-3p; hsa-miR-505-3p; hsa-miR-1260b; hsa-miR-204-5p; hsa-miR-331-3p; hsa-miR-197-3p; hsa-miR-1296-5p
Pathways (miRWalk)	WP437 EGF EGFR Signaling Pathway	enriched	0.0228868	68	hsa-miR-29c-3p; hsa-let-7c-5p; hsa-miR-155-5p; hsa-miR-193b-5p; hsa-let-7a-5p; hsa-miR-324-3p; hsa-let-7f-5p; hsa-miR-1-3p; hsa-miR-877-5p; hsa-miR-26b-5p; hsa-miR-320a; hsa-let-7g-5p; hsa-miR-103a-3p; hsa-miR-199b-5p; hsa-miR-106b-5p; hsa-miR-451a; hsa-miR-184; hsa-miR-146a-5p; hsa-miR-548d-3p; hsa-miR-142-3p; hsa-miR-107; hsa-miR-16-5p; hsa-miR-20a-5p; hsa-miR-24-3p; hsa-miR-92b-3p; hsa-miR-29b-3p; hsa-miR-671-5p; hsa-miR-181a-5p; hsa-miR-181d-5p; hsa-miR-93-5p; hsa-miR-181b-5p; hsa-miR-124-3p; hsa-miR-21-5p; hsa-miR-223-3p; hsa-miR-29a-3p; hsa-miR-26a-5p; hsa-miR-19a-3p; hsa-miR-1229-3p; hsa-miR-1914-3p; hsa-miR-15a-5p; hsa-miR-20b-5p; hsa-miR-148a-3p; hsa-miR-19b-3p; hsa-miR-494-3p; hsa-miR-25-3p; hsa-miR-766-3p; hsa-miR-17-5p; hsa-miR-18a-5p; hsa-miR-10a-5p; hsa-miR-129-1-3p; hsa-miR-15b-5p; hsa-miR-149-5p; hsa-miR-663a; hsa-miR-23a-3p; hsa-miR-92a-3p; hsa-miR-615-3p; hsa-miR-505-3p; hsa-miR-1260b; hsa-miR-30b-5p; hsa-miR-130b-5p; hsa-miR-337-3p; hsa-miR-1226-3p; hsa-miR-331-3p; hsa-miR-1914-5p; hsa-miR-744-3p; hsa-miR-30c-5p; hsa-miR-296-5p; hsa-miR-1296-5p

Pathways (miRWalk)	hsa04360 Axon guidance	enriched	0.0228868	55	hsa-miR-29c-3p; hsa-let-7c-5p; hsa-miR-155-5p; hsa-let-7a-5p; hsa-miR-584-5p; hsa-miR-324-3p; hsa-miR-30d-5p; hsa-miR-1-3p; hsa-miR-877-5p; hsa-miR-26b-5p; hsa-miR-320a; hsa-let-7g-5p; hsa-miR-103a-3p; hsa-miR-106b-5p; hsa-miR-184; hsa-miR-146a-5p; hsa-miR-142-3p; hsa-miR-16-5p; hsa-miR-20a-5p; hsa-miR-24-3p; hsa-miR-92b-3p; hsa-miR-29b-3p; hsa-miR-181a-5p; hsa-miR-483-5p; hsa-miR-181d-5p; hsa-miR-93-5p; hsa-miR-124-3p; hsa-miR-21-5p; hsa-miR-29a-3p; hsa-miR-26a-5p; hsa-miR-19a-3p; hsa-miR-1229-3p; hsa-miR-20b-5p; hsa-miR-940; hsa-miR-320c; hsa-miR-19b-3p; hsa-miR-25-3p; hsa-miR-766-3p; hsa-miR-17-5p; hsa-miR-10a-5p; hsa-miR-15b-5p; hsa-miR-148b-3p; hsa-miR-149-5p; hsa-miR-23a-3p; hsa-miR-92a-3p; hsa-miR-615-3p; hsa-miR-505-3p; hsa-miR-1260b; hsa-miR-130b-5p; hsa-miR-204-5p; hsa-miR-331-3p; hsa-miR-346; hsa-miR-34b-3p; hsa-miR-30c-5p; hsa-miR-1296-5p
Pathways (miRWalk)	hsa04540 Gap junction	enriched	0.0228868	47	hsa-let-7c-5p; hsa-miR-155-5p; hsa-let-7a-5p; hsa-miR-324-3p; hsa-miR-30d-5p; hsa-miR-1-3p; hsa-miR-877-5p; hsa-miR-26b-5p; hsa-miR-320a; hsa-let-7g-5p; hsa-miR-106b-5p; hsa-miR-760; hsa-miR-146a-5p; hsa-miR-142-3p; hsa-miR-107; hsa-miR-16-5p; hsa-miR-20a-5p; hsa-miR-24-3p; hsa-miR-92b-3p; hsa-miR-365a-3p; hsa-miR-181a-5p; hsa-miR-483-5p; hsa-miR-181d-5p; hsa-miR-93-5p; hsa-miR-124-3p; hsa-miR-21-5p; hsa-miR-26a-5p; hsa-miR-1229-3p; hsa-miR-940; hsa-miR-320c; hsa-miR-19b-3p; hsa-miR-25-3p; hsa-miR-766-3p; hsa-miR-17-5p; hsa-miR-10a-5p; hsa-miR-15b-5p; hsa-miR-148b-3p; hsa-miR-663a; hsa-miR-504-5p; hsa-miR-92a-3p; hsa-miR-615-3p; hsa-miR-505-3p; hsa-miR-1226-3p; hsa-miR-331-3p; hsa-miR-197-3p; hsa-miR-30c-5p; hsa-miR-1296-5p
Pathways (miRWalk)	hsa04666 Fc gamma R mediated phagocytosis	enriched	0.0228868	55	hsa-miR-29c-3p; hsa-miR-155-5p; hsa-miR-193b-5p; hsa-let-7a-5p; hsa-miR-324-3p; hsa-miR-1-3p; hsa-miR-877-5p; hsa-miR-26b-5p; hsa-miR-320a; hsa-miR-103a-3p; hsa-miR-106b-5p; hsa-let-7i-5p; hsa-miR-451a; hsa-miR-184; hsa-miR-146a-5p; hsa-miR-142-3p; hsa-miR-107; hsa-miR-16-5p; hsa-miR-20a-5p; hsa-miR-24-3p; hsa-miR-92b-3p; hsa-miR-29b-3p; hsa-miR-181a-5p; hsa-miR-483-5p; hsa-miR-93-5p; hsa-miR-124-3p; hsa-miR-21-5p; hsa-miR-29a-3p; hsa-miR-26a-5p; hsa-miR-99b-3p; hsa-miR-2116-3p; hsa-miR-1914-3p; hsa-miR-15a-5p; hsa-miR-449c-5p; hsa-miR-940; hsa-miR-1224-5p; hsa-miR-25-3p; hsa-miR-766-3p; hsa-miR-17-5p; hsa-miR-10a-5p; hsa-miR-129-1-3p; hsa-miR-15b-5p; hsa-miR-148b-3p; hsa-miR-149-5p; hsa-miR-92a-3p; hsa-miR-615-3p; hsa-miR-505-3p; hsa-miR-1260b; hsa-miR-1226-3p; hsa-miR-331-3p; hsa-miR-346; hsa-miR-744-3p; hsa-miR-30c-5p; hsa-miR-328-3p; hsa-miR-1296-5p
Pathways (miRWalk)	hsa05120 Epithelial cell signaling in Helicobacter pylori infection	enriched	0.0228868	37	hsa-miR-29c-3p; hsa-miR-155-5p; hsa-let-7a-5p; hsa-miR-324-3p; hsa-miR-30d-5p; hsa-miR-1-3p; hsa-miR-877-5p; hsa-miR-26b-5p; hsa-miR-320a; hsa-miR-106b-5p; hsa-miR-146a-5p; hsa-miR-142-3p; hsa-miR-16-5p; hsa-miR-20a-5p; hsa-miR-24-3p; hsa-miR-29b-3p; hsa-miR-181a-5p; hsa-miR-93-5p; hsa-miR-124-3p; hsa-miR-21-5p; hsa-miR-223-3p; hsa-miR-29a-3p; hsa-miR-1914-3p; hsa-miR-15a-5p; hsa-miR-19b-3p; hsa-miR-17-5p; hsa-miR-10a-5p; hsa-miR-15b-5p; hsa-miR-149-5p; hsa-miR-23a-3p; hsa-miR-92a-3p; hsa-miR-615-3p; hsa-miR-130b-5p; hsa-miR-204-5p; hsa-miR-744-3p; hsa-miR-34b-3p; hsa-miR-30c-5p

Gene Ontology (miRWalk)	GO0008063 toll signaling pathway	enriched	0.0234237	54	hsa-miR-29c-3p; hsa-let-7c-5p; hsa-miR-155-5p; hsa-let-7a-5p; hsa-miR-324-3p; hsa-let-7f-5p; hsa-miR-30d-5p; hsa-miR-1-3p; hsa-miR-877-5p; hsa-miR-26b-5p; hsa-miR-320a; hsa-let-7g-5p; hsa-miR-103a-3p; hsa-miR-106b-5p; hsa-let-7i-5p; hsa-miR-760; hsa-miR-1287-5p; hsa-miR-146a-5p; hsa-miR-16-5p; hsa-miR-20a-5p; hsa-miR-24-3p; hsa-miR-92b-3p; hsa-miR-29b-3p; hsa-miR-181a-5p; hsa-miR-483-5p; hsa-miR-93-5p; hsa-miR-181b-5p; hsa-miR-124-3p; hsa-miR-21-5p; hsa-miR-223-3p; hsa-miR-29a-3p; hsa-miR-26a-5p; hsa-miR-19a-3p; hsa-miR-1229-3p; hsa-miR-15a-5p; hsa-miR-148a-3p; hsa-miR-19b-3p; hsa-miR-766-3p; hsa-miR-17-5p; hsa-miR-18a-5p; hsa-miR-10a-5p; hsa-miR-15b-5p; hsa-miR-149-5p; hsa-miR-663a; hsa-miR-92a-3p; hsa-miR-105-5p; hsa-miR-615-3p; hsa-miR-30b-5p; hsa-miR-1226-3p; hsa-miR-346; hsa-miR-30c-5p; hsa-miR-296-5p; hsa-miR-328-3p; hsa-miR-1296-5p
Gene Ontology (miRWalk)	GO0010212 response to ionizing radiation	enriched	0.0234237	38	hsa-miR-29c-3p; hsa-let-7c-5p; hsa-miR-155-5p; hsa-miR-193b-5p; hsa-let-7a-5p; hsa-miR-324-3p; hsa-let-7f-5p; hsa-miR-26b-5p; hsa-miR-320a; hsa-miR-146a-5p; hsa-miR-107; hsa-miR-16-5p; hsa-miR-20a-5p; hsa-miR-24-3p; hsa-miR-92b-3p; hsa-miR-29b-3p; hsa-miR-671-5p; hsa-miR-181a-5p; hsa-miR-93-5p; hsa-miR-181b-5p; hsa-miR-124-3p; hsa-miR-21-5p; hsa-miR-29a-3p; hsa-miR-26a-5p; hsa-miR-99b-3p; hsa-miR-15a-5p; hsa-miR-148a-3p; hsa-miR-19b-3p; hsa-miR-766-3p; hsa-miR-17-5p; hsa-miR-18a-5p; hsa-miR-15b-5p; hsa-miR-92a-3p; hsa-miR-615-3p; hsa-miR-505-3p; hsa-miR-30b-5p; hsa-miR-130b-5p; hsa-miR-328-3p
Gene Ontology (miRWalk)	GO0030136 clathrin coated vesicle	enriched	0.0234237	21	hsa-miR-155-5p; hsa-miR-324-3p; hsa-miR-1-3p; hsa-miR-26b-5p; hsa-miR-320a; hsa-miR-106b-5p; hsa-let-7i-5p; hsa-miR-16-5p; hsa-miR-24-3p; hsa-miR-181d-5p; hsa-miR-93-5p; hsa-miR-124-3p; hsa-miR-21-5p; hsa-miR-223-3p; hsa-miR-29a-3p; hsa-miR-26a-5p; hsa-miR-19b-3p; hsa-miR-10a-5p; hsa-miR-92a-3p; hsa-miR-615-3p; hsa-miR-4326
Gene Ontology (miRWalk)	GO0032494 response to peptidoglycan	enriched	0.0234237	8	hsa-miR-155-5p; hsa-let-7a-5p; hsa-miR-1-3p; hsa-miR-26b-5p; hsa-miR-16-5p; hsa-miR-365a-3p; hsa-miR-124-3p; hsa-miR-26a-5p
Gene Ontology (miRWalk)	GO0035094 response to nicotine	enriched	0.0234237	29	hsa-miR-29c-3p; hsa-miR-155-5p; hsa-let-7a-5p; hsa-let-7f-5p; hsa-miR-1-3p; hsa-miR-26b-5p; hsa-let-7g-5p; hsa-miR-103a-3p; hsa-let-7i-5p; hsa-miR-451a; hsa-miR-16-5p; hsa-miR-20a-5p; hsa-miR-29b-3p; hsa-miR-365a-3p; hsa-miR-181a-5p; hsa-miR-181d-5p; hsa-miR-181b-5p; hsa-miR-124-3p; hsa-miR-21-5p; hsa-miR-29a-3p; hsa-miR-15a-5p; hsa-miR-148a-3p; hsa-miR-17-5p; hsa-miR-630; hsa-miR-15b-5p; hsa-miR-148b-3p; hsa-miR-30b-5p; hsa-miR-204-5p; hsa-miR-296-5p
Gene Ontology (miRWalk)	GO0035988 chondrocyte proliferation	enriched	0.0234237	8	hsa-let-7c-5p; hsa-let-7a-5p; hsa-let-7f-5p; hsa-miR-26b-5p; hsa-let-7g-5p; hsa-miR-760; hsa-miR-21-5p; hsa-miR-26a-5p
Gene Ontology (miRWalk)	GO0043542 endothelial cell migration	enriched	0.0234237	26	hsa-let-7c-5p; hsa-miR-155-5p; hsa-miR-1-3p; hsa-miR-26b-5p; hsa-miR-103a-3p; hsa-miR-106b-5p; hsa-miR-146a-5p; hsa-miR-107; hsa-miR-20a-5p; hsa-miR-24-3p; hsa-miR-92b-3p; hsa-miR-29b-3p; hsa-miR-93-5p; hsa-miR-181b-5p; hsa-miR-124-3p; hsa-miR-21-5p; hsa-miR-29a-3p; hsa-miR-26a-5p; hsa-miR-19a-3p; hsa-miR-19b-3p; hsa-miR-494-3p; hsa-miR-17-5p; hsa-miR-18a-5p; hsa-miR-148b-3p; hsa-miR-23a-3p; hsa-miR-615-3p
Gene Ontology (miRWalk)	GO0060736 prostate gland growth	enriched	0.0234237	26	hsa-miR-29c-3p; hsa-miR-324-3p; hsa-let-7f-5p; hsa-miR-26b-5p; hsa-miR-103a-3p; hsa-miR-106b-5p; hsa-miR-107; hsa-miR-16-5p; hsa-miR-20a-5p; hsa-miR-24-3p; hsa-miR-29b-3p; hsa-miR-181a-5p; hsa-miR-93-5p; hsa-miR-181b-5p; hsa-miR-124-3p; hsa-miR-21-5p; hsa-miR-29a-3p; hsa-miR-26a-5p; hsa-miR-19a-3p; hsa-miR-19b-3p; hsa-miR-494-3p; hsa-miR-17-5p; hsa-miR-18a-5p; hsa-miR-149-5p; hsa-miR-23a-3p; hsa-miR-615-3p

Gene Ontology (miRWalk)	GO0071300 cellular response to retinoic acid	enriched	0.0234237	26	hsa-miR-29c-3p; hsa-let-7c-5p; hsa-miR-155-5p; hsa-miR-324-3p; hsa-miR-1-3p; hsa-miR-26b-5p; hsa-miR-320a; hsa-miR-103a-3p; hsa-miR-16-5p; hsa-miR-24-3p; hsa-miR-29b-3p; hsa-miR-671-5p; hsa-miR-181a-5p; hsa-miR-93-5p; hsa-miR-124-3p; hsa-miR-21-5p; hsa-miR-29a-3p; hsa-miR-26a-5p; hsa-miR-19a-3p; hsa-miR-15a-5p; hsa-miR-940; hsa-miR-10a-5p; hsa-miR-92a-3p; hsa-miR-615-3p; hsa-miR-197-3p; hsa-miR-346
Diseases (miRWalk)	Glioblastoma	enriched	0.0234483	15	hsa-let-7a-5p; hsa-miR-199b-5p; hsa-miR-451a; hsa-miR-20a-5p; hsa-miR-24-3p; hsa-miR-181a-5p; hsa-miR-483-5p; hsa-miR-93-5p; hsa-miR-124-3p; hsa-miR-21-5p; hsa-miR-26a-5p; hsa-miR-19a-3p; hsa-miR-17-5p; hsa-miR-18a-5p; hsa-miR-92a-3p
Diseases (miRWalk)	Leukemia Myeloid Acute	enriched	0.0234483	9	hsa-miR-155-5p; hsa-miR-320a; hsa-miR-24-3p; hsa-miR-29b-3p; hsa-miR-181b-5p; hsa-miR-124-3p; hsa-miR-223-3p; hsa-miR-29a-3p; hsa-miR-204-5p
Diseases (miRWalk)	Lymphoma B-Cell	enriched	0.0234483	9	hsa-miR-29c-3p; hsa-miR-155-5p; hsa-let-7a-5p; hsa-miR-20a-5p; hsa-miR-29b-3p; hsa-miR-181a-5p; hsa-miR-21-5p; hsa-miR-29a-3p; hsa-miR-17-5p
Pathways (miRWalk)	WP422 MAPK Cascade	enriched	0.0234999	30	hsa-miR-29c-3p; hsa-let-7c-5p; hsa-miR-155-5p; hsa-let-7a-5p; hsa-miR-1-3p; hsa-miR-26b-5p; hsa-miR-320a; hsa-let-7g-5p; hsa-miR-16-5p; hsa-miR-20a-5p; hsa-miR-24-3p; hsa-miR-92b-3p; hsa-miR-181a-5p; hsa-miR-483-5p; hsa-miR-181d-5p; hsa-miR-93-5p; hsa-miR-124-3p; hsa-miR-21-5p; hsa-miR-26a-5p; hsa-miR-15a-5p; hsa-miR-766-3p; hsa-miR-17-5p; hsa-miR-15b-5p; hsa-miR-148b-3p; hsa-miR-149-5p; hsa-miR-92a-3p; hsa-miR-615-3p; hsa-miR-331-3p; hsa-miR-197-3p; hsa-miR-30c-5p
Pathways (miRWalk)	WP1545 miRNAs involved in DDR	enriched	0.0236151	53	hsa-miR-29c-3p; hsa-let-7c-5p; hsa-miR-155-5p; hsa-let-7a-5p; hsa-let-7f-5p; hsa-miR-30d-5p; hsa-miR-1-3p; hsa-miR-26b-5p; hsa-miR-320a; hsa-let-7g-5p; hsa-miR-103a-3p; hsa-miR-106b-5p; hsa-let-7i-5p; hsa-miR-451a; hsa-miR-146a-5p; hsa-miR-107; hsa-miR-16-5p; hsa-miR-20a-5p; hsa-miR-24-3p; hsa-miR-29b-3p; hsa-miR-365a-3p; hsa-miR-181a-5p; hsa-miR-93-5p; hsa-miR-181b-5p; hsa-miR-124-3p; hsa-miR-21-5p; hsa-miR-223-3p; hsa-miR-29a-3p; hsa-miR-26a-5p; hsa-miR-19a-3p; hsa-miR-1229-3p; hsa-miR-15a-5p; hsa-miR-449c-5p; hsa-miR-520a-3p; hsa-miR-20b-5p; hsa-miR-19b-3p; hsa-miR-25-3p; hsa-miR-17-5p; hsa-miR-18a-5p; hsa-miR-572; hsa-miR-10a-5p; hsa-miR-15b-5p; hsa-miR-149-5p; hsa-miR-504-5p; hsa-miR-92a-3p; hsa-miR-299-5p; hsa-miR-615-3p; hsa-miR-1260b; hsa-miR-331-3p; hsa-miR-34b-3p; hsa-miR-605-5p; hsa-miR-296-5p; hsa-miR-328-3p
Pathways (miRWalk)	hsa04120 Ubiquitin mediated proteolysis	enriched	0.0237921	66	hsa-miR-29c-3p; hsa-let-7c-5p; hsa-miR-155-5p; hsa-let-7a-5p; hsa-miR-324-3p; hsa-miR-1-3p; hsa-miR-877-5p; hsa-miR-26b-5p; hsa-miR-320a; hsa-let-7g-5p; hsa-miR-103a-3p; hsa-miR-1227-3p; hsa-miR-106b-5p; hsa-miR-18b-5p; hsa-let-7i-5p; hsa-miR-760; hsa-miR-451a; hsa-miR-146a-5p; hsa-miR-142-3p; hsa-miR-107; hsa-miR-16-5p; hsa-miR-20a-5p; hsa-miR-24-3p; hsa-miR-92b-3p; hsa-miR-671-5p; hsa-miR-181a-5p; hsa-miR-181d-5p; hsa-miR-93-5p; hsa-miR-181b-5p; hsa-miR-124-3p; hsa-miR-21-5p; hsa-miR-223-3p; hsa-miR-29a-3p; hsa-miR-26a-5p; hsa-miR-99b-3p; hsa-miR-19a-3p; hsa-miR-1229-3p; hsa-miR-188-5p; hsa-miR-15a-5p; hsa-miR-19b-3p; hsa-miR-25-3p; hsa-miR-766-3p; hsa-miR-17-5p; hsa-miR-18a-5p; hsa-miR-10a-5p; hsa-miR-15b-5p; hsa-miR-148b-3p; hsa-miR-149-5p; hsa-miR-504-5p; hsa-miR-129-2-3p; hsa-miR-26b-3p; hsa-miR-92a-3p; hsa-miR-615-3p; hsa-miR-521; hsa-miR-505-3p; hsa-miR-30b-5p; hsa-miR-130b-5p; hsa-miR-204-5p; hsa-miR-1226-3p; hsa-miR-331-3p; hsa-miR-197-3p; hsa-miR-30c-5p; hsa-miR-605-5p; hsa-miR-4326; hsa-miR-328-3p; hsa-miR-1296-5p

Gene Ontology (miRWalk)	GO0003148 outflow tract septum morphogenesis	enriched	0.0240684	11	hsa-miR-1-3p; hsa-miR-26b-5p; hsa-miR-320a; hsa-miR-20a-5p; hsa-miR-92b-3p; hsa-miR-181a-5p; hsa-miR-93-5p; hsa-miR-181b-5p; hsa-miR-124-3p; hsa-miR-26a-5p; hsa-miR-615-3p
Gene Ontology (miRWalk)	GO0006636 unsaturated fatty acid biosynthetic process	enriched	0.0240684	6	hsa-miR-155-5p; hsa-let-7a-5p; hsa-miR-324-3p; hsa-miR-1-3p; hsa-miR-26b-5p; hsa-miR-19b-3p
Gene Ontology (miRWalk)	GO0010564 regulation of cell cycle process	enriched	0.0240684	11	hsa-let-7c-5p; hsa-let-7a-5p; hsa-let-7f-5p; hsa-miR-1-3p; hsa-let-7g-5p; hsa-miR-103a-3p; hsa-miR-760; hsa-miR-16-5p; hsa-miR-124-3p; hsa-miR-26a-5p; hsa-miR-92a-3p
Gene Ontology (miRWalk)	GO0060252 positive regulation of glial cell proliferation	enriched	0.0240684	11	hsa-miR-29c-3p; hsa-miR-155-5p; hsa-miR-26b-5p; hsa-miR-103a-3p; hsa-miR-107; hsa-miR-16-5p; hsa-miR-181a-5p; hsa-miR-93-5p; hsa-miR-181b-5p; hsa-miR-26a-5p; hsa-miR-17-5p
Pathways (miRWalk)	P00021 FGF signaling pathway	enriched	0.0247916	54	hsa-miR-29c-3p; hsa-let-7c-5p; hsa-miR-155-5p; hsa-let-7a-5p; hsa-miR-324-3p; hsa-let-7f-5p; hsa-miR-1-3p; hsa-miR-877-5p; hsa-miR-26b-5p; hsa-miR-320a; hsa-let-7g-5p; hsa-miR-103a-3p; hsa-miR-106b-5p; hsa-let-7i-5p; hsa-miR-451a; hsa-miR-184; hsa-miR-142-3p; hsa-miR-16-5p; hsa-miR-20a-5p; hsa-miR-24-3p; hsa-miR-92b-3p; hsa-miR-365a-3p; hsa-miR-181a-5p; hsa-miR-483-5p; hsa-miR-181d-5p; hsa-miR-93-5p; hsa-miR-181b-5p; hsa-miR-124-3p; hsa-miR-21-5p; hsa-miR-26a-5p; hsa-miR-1229-3p; hsa-miR-1914-3p; hsa-miR-15a-5p; hsa-miR-148a-3p; hsa-miR-766-3p; hsa-miR-17-5p; hsa-miR-10a-5p; hsa-miR-15b-5p; hsa-miR-148b-3p; hsa-miR-149-5p; hsa-miR-23a-3p; hsa-miR-92a-3p; hsa-miR-615-3p; hsa-miR-505-3p; hsa-miR-30b-5p; hsa-miR-130b-5p; hsa-miR-1910-5p; hsa-miR-331-3p; hsa-miR-1914-5p; hsa-miR-197-3p; hsa-miR-744-3p; hsa-miR-30c-5p; hsa-miR-328-3p; hsa-miR-1296-5p
Pathways (miRWalk)	WP481 Insulin Signaling	enriched	0.0247916	64	hsa-miR-29c-3p; hsa-let-7c-5p; hsa-miR-155-5p; hsa-let-7a-5p; hsa-miR-324-3p; hsa-miR-1-3p; hsa-miR-877-5p; hsa-miR-26b-5p; hsa-miR-320a; hsa-let-7g-5p; hsa-miR-103a-3p; hsa-miR-106b-5p; hsa-let-7i-5p; hsa-miR-760; hsa-miR-451a; hsa-miR-184; hsa-miR-142-3p; hsa-miR-107; hsa-miR-16-5p; hsa-miR-20a-5p; hsa-miR-24-3p; hsa-miR-92b-3p; hsa-miR-29b-3p; hsa-miR-671-5p; hsa-miR-181a-5p; hsa-miR-483-5p; hsa-miR-181d-5p; hsa-miR-93-5p; hsa-miR-181b-5p; hsa-miR-124-3p; hsa-miR-21-5p; hsa-miR-223-3p; hsa-miR-29a-3p; hsa-miR-26a-5p; hsa-miR-19a-3p; hsa-miR-1229-3p; hsa-miR-15a-5p; hsa-miR-148a-3p; hsa-miR-19b-3p; hsa-miR-494-3p; hsa-miR-25-3p; hsa-miR-766-3p; hsa-miR-17-5p; hsa-miR-18a-5p; hsa-miR-625-3p; hsa-miR-10a-5p; hsa-miR-129-1-3p; hsa-miR-15b-5p; hsa-miR-148b-3p; hsa-miR-149-5p; hsa-miR-23a-3p; hsa-miR-92a-3p; hsa-miR-615-3p; hsa-miR-505-3p; hsa-miR-1260b; hsa-miR-30b-5p; hsa-miR-130b-5p; hsa-miR-1226-3p; hsa-miR-331-3p; hsa-miR-1914-5p; hsa-miR-197-3p; hsa-miR-346; hsa-miR-30c-5p; hsa-miR-1296-5p
Pathways (miRWalk)	hsa04910 Insulin signaling pathway	enriched	0.0247916	64	hsa-miR-29c-3p; hsa-let-7c-5p; hsa-miR-155-5p; hsa-miR-193b-5p; hsa-let-7a-5p; hsa-miR-324-3p; hsa-miR-1-3p; hsa-miR-877-5p; hsa-miR-26b-5p; hsa-miR-320a; hsa-let-7g-5p; hsa-miR-103a-3p; hsa-miR-106b-5p; hsa-let-7i-5p; hsa-miR-451a; hsa-miR-184; hsa-miR-425-3p; hsa-miR-107; hsa-miR-16-5p; hsa-miR-20a-5p; hsa-miR-24-3p; hsa-miR-92b-3p; hsa-miR-29b-3p; hsa-miR-365a-3p; hsa-miR-181a-5p; hsa-miR-483-5p; hsa-miR-181d-5p; hsa-miR-93-5p; hsa-miR-181b-5p; hsa-miR-124-3p; hsa-miR-21-5p; hsa-miR-223-3p; hsa-miR-29a-3p; hsa-miR-26a-5p; hsa-miR-19a-3p; hsa-miR-1229-3p; hsa-miR-15a-5p; hsa-miR-1268a; hsa-miR-148a-3p; hsa-miR-940; hsa-miR-19b-3p; hsa-miR-25-3p; hsa-miR-766-3p; hsa-miR-17-5p; hsa-miR-18a-5p; hsa-miR-10a-5p; hsa-miR-129-1-3p; hsa-miR-15b-5p; hsa-miR-148b-3p; hsa-miR-149-5p; hsa-miR-23a-3p; hsa-miR-92a-3p; hsa-miR-615-3p; hsa-miR-505-3p; hsa-miR-1260b; hsa-miR-

					30b-5p; hsa-miR-130b-5p; hsa-miR-204-5p; hsa-miR-331-3p; hsa-miR-1914-5p; hsa-miR-197-3p; hsa-miR-346; hsa-miR-30c-5p; hsa-miR-1296-5p
Gene Ontology (miRWalk)	GO0000012 single strand break repair	enriched	0.0248021	10	hsa-let-7c-5p; hsa-miR-1-3p; hsa-miR-26b-5p; hsa-miR-320a; hsa-miR-16-5p; hsa-miR-92b-3p; hsa-miR-181a-5p; hsa-miR-181b-5p; hsa-miR-124-3p; hsa-miR-331-3p
Gene Ontology (miRWalk)	GO0002062 chondrocyte differentiation	enriched	0.0248021	23	hsa-let-7c-5p; hsa-let-7a-5p; hsa-let-7f-5p; hsa-miR-877-5p; hsa-miR-26b-5p; hsa-miR-320a; hsa-let-7g-5p; hsa-miR-760; hsa-miR-16-5p; hsa-miR-20a-5p; hsa-miR-24-3p; hsa-miR-93-5p; hsa-miR-181b-5p; hsa-miR-124-3p; hsa-miR-21-5p; hsa-miR-223-3p; hsa-miR-26a-5p; hsa-miR-148a-3p; hsa-miR-19b-3p; hsa-miR-17-5p; hsa-miR-10a-5p; hsa-miR-92a-3p; hsa-miR-505-3p
Gene Ontology (miRWalk)	GO0004716 receptor signaling protein tyrosine kinase activity	enriched	0.0248021	14	hsa-miR-155-5p; hsa-let-7a-5p; hsa-miR-1-3p; hsa-miR-26b-5p; hsa-miR-199b-5p; hsa-miR-146a-5p; hsa-miR-548d-3p; hsa-miR-16-5p; hsa-miR-124-3p; hsa-miR-21-5p; hsa-miR-99b-3p; hsa-miR-19a-3p; hsa-miR-615-3p; hsa-miR-331-3p
Gene Ontology (miRWalk)	GO0007165 signal transduction	enriched	0.0248021	87	hsa-miR-29c-3p; hsa-miR-22-5p; hsa-let-7c-5p; hsa-miR-155-5p; hsa-let-7a-5p; hsa-miR-584-5p; hsa-miR-324-3p; hsa-let-7f-5p; hsa-miR-30d-5p; hsa-miR-1-3p; hsa-miR-877-5p; hsa-miR-26b-5p; hsa-miR-320a; hsa-let-7g-5p; hsa-miR-103a-3p; hsa-miR-1250-5p; hsa-miR-199b-5p; hsa-miR-106b-5p; hsa-miR-18b-5p; hsa-let-7i-5p; hsa-miR-760; hsa-miR-451a; hsa-miR-184; hsa-miR-146a-5p; hsa-miR-548d-3p; hsa-miR-142-3p; hsa-miR-425-3p; hsa-miR-107; hsa-miR-16-5p; hsa-miR-20a-5p; hsa-miR-24-3p; hsa-miR-92b-3p; hsa-miR-29b-3p; hsa-miR-671-5p; hsa-miR-939-5p; hsa-miR-365a-3p; hsa-miR-181a-5p; hsa-miR-181d-5p; hsa-miR-93-5p; hsa-miR-181b-5p; hsa-miR-124-3p; hsa-miR-4284; hsa-miR-21-5p; hsa-miR-223-3p; hsa-miR-29a-3p; hsa-miR-26a-5p; hsa-miR-99b-3p; hsa-miR-19a-3p; hsa-miR-1229-3p; hsa-miR-1914-3p; hsa-miR-15a-5p; hsa-miR-20b-5p; hsa-miR-148a-3p; hsa-miR-19b-3p; hsa-miR-486-5p; hsa-miR-25-3p; hsa-miR-766-3p; hsa-miR-17-5p; hsa-miR-18a-5p; hsa-miR-10a-5p; hsa-miR-15b-5p; hsa-miR-148b-3p; hsa-miR-149-5p; hsa-miR-23a-3p; hsa-miR-504-5p; hsa-miR-26b-3p; hsa-miR-92a-3p; hsa-miR-105-5p; hsa-miR-615-3p; hsa-miR-505-3p; hsa-miR-1260b; hsa-miR-30b-5p; hsa-miR-130b-5p; hsa-miR-204-5p; hsa-miR-337-3p; hsa-miR-1226-3p; hsa-miR-331-3p; hsa-miR-1914-5p; hsa-miR-197-3p; hsa-miR-346; hsa-miR-744-3p; hsa-miR-34b-3p; hsa-miR-30c-5p; hsa-miR-605-5p; hsa-miR-296-5p; hsa-miR-328-3p; hsa-miR-1296-5p
Gene Ontology (miRWalk)	GO0009396 folic acid containing compound biosynthetic process	enriched	0.0248021	12	hsa-miR-29c-3p; hsa-miR-155-5p; hsa-let-7a-5p; hsa-miR-30d-5p; hsa-miR-1-3p; hsa-miR-877-5p; hsa-miR-26b-5p; hsa-miR-106b-5p; hsa-miR-16-5p; hsa-miR-124-3p; hsa-miR-92a-3p; hsa-miR-615-3p

Gene Ontology (miRWalk)	GO0034097 response to cytokine stimulus	enriched	0.0248021	49	hsa-miR-29c-3p; hsa-let-7c-5p; hsa-miR-155-5p; hsa-let-7a-5p; hsa-miR-324-3p; hsa-miR-30d-5p; hsa-miR-1-3p; hsa-miR-876-3p; hsa-miR-26b-5p; hsa-miR-320a; hsa-let-7g-5p; hsa-miR-103a-3p; hsa-let-7i-5p; hsa-miR-451a; hsa-miR-146a-5p; hsa-miR-107; hsa-miR-16-5p; hsa-miR-20a-5p; hsa-miR-24-3p; hsa-miR-92b-3p; hsa-miR-29b-3p; hsa-miR-365a-3p; hsa-miR-181a-5p; hsa-miR-181d-5p; hsa-miR-93-5p; hsa-miR-181b-5p; hsa-miR-124-3p; hsa-miR-21-5p; hsa-miR-29a-3p; hsa-miR-26a-5p; hsa-miR-15a-5p; hsa-miR-148a-3p; hsa-miR-17-5p; hsa-miR-630; hsa-miR-10a-5p; hsa-miR-15b-5p; hsa-miR-149-5p; hsa-miR-663a; hsa-miR-23a-3p; hsa-miR-92a-3p; hsa-miR-3679-3p; hsa-miR-615-3p; hsa-miR-204-5p; hsa-miR-1226-3p; hsa-miR-331-3p; hsa-miR-346; hsa-miR-30c-5p; hsa-miR-296-5p; hsa-miR-1296-5p
Gene Ontology (miRWalk)	GO0035198 mirna binding	enriched	0.0248021	18	hsa-miR-29c-3p; hsa-let-7c-5p; hsa-miR-155-5p; hsa-let-7a-5p; hsa-miR-26b-5p; hsa-miR-103a-3p; hsa-miR-107; hsa-miR-16-5p; hsa-miR-24-3p; hsa-miR-154-5p; hsa-miR-124-3p; hsa-miR-21-5p; hsa-miR-223-3p; hsa-miR-29a-3p; hsa-miR-18a-5p; hsa-miR-92a-3p; hsa-miR-197-3p; hsa-miR-34b-3p
Gene Ontology (miRWalk)	GO0035872 nucleotide binding domain leucine rich repeat containing receptor signaling pathway	enriched	0.0248021	51	hsa-miR-29c-3p; hsa-let-7c-5p; hsa-miR-155-5p; hsa-let-7a-5p; hsa-miR-324-3p; hsa-let-7f-5p; hsa-miR-30d-5p; hsa-miR-1-3p; hsa-miR-877-5p; hsa-miR-26b-5p; hsa-miR-320a; hsa-let-7g-5p; hsa-miR-103a-3p; hsa-miR-106b-5p; hsa-miR-760; hsa-miR-1287-5p; hsa-miR-451a; hsa-miR-146a-5p; hsa-miR-16-5p; hsa-miR-20a-5p; hsa-miR-24-3p; hsa-miR-29b-3p; hsa-miR-365a-3p; hsa-miR-181a-5p; hsa-miR-181d-5p; hsa-miR-181b-5p; hsa-miR-124-3p; hsa-miR-21-5p; hsa-miR-223-3p; hsa-miR-29a-3p; hsa-miR-26a-5p; hsa-miR-15a-5p; hsa-miR-148a-3p; hsa-miR-3620-3p; hsa-miR-17-5p; hsa-miR-18a-5p; hsa-miR-630; hsa-miR-10a-5p; hsa-miR-15b-5p; hsa-miR-148b-3p; hsa-miR-149-5p; hsa-miR-92a-3p; hsa-miR-615-3p; hsa-miR-1260b; hsa-miR-30b-5p; hsa-miR-204-5p; hsa-miR-1226-3p; hsa-miR-197-3p; hsa-miR-30c-5p; hsa-miR-296-5p; hsa-miR-328-3p
Gene Ontology (miRWalk)	GO0042552 myelination	enriched	0.0248021	20	hsa-let-7c-5p; hsa-miR-155-5p; hsa-let-7a-5p; hsa-let-7f-5p; hsa-miR-1-3p; hsa-miR-26b-5p; hsa-miR-320a; hsa-miR-106b-5p; hsa-miR-760; hsa-miR-16-5p; hsa-miR-24-3p; hsa-miR-92b-3p; hsa-miR-93-5p; hsa-miR-124-3p; hsa-miR-21-5p; hsa-miR-15a-5p; hsa-miR-15b-5p; hsa-miR-92a-3p; hsa-miR-615-3p; hsa-miR-331-3p
Gene Ontology (miRWalk)	GO0071636 positive regulation of transforming growth factor beta production	enriched	0.0248021	10	hsa-let-7c-5p; hsa-let-7a-5p; hsa-miR-26b-5p; hsa-miR-320a; hsa-miR-106b-5p; hsa-miR-16-5p; hsa-miR-24-3p; hsa-miR-181a-5p; hsa-miR-124-3p; hsa-miR-17-5p
Gene Ontology (miRWalk)	GO0000982 rna polymerase ii core promoter proximal region sequence specific dna binding transcription factor activity	enriched	0.0250495	10	hsa-miR-26b-5p; hsa-miR-320a; hsa-miR-103a-3p; hsa-miR-106b-5p; hsa-miR-146a-5p; hsa-miR-142-3p; hsa-miR-16-5p; hsa-miR-124-3p; hsa-miR-21-5p; hsa-miR-494-3p

Gene Ontology (miRWalk)	GO0002755 myd88 dependent toll like receptor signaling pathway	enriched	0.0250495	55	hsa-miR-29c-3p; hsa-let-7c-5p; hsa-miR-155-5p; hsa-let-7a-5p; hsa-miR-324-3p; hsa-let-7f-5p; hsa-miR-30d-5p; hsa-miR-1-3p; hsa-miR-877-5p; hsa-miR-26b-5p; hsa-miR-320a; hsa-let-7g-5p; hsa-miR-103a-3p; hsa-miR-106b-5p; hsa-let-7i-5p; hsa-miR-760; hsa-miR-1287-5p; hsa-miR-146a-5p; hsa-miR-16-5p; hsa-miR-20a-5p; hsa-miR-24-3p; hsa-miR-92b-3p; hsa-miR-29b-3p; hsa-miR-181a-5p; hsa-miR-483-5p; hsa-miR-93-5p; hsa-miR-181b-5p; hsa-miR-124-3p; hsa-miR-21-5p; hsa-miR-223-3p; hsa-miR-29a-3p; hsa-miR-26a-5p; hsa-miR-19a-3p; hsa-miR-1229-3p; hsa-miR-15a-5p; hsa-miR-148a-3p; hsa-miR-19b-3p; hsa-miR-766-3p; hsa-miR-17-5p; hsa-miR-18a-5p; hsa-miR-10a-5p; hsa-miR-15b-5p; hsa-miR-149-5p; hsa-miR-663a; hsa-miR-92a-3p; hsa-miR-105-5p; hsa-miR-615-3p; hsa-miR-30b-5p; hsa-miR-1226-3p; hsa-miR-331-3p; hsa-miR-197-3p; hsa-miR-346; hsa-miR-30c-5p; hsa-miR-328-3p; hsa-miR-1296-5p
Gene Ontology (miRWalk)	GO0003009 skeletal muscle contraction	enriched	0.0250495	7	hsa-miR-1-3p; hsa-miR-877-5p; hsa-miR-103a-3p; hsa-let-7i-5p; hsa-miR-146a-5p; hsa-miR-16-5p; hsa-miR-21-5p
Gene Ontology (miRWalk)	GO0004252 serine type endopeptidase activity	enriched	0.0250495	26	hsa-let-7c-5p; hsa-miR-155-5p; hsa-let-7f-5p; hsa-miR-1-3p; hsa-miR-877-5p; hsa-miR-26b-5p; hsa-miR-320a; hsa-miR-103a-3p; hsa-miR-451a; hsa-miR-142-3p; hsa-miR-107; hsa-miR-16-5p; hsa-miR-24-3p; hsa-miR-29b-3p; hsa-miR-181a-5p; hsa-miR-93-5p; hsa-miR-124-3p; hsa-miR-21-5p; hsa-miR-25-3p; hsa-miR-17-5p; hsa-miR-10a-5p; hsa-miR-148b-3p; hsa-miR-615-3p; hsa-miR-204-5p; hsa-miR-197-3p; hsa-miR-1296-5p
Gene Ontology (miRWalk)	GO0004497 monooxygenase activity	enriched	0.0250495	10	hsa-let-7a-5p; hsa-miR-1-3p; hsa-miR-26b-5p; hsa-miR-320a; hsa-miR-103a-3p; hsa-miR-107; hsa-miR-24-3p; hsa-miR-124-3p; hsa-miR-21-5p; hsa-miR-92a-3p
Gene Ontology (miRWalk)	GO0005006 epidermal growth factor activated receptor activity	enriched	0.0250495	10	hsa-miR-155-5p; hsa-let-7a-5p; hsa-miR-1-3p; hsa-miR-199b-5p; hsa-miR-146a-5p; hsa-miR-548d-3p; hsa-miR-16-5p; hsa-miR-124-3p; hsa-miR-21-5p; hsa-miR-331-3p
Gene Ontology (miRWalk)	GO0005351 sugarhydrogen symporter activity	enriched	0.0250495	10	hsa-let-7c-5p; hsa-let-7a-5p; hsa-miR-1-3p; hsa-miR-26b-5p; hsa-miR-103a-3p; hsa-miR-16-5p; hsa-miR-124-3p; hsa-miR-21-5p; hsa-miR-26a-5p; hsa-miR-15a-5p
Gene Ontology (miRWalk)	GO0006359 regulation of transcription from rna polymerase iii promoter	enriched	0.0250495	10	hsa-miR-26b-5p; hsa-miR-146a-5p; hsa-miR-16-5p; hsa-miR-24-3p; hsa-miR-181a-5p; hsa-miR-93-5p; hsa-miR-124-3p; hsa-miR-21-5p; hsa-miR-99b-3p; hsa-miR-15a-5p
Gene Ontology (miRWalk)	GO0006959 humoral immune response	enriched	0.0250495	43	hsa-miR-29c-3p; hsa-let-7c-5p; hsa-miR-155-5p; hsa-let-7a-5p; hsa-miR-324-3p; hsa-miR-1-3p; hsa-miR-26b-5p; hsa-miR-103a-3p; hsa-miR-106b-5p; hsa-miR-760; hsa-miR-451a; hsa-miR-142-3p; hsa-miR-107; hsa-miR-16-5p; hsa-miR-20a-5p; hsa-miR-24-3p; hsa-miR-29b-3p; hsa-miR-671-5p; hsa-miR-365a-3p; hsa-miR-181a-5p; hsa-miR-181d-5p; hsa-miR-181b-5p; hsa-miR-124-3p; hsa-miR-21-5p; hsa-miR-223-3p; hsa-miR-29a-3p; hsa-miR-26a-5p; hsa-miR-1229-3p; hsa-miR-15a-5p; hsa-miR-148a-3p; hsa-miR-25-3p; hsa-miR-17-5p; hsa-miR-630; hsa-miR-15b-5p; hsa-miR-149-5p; hsa-miR-92a-3p; hsa-miR-615-3p; hsa-miR-130b-5p; hsa-miR-204-5p; hsa-miR-331-3p; hsa-miR-346; hsa-miR-296-5p; hsa-miR-1296-5p
Gene Ontology (miRWalk)	GO0007346 regulation of mitotic cell cycle	enriched	0.0250495	27	hsa-let-7c-5p; hsa-miR-155-5p; hsa-let-7a-5p; hsa-miR-1-3p; hsa-miR-877-5p; hsa-miR-26b-5p; hsa-miR-320a; hsa-miR-1227-3p; hsa-miR-106b-5p; hsa-miR-16-5p; hsa-miR-20a-5p; hsa-miR-24-3p; hsa-miR-671-5p; hsa-miR-181a-5p; hsa-miR-93-5p; hsa-miR-181b-5p; hsa-miR-124-3p; hsa-miR-21-5p; hsa-miR-26a-5p; hsa-miR-940; hsa-miR-17-5p; hsa-miR-10a-5p; hsa-miR-149-5p; hsa-miR-92a-3p; hsa-miR-615-3p; hsa-miR-331-3p; hsa-miR-197-3p

Gene Ontology (miRWalk)	GO0009750 response to fructose stimulus	enriched	0.0250495	10	hsa-let-7c-5p; hsa-miR-155-5p; hsa-let-7a-5p; hsa-miR-26b-5p; hsa-miR-320a; hsa-miR-16-5p; hsa-miR-181a-5p; hsa-miR-124-3p; hsa-miR-21-5p; hsa-miR-17-5p
Gene Ontology (miRWalk)	GO0010544 negative regulation of platelet activation	enriched	0.0250495	5	hsa-miR-155-5p; hsa-let-7a-5p; hsa-miR-1-3p; hsa-miR-26b-5p; hsa-miR-16-5p
Gene Ontology (miRWalk)	GO0016226 iron sulfur cluster assembly	enriched	0.0250495	10	hsa-miR-29c-3p; hsa-miR-155-5p; hsa-miR-26b-5p; hsa-miR-106b-5p; hsa-miR-16-5p; hsa-miR-24-3p; hsa-miR-92b-3p; hsa-miR-93-5p; hsa-miR-21-5p; hsa-miR-615-3p
Gene Ontology (miRWalk)	GO0016485 protein processing	enriched	0.0250495	27	hsa-let-7c-5p; hsa-miR-155-5p; hsa-let-7a-5p; hsa-let-7f-5p; hsa-miR-30d-5p; hsa-miR-26b-5p; hsa-miR-320a; hsa-let-7g-5p; hsa-miR-103a-3p; hsa-miR-106b-5p; hsa-miR-451a; hsa-miR-107; hsa-miR-16-5p; hsa-miR-20a-5p; hsa-miR-24-3p; hsa-miR-93-5p; hsa-miR-124-3p; hsa-miR-21-5p; hsa-miR-26a-5p; hsa-miR-449c-5p; hsa-miR-17-5p; hsa-miR-10a-5p; hsa-miR-148b-3p; hsa-miR-92a-3p; hsa-miR-615-3p; hsa-miR-331-3p; hsa-miR-34b-3p
Gene Ontology (miRWalk)	GO0019827 stem cell maintenance	enriched	0.0250495	37	hsa-miR-29c-3p; hsa-let-7c-5p; hsa-miR-155-5p; hsa-let-7a-5p; hsa-miR-1-3p; hsa-miR-877-5p; hsa-miR-26b-5p; hsa-miR-320a; hsa-miR-103a-3p; hsa-miR-106b-5p; hsa-miR-107; hsa-miR-16-5p; hsa-miR-20a-5p; hsa-miR-24-3p; hsa-miR-92b-3p; hsa-miR-29b-3p; hsa-miR-154-5p; hsa-miR-181a-5p; hsa-miR-181b-5p; hsa-miR-124-3p; hsa-miR-21-5p; hsa-miR-223-3p; hsa-miR-29a-3p; hsa-miR-26a-5p; hsa-miR-940; hsa-miR-19b-3p; hsa-miR-25-3p; hsa-miR-17-5p; hsa-miR-18a-5p; hsa-miR-10a-5p; hsa-miR-92a-3p; hsa-miR-615-3p; hsa-miR-30b-5p; hsa-miR-130b-5p; hsa-miR-1226-3p; hsa-miR-346; hsa-miR-1296-5p
Gene Ontology (miRWalk)	GO0030036 actin cytoskeleton organization	enriched	0.0250495	49	hsa-miR-29c-3p; hsa-let-7c-5p; hsa-miR-155-5p; hsa-let-7a-5p; hsa-miR-584-5p; hsa-miR-324-3p; hsa-let-7f-5p; hsa-miR-1-3p; hsa-miR-26b-5p; hsa-miR-320a; hsa-let-7g-5p; hsa-miR-103a-3p; hsa-miR-106b-5p; hsa-let-7i-5p; hsa-miR-760; hsa-miR-146a-5p; hsa-miR-107; hsa-miR-16-5p; hsa-miR-20a-5p; hsa-miR-24-3p; hsa-miR-92b-3p; hsa-miR-29b-3p; hsa-miR-181a-5p; hsa-miR-181d-5p; hsa-miR-93-5p; hsa-miR-124-3p; hsa-miR-21-5p; hsa-miR-29a-3p; hsa-miR-26a-5p; hsa-miR-99b-3p; hsa-miR-1229-3p; hsa-miR-940; hsa-miR-320c; hsa-miR-1224-5p; hsa-miR-19b-3p; hsa-miR-766-3p; hsa-miR-17-5p; hsa-miR-18a-5p; hsa-miR-10a-5p; hsa-miR-148b-3p; hsa-miR-149-5p; hsa-miR-92a-3p; hsa-miR-615-3p; hsa-miR-30b-5p; hsa-miR-130b-5p; hsa-miR-331-3p; hsa-miR-346; hsa-miR-30c-5p; hsa-miR-328-3p
Gene Ontology (miRWalk)	GO0030307 positive regulation of cell growth	enriched	0.0250495	40	hsa-miR-29c-3p; hsa-let-7c-5p; hsa-miR-155-5p; hsa-let-7a-5p; hsa-miR-324-3p; hsa-miR-1-3p; hsa-miR-26b-5p; hsa-miR-320a; hsa-miR-103a-3p; hsa-miR-199b-5p; hsa-miR-451a; hsa-miR-146a-5p; hsa-miR-548d-3p; hsa-miR-142-3p; hsa-miR-16-5p; hsa-miR-20a-5p; hsa-miR-92b-3p; hsa-miR-29b-3p; hsa-miR-365a-3p; hsa-miR-181a-5p; hsa-miR-181d-5p; hsa-miR-181b-5p; hsa-miR-124-3p; hsa-miR-21-5p; hsa-miR-29a-3p; hsa-miR-15a-5p; hsa-miR-148a-3p; hsa-miR-25-3p; hsa-miR-17-5p; hsa-miR-630; hsa-miR-15b-5p; hsa-miR-92a-3p; hsa-miR-629-3p; hsa-miR-1260b; hsa-miR-130b-5p; hsa-miR-204-5p; hsa-miR-331-3p; hsa-miR-197-3p; hsa-miR-296-5p; hsa-miR-1296-5p
Gene Ontology (miRWalk)	GO0035265 organ growth	enriched	0.0250495	31	hsa-miR-29c-3p; hsa-miR-155-5p; hsa-let-7a-5p; hsa-miR-1-3p; hsa-miR-26b-5p; hsa-miR-320a; hsa-miR-103a-3p; hsa-miR-451a; hsa-miR-107; hsa-miR-16-5p; hsa-miR-20a-5p; hsa-miR-29b-3p; hsa-miR-365a-3p; hsa-miR-181a-5p; hsa-miR-181d-5p; hsa-miR-93-5p; hsa-miR-181b-5p; hsa-miR-124-3p; hsa-miR-21-5p; hsa-miR-29a-3p; hsa-miR-26a-5p; hsa-miR-15a-5p; hsa-miR-148a-3p; hsa-miR-19b-3p; hsa-miR-17-5p; hsa-miR-630; hsa-miR-15b-5p; hsa-miR-23a-3p; hsa-miR-130b-5p; hsa-miR-204-5p; hsa-miR-296-5p

Gene Ontology (miRWalk)	GO0042482 positive regulation of odontogenesis	enriched	0.0250495	7	hsa-miR-155-5p; hsa-miR-1-3p; hsa-miR-26b-5p; hsa-miR-106b-5p; hsa-miR-24-3p; hsa-miR-124-3p; hsa-miR-21-5p
Gene Ontology (miRWalk)	GO0045792 negative regulation of cell size	enriched	0.0250495	21	hsa-miR-26b-5p; hsa-miR-320a; hsa-miR-103a-3p; hsa-miR-106b-5p; hsa-miR-451a; hsa-miR-107; hsa-miR-16-5p; hsa-miR-20a-5p; hsa-miR-29b-3p; hsa-miR-93-5p; hsa-miR-181b-5p; hsa-miR-124-3p; hsa-miR-21-5p; hsa-miR-29a-3p; hsa-miR-26a-5p; hsa-miR-19a-3p; hsa-miR-19b-3p; hsa-miR-494-3p; hsa-miR-17-5p; hsa-miR-18a-5p; hsa-miR-23a-3p
Gene Ontology (miRWalk)	GO0046907 intracellular transport	enriched	0.0250495	21	hsa-miR-155-5p; hsa-let-7a-5p; hsa-miR-324-3p; hsa-let-7f-5p; hsa-miR-1-3p; hsa-miR-26b-5p; hsa-miR-320a; hsa-miR-103a-3p; hsa-miR-451a; hsa-miR-142-3p; hsa-miR-16-5p; hsa-miR-24-3p; hsa-miR-181a-5p; hsa-miR-124-3p; hsa-miR-21-5p; hsa-miR-19a-3p; hsa-miR-148a-3p; hsa-miR-17-5p; hsa-miR-92a-3p; hsa-miR-615-3p; hsa-miR-331-3p
Gene Ontology (miRWalk)	GO0048536 spleen development	enriched	0.0250495	38	hsa-miR-29c-3p; hsa-let-7c-5p; hsa-miR-155-5p; hsa-let-7a-5p; hsa-miR-1-3p; hsa-miR-26b-5p; hsa-miR-103a-3p; hsa-miR-451a; hsa-miR-146a-5p; hsa-miR-107; hsa-miR-16-5p; hsa-miR-20a-5p; hsa-miR-24-3p; hsa-miR-29b-3p; hsa-miR-154-5p; hsa-miR-365a-3p; hsa-miR-181a-5p; hsa-miR-181d-5p; hsa-miR-93-5p; hsa-miR-181b-5p; hsa-miR-124-3p; hsa-miR-21-5p; hsa-miR-29a-3p; hsa-miR-26a-5p; hsa-miR-19a-3p; hsa-miR-15a-5p; hsa-miR-148a-3p; hsa-miR-19b-3p; hsa-miR-494-3p; hsa-miR-25-3p; hsa-miR-17-5p; hsa-miR-18a-5p; hsa-miR-630; hsa-miR-15b-5p; hsa-miR-504-5p; hsa-miR-615-3p; hsa-miR-204-5p; hsa-miR-296-5p
Gene Ontology (miRWalk)	GO0048661 positive regulation of smooth muscle cell proliferation	enriched	0.0250495	35	hsa-miR-29c-3p; hsa-let-7c-5p; hsa-miR-155-5p; hsa-let-7a-5p; hsa-miR-324-3p; hsa-let-7f-5p; hsa-miR-1-3p; hsa-miR-877-5p; hsa-miR-26b-5p; hsa-miR-320a; hsa-let-7g-5p; hsa-miR-103a-3p; hsa-miR-199b-5p; hsa-let-7i-5p; hsa-miR-146a-5p; hsa-miR-107; hsa-miR-16-5p; hsa-miR-20a-5p; hsa-miR-365a-3p; hsa-miR-181a-5p; hsa-miR-93-5p; hsa-miR-124-3p; hsa-miR-26a-5p; hsa-miR-1229-3p; hsa-miR-15a-5p; hsa-miR-20b-5p; hsa-miR-17-5p; hsa-miR-18a-5p; hsa-miR-148b-3p; hsa-miR-149-5p; hsa-miR-23a-3p; hsa-miR-92a-3p; hsa-miR-615-3p; hsa-miR-505-3p; hsa-miR-30b-5p
Gene Ontology (miRWalk)	GO0050679 positive regulation of epithelial cell proliferation	enriched	0.0250495	49	hsa-miR-29c-3p; hsa-let-7c-5p; hsa-miR-155-5p; hsa-let-7a-5p; hsa-miR-324-3p; hsa-let-7f-5p; hsa-miR-1-3p; hsa-miR-877-5p; hsa-miR-26b-5p; hsa-miR-320a; hsa-let-7g-5p; hsa-miR-199b-5p; hsa-miR-106b-5p; hsa-miR-760; hsa-miR-451a; hsa-miR-146a-5p; hsa-miR-548d-3p; hsa-miR-107; hsa-miR-16-5p; hsa-miR-20a-5p; hsa-miR-24-3p; hsa-miR-29b-3p; hsa-miR-181a-5p; hsa-miR-181d-5p; hsa-miR-93-5p; hsa-miR-124-3p; hsa-miR-21-5p; hsa-miR-26a-5p; hsa-miR-134-5p; hsa-miR-19a-3p; hsa-miR-1229-3p; hsa-miR-15a-5p; hsa-miR-449c-5p; hsa-miR-20b-5p; hsa-miR-148a-3p; hsa-miR-19b-3p; hsa-miR-520g-3p; hsa-miR-25-3p; hsa-miR-17-5p; hsa-miR-10a-5p; hsa-miR-15b-5p; hsa-miR-148b-3p; hsa-miR-504-5p; hsa-miR-92a-3p; hsa-miR-615-3p; hsa-miR-1226-3p; hsa-miR-331-3p; hsa-miR-197-3p; hsa-miR-34b-3p
Gene Ontology (miRWalk)	GO0050681 androgen receptor binding	enriched	0.0250495	44	hsa-miR-22-5p; hsa-miR-155-5p; hsa-miR-1-3p; hsa-miR-26b-5p; hsa-miR-320a; hsa-miR-103a-3p; hsa-miR-1250-5p; hsa-miR-106b-5p; hsa-miR-18b-5p; hsa-miR-146a-5p; hsa-miR-425-3p; hsa-miR-107; hsa-miR-16-5p; hsa-miR-20a-5p; hsa-miR-24-3p; hsa-miR-92b-3p; hsa-miR-29b-3p; hsa-miR-365a-3p; hsa-miR-181a-5p; hsa-miR-181d-5p; hsa-miR-93-5p; hsa-miR-181b-5p; hsa-miR-124-3p; hsa-miR-21-5p; hsa-miR-29a-3p; hsa-miR-26a-5p; hsa-miR-99b-3p; hsa-miR-19a-3p; hsa-miR-15a-5p; hsa-miR-19b-3p; hsa-miR-25-3p; hsa-miR-766-3p; hsa-miR-17-5p; hsa-miR-18a-5p; hsa-miR-15b-5p; hsa-miR-149-5p; hsa-miR-23a-

					3p; hsa-miR-92a-3p; hsa-miR-615-3p; hsa-miR-1260b; hsa-miR-130b-5p; hsa-miR-1226-3p; hsa-miR-331-3p; hsa-miR-30c-5p
Gene Ontology (miRWalk)	GO0051101 regulation of dna binding	enriched	0.0250495	7	hsa-miR-26b-5p; hsa-miR-103a-3p; hsa-miR-106b-5p; hsa-miR-16-5p; hsa-miR-24-3p; hsa-miR-671-5p; hsa-miR-21-5p
Gene Ontology (miRWalk)	GO0051403 stress activated mapk cascade	enriched	0.0250495	51	hsa-miR-29c-3p; hsa-let-7c-5p; hsa-miR-155-5p; hsa-let-7a-5p; hsa-miR-324-3p; hsa-let-7f-5p; hsa-miR-30d-5p; hsa-miR-1-3p; hsa-miR-877-5p; hsa-miR-26b-5p; hsa-miR-320a; hsa-miR-103a-3p; hsa-miR-106b-5p; hsa-miR-760; hsa-miR-1287-5p; hsa-miR-146a-5p; hsa-miR-16-5p; hsa-miR-20a-5p; hsa-miR-24-3p; hsa-miR-92b-3p; hsa-miR-29b-3p; hsa-miR-181a-5p; hsa-miR-483-5p; hsa-miR-93-5p; hsa-miR-181b-5p; hsa-miR-124-3p; hsa-miR-21-5p; hsa-miR-223-3p; hsa-miR-29a-3p; hsa-miR-26a-5p; hsa-miR-19a-3p; hsa-miR-1229-3p; hsa-miR-15a-5p; hsa-miR-148a-3p; hsa-miR-19b-3p; hsa-miR-766-3p; hsa-miR-17-5p; hsa-miR-18a-5p; hsa-miR-10a-5p; hsa-miR-15b-5p; hsa-miR-149-5p; hsa-miR-663a; hsa-miR-92a-3p; hsa-miR-615-3p; hsa-miR-30b-5p; hsa-miR-1226-3p; hsa-miR-331-3p; hsa-miR-197-3p; hsa-miR-30c-5p; hsa-miR-328-3p; hsa-miR-1296-5p
Gene Ontology (miRWalk)	GO0051926 negative regulation of calcium ion transport	enriched	0.0250495	10	hsa-let-7c-5p; hsa-miR-155-5p; hsa-let-7a-5p; hsa-miR-26b-5p; hsa-miR-320a; hsa-miR-16-5p; hsa-miR-181a-5p; hsa-miR-124-3p; hsa-miR-21-5p; hsa-miR-17-5p
Gene Ontology (miRWalk)	GO0055037 recycling endosome	enriched	0.0250495	22	hsa-miR-155-5p; hsa-miR-324-3p; hsa-let-7f-5p; hsa-miR-1-3p; hsa-miR-26b-5p; hsa-miR-320a; hsa-miR-103a-3p; hsa-miR-106b-5p; hsa-miR-16-5p; hsa-miR-20a-5p; hsa-miR-24-3p; hsa-miR-181a-5p; hsa-miR-93-5p; hsa-miR-124-3p; hsa-miR-21-5p; hsa-miR-29a-3p; hsa-miR-19b-3p; hsa-miR-766-3p; hsa-miR-92a-3p; hsa-miR-615-3p; hsa-miR-197-3p; hsa-miR-744-3p
Gene Ontology (miRWalk)	GO0070530 k63 linked polyubiquitin binding	enriched	0.0250495	10	hsa-miR-155-5p; hsa-let-7a-5p; hsa-let-7f-5p; hsa-miR-877-5p; hsa-miR-103a-3p; hsa-miR-16-5p; hsa-miR-92b-3p; hsa-miR-181a-5p; hsa-miR-21-5p; hsa-miR-15b-5p
Gene Ontology (miRWalk)	GO0071347 cellular response to interleukin 1	enriched	0.0250495	24	hsa-miR-155-5p; hsa-let-7a-5p; hsa-miR-1-3p; hsa-miR-26b-5p; hsa-miR-320a; hsa-miR-199b-5p; hsa-miR-451a; hsa-miR-146a-5p; hsa-miR-107; hsa-miR-16-5p; hsa-miR-20a-5p; hsa-miR-24-3p; hsa-miR-181a-5p; hsa-miR-93-5p; hsa-miR-181b-5p; hsa-miR-124-3p; hsa-miR-21-5p; hsa-miR-15a-5p; hsa-miR-20b-5p; hsa-miR-25-3p; hsa-miR-17-5p; hsa-miR-18a-5p; hsa-miR-92a-3p; hsa-miR-204-5p
Gene Ontology (miRWalk)	GO2000178 negative regulation of neural precursor cell proliferation	enriched	0.0250495	7	hsa-miR-324-3p; hsa-miR-1-3p; hsa-miR-26b-5p; hsa-miR-103a-3p; hsa-miR-16-5p; hsa-miR-124-3p; hsa-miR-21-5p
Diseases (miRWalk)	Metaplasia	enriched	0.0252877	3	hsa-let-7f-5p; hsa-let-7g-5p; hsa-let-7i-5p
Diseases (miRWalk)	Recurrence	enriched	0.0252877	6	hsa-miR-451a; hsa-miR-20a-5p; hsa-miR-29b-3p; hsa-miR-124-3p; hsa-miR-21-5p; hsa-miR-26a-5p

Gene Ontology (miRWalk)	GO0007015 actin filament organization	enriched	0.0253129	38	hsa-miR-29c-3p; hsa-miR-155-5p; hsa-let-7a-5p; hsa-miR-324-3p; hsa-miR-1-3p; hsa-miR-26b-5p; hsa-miR-320a; hsa-miR-103a-3p; hsa-miR-451a; hsa-miR-184; hsa-miR-16-5p; hsa-miR-20a-5p; hsa-miR-24-3p; hsa-miR-92b-3p; hsa-miR-29b-3p; hsa-miR-671-5p; hsa-miR-365a-3p; hsa-miR-181a-5p; hsa-miR-181d-5p; hsa-miR-93-5p; hsa-miR-181b-5p; hsa-miR-124-3p; hsa-miR-21-5p; hsa-miR-29a-3p; hsa-miR-15a-5p; hsa-miR-148a-3p; hsa-miR-25-3p; hsa-miR-17-5p; hsa-miR-630; hsa-miR-10a-5p; hsa-miR-15b-5p; hsa-miR-148b-3p; hsa-miR-149-5p; hsa-miR-92a-3p; hsa-miR-615-3p; hsa-miR-1260b; hsa-miR-204-5p; hsa-miR-296-5p
Gene Ontology (miRWalk)	GO0031529 ruffle organization	enriched	0.0253129	15	hsa-miR-155-5p; hsa-miR-1-3p; hsa-miR-26b-5p; hsa-miR-103a-3p; hsa-miR-142-3p; hsa-miR-16-5p; hsa-miR-365a-3p; hsa-miR-181a-5p; hsa-miR-93-5p; hsa-miR-181b-5p; hsa-miR-124-3p; hsa-miR-21-5p; hsa-miR-10a-5p; hsa-miR-149-5p; hsa-miR-30c-5p
Gene Ontology (miRWalk)	GO0035497 camp response element binding	enriched	0.025413	18	hsa-miR-29c-3p; hsa-let-7c-5p; hsa-miR-155-5p; hsa-let-7a-5p; hsa-let-7f-5p; hsa-miR-26b-5p; hsa-let-7g-5p; hsa-miR-103a-3p; hsa-miR-760; hsa-miR-16-5p; hsa-miR-93-5p; hsa-miR-124-3p; hsa-miR-21-5p; hsa-miR-26a-5p; hsa-miR-15a-5p; hsa-miR-19b-3p; hsa-miR-149-5p; hsa-miR-92a-3p
Pathways (miRWalk)	hsa04142 Lysosome	enriched	0.0257092	42	hsa-miR-29c-3p; hsa-miR-155-5p; hsa-let-7a-5p; hsa-miR-324-3p; hsa-miR-1-3p; hsa-miR-877-5p; hsa-miR-26b-5p; hsa-miR-320a; hsa-miR-103a-3p; hsa-miR-1227-3p; hsa-miR-106b-5p; hsa-miR-760; hsa-miR-107; hsa-miR-16-5p; hsa-miR-20a-5p; hsa-miR-24-3p; hsa-miR-92b-3p; hsa-miR-365a-3p; hsa-miR-181a-5p; hsa-miR-93-5p; hsa-miR-124-3p; hsa-miR-21-5p; hsa-miR-29a-3p; hsa-miR-26a-5p; hsa-miR-19a-3p; hsa-miR-15a-5p; hsa-miR-19b-3p; hsa-miR-25-3p; hsa-miR-17-5p; hsa-miR-18a-5p; hsa-miR-15b-5p; hsa-miR-148b-3p; hsa-miR-149-5p; hsa-miR-23a-3p; hsa-miR-92a-3p; hsa-miR-615-3p; hsa-miR-505-3p; hsa-miR-204-5p; hsa-miR-331-3p; hsa-miR-30c-5p; hsa-miR-4326; hsa-miR-1296-5p
Pathways (miRWalk)	hsa05210 Colorectal cancer	enriched	0.0258404	64	hsa-miR-29c-3p; hsa-let-7c-5p; hsa-miR-155-5p; hsa-let-7a-5p; hsa-miR-324-3p; hsa-let-7f-5p; hsa-miR-30d-5p; hsa-miR-1-3p; hsa-miR-877-5p; hsa-miR-26b-5p; hsa-miR-320a; hsa-let-7g-5p; hsa-miR-103a-3p; hsa-miR-106b-5p; hsa-miR-451a; hsa-miR-184; hsa-miR-146a-5p; hsa-miR-142-3p; hsa-miR-16-5p; hsa-miR-20a-5p; hsa-miR-24-3p; hsa-miR-92b-3p; hsa-miR-29b-3p; hsa-miR-365a-3p; hsa-miR-181a-5p; hsa-miR-483-5p; hsa-miR-181d-5p; hsa-miR-93-5p; hsa-miR-181b-5p; hsa-miR-124-3p; hsa-miR-21-5p; hsa-miR-223-3p; hsa-miR-29a-3p; hsa-miR-26a-5p; hsa-miR-19a-3p; hsa-miR-15a-5p; hsa-miR-449c-5p; hsa-miR-148a-3p; hsa-miR-940; hsa-miR-320c; hsa-miR-19b-3p; hsa-miR-25-3p; hsa-miR-766-3p; hsa-miR-17-5p; hsa-miR-18a-5p; hsa-miR-630; hsa-miR-10a-5p; hsa-miR-15b-5p; hsa-miR-148b-3p; hsa-miR-149-5p; hsa-miR-504-5p; hsa-miR-92a-3p; hsa-miR-615-3p; hsa-miR-483-3p; hsa-miR-204-5p; hsa-miR-1226-3p; hsa-miR-331-3p; hsa-miR-197-3p; hsa-miR-346; hsa-miR-34b-3p; hsa-miR-30c-5p; hsa-miR-605-5p; hsa-miR-296-5p; hsa-miR-1296-5p
Organs (miRWalk)	B-Lymphocytes	enriched	0.0261812	14	hsa-miR-29c-3p; hsa-miR-155-5p; hsa-let-7a-5p; hsa-miR-107; hsa-miR-16-5p; hsa-miR-20a-5p; hsa-miR-29b-3p; hsa-miR-181a-5p; hsa-miR-181b-5p; hsa-miR-21-5p; hsa-miR-29a-3p; hsa-miR-15a-5p; hsa-miR-17-5p; hsa-miR-34b-3p
Organs (miRWalk)	Neoplastic Stem Cells	enriched	0.0261812	9	hsa-let-7a-5p; hsa-miR-199b-5p; hsa-miR-16-5p; hsa-miR-181a-5p; hsa-miR-181d-5p; hsa-miR-181b-5p; hsa-miR-124-3p; hsa-miR-21-5p; hsa-miR-34b-3p

Gene Ontology (miRWalk)	GO0001541 ovarian follicle development	enriched	0.0265366	49	hsa-miR-29c-3p; hsa-let-7c-5p; hsa-miR-155-5p; hsa-let-7a-5p; hsa-miR-324-3p; hsa-miR-1-3p; hsa-miR-877-5p; hsa-miR-26b-5p; hsa-miR-320a; hsa-let-7g-5p; hsa-miR-103a-3p; hsa-miR-106b-5p; hsa-miR-760; hsa-miR-451a; hsa-miR-107; hsa-miR-16-5p; hsa-miR-20a-5p; hsa-miR-24-3p; hsa-miR-92b-3p; hsa-miR-29b-3p; hsa-miR-365a-3p; hsa-miR-181a-5p; hsa-miR-181d-5p; hsa-miR-93-5p; hsa-miR-181b-5p; hsa-miR-124-3p; hsa-miR-21-5p; hsa-miR-29a-3p; hsa-miR-26a-5p; hsa-miR-134-5p; hsa-miR-15a-5p; hsa-miR-20b-5p; hsa-miR-148a-3p; hsa-miR-940; hsa-miR-19b-3p; hsa-miR-520g-3p; hsa-miR-17-5p; hsa-miR-630; hsa-miR-15b-5p; hsa-miR-148b-3p; hsa-miR-23a-3p; hsa-miR-504-5p; hsa-miR-92a-3p; hsa-miR-615-3p; hsa-miR-204-5p; hsa-miR-1236-3p; hsa-miR-197-3p; hsa-miR-34b-3p; hsa-miR-296-5p
Gene Ontology (miRWalk)	GO0001706 endoderm formation	enriched	0.0265366	7	hsa-miR-1-3p; hsa-miR-26b-5p; hsa-miR-106b-5p; hsa-miR-29b-3p; hsa-miR-181a-5p; hsa-miR-124-3p; hsa-miR-29a-3p
Gene Ontology (miRWalk)	GO0001843 neural tube closure	enriched	0.0265366	35	hsa-let-7c-5p; hsa-miR-155-5p; hsa-let-7a-5p; hsa-miR-324-3p; hsa-miR-1-3p; hsa-miR-26b-5p; hsa-miR-320a; hsa-miR-103a-3p; hsa-miR-106b-5p; hsa-miR-18b-5p; hsa-miR-146a-5p; hsa-miR-142-3p; hsa-miR-16-5p; hsa-miR-20a-5p; hsa-miR-92b-3p; hsa-miR-181a-5p; hsa-miR-93-5p; hsa-miR-181b-5p; hsa-miR-124-3p; hsa-miR-21-5p; hsa-miR-29a-3p; hsa-miR-26a-5p; hsa-miR-99b-3p; hsa-miR-940; hsa-miR-19b-3p; hsa-miR-25-3p; hsa-miR-766-3p; hsa-miR-17-5p; hsa-miR-149-5p; hsa-miR-92a-3p; hsa-miR-615-3p; hsa-miR-331-3p; hsa-miR-346; hsa-miR-30c-5p; hsa-miR-296-5p
Gene Ontology (miRWalk)	GO0014911 positive regulation of smooth muscle cell migration	enriched	0.0265366	26	hsa-miR-29c-3p; hsa-let-7a-5p; hsa-miR-1-3p; hsa-miR-877-5p; hsa-miR-26b-5p; hsa-miR-103a-3p; hsa-miR-451a; hsa-miR-16-5p; hsa-miR-20a-5p; hsa-miR-29b-3p; hsa-miR-365a-3p; hsa-miR-181a-5p; hsa-miR-181d-5p; hsa-miR-93-5p; hsa-miR-181b-5p; hsa-miR-124-3p; hsa-miR-21-5p; hsa-miR-29a-3p; hsa-miR-15a-5p; hsa-miR-148a-3p; hsa-miR-17-5p; hsa-miR-630; hsa-miR-15b-5p; hsa-miR-615-3p; hsa-miR-204-5p; hsa-miR-296-5p
Gene Ontology (miRWalk)	GO0016070 rna metabolic process	enriched	0.0265366	86	hsa-miR-29c-3p; hsa-miR-22-5p; hsa-let-7c-5p; hsa-miR-155-5p; hsa-miR-193b-5p; hsa-let-7a-5p; hsa-miR-324-3p; hsa-let-7f-5p; hsa-miR-193a-5p; hsa-miR-30d-5p; hsa-miR-1-3p; hsa-miR-498; hsa-miR-877-5p; hsa-miR-26b-5p; hsa-miR-320a; hsa-let-7g-5p; hsa-miR-103a-3p; hsa-miR-1250-5p; hsa-miR-1227-3p; hsa-miR-199b-5p; hsa-miR-106b-5p; hsa-miR-3188; hsa-miR-760; hsa-miR-1287-5p; hsa-miR-451a; hsa-miR-320d; hsa-miR-1295a; hsa-miR-142-3p; hsa-miR-425-3p; hsa-miR-107; hsa-miR-16-5p; hsa-miR-20a-5p; hsa-miR-24-3p; hsa-miR-92b-3p; hsa-miR-422a; hsa-miR-29b-3p; hsa-miR-671-5p; hsa-miR-181a-5p; hsa-miR-181d-5p; hsa-miR-93-5p; hsa-miR-181b-5p; hsa-miR-124-3p; hsa-miR-21-5p; hsa-miR-29a-3p; hsa-miR-26a-5p; hsa-miR-99b-3p; hsa-miR-19a-3p; hsa-miR-1229-3p; hsa-miR-15a-5p; hsa-miR-148a-3p; hsa-miR-940; hsa-miR-320c; hsa-miR-636; hsa-miR-19b-3p; hsa-miR-25-3p; hsa-miR-766-3p; hsa-miR-17-5p; hsa-miR-576-5p; hsa-miR-18a-5p; hsa-miR-10a-5p; hsa-miR-15b-5p; hsa-miR-148b-3p; hsa-miR-149-5p; hsa-miR-3605-3p; hsa-miR-23a-3p; hsa-miR-504-5p; hsa-miR-92a-3p; hsa-miR-1225-5p; hsa-miR-615-3p; hsa-miR-185-3p; hsa-miR-1237-3p; hsa-miR-505-3p; hsa-miR-1260b; hsa-miR-30b-5p; hsa-miR-130b-5p; hsa-miR-204-5p; hsa-miR-1226-3p; hsa-miR-331-3p; hsa-miR-1914-5p; hsa-miR-1236-3p; hsa-miR-197-3p; hsa-miR-346; hsa-miR-30c-5p; hsa-miR-328-3p; hsa-miR-1296-5p; hsa-miR-196b-3p

Gene Ontology (miRWalk)	GO0030318 melanocyte differentiation	enriched	0.0265366	32	hsa-miR-29c-3p; hsa-let-7c-5p; hsa-miR-155-5p; hsa-let-7a-5p; hsa-miR-1-3p; hsa-miR-26b-5p; hsa-miR-103a-3p; hsa-miR-451a; hsa-miR-16-5p; hsa-miR-20a-5p; hsa-miR-92b-3p; hsa-miR-29b-3p; hsa-miR-365a-3p; hsa-miR-181a-5p; hsa-miR-181d-5p; hsa-miR-93-5p; hsa-miR-181b-5p; hsa-miR-124-3p; hsa-miR-21-5p; hsa-miR-223-3p; hsa-miR-29a-3p; hsa-miR-15a-5p; hsa-miR-148a-3p; hsa-miR-19b-3p; hsa-miR-17-5p; hsa-miR-630; hsa-miR-15b-5p; hsa-miR-148b-3p; hsa-miR-92a-3p; hsa-miR-30b-5p; hsa-miR-204-5p; hsa-miR-296-5p
Gene Ontology (miRWalk)	GO0045717 negative regulation of fatty acid biosynthetic process	enriched	0.0265366	18	hsa-miR-1-3p; hsa-miR-26b-5p; hsa-miR-103a-3p; hsa-miR-106b-5p; hsa-miR-146a-5p; hsa-miR-142-3p; hsa-miR-107; hsa-miR-16-5p; hsa-miR-20a-5p; hsa-miR-24-3p; hsa-miR-181a-5p; hsa-miR-124-3p; hsa-miR-21-5p; hsa-miR-99b-3p; hsa-miR-15a-5p; hsa-miR-148a-3p; hsa-miR-92a-3p; hsa-miR-615-3p
Gene Ontology (miRWalk)	GO0048546 digestive tract morphogenesis	enriched	0.0265366	32	hsa-miR-29c-3p; hsa-miR-155-5p; hsa-let-7a-5p; hsa-miR-193a-5p; hsa-miR-1-3p; hsa-miR-26b-5p; hsa-miR-103a-3p; hsa-miR-199b-5p; hsa-miR-451a; hsa-miR-146a-5p; hsa-miR-107; hsa-miR-16-5p; hsa-miR-20a-5p; hsa-miR-29b-3p; hsa-miR-365a-3p; hsa-miR-181a-5p; hsa-miR-181d-5p; hsa-miR-181b-5p; hsa-miR-124-3p; hsa-miR-21-5p; hsa-miR-29a-3p; hsa-miR-15a-5p; hsa-miR-20b-5p; hsa-miR-148a-3p; hsa-miR-17-5p; hsa-miR-18a-5p; hsa-miR-630; hsa-miR-15b-5p; hsa-miR-92a-3p; hsa-miR-204-5p; hsa-miR-346; hsa-miR-296-5p
Gene Ontology (miRWalk)	GO2000679 positive regulation of transcription regulatory region dna binding	enriched	0.0265366	9	hsa-let-7c-5p; hsa-let-7a-5p; hsa-let-7f-5p; hsa-miR-1-3p; hsa-let-7g-5p; hsa-miR-760; hsa-miR-146a-5p; hsa-miR-26a-5p; hsa-miR-92a-3p
Pathways (miRWalk)	WP107 Translation Factors	enriched	0.0265788	43	hsa-let-7c-5p; hsa-miR-155-5p; hsa-let-7a-5p; hsa-miR-324-3p; hsa-let-7f-5p; hsa-miR-1-3p; hsa-miR-877-5p; hsa-miR-26b-5p; hsa-miR-320a; hsa-let-7g-5p; hsa-miR-106b-5p; hsa-miR-760; hsa-miR-1295a; hsa-miR-16-5p; hsa-miR-20a-5p; hsa-miR-24-3p; hsa-miR-92b-3p; hsa-miR-181a-5p; hsa-miR-93-5p; hsa-miR-181b-5p; hsa-miR-124-3p; hsa-miR-21-5p; hsa-miR-29a-3p; hsa-miR-26a-5p; hsa-miR-99b-3p; hsa-miR-1229-3p; hsa-miR-320c; hsa-miR-1228-3p; hsa-miR-25-3p; hsa-miR-17-5p; hsa-miR-18a-5p; hsa-miR-10a-5p; hsa-miR-15b-5p; hsa-miR-149-5p; hsa-miR-92a-3p; hsa-miR-615-3p; hsa-miR-505-3p; hsa-miR-1260b; hsa-miR-30b-5p; hsa-miR-1226-3p; hsa-miR-331-3p; hsa-miR-197-3p; hsa-miR-30c-5p
Pathways (miRWalk)	WP15 selenium	enriched	0.0265788	45	hsa-let-7c-5p; hsa-miR-155-5p; hsa-let-7a-5p; hsa-miR-1-3p; hsa-miR-26b-5p; hsa-miR-320a; hsa-miR-103a-3p; hsa-miR-106b-5p; hsa-miR-550a-5p; hsa-miR-760; hsa-miR-146a-5p; hsa-miR-16-5p; hsa-miR-24-3p; hsa-miR-92b-3p; hsa-miR-29b-3p; hsa-miR-671-5p; hsa-miR-365a-3p; hsa-miR-181a-5p; hsa-miR-93-5p; hsa-miR-181b-5p; hsa-miR-124-3p; hsa-miR-21-5p; hsa-miR-223-3p; hsa-miR-26a-5p; hsa-miR-99b-3p; hsa-miR-19a-3p; hsa-miR-1229-3p; hsa-miR-15a-5p; hsa-miR-320c; hsa-miR-19b-3p; hsa-miR-766-3p; hsa-miR-17-5p; hsa-miR-18a-5p; hsa-miR-10a-5p; hsa-miR-15b-5p; hsa-miR-149-5p; hsa-miR-504-5p; hsa-miR-26b-3p; hsa-miR-92a-3p; hsa-miR-615-3p; hsa-miR-30b-5p; hsa-miR-204-5p; hsa-miR-331-3p; hsa-miR-197-3p; hsa-miR-30c-5p
Pathways (miRWalk)	WP22 IL 9 signaling pathway	enriched	0.0265788	25	hsa-miR-155-5p; hsa-let-7a-5p; hsa-miR-324-3p; hsa-miR-1-3p; hsa-miR-320a; hsa-miR-106b-5p; hsa-miR-146a-5p; hsa-miR-16-5p; hsa-miR-20a-5p; hsa-miR-92b-3p; hsa-miR-181a-5p; hsa-miR-483-5p; hsa-miR-93-5p; hsa-miR-124-3p; hsa-miR-21-5p; hsa-miR-29a-3p; hsa-miR-1229-3p; hsa-miR-20b-5p; hsa-miR-766-3p; hsa-miR-17-5p; hsa-miR-15b-5p; hsa-miR-92a-3p; hsa-miR-615-3p; hsa-miR-337-3p; hsa-miR-30c-5p

Pathways (miRWalk)	hsa00360 Phenylalanine metabolism	enriched	0.0265788	10	hsa-miR-155-5p; hsa-let-7a-5p; hsa-miR-1-3p; hsa-miR-320a; hsa-miR-451a; hsa-miR-16-5p; hsa-miR-181a-5p; hsa-miR-124-3p; hsa-miR-148b-3p; hsa-miR-92a-3p
Pathways (miRWalk)	hsa04630 Jak STAT signaling pathway	enriched	0.0265788	60	hsa-miR-29c-3p; hsa-let-7c-5p; hsa-miR-155-5p; hsa-let-7a-5p; hsa-miR-324-3p; hsa-let-7f-5p; hsa-miR-1-3p; hsa-miR-877-5p; hsa-miR-26b-5p; hsa-miR-320a; hsa-let-7g-5p; hsa-miR-103a-3p; hsa-miR-106b-5p; hsa-let-7i-5p; hsa-miR-451a; hsa-miR-184; hsa-miR-146a-5p; hsa-miR-16-5p; hsa-miR-20a-5p; hsa-miR-24-3p; hsa-miR-92b-3p; hsa-miR-29b-3p; hsa-miR-365a-3p; hsa-miR-181a-5p; hsa-miR-181d-5p; hsa-miR-93-5p; hsa-miR-181b-5p; hsa-miR-124-3p; hsa-miR-21-5p; hsa-miR-223-3p; hsa-miR-29a-3p; hsa-miR-26a-5p; hsa-miR-19a-3p; hsa-miR-1229-3p; hsa-miR-15a-5p; hsa-miR-449c-5p; hsa-miR-20b-5p; hsa-miR-148a-3p; hsa-miR-19b-3p; hsa-miR-25-3p; hsa-miR-17-5p; hsa-miR-10a-5p; hsa-miR-15b-5p; hsa-miR-148b-3p; hsa-miR-149-5p; hsa-miR-23a-3p; hsa-miR-92a-3p; hsa-miR-615-3p; hsa-miR-30b-5p; hsa-miR-130b-5p; hsa-miR-204-5p; hsa-miR-337-3p; hsa-miR-331-3p; hsa-miR-454-5p; hsa-miR-346; hsa-miR-34b-3p; hsa-miR-211-5p; hsa-miR-30c-5p; hsa-miR-296-5p; hsa-miR-1296-5p
Pathways (miRWalk)	P00030 Hypoxia response via HIF activation	enriched	0.0265982	38	hsa-miR-29c-3p; hsa-let-7a-5p; hsa-miR-324-3p; hsa-miR-877-5p; hsa-miR-26b-5p; hsa-miR-103a-3p; hsa-miR-199b-5p; hsa-miR-106b-5p; hsa-let-7i-5p; hsa-miR-451a; hsa-miR-184; hsa-miR-107; hsa-miR-16-5p; hsa-miR-20a-5p; hsa-miR-24-3p; hsa-miR-29b-3p; hsa-miR-93-5p; hsa-miR-181b-5p; hsa-miR-124-3p; hsa-miR-21-5p; hsa-miR-29a-3p; hsa-miR-26a-5p; hsa-miR-19a-3p; hsa-miR-15a-5p; hsa-miR-20b-5p; hsa-miR-19b-3p; hsa-miR-494-3p; hsa-miR-17-5p; hsa-miR-18a-5p; hsa-miR-15b-5p; hsa-miR-148b-3p; hsa-miR-149-5p; hsa-miR-23a-3p; hsa-miR-92a-3p; hsa-miR-331-3p; hsa-miR-346; hsa-miR-30c-5p; hsa-miR-1296-5p
Pathways (miRWalk)	hsa04330 Notch signaling pathway	enriched	0.0265982	38	hsa-miR-29c-3p; hsa-let-7c-5p; hsa-miR-155-5p; hsa-miR-324-3p; hsa-miR-1-3p; hsa-miR-26b-5p; hsa-miR-320a; hsa-miR-103a-3p; hsa-miR-199b-5p; hsa-miR-106b-5p; hsa-miR-107; hsa-miR-16-5p; hsa-miR-20a-5p; hsa-miR-24-3p; hsa-miR-671-5p; hsa-miR-181a-5p; hsa-miR-181d-5p; hsa-miR-93-5p; hsa-miR-181b-5p; hsa-miR-124-3p; hsa-miR-21-5p; hsa-miR-26a-5p; hsa-miR-19a-3p; hsa-miR-940; hsa-miR-19b-3p; hsa-miR-25-3p; hsa-miR-17-5p; hsa-miR-18a-5p; hsa-miR-10a-5p; hsa-miR-149-5p; hsa-miR-23a-3p; hsa-miR-92a-3p; hsa-miR-615-3p; hsa-miR-130b-5p; hsa-miR-331-3p; hsa-miR-197-3p; hsa-miR-34b-3p; hsa-miR-30c-5p
Pathways (miRWalk)	WP673 ErbB signaling pathway	enriched	0.0268886	52	hsa-miR-29c-3p; hsa-let-7c-5p; hsa-miR-155-5p; hsa-let-7a-5p; hsa-let-7f-5p; hsa-miR-1-3p; hsa-miR-877-5p; hsa-miR-26b-5p; hsa-miR-320a; hsa-let-7g-5p; hsa-miR-103a-3p; hsa-miR-199b-5p; hsa-miR-106b-5p; hsa-miR-451a; hsa-miR-146a-5p; hsa-miR-548d-3p; hsa-miR-16-5p; hsa-miR-20a-5p; hsa-miR-24-3p; hsa-miR-92b-3p; hsa-miR-181a-5p; hsa-miR-181d-5p; hsa-miR-93-5p; hsa-miR-181b-5p; hsa-miR-124-3p; hsa-miR-21-5p; hsa-miR-29a-3p; hsa-miR-26a-5p; hsa-miR-19a-3p; hsa-miR-1229-3p; hsa-miR-1914-3p; hsa-miR-15a-5p; hsa-miR-449c-5p; hsa-miR-520a-3p; hsa-miR-20b-5p; hsa-miR-766-3p; hsa-miR-17-5p; hsa-miR-572; hsa-miR-10a-5p; hsa-miR-129-1-3p; hsa-miR-15b-5p; hsa-miR-149-5p; hsa-miR-92a-3p; hsa-miR-299-5p; hsa-miR-615-3p; hsa-miR-505-3p; hsa-miR-1260b; hsa-miR-331-3p; hsa-miR-744-3p; hsa-miR-34b-3p; hsa-miR-30c-5p; hsa-miR-296-5p

Gene Ontology (miRWalk)	GO0009791 post embryonic development	enriched	0.0269506	49	hsa-miR-29c-3p; hsa-let-7c-5p; hsa-miR-155-5p; hsa-let-7a-5p; hsa-miR-193a-5p; hsa-miR-30d-5p; hsa-miR-1-3p; hsa-miR-26b-5p; hsa-miR-320a; hsa-miR-103a-3p; hsa-miR-106b-5p; hsa-miR-451a; hsa-miR-184; hsa-miR-142-3p; hsa-miR-425-3p; hsa-miR-107; hsa-miR-16-5p; hsa-miR-20a-5p; hsa-miR-29b-3p; hsa-miR-154-5p; hsa-miR-671-5p; hsa-miR-365a-3p; hsa-miR-181a-5p; hsa-miR-181d-5p; hsa-miR-93-5p; hsa-miR-181b-5p; hsa-miR-124-3p; hsa-miR-21-5p; hsa-miR-29a-3p; hsa-miR-19a-3p; hsa-miR-15a-5p; hsa-miR-148a-3p; hsa-miR-320c; hsa-miR-19b-3p; hsa-miR-17-5p; hsa-miR-18a-5p; hsa-miR-630; hsa-miR-15b-5p; hsa-miR-148b-3p; hsa-miR-92a-3p; hsa-miR-802; hsa-miR-615-3p; hsa-miR-505-3p; hsa-miR-1260b; hsa-miR-204-5p; hsa-miR-1226-3p; hsa-miR-331-3p; hsa-miR-197-3p; hsa-miR-296-5p
Gene Ontology (miRWalk)	GO0042487 regulation of odontogenesis of dentin containing tooth	enriched	0.0269506	10	hsa-miR-29c-3p; hsa-let-7c-5p; hsa-let-7a-5p; hsa-miR-26b-5p; hsa-miR-103a-3p; hsa-miR-107; hsa-miR-16-5p; hsa-miR-154-5p; hsa-miR-29a-3p; hsa-miR-18a-5p
Pathways (miRWalk)	WP1434 Osteopontin Signaling	enriched	0.0269521	19	hsa-miR-155-5p; hsa-let-7a-5p; hsa-miR-26b-5p; hsa-miR-320a; hsa-miR-451a; hsa-miR-146a-5p; hsa-miR-16-5p; hsa-miR-92b-3p; hsa-miR-181a-5p; hsa-miR-483-5p; hsa-miR-124-3p; hsa-miR-21-5p; hsa-miR-223-3p; hsa-miR-15a-5p; hsa-miR-766-3p; hsa-miR-15b-5p; hsa-miR-92a-3p; hsa-miR-299-5p; hsa-miR-30c-5p
Pathways (miRWalk)	WP268 Notch Signaling Pathway	enriched	0.0269521	36	hsa-miR-29c-3p; hsa-let-7c-5p; hsa-miR-155-5p; hsa-miR-324-3p; hsa-miR-1-3p; hsa-miR-26b-5p; hsa-miR-320a; hsa-miR-103a-3p; hsa-miR-199b-5p; hsa-miR-106b-5p; hsa-miR-107; hsa-miR-16-5p; hsa-miR-20a-5p; hsa-miR-24-3p; hsa-miR-671-5p; hsa-miR-181a-5p; hsa-miR-93-5p; hsa-miR-181b-5p; hsa-miR-124-3p; hsa-miR-21-5p; hsa-miR-26a-5p; hsa-miR-19a-3p; hsa-miR-940; hsa-miR-19b-3p; hsa-miR-25-3p; hsa-miR-17-5p; hsa-miR-18a-5p; hsa-miR-10a-5p; hsa-miR-149-5p; hsa-miR-23a-3p; hsa-miR-92a-3p; hsa-miR-615-3p; hsa-miR-130b-5p; hsa-miR-331-3p; hsa-miR-197-3p; hsa-miR-34b-3p
Pathways (miRWalk)	WP560 TGF beta Signaling Pathway2	enriched	0.0269521	51	hsa-miR-29c-3p; hsa-let-7c-5p; hsa-miR-155-5p; hsa-let-7a-5p; hsa-miR-324-3p; hsa-miR-30d-5p; hsa-miR-1-3p; hsa-miR-26b-5p; hsa-miR-320a; hsa-miR-103a-3p; hsa-miR-106b-5p; hsa-miR-760; hsa-miR-146a-5p; hsa-miR-142-3p; hsa-miR-16-5p; hsa-miR-20a-5p; hsa-miR-24-3p; hsa-miR-92b-3p; hsa-miR-29b-3p; hsa-miR-181a-5p; hsa-miR-483-5p; hsa-miR-181d-5p; hsa-miR-93-5p; hsa-miR-181b-5p; hsa-miR-124-3p; hsa-miR-21-5p; hsa-miR-223-3p; hsa-miR-26a-5p; hsa-miR-19a-3p; hsa-miR-15a-5p; hsa-miR-20b-5p; hsa-miR-19b-3p; hsa-miR-25-3p; hsa-miR-17-5p; hsa-miR-18a-5p; hsa-miR-10a-5p; hsa-miR-15b-5p; hsa-miR-148b-3p; hsa-miR-149-5p; hsa-miR-92a-3p; hsa-miR-299-5p; hsa-miR-615-3p; hsa-miR-483-3p; hsa-miR-30b-5p; hsa-miR-130b-5p; hsa-miR-204-5p; hsa-miR-337-3p; hsa-miR-1226-3p; hsa-miR-331-3p; hsa-miR-346; hsa-miR-30c-5p
Diseases (published studies about miRNA profiles from peripheral blood)	Alzheimers Disease downregulated	enriched	0.0272233	22	hsa-miR-29c-3p; hsa-let-7c-5p; hsa-let-7a-5p; hsa-let-7f-5p; hsa-miR-26b-5p; hsa-let-7g-5p; hsa-miR-103a-3p; hsa-let-7i-5p; hsa-miR-107; hsa-miR-548d-5p; hsa-miR-29b-3p; hsa-miR-374a-3p; hsa-miR-21-5p; hsa-miR-223-3p; hsa-miR-29a-3p; hsa-miR-15a-5p; hsa-miR-148a-3p; hsa-miR-1234-3p; hsa-miR-15b-5p; hsa-miR-148b-3p; hsa-miR-504-5p; hsa-miR-6803-3p

Diseases (published studies about miRNA profiles from peripheral blood)	wilms tumor downregulated	enriched	0.0272233	40	hsa-miR-29c-3p; hsa-let-7c-5p; hsa-miR-155-5p; hsa-let-7a-5p; hsa-miR-584-5p; hsa-let-7f-5p; hsa-miR-26b-5p; hsa-let-7g-5p; hsa-miR-520d-3p; hsa-miR-103a-3p; hsa-miR-106b-5p; hsa-miR-18b-5p; hsa-let-7i-5p; hsa-miR-760; hsa-miR-548d-3p; hsa-miR-16-5p; hsa-miR-20a-5p; hsa-miR-29b-3p; hsa-miR-374a-3p; hsa-miR-181a-5p; hsa-miR-93-5p; hsa-miR-181b-5p; hsa-miR-21-5p; hsa-miR-26a-5p; hsa-miR-15a-5p; hsa-miR-20b-5p; hsa-miR-148a-3p; hsa-miR-17-5p; hsa-miR-581; hsa-miR-634; hsa-miR-18a-5p; hsa-miR-335-3p; hsa-miR-129-2-3p; hsa-miR-92a-3p; hsa-miR-640; hsa-miR-758-3p; hsa-miR-218-1-3p; hsa-let-7g-3p; hsa-miR-7-2-3p; hsa-miR-1296-5p
Gene Ontology (miRWalk)	GO0030073 insulin secretion	enriched	0.0273035	12	hsa-miR-155-5p; hsa-miR-1-3p; hsa-miR-877-5p; hsa-miR-26b-5p; hsa-miR-320a; hsa-miR-103a-3p; hsa-miR-106b-5p; hsa-miR-16-5p; hsa-miR-24-3p; hsa-miR-17-5p; hsa-miR-148b-3p; hsa-miR-331-3p
Diseases (miRWalk)	Inflammation	enriched	0.027863	21	hsa-miR-29c-3p; hsa-miR-155-5p; hsa-let-7a-5p; hsa-let-7f-5p; hsa-let-7g-5p; hsa-let-7i-5p; hsa-miR-146a-5p; hsa-miR-107; hsa-miR-29b-3p; hsa-miR-181a-5p; hsa-miR-181b-5p; hsa-miR-595; hsa-miR-21-5p; hsa-miR-223-3p; hsa-miR-19a-3p; hsa-miR-19b-3p; hsa-miR-25-3p; hsa-miR-10a-5p; hsa-miR-574-5p; hsa-miR-105-5p; hsa-miR-483-3p
Pathways (miRWalk)	WP138 Androgen receptor signaling pathway	enriched	0.0279721	76	hsa-miR-29c-3p; hsa-miR-22-5p; hsa-let-7c-5p; hsa-miR-155-5p; hsa-let-7a-5p; hsa-miR-584-5p; hsa-miR-324-3p; hsa-let-7f-5p; hsa-miR-30d-5p; hsa-miR-1-3p; hsa-miR-877-5p; hsa-miR-26b-5p; hsa-miR-320a; hsa-miR-103a-3p; hsa-miR-1250-5p; hsa-miR-106b-5p; hsa-miR-18b-5p; hsa-miR-760; hsa-miR-451a; hsa-miR-146a-5p; hsa-miR-142-3p; hsa-miR-107; hsa-miR-16-5p; hsa-miR-20a-5p; hsa-miR-24-3p; hsa-miR-92b-3p; hsa-miR-29b-3p; hsa-miR-671-5p; hsa-miR-365a-3p; hsa-miR-181a-5p; hsa-miR-181d-5p; hsa-miR-93-5p; hsa-miR-181b-5p; hsa-miR-124-3p; hsa-miR-21-5p; hsa-miR-223-3p; hsa-miR-29a-3p; hsa-miR-26a-5p; hsa-miR-99b-3p; hsa-miR-19a-3p; hsa-miR-1229-3p; hsa-miR-188-5p; hsa-miR-15a-5p; hsa-miR-520a-3p; hsa-miR-20b-5p; hsa-miR-320c; hsa-miR-19b-3p; hsa-miR-494-3p; hsa-miR-25-3p; hsa-miR-766-3p; hsa-miR-17-5p; hsa-miR-18a-5p; hsa-miR-572; hsa-miR-10a-5p; hsa-miR-15b-5p; hsa-miR-148b-3p; hsa-miR-149-5p; hsa-miR-23a-3p; hsa-miR-504-5p; hsa-miR-92a-3p; hsa-miR-299-5p; hsa-miR-615-3p; hsa-miR-483-3p; hsa-miR-1260b; hsa-miR-30b-5p; hsa-miR-130b-5p; hsa-miR-204-5p; hsa-miR-337-3p; hsa-miR-1226-3p; hsa-miR-331-3p; hsa-miR-197-3p; hsa-miR-34b-3p; hsa-miR-30c-5p; hsa-miR-605-5p; hsa-miR-296-5p; hsa-miR-328-3p
Gene Ontology (miRWalk)	GO0006298 mismatch repair	enriched	0.028398	20	hsa-miR-29c-3p; hsa-miR-155-5p; hsa-miR-324-3p; hsa-miR-193a-5p; hsa-miR-1-3p; hsa-miR-26b-5p; hsa-miR-103a-3p; hsa-miR-106b-5p; hsa-miR-16-5p; hsa-miR-24-3p; hsa-miR-92b-3p; hsa-miR-124-3p; hsa-miR-21-5p; hsa-miR-29a-3p; hsa-miR-26a-5p; hsa-miR-15a-5p; hsa-miR-18a-5p; hsa-miR-149-5p; hsa-miR-615-3p; hsa-miR-130b-5p
Gene Ontology (miRWalk)	GO0030665 clathrin coated vesicle membrane	enriched	0.028398	12	hsa-miR-155-5p; hsa-miR-324-3p; hsa-miR-1-3p; hsa-miR-26b-5p; hsa-miR-320a; hsa-miR-106b-5p; hsa-miR-92b-3p; hsa-miR-181a-5p; hsa-miR-93-5p; hsa-miR-124-3p; hsa-miR-19b-3p; hsa-miR-92a-3p
Gene Ontology (miRWalk)	GO0032228 regulation of synaptic transmission gabaergic	enriched	0.028398	12	hsa-let-7c-5p; hsa-miR-155-5p; hsa-let-7a-5p; hsa-let-7g-5p; hsa-miR-106b-5p; hsa-miR-16-5p; hsa-miR-20a-5p; hsa-miR-181a-5p; hsa-miR-181d-5p; hsa-miR-124-3p; hsa-miR-148b-3p; hsa-miR-615-3p

Pathways (miRWalk)	hsa04512 ECM receptor interaction	enriched	0.0293603	38	hsa-miR-29c-3p; hsa-let-7c-5p; hsa-miR-155-5p; hsa-let-7a-5p; hsa-miR-1-3p; hsa-miR-26b-5p; hsa-miR-320a; hsa-let-7g-5p; hsa-miR-103a-3p; hsa-miR-199b-5p; hsa-miR-106b-5p; hsa-miR-760; hsa-miR-146a-5p; hsa-miR-142-3p; hsa-miR-16-5p; hsa-miR-20a-5p; hsa-miR-29b-3p; hsa-miR-181a-5p; hsa-miR-93-5p; hsa-miR-124-3p; hsa-miR-21-5p; hsa-miR-29a-3p; hsa-miR-1229-3p; hsa-miR-25-3p; hsa-miR-17-5p; hsa-miR-10a-5p; hsa-miR-15b-5p; hsa-miR-148b-3p; hsa-miR-149-5p; hsa-miR-663a; hsa-miR-92a-3p; hsa-miR-299-5p; hsa-miR-615-3p; hsa-miR-505-3p; hsa-miR-204-5p; hsa-miR-197-3p; hsa-miR-30c-5p; hsa-miR-328-3p
Diseases (miRWalk)	Anoxia	enriched	0.0296776	11	hsa-miR-29c-3p; hsa-miR-155-5p; hsa-miR-107; hsa-miR-16-5p; hsa-miR-20a-5p; hsa-miR-29b-3p; hsa-miR-93-5p; hsa-miR-21-5p; hsa-miR-29a-3p; hsa-miR-20b-5p; hsa-miR-15b-5p
Pathways (miRWalk)	hsa00140 Steroid hormone biosynthesis	enriched	0.0298433	12	hsa-miR-155-5p; hsa-let-7f-5p; hsa-miR-1-3p; hsa-miR-26b-5p; hsa-miR-142-3p; hsa-miR-16-5p; hsa-miR-422a; hsa-miR-181a-5p; hsa-miR-124-3p; hsa-miR-19b-3p; hsa-miR-148b-3p; hsa-miR-92a-3p
Gene Ontology (miRWalk)	GO0000979 rna polymerase ii core promoter sequence specific dna binding	enriched	0.0300379	30	hsa-miR-155-5p; hsa-let-7a-5p; hsa-miR-324-3p; hsa-let-7f-5p; hsa-miR-30d-5p; hsa-miR-1-3p; hsa-miR-26b-5p; hsa-miR-320a; hsa-miR-103a-3p; hsa-miR-146a-5p; hsa-miR-16-5p; hsa-miR-20a-5p; hsa-miR-92b-3p; hsa-miR-29b-3p; hsa-miR-181a-5p; hsa-miR-181b-5p; hsa-miR-124-3p; hsa-miR-21-5p; hsa-miR-223-3p; hsa-miR-26a-5p; hsa-miR-1229-3p; hsa-miR-17-5p; hsa-miR-10a-5p; hsa-miR-148b-3p; hsa-miR-149-5p; hsa-miR-92a-3p; hsa-miR-615-3p; hsa-miR-1260b; hsa-miR-30b-5p; hsa-miR-30c-5p
Gene Ontology (miRWalk)	GO0001658 branching involved in ureteric bud morphogenesis	enriched	0.0302576	47	hsa-miR-29c-3p; hsa-let-7c-5p; hsa-miR-155-5p; hsa-let-7a-5p; hsa-miR-324-3p; hsa-let-7f-5p; hsa-miR-30d-5p; hsa-miR-1-3p; hsa-miR-26b-5p; hsa-miR-320a; hsa-let-7g-5p; hsa-miR-103a-3p; hsa-miR-106b-5p; hsa-miR-451a; hsa-miR-146a-5p; hsa-miR-16-5p; hsa-miR-20a-5p; hsa-miR-24-3p; hsa-miR-29b-3p; hsa-miR-365a-3p; hsa-miR-181a-5p; hsa-miR-181d-5p; hsa-miR-93-5p; hsa-miR-181b-5p; hsa-miR-124-3p; hsa-miR-21-5p; hsa-miR-29a-3p; hsa-miR-26a-5p; hsa-miR-19a-3p; hsa-miR-15a-5p; hsa-miR-449c-5p; hsa-miR-148a-3p; hsa-miR-19b-3p; hsa-miR-17-5p; hsa-miR-18a-5p; hsa-miR-630; hsa-miR-15b-5p; hsa-miR-148b-3p; hsa-miR-92a-3p; hsa-miR-483-3p; hsa-miR-204-5p; hsa-miR-1226-3p; hsa-miR-331-3p; hsa-miR-34b-3p; hsa-miR-30c-5p; hsa-miR-296-5p; hsa-miR-328-3p
Gene Ontology (miRWalk)	GO0016585 chromatin remodeling complex	enriched	0.0302576	27	hsa-let-7c-5p; hsa-let-7a-5p; hsa-miR-324-3p; hsa-let-7f-5p; hsa-miR-1-3p; hsa-miR-26b-5p; hsa-miR-106b-5p; hsa-miR-18b-5p; hsa-miR-16-5p; hsa-miR-20a-5p; hsa-miR-92b-3p; hsa-miR-29b-3p; hsa-miR-181a-5p; hsa-miR-93-5p; hsa-miR-181b-5p; hsa-miR-21-5p; hsa-miR-223-3p; hsa-miR-26a-5p; hsa-miR-19a-3p; hsa-miR-20b-5p; hsa-miR-19b-3p; hsa-miR-25-3p; hsa-miR-18a-5p; hsa-miR-92a-3p; hsa-miR-615-3p; hsa-miR-30b-5p; hsa-miR-1226-3p
Pathways (miRWalk)	P00020 FAS signaling pathway	enriched	0.0309313	33	hsa-miR-29c-3p; hsa-miR-155-5p; hsa-let-7a-5p; hsa-miR-30d-5p; hsa-miR-1-3p; hsa-miR-877-5p; hsa-miR-26b-5p; hsa-miR-320a; hsa-miR-106b-5p; hsa-miR-451a; hsa-miR-184; hsa-miR-146a-5p; hsa-miR-16-5p; hsa-miR-20a-5p; hsa-miR-24-3p; hsa-miR-93-5p; hsa-miR-124-3p; hsa-miR-21-5p; hsa-miR-223-3p; hsa-miR-26a-5p; hsa-miR-15a-5p; hsa-miR-17-5p; hsa-miR-18a-5p; hsa-miR-10a-5p; hsa-miR-15b-5p; hsa-miR-149-5p; hsa-miR-504-5p; hsa-miR-92a-3p; hsa-miR-615-3p; hsa-miR-1260b; hsa-miR-30b-5p; hsa-miR-346; hsa-miR-1296-5p

Pathways (miRWalk)	WP195 IL 1 signaling pathway	enriched	0.0309313	46	hsa-miR-29c-3p; hsa-miR-155-5p; hsa-let-7a-5p; hsa-let-7f-5p; hsa-miR-30d-5p; hsa-miR-1-3p; hsa-miR-877-5p; hsa-miR-26b-5p; hsa-miR-320a; hsa-let-7g-5p; hsa-miR-103a-3p; hsa-miR-106b-5p; hsa-miR-451a; hsa-miR-146a-5p; hsa-miR-16-5p; hsa-miR-20a-5p; hsa-miR-24-3p; hsa-miR-92b-3p; hsa-miR-181a-5p; hsa-miR-483-5p; hsa-miR-93-5p; hsa-miR-124-3p; hsa-miR-21-5p; hsa-miR-223-3p; hsa-miR-29a-3p; hsa-miR-26a-5p; hsa-miR-1229-3p; hsa-miR-1914-3p; hsa-miR-15a-5p; hsa-miR-186-3p; hsa-miR-1228-3p; hsa-miR-766-3p; hsa-miR-17-5p; hsa-miR-10a-5p; hsa-miR-15b-5p; hsa-miR-149-5p; hsa-miR-23a-3p; hsa-miR-92a-3p; hsa-miR-615-3p; hsa-miR-130b-5p; hsa-miR-204-5p; hsa-miR-1226-3p; hsa-miR-197-3p; hsa-miR-744-3p; hsa-miR-30c-5p; hsa-miR-328-3p
Pathways (miRWalk)	hsa00310 Lysine degradation	enriched	0.0309313	33	hsa-miR-29c-3p; hsa-let-7c-5p; hsa-miR-155-5p; hsa-let-7a-5p; hsa-miR-324-3p; hsa-let-7f-5p; hsa-miR-1-3p; hsa-miR-877-5p; hsa-miR-26b-5p; hsa-miR-320a; hsa-miR-103a-3p; hsa-miR-106b-5p; hsa-miR-760; hsa-miR-16-5p; hsa-miR-92b-3p; hsa-miR-93-5p; hsa-miR-124-3p; hsa-miR-21-5p; hsa-miR-29a-3p; hsa-miR-26a-5p; hsa-miR-19b-3p; hsa-miR-25-3p; hsa-miR-17-5p; hsa-miR-15b-5p; hsa-miR-149-5p; hsa-miR-92a-3p; hsa-miR-615-3p; hsa-miR-1260b; hsa-miR-130b-5p; hsa-miR-1226-3p; hsa-miR-331-3p; hsa-miR-197-3p; hsa-miR-328-3p
Pathways (miRWalk)	hsa04916 Melanogenesis	enriched	0.0309313	46	hsa-miR-29c-3p; hsa-let-7c-5p; hsa-miR-155-5p; hsa-let-7a-5p; hsa-miR-324-3p; hsa-miR-30d-5p; hsa-miR-1-3p; hsa-miR-877-5p; hsa-miR-26b-5p; hsa-miR-320a; hsa-let-7g-5p; hsa-miR-103a-3p; hsa-miR-142-3p; hsa-miR-16-5p; hsa-miR-20a-5p; hsa-miR-24-3p; hsa-miR-92b-3p; hsa-miR-365a-3p; hsa-miR-181a-5p; hsa-miR-483-5p; hsa-miR-181d-5p; hsa-miR-93-5p; hsa-miR-181b-5p; hsa-miR-124-3p; hsa-miR-21-5p; hsa-miR-26a-5p; hsa-miR-15a-5p; hsa-miR-940; hsa-miR-19b-3p; hsa-miR-25-3p; hsa-miR-766-3p; hsa-miR-17-5p; hsa-miR-10a-5p; hsa-miR-15b-5p; hsa-miR-148b-3p; hsa-miR-149-5p; hsa-miR-92a-3p; hsa-miR-615-3p; hsa-miR-505-3p; hsa-miR-130b-5p; hsa-miR-204-5p; hsa-miR-1226-3p; hsa-miR-331-3p; hsa-miR-197-3p; hsa-miR-30c-5p; hsa-miR-1296-5p
Pathways (miRWalk)	P00006 Apoptosis signaling pathway	enriched	0.0309775	65	hsa-miR-29c-3p; hsa-let-7c-5p; hsa-miR-155-5p; hsa-let-7a-5p; hsa-miR-324-3p; hsa-miR-30d-5p; hsa-miR-1-3p; hsa-miR-876-3p; hsa-miR-877-5p; hsa-miR-26b-5p; hsa-miR-320a; hsa-let-7g-5p; hsa-miR-103a-3p; hsa-miR-106b-5p; hsa-miR-451a; hsa-miR-184; hsa-miR-146a-5p; hsa-miR-425-3p; hsa-miR-16-5p; hsa-miR-20a-5p; hsa-miR-24-3p; hsa-miR-92b-3p; hsa-miR-29b-3p; hsa-miR-365a-3p; hsa-miR-181a-5p; hsa-miR-483-5p; hsa-miR-181d-5p; hsa-miR-93-5p; hsa-miR-181b-5p; hsa-miR-124-3p; hsa-miR-21-5p; hsa-miR-223-3p; hsa-miR-29a-3p; hsa-miR-26a-5p; hsa-miR-19a-3p; hsa-miR-15a-5p; hsa-miR-148a-3p; hsa-miR-940; hsa-miR-320c; hsa-miR-19b-3p; hsa-miR-494-3p; hsa-miR-25-3p; hsa-miR-766-3p; hsa-miR-17-5p; hsa-miR-630; hsa-miR-10a-5p; hsa-miR-15b-5p; hsa-miR-148b-3p; hsa-miR-149-5p; hsa-miR-504-5p; hsa-miR-92a-3p; hsa-miR-3679-3p; hsa-miR-615-3p; hsa-miR-505-3p; hsa-miR-130b-5p; hsa-miR-204-5p; hsa-miR-1226-3p; hsa-miR-331-3p; hsa-miR-197-3p; hsa-miR-346; hsa-miR-30c-5p; hsa-miR-605-5p; hsa-miR-296-5p; hsa-miR-328-3p; hsa-miR-1296-5p
Pathways (miRWalk)	WP78 TCA Cycle	enriched	0.0309775	14	hsa-let-7a-5p; hsa-miR-324-3p; hsa-miR-1-3p; hsa-miR-26b-5p; hsa-miR-320a; hsa-miR-760; hsa-miR-425-3p; hsa-miR-16-5p; hsa-miR-92b-3p; hsa-miR-124-3p; hsa-miR-17-5p; hsa-miR-92a-3p; hsa-miR-615-3p; hsa-miR-1260b

Pathways (miRWalk)	hsa05211 Renal cell carcinoma	enriched	0.0311765	59	hsa-miR-29c-3p; hsa-let-7c-5p; hsa-miR-155-5p; hsa-miR-193b-5p; hsa-let-7a-5p; hsa-miR-324-3p; hsa-miR-1-3p; hsa-miR-26b-5p; hsa-miR-320a; hsa-let-7g-5p; hsa-miR-103a-3p; hsa-miR-199b-5p; hsa-miR-106b-5p; hsa-let-7i-5p; hsa-miR-451a; hsa-miR-184; hsa-miR-142-3p; hsa-miR-107; hsa-miR-16-5p; hsa-miR-20a-5p; hsa-miR-24-3p; hsa-miR-92b-3p; hsa-miR-29b-3p; hsa-miR-181a-5p; hsa-miR-483-5p; hsa-miR-181d-5p; hsa-miR-93-5p; hsa-miR-181b-5p; hsa-miR-124-3p; hsa-miR-21-5p; hsa-miR-29a-3p; hsa-miR-134-5p; hsa-miR-1229-3p; hsa-miR-15a-5p; hsa-miR-20b-5p; hsa-miR-19b-3p; hsa-miR-520g-3p; hsa-miR-25-3p; hsa-miR-766-3p; hsa-miR-17-5p; hsa-miR-18a-5p; hsa-miR-10a-5p; hsa-miR-129-1-3p; hsa-miR-15b-5p; hsa-miR-148b-3p; hsa-miR-149-5p; hsa-miR-23a-3p; hsa-miR-504-5p; hsa-miR-92a-3p; hsa-miR-615-3p; hsa-miR-1260b; hsa-miR-130b-5p; hsa-miR-337-3p; hsa-miR-331-3p; hsa-miR-346; hsa-miR-34b-3p; hsa-miR-30c-5p; hsa-miR-4326; hsa-miR-1296-5p
Pathways (miRWalk)	P00044 Nicotinic acetylcholine receptor signaling pathway	enriched	0.0313213	35	hsa-let-7c-5p; hsa-miR-155-5p; hsa-let-7a-5p; hsa-miR-324-3p; hsa-miR-1-3p; hsa-miR-877-5p; hsa-miR-26b-5p; hsa-miR-320a; hsa-miR-103a-3p; hsa-miR-1227-3p; hsa-miR-1295a; hsa-miR-16-5p; hsa-miR-24-3p; hsa-miR-92b-3p; hsa-miR-181a-5p; hsa-miR-93-5p; hsa-miR-181b-5p; hsa-miR-124-3p; hsa-miR-21-5p; hsa-miR-26a-5p; hsa-miR-99b-3p; hsa-miR-15a-5p; hsa-miR-19b-3p; hsa-miR-10a-5p; hsa-miR-149-5p; hsa-miR-23a-3p; hsa-miR-92a-3p; hsa-miR-615-3p; hsa-miR-505-3p; hsa-miR-1260b; hsa-miR-30b-5p; hsa-miR-1226-3p; hsa-miR-331-3p; hsa-miR-197-3p; hsa-miR-1296-5p
Pathways (miRWalk)	WP51 Regulation of Actin Cytoskeleton	enriched	0.0313213	64	hsa-miR-29c-3p; hsa-let-7c-5p; hsa-miR-155-5p; hsa-let-7a-5p; hsa-miR-584-5p; hsa-miR-324-3p; hsa-miR-1-3p; hsa-miR-877-5p; hsa-miR-26b-5p; hsa-miR-320a; hsa-let-7g-5p; hsa-miR-103a-3p; hsa-miR-1227-3p; hsa-miR-106b-5p; hsa-miR-18b-5p; hsa-let-7i-5p; hsa-miR-146a-5p; hsa-miR-1295a; hsa-miR-142-3p; hsa-miR-107; hsa-miR-16-5p; hsa-miR-20a-5p; hsa-miR-24-3p; hsa-miR-92b-3p; hsa-miR-29b-3p; hsa-miR-181a-5p; hsa-miR-483-5p; hsa-miR-93-5p; hsa-miR-181b-5p; hsa-miR-124-3p; hsa-miR-21-5p; hsa-miR-29a-3p; hsa-miR-26a-5p; hsa-miR-99b-3p; hsa-miR-1229-3p; hsa-miR-15a-5p; hsa-miR-148a-3p; hsa-miR-940; hsa-miR-320c; hsa-miR-1224-5p; hsa-miR-19b-3p; hsa-miR-25-3p; hsa-miR-766-3p; hsa-miR-17-5p; hsa-miR-18a-5p; hsa-miR-10a-5p; hsa-miR-129-1-3p; hsa-miR-15b-5p; hsa-miR-148b-3p; hsa-miR-149-5p; hsa-miR-23a-3p; hsa-miR-92a-3p; hsa-miR-615-3p; hsa-miR-505-3p; hsa-miR-1260b; hsa-miR-30b-5p; hsa-miR-130b-5p; hsa-miR-204-5p; hsa-miR-1226-3p; hsa-miR-331-3p; hsa-miR-197-3p; hsa-miR-346; hsa-miR-30c-5p; hsa-miR-328-3p
Organs (miRWalk)	Mucus	enriched	0.0313327	3	hsa-let-7f-5p; hsa-let-7g-5p; hsa-let-7i-5p
Gene Ontology (miRWalk)	GO0002639 positive regulation of immunoglobulin production	enriched	0.0313477	6	hsa-let-7f-5p; hsa-miR-1-3p; hsa-miR-320a; hsa-let-7g-5p; hsa-let-7i-5p; hsa-miR-1229-3p

Gene Ontology (miRWalk)	GO0006412 translation	enriched	0.0313477	79	hsa-miR-22-5p; hsa-let-7c-5p; hsa-miR-155-5p; hsa-miR-193b-5p; hsa-let-7a-5p; hsa-miR-324-3p; hsa-let-7f-5p; hsa-miR-193a-5p; hsa-miR-30d-5p; hsa-miR-1-3p; hsa-miR-877-5p; hsa-miR-26b-5p; hsa-miR-320a; hsa-let-7g-5p; hsa-miR-103a-3p; hsa-miR-1250-5p; hsa-miR-1227-3p; hsa-miR-106b-5p; hsa-let-7i-5p; hsa-miR-3188; hsa-miR-760; hsa-miR-451a; hsa-miR-1295a; hsa-miR-142-3p; hsa-miR-425-3p; hsa-miR-107; hsa-miR-16-5p; hsa-miR-20a-5p; hsa-miR-24-3p; hsa-miR-92b-3p; hsa-miR-422a; hsa-miR-29b-3p; hsa-miR-671-5p; hsa-miR-365a-3p; hsa-miR-181a-5p; hsa-miR-181d-5p; hsa-miR-93-5p; hsa-miR-181b-5p; hsa-miR-124-3p; hsa-miR-21-5p; hsa-miR-29a-3p; hsa-miR-26a-5p; hsa-miR-99b-3p; hsa-miR-1229-3p; hsa-miR-148a-3p; hsa-miR-940; hsa-miR-320c; hsa-miR-636; hsa-miR-19b-3p; hsa-miR-1228-3p; hsa-miR-25-3p; hsa-miR-766-3p; hsa-miR-17-5p; hsa-miR-576-5p; hsa-miR-18a-5p; hsa-miR-10a-5p; hsa-miR-15b-5p; hsa-miR-148b-3p; hsa-miR-149-5p; hsa-miR-3605-3p; hsa-miR-23a-3p; hsa-miR-504-5p; hsa-miR-92a-3p; hsa-miR-615-3p; hsa-miR-1237-3p; hsa-miR-505-3p; hsa-miR-1260b; hsa-miR-30b-5p; hsa-miR-130b-5p; hsa-miR-204-5p; hsa-miR-1226-3p; hsa-miR-331-3p; hsa-miR-1914-5p; hsa-miR-197-3p; hsa-miR-346; hsa-miR-30c-5p; hsa-miR-328-3p; hsa-miR-1296-5p; hsa-miR-196b-3p
Gene Ontology (miRWalk)	GO0021670 lateral ventricle development	enriched	0.0313477	21	hsa-miR-29c-3p; hsa-let-7a-5p; hsa-miR-324-3p; hsa-miR-1-3p; hsa-miR-26b-5p; hsa-miR-320a; hsa-miR-103a-3p; hsa-miR-107; hsa-miR-16-5p; hsa-miR-92b-3p; hsa-miR-29b-3p; hsa-miR-124-3p; hsa-miR-21-5p; hsa-miR-29a-3p; hsa-miR-26a-5p; hsa-miR-1229-3p; hsa-miR-23a-3p; hsa-miR-615-3p; hsa-miR-505-3p; hsa-miR-197-3p; hsa-miR-34b-3p
Gene Ontology (miRWalk)	GO0045638 negative regulation of myeloid cell differentiation	enriched	0.0313477	22	hsa-miR-29c-3p; hsa-let-7c-5p; hsa-miR-155-5p; hsa-let-7a-5p; hsa-miR-26b-5p; hsa-miR-320a; hsa-miR-103a-3p; hsa-miR-107; hsa-miR-16-5p; hsa-miR-20a-5p; hsa-miR-24-3p; hsa-miR-29b-3p; hsa-miR-124-3p; hsa-miR-21-5p; hsa-miR-29a-3p; hsa-miR-26a-5p; hsa-miR-19b-3p; hsa-miR-615-3p; hsa-miR-1226-3p; hsa-miR-346; hsa-miR-34b-3p; hsa-miR-1296-5p
Gene Ontology (miRWalk)	GO0050871 positive regulation of b cell activation	enriched	0.0314005	7	hsa-miR-155-5p; hsa-let-7a-5p; hsa-miR-1-3p; hsa-miR-24-3p; hsa-miR-365a-3p; hsa-miR-124-3p; hsa-miR-26a-5p
Gene Ontology (miRWalk)	GO0001568 blood vessel development	enriched	0.0314293	33	hsa-miR-29c-3p; hsa-let-7c-5p; hsa-miR-155-5p; hsa-miR-193b-5p; hsa-let-7a-5p; hsa-miR-26b-5p; hsa-miR-320a; hsa-let-7g-5p; hsa-miR-103a-3p; hsa-miR-107; hsa-miR-16-5p; hsa-miR-20a-5p; hsa-miR-29b-3p; hsa-miR-181a-5p; hsa-miR-93-5p; hsa-miR-181b-5p; hsa-miR-124-3p; hsa-miR-21-5p; hsa-miR-223-3p; hsa-miR-29a-3p; hsa-miR-26a-5p; hsa-miR-19a-3p; hsa-miR-15a-5p; hsa-miR-19b-3p; hsa-miR-17-5p; hsa-miR-18a-5p; hsa-miR-10a-5p; hsa-miR-148b-3p; hsa-miR-92a-3p; hsa-miR-615-3p; hsa-miR-1260b; hsa-miR-204-5p; hsa-miR-1226-3p
Gene Ontology (miRWalk)	GO0001709 cell fate determination	enriched	0.0314293	28	hsa-miR-29c-3p; hsa-let-7c-5p; hsa-miR-155-5p; hsa-miR-30d-5p; hsa-miR-1-3p; hsa-miR-876-3p; hsa-miR-877-5p; hsa-miR-26b-5p; hsa-miR-320a; hsa-miR-103a-3p; hsa-miR-107; hsa-miR-16-5p; hsa-miR-29b-3p; hsa-miR-181a-5p; hsa-miR-181b-5p; hsa-miR-124-3p; hsa-miR-21-5p; hsa-miR-29a-3p; hsa-miR-26a-5p; hsa-miR-15a-5p; hsa-miR-25-3p; hsa-miR-17-5p; hsa-miR-148b-3p; hsa-miR-92a-3p; hsa-miR-3679-3p; hsa-miR-615-3p; hsa-miR-130b-5p; hsa-miR-34b-3p

Gene Ontology (miRWalk)	GO0001934 positive regulation of protein phosphorylation	enriched	0.0314293	56	hsa-miR-29c-3p; hsa-let-7c-5p; hsa-miR-155-5p; hsa-let-7a-5p; hsa-miR-324-3p; hsa-let-7f-5p; hsa-miR-1-3p; hsa-miR-877-5p; hsa-miR-26b-5p; hsa-miR-320a; hsa-let-7g-5p; hsa-miR-103a-3p; hsa-miR-199b-5p; hsa-miR-106b-5p; hsa-miR-451a; hsa-miR-146a-5p; hsa-miR-548d-3p; hsa-miR-107; hsa-miR-16-5p; hsa-miR-20a-5p; hsa-miR-24-3p; hsa-miR-29b-3p; hsa-miR-365a-3p; hsa-miR-181a-5p; hsa-miR-483-5p; hsa-miR-181d-5p; hsa-miR-93-5p; hsa-miR-181b-5p; hsa-miR-124-3p; hsa-miR-21-5p; hsa-miR-26a-5p; hsa-miR-134-5p; hsa-miR-19a-3p; hsa-miR-15a-5p; hsa-miR-20b-5p; hsa-miR-320c; hsa-miR-19b-3p; hsa-miR-486-5p; hsa-miR-520g-3p; hsa-miR-25-3p; hsa-miR-17-5p; hsa-miR-18a-5p; hsa-miR-10a-5p; hsa-miR-15b-5p; hsa-miR-149-5p; hsa-miR-504-5p; hsa-miR-92a-3p; hsa-miR-615-3p; hsa-miR-505-3p; hsa-miR-130b-5p; hsa-miR-204-5p; hsa-miR-1226-3p; hsa-miR-331-3p; hsa-miR-34b-3p; hsa-miR-30c-5p; hsa-miR-296-5p
Gene Ontology (miRWalk)	GO0004521 endoribonuclease activity	enriched	0.0314293	12	hsa-let-7a-5p; hsa-miR-30d-5p; hsa-miR-1-3p; hsa-miR-877-5p; hsa-miR-26b-5p; hsa-miR-107; hsa-miR-16-5p; hsa-miR-671-5p; hsa-miR-93-5p; hsa-miR-21-5p; hsa-miR-15a-5p; hsa-miR-331-3p
Gene Ontology (miRWalk)	GO0005095 gtpase inhibitor activity	enriched	0.0314293	14	hsa-miR-29c-3p; hsa-let-7c-5p; hsa-miR-324-3p; hsa-let-7f-5p; hsa-miR-1-3p; hsa-miR-26b-5p; hsa-miR-320a; hsa-let-7i-5p; hsa-miR-16-5p; hsa-miR-93-5p; hsa-miR-124-3p; hsa-miR-92a-3p; hsa-let-7e-3p; hsa-miR-1296-5p
Gene Ontology (miRWalk)	GO0005694 chromosome	enriched	0.0314293	30	hsa-let-7c-5p; hsa-miR-155-5p; hsa-let-7a-5p; hsa-miR-324-3p; hsa-miR-1-3p; hsa-miR-877-5p; hsa-miR-26b-5p; hsa-miR-320a; hsa-miR-103a-3p; hsa-miR-106b-5p; hsa-miR-760; hsa-miR-142-3p; hsa-miR-16-5p; hsa-miR-20a-5p; hsa-miR-24-3p; hsa-miR-92b-3p; hsa-miR-93-5p; hsa-miR-124-3p; hsa-miR-21-5p; hsa-miR-26a-5p; hsa-miR-19b-3p; hsa-miR-25-3p; hsa-miR-17-5p; hsa-miR-148b-3p; hsa-miR-149-5p; hsa-miR-23a-3p; hsa-miR-92a-3p; hsa-miR-615-3p; hsa-miR-1260b; hsa-miR-197-3p
Gene Ontology (miRWalk)	GO0005765 lysosomal membrane	enriched	0.0314293	40	hsa-let-7c-5p; hsa-miR-155-5p; hsa-let-7a-5p; hsa-miR-324-3p; hsa-miR-1-3p; hsa-miR-877-5p; hsa-miR-26b-5p; hsa-miR-320a; hsa-miR-103a-3p; hsa-miR-1227-3p; hsa-miR-106b-5p; hsa-miR-146a-5p; hsa-miR-107; hsa-miR-16-5p; hsa-miR-20a-5p; hsa-miR-24-3p; hsa-miR-92b-3p; hsa-miR-365a-3p; hsa-miR-181a-5p; hsa-miR-93-5p; hsa-miR-124-3p; hsa-miR-21-5p; hsa-miR-26a-5p; hsa-miR-2116-3p; hsa-miR-19a-3p; hsa-miR-1229-3p; hsa-miR-19b-3p; hsa-miR-17-5p; hsa-miR-18a-5p; hsa-miR-10a-5p; hsa-miR-15b-5p; hsa-miR-148b-3p; hsa-miR-149-5p; hsa-miR-23a-3p; hsa-miR-92a-3p; hsa-miR-615-3p; hsa-miR-30b-5p; hsa-miR-204-5p; hsa-miR-1226-3p; hsa-miR-331-3p
Gene Ontology (miRWalk)	GO0006337 nucleosome disassembly	enriched	0.0314293	30	hsa-let-7c-5p; hsa-miR-155-5p; hsa-let-7a-5p; hsa-miR-324-3p; hsa-let-7f-5p; hsa-miR-1-3p; hsa-miR-877-5p; hsa-miR-26b-5p; hsa-miR-320a; hsa-miR-103a-3p; hsa-miR-199b-5p; hsa-miR-16-5p; hsa-miR-92b-3p; hsa-miR-29b-3p; hsa-miR-671-5p; hsa-miR-93-5p; hsa-miR-124-3p; hsa-miR-21-5p; hsa-miR-223-3p; hsa-miR-26a-5p; hsa-miR-25-3p; hsa-miR-15b-5p; hsa-miR-92a-3p; hsa-miR-615-3p; hsa-miR-1260b; hsa-miR-30b-5p; hsa-miR-181a-2-3p; hsa-miR-331-3p; hsa-miR-30c-5p; hsa-miR-328-3p
Gene Ontology (miRWalk)	GO0006493 protein o linked glycosylation	enriched	0.0314293	15	hsa-miR-155-5p; hsa-miR-324-3p; hsa-miR-1-3p; hsa-miR-26b-5p; hsa-miR-103a-3p; hsa-miR-16-5p; hsa-miR-92b-3p; hsa-miR-671-5p; hsa-miR-181a-5p; hsa-miR-93-5p; hsa-miR-124-3p; hsa-miR-26a-5p; hsa-miR-148a-3p; hsa-miR-10a-5p; hsa-miR-92a-3p

Gene Ontology (miRWalk)	GO0006641 triglyceride metabolic process	enriched	0.0314293	22	hsa-miR-155-5p; hsa-miR-26b-5p; hsa-miR-320a; hsa-miR-103a-3p; hsa-miR-106b-5p; hsa-miR-142-3p; hsa-miR-107; hsa-miR-16-5p; hsa-miR-20a-5p; hsa-miR-92b-3p; hsa-miR-181a-5p; hsa-miR-181b-5p; hsa-miR-124-3p; hsa-miR-21-5p; hsa-miR-29a-3p; hsa-miR-99b-3p; hsa-miR-148b-3p; hsa-miR-23a-3p; hsa-miR-92a-3p; hsa-miR-30b-5p; hsa-miR-130b-5p; hsa-miR-197-3p
Gene Ontology (miRWalk)	GO0007565 female pregnancy	enriched	0.0314293	38	hsa-miR-29c-3p; hsa-miR-155-5p; hsa-let-7a-5p; hsa-miR-324-3p; hsa-miR-1-3p; hsa-miR-877-5p; hsa-miR-26b-5p; hsa-miR-320a; hsa-miR-103a-3p; hsa-miR-451a; hsa-miR-142-3p; hsa-miR-16-5p; hsa-miR-20a-5p; hsa-miR-24-3p; hsa-miR-29b-3p; hsa-miR-365a-3p; hsa-miR-181a-5p; hsa-miR-181d-5p; hsa-miR-93-5p; hsa-miR-181b-5p; hsa-miR-124-3p; hsa-miR-21-5p; hsa-miR-29a-3p; hsa-miR-26a-5p; hsa-miR-1229-3p; hsa-miR-15a-5p; hsa-miR-148a-3p; hsa-miR-17-5p; hsa-miR-630; hsa-miR-10a-5p; hsa-miR-15b-5p; hsa-miR-148b-3p; hsa-miR-92a-3p; hsa-miR-615-3p; hsa-miR-204-5p; hsa-miR-331-3p; hsa-miR-30c-5p; hsa-miR-296-5p
Gene Ontology (miRWalk)	GO0007584 response to nutrient	enriched	0.0314293	28	hsa-miR-29c-3p; hsa-miR-155-5p; hsa-let-7a-5p; hsa-miR-30d-5p; hsa-miR-1-3p; hsa-miR-877-5p; hsa-miR-26b-5p; hsa-miR-103a-3p; hsa-miR-106b-5p; hsa-miR-146a-5p; hsa-miR-16-5p; hsa-miR-24-3p; hsa-miR-92b-3p; hsa-miR-671-5p; hsa-miR-93-5p; hsa-miR-181b-5p; hsa-miR-124-3p; hsa-miR-21-5p; hsa-miR-26a-5p; hsa-miR-20b-5p; hsa-miR-25-3p; hsa-miR-17-5p; hsa-miR-148b-3p; hsa-miR-23a-3p; hsa-miR-92a-3p; hsa-miR-615-3p; hsa-miR-30b-5p; hsa-miR-331-3p
Gene Ontology (miRWalk)	GO0009952 anterior posterior pattern specification	enriched	0.0314293	39	hsa-miR-29c-3p; hsa-let-7c-5p; hsa-miR-155-5p; hsa-miR-193b-5p; hsa-let-7a-5p; hsa-miR-324-3p; hsa-miR-30d-5p; hsa-miR-877-5p; hsa-miR-26b-5p; hsa-miR-320a; hsa-miR-103a-3p; hsa-miR-106b-5p; hsa-miR-142-3p; hsa-miR-107; hsa-miR-16-5p; hsa-miR-20a-5p; hsa-miR-29b-3p; hsa-miR-181a-5p; hsa-miR-93-5p; hsa-miR-181b-5p; hsa-miR-124-3p; hsa-miR-21-5p; hsa-miR-29a-3p; hsa-miR-26a-5p; hsa-miR-19a-3p; hsa-miR-148a-3p; hsa-miR-940; hsa-miR-320c; hsa-miR-19b-3p; hsa-miR-17-5p; hsa-miR-10a-5p; hsa-miR-148b-3p; hsa-miR-149-5p; hsa-miR-92a-3p; hsa-miR-615-3p; hsa-miR-1260b; hsa-miR-204-5p; hsa-miR-331-3p; hsa-miR-1296-5p
Gene Ontology (miRWalk)	GO0016071 mrna metabolic process	enriched	0.0314293	82	hsa-miR-29c-3p; hsa-miR-22-5p; hsa-let-7c-5p; hsa-miR-155-5p; hsa-miR-193b-5p; hsa-let-7a-5p; hsa-miR-324-3p; hsa-let-7f-5p; hsa-miR-193a-5p; hsa-miR-30d-5p; hsa-miR-1-3p; hsa-miR-877-5p; hsa-miR-26b-5p; hsa-miR-320a; hsa-let-7g-5p; hsa-miR-103a-3p; hsa-miR-1250-5p; hsa-miR-1227-3p; hsa-miR-199b-5p; hsa-miR-106b-5p; hsa-miR-3188; hsa-miR-760; hsa-miR-1287-5p; hsa-miR-451a; hsa-miR-1295a; hsa-miR-142-3p; hsa-miR-425-3p; hsa-miR-107; hsa-miR-16-5p; hsa-miR-20a-5p; hsa-miR-24-3p; hsa-miR-92b-3p; hsa-miR-422a; hsa-miR-29b-3p; hsa-miR-671-5p; hsa-miR-181a-5p; hsa-miR-181d-5p; hsa-miR-93-5p; hsa-miR-181b-5p; hsa-miR-124-3p; hsa-miR-21-5p; hsa-miR-29a-3p; hsa-miR-26a-5p; hsa-miR-99b-3p; hsa-miR-1229-3p; hsa-miR-15a-5p; hsa-miR-148a-3p; hsa-miR-940; hsa-miR-320c; hsa-miR-636; hsa-miR-19b-3p; hsa-miR-25-3p; hsa-miR-766-3p; hsa-miR-17-5p; hsa-miR-576-5p; hsa-miR-18a-5p; hsa-miR-10a-5p; hsa-miR-15b-5p; hsa-miR-148b-3p; hsa-miR-149-5p; hsa-miR-3605-3p; hsa-miR-23a-3p; hsa-miR-504-5p; hsa-miR-92a-3p; hsa-miR-615-3p; hsa-miR-185-3p; hsa-miR-1237-3p; hsa-miR-505-3p; hsa-miR-1260b; hsa-miR-30b-5p; hsa-miR-130b-5p; hsa-miR-204-5p; hsa-miR-1226-3p; hsa-miR-331-3p; hsa-miR-1914-5p; hsa-miR-1236-3p; hsa-miR-197-3p; hsa-miR-346; hsa-miR-30c-5p; hsa-miR-328-3p; hsa-miR-1296-5p; hsa-miR-196b-3p

Gene Ontology (miRWalk)	GO0016604 nuclear body	enriched	0.0314293	42	hsa-miR-29c-3p; hsa-let-7c-5p; hsa-miR-155-5p; hsa-let-7a-5p; hsa-miR-324-3p; hsa-let-7f-5p; hsa-miR-30d-5p; hsa-miR-1-3p; hsa-miR-26b-5p; hsa-miR-320a; hsa-let-7g-5p; hsa-miR-103a-3p; hsa-miR-106b-5p; hsa-miR-18b-5p; hsa-miR-760; hsa-miR-451a; hsa-miR-16-5p; hsa-miR-20a-5p; hsa-miR-24-3p; hsa-miR-92b-3p; hsa-miR-671-5p; hsa-miR-181a-5p; hsa-miR-93-5p; hsa-miR-124-3p; hsa-miR-21-5p; hsa-miR-26a-5p; hsa-miR-15a-5p; hsa-miR-449c-5p; hsa-miR-19b-3p; hsa-miR-25-3p; hsa-miR-17-5p; hsa-miR-10a-5p; hsa-miR-148b-3p; hsa-miR-149-5p; hsa-miR-504-5p; hsa-miR-92a-3p; hsa-miR-615-3p; hsa-miR-30b-5p; hsa-miR-130b-5p; hsa-miR-34b-3p; hsa-miR-30c-5p; hsa-miR-605-5p
Gene Ontology (miRWalk)	GO0019079 viral genome replication	enriched	0.0314293	10	hsa-miR-155-5p; hsa-miR-1-3p; hsa-miR-26b-5p; hsa-miR-107; hsa-miR-16-5p; hsa-miR-24-3p; hsa-miR-124-3p; hsa-miR-223-3p; hsa-miR-26a-5p; hsa-miR-940
Gene Ontology (miRWalk)	GO0030332 cyclin binding	enriched	0.0314293	36	hsa-miR-29c-3p; hsa-miR-155-5p; hsa-let-7a-5p; hsa-miR-324-3p; hsa-let-7f-5p; hsa-miR-1-3p; hsa-miR-877-5p; hsa-miR-26b-5p; hsa-miR-320a; hsa-miR-103a-3p; hsa-miR-106b-5p; hsa-miR-146a-5p; hsa-miR-107; hsa-miR-16-5p; hsa-miR-20a-5p; hsa-miR-24-3p; hsa-miR-92b-3p; hsa-miR-29b-3p; hsa-miR-93-5p; hsa-miR-124-3p; hsa-miR-21-5p; hsa-miR-29a-3p; hsa-miR-26a-5p; hsa-miR-1229-3p; hsa-miR-520a-3p; hsa-miR-20b-5p; hsa-miR-17-5p; hsa-miR-18a-5p; hsa-miR-572; hsa-miR-663a; hsa-miR-92a-3p; hsa-miR-299-5p; hsa-miR-615-3p; hsa-miR-1260b; hsa-miR-34b-3p; hsa-miR-885-5p
Gene Ontology (miRWalk)	GO0031659 positive regulation of cyclin dependent protein kinase activity involved in g1 s	enriched	0.0314293	12	hsa-miR-155-5p; hsa-let-7a-5p; hsa-miR-1-3p; hsa-miR-26b-5p; hsa-miR-106b-5p; hsa-miR-451a; hsa-miR-146a-5p; hsa-miR-16-5p; hsa-miR-93-5p; hsa-miR-21-5p; hsa-miR-17-5p; hsa-miR-615-3p
Gene Ontology (miRWalk)	GO0032880 regulation of protein localization	enriched	0.0314293	37	hsa-miR-29c-3p; hsa-miR-155-5p; hsa-let-7a-5p; hsa-miR-324-3p; hsa-miR-1-3p; hsa-miR-877-5p; hsa-miR-26b-5p; hsa-miR-103a-3p; hsa-miR-18b-5p; hsa-miR-451a; hsa-miR-142-3p; hsa-miR-107; hsa-miR-16-5p; hsa-miR-20a-5p; hsa-miR-29b-3p; hsa-miR-365a-3p; hsa-miR-181a-5p; hsa-miR-181d-5p; hsa-miR-181b-5p; hsa-miR-124-3p; hsa-miR-21-5p; hsa-miR-223-3p; hsa-miR-29a-3p; hsa-miR-19a-3p; hsa-miR-15a-5p; hsa-miR-148a-3p; hsa-miR-17-5p; hsa-miR-18a-5p; hsa-miR-630; hsa-miR-10a-5p; hsa-miR-15b-5p; hsa-miR-92a-3p; hsa-miR-615-3p; hsa-miR-204-5p; hsa-miR-1226-3p; hsa-miR-197-3p; hsa-miR-296-5p
Gene Ontology (miRWalk)	GO0042127 regulation of cell proliferation	enriched	0.0314293	56	hsa-miR-29c-3p; hsa-let-7c-5p; hsa-miR-155-5p; hsa-let-7a-5p; hsa-miR-30d-5p; hsa-miR-1-3p; hsa-miR-498; hsa-miR-26b-5p; hsa-miR-320a; hsa-miR-103a-3p; hsa-miR-106b-5p; hsa-let-7i-5p; hsa-miR-550a-5p; hsa-miR-760; hsa-miR-320d; hsa-miR-146a-5p; hsa-miR-107; hsa-miR-16-5p; hsa-miR-20a-5p; hsa-miR-24-3p; hsa-miR-92b-3p; hsa-miR-29b-3p; hsa-miR-181a-5p; hsa-miR-93-5p; hsa-miR-181b-5p; hsa-miR-124-3p; hsa-miR-21-5p; hsa-miR-223-3p; hsa-miR-29a-3p; hsa-miR-26a-5p; hsa-miR-99b-3p; hsa-miR-134-5p; hsa-miR-19a-3p; hsa-miR-15a-5p; hsa-miR-148a-3p; hsa-miR-320c; hsa-miR-19b-3p; hsa-miR-25-3p; hsa-miR-766-3p; hsa-miR-17-5p; hsa-miR-18a-5p; hsa-miR-10a-5p; hsa-miR-148b-3p; hsa-miR-149-5p; hsa-miR-23a-3p; hsa-miR-504-5p; hsa-miR-92a-3p; hsa-miR-802; hsa-miR-615-3p; hsa-miR-1260b; hsa-miR-30b-5p; hsa-miR-204-5p; hsa-miR-331-3p; hsa-miR-197-3p; hsa-miR-34b-3p; hsa-miR-30c-5p
Gene Ontology (miRWalk)	GO0043022 ribosome binding	enriched	0.0314293	26	hsa-let-7c-5p; hsa-miR-155-5p; hsa-let-7a-5p; hsa-miR-1-3p; hsa-miR-877-5p; hsa-miR-26b-5p; hsa-miR-320a; hsa-miR-103a-3p; hsa-miR-1227-3p; hsa-miR-106b-5p; hsa-miR-760; hsa-miR-107; hsa-miR-16-5p; hsa-miR-92b-3p; hsa-miR-181b-5p; hsa-miR-124-3p; hsa-miR-21-5p; hsa-miR-26a-5p; hsa-miR-25-3p;

					hsa-miR-17-5p; hsa-miR-18a-5p; hsa-miR-92a-3p; hsa-miR-615-3p; hsa-miR-1226-3p; hsa-miR-331-3p; hsa-miR-30c-5p
Gene Ontology (miRWalk)	GO0043473 pigmentation	enriched	0.0314293	24	hsa-let-7c-5p; hsa-miR-155-5p; hsa-let-7a-5p; hsa-let-7f-5p; hsa-miR-1-3p; hsa-miR-320a; hsa-let-7g-5p; hsa-miR-451a; hsa-miR-16-5p; hsa-miR-20a-5p; hsa-miR-24-3p; hsa-miR-92b-3p; hsa-miR-181a-5p; hsa-miR-93-5p; hsa-miR-124-3p; hsa-miR-21-5p; hsa-miR-26a-5p; hsa-miR-449c-5p; hsa-miR-636; hsa-miR-17-5p; hsa-miR-630; hsa-miR-148b-3p; hsa-miR-204-5p; hsa-miR-34b-3p
Gene Ontology (miRWalk)	GO0043507 positive regulation of jun kinase activity	enriched	0.0314293	21	hsa-let-7c-5p; hsa-miR-155-5p; hsa-miR-324-3p; hsa-miR-1-3p; hsa-miR-26b-5p; hsa-miR-320a; hsa-miR-103a-3p; hsa-miR-146a-5p; hsa-miR-16-5p; hsa-miR-24-3p; hsa-miR-181a-5p; hsa-miR-93-5p; hsa-miR-181b-5p; hsa-miR-124-3p; hsa-miR-21-5p; hsa-miR-148a-3p; hsa-miR-10a-5p; hsa-miR-149-5p; hsa-miR-92a-3p; hsa-miR-615-3p; hsa-miR-204-5p
Gene Ontology (miRWalk)	GO0045409 negative regulation of interleukin 6 biosynthetic process	enriched	0.0314293	3	hsa-miR-155-5p; hsa-miR-26b-5p; hsa-miR-320a
Gene Ontology (miRWalk)	GO0045736 negative regulation of cyclin dependent protein kinase activity	enriched	0.0314293	36	hsa-miR-155-5p; hsa-let-7a-5p; hsa-miR-324-3p; hsa-let-7f-5p; hsa-miR-30d-5p; hsa-miR-1-3p; hsa-miR-877-5p; hsa-miR-26b-5p; hsa-miR-320a; hsa-let-7g-5p; hsa-miR-103a-3p; hsa-miR-106b-5p; hsa-miR-146a-5p; hsa-miR-16-5p; hsa-miR-20a-5p; hsa-miR-24-3p; hsa-miR-181a-5p; hsa-miR-93-5p; hsa-miR-181b-5p; hsa-miR-124-3p; hsa-miR-21-5p; hsa-miR-29a-3p; hsa-miR-19a-3p; hsa-miR-1229-3p; hsa-miR-520a-3p; hsa-miR-20b-5p; hsa-miR-148a-3p; hsa-miR-19b-3p; hsa-miR-25-3p; hsa-miR-17-5p; hsa-miR-572; hsa-miR-92a-3p; hsa-miR-299-5p; hsa-miR-615-3p; hsa-miR-1260b; hsa-miR-296-5p
Gene Ontology (miRWalk)	GO0045740 positive regulation of dna replication	enriched	0.0314293	32	hsa-miR-29c-3p; hsa-miR-155-5p; hsa-let-7a-5p; hsa-miR-1-3p; hsa-miR-26b-5p; hsa-miR-320a; hsa-miR-106b-5p; hsa-miR-146a-5p; hsa-miR-16-5p; hsa-miR-24-3p; hsa-miR-92b-3p; hsa-miR-29b-3p; hsa-miR-181a-5p; hsa-miR-181d-5p; hsa-miR-93-5p; hsa-miR-181b-5p; hsa-miR-124-3p; hsa-miR-21-5p; hsa-miR-223-3p; hsa-miR-29a-3p; hsa-miR-26a-5p; hsa-miR-15a-5p; hsa-miR-25-3p; hsa-miR-10a-5p; hsa-miR-149-5p; hsa-miR-663a; hsa-miR-92a-3p; hsa-miR-615-3p; hsa-miR-30b-5p; hsa-miR-1226-3p; hsa-miR-331-3p; hsa-miR-34b-3p
Gene Ontology (miRWalk)	GO0048666 neuron development	enriched	0.0314293	26	hsa-miR-29c-3p; hsa-let-7c-5p; hsa-miR-155-5p; hsa-miR-324-3p; hsa-miR-193a-5p; hsa-miR-30d-5p; hsa-miR-1-3p; hsa-miR-26b-5p; hsa-miR-320a; hsa-miR-103a-3p; hsa-miR-106b-5p; hsa-miR-550a-5p; hsa-miR-16-5p; hsa-miR-181a-5p; hsa-miR-21-5p; hsa-miR-223-3p; hsa-miR-29a-3p; hsa-miR-26a-5p; hsa-miR-17-5p; hsa-miR-15b-5p; hsa-miR-149-5p; hsa-miR-92a-3p; hsa-miR-615-3p; hsa-miR-331-3p; hsa-miR-197-3p; hsa-miR-346
Gene Ontology (miRWalk)	GO0050671 positive regulation of lymphocyte proliferation	enriched	0.0314293	3	hsa-miR-155-5p; hsa-miR-26b-5p; hsa-miR-320a
Gene Ontology (miRWalk)	GO0051233 spindle midzone	enriched	0.0314293	15	hsa-miR-29c-3p; hsa-miR-155-5p; hsa-miR-1-3p; hsa-miR-877-5p; hsa-miR-26b-5p; hsa-miR-103a-3p; hsa-miR-106b-5p; hsa-miR-142-3p; hsa-miR-16-5p; hsa-miR-29b-3p; hsa-miR-29a-3p; hsa-miR-26a-5p; hsa-miR-148b-3p; hsa-miR-615-3p; hsa-miR-331-3p

Gene Ontology (miRWalk)	GO0051434 bh3 domain binding	enriched	0.0314293	32	hsa-miR-29c-3p; hsa-let-7c-5p; hsa-let-7a-5p; hsa-miR-30d-5p; hsa-miR-1-3p; hsa-miR-876-3p; hsa-miR-26b-5p; hsa-miR-320a; hsa-let-7g-5p; hsa-miR-103a-3p; hsa-miR-451a; hsa-miR-16-5p; hsa-miR-20a-5p; hsa-miR-29b-3p; hsa-miR-365a-3p; hsa-miR-181a-5p; hsa-miR-181d-5p; hsa-miR-181b-5p; hsa-miR-21-5p; hsa-miR-29a-3p; hsa-miR-26a-5p; hsa-miR-15a-5p; hsa-miR-148a-3p; hsa-miR-17-5p; hsa-miR-630; hsa-miR-15b-5p; hsa-miR-148b-3p; hsa-miR-504-5p; hsa-miR-3679-3p; hsa-miR-615-3p; hsa-miR-204-5p; hsa-miR-296-5p
Gene Ontology (miRWalk)	GO0051489 regulation of filopodium assembly	enriched	0.0314293	10	hsa-miR-29c-3p; hsa-miR-155-5p; hsa-miR-1-3p; hsa-miR-103a-3p; hsa-miR-106b-5p; hsa-miR-29b-3p; hsa-miR-181a-5p; hsa-miR-29a-3p; hsa-miR-26a-5p; hsa-miR-18a-5p
Gene Ontology (miRWalk)	GO0051591 response to camp	enriched	0.0314293	36	hsa-miR-29c-3p; hsa-miR-155-5p; hsa-let-7a-5p; hsa-miR-324-3p; hsa-miR-1-3p; hsa-miR-877-5p; hsa-miR-26b-5p; hsa-miR-320a; hsa-miR-106b-5p; hsa-miR-146a-5p; hsa-miR-16-5p; hsa-miR-20a-5p; hsa-miR-24-3p; hsa-miR-92b-3p; hsa-miR-765; hsa-miR-29b-3p; hsa-miR-181a-5p; hsa-miR-93-5p; hsa-miR-181b-5p; hsa-miR-124-3p; hsa-miR-21-5p; hsa-miR-29a-3p; hsa-miR-26a-5p; hsa-miR-15a-5p; hsa-miR-19b-3p; hsa-miR-766-3p; hsa-miR-17-5p; hsa-miR-10a-5p; hsa-miR-149-5p; hsa-miR-663a; hsa-miR-92a-3p; hsa-miR-615-3p; hsa-miR-204-5p; hsa-miR-1226-3p; hsa-miR-197-3p; hsa-miR-328-3p
Gene Ontology (miRWalk)	GO0051770 positive regulation of nitric oxide synthase biosynthetic process	enriched	0.0314293	13	hsa-miR-155-5p; hsa-miR-1-3p; hsa-miR-26b-5p; hsa-miR-106b-5p; hsa-let-7i-5p; hsa-miR-146a-5p; hsa-miR-16-5p; hsa-miR-24-3p; hsa-miR-124-3p; hsa-miR-21-5p; hsa-miR-19a-3p; hsa-miR-19b-3p; hsa-miR-105-5p
Gene Ontology (miRWalk)	GO0051879 hsp90 protein binding	enriched	0.0314293	30	hsa-let-7c-5p; hsa-miR-155-5p; hsa-let-7a-5p; hsa-miR-324-3p; hsa-miR-1-3p; hsa-miR-26b-5p; hsa-miR-199b-5p; hsa-miR-106b-5p; hsa-miR-760; hsa-miR-142-3p; hsa-miR-107; hsa-miR-16-5p; hsa-miR-20a-5p; hsa-miR-24-3p; hsa-miR-29b-3p; hsa-miR-181a-5p; hsa-miR-93-5p; hsa-miR-124-3p; hsa-miR-29a-3p; hsa-miR-26a-5p; hsa-miR-20b-5p; hsa-miR-940; hsa-miR-19b-3p; hsa-miR-494-3p; hsa-miR-17-5p; hsa-miR-18a-5p; hsa-miR-10a-5p; hsa-miR-92a-3p; hsa-miR-615-3p; hsa-miR-337-3p
Gene Ontology (miRWalk)	GO0051891 positive regulation of cardioblast differentiation	enriched	0.0314293	10	hsa-miR-1-3p; hsa-miR-26b-5p; hsa-miR-20a-5p; hsa-miR-92b-3p; hsa-miR-181a-5p; hsa-miR-181b-5p; hsa-miR-124-3p; hsa-miR-21-5p; hsa-miR-26a-5p; hsa-miR-615-3p
Gene Ontology (miRWalk)	GO0055074 calcium ion homeostasis	enriched	0.0314293	12	hsa-miR-155-5p; hsa-miR-1-3p; hsa-miR-26b-5p; hsa-miR-103a-3p; hsa-miR-106b-5p; hsa-miR-16-5p; hsa-miR-20a-5p; hsa-miR-93-5p; hsa-miR-124-3p; hsa-miR-21-5p; hsa-miR-17-5p; hsa-miR-1226-3p
Gene Ontology (miRWalk)	GO0060510 type ii pneumocyte differentiation	enriched	0.0314293	12	hsa-let-7f-5p; hsa-miR-1-3p; hsa-miR-20a-5p; hsa-miR-92b-3p; hsa-miR-365a-3p; hsa-miR-181a-5p; hsa-miR-93-5p; hsa-miR-181b-5p; hsa-miR-124-3p; hsa-miR-21-5p; hsa-miR-19b-3p; hsa-miR-92a-3p
Gene Ontology (miRWalk)	GO0070423 nucleotide binding oligomerization domain containing signaling pathway	enriched	0.0314293	36	hsa-let-7c-5p; hsa-miR-155-5p; hsa-let-7a-5p; hsa-miR-324-3p; hsa-let-7f-5p; hsa-miR-30d-5p; hsa-miR-1-3p; hsa-miR-877-5p; hsa-miR-26b-5p; hsa-miR-320a; hsa-miR-103a-3p; hsa-miR-106b-5p; hsa-miR-760; hsa-miR-1287-5p; hsa-miR-146a-5p; hsa-miR-16-5p; hsa-miR-24-3p; hsa-miR-181a-5p; hsa-miR-181b-5p; hsa-miR-21-5p; hsa-miR-223-3p; hsa-miR-29a-3p; hsa-miR-26a-5p; hsa-miR-15a-5p; hsa-miR-17-5p; hsa-miR-18a-5p; hsa-miR-10a-5p; hsa-miR-15b-5p; hsa-miR-149-5p; hsa-miR-92a-3p; hsa-miR-615-3p; hsa-miR-30b-5p; hsa-miR-1226-3p; hsa-miR-197-3p; hsa-miR-30c-5p; hsa-miR-328-3p

Gene Ontology (miRWalk)	GO0070888 e box binding	enriched	0.0314293	30	hsa-let-7c-5p; hsa-miR-155-5p; hsa-let-7a-5p; hsa-let-7f-5p; hsa-miR-1-3p; hsa-miR-877-5p; hsa-miR-26b-5p; hsa-miR-320a; hsa-let-7g-5p; hsa-miR-106b-5p; hsa-miR-451a; hsa-miR-142-3p; hsa-miR-16-5p; hsa-miR-20a-5p; hsa-miR-24-3p; hsa-miR-181a-5p; hsa-miR-124-3p; hsa-miR-21-5p; hsa-miR-223-3p; hsa-miR-26a-5p; hsa-miR-449c-5p; hsa-miR-19b-3p; hsa-miR-17-5p; hsa-miR-148b-3p; hsa-miR-92a-3p; hsa-miR-615-3p; hsa-miR-204-5p; hsa-miR-331-3p; hsa-miR-34b-3p; hsa-miR-211-5p
Gene Ontology (miRWalk)	GO0071813 lipoprotein particle binding	enriched	0.0314293	3	hsa-miR-155-5p; hsa-miR-1-3p; hsa-miR-26b-5p
Pathways (miRWalk)	P00012 Cadherin signaling pathway	enriched	0.0314477	48	hsa-miR-29c-3p; hsa-let-7c-5p; hsa-miR-155-5p; hsa-let-7a-5p; hsa-miR-324-3p; hsa-miR-1-3p; hsa-miR-877-5p; hsa-miR-26b-5p; hsa-miR-320a; hsa-miR-103a-3p; hsa-miR-1227-3p; hsa-miR-199b-5p; hsa-miR-106b-5p; hsa-miR-760; hsa-miR-146a-5p; hsa-miR-1295a; hsa-miR-548d-3p; hsa-miR-142-3p; hsa-miR-16-5p; hsa-miR-24-3p; hsa-miR-181a-5p; hsa-miR-93-5p; hsa-miR-124-3p; hsa-miR-21-5p; hsa-miR-26a-5p; hsa-miR-99b-3p; hsa-miR-19a-3p; hsa-miR-1229-3p; hsa-miR-15a-5p; hsa-miR-26a-2-3p; hsa-miR-940; hsa-miR-320c; hsa-miR-19b-3p; hsa-miR-25-3p; hsa-miR-17-5p; hsa-miR-10a-5p; hsa-miR-148b-3p; hsa-miR-149-5p; hsa-miR-92a-3p; hsa-miR-615-3p; hsa-miR-204-5p; hsa-miR-337-3p; hsa-miR-1226-3p; hsa-miR-331-3p; hsa-miR-197-3p; hsa-miR-744-3p; hsa-miR-211-5p; hsa-miR-30c-5p
Pathways (miRWalk)	P00050 Plasminogen activating cascade	enriched	0.0314477	10	hsa-miR-29c-3p; hsa-miR-155-5p; hsa-miR-26b-5p; hsa-miR-16-5p; hsa-miR-29b-3p; hsa-miR-124-3p; hsa-miR-21-5p; hsa-miR-29a-3p; hsa-miR-204-5p; hsa-miR-30c-5p
Pathways (miRWalk)	WP1539 Angiogenesis	enriched	0.0314477	29	hsa-miR-155-5p; hsa-miR-324-3p; hsa-miR-30d-5p; hsa-miR-1-3p; hsa-miR-26b-5p; hsa-miR-320a; hsa-miR-103a-3p; hsa-miR-199b-5p; hsa-miR-18b-5p; hsa-miR-451a; hsa-miR-107; hsa-miR-16-5p; hsa-miR-20a-5p; hsa-miR-92b-3p; hsa-miR-181b-5p; hsa-miR-124-3p; hsa-miR-21-5p; hsa-miR-26a-5p; hsa-miR-1914-3p; hsa-miR-20b-5p; hsa-miR-766-3p; hsa-miR-17-5p; hsa-miR-18a-5p; hsa-miR-15b-5p; hsa-miR-148b-3p; hsa-miR-92a-3p; hsa-miR-30b-5p; hsa-miR-744-3p; hsa-miR-30c-5p
Pathways (miRWalk)	hsa00240 Pyrimidine metabolism	enriched	0.0314477	44	hsa-let-7c-5p; hsa-miR-155-5p; hsa-let-7a-5p; hsa-miR-324-3p; hsa-miR-30d-5p; hsa-miR-1-3p; hsa-miR-365a-5p; hsa-miR-26b-5p; hsa-miR-320a; hsa-let-7g-5p; hsa-miR-103a-3p; hsa-miR-106b-5p; hsa-let-7i-5p; hsa-miR-142-3p; hsa-miR-16-5p; hsa-miR-24-3p; hsa-miR-92b-3p; hsa-miR-671-5p; hsa-miR-365a-3p; hsa-miR-374a-3p; hsa-miR-93-5p; hsa-miR-124-3p; hsa-miR-21-5p; hsa-miR-29a-3p; hsa-miR-26a-5p; hsa-miR-1229-3p; hsa-miR-15a-5p; hsa-miR-1268a; hsa-miR-186-3p; hsa-miR-10a-5p; hsa-miR-148b-3p; hsa-miR-149-5p; hsa-miR-504-5p; hsa-miR-92a-3p; hsa-miR-615-3p; hsa-miR-1260b; hsa-miR-130b-5p; hsa-miR-1226-3p; hsa-miR-331-3p; hsa-miR-197-3p; hsa-miR-346; hsa-miR-30c-5p; hsa-miR-328-3p; hsa-miR-1296-5p
Pathways (miRWalk)	hsa00970 Aminoacyl tRNA biosynthesis	enriched	0.0314477	29	hsa-let-7c-5p; hsa-miR-155-5p; hsa-let-7a-5p; hsa-miR-324-3p; hsa-let-7f-5p; hsa-miR-30d-5p; hsa-miR-26b-5p; hsa-miR-320a; hsa-miR-106b-5p; hsa-miR-760; hsa-miR-16-5p; hsa-miR-24-3p; hsa-miR-92b-3p; hsa-miR-93-5p; hsa-miR-124-3p; hsa-miR-21-5p; hsa-miR-29a-3p; hsa-miR-26a-5p; hsa-miR-19b-3p; hsa-miR-766-3p; hsa-miR-17-5p; hsa-miR-10a-5p; hsa-miR-15b-5p; hsa-miR-149-5p; hsa-miR-92a-3p; hsa-miR-615-3p; hsa-miR-1226-3p; hsa-miR-331-3p; hsa-miR-197-3p
Pathways (miRWalk)	hsa04742 Taste transduction	enriched	0.0314477	18	hsa-miR-155-5p; hsa-miR-324-3p; hsa-miR-1-3p; hsa-miR-26b-5p; hsa-miR-320a; hsa-miR-103a-3p; hsa-miR-1227-3p; hsa-miR-142-3p; hsa-miR-16-5p;

					hsa-miR-365a-3p; hsa-miR-93-5p; hsa-miR-124-3p; hsa-miR-19b-3p; hsa-miR-17-5p; hsa-miR-92a-3p; hsa-miR-615-3p; hsa-miR-1226-3p; hsa-miR-331-3p
Gene Ontology (miRWalk)	GO0003924 gtpase activity	enriched	0.0315028	65	hsa-miR-29c-3p; hsa-miR-22-5p; hsa-let-7c-5p; hsa-miR-155-5p; hsa-let-7a-5p; hsa-miR-324-3p; hsa-let-7f-5p; hsa-miR-30d-5p; hsa-miR-1-3p; hsa-miR-877-5p; hsa-miR-26b-5p; hsa-miR-320a; hsa-let-7g-5p; hsa-miR-103a-3p; hsa-miR-1250-5p; hsa-miR-106b-5p; hsa-miR-760; hsa-miR-451a; hsa-miR-146a-5p; hsa-miR-142-3p; hsa-miR-107; hsa-miR-16-5p; hsa-miR-20a-5p; hsa-miR-24-3p; hsa-miR-92b-3p; hsa-miR-29b-3p; hsa-miR-365a-3p; hsa-miR-181a-5p; hsa-miR-181d-5p; hsa-miR-93-5p; hsa-miR-124-3p; hsa-miR-21-5p; hsa-miR-223-3p; hsa-miR-29a-3p; hsa-miR-26a-5p; hsa-miR-99b-3p; hsa-miR-19a-3p; hsa-miR-1229-3p; hsa-miR-15a-5p; hsa-miR-148a-3p; hsa-miR-940; hsa-miR-320c; hsa-miR-19b-3p; hsa-miR-25-3p; hsa-miR-17-5p; hsa-miR-18a-5p; hsa-miR-10a-5p; hsa-miR-15b-5p; hsa-miR-148b-3p; hsa-miR-149-5p; hsa-miR-23a-3p; hsa-miR-92a-3p; hsa-miR-615-3p; hsa-miR-505-3p; hsa-miR-1260b; hsa-miR-30b-5p; hsa-miR-130b-5p; hsa-miR-204-5p; hsa-miR-337-3p; hsa-miR-1226-3p; hsa-miR-331-3p; hsa-miR-197-3p; hsa-miR-211-5p; hsa-miR-30c-5p; hsa-miR-1296-5p
Gene Ontology (miRWalk)	GO0005337 nucleoside transmembrane transporter activity	enriched	0.0315028	6	hsa-miR-1-3p; hsa-miR-26b-5p; hsa-miR-16-5p; hsa-miR-24-3p; hsa-miR-93-5p; hsa-miR-124-3p
Gene Ontology (miRWalk)	GO0006465 signal peptide processing	enriched	0.0315028	9	hsa-miR-155-5p; hsa-miR-1-3p; hsa-miR-320a; hsa-miR-103a-3p; hsa-miR-107; hsa-miR-16-5p; hsa-miR-24-3p; hsa-miR-124-3p; hsa-miR-615-3p
Gene Ontology (miRWalk)	GO0006906 vesicle fusion	enriched	0.0315028	6	hsa-miR-1-3p; hsa-miR-26b-5p; hsa-miR-16-5p; hsa-miR-181a-5p; hsa-miR-93-5p; hsa-miR-124-3p
Gene Ontology (miRWalk)	GO0007281 germ cell development	enriched	0.0315028	30	hsa-let-7c-5p; hsa-miR-155-5p; hsa-let-7a-5p; hsa-let-7f-5p; hsa-miR-1-3p; hsa-miR-877-5p; hsa-miR-26b-5p; hsa-let-7g-5p; hsa-miR-103a-3p; hsa-miR-451a; hsa-miR-146a-5p; hsa-miR-16-5p; hsa-miR-24-3p; hsa-miR-365a-3p; hsa-miR-181a-5p; hsa-miR-93-5p; hsa-miR-181b-5p; hsa-miR-124-3p; hsa-miR-21-5p; hsa-miR-99b-3p; hsa-miR-15a-5p; hsa-miR-148a-3p; hsa-miR-19b-3p; hsa-miR-25-3p; hsa-miR-15b-5p; hsa-miR-148b-3p; hsa-miR-23a-3p; hsa-miR-504-5p; hsa-miR-197-3p; hsa-miR-296-5p
Gene Ontology (miRWalk)	GO0008333 endosome to lysosome transport	enriched	0.0315028	21	hsa-miR-155-5p; hsa-miR-324-3p; hsa-miR-1-3p; hsa-miR-26b-5p; hsa-miR-103a-3p; hsa-miR-760; hsa-miR-16-5p; hsa-miR-20a-5p; hsa-miR-181a-5p; hsa-miR-181d-5p; hsa-miR-93-5p; hsa-miR-181b-5p; hsa-miR-124-3p; hsa-miR-21-5p; hsa-miR-223-3p; hsa-miR-149-5p; hsa-miR-92a-3p; hsa-miR-615-3p; hsa-miR-204-5p; hsa-miR-331-3p; hsa-miR-30c-5p
Gene Ontology (miRWalk)	GO0009058 biosynthetic process	enriched	0.0315028	6	hsa-miR-155-5p; hsa-miR-1-3p; hsa-miR-26b-5p; hsa-miR-103a-3p; hsa-miR-16-5p; hsa-miR-124-3p
Gene Ontology (miRWalk)	GO0010506 regulation of autophagy	enriched	0.0315028	19	hsa-miR-155-5p; hsa-miR-1-3p; hsa-miR-26b-5p; hsa-miR-320a; hsa-miR-103a-3p; hsa-miR-107; hsa-miR-16-5p; hsa-miR-20a-5p; hsa-miR-24-3p; hsa-miR-92b-3p; hsa-miR-93-5p; hsa-miR-181b-5p; hsa-miR-124-3p; hsa-miR-29a-3p; hsa-miR-19b-3p; hsa-miR-25-3p; hsa-miR-17-5p; hsa-miR-149-5p; hsa-miR-92a-3p
Gene Ontology (miRWalk)	GO0010894 negative regulation of steroid biosynthetic process	enriched	0.0315028	9	hsa-miR-29c-3p; hsa-miR-26b-5p; hsa-miR-103a-3p; hsa-miR-106b-5p; hsa-miR-142-3p; hsa-miR-107; hsa-miR-20a-5p; hsa-miR-124-3p; hsa-miR-92a-3p
Gene Ontology (miRWalk)	GO0014065 phosphatidylinositol 3 kinase cascade	enriched	0.0315028	19	hsa-miR-155-5p; hsa-let-7a-5p; hsa-miR-1-3p; hsa-miR-26b-5p; hsa-miR-320a; hsa-miR-199b-5p; hsa-miR-548d-3p; hsa-miR-24-3p; hsa-miR-93-5p; hsa-miR-181b-5p; hsa-miR-124-3p; hsa-miR-21-5p; hsa-miR-223-3p; hsa-miR-29a-3p;

					hsa-miR-148a-3p; hsa-miR-148b-3p; hsa-miR-92a-3p; hsa-miR-1226-3p; hsa-miR-331-3p
Gene Ontology (miRWalk)	GO0015020 glucuronosyltransferase activity	enriched	0.0315028	6	hsa-miR-26b-5p; hsa-miR-320a; hsa-miR-142-3p; hsa-miR-16-5p; hsa-miR-181a-5p; hsa-miR-124-3p
Gene Ontology (miRWalk)	GO0018206 peptidyl methionine modification	enriched	0.0315028	6	hsa-let-7c-5p; hsa-miR-155-5p; hsa-miR-16-5p; hsa-miR-24-3p; hsa-miR-181a-5p; hsa-miR-124-3p
Gene Ontology (miRWalk)	GO0020037 heme binding	enriched	0.0315028	38	hsa-let-7c-5p; hsa-miR-155-5p; hsa-let-7a-5p; hsa-miR-324-3p; hsa-let-7f-5p; hsa-miR-1-3p; hsa-miR-26b-5p; hsa-miR-320a; hsa-miR-103a-3p; hsa-miR-550a-5p; hsa-miR-760; hsa-miR-107; hsa-miR-16-5p; hsa-miR-20a-5p; hsa-miR-24-3p; hsa-miR-92b-3p; hsa-miR-422a; hsa-miR-181a-5p; hsa-miR-181b-5p; hsa-miR-124-3p; hsa-miR-21-5p; hsa-miR-29a-3p; hsa-miR-26a-5p; hsa-miR-99b-3p; hsa-miR-148a-3p; hsa-miR-320c; hsa-miR-19b-3p; hsa-miR-25-3p; hsa-miR-766-3p; hsa-miR-17-5p; hsa-miR-10a-5p; hsa-miR-15b-5p; hsa-miR-148b-3p; hsa-miR-149-5p; hsa-miR-92a-3p; hsa-miR-615-3p; hsa-miR-30b-5p; hsa-miR-1226-3p
Gene Ontology (miRWalk)	GO0030193 regulation of blood coagulation	enriched	0.0315028	6	hsa-let-7a-5p; hsa-miR-1-3p; hsa-miR-26b-5p; hsa-miR-103a-3p; hsa-miR-16-5p; hsa-miR-124-3p
Gene Ontology (miRWalk)	GO0030449 regulation of complement activation	enriched	0.0315028	9	hsa-let-7a-5p; hsa-miR-26b-5p; hsa-miR-146a-5p; hsa-miR-142-3p; hsa-miR-16-5p; hsa-miR-181a-5p; hsa-miR-93-5p; hsa-miR-124-3p; hsa-miR-19b-3p
Gene Ontology (miRWalk)	GO0031365 n terminal protein amino acid modification	enriched	0.0315028	6	hsa-let-7c-5p; hsa-miR-155-5p; hsa-miR-16-5p; hsa-miR-24-3p; hsa-miR-181a-5p; hsa-miR-124-3p
Gene Ontology (miRWalk)	GO0031998 regulation of fatty acid beta oxidation	enriched	0.0315028	6	hsa-miR-155-5p; hsa-miR-1-3p; hsa-miR-320a; hsa-miR-16-5p; hsa-miR-93-5p; hsa-miR-181b-5p
Gene Ontology (miRWalk)	GO0032259 methylation	enriched	0.0315028	9	hsa-let-7c-5p; hsa-miR-155-5p; hsa-let-7a-5p; hsa-miR-324-3p; hsa-miR-26b-5p; hsa-miR-106b-5p; hsa-miR-16-5p; hsa-miR-124-3p; hsa-miR-10a-5p
Gene Ontology (miRWalk)	GO0034405 response to fluid shear stress	enriched	0.0315028	6	hsa-miR-155-5p; hsa-miR-26b-5p; hsa-miR-103a-3p; hsa-miR-451a; hsa-miR-93-5p; hsa-miR-124-3p
Gene Ontology (miRWalk)	GO0043190 atp binding cassette abc transporter complex	enriched	0.0315028	6	hsa-miR-1-3p; hsa-miR-26b-5p; hsa-miR-320a; hsa-miR-103a-3p; hsa-miR-16-5p; hsa-miR-124-3p
Gene Ontology (miRWalk)	GO0044548 s100 protein binding	enriched	0.0315028	21	hsa-let-7c-5p; hsa-miR-155-5p; hsa-let-7a-5p; hsa-miR-324-3p; hsa-let-7f-5p; hsa-miR-1-3p; hsa-miR-26b-5p; hsa-miR-320a; hsa-miR-142-3p; hsa-miR-16-5p; hsa-miR-29b-3p; hsa-miR-671-5p; hsa-miR-181a-5p; hsa-miR-124-3p; hsa-miR-29a-3p; hsa-miR-25-3p; hsa-miR-10a-5p; hsa-miR-15b-5p; hsa-miR-92a-3p; hsa-miR-1226-3p; hsa-miR-197-3p
Gene Ontology (miRWalk)	GO0045947 negative regulation of translational initiation	enriched	0.0315028	12	hsa-miR-155-5p; hsa-let-7a-5p; hsa-miR-30d-5p; hsa-miR-760; hsa-miR-107; hsa-miR-16-5p; hsa-miR-671-5p; hsa-miR-124-3p; hsa-miR-21-5p; hsa-miR-29a-3p; hsa-miR-18a-5p; hsa-miR-197-3p
Gene Ontology (miRWalk)	GO0046655 folic acid metabolic process	enriched	0.0315028	9	hsa-let-7a-5p; hsa-miR-1-3p; hsa-miR-877-5p; hsa-miR-103a-3p; hsa-miR-106b-5p; hsa-miR-16-5p; hsa-miR-93-5p; hsa-miR-124-3p; hsa-miR-149-5p

Gene Ontology (miRWalk)	GO0071208 histone pre mrna dcp binding	enriched	0.0315028	6	hsa-miR-1-3p; hsa-miR-26b-5p; hsa-miR-320a; hsa-miR-16-5p; hsa-miR-24-3p; hsa-miR-124-3p
Gene Ontology (miRWalk)	GO0090004 positive regulation of establishment of protein localization in plasma membrane	enriched	0.0315028	19	hsa-miR-155-5p; hsa-let-7a-5p; hsa-miR-1-3p; hsa-miR-26b-5p; hsa-miR-320a; hsa-miR-103a-3p; hsa-miR-451a; hsa-miR-16-5p; hsa-miR-24-3p; hsa-miR-181a-5p; hsa-miR-93-5p; hsa-miR-124-3p; hsa-miR-21-5p; hsa-miR-29a-3p; hsa-miR-940; hsa-miR-17-5p; hsa-miR-10a-5p; hsa-miR-204-5p; hsa-miR-331-3p
Gene Ontology (miRWalk)	GO0090201 negative regulation of release of cytochrome c from mitochondria	enriched	0.0315028	6	hsa-let-7c-5p; hsa-miR-26b-5p; hsa-miR-320a; hsa-let-7g-5p; hsa-miR-451a; hsa-miR-124-3p
Gene Ontology (miRWalk)	GO0006464 cellular protein modification process	enriched	0.0318084	45	hsa-miR-29c-3p; hsa-let-7c-5p; hsa-miR-155-5p; hsa-let-7a-5p; hsa-miR-324-3p; hsa-miR-30d-5p; hsa-miR-1-3p; hsa-miR-877-5p; hsa-miR-26b-5p; hsa-miR-320a; hsa-miR-103a-3p; hsa-miR-106b-5p; hsa-miR-760; hsa-miR-451a; hsa-miR-184; hsa-miR-142-3p; hsa-miR-107; hsa-miR-16-5p; hsa-miR-20a-5p; hsa-miR-29b-3p; hsa-miR-671-5p; hsa-miR-181a-5p; hsa-miR-93-5p; hsa-miR-124-3p; hsa-miR-21-5p; hsa-miR-29a-3p; hsa-miR-26a-5p; hsa-miR-1229-3p; hsa-miR-188-5p; hsa-miR-15a-5p; hsa-miR-320c; hsa-miR-19b-3p; hsa-miR-17-5p; hsa-miR-10a-5p; hsa-miR-149-5p; hsa-miR-92a-3p; hsa-miR-615-3p; hsa-miR-1260b; hsa-miR-30b-5p; hsa-miR-204-5p; hsa-miR-1226-3p; hsa-miR-331-3p; hsa-miR-454-5p; hsa-miR-30c-5p; hsa-miR-1296-5p
Gene Ontology (miRWalk)	GO0007507 heart development	enriched	0.0318084	63	hsa-miR-29c-3p; hsa-let-7c-5p; hsa-miR-155-5p; hsa-miR-193b-5p; hsa-let-7a-5p; hsa-miR-324-3p; hsa-let-7f-5p; hsa-miR-30d-5p; hsa-miR-1-3p; hsa-miR-877-5p; hsa-miR-26b-5p; hsa-miR-320a; hsa-miR-103a-3p; hsa-miR-199b-5p; hsa-miR-106b-5p; hsa-miR-760; hsa-miR-146a-5p; hsa-miR-548d-3p; hsa-miR-142-3p; hsa-miR-107; hsa-miR-16-5p; hsa-miR-20a-5p; hsa-miR-24-3p; hsa-miR-92b-3p; hsa-miR-29b-3p; hsa-miR-671-5p; hsa-miR-181a-5p; hsa-miR-181d-5p; hsa-miR-93-5p; hsa-miR-181b-5p; hsa-miR-124-3p; hsa-miR-21-5p; hsa-miR-223-3p; hsa-miR-29a-3p; hsa-miR-26a-5p; hsa-miR-19a-3p; hsa-miR-15a-5p; hsa-miR-20b-5p; hsa-miR-148a-3p; hsa-miR-940; hsa-miR-19b-3p; hsa-miR-494-3p; hsa-miR-25-3p; hsa-miR-17-5p; hsa-miR-18a-5p; hsa-miR-10a-5p; hsa-miR-15b-5p; hsa-miR-148b-3p; hsa-miR-149-5p; hsa-miR-23a-3p; hsa-miR-129-2-3p; hsa-miR-92a-3p; hsa-miR-615-3p; hsa-miR-1260b; hsa-miR-30b-5p; hsa-miR-130b-5p; hsa-miR-204-5p; hsa-miR-331-3p; hsa-miR-1236-3p; hsa-miR-346; hsa-miR-34b-3p; hsa-miR-30c-5p; hsa-miR-328-3p
Gene Ontology (miRWalk)	GO0030521 androgen receptor signaling pathway	enriched	0.0318084	50	hsa-miR-22-5p; hsa-miR-155-5p; hsa-miR-324-3p; hsa-miR-1-3p; hsa-miR-877-5p; hsa-miR-26b-5p; hsa-miR-320a; hsa-miR-103a-3p; hsa-miR-1250-5p; hsa-miR-106b-5p; hsa-miR-18b-5p; hsa-miR-760; hsa-miR-146a-5p; hsa-miR-425-3p; hsa-miR-107; hsa-miR-16-5p; hsa-miR-20a-5p; hsa-miR-24-3p; hsa-miR-92b-3p; hsa-miR-29b-3p; hsa-miR-365a-3p; hsa-miR-181a-5p; hsa-miR-93-5p; hsa-miR-181b-5p; hsa-miR-124-3p; hsa-miR-21-5p; hsa-miR-29a-3p; hsa-miR-26a-5p; hsa-miR-99b-3p; hsa-miR-19a-3p; hsa-miR-15a-5p; hsa-miR-320c; hsa-miR-19b-3p; hsa-miR-766-3p; hsa-miR-17-5p; hsa-miR-18a-5p; hsa-miR-10a-5p; hsa-miR-15b-5p; hsa-miR-149-5p; hsa-miR-23a-3p; hsa-miR-92a-3p; hsa-miR-615-3p; hsa-miR-1260b; hsa-miR-181a-2-3p; hsa-miR-1226-3p; hsa-miR-331-3p; hsa-miR-197-3p; hsa-miR-346; hsa-miR-30c-5p; hsa-miR-328-3p
Gene Ontology (miRWalk)	GO0030889 negative regulation of b cell proliferation	enriched	0.0318084	16	hsa-miR-155-5p; hsa-let-7a-5p; hsa-let-7f-5p; hsa-miR-30d-5p; hsa-miR-877-5p; hsa-miR-26b-5p; hsa-miR-320a; hsa-let-7g-5p; hsa-miR-16-5p; hsa-miR-24-3p;

					hsa-miR-181a-5p; hsa-miR-124-3p; hsa-miR-19b-3p; hsa-miR-18a-5p; hsa-miR-92a-3p; hsa-miR-615-3p
Gene Ontology (miRWalk)	GO0032947 protein complex scaffold	enriched	0.0318084	25	hsa-let-7c-5p; hsa-let-7a-5p; hsa-miR-324-3p; hsa-let-7f-5p; hsa-miR-1-3p; hsa-miR-26b-5p; hsa-miR-320a; hsa-let-7g-5p; hsa-miR-103a-3p; hsa-miR-106b-5p; hsa-miR-16-5p; hsa-miR-20a-5p; hsa-miR-24-3p; hsa-miR-93-5p; hsa-miR-124-3p; hsa-miR-21-5p; hsa-miR-223-3p; hsa-miR-320c; hsa-miR-766-3p; hsa-miR-148b-3p; hsa-miR-149-5p; hsa-miR-92a-3p; hsa-miR-615-3p; hsa-miR-30b-5p; hsa-miR-331-3p
Gene Ontology (miRWalk)	GO0043621 protein self association	enriched	0.0318084	22	hsa-miR-155-5p; hsa-miR-1-3p; hsa-miR-26b-5p; hsa-miR-320a; hsa-miR-199b-5p; hsa-miR-146a-5p; hsa-miR-425-3p; hsa-miR-16-5p; hsa-miR-20a-5p; hsa-miR-24-3p; hsa-miR-93-5p; hsa-miR-124-3p; hsa-miR-21-5p; hsa-miR-29a-3p; hsa-miR-26a-5p; hsa-miR-19a-3p; hsa-miR-148a-3p; hsa-miR-320c; hsa-miR-19b-3p; hsa-miR-10a-5p; hsa-miR-92a-3p; hsa-miR-615-3p
Gene Ontology (miRWalk)	GO0060045 positive regulation of cardiac muscle cell proliferation	enriched	0.0318084	22	hsa-let-7c-5p; hsa-miR-155-5p; hsa-miR-1-3p; hsa-miR-26b-5p; hsa-miR-103a-3p; hsa-miR-146a-5p; hsa-miR-16-5p; hsa-miR-20a-5p; hsa-miR-24-3p; hsa-miR-92b-3p; hsa-miR-181a-5p; hsa-miR-181b-5p; hsa-miR-124-3p; hsa-miR-21-5p; hsa-miR-223-3p; hsa-miR-19a-3p; hsa-miR-18a-5p; hsa-miR-10a-5p; hsa-miR-148b-3p; hsa-miR-663a; hsa-miR-92a-3p; hsa-miR-34b-3p
Gene Ontology (miRWalk)	GO0097191 extrinsic apoptotic signaling pathway	enriched	0.0318084	7	hsa-miR-155-5p; hsa-let-7a-5p; hsa-miR-1-3p; hsa-miR-26b-5p; hsa-miR-146a-5p; hsa-miR-16-5p; hsa-miR-15b-5p
Pathways (miRWalk)	hsa04320 Dorso ventral axis formation	enriched	0.0320413	28	hsa-let-7c-5p; hsa-miR-155-5p; hsa-let-7a-5p; hsa-miR-1-3p; hsa-miR-877-5p; hsa-miR-320a; hsa-let-7g-5p; hsa-miR-146a-5p; hsa-miR-107; hsa-miR-16-5p; hsa-miR-24-3p; hsa-miR-92b-3p; hsa-miR-181a-5p; hsa-miR-483-5p; hsa-miR-93-5p; hsa-miR-124-3p; hsa-miR-21-5p; hsa-miR-25-3p; hsa-miR-766-3p; hsa-miR-17-5p; hsa-miR-18a-5p; hsa-miR-10a-5p; hsa-miR-15b-5p; hsa-miR-149-5p; hsa-miR-92a-3p; hsa-miR-130b-5p; hsa-miR-34b-3p; hsa-miR-30c-5p
Gene Ontology (miRWalk)	GO0001892 embryonic placenta development	enriched	0.0323687	27	hsa-miR-155-5p; hsa-let-7a-5p; hsa-let-7f-5p; hsa-miR-193a-5p; hsa-miR-1-3p; hsa-miR-26b-5p; hsa-miR-103a-3p; hsa-miR-199b-5p; hsa-miR-106b-5p; hsa-miR-146a-5p; hsa-miR-107; hsa-miR-16-5p; hsa-miR-20a-5p; hsa-miR-29b-3p; hsa-miR-93-5p; hsa-miR-124-3p; hsa-miR-21-5p; hsa-miR-223-3p; hsa-miR-20b-5p; hsa-miR-17-5p; hsa-miR-18a-5p; hsa-miR-10a-5p; hsa-miR-149-5p; hsa-miR-615-3p; hsa-miR-1260b; hsa-miR-1226-3p; hsa-miR-30c-5p
Gene Ontology (miRWalk)	GO0031333 negative regulation of protein complex assembly	enriched	0.0323687	17	hsa-miR-29c-3p; hsa-miR-155-5p; hsa-miR-1-3p; hsa-miR-26b-5p; hsa-miR-106b-5p; hsa-miR-16-5p; hsa-miR-92b-3p; hsa-miR-29b-3p; hsa-miR-181a-5p; hsa-miR-124-3p; hsa-miR-21-5p; hsa-miR-29a-3p; hsa-miR-26a-5p; hsa-miR-92a-3p; hsa-miR-629-3p; hsa-miR-130b-5p; hsa-miR-204-5p
Gene Ontology (miRWalk)	GO0045859 regulation of protein kinase activity	enriched	0.0323687	17	hsa-miR-29c-3p; hsa-miR-1-3p; hsa-miR-26b-5p; hsa-miR-320a; hsa-miR-103a-3p; hsa-miR-106b-5p; hsa-miR-146a-5p; hsa-miR-16-5p; hsa-miR-24-3p; hsa-miR-29b-3p; hsa-miR-181b-5p; hsa-miR-29a-3p; hsa-miR-26a-5p; hsa-miR-15a-5p; hsa-miR-92a-3p; hsa-miR-197-3p; hsa-miR-30c-5p
Diseases (published studies about miRNA profiles from peripheral blood)	benign prostate hyperplasia downregulated	enriched	0.032631	24	hsa-miR-371a-5p; hsa-miR-155-5p; hsa-let-7a-5p; hsa-miR-26b-5p; hsa-let-7g-5p; hsa-miR-520d-3p; hsa-miR-106b-5p; hsa-miR-18b-5p; hsa-miR-760; hsa-miR-548d-3p; hsa-miR-20a-5p; hsa-miR-93-5p; hsa-miR-181b-5p; hsa-miR-595; hsa-miR-21-5p; hsa-miR-26a-5p; hsa-miR-130a-5p; hsa-miR-20b-5p; hsa-miR-148a-3p; hsa-miR-17-5p; hsa-miR-581; hsa-miR-18a-5p; hsa-miR-148b-3p; hsa-miR-218-1-3p

Pathways (miRWalk)	hsa04010 MAPK signaling pathway	enriched	0.0329955	77	hsa-miR-29c-3p; hsa-let-7c-5p; hsa-miR-155-5p; hsa-miR-193b-5p; hsa-let-7a-5p; hsa-miR-324-3p; hsa-let-7f-5p; hsa-miR-30d-5p; hsa-miR-1-3p; hsa-miR-877-5p; hsa-miR-26b-5p; hsa-miR-320a; hsa-let-7g-5p; hsa-miR-103a-3p; hsa-miR-106b-5p; hsa-miR-760; hsa-miR-451a; hsa-miR-184; hsa-miR-146a-5p; hsa-miR-142-3p; hsa-miR-425-3p; hsa-miR-107; hsa-miR-16-5p; hsa-miR-20a-5p; hsa-miR-24-3p; hsa-miR-92b-3p; hsa-miR-29b-3p; hsa-miR-365a-3p; hsa-miR-181a-5p; hsa-miR-483-5p; hsa-miR-181d-5p; hsa-miR-93-5p; hsa-miR-181b-5p; hsa-miR-124-3p; hsa-miR-21-5p; hsa-miR-223-3p; hsa-miR-29a-3p; hsa-miR-26a-5p; hsa-miR-99b-3p; hsa-miR-19a-3p; hsa-miR-1229-3p; hsa-miR-15a-5p; hsa-miR-449c-5p; hsa-miR-186-3p; hsa-miR-148a-3p; hsa-miR-940; hsa-miR-191-3p; hsa-miR-19b-3p; hsa-miR-25-3p; hsa-miR-766-3p; hsa-miR-17-5p; hsa-miR-18a-5p; hsa-miR-10a-5p; hsa-miR-129-1-3p; hsa-miR-15b-5p; hsa-miR-148b-3p; hsa-miR-149-5p; hsa-miR-663a; hsa-miR-23a-3p; hsa-miR-504-5p; hsa-miR-92a-3p; hsa-miR-615-3p; hsa-miR-505-3p; hsa-miR-1260b; hsa-miR-30b-5p; hsa-miR-130b-5p; hsa-miR-204-5p; hsa-miR-337-3p; hsa-miR-1226-3p; hsa-miR-331-3p; hsa-miR-197-3p; hsa-miR-346; hsa-miR-34b-3p; hsa-miR-30c-5p; hsa-miR-605-5p; hsa-miR-328-3p; hsa-miR-1296-5p
Gene Ontology (miRWalk)	GO0007030 golgi organization	enriched	0.0330136	29	hsa-miR-29c-3p; hsa-let-7c-5p; hsa-miR-155-5p; hsa-let-7a-5p; hsa-miR-324-3p; hsa-miR-1-3p; hsa-miR-877-5p; hsa-miR-26b-5p; hsa-miR-320a; hsa-miR-106b-5p; hsa-miR-142-3p; hsa-miR-16-5p; hsa-miR-20a-5p; hsa-miR-24-3p; hsa-miR-29b-3p; hsa-miR-93-5p; hsa-miR-124-3p; hsa-miR-21-5p; hsa-miR-29a-3p; hsa-miR-15a-5p; hsa-miR-17-5p; hsa-miR-10a-5p; hsa-miR-148b-3p; hsa-miR-149-5p; hsa-miR-92a-3p; hsa-miR-615-3p; hsa-miR-331-3p; hsa-miR-197-3p; hsa-miR-1296-5p
Pathways (miRWalk)	P00003 Alzheimer disease amyloid secretase pathway	enriched	0.0333769	41	hsa-miR-29c-3p; hsa-miR-155-5p; hsa-let-7a-5p; hsa-miR-324-3p; hsa-miR-1-3p; hsa-miR-877-5p; hsa-miR-26b-5p; hsa-miR-320a; hsa-miR-103a-3p; hsa-miR-106b-5p; hsa-miR-425-3p; hsa-miR-107; hsa-miR-16-5p; hsa-miR-20a-5p; hsa-miR-24-3p; hsa-miR-92b-3p; hsa-miR-29b-3p; hsa-miR-181a-5p; hsa-miR-483-5p; hsa-miR-93-5p; hsa-miR-124-3p; hsa-miR-21-5p; hsa-miR-29a-3p; hsa-miR-15a-5p; hsa-miR-3620-3p; hsa-miR-19b-3p; hsa-miR-766-3p; hsa-miR-17-5p; hsa-miR-10a-5p; hsa-miR-15b-5p; hsa-miR-148b-3p; hsa-miR-23a-3p; hsa-miR-92a-3p; hsa-miR-615-3p; hsa-miR-505-3p; hsa-miR-1260b; hsa-miR-1226-3p; hsa-miR-331-3p; hsa-miR-197-3p; hsa-miR-30c-5p; hsa-miR-328-3p
Pathways (miRWalk)	WP167 Eicosanoid Synthesis	enriched	0.0333769	19	hsa-let-7c-5p; hsa-miR-155-5p; hsa-let-7a-5p; hsa-miR-26b-5p; hsa-miR-320a; hsa-miR-550a-5p; hsa-miR-760; hsa-miR-16-5p; hsa-miR-92b-3p; hsa-miR-181a-5p; hsa-miR-124-3p; hsa-miR-26a-5p; hsa-miR-99b-3p; hsa-miR-320c; hsa-miR-766-3p; hsa-miR-17-5p; hsa-miR-149-5p; hsa-miR-92a-3p; hsa-miR-615-3p
Pathways (miRWalk)	WP254 Apoptosis	enriched	0.0333769	61	hsa-miR-29c-3p; hsa-let-7c-5p; hsa-miR-155-5p; hsa-let-7a-5p; hsa-let-7f-5p; hsa-miR-193a-5p; hsa-miR-30d-5p; hsa-miR-1-3p; hsa-miR-876-3p; hsa-miR-877-5p; hsa-miR-26b-5p; hsa-miR-320a; hsa-let-7g-5p; hsa-miR-103a-3p; hsa-miR-106b-5p; hsa-miR-18b-5p; hsa-miR-451a; hsa-miR-146a-5p; hsa-miR-16-5p; hsa-miR-20a-5p; hsa-miR-24-3p; hsa-miR-29b-3p; hsa-miR-365a-3p; hsa-miR-181a-5p; hsa-miR-181d-5p; hsa-miR-93-5p; hsa-miR-181b-5p; hsa-miR-124-3p; hsa-miR-21-5p; hsa-miR-223-3p; hsa-miR-29a-3p; hsa-miR-26a-5p; hsa-miR-19a-3p; hsa-miR-15a-5p; hsa-miR-449c-5p; hsa-miR-148a-3p; hsa-miR-940; hsa-miR-320c; hsa-miR-19b-3p; hsa-miR-494-3p; hsa-miR-25-3p; hsa-miR-17-5p; hsa-miR-630; hsa-miR-10a-5p; hsa-miR-15b-5p; hsa-miR-148b-3p; hsa-miR-149-5p; hsa-miR-504-5p; hsa-miR-92a-3p; hsa-miR-3679-3p; hsa-miR-615-3p; hsa-miR-483-3p; hsa-miR-204-5p; hsa-miR-1226-3p; hsa-miR-331-3p; hsa-

					miR-346; hsa-miR-34b-3p; hsa-miR-30c-5p; hsa-miR-605-5p; hsa-miR-296-5p; hsa-miR-1296-5p
Pathways (miRWalk)	WP477 Cytoplasmic Ribosomal Proteins	enriched	0.0333769	69	hsa-miR-22-5p; hsa-let-7c-5p; hsa-miR-155-5p; hsa-miR-193b-5p; hsa-let-7a-5p; hsa-miR-324-3p; hsa-miR-193a-5p; hsa-miR-30d-5p; hsa-miR-1-3p; hsa-miR-877-5p; hsa-miR-26b-5p; hsa-miR-320a; hsa-let-7g-5p; hsa-miR-103a-3p; hsa-miR-1250-5p; hsa-miR-1227-3p; hsa-miR-106b-5p; hsa-miR-3188; hsa-miR-760; hsa-miR-107; hsa-miR-16-5p; hsa-miR-20a-5p; hsa-miR-24-3p; hsa-miR-92b-3p; hsa-miR-422a; hsa-miR-29b-3p; hsa-miR-671-5p; hsa-miR-181a-5p; hsa-miR-181d-5p; hsa-miR-93-5p; hsa-miR-181b-5p; hsa-miR-124-3p; hsa-miR-21-5p; hsa-miR-29a-3p; hsa-miR-26a-5p; hsa-miR-99b-3p; hsa-miR-1229-3p; hsa-miR-148a-3p; hsa-miR-940; hsa-miR-636; hsa-miR-19b-3p; hsa-miR-25-3p; hsa-miR-766-3p; hsa-miR-17-5p; hsa-miR-576-5p; hsa-miR-18a-5p; hsa-miR-10a-5p; hsa-miR-15b-5p; hsa-miR-149-5p; hsa-miR-3605-3p; hsa-miR-23a-3p; hsa-miR-504-5p; hsa-miR-92a-3p; hsa-miR-615-3p; hsa-miR-1237-3p; hsa-miR-505-3p; hsa-miR-1260b; hsa-miR-30b-5p; hsa-miR-130b-5p; hsa-miR-204-5p; hsa-miR-1226-3p; hsa-miR-331-3p; hsa-miR-1914-5p; hsa-miR-197-3p; hsa-miR-346; hsa-miR-30c-5p; hsa-miR-328-3p; hsa-miR-1296-5p; hsa-miR-196b-3p
Pathways (miRWalk)	WP545 Complement Activation Classical Pathway	enriched	0.0333769	7	hsa-let-7a-5p; hsa-miR-26b-5p; hsa-miR-142-3p; hsa-miR-16-5p; hsa-miR-181a-5p; hsa-miR-124-3p; hsa-miR-10a-5p
Pathways (miRWalk)	WP691 Tamoxifen metabolism	enriched	0.0333769	7	hsa-miR-26b-5p; hsa-miR-320a; hsa-miR-103a-3p; hsa-miR-107; hsa-miR-24-3p; hsa-miR-124-3p; hsa-miR-631
Pathways (miRWalk)	WP706 SIDS Susceptibility Pathways	enriched	0.0333769	42	hsa-let-7c-5p; hsa-miR-155-5p; hsa-let-7a-5p; hsa-let-7f-5p; hsa-miR-1-3p; hsa-miR-26b-5p; hsa-miR-103a-3p; hsa-miR-199b-5p; hsa-miR-106b-5p; hsa-miR-146a-5p; hsa-miR-425-3p; hsa-miR-16-5p; hsa-miR-20a-5p; hsa-miR-24-3p; hsa-miR-29b-3p; hsa-miR-365a-3p; hsa-miR-181a-5p; hsa-miR-181d-5p; hsa-miR-181b-5p; hsa-miR-124-3p; hsa-miR-21-5p; hsa-miR-29a-3p; hsa-miR-26a-5p; hsa-miR-134-5p; hsa-miR-1229-3p; hsa-miR-15a-5p; hsa-miR-19b-3p; hsa-miR-25-3p; hsa-miR-17-5p; hsa-miR-18a-5p; hsa-miR-149-5p; hsa-miR-23a-3p; hsa-miR-92a-3p; hsa-miR-615-3p; hsa-miR-130b-5p; hsa-miR-204-5p; hsa-miR-331-3p; hsa-miR-197-3p; hsa-miR-211-5p; hsa-miR-30c-5p; hsa-miR-605-5p; hsa-miR-1296-5p
Pathways (miRWalk)	hsa00130 Ubiquinone and other terpenoid quinone biosynthesis	enriched	0.0333769	4	hsa-miR-1-3p; hsa-miR-26b-5p; hsa-miR-16-5p; hsa-miR-93-5p
Pathways (miRWalk)	hsa03430 Mismatch repair	enriched	0.0333769	23	hsa-miR-155-5p; hsa-miR-324-3p; hsa-miR-1-3p; hsa-miR-26b-5p; hsa-miR-103a-3p; hsa-miR-106b-5p; hsa-miR-16-5p; hsa-miR-20a-5p; hsa-miR-24-3p; hsa-miR-92b-3p; hsa-miR-93-5p; hsa-miR-124-3p; hsa-miR-21-5p; hsa-miR-29a-3p; hsa-miR-26a-5p; hsa-miR-15a-5p; hsa-miR-19b-3p; hsa-miR-18a-5p;

					hsa-miR-149-5p; hsa-miR-92a-3p; hsa-miR-615-3p; hsa-miR-130b-5p; hsa-miR-1226-3p
Gene Ontology (miRWalk)	GO0045471 response to ethanol	enriched	0.0334593	57	hsa-miR-29c-3p; hsa-miR-155-5p; hsa-let-7a-5p; hsa-miR-324-3p; hsa-let-7f-5p; hsa-miR-1-3p; hsa-miR-877-5p; hsa-miR-26b-5p; hsa-miR-320a; hsa-let-7g-5p; hsa-miR-103a-3p; hsa-miR-106b-5p; hsa-let-7i-5p; hsa-miR-451a; hsa-miR-146a-5p; hsa-miR-107; hsa-miR-16-5p; hsa-miR-20a-5p; hsa-miR-24-3p; hsa-miR-92b-3p; hsa-miR-29b-3p; hsa-miR-671-5p; hsa-miR-365a-3p; hsa-miR-181a-5p; hsa-miR-181d-5p; hsa-miR-93-5p; hsa-miR-181b-5p; hsa-miR-124-3p; hsa-miR-21-5p; hsa-miR-223-3p; hsa-miR-29a-3p; hsa-miR-26a-5p; hsa-miR-19a-3p; hsa-miR-1229-3p; hsa-miR-15a-5p; hsa-miR-20b-5p; hsa-miR-148a-3p; hsa-miR-19b-3p; hsa-miR-25-3p; hsa-miR-17-5p; hsa-miR-630; hsa-miR-10a-5p; hsa-miR-15b-5p; hsa-miR-149-5p; hsa-miR-663a; hsa-miR-92a-3p; hsa-miR-615-3p; hsa-miR-505-3p; hsa-miR-1260b; hsa-miR-30b-5p; hsa-miR-130b-5p; hsa-miR-204-5p; hsa-miR-337-3p; hsa-miR-197-3p; hsa-miR-34b-3p; hsa-miR-30c-5p; hsa-miR-296-5p
Gene Ontology (miRWalk)	GO0071425 hemopoietic stem cell proliferation	enriched	0.0334593	15	hsa-miR-155-5p; hsa-miR-103a-3p; hsa-miR-106b-5p; hsa-miR-16-5p; hsa-miR-20a-5p; hsa-miR-24-3p; hsa-miR-181a-5p; hsa-miR-93-5p; hsa-miR-124-3p; hsa-miR-21-5p; hsa-miR-29a-3p; hsa-miR-19a-3p; hsa-miR-15a-5p; hsa-miR-17-5p; hsa-miR-92a-3p
Gene Ontology (miRWalk)	GO0001889 liver development	enriched	0.0334747	53	hsa-miR-29c-3p; hsa-let-7c-5p; hsa-miR-155-5p; hsa-let-7a-5p; hsa-miR-324-3p; hsa-let-7f-5p; hsa-miR-193a-5p; hsa-miR-1-3p; hsa-miR-877-5p; hsa-miR-26b-5p; hsa-miR-320a; hsa-miR-103a-3p; hsa-miR-199b-5p; hsa-miR-106b-5p; hsa-miR-107; hsa-miR-16-5p; hsa-miR-20a-5p; hsa-miR-24-3p; hsa-miR-92b-3p; hsa-miR-29b-3p; hsa-miR-365a-3p; hsa-miR-181a-5p; hsa-miR-181d-5p; hsa-miR-93-5p; hsa-miR-181b-5p; hsa-miR-124-3p; hsa-miR-21-5p; hsa-miR-223-3p; hsa-miR-26a-5p; hsa-miR-134-5p; hsa-miR-19a-3p; hsa-miR-1229-3p; hsa-miR-15a-5p; hsa-miR-320c; hsa-miR-19b-3p; hsa-miR-25-3p; hsa-miR-17-5p; hsa-miR-18a-5p; hsa-miR-10a-5p; hsa-miR-15b-5p; hsa-miR-148b-3p; hsa-miR-149-5p; hsa-miR-23a-3p; hsa-miR-92a-3p; hsa-miR-615-3p; hsa-miR-1260b; hsa-miR-130b-5p; hsa-miR-204-5p; hsa-miR-1226-3p; hsa-miR-331-3p; hsa-miR-34b-3p; hsa-miR-30c-5p; hsa-miR-296-5p
Gene Ontology (miRWalk)	GO0002282 microglial cell activation involved in immune response	enriched	0.0334747	6	hsa-miR-1-3p; hsa-miR-26b-5p; hsa-let-7i-5p; hsa-miR-146a-5p; hsa-miR-124-3p; hsa-miR-21-5p
Gene Ontology (miRWalk)	GO0004407 histone deacetylase activity	enriched	0.0334747	22	hsa-let-7c-5p; hsa-let-7a-5p; hsa-miR-1-3p; hsa-miR-26b-5p; hsa-miR-320a; hsa-miR-103a-3p; hsa-miR-146a-5p; hsa-miR-16-5p; hsa-miR-24-3p; hsa-miR-29b-3p; hsa-miR-671-5p; hsa-miR-365a-3p; hsa-miR-181a-5p; hsa-miR-181b-5p; hsa-miR-124-3p; hsa-miR-17-5p; hsa-miR-149-5p; hsa-miR-92a-3p; hsa-miR-615-3p; hsa-miR-505-3p; hsa-miR-331-3p; hsa-miR-30c-5p
Gene Ontology (miRWalk)	GO0004709 map kinase kinase kinase activity	enriched	0.0334747	19	hsa-miR-155-5p; hsa-let-7a-5p; hsa-miR-1-3p; hsa-miR-26b-5p; hsa-let-7g-5p; hsa-miR-103a-3p; hsa-miR-146a-5p; hsa-miR-16-5p; hsa-miR-20a-5p; hsa-miR-92b-3p; hsa-miR-93-5p; hsa-miR-124-3p; hsa-miR-21-5p; hsa-miR-26a-5p; hsa-miR-148a-3p; hsa-miR-17-5p; hsa-miR-10a-5p; hsa-miR-92a-3p; hsa-miR-615-3p

Gene Ontology (miRWalk)	GO0006284 base excision repair	enriched	0.0334747	43	hsa-miR-29c-3p; hsa-let-7c-5p; hsa-let-7a-5p; hsa-miR-324-3p; hsa-let-7f-5p; hsa-miR-30d-5p; hsa-miR-1-3p; hsa-miR-877-5p; hsa-miR-26b-5p; hsa-miR-320a; hsa-let-7g-5p; hsa-miR-103a-3p; hsa-miR-106b-5p; hsa-miR-760; hsa-miR-16-5p; hsa-miR-24-3p; hsa-miR-92b-3p; hsa-miR-671-5p; hsa-miR-181a-5p; hsa-miR-93-5p; hsa-miR-181b-5p; hsa-miR-124-3p; hsa-miR-21-5p; hsa-miR-223-3p; hsa-miR-29a-3p; hsa-miR-26a-5p; hsa-miR-1229-3p; hsa-miR-15a-5p; hsa-miR-25-3p; hsa-miR-766-3p; hsa-miR-17-5p; hsa-miR-18a-5p; hsa-miR-149-5p; hsa-miR-504-5p; hsa-miR-129-2-3p; hsa-miR-92a-3p; hsa-miR-615-3p; hsa-miR-1260b; hsa-miR-30b-5p; hsa-miR-130b-5p; hsa-miR-331-3p; hsa-miR-30c-5p; hsa-miR-605-5p
Gene Ontology (miRWalk)	GO0008253 5 nucleotidase activity	enriched	0.0334747	6	hsa-miR-155-5p; hsa-miR-1-3p; hsa-miR-320a; hsa-miR-16-5p; hsa-miR-124-3p; hsa-miR-21-5p
Gene Ontology (miRWalk)	GO0008312 7s rna binding	enriched	0.0334747	7	hsa-miR-155-5p; hsa-miR-26b-5p; hsa-miR-320a; hsa-miR-16-5p; hsa-miR-92b-3p; hsa-miR-365a-3p; hsa-miR-1229-3p
Gene Ontology (miRWalk)	GO0008324 cation transmembrane transporter activity	enriched	0.0334747	9	hsa-miR-155-5p; hsa-miR-1-3p; hsa-miR-26b-5p; hsa-miR-16-5p; hsa-miR-92b-3p; hsa-miR-93-5p; hsa-miR-124-3p; hsa-miR-21-5p; hsa-miR-10a-5p
Gene Ontology (miRWalk)	GO0008408 3 5 exonuclease activity	enriched	0.0334747	13	hsa-miR-155-5p; hsa-miR-1-3p; hsa-miR-26b-5p; hsa-miR-103a-3p; hsa-miR-16-5p; hsa-miR-24-3p; hsa-miR-92b-3p; hsa-miR-93-5p; hsa-miR-124-3p; hsa-miR-21-5p; hsa-miR-1229-3p; hsa-miR-17-5p; hsa-miR-30c-5p
Gene Ontology (miRWalk)	GO0010332 response to gamma radiation	enriched	0.0334747	45	hsa-miR-29c-3p; hsa-let-7c-5p; hsa-miR-155-5p; hsa-let-7a-5p; hsa-let-7f-5p; hsa-miR-193a-5p; hsa-miR-30d-5p; hsa-miR-1-3p; hsa-miR-26b-5p; hsa-miR-320a; hsa-let-7g-5p; hsa-miR-103a-3p; hsa-miR-451a; hsa-miR-146a-5p; hsa-miR-16-5p; hsa-miR-20a-5p; hsa-miR-24-3p; hsa-miR-92b-3p; hsa-miR-29b-3p; hsa-miR-365a-3p; hsa-miR-181a-5p; hsa-miR-181d-5p; hsa-miR-181b-5p; hsa-miR-124-3p; hsa-miR-21-5p; hsa-miR-29a-3p; hsa-miR-26a-5p; hsa-miR-15a-5p; hsa-miR-449c-5p; hsa-miR-148a-3p; hsa-miR-25-3p; hsa-miR-17-5p; hsa-miR-18a-5p; hsa-miR-630; hsa-miR-15b-5p; hsa-miR-148b-3p; hsa-miR-504-5p; hsa-miR-92a-3p; hsa-miR-505-3p; hsa-miR-204-5p; hsa-miR-331-3p; hsa-miR-34b-3p; hsa-miR-30c-5p; hsa-miR-605-5p; hsa-miR-296-5p
Gene Ontology (miRWalk)	GO0016574 histone ubiquitination	enriched	0.0334747	24	hsa-miR-29c-3p; hsa-let-7c-5p; hsa-miR-155-5p; hsa-let-7a-5p; hsa-miR-324-3p; hsa-miR-1-3p; hsa-miR-877-5p; hsa-miR-320a; hsa-miR-106b-5p; hsa-miR-365a-3p; hsa-miR-181a-5p; hsa-miR-93-5p; hsa-miR-181b-5p; hsa-miR-124-3p; hsa-miR-21-5p; hsa-miR-29a-3p; hsa-miR-19a-3p; hsa-miR-17-5p; hsa-miR-29c-5p; hsa-miR-10a-5p; hsa-miR-149-5p; hsa-miR-615-3p; hsa-miR-346; hsa-miR-30c-5p
Gene Ontology (miRWalk)	GO0018105 peptidyl serine phosphorylation	enriched	0.0334747	51	hsa-miR-29c-3p; hsa-let-7c-5p; hsa-miR-155-5p; hsa-let-7a-5p; hsa-miR-324-3p; hsa-let-7f-5p; hsa-miR-1-3p; hsa-miR-877-5p; hsa-miR-26b-5p; hsa-miR-320a; hsa-let-7g-5p; hsa-miR-103a-3p; hsa-miR-760; hsa-miR-451a; hsa-miR-142-3p; hsa-miR-16-5p; hsa-miR-20a-5p; hsa-miR-24-3p; hsa-miR-92b-3p; hsa-miR-29b-3p; hsa-miR-365a-3p; hsa-miR-181a-5p; hsa-miR-181d-5p; hsa-miR-93-5p; hsa-miR-181b-5p; hsa-miR-124-3p; hsa-miR-21-5p; hsa-miR-29a-3p; hsa-miR-26a-5p; hsa-miR-19a-3p; hsa-miR-1229-3p; hsa-miR-15a-5p; hsa-miR-20b-5p; hsa-miR-148a-3p; hsa-miR-19b-3p; hsa-miR-25-3p; hsa-miR-766-3p; hsa-miR-17-5p; hsa-miR-18a-5p; hsa-miR-630; hsa-miR-10a-5p; hsa-miR-15b-5p; hsa-miR-148b-3p; hsa-miR-149-5p; hsa-miR-92a-3p; hsa-miR-615-3p; hsa-miR-505-3p; hsa-miR-204-5p; hsa-miR-331-3p; hsa-miR-197-3p; hsa-miR-296-5p

Gene Ontology (miRWalk)	GO0018107 peptidyl threonine phosphorylation	enriched	0.0334747	44	hsa-miR-29c-3p; hsa-let-7c-5p; hsa-miR-155-5p; hsa-let-7a-5p; hsa-miR-324-3p; hsa-miR-1-3p; hsa-miR-877-5p; hsa-miR-26b-5p; hsa-miR-103a-3p; hsa-miR-199b-5p; hsa-miR-760; hsa-miR-451a; hsa-miR-142-3p; hsa-miR-16-5p; hsa-miR-20a-5p; hsa-miR-24-3p; hsa-miR-92b-3p; hsa-miR-29b-3p; hsa-miR-365a-3p; hsa-miR-181a-5p; hsa-miR-181d-5p; hsa-miR-93-5p; hsa-miR-181b-5p; hsa-miR-124-3p; hsa-miR-21-5p; hsa-miR-29a-3p; hsa-miR-19a-3p; hsa-miR-15a-5p; hsa-miR-20b-5p; hsa-miR-148a-3p; hsa-miR-19b-3p; hsa-miR-17-5p; hsa-miR-18a-5p; hsa-miR-630; hsa-miR-10a-5p; hsa-miR-15b-5p; hsa-miR-149-5p; hsa-miR-23a-3p; hsa-miR-92a-3p; hsa-miR-615-3p; hsa-miR-204-5p; hsa-miR-331-3p; hsa-miR-197-3p; hsa-miR-296-5p
Gene Ontology (miRWalk)	GO0021542 dentate gyrus development	enriched	0.0334747	29	hsa-miR-29c-3p; hsa-let-7a-5p; hsa-miR-877-5p; hsa-miR-26b-5p; hsa-miR-103a-3p; hsa-miR-106b-5p; hsa-miR-107; hsa-miR-16-5p; hsa-miR-20a-5p; hsa-miR-92b-3p; hsa-miR-29b-3p; hsa-miR-181a-5p; hsa-miR-93-5p; hsa-miR-181b-5p; hsa-miR-124-3p; hsa-miR-21-5p; hsa-miR-29a-3p; hsa-miR-26a-5p; hsa-miR-19a-3p; hsa-miR-1229-3p; hsa-miR-19b-3p; hsa-miR-494-3p; hsa-miR-17-5p; hsa-miR-18a-5p; hsa-miR-148b-3p; hsa-miR-23a-3p; hsa-miR-504-5p; hsa-miR-615-3p; hsa-miR-34b-3p
Gene Ontology (miRWalk)	GO0030324 lung development	enriched	0.0334747	59	hsa-miR-29c-3p; hsa-let-7c-5p; hsa-miR-155-5p; hsa-let-7a-5p; hsa-let-7f-5p; hsa-miR-193a-5p; hsa-miR-1-3p; hsa-miR-877-5p; hsa-miR-26b-5p; hsa-miR-320a; hsa-let-7g-5p; hsa-miR-103a-3p; hsa-miR-199b-5p; hsa-miR-106b-5p; hsa-miR-18b-5p; hsa-miR-760; hsa-miR-142-3p; hsa-miR-107; hsa-miR-16-5p; hsa-miR-20a-5p; hsa-miR-92b-3p; hsa-miR-29b-3p; hsa-miR-154-5p; hsa-miR-181a-5p; hsa-miR-181d-5p; hsa-miR-93-5p; hsa-miR-181b-5p; hsa-miR-124-3p; hsa-miR-21-5p; hsa-miR-223-3p; hsa-miR-29a-3p; hsa-miR-26a-5p; hsa-miR-134-5p; hsa-miR-19a-3p; hsa-miR-15a-5p; hsa-miR-20b-5p; hsa-miR-148a-3p; hsa-miR-19b-3p; hsa-miR-520g-3p; hsa-miR-25-3p; hsa-miR-17-5p; hsa-miR-18a-5p; hsa-miR-10a-5p; hsa-miR-15b-5p; hsa-miR-148b-3p; hsa-miR-149-5p; hsa-miR-23a-3p; hsa-miR-504-5p; hsa-miR-92a-3p; hsa-miR-615-3p; hsa-miR-1260b; hsa-miR-30b-5p; hsa-miR-130b-5p; hsa-miR-1226-3p; hsa-miR-331-3p; hsa-miR-346; hsa-miR-34b-3p; hsa-miR-30c-5p; hsa-miR-328-3p
Gene Ontology (miRWalk)	GO0030890 positive regulation of b cell proliferation	enriched	0.0334747	46	hsa-miR-29c-3p; hsa-let-7c-5p; hsa-miR-155-5p; hsa-let-7a-5p; hsa-let-7f-5p; hsa-miR-1-3p; hsa-miR-26b-5p; hsa-miR-320a; hsa-let-7g-5p; hsa-miR-103a-3p; hsa-miR-106b-5p; hsa-let-7i-5p; hsa-miR-451a; hsa-miR-184; hsa-miR-146a-5p; hsa-miR-16-5p; hsa-miR-20a-5p; hsa-miR-29b-3p; hsa-miR-365a-3p; hsa-miR-181a-5p; hsa-miR-181d-5p; hsa-miR-93-5p; hsa-miR-181b-5p; hsa-miR-124-3p; hsa-miR-21-5p; hsa-miR-223-3p; hsa-miR-29a-3p; hsa-miR-1229-3p; hsa-miR-15a-5p; hsa-miR-520a-3p; hsa-miR-20b-5p; hsa-miR-148a-3p; hsa-miR-486-5p; hsa-miR-17-5p; hsa-miR-630; hsa-miR-572; hsa-miR-15b-5p; hsa-miR-149-5p; hsa-miR-92a-3p; hsa-miR-299-5p; hsa-miR-615-3p; hsa-miR-1260b; hsa-miR-30b-5p; hsa-miR-204-5p; hsa-miR-454-5p; hsa-miR-296-5p
Gene Ontology (miRWalk)	GO0031088 platelet dense granule membrane	enriched	0.0334747	6	hsa-miR-155-5p; hsa-miR-1-3p; hsa-miR-103a-3p; hsa-miR-16-5p; hsa-miR-124-3p; hsa-miR-21-5p

Gene Ontology (miRWalk)	GO0042059 negative regulation of epidermal growth factor receptor signaling pathway	enriched	0.0334747	43	hsa-miR-29c-3p; hsa-let-7c-5p; hsa-miR-155-5p; hsa-let-7a-5p; hsa-miR-324-3p; hsa-miR-1-3p; hsa-miR-877-5p; hsa-miR-26b-5p; hsa-miR-320a; hsa-miR-103a-3p; hsa-miR-106b-5p; hsa-miR-760; hsa-miR-1287-5p; hsa-miR-146a-5p; hsa-miR-16-5p; hsa-miR-20a-5p; hsa-miR-24-3p; hsa-miR-92b-3p; hsa-miR-29b-3p; hsa-miR-181a-5p; hsa-miR-93-5p; hsa-miR-181b-5p; hsa-miR-124-3p; hsa-miR-21-5p; hsa-miR-29a-3p; hsa-miR-26a-5p; hsa-miR-1229-3p; hsa-miR-148a-3p; hsa-miR-1224-5p; hsa-miR-19b-3p; hsa-miR-17-5p; hsa-miR-18a-5p; hsa-miR-15b-5p; hsa-miR-92a-3p; hsa-miR-615-3p; hsa-miR-30b-5p; hsa-miR-1226-3p; hsa-miR-331-3p; hsa-miR-1914-5p; hsa-miR-30c-5p; hsa-miR-296-5p; hsa-miR-4326; hsa-miR-328-3p
Gene Ontology (miRWalk)	GO0042327 positive regulation of phosphorylation	enriched	0.0334747	19	hsa-miR-155-5p; hsa-let-7a-5p; hsa-miR-1-3p; hsa-miR-877-5p; hsa-miR-320a; hsa-miR-103a-3p; hsa-miR-451a; hsa-miR-146a-5p; hsa-miR-16-5p; hsa-miR-20a-5p; hsa-miR-181a-5p; hsa-miR-124-3p; hsa-miR-21-5p; hsa-miR-26a-5p; hsa-miR-148a-3p; hsa-miR-940; hsa-miR-17-5p; hsa-miR-15b-5p; hsa-miR-92a-3p
Gene Ontology (miRWalk)	GO0042698 ovulation cycle	enriched	0.0334747	9	hsa-miR-155-5p; hsa-let-7a-5p; hsa-miR-1-3p; hsa-miR-26b-5p; hsa-miR-199b-5p; hsa-miR-146a-5p; hsa-miR-124-3p; hsa-miR-21-5p; hsa-miR-504-5p
Gene Ontology (miRWalk)	GO0043537 negative regulation of blood vessel endothelial cell migration	enriched	0.0334747	14	hsa-miR-155-5p; hsa-let-7a-5p; hsa-miR-1-3p; hsa-miR-26b-5p; hsa-miR-103a-3p; hsa-miR-142-3p; hsa-miR-16-5p; hsa-miR-20a-5p; hsa-miR-24-3p; hsa-miR-124-3p; hsa-miR-21-5p; hsa-miR-17-5p; hsa-miR-148b-3p; hsa-miR-92a-3p
Gene Ontology (miRWalk)	GO0043627 response to estrogen stimulus	enriched	0.0334747	53	hsa-miR-29c-3p; hsa-miR-155-5p; hsa-let-7a-5p; hsa-let-7f-5p; hsa-miR-1-3p; hsa-miR-877-5p; hsa-miR-26b-5p; hsa-miR-320a; hsa-miR-103a-3p; hsa-miR-106b-5p; hsa-miR-18b-5p; hsa-miR-760; hsa-miR-451a; hsa-miR-146a-5p; hsa-miR-16-5p; hsa-miR-20a-5p; hsa-miR-24-3p; hsa-miR-92b-3p; hsa-miR-29b-3p; hsa-miR-671-5p; hsa-miR-365a-3p; hsa-miR-181a-5p; hsa-miR-181d-5p; hsa-miR-93-5p; hsa-miR-181b-5p; hsa-miR-124-3p; hsa-miR-21-5p; hsa-miR-29a-3p; hsa-miR-26a-5p; hsa-miR-99b-3p; hsa-miR-19a-3p; hsa-miR-1229-3p; hsa-miR-15a-5p; hsa-miR-20b-5p; hsa-miR-148a-3p; hsa-miR-19b-3p; hsa-miR-25-3p; hsa-miR-766-3p; hsa-miR-17-5p; hsa-miR-18a-5p; hsa-miR-630; hsa-miR-10a-5p; hsa-miR-15b-5p; hsa-miR-148b-3p; hsa-miR-149-5p; hsa-miR-504-5p; hsa-miR-92a-3p; hsa-miR-615-3p; hsa-miR-130b-5p; hsa-miR-204-5p; hsa-miR-34b-3p; hsa-miR-30c-5p; hsa-miR-296-5p
Gene Ontology (miRWalk)	GO0043983 histone h4 k12 acetylation	enriched	0.0334747	9	hsa-miR-155-5p; hsa-miR-324-3p; hsa-miR-26b-5p; hsa-miR-320a; hsa-miR-106b-5p; hsa-miR-24-3p; hsa-miR-124-3p; hsa-miR-21-5p; hsa-miR-10a-5p
Gene Ontology (miRWalk)	GO0045348 positive regulation of mhc class ii biosynthetic process	enriched	0.0334747	14	hsa-let-7c-5p; hsa-miR-1-3p; hsa-miR-26b-5p; hsa-let-7i-5p; hsa-miR-146a-5p; hsa-miR-16-5p; hsa-miR-29b-3p; hsa-miR-181a-5p; hsa-miR-181b-5p; hsa-miR-124-3p; hsa-miR-21-5p; hsa-miR-15a-5p; hsa-miR-15b-5p; hsa-miR-331-3p
Gene Ontology (miRWalk)	GO0045454 cell redox homeostasis	enriched	0.0334747	27	hsa-miR-29c-3p; hsa-let-7c-5p; hsa-miR-155-5p; hsa-let-7a-5p; hsa-miR-324-3p; hsa-miR-1-3p; hsa-miR-877-5p; hsa-miR-26b-5p; hsa-miR-103a-3p; hsa-miR-106b-5p; hsa-miR-16-5p; hsa-miR-20a-5p; hsa-miR-92b-3p; hsa-miR-671-5p; hsa-miR-93-5p; hsa-miR-124-3p; hsa-miR-21-5p; hsa-miR-26a-5p; hsa-miR-1229-3p; hsa-miR-25-3p; hsa-miR-18a-5p; hsa-miR-149-5p; hsa-miR-504-5p; hsa-miR-92a-3p; hsa-miR-615-3p; hsa-miR-197-3p; hsa-miR-30c-5p
Gene Ontology (miRWalk)	GO0045765 regulation of angiogenesis	enriched	0.0334747	19	hsa-miR-155-5p; hsa-let-7a-5p; hsa-miR-1-3p; hsa-miR-26b-5p; hsa-miR-320a; hsa-miR-103a-3p; hsa-miR-199b-5p; hsa-miR-548d-3p; hsa-miR-16-5p; hsa-miR-92b-3p; hsa-miR-365a-3p; hsa-miR-124-3p; hsa-miR-21-5p; hsa-miR-26a-5p; hsa-miR-940; hsa-miR-148b-3p; hsa-miR-92a-3p; hsa-miR-1226-3p; hsa-miR-331-3p

Gene Ontology (miRWalk)	GO0046324 regulation of glucose import	enriched	0.0334747	6	hsa-miR-155-5p; hsa-miR-324-3p; hsa-miR-877-5p; hsa-miR-103a-3p; hsa-miR-16-5p; hsa-miR-21-5p
Gene Ontology (miRWalk)	GO0046856 phosphatidylinositol dephosphorylation	enriched	0.0334747	26	hsa-miR-155-5p; hsa-miR-324-3p; hsa-miR-877-5p; hsa-miR-26b-5p; hsa-miR-320a; hsa-miR-103a-3p; hsa-miR-106b-5p; hsa-miR-107; hsa-miR-16-5p; hsa-miR-20a-5p; hsa-miR-29b-3p; hsa-miR-181a-5p; hsa-miR-93-5p; hsa-miR-181b-5p; hsa-miR-21-5p; hsa-miR-29a-3p; hsa-miR-26a-5p; hsa-miR-19a-3p; hsa-miR-19b-3p; hsa-miR-494-3p; hsa-miR-17-5p; hsa-miR-18a-5p; hsa-miR-10a-5p; hsa-miR-15b-5p; hsa-miR-23a-3p; hsa-miR-92a-3p
Gene Ontology (miRWalk)	GO0048699 generation of neurons	enriched	0.0334747	19	hsa-miR-29c-3p; hsa-let-7a-5p; hsa-miR-324-3p; hsa-miR-26b-5p; hsa-miR-320a; hsa-miR-103a-3p; hsa-miR-107; hsa-miR-16-5p; hsa-miR-92b-3p; hsa-miR-29b-3p; hsa-miR-124-3p; hsa-miR-21-5p; hsa-miR-29a-3p; hsa-miR-26a-5p; hsa-miR-19b-3p; hsa-miR-148b-3p; hsa-miR-92a-3p; hsa-miR-615-3p; hsa-miR-34b-3p
Gene Ontology (miRWalk)	GO0050775 positive regulation of dendrite morphogenesis	enriched	0.0334747	19	hsa-miR-29c-3p; hsa-miR-155-5p; hsa-let-7f-5p; hsa-miR-30d-5p; hsa-miR-1-3p; hsa-miR-26b-5p; hsa-miR-320a; hsa-miR-103a-3p; hsa-miR-16-5p; hsa-miR-92b-3p; hsa-miR-93-5p; hsa-miR-124-3p; hsa-miR-21-5p; hsa-miR-26a-5p; hsa-miR-92a-3p; hsa-miR-30b-5p; hsa-miR-34b-3p; hsa-miR-30c-5p; hsa-miR-1296-5p
Gene Ontology (miRWalk)	GO0050777 negative regulation of immune response	enriched	0.0334747	6	hsa-miR-29c-3p; hsa-miR-155-5p; hsa-miR-26b-5p; hsa-miR-24-3p; hsa-miR-29b-3p; hsa-miR-21-5p
Gene Ontology (miRWalk)	GO0050830 defense response to gram positive bacterium	enriched	0.0334747	15	hsa-miR-155-5p; hsa-let-7a-5p; hsa-miR-1-3p; hsa-miR-26b-5p; hsa-miR-106b-5p; hsa-miR-146a-5p; hsa-miR-16-5p; hsa-miR-365a-3p; hsa-miR-124-3p; hsa-miR-21-5p; hsa-miR-26a-5p; hsa-miR-19a-3p; hsa-miR-19b-3p; hsa-miR-23a-3p; hsa-miR-105-5p
Gene Ontology (miRWalk)	GO0052033 pathogen associated molecular pattern dependent induction by symbiont of host innate immune response	enriched	0.0334747	6	hsa-miR-1-3p; hsa-miR-26b-5p; hsa-let-7i-5p; hsa-miR-146a-5p; hsa-miR-124-3p; hsa-miR-21-5p
Gene Ontology (miRWalk)	GO0060052 neurofilament cytoskeleton organization	enriched	0.0334747	9	hsa-miR-155-5p; hsa-let-7a-5p; hsa-miR-26b-5p; hsa-miR-106b-5p; hsa-miR-142-3p; hsa-miR-93-5p; hsa-miR-124-3p; hsa-miR-21-5p; hsa-miR-197-3p
Gene Ontology (miRWalk)	GO0005771 multivesicular body	enriched	0.0335133	14	hsa-miR-155-5p; hsa-miR-1-3p; hsa-miR-26b-5p; hsa-miR-320a; hsa-miR-1227-3p; hsa-miR-16-5p; hsa-miR-20a-5p; hsa-miR-92b-3p; hsa-miR-29b-3p; hsa-miR-93-5p; hsa-miR-223-3p; hsa-miR-92a-3p; hsa-miR-615-3p; hsa-miR-331-3p
Gene Ontology (miRWalk)	GO0007162 negative regulation of cell adhesion	enriched	0.0335133	12	hsa-miR-155-5p; hsa-miR-1-3p; hsa-miR-26b-5p; hsa-miR-760; hsa-miR-16-5p; hsa-miR-24-3p; hsa-miR-93-5p; hsa-miR-124-3p; hsa-miR-21-5p; hsa-miR-26a-5p; hsa-miR-92a-3p; hsa-miR-30c-5p
Gene Ontology (miRWalk)	GO0007422 peripheral nervous system development	enriched	0.0335133	14	hsa-miR-155-5p; hsa-let-7a-5p; hsa-miR-1-3p; hsa-miR-26b-5p; hsa-miR-103a-3p; hsa-miR-16-5p; hsa-miR-24-3p; hsa-miR-93-5p; hsa-miR-124-3p; hsa-miR-21-5p; hsa-miR-223-3p; hsa-miR-148b-3p; hsa-miR-615-3p; hsa-miR-1260b
Gene Ontology (miRWalk)	GO0032481 positive regulation of type i interferon production	enriched	0.0335133	14	hsa-miR-155-5p; hsa-let-7a-5p; hsa-miR-26b-5p; hsa-miR-320a; hsa-miR-146a-5p; hsa-miR-16-5p; hsa-miR-24-3p; hsa-miR-93-5p; hsa-miR-124-3p; hsa-miR-21-5p; hsa-miR-223-3p; hsa-miR-15a-5p; hsa-miR-92a-3p; hsa-miR-615-3p

Gene Ontology (miRWalk)	GO0048589 developmental growth	enriched	0.0335133	40	hsa-miR-29c-3p; hsa-miR-155-5p; hsa-let-7a-5p; hsa-miR-324-3p; hsa-miR-30d-5p; hsa-miR-1-3p; hsa-miR-26b-5p; hsa-miR-320a; hsa-miR-103a-3p; hsa-miR-451a; hsa-miR-146a-5p; hsa-miR-16-5p; hsa-miR-20a-5p; hsa-miR-29b-3p; hsa-miR-365a-3p; hsa-miR-181a-5p; hsa-miR-181d-5p; hsa-miR-93-5p; hsa-miR-181b-5p; hsa-miR-124-3p; hsa-miR-21-5p; hsa-miR-29a-3p; hsa-miR-26a-5p; hsa-miR-99b-3p; hsa-miR-19a-3p; hsa-miR-15a-5p; hsa-miR-148a-3p; hsa-miR-19b-3p; hsa-miR-17-5p; hsa-miR-18a-5p; hsa-miR-630; hsa-miR-15b-5p; hsa-miR-148b-3p; hsa-miR-92a-3p; hsa-miR-483-3p; hsa-miR-30b-5p; hsa-miR-204-5p; hsa-miR-331-3p; hsa-miR-197-3p; hsa-miR-296-5p
Gene Ontology (miRWalk)	GO0072089 stem cell proliferation	enriched	0.0335133	7	hsa-miR-155-5p; hsa-miR-103a-3p; hsa-miR-106b-5p; hsa-miR-451a; hsa-miR-16-5p; hsa-miR-24-3p; hsa-miR-296-5p
Gene Ontology (miRWalk)	GO0045664 regulation of neuron differentiation	enriched	0.0336236	28	hsa-miR-29c-3p; hsa-let-7c-5p; hsa-miR-155-5p; hsa-let-7a-5p; hsa-miR-324-3p; hsa-miR-877-5p; hsa-miR-26b-5p; hsa-miR-320a; hsa-miR-103a-3p; hsa-miR-106b-5p; hsa-miR-107; hsa-miR-16-5p; hsa-miR-20a-5p; hsa-miR-29b-3p; hsa-miR-154-5p; hsa-miR-124-3p; hsa-miR-29a-3p; hsa-miR-26a-5p; hsa-miR-19a-3p; hsa-miR-15a-5p; hsa-miR-19b-3p; hsa-miR-25-3p; hsa-miR-17-5p; hsa-miR-18a-5p; hsa-miR-10a-5p; hsa-miR-149-5p; hsa-miR-615-3p; hsa-miR-30c-5p
Gene Ontology (miRWalk)	GO2000036 regulation of stem cell maintenance	enriched	0.0337412	9	hsa-let-7c-5p; hsa-let-7a-5p; hsa-let-7f-5p; hsa-let-7g-5p; hsa-miR-103a-3p; hsa-miR-760; hsa-miR-16-5p; hsa-miR-26a-5p; hsa-miR-331-3p
Pathways (miRWalk)	P00023 General transcription regulation	enriched	0.0341586	24	hsa-miR-155-5p; hsa-let-7a-5p; hsa-miR-324-3p; hsa-let-7f-5p; hsa-miR-30d-5p; hsa-miR-1-3p; hsa-miR-877-5p; hsa-miR-26b-5p; hsa-miR-320a; hsa-miR-16-5p; hsa-miR-24-3p; hsa-miR-92b-3p; hsa-miR-181a-5p; hsa-miR-93-5p; hsa-miR-21-5p; hsa-miR-15a-5p; hsa-miR-19b-3p; hsa-miR-766-3p; hsa-miR-17-5p; hsa-miR-10a-5p; hsa-miR-149-5p; hsa-miR-92a-3p; hsa-miR-615-3p; hsa-miR-1296-5p
Pathways (miRWalk)	hsa05414 Dilated cardiomyopathy	enriched	0.0341586	40	hsa-let-7c-5p; hsa-miR-155-5p; hsa-let-7a-5p; hsa-miR-324-3p; hsa-let-7f-5p; hsa-miR-1-3p; hsa-miR-877-5p; hsa-miR-26b-5p; hsa-miR-320a; hsa-miR-103a-3p; hsa-miR-1227-3p; hsa-miR-1295a; hsa-miR-142-3p; hsa-miR-16-5p; hsa-miR-24-3p; hsa-miR-671-5p; hsa-miR-365a-3p; hsa-miR-93-5p; hsa-miR-124-3p; hsa-miR-21-5p; hsa-miR-29a-3p; hsa-miR-26a-5p; hsa-miR-99b-3p; hsa-miR-1229-3p; hsa-miR-940; hsa-miR-19b-3p; hsa-miR-25-3p; hsa-miR-17-5p; hsa-miR-10a-5p; hsa-miR-15b-5p; hsa-miR-148b-3p; hsa-miR-149-5p; hsa-miR-92a-3p; hsa-miR-615-3p; hsa-miR-204-5p; hsa-miR-1226-3p; hsa-miR-331-3p; hsa-miR-197-3p; hsa-miR-30c-5p; hsa-miR-1296-5p
Gene Ontology (miRWalk)	GO0071310 cellular response to organic substance	enriched	0.03417	38	hsa-miR-29c-3p; hsa-let-7c-5p; hsa-miR-155-5p; hsa-let-7a-5p; hsa-miR-324-3p; hsa-miR-30d-5p; hsa-miR-1-3p; hsa-miR-26b-5p; hsa-miR-320a; hsa-miR-103a-3p; hsa-miR-18b-5p; hsa-miR-451a; hsa-miR-16-5p; hsa-miR-20a-5p; hsa-miR-29b-3p; hsa-miR-365a-3p; hsa-miR-181a-5p; hsa-miR-181d-5p; hsa-miR-93-5p; hsa-miR-181b-5p; hsa-miR-124-3p; hsa-miR-21-5p; hsa-miR-29a-3p; hsa-miR-19a-3p; hsa-miR-15a-5p; hsa-miR-148a-3p; hsa-miR-19b-3p; hsa-miR-17-5p; hsa-miR-630; hsa-miR-15b-5p; hsa-miR-148b-3p; hsa-miR-149-5p; hsa-miR-23a-3p; hsa-miR-504-5p; hsa-miR-615-3p; hsa-miR-204-5p; hsa-miR-211-5p; hsa-miR-296-5p

Diseases (published studies about miRNA profiles from peripheral blood)	lung cancer deregulated	depleted	0.0342329	87	hsa-miR-155-5p; hsa-miR-193b-5p; hsa-let-7a-5p; hsa-miR-324-3p; hsa-miR-193a-5p; hsa-miR-30d-5p; hsa-miR-498; hsa-miR-877-5p; hsa-miR-26b-5p; hsa-miR-520d-3p; hsa-miR-103a-3p; hsa-miR-1290; hsa-miR-1227-3p; hsa-miR-199b-5p; hsa-miR-106b-5p; hsa-miR-548d-3p; hsa-miR-142-3p; hsa-miR-425-3p; hsa-miR-107; hsa-miR-16-5p; hsa-miR-1299; hsa-miR-20a-5p; hsa-miR-92b-3p; hsa-miR-548i; hsa-miR-765; hsa-miR-154-5p; hsa-miR-939-5p; hsa-miR-365a-3p; hsa-miR-374a-3p; hsa-miR-181a-5p; hsa-miR-93-5p; hsa-miR-1207-5p; hsa-miR-223-3p; hsa-miR-29a-3p; hsa-miR-26a-5p; hsa-miR-1914-3p; hsa-miR-20b-5p; hsa-miR-1275; hsa-miR-148a-3p; hsa-miR-320c; hsa-miR-19b-3p; hsa-miR-135a-3p; hsa-miR-766-3p; hsa-miR-17-5p; hsa-miR-29c-5p; hsa-miR-576-5p; hsa-miR-581; hsa-miR-18a-5p; hsa-miR-129-1-3p; hsa-miR-490-5p; hsa-miR-23a-3p; hsa-miR-1281; hsa-miR-26b-3p; hsa-miR-1249-3p; hsa-miR-802; hsa-miR-125b-1-3p; hsa-miR-1225-5p; hsa-miR-521; hsa-miR-640; hsa-miR-1260a; hsa-miR-629-3p; hsa-miR-483-3p; hsa-miR-218-1-3p; hsa-miR-574-3p; hsa-miR-582-3p; hsa-miR-30b-5p; hsa-miR-130b-5p; hsa-let-7g-3p; hsa-miR-204-5p; hsa-miR-181a-2-3p; hsa-miR-337-3p; hsa-let-7e-3p; hsa-miR-1226-3p; hsa-miR-331-3p; hsa-miR-1236-3p; hsa-miR-197-3p; hsa-miR-346; hsa-miR-744-3p; hsa-miR-631; hsa-miR-1909-5p; hsa-miR-605-5p; hsa-let-7d-3p; hsa-miR-566; hsa-miR-328-3p; hsa-miR-7-2-3p; hsa-miR-885-5p; hsa-miR-668-3p
Gene Ontology (miRWalk)	GO0006346 methylation dependent chromatin silencing	enriched	0.0345512	14	hsa-miR-29c-3p; hsa-let-7c-5p; hsa-miR-155-5p; hsa-miR-16-5p; hsa-miR-29b-3p; hsa-miR-181a-5p; hsa-miR-93-5p; hsa-miR-181b-5p; hsa-miR-124-3p; hsa-miR-21-5p; hsa-miR-29a-3p; hsa-miR-148a-3p; hsa-miR-615-3p; hsa-miR-331-3p
Gene Ontology (miRWalk)	GO0007275 multicellular organismal development	enriched	0.0345512	66	hsa-miR-29c-3p; hsa-let-7c-5p; hsa-miR-155-5p; hsa-let-7a-5p; hsa-miR-324-3p; hsa-let-7f-5p; hsa-miR-30d-5p; hsa-miR-1-3p; hsa-miR-876-3p; hsa-miR-877-5p; hsa-miR-26b-5p; hsa-miR-320a; hsa-let-7g-5p; hsa-miR-103a-3p; hsa-miR-1227-3p; hsa-miR-106b-5p; hsa-miR-18b-5p; hsa-miR-760; hsa-miR-146a-5p; hsa-miR-107; hsa-miR-16-5p; hsa-miR-20a-5p; hsa-miR-24-3p; hsa-miR-92b-3p; hsa-miR-29b-3p; hsa-miR-671-5p; hsa-miR-365a-3p; hsa-miR-181a-5p; hsa-miR-93-5p; hsa-miR-181b-5p; hsa-miR-124-3p; hsa-miR-21-5p; hsa-miR-223-3p; hsa-miR-29a-3p; hsa-miR-26a-5p; hsa-miR-99b-3p; hsa-miR-19a-3p; hsa-miR-1229-3p; hsa-miR-15a-5p; hsa-miR-148a-3p; hsa-miR-940; hsa-miR-320c; hsa-miR-19b-3p; hsa-miR-25-3p; hsa-miR-17-5p; hsa-miR-18a-5p; hsa-miR-10a-5p; hsa-miR-15b-5p; hsa-miR-148b-3p; hsa-miR-149-5p; hsa-miR-23a-3p; hsa-miR-504-5p; hsa-miR-92a-3p; hsa-miR-3679-3p; hsa-miR-615-3p; hsa-miR-1260a; hsa-miR-483-3p; hsa-miR-1260b; hsa-miR-30b-5p; hsa-miR-130b-5p; hsa-miR-204-5p; hsa-miR-1226-3p; hsa-miR-331-3p; hsa-miR-346; hsa-miR-34b-3p; hsa-miR-605-5p
Pathways (miRWalk)	hsa00760 Nicotinate and nicotinamide metabolism	enriched	0.0345936	14	hsa-miR-155-5p; hsa-miR-1-3p; hsa-miR-26b-5p; hsa-miR-320a; hsa-miR-106b-5p; hsa-miR-16-5p; hsa-miR-29b-3p; hsa-miR-181a-5p; hsa-miR-124-3p; hsa-miR-21-5p; hsa-miR-92a-3p; hsa-miR-615-3p; hsa-miR-1226-3p; hsa-miR-1296-5p
Pathways (miRWalk)	hsa00830 Retinol metabolism	enriched	0.0345936	14	hsa-miR-155-5p; hsa-miR-26b-5p; hsa-miR-103a-3p; hsa-miR-106b-5p; hsa-miR-142-3p; hsa-miR-107; hsa-miR-16-5p; hsa-miR-181d-5p; hsa-miR-124-3p; hsa-miR-21-5p; hsa-miR-19b-3p; hsa-miR-10a-5p; hsa-miR-92a-3p; hsa-miR-615-3p

Pathways (miRWalk)	WP410 Diurnally regulated genes with circadian orthologs	enriched	0.0346859	38	hsa-let-7c-5p; hsa-miR-155-5p; hsa-let-7a-5p; hsa-miR-324-3p; hsa-let-7f-5p; hsa-miR-877-5p; hsa-miR-26b-5p; hsa-miR-320a; hsa-miR-103a-3p; hsa-miR-106b-5p; hsa-miR-18b-5p; hsa-miR-142-3p; hsa-miR-16-5p; hsa-miR-20a-5p; hsa-miR-24-3p; hsa-miR-92b-3p; hsa-miR-365a-3p; hsa-miR-181a-5p; hsa-miR-93-5p; hsa-miR-124-3p; hsa-miR-21-5p; hsa-miR-26a-5p; hsa-miR-1229-3p; hsa-miR-15a-5p; hsa-miR-148a-3p; hsa-miR-494-3p; hsa-miR-25-3p; hsa-miR-17-5p; hsa-miR-10a-5p; hsa-miR-15b-5p; hsa-miR-148b-3p; hsa-miR-149-5p; hsa-miR-92a-3p; hsa-miR-615-3p; hsa-miR-505-3p; hsa-miR-30b-5p; hsa-miR-130b-5p; hsa-miR-331-3p
Gene Ontology (miRWalk)	GO0043029 t cell homeostasis	enriched	0.0346929	35	hsa-miR-29c-3p; hsa-let-7c-5p; hsa-let-7a-5p; hsa-miR-324-3p; hsa-let-7f-5p; hsa-miR-30d-5p; hsa-miR-1-3p; hsa-miR-26b-5p; hsa-miR-103a-3p; hsa-miR-451a; hsa-miR-16-5p; hsa-miR-20a-5p; hsa-miR-24-3p; hsa-miR-29b-3p; hsa-miR-365a-3p; hsa-miR-181a-5p; hsa-miR-181d-5p; hsa-miR-181b-5p; hsa-miR-124-3p; hsa-miR-21-5p; hsa-miR-29a-3p; hsa-miR-19a-3p; hsa-miR-1229-3p; hsa-miR-15a-5p; hsa-miR-148a-3p; hsa-miR-19b-3p; hsa-miR-494-3p; hsa-miR-25-3p; hsa-miR-17-5p; hsa-miR-630; hsa-miR-15b-5p; hsa-miR-615-3p; hsa-miR-204-5p; hsa-miR-331-3p; hsa-miR-296-5p
Gene Ontology (miRWalk)	GO0043966 histone h3 acetylation	enriched	0.0346929	32	hsa-let-7c-5p; hsa-miR-155-5p; hsa-let-7a-5p; hsa-miR-324-3p; hsa-miR-1-3p; hsa-miR-26b-5p; hsa-miR-320a; hsa-let-7g-5p; hsa-miR-103a-3p; hsa-miR-106b-5p; hsa-miR-146a-5p; hsa-miR-16-5p; hsa-miR-24-3p; hsa-miR-92b-3p; hsa-miR-181a-5p; hsa-miR-93-5p; hsa-miR-181b-5p; hsa-miR-124-3p; hsa-miR-21-5p; hsa-miR-26a-5p; hsa-miR-19a-3p; hsa-miR-19b-3p; hsa-miR-25-3p; hsa-miR-17-5p; hsa-miR-18a-5p; hsa-miR-10a-5p; hsa-miR-15b-5p; hsa-miR-92a-3p; hsa-miR-615-3p; hsa-miR-331-3p; hsa-miR-1236-3p; hsa-miR-30c-5p
Gene Ontology (miRWalk)	GO0060445 branching involved in salivary gland morphogenesis	enriched	0.0346929	10	hsa-miR-155-5p; hsa-miR-1-3p; hsa-miR-26b-5p; hsa-miR-320a; hsa-miR-20a-5p; hsa-miR-181b-5p; hsa-miR-124-3p; hsa-miR-29a-3p; hsa-miR-1229-3p; hsa-miR-10a-5p
Gene Ontology (miRWalk)	GO0005245 voltage gated calcium channel activity	enriched	0.0347036	16	hsa-let-7c-5p; hsa-miR-155-5p; hsa-miR-324-3p; hsa-miR-1-3p; hsa-miR-26b-5p; hsa-miR-103a-3p; hsa-miR-106b-5p; hsa-miR-142-3p; hsa-miR-16-5p; hsa-miR-93-5p; hsa-miR-124-3p; hsa-miR-21-5p; hsa-miR-17-5p; hsa-miR-148b-3p; hsa-miR-92a-3p; hsa-miR-331-3p
Gene Ontology (miRWalk)	GO0016580 sin3 complex	enriched	0.0347036	21	hsa-miR-29c-3p; hsa-let-7c-5p; hsa-miR-155-5p; hsa-let-7a-5p; hsa-miR-1-3p; hsa-miR-26b-5p; hsa-miR-320a; hsa-miR-760; hsa-miR-142-3p; hsa-miR-16-5p; hsa-miR-24-3p; hsa-miR-92b-3p; hsa-miR-671-5p; hsa-miR-181a-5p; hsa-miR-10a-5p; hsa-miR-148b-3p; hsa-miR-149-5p; hsa-miR-92a-3p; hsa-miR-615-3p; hsa-miR-337-3p; hsa-miR-197-3p
Gene Ontology (miRWalk)	GO0043032 positive regulation of macrophage activation	enriched	0.0347036	15	hsa-miR-155-5p; hsa-let-7a-5p; hsa-let-7f-5p; hsa-miR-1-3p; hsa-miR-26b-5p; hsa-let-7g-5p; hsa-let-7i-5p; hsa-miR-16-5p; hsa-miR-20a-5p; hsa-miR-24-3p; hsa-miR-21-5p; hsa-miR-17-5p; hsa-miR-92a-3p; hsa-miR-331-3p; hsa-miR-197-3p
Gene Ontology (miRWalk)	GO0043596 nuclear replication fork	enriched	0.0347036	16	hsa-let-7c-5p; hsa-miR-155-5p; hsa-let-7a-5p; hsa-miR-324-3p; hsa-let-7f-5p; hsa-miR-1-3p; hsa-miR-26b-5p; hsa-miR-320a; hsa-miR-16-5p; hsa-miR-24-3p; hsa-miR-124-3p; hsa-miR-21-5p; hsa-miR-18a-5p; hsa-miR-92a-3p; hsa-miR-615-3p; hsa-miR-30b-5p
Gene Ontology (miRWalk)	GO0045840 positive regulation of mitosis	enriched	0.0347036	16	hsa-miR-155-5p; hsa-let-7a-5p; hsa-miR-1-3p; hsa-miR-877-5p; hsa-miR-26b-5p; hsa-miR-320a; hsa-miR-760; hsa-miR-142-3p; hsa-miR-16-5p; hsa-miR-93-5p; hsa-miR-124-3p; hsa-miR-21-5p; hsa-miR-92a-3p; hsa-miR-204-5p; hsa-miR-346; hsa-miR-34b-3p

Gene Ontology (miRWalk)	GO0004402 histone acetyltransferase activity	enriched	0.0347977	39	hsa-let-7c-5p; hsa-miR-155-5p; hsa-let-7a-5p; hsa-miR-324-3p; hsa-miR-1-3p; hsa-miR-26b-5p; hsa-miR-320a; hsa-miR-103a-3p; hsa-miR-106b-5p; hsa-miR-146a-5p; hsa-miR-142-3p; hsa-miR-16-5p; hsa-miR-20a-5p; hsa-miR-24-3p; hsa-miR-92b-3p; hsa-miR-29b-3p; hsa-miR-181a-5p; hsa-miR-181d-5p; hsa-miR-93-5p; hsa-miR-181b-5p; hsa-miR-124-3p; hsa-miR-21-5p; hsa-miR-26a-5p; hsa-miR-19a-3p; hsa-miR-1229-3p; hsa-miR-19b-3p; hsa-miR-25-3p; hsa-miR-766-3p; hsa-miR-17-5p; hsa-miR-18a-5p; hsa-miR-15b-5p; hsa-miR-149-5p; hsa-miR-92a-3p; hsa-miR-615-3p; hsa-miR-130b-5p; hsa-miR-331-3p; hsa-miR-1236-3p; hsa-miR-197-3p; hsa-miR-30c-5p
Gene Ontology (miRWalk)	GO0045779 negative regulation of bone resorption	enriched	0.0347977	6	hsa-miR-155-5p; hsa-miR-26b-5p; hsa-miR-24-3p; hsa-miR-124-3p; hsa-miR-21-5p; hsa-miR-29a-3p
Pathways (miRWalk)	P00009 Axon guidance mediated by netrin	enriched	0.0348148	28	hsa-miR-29c-3p; hsa-miR-155-5p; hsa-let-7a-5p; hsa-miR-324-3p; hsa-miR-1-3p; hsa-miR-26b-5p; hsa-miR-320a; hsa-miR-106b-5p; hsa-let-7i-5p; hsa-miR-184; hsa-miR-142-3p; hsa-miR-92b-3p; hsa-miR-29b-3p; hsa-miR-93-5p; hsa-miR-124-3p; hsa-miR-21-5p; hsa-miR-29a-3p; hsa-miR-1914-3p; hsa-miR-17-5p; hsa-miR-15b-5p; hsa-miR-148b-3p; hsa-miR-92a-3p; hsa-miR-615-3p; hsa-miR-331-3p; hsa-miR-346; hsa-miR-744-3p; hsa-miR-30c-5p; hsa-miR-1296-5p
Pathways (miRWalk)	P00028 Heterotrimeric G protein signaling pathway rod outer segment phototransduction	enriched	0.0348148	18	hsa-miR-29c-3p; hsa-miR-155-5p; hsa-miR-1-3p; hsa-miR-26b-5p; hsa-miR-320a; hsa-miR-103a-3p; hsa-miR-16-5p; hsa-miR-92b-3p; hsa-miR-365a-3p; hsa-miR-93-5p; hsa-miR-124-3p; hsa-miR-21-5p; hsa-miR-148a-3p; hsa-miR-19b-3p; hsa-miR-17-5p; hsa-miR-148b-3p; hsa-miR-23a-3p; hsa-miR-331-3p
Pathways (miRWalk)	P02738 De novo purine biosynthesis	enriched	0.0348148	28	hsa-miR-155-5p; hsa-let-7a-5p; hsa-miR-324-3p; hsa-miR-1-3p; hsa-miR-26b-5p; hsa-miR-320a; hsa-let-7g-5p; hsa-let-7i-5p; hsa-miR-142-3p; hsa-miR-16-5p; hsa-miR-20a-5p; hsa-miR-24-3p; hsa-miR-29b-3p; hsa-miR-93-5p; hsa-miR-124-3p; hsa-miR-29a-3p; hsa-miR-26a-5p; hsa-miR-1229-3p; hsa-miR-19b-3p; hsa-miR-17-5p; hsa-miR-10a-5p; hsa-miR-148b-3p; hsa-miR-23a-3p; hsa-miR-92a-3p; hsa-miR-615-3p; hsa-miR-30b-5p; hsa-miR-197-3p; hsa-miR-30c-5p
Pathways (miRWalk)	P02769 Purine metabolism	enriched	0.0348148	4	hsa-miR-155-5p; hsa-miR-1-3p; hsa-miR-26b-5p; hsa-miR-124-3p
Pathways (miRWalk)	P05730 Endogenous cannabinoid signaling	enriched	0.0348148	16	hsa-miR-29c-3p; hsa-miR-155-5p; hsa-miR-1-3p; hsa-miR-26b-5p; hsa-miR-103a-3p; hsa-miR-16-5p; hsa-miR-92b-3p; hsa-miR-181a-5p; hsa-miR-93-5p; hsa-miR-124-3p; hsa-miR-21-5p; hsa-miR-17-5p; hsa-miR-23a-3p; hsa-miR-331-3p; hsa-miR-197-3p; hsa-miR-30c-5p
Pathways (miRWalk)	WP496 Steroid Biosynthesis	enriched	0.0348148	4	hsa-miR-155-5p; hsa-miR-26b-5p; hsa-miR-181a-5p; hsa-miR-124-3p
Pathways (miRWalk)	hsa00510 N Glycan biosynthesis	enriched	0.0348148	34	hsa-miR-155-5p; hsa-miR-324-3p; hsa-miR-30d-5p; hsa-miR-1-3p; hsa-miR-26b-5p; hsa-miR-320a; hsa-miR-103a-3p; hsa-miR-18b-5p; hsa-let-7i-5p; hsa-miR-142-3p; hsa-miR-107; hsa-miR-16-5p; hsa-miR-20a-5p; hsa-miR-24-3p; hsa-miR-181a-5p; hsa-miR-93-5p; hsa-miR-181b-5p; hsa-miR-124-3p; hsa-miR-21-5p; hsa-miR-26a-5p; hsa-miR-1229-3p; hsa-miR-19b-3p; hsa-miR-25-3p; hsa-miR-17-5p; hsa-miR-18a-5p; hsa-miR-10a-5p; hsa-miR-15b-5p; hsa-miR-149-5p; hsa-miR-23a-3p; hsa-miR-92a-3p; hsa-miR-615-3p; hsa-miR-30c-5p; hsa-miR-328-3p; hsa-miR-1296-5p

Pathways (miRWalk)	hsa04622 RIG I like receptor signaling pathway	enriched	0.0348148	38	hsa-miR-155-5p; hsa-let-7a-5p; hsa-miR-1-3p; hsa-miR-877-5p; hsa-miR-26b-5p; hsa-miR-320a; hsa-let-7g-5p; hsa-miR-103a-3p; hsa-miR-106b-5p; hsa-miR-146a-5p; hsa-miR-16-5p; hsa-miR-20a-5p; hsa-miR-24-3p; hsa-miR-92b-3p; hsa-miR-29b-3p; hsa-miR-181a-5p; hsa-miR-93-5p; hsa-miR-181b-5p; hsa-miR-124-3p; hsa-miR-21-5p; hsa-miR-223-3p; hsa-miR-26a-5p; hsa-miR-15a-5p; hsa-miR-25-3p; hsa-miR-17-5p; hsa-miR-10a-5p; hsa-miR-15b-5p; hsa-miR-92a-3p; hsa-miR-505-3p; hsa-miR-629-3p; hsa-miR-1260b; hsa-miR-30b-5p; hsa-miR-130b-5p; hsa-miR-204-5p; hsa-miR-331-3p; hsa-miR-197-3p; hsa-miR-296-5p; hsa-miR-328-3p
Pathways (miRWalk)	hsa04730 Long term depression	enriched	0.0348148	37	hsa-let-7c-5p; hsa-miR-155-5p; hsa-let-7a-5p; hsa-miR-324-3p; hsa-miR-30d-5p; hsa-miR-1-3p; hsa-miR-26b-5p; hsa-miR-320a; hsa-let-7g-5p; hsa-miR-142-3p; hsa-miR-16-5p; hsa-miR-20a-5p; hsa-miR-92b-3p; hsa-miR-181a-5p; hsa-miR-483-5p; hsa-miR-181d-5p; hsa-miR-93-5p; hsa-miR-181b-5p; hsa-miR-124-3p; hsa-miR-21-5p; hsa-miR-223-3p; hsa-miR-15a-5p; hsa-miR-449c-5p; hsa-miR-766-3p; hsa-miR-17-5p; hsa-miR-10a-5p; hsa-miR-15b-5p; hsa-miR-148b-3p; hsa-miR-149-5p; hsa-miR-92a-3p; hsa-miR-615-3p; hsa-miR-505-3p; hsa-miR-30b-5p; hsa-miR-1226-3p; hsa-miR-331-3p; hsa-miR-197-3p; hsa-miR-30c-5p
Pathways (miRWalk)	hsa05218 Melanoma	enriched	0.0348148	64	hsa-miR-29c-3p; hsa-let-7c-5p; hsa-miR-155-5p; hsa-let-7a-5p; hsa-let-7f-5p; hsa-miR-30d-5p; hsa-miR-1-3p; hsa-miR-26b-5p; hsa-miR-320a; hsa-let-7g-5p; hsa-miR-103a-3p; hsa-miR-106b-5p; hsa-miR-18b-5p; hsa-miR-451a; hsa-miR-184; hsa-miR-146a-5p; hsa-miR-107; hsa-miR-16-5p; hsa-miR-20a-5p; hsa-miR-24-3p; hsa-miR-92b-3p; hsa-miR-29b-3p; hsa-miR-365a-3p; hsa-miR-181a-5p; hsa-miR-483-5p; hsa-miR-181d-5p; hsa-miR-93-5p; hsa-miR-181b-5p; hsa-miR-124-3p; hsa-miR-21-5p; hsa-miR-223-3p; hsa-miR-29a-3p; hsa-miR-26a-5p; hsa-miR-19a-3p; hsa-miR-1229-3p; hsa-miR-15a-5p; hsa-miR-520a-3p; hsa-miR-20b-5p; hsa-miR-19b-3p; hsa-miR-494-3p; hsa-miR-25-3p; hsa-miR-766-3p; hsa-miR-17-5p; hsa-miR-18a-5p; hsa-miR-572; hsa-miR-10a-5p; hsa-miR-15b-5p; hsa-miR-148b-3p; hsa-miR-149-5p; hsa-miR-23a-3p; hsa-miR-504-5p; hsa-miR-92a-3p; hsa-miR-299-5p; hsa-miR-615-3p; hsa-miR-1260b; hsa-miR-1226-3p; hsa-miR-331-3p; hsa-miR-346; hsa-miR-34b-3p; hsa-miR-30c-5p; hsa-miR-605-5p; hsa-miR-296-5p; hsa-miR-328-3p; hsa-miR-1296-5p
Pathways (miRWalk)	hsa05220 Chronic myeloid leukemia	enriched	0.0348148	73	hsa-miR-29c-3p; hsa-let-7c-5p; hsa-miR-155-5p; hsa-miR-193b-5p; hsa-let-7a-5p; hsa-miR-324-3p; hsa-let-7f-5p; hsa-miR-30d-5p; hsa-miR-1-3p; hsa-miR-26b-5p; hsa-miR-320a; hsa-let-7g-5p; hsa-miR-103a-3p; hsa-miR-106b-5p; hsa-miR-18b-5p; hsa-miR-451a; hsa-miR-184; hsa-miR-146a-5p; hsa-miR-142-3p; hsa-miR-107; hsa-miR-16-5p; hsa-miR-20a-5p; hsa-miR-24-3p; hsa-miR-92b-3p; hsa-miR-29b-3p; hsa-miR-671-5p; hsa-miR-365a-3p; hsa-miR-181a-5p; hsa-miR-483-5p; hsa-miR-181d-5p; hsa-miR-93-5p; hsa-miR-181b-5p; hsa-miR-124-3p; hsa-miR-21-5p; hsa-miR-223-3p; hsa-miR-29a-3p; hsa-miR-26a-5p; hsa-miR-19a-3p; hsa-miR-1229-3p; hsa-miR-15a-5p; hsa-miR-449c-5p; hsa-miR-520a-3p; hsa-miR-20b-5p; hsa-miR-940; hsa-miR-19b-3p; hsa-miR-25-3p; hsa-miR-766-3p; hsa-miR-17-5p; hsa-miR-18a-5p; hsa-miR-572; hsa-miR-10a-5p; hsa-miR-129-1-3p; hsa-miR-15b-5p; hsa-miR-148b-3p; hsa-miR-149-5p; hsa-miR-23a-3p; hsa-miR-504-5p; hsa-miR-92a-3p; hsa-miR-299-5p; hsa-miR-615-3p; hsa-miR-483-3p; hsa-miR-1260b; hsa-miR-30b-5p; hsa-miR-130b-5p; hsa-miR-204-5p; hsa-miR-331-3p; hsa-miR-197-3p; hsa-miR-346; hsa-miR-34b-3p; hsa-miR-30c-5p; hsa-miR-605-5p; hsa-miR-296-5p; hsa-miR-1296-5p

Pathways (miRWalk)	hsa05222 Small cell lung cancer	enriched	0.0348148	66	hsa-miR-29c-3p; hsa-let-7c-5p; hsa-miR-155-5p; hsa-let-7a-5p; hsa-let-7f-5p; hsa-miR-30d-5p; hsa-miR-1-3p; hsa-miR-26b-5p; hsa-miR-320a; hsa-let-7g-5p; hsa-miR-103a-3p; hsa-miR-199b-5p; hsa-miR-106b-5p; hsa-miR-760; hsa-miR-451a; hsa-miR-184; hsa-miR-146a-5p; hsa-miR-142-3p; hsa-miR-107; hsa-miR-16-5p; hsa-miR-20a-5p; hsa-miR-24-3p; hsa-miR-29b-3p; hsa-miR-365a-3p; hsa-miR-181a-5p; hsa-miR-181d-5p; hsa-miR-93-5p; hsa-miR-181b-5p; hsa-miR-124-3p; hsa-miR-4284; hsa-miR-21-5p; hsa-miR-223-3p; hsa-miR-29a-3p; hsa-miR-26a-5p; hsa-miR-19a-3p; hsa-miR-15a-5p; hsa-miR-449c-5p; hsa-miR-148a-3p; hsa-miR-320c; hsa-miR-19b-3p; hsa-miR-494-3p; hsa-miR-25-3p; hsa-miR-766-3p; hsa-miR-17-5p; hsa-miR-18a-5p; hsa-miR-630; hsa-miR-10a-5p; hsa-miR-15b-5p; hsa-miR-148b-3p; hsa-miR-149-5p; hsa-miR-23a-3p; hsa-miR-504-5p; hsa-miR-92a-3p; hsa-miR-615-3p; hsa-miR-1260b; hsa-miR-30b-5p; hsa-miR-204-5p; hsa-miR-331-3p; hsa-miR-197-3p; hsa-miR-346; hsa-miR-34b-3p; hsa-miR-30c-5p; hsa-miR-605-5p; hsa-miR-296-5p; hsa-miR-1296-5p; hsa-miR-885-5p
Gene Ontology (miRWalk)	GO0035518 histone h2a monoubiquitination	enriched	0.035685	15	hsa-let-7c-5p; hsa-miR-155-5p; hsa-let-7a-5p; hsa-miR-324-3p; hsa-let-7f-5p; hsa-miR-1-3p; hsa-miR-26b-5p; hsa-miR-320a; hsa-miR-24-3p; hsa-miR-92b-3p; hsa-miR-93-5p; hsa-miR-92a-3p; hsa-miR-615-3p; hsa-miR-346; hsa-miR-328-3p
Pathways (miRWalk)	WP129 Matrix Metalloproteinases	enriched	0.0357071	17	hsa-miR-29c-3p; hsa-miR-1-3p; hsa-miR-26b-5p; hsa-miR-103a-3p; hsa-miR-18b-5p; hsa-miR-451a; hsa-miR-16-5p; hsa-miR-29b-3p; hsa-miR-181b-5p; hsa-miR-124-3p; hsa-miR-21-5p; hsa-miR-181c-3p; hsa-miR-17-5p; hsa-miR-149-5p; hsa-miR-92a-3p; hsa-miR-197-3p; hsa-miR-328-3p
Pathways (miRWalk)	WP702 metapathway biotransformation	enriched	0.0357071	33	hsa-let-7c-5p; hsa-miR-155-5p; hsa-let-7a-5p; hsa-let-7f-5p; hsa-miR-1-3p; hsa-miR-26b-5p; hsa-miR-320a; hsa-miR-103a-3p; hsa-miR-142-3p; hsa-miR-107; hsa-miR-16-5p; hsa-miR-20a-5p; hsa-miR-24-3p; hsa-miR-92b-3p; hsa-miR-422a; hsa-miR-181a-5p; hsa-miR-93-5p; hsa-miR-124-3p; hsa-miR-21-5p; hsa-miR-1229-3p; hsa-miR-19b-3p; hsa-miR-25-3p; hsa-miR-17-5p; hsa-miR-10a-5p; hsa-miR-15b-5p; hsa-miR-148b-3p; hsa-miR-149-5p; hsa-miR-92a-3p; hsa-miR-615-3p; hsa-miR-1226-3p; hsa-miR-331-3p; hsa-miR-197-3p; hsa-miR-631
Pathways (miRWalk)	hsa05014 Amyotrophic lateral sclerosis ALS	enriched	0.0357071	46	hsa-miR-29c-3p; hsa-let-7c-5p; hsa-miR-155-5p; hsa-let-7a-5p; hsa-miR-324-3p; hsa-miR-30d-5p; hsa-miR-1-3p; hsa-miR-26b-5p; hsa-let-7g-5p; hsa-miR-103a-3p; hsa-miR-106b-5p; hsa-miR-451a; hsa-miR-142-3p; hsa-miR-16-5p; hsa-miR-20a-5p; hsa-miR-24-3p; hsa-miR-92b-3p; hsa-miR-29b-3p; hsa-miR-365a-3p; hsa-miR-181a-5p; hsa-miR-181d-5p; hsa-miR-181b-5p; hsa-miR-124-3p; hsa-miR-21-5p; hsa-miR-29a-3p; hsa-miR-1229-3p; hsa-miR-15a-5p; hsa-miR-148a-3p; hsa-miR-320c; hsa-miR-19b-3p; hsa-miR-25-3p; hsa-miR-17-5p; hsa-miR-18a-5p; hsa-miR-630; hsa-miR-15b-5p; hsa-miR-148b-3p; hsa-miR-149-5p; hsa-miR-504-5p; hsa-miR-92a-3p; hsa-miR-615-3p; hsa-miR-30b-5p; hsa-miR-204-5p; hsa-miR-197-3p; hsa-miR-30c-5p; hsa-miR-605-5p; hsa-miR-296-5p
Pathways (miRWalk)	hsa05416 Viral myocarditis	enriched	0.0357071	51	hsa-let-7c-5p; hsa-miR-155-5p; hsa-let-7a-5p; hsa-miR-324-3p; hsa-let-7f-5p; hsa-miR-30d-5p; hsa-miR-1-3p; hsa-miR-877-5p; hsa-miR-26b-5p; hsa-miR-320a; hsa-miR-103a-3p; hsa-miR-1227-3p; hsa-miR-106b-5p; hsa-miR-146a-5p; hsa-miR-1295a; hsa-miR-142-3p; hsa-miR-16-5p; hsa-miR-20a-5p; hsa-miR-24-3p; hsa-miR-92b-3p; hsa-miR-365a-3p; hsa-miR-93-5p; hsa-miR-124-3p; hsa-miR-21-5p; hsa-miR-29a-3p; hsa-miR-26a-5p; hsa-miR-99b-3p; hsa-miR-19a-3p; hsa-miR-1229-3p; hsa-miR-15a-5p; hsa-miR-148a-3p; hsa-miR-320c; hsa-miR-486-5p; hsa-miR-25-3p; hsa-miR-17-5p; hsa-miR-10a-5p; hsa-miR-15b-5p; hsa-miR-148b-3p; hsa-miR-149-5p; hsa-miR-23a-3p; hsa-miR-92a-3p; hsa-miR-615-3p; hsa-miR-505-3p; hsa-miR-1260b; hsa-miR-1226-3p; hsa-miR-331-3p;

					hsa-miR-197-3p; hsa-miR-34b-3p; hsa-miR-30c-5p; hsa-miR-296-5p; hsa-miR-1296-5p
Pathways (miRWalk)	WP524 G13 Signaling Pathway	enriched	0.0357889	38	hsa-miR-29c-3p; hsa-miR-155-5p; hsa-let-7a-5p; hsa-miR-584-5p; hsa-miR-324-3p; hsa-miR-1-3p; hsa-miR-877-5p; hsa-miR-26b-5p; hsa-miR-320a; hsa-miR-103a-3p; hsa-miR-106b-5p; hsa-miR-146a-5p; hsa-miR-142-3p; hsa-miR-425-3p; hsa-miR-16-5p; hsa-miR-92b-3p; hsa-miR-29b-3p; hsa-miR-93-5p; hsa-miR-124-3p; hsa-miR-29a-3p; hsa-miR-26a-5p; hsa-miR-99b-3p; hsa-miR-148a-3p; hsa-miR-320c; hsa-miR-766-3p; hsa-miR-17-5p; hsa-miR-18a-5p; hsa-miR-15b-5p; hsa-miR-148b-3p; hsa-miR-92a-3p; hsa-miR-615-3p; hsa-miR-505-3p; hsa-miR-30b-5p; hsa-miR-331-3p; hsa-miR-346; hsa-miR-30c-5p; hsa-miR-328-3p; hsa-miR-1296-5p
Pathways (miRWalk)	WP313 Signaling of Hepatocyte Growth Factor Receptor	enriched	0.0358234	46	hsa-miR-29c-3p; hsa-let-7c-5p; hsa-miR-155-5p; hsa-miR-193b-5p; hsa-let-7a-5p; hsa-miR-1-3p; hsa-miR-877-5p; hsa-miR-26b-5p; hsa-miR-320a; hsa-miR-103a-3p; hsa-miR-106b-5p; hsa-miR-107; hsa-miR-16-5p; hsa-miR-20a-5p; hsa-miR-92b-3p; hsa-miR-29b-3p; hsa-miR-181a-5p; hsa-miR-483-5p; hsa-miR-181d-5p; hsa-miR-93-5p; hsa-miR-181b-5p; hsa-miR-124-3p; hsa-miR-21-5p; hsa-miR-29a-3p; hsa-miR-26a-5p; hsa-miR-19a-3p; hsa-miR-15a-5p; hsa-miR-20b-5p; hsa-miR-19b-3p; hsa-miR-494-3p; hsa-miR-25-3p; hsa-miR-766-3p; hsa-miR-17-5p; hsa-miR-18a-5p; hsa-miR-10a-5p; hsa-miR-129-1-3p; hsa-miR-15b-5p; hsa-miR-148b-3p; hsa-miR-149-5p; hsa-miR-23a-3p; hsa-miR-92a-3p; hsa-miR-615-3p; hsa-miR-1260b; hsa-miR-130b-5p; hsa-miR-337-3p; hsa-miR-30c-5p
Pathways (miRWalk)	hsa04920 Adipocytokine signaling pathway	enriched	0.0358838	44	hsa-miR-29c-3p; hsa-miR-155-5p; hsa-let-7a-5p; hsa-miR-324-3p; hsa-miR-1-3p; hsa-miR-877-5p; hsa-miR-26b-5p; hsa-miR-320a; hsa-miR-103a-3p; hsa-miR-106b-5p; hsa-miR-451a; hsa-miR-184; hsa-miR-146a-5p; hsa-miR-425-3p; hsa-miR-16-5p; hsa-miR-20a-5p; hsa-miR-24-3p; hsa-miR-29b-3p; hsa-miR-181a-5p; hsa-miR-93-5p; hsa-miR-181b-5p; hsa-miR-124-3p; hsa-miR-21-5p; hsa-miR-223-3p; hsa-miR-1229-3p; hsa-miR-15a-5p; hsa-miR-20b-5p; hsa-miR-148a-3p; hsa-miR-19b-3p; hsa-miR-17-5p; hsa-miR-18a-5p; hsa-miR-10a-5p; hsa-miR-148b-3p; hsa-miR-149-5p; hsa-miR-23a-3p; hsa-miR-92a-3p; hsa-miR-615-3p; hsa-miR-130b-5p; hsa-miR-337-3p; hsa-miR-331-3p; hsa-miR-197-3p; hsa-miR-346; hsa-miR-30c-5p; hsa-miR-1296-5p
Diseases (miRWalk)	Leukemia Myeloid	enriched	0.036025	13	hsa-miR-155-5p; hsa-miR-320a; hsa-miR-107; hsa-miR-24-3p; hsa-miR-29b-3p; hsa-miR-181b-5p; hsa-miR-124-3p; hsa-miR-21-5p; hsa-miR-223-3p; hsa-miR-29a-3p; hsa-miR-15a-5p; hsa-miR-10a-5p; hsa-miR-204-5p
Diseases (miRWalk)	Neoplasm Metastasis	enriched	0.036025	32	hsa-miR-29c-3p; hsa-miR-155-5p; hsa-let-7a-5p; hsa-miR-30d-5p; hsa-miR-26b-5p; hsa-miR-320a; hsa-miR-103a-3p; hsa-miR-199b-5p; hsa-miR-451a; hsa-miR-146a-5p; hsa-miR-107; hsa-miR-16-5p; hsa-miR-20a-5p; hsa-miR-29b-3p; hsa-miR-154-5p; hsa-miR-124-3p; hsa-miR-21-5p; hsa-miR-223-3p; hsa-miR-29a-3p; hsa-miR-26a-5p; hsa-miR-148a-3p; hsa-miR-494-3p; hsa-miR-486-5p; hsa-miR-17-5p; hsa-miR-10a-5p; hsa-miR-23a-3p; hsa-miR-516a-3p; hsa-miR-299-5p; hsa-miR-204-5p; hsa-miR-197-3p; hsa-miR-34b-3p; hsa-miR-211-5p

Gene Ontology (miRWalk)	GO0005096 gtpase activator activity	enriched	0.0362263	37	hsa-let-7c-5p; hsa-miR-155-5p; hsa-let-7a-5p; hsa-miR-324-3p; hsa-let-7f-5p; hsa-miR-30d-5p; hsa-miR-1-3p; hsa-miR-877-5p; hsa-miR-26b-5p; hsa-miR-320a; hsa-miR-103a-3p; hsa-miR-146a-5p; hsa-miR-142-3p; hsa-miR-16-5p; hsa-miR-20a-5p; hsa-miR-24-3p; hsa-miR-92b-3p; hsa-miR-181a-5p; hsa-miR-93-5p; hsa-miR-181b-5p; hsa-miR-124-3p; hsa-miR-21-5p; hsa-miR-26a-5p; hsa-miR-320c; hsa-miR-19b-3p; hsa-miR-25-3p; hsa-miR-17-5p; hsa-miR-18a-5p; hsa-miR-10a-5p; hsa-miR-149-5p; hsa-miR-504-5p; hsa-miR-92a-3p; hsa-miR-615-3p; hsa-miR-331-3p; hsa-miR-1914-5p; hsa-miR-197-3p; hsa-miR-30c-5p
Gene Ontology (miRWalk)	GO0009187 cyclic nucleotide metabolic process	enriched	0.0362263	2	hsa-let-7a-5p; hsa-let-7f-5p
Gene Ontology (miRWalk)	GO0010042 response to manganese ion	enriched	0.0362263	11	hsa-let-7c-5p; hsa-let-7a-5p; hsa-miR-1-3p; hsa-miR-26b-5p; hsa-miR-320a; hsa-miR-16-5p; hsa-miR-24-3p; hsa-miR-181a-5p; hsa-miR-124-3p; hsa-miR-17-5p; hsa-miR-23a-3p
Gene Ontology (miRWalk)	GO0010389 regulation of g2 m transition of mitotic cell cycle	enriched	0.0362263	11	hsa-miR-155-5p; hsa-miR-1-3p; hsa-miR-877-5p; hsa-miR-26b-5p; hsa-miR-320a; hsa-let-7g-5p; hsa-miR-16-5p; hsa-miR-24-3p; hsa-miR-124-3p; hsa-miR-615-3p; hsa-miR-1226-3p
Gene Ontology (miRWalk)	GO0017156 calcium ion dependent exocytosis	enriched	0.0362263	11	hsa-miR-155-5p; hsa-miR-324-3p; hsa-miR-1-3p; hsa-miR-26b-5p; hsa-miR-320a; hsa-miR-103a-3p; hsa-miR-16-5p; hsa-miR-93-5p; hsa-miR-124-3p; hsa-miR-19b-3p; hsa-miR-25-3p
Gene Ontology (miRWalk)	GO0045596 negative regulation of cell differentiation	enriched	0.0362263	17	hsa-miR-29c-3p; hsa-miR-155-5p; hsa-let-7a-5p; hsa-miR-26b-5p; hsa-miR-103a-3p; hsa-miR-107; hsa-miR-16-5p; hsa-miR-29b-3p; hsa-miR-124-3p; hsa-miR-21-5p; hsa-miR-29a-3p; hsa-miR-26a-5p; hsa-miR-19a-3p; hsa-miR-615-3p; hsa-miR-30b-5p; hsa-miR-34b-3p; hsa-miR-30c-5p
Gene Ontology (miRWalk)	GO0048645 organ formation	enriched	0.0362263	11	hsa-miR-1-3p; hsa-miR-26b-5p; hsa-miR-103a-3p; hsa-miR-16-5p; hsa-miR-20a-5p; hsa-miR-92b-3p; hsa-miR-181a-5p; hsa-miR-181b-5p; hsa-miR-124-3p; hsa-miR-10a-5p; hsa-miR-1296-5p
Gene Ontology (miRWalk)	GO0051018 protein kinase a binding	enriched	0.0362263	11	hsa-let-7a-5p; hsa-miR-1-3p; hsa-miR-26b-5p; hsa-let-7g-5p; hsa-miR-106b-5p; hsa-miR-24-3p; hsa-miR-181a-5p; hsa-miR-93-5p; hsa-miR-124-3p; hsa-miR-148b-3p; hsa-miR-615-3p
Gene Ontology (miRWalk)	GO0070403 nadposi binding	enriched	0.0362263	11	hsa-let-7c-5p; hsa-miR-1-3p; hsa-miR-877-5p; hsa-miR-26b-5p; hsa-miR-103a-3p; hsa-miR-16-5p; hsa-miR-181a-5p; hsa-miR-181b-5p; hsa-miR-124-3p; hsa-miR-148b-3p; hsa-miR-331-3p
Pathways (miRWalk)	WP2064 Neural Crest Differentiation	enriched	0.036236	45	hsa-let-7c-5p; hsa-miR-155-5p; hsa-let-7a-5p; hsa-miR-324-3p; hsa-let-7f-5p; hsa-miR-30d-5p; hsa-miR-1-3p; hsa-miR-26b-5p; hsa-miR-320a; hsa-let-7g-5p; hsa-miR-103a-3p; hsa-miR-199b-5p; hsa-miR-451a; hsa-miR-146a-5p; hsa-miR-107; hsa-miR-16-5p; hsa-miR-20a-5p; hsa-miR-24-3p; hsa-miR-181a-5p; hsa-miR-93-5p; hsa-miR-181b-5p; hsa-miR-124-3p; hsa-miR-21-5p; hsa-miR-223-3p; hsa-miR-26a-5p; hsa-miR-15a-5p; hsa-miR-449c-5p; hsa-miR-320c; hsa-miR-19b-3p; hsa-miR-25-3p; hsa-miR-17-5p; hsa-miR-18a-5p; hsa-miR-630; hsa-miR-10a-5p; hsa-miR-148b-3p; hsa-miR-23a-3p; hsa-miR-92a-3p; hsa-miR-615-3p; hsa-miR-30b-5p; hsa-miR-130b-5p; hsa-miR-204-5p; hsa-miR-1226-3p; hsa-miR-331-3p; hsa-miR-34b-3p; hsa-miR-30c-5p

Gene Ontology (miRWalk)	GO0007264 small gtpase mediated signal transduction	enriched	0.036712	64	hsa-miR-29c-3p; hsa-miR-22-5p; hsa-let-7c-5p; hsa-miR-155-5p; hsa-let-7a-5p; hsa-miR-324-3p; hsa-let-7f-5p; hsa-miR-30d-5p; hsa-miR-1-3p; hsa-miR-877-5p; hsa-miR-26b-5p; hsa-miR-320a; hsa-let-7g-5p; hsa-miR-103a-3p; hsa-miR-1250-5p; hsa-miR-106b-5p; hsa-miR-760; hsa-miR-451a; hsa-miR-142-3p; hsa-miR-107; hsa-miR-16-5p; hsa-miR-20a-5p; hsa-miR-24-3p; hsa-miR-92b-3p; hsa-miR-29b-3p; hsa-miR-181a-5p; hsa-miR-483-5p; hsa-miR-181d-5p; hsa-miR-93-5p; hsa-miR-181b-5p; hsa-miR-124-3p; hsa-miR-21-5p; hsa-miR-223-3p; hsa-miR-29a-3p; hsa-miR-26a-5p; hsa-miR-19a-3p; hsa-miR-15a-5p; hsa-miR-148a-3p; hsa-miR-320c; hsa-miR-19b-3p; hsa-miR-25-3p; hsa-miR-766-3p; hsa-miR-17-5p; hsa-miR-18a-5p; hsa-miR-10a-5p; hsa-miR-15b-5p; hsa-miR-148b-3p; hsa-miR-149-5p; hsa-miR-663a; hsa-miR-92a-3p; hsa-miR-615-3p; hsa-miR-1260b; hsa-miR-30b-5p; hsa-miR-1910-5p; hsa-miR-204-5p; hsa-miR-337-3p; hsa-miR-1226-3p; hsa-miR-331-3p; hsa-miR-1914-5p; hsa-miR-197-3p; hsa-miR-211-5p; hsa-miR-30c-5p; hsa-miR-4326; hsa-miR-328-3p
Gene Ontology (miRWalk)	GO0046330 positive regulation of jnk cascade	enriched	0.036712	35	hsa-miR-29c-3p; hsa-miR-155-5p; hsa-let-7a-5p; hsa-miR-1-3p; hsa-miR-26b-5p; hsa-miR-320a; hsa-miR-103a-3p; hsa-miR-106b-5p; hsa-let-7i-5p; hsa-miR-760; hsa-miR-146a-5p; hsa-miR-142-3p; hsa-miR-16-5p; hsa-miR-29b-3p; hsa-miR-181a-5p; hsa-miR-181d-5p; hsa-miR-181b-5p; hsa-miR-124-3p; hsa-miR-4284; hsa-miR-21-5p; hsa-miR-29a-3p; hsa-miR-26a-5p; hsa-miR-940; hsa-miR-320c; hsa-miR-18a-5p; hsa-miR-10a-5p; hsa-miR-148b-3p; hsa-miR-149-5p; hsa-miR-92a-3p; hsa-miR-615-3p; hsa-miR-30b-5p; hsa-miR-204-5p; hsa-miR-331-3p; hsa-miR-30c-5p; hsa-miR-605-5p
Diseases (miRWalk)	Carcinoma Non-Small-Cell Lung	enriched	0.0370393	7	hsa-let-7g-5p; hsa-miR-451a; hsa-miR-107; hsa-miR-16-5p; hsa-miR-21-5p; hsa-miR-15a-5p; hsa-miR-449c-5p
Diseases (miRWalk)	Cardiovascular Diseases	enriched	0.0370393	5	hsa-miR-29c-3p; hsa-miR-155-5p; hsa-miR-1-3p; hsa-miR-29b-3p; hsa-miR-29a-3p
Diseases (miRWalk)	Neurodegenerative Diseases	enriched	0.0370393	5	hsa-miR-146a-5p; hsa-miR-107; hsa-miR-29b-3p; hsa-miR-21-5p; hsa-miR-29a-3p
Gene Ontology (miRWalk)	GO0005528 fk506 binding	enriched	0.0371188	21	hsa-let-7c-5p; hsa-miR-155-5p; hsa-let-7a-5p; hsa-miR-324-3p; hsa-miR-26b-5p; hsa-miR-320a; hsa-miR-103a-3p; hsa-miR-760; hsa-miR-24-3p; hsa-miR-92b-3p; hsa-miR-181a-5p; hsa-miR-93-5p; hsa-miR-124-3p; hsa-miR-21-5p; hsa-miR-99b-3p; hsa-miR-149-5p; hsa-miR-23a-3p; hsa-miR-92a-3p; hsa-miR-615-3p; hsa-miR-197-3p; hsa-miR-328-3p
Gene Ontology (miRWalk)	GO0008601 protein phosphatase type 2a regulator activity	enriched	0.0371188	23	hsa-let-7c-5p; hsa-miR-155-5p; hsa-let-7a-5p; hsa-miR-1-3p; hsa-miR-877-5p; hsa-miR-26b-5p; hsa-miR-320a; hsa-miR-199b-5p; hsa-miR-451a; hsa-miR-142-3p; hsa-miR-16-5p; hsa-miR-20a-5p; hsa-miR-365a-3p; hsa-miR-93-5p; hsa-miR-124-3p; hsa-miR-26a-5p; hsa-miR-17-5p; hsa-miR-15b-5p; hsa-miR-148b-3p; hsa-miR-92a-3p; hsa-miR-615-3p; hsa-miR-1260b; hsa-miR-1914-5p
Gene Ontology (miRWalk)	GO0018208 peptidyl proline modification	enriched	0.0371188	21	hsa-let-7c-5p; hsa-miR-155-5p; hsa-let-7a-5p; hsa-miR-324-3p; hsa-miR-26b-5p; hsa-miR-320a; hsa-miR-103a-3p; hsa-miR-760; hsa-miR-24-3p; hsa-miR-92b-3p; hsa-miR-181a-5p; hsa-miR-93-5p; hsa-miR-124-3p; hsa-miR-21-5p; hsa-miR-99b-3p; hsa-miR-149-5p; hsa-miR-23a-3p; hsa-miR-92a-3p; hsa-miR-615-3p; hsa-miR-197-3p; hsa-miR-328-3p
Gene Ontology (miRWalk)	GO0033574 response to testosterone stimulus	enriched	0.0371188	23	hsa-miR-155-5p; hsa-let-7a-5p; hsa-miR-324-3p; hsa-let-7f-5p; hsa-miR-1-3p; hsa-miR-877-5p; hsa-miR-26b-5p; hsa-miR-320a; hsa-miR-103a-3p; hsa-miR-106b-5p; hsa-miR-146a-5p; hsa-miR-16-5p; hsa-miR-20a-5p; hsa-miR-124-3p; hsa-miR-21-5p; hsa-miR-26a-5p; hsa-miR-15a-5p; hsa-miR-19b-3p; hsa-miR-17-5p; hsa-miR-148b-3p; hsa-miR-149-5p; hsa-miR-92a-3p; hsa-miR-615-3p

Gene Ontology (miRWalk)	GO0051146 striated muscle cell differentiation	enriched	0.0371188	23	hsa-let-7c-5p; hsa-miR-155-5p; hsa-let-7a-5p; hsa-miR-1-3p; hsa-miR-26b-5p; hsa-let-7g-5p; hsa-miR-106b-5p; hsa-miR-451a; hsa-miR-20a-5p; hsa-miR-24-3p; hsa-miR-181a-5p; hsa-miR-181d-5p; hsa-miR-93-5p; hsa-miR-124-3p; hsa-miR-21-5p; hsa-miR-26a-5p; hsa-miR-17-5p; hsa-miR-148b-3p; hsa-miR-149-5p; hsa-miR-615-3p; hsa-miR-130b-5p; hsa-miR-204-5p; hsa-miR-331-3p
Gene Ontology (miRWalk)	GO0042339 keratan sulfate metabolic process	enriched	0.0371579	18	hsa-let-7c-5p; hsa-miR-155-5p; hsa-miR-324-3p; hsa-miR-1-3p; hsa-miR-26b-5p; hsa-miR-103a-3p; hsa-miR-760; hsa-miR-107; hsa-miR-16-5p; hsa-miR-20a-5p; hsa-miR-29b-3p; hsa-miR-124-3p; hsa-miR-21-5p; hsa-miR-18a-5p; hsa-miR-15b-5p; hsa-miR-92a-3p; hsa-miR-615-3p; hsa-miR-204-5p
Gene Ontology (miRWalk)	GO0043547 positive regulation of gtpase activity	enriched	0.0371579	30	hsa-miR-155-5p; hsa-miR-324-3p; hsa-miR-30d-5p; hsa-miR-1-3p; hsa-miR-877-5p; hsa-miR-26b-5p; hsa-miR-320a; hsa-miR-103a-3p; hsa-miR-146a-5p; hsa-miR-142-3p; hsa-miR-16-5p; hsa-miR-20a-5p; hsa-miR-24-3p; hsa-miR-92b-3p; hsa-miR-29b-3p; hsa-miR-181a-5p; hsa-miR-93-5p; hsa-miR-124-3p; hsa-miR-21-5p; hsa-miR-320c; hsa-miR-17-5p; hsa-miR-18a-5p; hsa-miR-10a-5p; hsa-miR-149-5p; hsa-miR-504-5p; hsa-miR-92a-3p; hsa-miR-615-3p; hsa-miR-1226-3p; hsa-miR-197-3p; hsa-miR-30c-5p
Gene Ontology (miRWalk)	GO0019725 cellular homeostasis	enriched	0.0374222	25	hsa-miR-29c-3p; hsa-miR-155-5p; hsa-miR-30d-5p; hsa-miR-1-3p; hsa-miR-876-3p; hsa-miR-26b-5p; hsa-miR-320a; hsa-miR-760; hsa-miR-16-5p; hsa-miR-20a-5p; hsa-miR-24-3p; hsa-miR-29b-3p; hsa-miR-181a-5p; hsa-miR-181b-5p; hsa-miR-124-3p; hsa-miR-29a-3p; hsa-miR-26a-5p; hsa-miR-15a-5p; hsa-miR-20b-5p; hsa-miR-17-5p; hsa-miR-92a-3p; hsa-miR-3679-3p; hsa-miR-615-3p; hsa-miR-744-3p; hsa-miR-30c-5p
Gene Ontology (miRWalk)	GO0022612 gland morphogenesis	enriched	0.0374222	25	hsa-miR-29c-3p; hsa-let-7a-5p; hsa-miR-1-3p; hsa-miR-26b-5p; hsa-miR-103a-3p; hsa-miR-451a; hsa-miR-107; hsa-miR-16-5p; hsa-miR-20a-5p; hsa-miR-29b-3p; hsa-miR-365a-3p; hsa-miR-181a-5p; hsa-miR-181d-5p; hsa-miR-181b-5p; hsa-miR-21-5p; hsa-miR-29a-3p; hsa-miR-26a-5p; hsa-miR-15a-5p; hsa-miR-148a-3p; hsa-miR-17-5p; hsa-miR-630; hsa-miR-15b-5p; hsa-miR-204-5p; hsa-miR-197-3p; hsa-miR-296-5p
Gene Ontology (miRWalk)	GO2000811 negative regulation of anoikis	enriched	0.0374222	25	hsa-miR-29c-3p; hsa-let-7c-5p; hsa-miR-30d-5p; hsa-miR-1-3p; hsa-miR-876-3p; hsa-miR-26b-5p; hsa-miR-320a; hsa-miR-103a-3p; hsa-miR-16-5p; hsa-miR-24-3p; hsa-miR-29b-3p; hsa-miR-181a-5p; hsa-miR-181b-5p; hsa-miR-124-3p; hsa-miR-21-5p; hsa-miR-29a-3p; hsa-miR-26a-5p; hsa-miR-15a-5p; hsa-miR-17-5p; hsa-miR-630; hsa-miR-148b-3p; hsa-miR-3679-3p; hsa-miR-615-3p; hsa-miR-204-5p; hsa-miR-34b-3p
Gene Ontology (miRWalk)	GO0000062 fatty acyl coa binding	enriched	0.0375289	15	hsa-miR-155-5p; hsa-miR-324-3p; hsa-miR-877-5p; hsa-miR-26b-5p; hsa-miR-106b-5p; hsa-miR-760; hsa-miR-16-5p; hsa-miR-24-3p; hsa-miR-765; hsa-miR-124-3p; hsa-miR-21-5p; hsa-miR-1229-3p; hsa-miR-766-3p; hsa-miR-92a-3p; hsa-miR-197-3p
Gene Ontology (miRWalk)	GO0000139 golgi membrane	enriched	0.0375289	71	hsa-miR-29c-3p; hsa-let-7c-5p; hsa-miR-155-5p; hsa-let-7a-5p; hsa-miR-584-5p; hsa-miR-324-3p; hsa-let-7f-5p; hsa-miR-30d-5p; hsa-miR-1-3p; hsa-miR-365a-5p; hsa-miR-877-5p; hsa-miR-26b-5p; hsa-miR-320a; hsa-let-7g-5p; hsa-miR-103a-3p; hsa-miR-106b-5p; hsa-miR-18b-5p; hsa-miR-760; hsa-miR-451a; hsa-miR-146a-5p; hsa-miR-142-3p; hsa-miR-107; hsa-miR-16-5p; hsa-miR-20a-5p; hsa-miR-24-3p; hsa-miR-92b-3p; hsa-miR-29b-3p; hsa-miR-671-5p; hsa-miR-365a-3p; hsa-miR-181a-5p; hsa-miR-181d-5p; hsa-miR-93-5p; hsa-miR-181b-5p; hsa-miR-124-3p; hsa-miR-21-5p; hsa-miR-29a-3p; hsa-miR-26a-5p; hsa-miR-99b-3p; hsa-miR-19a-3p; hsa-miR-1229-3p; hsa-miR-15a-5p; hsa-miR-148a-3p; hsa-miR-19b-3p; hsa-miR-25-3p; hsa-miR-766-3p; hsa-miR-17-5p; hsa-miR-18a-5p; hsa-miR-10a-5p; hsa-miR-15b-5p; hsa-miR-148b-3p; hsa-miR-149-5p; hsa-miR-92a-3p; hsa-miR-615-3p; hsa-miR-505-3p; hsa-miR-1260b;

					hsa-miR-30b-5p; hsa-miR-130b-5p; hsa-miR-4324; hsa-miR-204-5p; hsa-miR-1226-3p; hsa-miR-331-3p; hsa-miR-1914-5p; hsa-miR-454-5p; hsa-miR-197-3p; hsa-miR-346; hsa-miR-34b-3p; hsa-miR-30c-5p; hsa-miR-605-5p; hsa-miR-296-5p; hsa-miR-328-3p; hsa-miR-1296-5p
Gene Ontology (miRWalk)	GO0000188 inactivation of mapk activity	enriched	0.0375289	20	hsa-let-7c-5p; hsa-let-7f-5p; hsa-miR-26b-5p; hsa-miR-320a; hsa-miR-103a-3p; hsa-let-7i-5p; hsa-miR-760; hsa-miR-16-5p; hsa-miR-20a-5p; hsa-miR-29b-3p; hsa-miR-181a-5p; hsa-miR-93-5p; hsa-miR-124-3p; hsa-miR-21-5p; hsa-miR-17-5p; hsa-miR-10a-5p; hsa-miR-92a-3p; hsa-miR-505-3p; hsa-let-7e-3p; hsa-miR-197-3p
Gene Ontology (miRWalk)	GO0001501 skeletal system development	enriched	0.0375289	47	hsa-miR-29c-3p; hsa-let-7c-5p; hsa-miR-155-5p; hsa-let-7a-5p; hsa-miR-324-3p; hsa-miR-30d-5p; hsa-miR-1-3p; hsa-miR-877-5p; hsa-miR-26b-5p; hsa-miR-320a; hsa-let-7g-5p; hsa-miR-103a-3p; hsa-miR-760; hsa-miR-451a; hsa-miR-146a-5p; hsa-miR-142-3p; hsa-miR-16-5p; hsa-miR-20a-5p; hsa-miR-24-3p; hsa-miR-92b-3p; hsa-miR-29b-3p; hsa-miR-181a-5p; hsa-miR-93-5p; hsa-miR-181b-5p; hsa-miR-124-3p; hsa-miR-21-5p; hsa-miR-29a-3p; hsa-miR-940; hsa-miR-19b-3p; hsa-miR-25-3p; hsa-miR-17-5p; hsa-miR-18a-5p; hsa-miR-10a-5p; hsa-miR-148b-3p; hsa-miR-149-5p; hsa-miR-129-2-3p; hsa-miR-92a-3p; hsa-miR-615-3p; hsa-miR-1260b; hsa-miR-30b-5p; hsa-miR-204-5p; hsa-miR-1226-3p; hsa-miR-331-3p; hsa-miR-1236-3p; hsa-miR-197-3p; hsa-miR-30c-5p; hsa-miR-1296-5p
Gene Ontology (miRWalk)	GO0006353 dna dependent transcription termination	enriched	0.0375289	4	hsa-miR-155-5p; hsa-miR-26b-5p; hsa-miR-760; hsa-miR-16-5p
Gene Ontology (miRWalk)	GO0006418 trna aminoacylation for protein translation	enriched	0.0375289	31	hsa-let-7c-5p; hsa-miR-155-5p; hsa-let-7a-5p; hsa-miR-324-3p; hsa-let-7f-5p; hsa-miR-30d-5p; hsa-miR-1-3p; hsa-miR-26b-5p; hsa-miR-320a; hsa-miR-106b-5p; hsa-miR-760; hsa-miR-16-5p; hsa-miR-24-3p; hsa-miR-92b-3p; hsa-miR-365a-3p; hsa-miR-93-5p; hsa-miR-124-3p; hsa-miR-21-5p; hsa-miR-29a-3p; hsa-miR-26a-5p; hsa-miR-19b-3p; hsa-miR-766-3p; hsa-miR-17-5p; hsa-miR-10a-5p; hsa-miR-15b-5p; hsa-miR-149-5p; hsa-miR-92a-3p; hsa-miR-615-3p; hsa-miR-1226-3p; hsa-miR-331-3p; hsa-miR-197-3p
Gene Ontology (miRWalk)	GO0006554 lysine catabolic process	enriched	0.0375289	7	hsa-let-7a-5p; hsa-miR-324-3p; hsa-let-7f-5p; hsa-miR-103a-3p; hsa-miR-16-5p; hsa-miR-92b-3p; hsa-miR-615-3p

Gene Ontology (miRWalk)	GO0007067 mitosis	enriched	0.0375289	70	hsa-miR-29c-3p; hsa-miR-22-5p; hsa-let-7c-5p; hsa-miR-155-5p; hsa-let-7a-5p; hsa-miR-324-3p; hsa-let-7f-5p; hsa-miR-1-3p; hsa-miR-877-5p; hsa-miR-26b-5p; hsa-miR-320a; hsa-let-7g-5p; hsa-miR-103a-3p; hsa-miR-1250-5p; hsa-miR-106b-5p; hsa-miR-18b-5p; hsa-let-7i-5p; hsa-miR-760; hsa-miR-451a; hsa-miR-146a-5p; hsa-miR-142-3p; hsa-miR-107; hsa-miR-16-5p; hsa-miR-20a-5p; hsa-miR-24-3p; hsa-miR-92b-3p; hsa-miR-29b-3p; hsa-miR-671-5p; hsa-miR-365a-3p; hsa-miR-181a-5p; hsa-miR-181d-5p; hsa-miR-93-5p; hsa-miR-181b-5p; hsa-miR-124-3p; hsa-miR-21-5p; hsa-miR-29a-3p; hsa-miR-26a-5p; hsa-miR-99b-3p; hsa-miR-1229-3p; hsa-miR-188-5p; hsa-miR-15a-5p; hsa-miR-148a-3p; hsa-miR-320c; hsa-miR-19b-3p; hsa-miR-25-3p; hsa-miR-766-3p; hsa-miR-17-5p; hsa-miR-18a-5p; hsa-miR-10a-5p; hsa-miR-15b-5p; hsa-miR-148b-3p; hsa-miR-149-5p; hsa-miR-663a; hsa-miR-23a-3p; hsa-miR-92a-3p; hsa-miR-615-3p; hsa-miR-505-3p; hsa-miR-1260b; hsa-miR-30b-5p; hsa-miR-130b-5p; hsa-miR-204-5p; hsa-miR-1226-3p; hsa-miR-331-3p; hsa-miR-197-3p; hsa-miR-346; hsa-miR-30c-5p; hsa-miR-4326; hsa-miR-328-3p; hsa-miR-1296-5p; hsa-miR-885-5p
Gene Ontology (miRWalk)	GO0007417 central nervous system development	enriched	0.0375289	49	hsa-let-7c-5p; hsa-miR-155-5p; hsa-let-7a-5p; hsa-let-7f-5p; hsa-miR-30d-5p; hsa-miR-1-3p; hsa-miR-877-5p; hsa-miR-26b-5p; hsa-miR-320a; hsa-miR-103a-3p; hsa-miR-106b-5p; hsa-miR-18b-5p; hsa-miR-760; hsa-miR-146a-5p; hsa-miR-142-3p; hsa-miR-107; hsa-miR-16-5p; hsa-miR-20a-5p; hsa-miR-24-3p; hsa-miR-92b-3p; hsa-miR-29b-3p; hsa-miR-181a-5p; hsa-miR-93-5p; hsa-miR-181b-5p; hsa-miR-124-3p; hsa-miR-21-5p; hsa-miR-29a-3p; hsa-miR-26a-5p; hsa-miR-19a-3p; hsa-miR-15a-5p; hsa-miR-19b-3p; hsa-miR-494-3p; hsa-miR-25-3p; hsa-miR-17-5p; hsa-miR-18a-5p; hsa-miR-10a-5p; hsa-miR-148b-3p; hsa-miR-149-5p; hsa-miR-23a-3p; hsa-miR-504-5p; hsa-miR-92a-3p; hsa-miR-615-3p; hsa-miR-1260b; hsa-miR-30b-5p; hsa-miR-204-5p; hsa-miR-197-3p; hsa-miR-30c-5p; hsa-miR-605-5p; hsa-miR-1296-5p
Gene Ontology (miRWalk)	GO0008599 protein phosphatase type 1 regulator activity	enriched	0.0375289	4	hsa-let-7c-5p; hsa-miR-877-5p; hsa-miR-320a; hsa-miR-16-5p
Gene Ontology (miRWalk)	GO0010033 response to organic substance	enriched	0.0375289	22	hsa-miR-155-5p; hsa-miR-324-3p; hsa-miR-1-3p; hsa-miR-877-5p; hsa-miR-26b-5p; hsa-miR-320a; hsa-miR-103a-3p; hsa-miR-106b-5p; hsa-miR-16-5p; hsa-miR-20a-5p; hsa-miR-24-3p; hsa-miR-92b-3p; hsa-miR-181a-5p; hsa-miR-124-3p; hsa-miR-21-5p; hsa-miR-1229-3p; hsa-miR-17-5p; hsa-miR-10a-5p; hsa-miR-149-5p; hsa-miR-92a-3p; hsa-miR-615-3p; hsa-miR-197-3p
Gene Ontology (miRWalk)	GO0010155 regulation of proton transport	enriched	0.0375289	3	hsa-let-7f-5p; hsa-let-7g-5p; hsa-let-7i-5p
Gene Ontology (miRWalk)	GO0010880 regulation of release of sequestered calcium ion into cytosol by sarcoplasmic reticulum	enriched	0.0375289	4	hsa-miR-155-5p; hsa-miR-1-3p; hsa-miR-320a; hsa-miR-16-5p
Gene Ontology (miRWalk)	GO0016740 transferase activity	enriched	0.0375289	26	hsa-let-7c-5p; hsa-miR-155-5p; hsa-let-7a-5p; hsa-miR-30d-5p; hsa-miR-1-3p; hsa-miR-877-5p; hsa-miR-26b-5p; hsa-miR-760; hsa-miR-142-3p; hsa-miR-16-5p; hsa-miR-92b-3p; hsa-miR-29b-3p; hsa-miR-671-5p; hsa-miR-93-5p; hsa-miR-181b-5p; hsa-miR-124-3p; hsa-miR-21-5p; hsa-miR-940; hsa-miR-19b-3p; hsa-miR-148b-3p; hsa-miR-149-5p; hsa-miR-92a-3p; hsa-miR-615-3p; hsa-miR-30b-5p; hsa-miR-331-3p; hsa-miR-30c-5p

Gene Ontology (miRWalk)	GO0030199 collagen fibril organization	enriched	0.0375289	16	hsa-miR-29c-3p; hsa-let-7c-5p; hsa-miR-1-3p; hsa-miR-877-5p; hsa-miR-26b-5p; hsa-miR-320a; hsa-let-7g-5p; hsa-miR-142-3p; hsa-miR-29b-3p; hsa-miR-124-3p; hsa-miR-21-5p; hsa-miR-29a-3p; hsa-miR-940; hsa-miR-148b-3p; hsa-miR-204-5p; hsa-miR-1236-3p
Gene Ontology (miRWalk)	GO0030900 forebrain development	enriched	0.0375289	31	hsa-miR-155-5p; hsa-let-7a-5p; hsa-miR-324-3p; hsa-let-7f-5p; hsa-miR-1-3p; hsa-miR-877-5p; hsa-miR-26b-5p; hsa-miR-320a; hsa-miR-106b-5p; hsa-miR-16-5p; hsa-miR-20a-5p; hsa-miR-24-3p; hsa-miR-181a-5p; hsa-miR-93-5p; hsa-miR-181b-5p; hsa-miR-124-3p; hsa-miR-21-5p; hsa-miR-223-3p; hsa-miR-29a-3p; hsa-miR-26a-5p; hsa-miR-17-5p; hsa-miR-10a-5p; hsa-miR-149-5p; hsa-miR-92a-3p; hsa-miR-615-3p; hsa-miR-1260b; hsa-miR-1226-3p; hsa-miR-331-3p; hsa-miR-346; hsa-miR-34b-3p; hsa-miR-30c-5p
Gene Ontology (miRWalk)	GO0030992 intraflagellar transport particle b	enriched	0.0375289	4	hsa-miR-155-5p; hsa-miR-1-3p; hsa-miR-103a-3p; hsa-miR-16-5p
Gene Ontology (miRWalk)	GO0032330 regulation of chondrocyte differentiation	enriched	0.0375289	6	hsa-miR-155-5p; hsa-miR-1-3p; hsa-miR-26b-5p; hsa-miR-124-3p; hsa-miR-21-5p; hsa-miR-26a-5p
Gene Ontology (miRWalk)	GO0032590 dendrite membrane	enriched	0.0375289	4	hsa-miR-155-5p; hsa-miR-26b-5p; hsa-miR-103a-3p; hsa-miR-16-5p
Gene Ontology (miRWalk)	GO0043922 negative regulation by host of viral transcription	enriched	0.0375289	22	hsa-miR-29c-3p; hsa-let-7c-5p; hsa-miR-155-5p; hsa-let-7a-5p; hsa-let-7f-5p; hsa-miR-26b-5p; hsa-let-7g-5p; hsa-miR-760; hsa-miR-16-5p; hsa-miR-24-3p; hsa-miR-671-5p; hsa-miR-93-5p; hsa-miR-124-3p; hsa-miR-21-5p; hsa-miR-26a-5p; hsa-miR-1229-3p; hsa-miR-15a-5p; hsa-miR-15b-5p; hsa-miR-149-5p; hsa-miR-92a-3p; hsa-miR-615-3p; hsa-miR-1260b
Gene Ontology (miRWalk)	GO0048538 thymus development	enriched	0.0375289	47	hsa-miR-29c-3p; hsa-let-7c-5p; hsa-miR-155-5p; hsa-miR-193b-5p; hsa-let-7a-5p; hsa-miR-324-3p; hsa-let-7f-5p; hsa-miR-1-3p; hsa-miR-26b-5p; hsa-miR-320a; hsa-miR-103a-3p; hsa-miR-199b-5p; hsa-miR-106b-5p; hsa-miR-451a; hsa-miR-142-3p; hsa-miR-107; hsa-miR-16-5p; hsa-miR-20a-5p; hsa-miR-24-3p; hsa-miR-29b-3p; hsa-miR-365a-3p; hsa-miR-181a-5p; hsa-miR-181d-5p; hsa-miR-181b-5p; hsa-miR-124-3p; hsa-miR-21-5p; hsa-miR-29a-3p; hsa-miR-19a-3p; hsa-miR-15a-5p; hsa-miR-148a-3p; hsa-miR-19b-3p; hsa-miR-494-3p; hsa-miR-25-3p; hsa-miR-17-5p; hsa-miR-630; hsa-miR-10a-5p; hsa-miR-15b-5p; hsa-miR-23a-3p; hsa-miR-92a-3p; hsa-miR-615-3p; hsa-miR-204-5p; hsa-miR-1226-3p; hsa-miR-331-3p; hsa-miR-197-3p; hsa-miR-296-5p; hsa-miR-328-3p; hsa-miR-1296-5p
Gene Ontology (miRWalk)	GO0050852 t cell receptor signaling pathway	enriched	0.0375289	49	hsa-let-7c-5p; hsa-miR-155-5p; hsa-let-7a-5p; hsa-miR-324-3p; hsa-let-7f-5p; hsa-miR-1-3p; hsa-miR-877-5p; hsa-miR-26b-5p; hsa-miR-320a; hsa-miR-103a-3p; hsa-miR-106b-5p; hsa-miR-760; hsa-miR-1287-5p; hsa-miR-146a-5p; hsa-miR-142-3p; hsa-miR-107; hsa-miR-16-5p; hsa-miR-20a-5p; hsa-miR-24-3p; hsa-miR-29b-3p; hsa-miR-181a-5p; hsa-miR-93-5p; hsa-miR-181b-5p; hsa-miR-124-3p; hsa-miR-21-5p; hsa-miR-223-3p; hsa-miR-29a-3p; hsa-miR-26a-5p; hsa-miR-19a-3p; hsa-miR-1914-3p; hsa-miR-15a-5p; hsa-miR-19b-3p; hsa-miR-494-3p; hsa-miR-17-5p; hsa-miR-18a-5p; hsa-miR-10a-5p; hsa-miR-15b-5p; hsa-miR-148b-3p; hsa-miR-149-5p; hsa-miR-23a-3p; hsa-miR-92a-3p; hsa-miR-615-3p; hsa-miR-30b-5p; hsa-miR-331-3p; hsa-miR-197-3p; hsa-miR-346; hsa-miR-744-3p; hsa-miR-34b-3p; hsa-miR-30c-5p
Gene Ontology (miRWalk)	GO0060487 lung epithelial cell differentiation	enriched	0.0375289	4	hsa-miR-29c-3p; hsa-let-7a-5p; hsa-miR-103a-3p; hsa-miR-16-5p

Gene Ontology (miRWalk)	GO0071356 cellular response to tumor necrosis factor	enriched	0.0375289	20	hsa-let-7c-5p; hsa-miR-155-5p; hsa-let-7a-5p; hsa-miR-1-3p; hsa-miR-26b-5p; hsa-miR-320a; hsa-miR-146a-5p; hsa-miR-16-5p; hsa-miR-24-3p; hsa-miR-181a-5p; hsa-miR-93-5p; hsa-miR-181b-5p; hsa-miR-124-3p; hsa-miR-21-5p; hsa-miR-25-3p; hsa-miR-17-5p; hsa-miR-92a-3p; hsa-miR-204-5p; hsa-miR-331-3p; hsa-miR-346
Gene Ontology (miRWalk)	GO0072593 reactive oxygen species metabolic process	enriched	0.0375289	31	hsa-miR-29c-3p; hsa-miR-155-5p; hsa-let-7a-5p; hsa-miR-324-3p; hsa-miR-1-3p; hsa-miR-26b-5p; hsa-miR-103a-3p; hsa-miR-106b-5p; hsa-miR-451a; hsa-miR-16-5p; hsa-miR-20a-5p; hsa-miR-29b-3p; hsa-miR-365a-3p; hsa-miR-181a-5p; hsa-miR-181d-5p; hsa-miR-181b-5p; hsa-miR-124-3p; hsa-miR-21-5p; hsa-miR-29a-3p; hsa-miR-26a-5p; hsa-miR-15a-5p; hsa-miR-148a-3p; hsa-miR-17-5p; hsa-miR-18a-5p; hsa-miR-630; hsa-miR-15b-5p; hsa-miR-148b-3p; hsa-miR-615-3p; hsa-miR-204-5p; hsa-miR-331-3p; hsa-miR-296-5p
Gene Ontology (miRWalk)	GO0042472 inner ear morphogenesis	enriched	0.0375298	24	hsa-miR-155-5p; hsa-miR-324-3p; hsa-let-7f-5p; hsa-miR-1-3p; hsa-miR-26b-5p; hsa-miR-320a; hsa-miR-103a-3p; hsa-miR-106b-5p; hsa-miR-142-3p; hsa-miR-107; hsa-miR-16-5p; hsa-miR-20a-5p; hsa-miR-181a-5p; hsa-miR-181b-5p; hsa-miR-124-3p; hsa-miR-21-5p; hsa-miR-15a-5p; hsa-miR-148a-3p; hsa-miR-19b-3p; hsa-miR-10a-5p; hsa-miR-148b-3p; hsa-miR-92a-3p; hsa-miR-615-3p; hsa-miR-34b-3p
Gene Ontology (miRWalk)	GO0045907 positive regulation of vasoconstriction	enriched	0.0375298	23	hsa-let-7c-5p; hsa-miR-155-5p; hsa-let-7a-5p; hsa-miR-1-3p; hsa-miR-26b-5p; hsa-miR-320a; hsa-miR-103a-3p; hsa-miR-550a-5p; hsa-miR-760; hsa-miR-16-5p; hsa-miR-92b-3p; hsa-miR-181a-5p; hsa-miR-124-3p; hsa-miR-21-5p; hsa-miR-26a-5p; hsa-miR-99b-3p; hsa-miR-320c; hsa-miR-766-3p; hsa-miR-17-5p; hsa-miR-149-5p; hsa-miR-92a-3p; hsa-miR-615-3p; hsa-miR-30b-5p
Gene Ontology (miRWalk)	GO0050853 b cell receptor signaling pathway	enriched	0.0375298	35	hsa-miR-29c-3p; hsa-let-7c-5p; hsa-miR-155-5p; hsa-let-7a-5p; hsa-miR-1-3p; hsa-miR-26b-5p; hsa-miR-103a-3p; hsa-miR-451a; hsa-miR-184; hsa-miR-16-5p; hsa-miR-20a-5p; hsa-miR-29b-3p; hsa-miR-365a-3p; hsa-miR-181a-5p; hsa-miR-181d-5p; hsa-miR-93-5p; hsa-miR-181b-5p; hsa-miR-124-3p; hsa-miR-21-5p; hsa-miR-223-3p; hsa-miR-29a-3p; hsa-miR-99b-3p; hsa-miR-15a-5p; hsa-miR-148a-3p; hsa-miR-17-5p; hsa-miR-630; hsa-miR-10a-5p; hsa-miR-15b-5p; hsa-miR-149-5p; hsa-miR-92a-3p; hsa-miR-615-3p; hsa-miR-204-5p; hsa-miR-331-3p; hsa-miR-346; hsa-miR-296-5p
Pathways (miRWalk)	hsa05216 Thyroid cancer	enriched	0.0375945	44	hsa-let-7c-5p; hsa-miR-155-5p; hsa-let-7a-5p; hsa-let-7f-5p; hsa-miR-30d-5p; hsa-miR-1-3p; hsa-miR-26b-5p; hsa-miR-320a; hsa-let-7g-5p; hsa-miR-106b-5p; hsa-miR-451a; hsa-miR-16-5p; hsa-miR-20a-5p; hsa-miR-24-3p; hsa-miR-92b-3p; hsa-miR-365a-3p; hsa-miR-181a-5p; hsa-miR-483-5p; hsa-miR-181d-5p; hsa-miR-93-5p; hsa-miR-124-3p; hsa-miR-21-5p; hsa-miR-29a-3p; hsa-miR-26a-5p; hsa-miR-19a-3p; hsa-miR-15a-5p; hsa-miR-449c-5p; hsa-miR-20b-5p; hsa-miR-25-3p; hsa-miR-766-3p; hsa-miR-17-5p; hsa-miR-15b-5p; hsa-miR-148b-3p; hsa-miR-504-5p; hsa-miR-92a-3p; hsa-miR-615-3p; hsa-miR-1226-3p; hsa-miR-331-3p; hsa-miR-197-3p; hsa-miR-346; hsa-miR-34b-3p; hsa-miR-30c-5p; hsa-miR-605-5p; hsa-miR-296-5p
Pathways (miRWalk)	P00008 Axon guidance mediated by Slit Robo	enriched	0.0380151	18	hsa-miR-29c-3p; hsa-miR-155-5p; hsa-miR-1-3p; hsa-miR-26b-5p; hsa-miR-106b-5p; hsa-miR-146a-5p; hsa-miR-142-3p; hsa-miR-29b-3p; hsa-miR-124-3p; hsa-miR-21-5p; hsa-miR-223-3p; hsa-miR-29a-3p; hsa-miR-25-3p; hsa-miR-148b-3p; hsa-miR-149-5p; hsa-miR-23a-3p; hsa-miR-30c-5p; hsa-miR-1296-5p

Pathways (miRWalk)	WP2256 Integrated Pancreatic Cancer Pathway	enriched	0.0380151	83	hsa-miR-29c-3p; hsa-let-7c-5p; hsa-miR-155-5p; hsa-let-7a-5p; hsa-miR-324-3p; hsa-let-7f-5p; hsa-miR-30d-5p; hsa-miR-1-3p; hsa-miR-877-5p; hsa-miR-26b-5p; hsa-miR-320a; hsa-let-7g-5p; hsa-miR-103a-3p; hsa-miR-199b-5p; hsa-miR-106b-5p; hsa-miR-18b-5p; hsa-let-7i-5p; hsa-miR-451a; hsa-miR-146a-5p; hsa-miR-548d-3p; hsa-miR-142-3p; hsa-miR-107; hsa-miR-16-5p; hsa-miR-20a-5p; hsa-miR-24-3p; hsa-miR-92b-3p; hsa-miR-765; hsa-miR-29b-3p; hsa-miR-365a-3p; hsa-miR-181a-5p; hsa-miR-483-5p; hsa-miR-181d-5p; hsa-miR-93-5p; hsa-miR-181b-5p; hsa-miR-124-3p; hsa-miR-21-5p; hsa-miR-223-3p; hsa-miR-29a-3p; hsa-miR-26a-5p; hsa-miR-99b-3p; hsa-miR-134-5p; hsa-miR-19a-3p; hsa-miR-1229-3p; hsa-miR-15a-5p; hsa-miR-449c-5p; hsa-miR-20b-5p; hsa-miR-148a-3p; hsa-miR-3620-3p; hsa-miR-19b-3p; hsa-miR-494-3p; hsa-miR-520g-3p; hsa-miR-25-3p; hsa-miR-766-3p; hsa-miR-17-5p; hsa-miR-18a-5p; hsa-miR-630; hsa-miR-10a-5p; hsa-miR-129-1-3p; hsa-miR-15b-5p; hsa-miR-148b-3p; hsa-miR-149-5p; hsa-miR-663a; hsa-miR-23a-3p; hsa-miR-504-5p; hsa-miR-92a-3p; hsa-miR-615-3p; hsa-miR-505-3p; hsa-miR-483-3p; hsa-miR-1260b; hsa-miR-30b-5p; hsa-miR-130b-5p; hsa-miR-204-5p; hsa-miR-337-3p; hsa-miR-1226-3p; hsa-miR-331-3p; hsa-miR-197-3p; hsa-miR-34b-3p; hsa-miR-211-5p; hsa-miR-30c-5p; hsa-miR-605-5p; hsa-miR-296-5p; hsa-miR-328-3p; hsa-miR-885-5p
Gene Ontology (miRWalk)	GO0003755 peptidyl prolyl cis trans isomerase activity	enriched	0.038143	33	hsa-let-7c-5p; hsa-miR-155-5p; hsa-let-7a-5p; hsa-miR-324-3p; hsa-miR-1-3p; hsa-miR-877-5p; hsa-miR-26b-5p; hsa-miR-320a; hsa-let-7g-5p; hsa-miR-103a-3p; hsa-miR-106b-5p; hsa-miR-760; hsa-miR-16-5p; hsa-miR-24-3p; hsa-miR-92b-3p; hsa-miR-181a-5p; hsa-miR-93-5p; hsa-miR-124-3p; hsa-miR-21-5p; hsa-miR-26a-5p; hsa-miR-99b-3p; hsa-miR-766-3p; hsa-miR-18a-5p; hsa-miR-15b-5p; hsa-miR-149-5p; hsa-miR-23a-3p; hsa-miR-504-5p; hsa-miR-92a-3p; hsa-miR-615-3p; hsa-miR-1226-3p; hsa-miR-331-3p; hsa-miR-197-3p; hsa-miR-328-3p
Gene Ontology (miRWalk)	GO0031369 translation initiation factor binding	enriched	0.038143	14	hsa-let-7c-5p; hsa-miR-155-5p; hsa-let-7a-5p; hsa-miR-26b-5p; hsa-miR-760; hsa-miR-16-5p; hsa-miR-92b-3p; hsa-miR-181a-5p; hsa-miR-124-3p; hsa-miR-21-5p; hsa-miR-26a-5p; hsa-miR-92a-3p; hsa-miR-629-3p; hsa-miR-130b-5p
Gene Ontology (miRWalk)	GO0032405 mutlalpha complex binding	enriched	0.038143	14	hsa-miR-155-5p; hsa-miR-324-3p; hsa-let-7f-5p; hsa-miR-1-3p; hsa-miR-26b-5p; hsa-miR-106b-5p; hsa-miR-16-5p; hsa-miR-24-3p; hsa-miR-124-3p; hsa-miR-21-5p; hsa-miR-26a-5p; hsa-miR-15a-5p; hsa-miR-18a-5p; hsa-miR-615-3p
Gene Ontology (miRWalk)	GO0042805 actinin binding	enriched	0.038143	9	hsa-miR-155-5p; hsa-miR-26b-5p; hsa-miR-106b-5p; hsa-miR-142-3p; hsa-miR-16-5p; hsa-miR-93-5p; hsa-miR-21-5p; hsa-miR-26a-5p; hsa-miR-17-5p
Gene Ontology (miRWalk)	GO0045190 isotype switching	enriched	0.038143	14	hsa-miR-155-5p; hsa-miR-324-3p; hsa-miR-1-3p; hsa-miR-26b-5p; hsa-miR-106b-5p; hsa-miR-146a-5p; hsa-miR-16-5p; hsa-miR-92b-3p; hsa-miR-124-3p; hsa-miR-21-5p; hsa-miR-26a-5p; hsa-miR-15a-5p; hsa-miR-92a-3p; hsa-miR-615-3p
Gene Ontology (miRWalk)	GO0048387 negative regulation of retinoic acid receptor signaling pathway	enriched	0.038143	14	hsa-let-7a-5p; hsa-miR-30d-5p; hsa-miR-1-3p; hsa-miR-320a; hsa-miR-103a-3p; hsa-miR-16-5p; hsa-miR-92b-3p; hsa-miR-93-5p; hsa-miR-124-3p; hsa-miR-21-5p; hsa-miR-26a-5p; hsa-miR-25-3p; hsa-miR-149-5p; hsa-miR-331-3p
Gene Ontology (miRWalk)	GO0051571 positive regulation of histone h3 k4 methylation	enriched	0.038143	25	hsa-miR-29c-3p; hsa-let-7c-5p; hsa-miR-155-5p; hsa-miR-26b-5p; hsa-miR-146a-5p; hsa-miR-107; hsa-miR-16-5p; hsa-miR-20a-5p; hsa-miR-24-3p; hsa-miR-29b-3p; hsa-miR-671-5p; hsa-miR-181a-5p; hsa-miR-93-5p; hsa-miR-21-5p; hsa-miR-29a-3p; hsa-miR-26a-5p; hsa-miR-99b-3p; hsa-miR-15a-5p; hsa-miR-148a-3p; hsa-miR-19b-3p; hsa-miR-148b-3p; hsa-miR-149-5p; hsa-miR-92a-3p; hsa-miR-1260b; hsa-miR-30b-5p

Gene Ontology (miRWalk)	GO0048255 mrna stabilization	enriched	0.0383348	18	hsa-let-7a-5p; hsa-miR-103a-3p; hsa-miR-106b-5p; hsa-miR-760; hsa-miR-1295a; hsa-miR-16-5p; hsa-miR-20a-5p; hsa-miR-24-3p; hsa-miR-181a-5p; hsa-miR-93-5p; hsa-miR-181b-5p; hsa-miR-21-5p; hsa-miR-223-3p; hsa-miR-17-5p; hsa-miR-10a-5p; hsa-miR-148b-3p; hsa-miR-149-5p; hsa-miR-331-3p
Pathways (miRWalk)	WP395 IL 4 signaling pathway	enriched	0.0384257	47	hsa-miR-155-5p; hsa-let-7a-5p; hsa-miR-324-3p; hsa-miR-193a-5p; hsa-miR-1-3p; hsa-miR-26b-5p; hsa-miR-320a; hsa-miR-106b-5p; hsa-let-7i-5p; hsa-miR-451a; hsa-miR-146a-5p; hsa-miR-16-5p; hsa-miR-20a-5p; hsa-miR-24-3p; hsa-miR-92b-3p; hsa-miR-29b-3p; hsa-miR-181a-5p; hsa-miR-483-5p; hsa-miR-181d-5p; hsa-miR-93-5p; hsa-miR-181b-5p; hsa-miR-124-3p; hsa-miR-21-5p; hsa-miR-223-3p; hsa-miR-29a-3p; hsa-miR-19a-3p; hsa-miR-1229-3p; hsa-miR-15a-5p; hsa-miR-20b-5p; hsa-miR-148a-3p; hsa-miR-19b-3p; hsa-miR-25-3p; hsa-miR-766-3p; hsa-miR-17-5p; hsa-miR-10a-5p; hsa-miR-15b-5p; hsa-miR-148b-3p; hsa-miR-149-5p; hsa-miR-23a-3p; hsa-miR-92a-3p; hsa-miR-615-3p; hsa-miR-30b-5p; hsa-miR-130b-5p; hsa-miR-337-3p; hsa-miR-1226-3p; hsa-miR-331-3p; hsa-miR-30c-5p
Pathways (miRWalk)	WP35 G Protein Signaling Pathways	enriched	0.0386692	43	hsa-let-7c-5p; hsa-miR-155-5p; hsa-let-7a-5p; hsa-miR-324-3p; hsa-miR-30d-5p; hsa-miR-1-3p; hsa-miR-877-5p; hsa-miR-26b-5p; hsa-miR-320a; hsa-let-7g-5p; hsa-miR-103a-3p; hsa-miR-106b-5p; hsa-miR-142-3p; hsa-miR-16-5p; hsa-miR-20a-5p; hsa-miR-24-3p; hsa-miR-92b-3p; hsa-miR-365a-3p; hsa-miR-181a-5p; hsa-miR-181d-5p; hsa-miR-93-5p; hsa-miR-124-3p; hsa-miR-21-5p; hsa-miR-26a-5p; hsa-miR-148a-3p; hsa-miR-320c; hsa-miR-19b-3p; hsa-miR-25-3p; hsa-miR-17-5p; hsa-miR-18a-5p; hsa-miR-10a-5p; hsa-miR-148b-3p; hsa-miR-149-5p; hsa-miR-23a-3p; hsa-miR-92a-3p; hsa-miR-615-3p; hsa-miR-505-3p; hsa-miR-1260b; hsa-miR-30b-5p; hsa-miR-204-5p; hsa-miR-331-3p; hsa-miR-197-3p; hsa-miR-1296-5p
Gene Ontology (miRWalk)	GO0008217 regulation of blood pressure	enriched	0.0389026	31	hsa-miR-29c-3p; hsa-let-7c-5p; hsa-miR-155-5p; hsa-let-7a-5p; hsa-miR-30d-5p; hsa-miR-1-3p; hsa-miR-26b-5p; hsa-miR-320a; hsa-let-7g-5p; hsa-miR-106b-5p; hsa-miR-550a-5p; hsa-miR-760; hsa-miR-142-3p; hsa-miR-16-5p; hsa-miR-24-3p; hsa-miR-92b-3p; hsa-miR-181a-5p; hsa-miR-124-3p; hsa-miR-26a-5p; hsa-miR-99b-3p; hsa-miR-20b-5p; hsa-miR-320c; hsa-miR-766-3p; hsa-miR-17-5p; hsa-miR-18a-5p; hsa-miR-10a-5p; hsa-miR-148b-3p; hsa-miR-149-5p; hsa-miR-92a-3p; hsa-miR-615-3p; hsa-miR-197-3p
Gene Ontology (miRWalk)	GO0000413 protein peptidyl prolyl isomerization	enriched	0.0392187	27	hsa-let-7c-5p; hsa-miR-155-5p; hsa-let-7a-5p; hsa-miR-324-3p; hsa-miR-1-3p; hsa-miR-26b-5p; hsa-miR-320a; hsa-miR-103a-3p; hsa-miR-106b-5p; hsa-miR-760; hsa-miR-24-3p; hsa-miR-92b-3p; hsa-miR-181a-5p; hsa-miR-93-5p; hsa-miR-124-3p; hsa-miR-21-5p; hsa-miR-26a-5p; hsa-miR-99b-3p; hsa-miR-766-3p; hsa-miR-18a-5p; hsa-miR-149-5p; hsa-miR-23a-3p; hsa-miR-92a-3p; hsa-miR-615-3p; hsa-miR-1226-3p; hsa-miR-197-3p; hsa-miR-328-3p
Gene Ontology (miRWalk)	GO0010739 positive regulation of protein kinase a signaling cascade	enriched	0.0392187	5	hsa-miR-1-3p; hsa-miR-26b-5p; hsa-miR-320a; hsa-miR-451a; hsa-miR-181a-5p
Gene Ontology (miRWalk)	GO0030279 negative regulation of ossification	enriched	0.0392187	32	hsa-miR-29c-3p; hsa-let-7c-5p; hsa-miR-155-5p; hsa-let-7a-5p; hsa-miR-1-3p; hsa-miR-26b-5p; hsa-miR-103a-3p; hsa-miR-451a; hsa-miR-16-5p; hsa-miR-20a-5p; hsa-miR-24-3p; hsa-miR-29b-3p; hsa-miR-365a-3p; hsa-miR-181a-5p; hsa-miR-181d-5p; hsa-miR-181b-5p; hsa-miR-124-3p; hsa-miR-21-5p; hsa-miR-223-3p; hsa-miR-29a-3p; hsa-miR-15a-5p; hsa-miR-148a-3p; hsa-miR-17-5p; hsa-miR-18a-5p; hsa-miR-630; hsa-miR-15b-5p; hsa-miR-148b-3p; hsa-miR-92a-3p; hsa-miR-204-5p; hsa-miR-197-3p; hsa-miR-34b-3p; hsa-miR-296-5p

Gene Ontology (miRWalk)	GO0031295 t cell costimulation	enriched	0.0392187	32	hsa-miR-29c-3p; hsa-miR-155-5p; hsa-let-7a-5p; hsa-miR-1-3p; hsa-miR-26b-5p; hsa-miR-320a; hsa-miR-103a-3p; hsa-miR-106b-5p; hsa-miR-451a; hsa-miR-142-3p; hsa-miR-16-5p; hsa-miR-24-3p; hsa-miR-92b-3p; hsa-miR-29b-3p; hsa-miR-181a-5p; hsa-miR-93-5p; hsa-miR-181b-5p; hsa-miR-124-3p; hsa-miR-21-5p; hsa-miR-29a-3p; hsa-miR-766-3p; hsa-miR-17-5p; hsa-miR-18a-5p; hsa-miR-10a-5p; hsa-miR-148b-3p; hsa-miR-23a-3p; hsa-miR-92a-3p; hsa-miR-130b-5p; hsa-miR-331-3p; hsa-miR-346; hsa-miR-30c-5p; hsa-miR-4326
Gene Ontology (miRWalk)	GO0032418 lysosome localization	enriched	0.0392187	5	hsa-let-7c-5p; hsa-miR-26b-5p; hsa-miR-16-5p; hsa-miR-20a-5p; hsa-miR-181a-5p
Gene Ontology (miRWalk)	GO0043171 peptide catabolic process	enriched	0.0392187	5	hsa-let-7c-5p; hsa-let-7a-5p; hsa-miR-877-5p; hsa-miR-26b-5p; hsa-miR-124-3p
Gene Ontology (miRWalk)	GO0048709 oligodendrocyte differentiation	enriched	0.0392187	32	hsa-miR-29c-3p; hsa-miR-155-5p; hsa-let-7a-5p; hsa-let-7f-5p; hsa-miR-1-3p; hsa-miR-26b-5p; hsa-miR-320a; hsa-miR-103a-3p; hsa-miR-451a; hsa-miR-16-5p; hsa-miR-20a-5p; hsa-miR-24-3p; hsa-miR-29b-3p; hsa-miR-365a-3p; hsa-miR-181a-5p; hsa-miR-181d-5p; hsa-miR-93-5p; hsa-miR-181b-5p; hsa-miR-21-5p; hsa-miR-29a-3p; hsa-miR-15a-5p; hsa-miR-148a-3p; hsa-miR-17-5p; hsa-miR-630; hsa-miR-15b-5p; hsa-miR-148b-3p; hsa-miR-615-3p; hsa-miR-204-5p; hsa-miR-331-3p; hsa-miR-34b-3p; hsa-miR-30c-5p; hsa-miR-296-5p
Gene Ontology (miRWalk)	GO0071985 multivesicular body sorting pathway	enriched	0.0392187	5	hsa-miR-155-5p; hsa-let-7a-5p; hsa-miR-1-3p; hsa-miR-26b-5p; hsa-miR-124-3p
Diseases (published studies about miRNA profiles from peripheral blood)	benign prostate hyperplasia upregulated	depleted	0.0392801	17	hsa-miR-320a; hsa-miR-320d; hsa-miR-26a-2-3p; hsa-miR-1915-3p; hsa-miR-940; hsa-miR-320c; hsa-miR-766-3p; hsa-miR-663a; hsa-miR-1249-3p; hsa-miR-1538; hsa-miR-574-3p; hsa-let-7e-3p; hsa-miR-1226-3p; hsa-miR-197-3p; hsa-miR-346; hsa-miR-30c-5p; hsa-miR-296-5p
Diseases (published studies about miRNA profiles from peripheral blood)	multiple sclerosis upregulated	enriched	0.0392801	36	hsa-miR-29c-3p; hsa-miR-22-5p; hsa-let-7c-5p; hsa-let-7f-5p; hsa-miR-30d-5p; hsa-miR-877-5p; hsa-miR-26b-5p; hsa-let-7g-5p; hsa-miR-18b-5p; hsa-let-7i-5p; hsa-miR-1287-5p; hsa-miR-451a; hsa-miR-146a-5p; hsa-miR-142-3p; hsa-miR-422a; hsa-miR-939-5p; hsa-miR-365a-3p; hsa-miR-181a-5p; hsa-miR-93-5p; hsa-miR-1207-5p; hsa-miR-223-3p; hsa-miR-1914-3p; hsa-miR-130a-5p; hsa-miR-1275; hsa-miR-636; hsa-miR-572; hsa-miR-663a; hsa-miR-142-5p; hsa-miR-92a-3p; hsa-miR-1225-5p; hsa-miR-582-3p; hsa-miR-130b-5p; hsa-miR-1226-3p; hsa-miR-331-3p; hsa-miR-744-3p; hsa-miR-328-3p
Pathways (miRWalk)	hsa04670 Leukocyte transendothelial migration	enriched	0.0393311	54	hsa-miR-29c-3p; hsa-let-7c-5p; hsa-miR-155-5p; hsa-let-7a-5p; hsa-miR-584-5p; hsa-miR-324-3p; hsa-miR-30d-5p; hsa-miR-1-3p; hsa-miR-877-5p; hsa-miR-26b-5p; hsa-miR-320a; hsa-miR-1227-3p; hsa-miR-106b-5p; hsa-miR-451a; hsa-miR-146a-5p; hsa-miR-1295a; hsa-miR-142-3p; hsa-miR-16-5p; hsa-miR-24-3p; hsa-miR-92b-3p; hsa-miR-29b-3p; hsa-miR-181a-5p; hsa-miR-93-5p; hsa-miR-181b-5p; hsa-miR-124-3p; hsa-miR-21-5p; hsa-miR-29a-3p; hsa-miR-26a-5p; hsa-miR-99b-3p; hsa-miR-1229-3p; hsa-miR-1914-3p; hsa-miR-320c; hsa-miR-25-3p; hsa-miR-17-5p; hsa-miR-18a-5p; hsa-miR-10a-5p; hsa-miR-15b-5p; hsa-miR-148b-3p; hsa-miR-149-5p; hsa-miR-23a-3p; hsa-miR-92a-3p; hsa-miR-615-3p; hsa-miR-505-3p; hsa-miR-130b-5p; hsa-miR-204-5p; hsa-miR-337-3p; hsa-miR-1226-3p; hsa-miR-331-3p; hsa-miR-197-3p; hsa-miR-346; hsa-miR-744-3p; hsa-miR-211-5p; hsa-miR-30c-5p; hsa-miR-328-3p

Gene Ontology (miRWalk)	GO0001829 trophectodermal cell differentiation	enriched	0.0394148	28	hsa-miR-29c-3p; hsa-miR-155-5p; hsa-let-7a-5p; hsa-miR-324-3p; hsa-let-7f-5p; hsa-miR-1-3p; hsa-miR-877-5p; hsa-miR-26b-5p; hsa-miR-320a; hsa-miR-103a-3p; hsa-miR-106b-5p; hsa-miR-16-5p; hsa-miR-29b-3p; hsa-miR-181a-5p; hsa-miR-181b-5p; hsa-miR-124-3p; hsa-miR-21-5p; hsa-miR-223-3p; hsa-miR-25-3p; hsa-miR-10a-5p; hsa-miR-149-5p; hsa-miR-663a; hsa-miR-92a-3p; hsa-miR-1260b; hsa-miR-204-5p; hsa-miR-1226-3p; hsa-miR-331-3p; hsa-miR-30c-5p
Gene Ontology (miRWalk)	GO0006027 glycosaminoglycan catabolic process	enriched	0.0394148	13	hsa-miR-155-5p; hsa-miR-1-3p; hsa-miR-26b-5p; hsa-miR-103a-3p; hsa-miR-760; hsa-miR-142-3p; hsa-miR-107; hsa-miR-16-5p; hsa-miR-181a-5p; hsa-miR-124-3p; hsa-miR-149-5p; hsa-miR-663a; hsa-miR-615-3p
Gene Ontology (miRWalk)	GO0006195 purine nucleotide catabolic process	enriched	0.0394148	11	hsa-miR-155-5p; hsa-miR-1-3p; hsa-miR-26b-5p; hsa-miR-320a; hsa-miR-16-5p; hsa-miR-92b-3p; hsa-miR-181b-5p; hsa-miR-124-3p; hsa-miR-21-5p; hsa-miR-30b-5p; hsa-miR-1296-5p
Gene Ontology (miRWalk)	GO0006885 regulation of ph	enriched	0.0394148	11	hsa-miR-155-5p; hsa-miR-1-3p; hsa-miR-26b-5p; hsa-miR-103a-3p; hsa-miR-106b-5p; hsa-miR-16-5p; hsa-miR-93-5p; hsa-miR-124-3p; hsa-miR-21-5p; hsa-miR-148b-3p; hsa-miR-331-3p
Gene Ontology (miRWalk)	GO0008236 serine type peptidase activity	enriched	0.0394148	13	hsa-miR-155-5p; hsa-let-7f-5p; hsa-miR-1-3p; hsa-miR-877-5p; hsa-miR-26b-5p; hsa-miR-106b-5p; hsa-miR-142-3p; hsa-miR-16-5p; hsa-miR-93-5p; hsa-miR-124-3p; hsa-miR-10a-5p; hsa-miR-615-3p; hsa-miR-197-3p
Gene Ontology (miRWalk)	GO0009798 axis specification	enriched	0.0394148	10	hsa-miR-29c-3p; hsa-miR-155-5p; hsa-miR-324-3p; hsa-miR-103a-3p; hsa-miR-106b-5p; hsa-miR-16-5p; hsa-miR-181a-5p; hsa-miR-21-5p; hsa-miR-15a-5p; hsa-miR-148a-3p
Gene Ontology (miRWalk)	GO0019955 cytokine binding	enriched	0.0394148	11	hsa-miR-155-5p; hsa-miR-1-3p; hsa-miR-26b-5p; hsa-miR-16-5p; hsa-miR-24-3p; hsa-miR-181a-5p; hsa-miR-181b-5p; hsa-miR-124-3p; hsa-miR-21-5p; hsa-miR-331-3p; hsa-miR-1296-5p
Gene Ontology (miRWalk)	GO0030030 cell projection organization	enriched	0.0394148	11	hsa-miR-155-5p; hsa-miR-1-3p; hsa-miR-26b-5p; hsa-miR-320a; hsa-miR-106b-5p; hsa-miR-451a; hsa-miR-16-5p; hsa-miR-124-3p; hsa-miR-21-5p; hsa-miR-92a-3p; hsa-miR-615-3p
Gene Ontology (miRWalk)	GO0035815 positive regulation of renal sodium excretion	enriched	0.0394148	5	hsa-miR-155-5p; hsa-miR-1-3p; hsa-miR-26b-5p; hsa-miR-320a; hsa-miR-124-3p
Gene Ontology (miRWalk)	GO0048016 inositol phosphate mediated signaling	enriched	0.0394148	5	hsa-miR-155-5p; hsa-miR-1-3p; hsa-miR-26b-5p; hsa-miR-320a; hsa-miR-124-3p
Gene Ontology (miRWalk)	GO0050821 protein stabilization	enriched	0.0394148	54	hsa-miR-29c-3p; hsa-let-7c-5p; hsa-miR-155-5p; hsa-let-7a-5p; hsa-miR-324-3p; hsa-let-7f-5p; hsa-miR-1-3p; hsa-miR-877-5p; hsa-miR-26b-5p; hsa-miR-320a; hsa-let-7g-5p; hsa-miR-103a-3p; hsa-miR-106b-5p; hsa-miR-18b-5p; hsa-miR-760; hsa-miR-142-3p; hsa-miR-425-3p; hsa-miR-107; hsa-miR-16-5p; hsa-miR-20a-5p; hsa-miR-24-3p; hsa-miR-92b-3p; hsa-miR-29b-3p; hsa-miR-93-5p; hsa-miR-181b-5p; hsa-miR-124-3p; hsa-miR-21-5p; hsa-miR-223-3p; hsa-miR-29a-3p; hsa-miR-26a-5p; hsa-miR-19a-3p; hsa-miR-1229-3p; hsa-miR-15a-5p; hsa-miR-19b-3p; hsa-miR-494-3p; hsa-miR-25-3p; hsa-miR-766-3p; hsa-miR-17-5p; hsa-miR-18a-5p; hsa-miR-15b-5p; hsa-miR-148b-3p; hsa-miR-149-5p; hsa-miR-23a-3p; hsa-miR-129-2-3p; hsa-miR-92a-3p; hsa-miR-615-3p; hsa-miR-30b-5p; hsa-miR-204-5p; hsa-miR-1226-3p; hsa-miR-331-3p; hsa-miR-197-3p; hsa-miR-30c-5p; hsa-miR-328-3p; hsa-miR-1296-5p

Gene Ontology (miRWalk)	GO0006644 phospholipid metabolic process	enriched	0.0395096	52	hsa-let-7c-5p; hsa-miR-155-5p; hsa-let-7a-5p; hsa-miR-324-3p; hsa-let-7f-5p; hsa-miR-1-3p; hsa-miR-877-5p; hsa-miR-26b-5p; hsa-miR-320a; hsa-miR-103a-3p; hsa-miR-106b-5p; hsa-let-7i-5p; hsa-miR-184; hsa-miR-142-3p; hsa-miR-107; hsa-miR-16-5p; hsa-miR-20a-5p; hsa-miR-24-3p; hsa-miR-92b-3p; hsa-miR-29b-3p; hsa-miR-671-5p; hsa-miR-181a-5p; hsa-miR-93-5p; hsa-miR-181b-5p; hsa-miR-124-3p; hsa-miR-21-5p; hsa-miR-29a-3p; hsa-miR-26a-5p; hsa-miR-2116-3p; hsa-miR-19a-3p; hsa-miR-1229-3p; hsa-miR-15a-5p; hsa-miR-449c-5p; hsa-miR-19b-3p; hsa-miR-494-3p; hsa-miR-17-5p; hsa-miR-18a-5p; hsa-miR-10a-5p; hsa-miR-15b-5p; hsa-miR-148b-3p; hsa-miR-149-5p; hsa-miR-23a-3p; hsa-miR-92a-3p; hsa-miR-615-3p; hsa-miR-1260b; hsa-miR-30b-5p; hsa-miR-1226-3p; hsa-miR-331-3p; hsa-miR-346; hsa-miR-30c-5p; hsa-miR-328-3p; hsa-miR-1296-5p
Pathways (miRWalk)	WP366 TGF beta Signaling Pathway1	enriched	0.0396411	69	hsa-miR-29c-3p; hsa-let-7c-5p; hsa-miR-155-5p; hsa-let-7a-5p; hsa-miR-324-3p; hsa-let-7f-5p; hsa-miR-30d-5p; hsa-miR-1-3p; hsa-miR-498; hsa-miR-877-5p; hsa-miR-26b-5p; hsa-miR-320a; hsa-let-7g-5p; hsa-miR-103a-3p; hsa-miR-106b-5p; hsa-miR-451a; hsa-miR-146a-5p; hsa-miR-142-3p; hsa-miR-16-5p; hsa-miR-20a-5p; hsa-miR-24-3p; hsa-miR-92b-3p; hsa-miR-29b-3p; hsa-miR-671-5p; hsa-miR-365a-3p; hsa-miR-181a-5p; hsa-miR-181d-5p; hsa-miR-93-5p; hsa-miR-181b-5p; hsa-miR-124-3p; hsa-miR-595; hsa-miR-21-5p; hsa-miR-223-3p; hsa-miR-29a-3p; hsa-miR-26a-5p; hsa-miR-19a-3p; hsa-miR-188-5p; hsa-miR-15a-5p; hsa-miR-449c-5p; hsa-miR-320c; hsa-miR-19b-3p; hsa-miR-25-3p; hsa-miR-766-3p; hsa-miR-17-5p; hsa-miR-18a-5p; hsa-miR-630; hsa-miR-10a-5p; hsa-miR-15b-5p; hsa-miR-148b-3p; hsa-miR-149-5p; hsa-miR-663a; hsa-miR-23a-3p; hsa-miR-504-5p; hsa-miR-92a-3p; hsa-miR-615-3p; hsa-miR-483-3p; hsa-miR-1260b; hsa-miR-30b-5p; hsa-miR-130b-5p; hsa-miR-204-5p; hsa-miR-1226-3p; hsa-miR-331-3p; hsa-miR-197-3p; hsa-miR-34b-3p; hsa-miR-30c-5p; hsa-miR-605-5p; hsa-miR-296-5p; hsa-miR-328-3p; hsa-miR-1296-5p
Gene Ontology (miRWalk)	GO0090344 negative regulation of cell aging	enriched	0.0402657	21	hsa-miR-26b-5p; hsa-miR-320a; hsa-miR-103a-3p; hsa-miR-106b-5p; hsa-miR-451a; hsa-miR-107; hsa-miR-20a-5p; hsa-miR-29b-3p; hsa-miR-93-5p; hsa-miR-181b-5p; hsa-miR-21-5p; hsa-miR-29a-3p; hsa-miR-26a-5p; hsa-miR-134-5p; hsa-miR-19a-3p; hsa-miR-19b-3p; hsa-miR-494-3p; hsa-miR-17-5p; hsa-miR-18a-5p; hsa-miR-149-5p; hsa-miR-23a-3p
Gene Ontology (miRWalk)	GO0006342 chromatin silencing	enriched	0.0404752	26	hsa-let-7c-5p; hsa-miR-155-5p; hsa-let-7a-5p; hsa-miR-877-5p; hsa-miR-26b-5p; hsa-miR-320a; hsa-miR-103a-3p; hsa-miR-106b-5p; hsa-miR-760; hsa-miR-425-3p; hsa-miR-16-5p; hsa-miR-92b-3p; hsa-miR-181a-5p; hsa-miR-93-5p; hsa-miR-181b-5p; hsa-miR-124-3p; hsa-miR-19a-3p; hsa-miR-1229-3p; hsa-miR-766-3p; hsa-miR-15b-5p; hsa-miR-148b-3p; hsa-miR-92a-3p; hsa-miR-802; hsa-miR-615-3p; hsa-miR-331-3p; hsa-miR-30c-5p
Gene Ontology (miRWalk)	GO0043499 eukaryotic cell surface binding	enriched	0.0404752	26	hsa-miR-29c-3p; hsa-miR-155-5p; hsa-let-7a-5p; hsa-miR-324-3p; hsa-let-7f-5p; hsa-miR-1-3p; hsa-miR-26b-5p; hsa-miR-320a; hsa-miR-16-5p; hsa-miR-20a-5p; hsa-miR-24-3p; hsa-miR-29b-3p; hsa-miR-93-5p; hsa-miR-124-3p; hsa-miR-21-5p; hsa-miR-29a-3p; hsa-miR-26a-5p; hsa-miR-1229-3p; hsa-miR-17-5p; hsa-miR-148b-3p; hsa-miR-149-5p; hsa-miR-23a-3p; hsa-miR-92a-3p; hsa-miR-615-3p; hsa-miR-629-3p; hsa-miR-331-3p

Gene Ontology (miRWalk)	GO0031647 regulation of protein stability	enriched	0.040522	43	hsa-miR-29c-3p; hsa-miR-155-5p; hsa-let-7a-5p; hsa-miR-1-3p; hsa-miR-877-5p; hsa-miR-26b-5p; hsa-miR-320a; hsa-let-7g-5p; hsa-miR-103a-3p; hsa-miR-106b-5p; hsa-miR-451a; hsa-miR-107; hsa-miR-16-5p; hsa-miR-20a-5p; hsa-miR-24-3p; hsa-miR-29b-3p; hsa-miR-365a-3p; hsa-miR-181a-5p; hsa-miR-181d-5p; hsa-miR-93-5p; hsa-miR-181b-5p; hsa-miR-124-3p; hsa-miR-21-5p; hsa-miR-29a-3p; hsa-miR-26a-5p; hsa-miR-19a-3p; hsa-miR-15a-5p; hsa-miR-148a-3p; hsa-miR-19b-3p; hsa-miR-494-3p; hsa-miR-17-5p; hsa-miR-18a-5p; hsa-miR-630; hsa-miR-15b-5p; hsa-miR-23a-3p; hsa-miR-129-2-3p; hsa-miR-92a-3p; hsa-miR-615-3p; hsa-miR-30b-5p; hsa-miR-204-5p; hsa-miR-331-3p; hsa-miR-30c-5p; hsa-miR-296-5p
Gene Ontology (miRWalk)	GO0046870 cadmium ion binding	enriched	0.0408111	4	hsa-miR-155-5p; hsa-miR-26b-5p; hsa-miR-16-5p; hsa-miR-24-3p
Gene Ontology (miRWalk)	GO0001952 regulation of cell matrix adhesion	enriched	0.0409681	26	hsa-miR-29c-3p; hsa-let-7a-5p; hsa-miR-1-3p; hsa-miR-320a; hsa-miR-103a-3p; hsa-miR-106b-5p; hsa-miR-760; hsa-miR-451a; hsa-miR-16-5p; hsa-miR-20a-5p; hsa-miR-29b-3p; hsa-miR-365a-3p; hsa-miR-181a-5p; hsa-miR-181d-5p; hsa-miR-181b-5p; hsa-miR-21-5p; hsa-miR-29a-3p; hsa-miR-15a-5p; hsa-miR-148a-3p; hsa-miR-17-5p; hsa-miR-630; hsa-miR-15b-5p; hsa-miR-148b-3p; hsa-miR-92a-3p; hsa-miR-204-5p; hsa-miR-296-5p
Gene Ontology (miRWalk)	GO0002320 lymphoid progenitor cell differentiation	enriched	0.0409681	22	hsa-miR-29c-3p; hsa-miR-155-5p; hsa-let-7a-5p; hsa-miR-1-3p; hsa-miR-103a-3p; hsa-miR-451a; hsa-miR-16-5p; hsa-miR-20a-5p; hsa-miR-29b-3p; hsa-miR-365a-3p; hsa-miR-181a-5p; hsa-miR-181d-5p; hsa-miR-181b-5p; hsa-miR-21-5p; hsa-miR-29a-3p; hsa-miR-15a-5p; hsa-miR-148a-3p; hsa-miR-17-5p; hsa-miR-630; hsa-miR-15b-5p; hsa-miR-204-5p; hsa-miR-296-5p
Gene Ontology (miRWalk)	GO0003014 renal system process	enriched	0.0409681	24	hsa-miR-29c-3p; hsa-let-7a-5p; hsa-miR-1-3p; hsa-miR-103a-3p; hsa-miR-451a; hsa-miR-146a-5p; hsa-miR-142-3p; hsa-miR-16-5p; hsa-miR-20a-5p; hsa-miR-29b-3p; hsa-miR-365a-3p; hsa-miR-181a-5p; hsa-miR-181d-5p; hsa-miR-181b-5p; hsa-miR-21-5p; hsa-miR-29a-3p; hsa-miR-15a-5p; hsa-miR-148a-3p; hsa-miR-17-5p; hsa-miR-630; hsa-miR-15b-5p; hsa-miR-504-5p; hsa-miR-204-5p; hsa-miR-296-5p
Gene Ontology (miRWalk)	GO0005815 microtubule organizing center	enriched	0.0409681	43	hsa-miR-29c-3p; hsa-let-7c-5p; hsa-miR-155-5p; hsa-let-7a-5p; hsa-miR-324-3p; hsa-miR-30d-5p; hsa-miR-1-3p; hsa-miR-26b-5p; hsa-miR-320a; hsa-let-7g-5p; hsa-miR-103a-3p; hsa-miR-106b-5p; hsa-miR-760; hsa-miR-142-3p; hsa-miR-16-5p; hsa-miR-20a-5p; hsa-miR-24-3p; hsa-miR-92b-3p; hsa-miR-422a; hsa-miR-29b-3p; hsa-miR-93-5p; hsa-miR-181b-5p; hsa-miR-124-3p; hsa-miR-21-5p; hsa-miR-29a-3p; hsa-miR-15a-5p; hsa-miR-19b-3p; hsa-miR-766-3p; hsa-miR-17-5p; hsa-miR-18a-5p; hsa-miR-10a-5p; hsa-miR-15b-5p; hsa-miR-148b-3p; hsa-miR-149-5p; hsa-miR-663a; hsa-miR-23a-3p; hsa-miR-92a-3p; hsa-miR-615-3p; hsa-miR-1260b; hsa-miR-331-3p; hsa-miR-197-3p; hsa-miR-30c-5p; hsa-miR-1296-5p
Gene Ontology (miRWalk)	GO0010008 endosome membrane	enriched	0.0409681	56	hsa-let-7c-5p; hsa-miR-155-5p; hsa-let-7a-5p; hsa-miR-324-3p; hsa-let-7f-5p; hsa-miR-30d-5p; hsa-miR-1-3p; hsa-miR-877-5p; hsa-miR-26b-5p; hsa-miR-320a; hsa-miR-103a-3p; hsa-miR-1227-3p; hsa-miR-199b-5p; hsa-miR-106b-5p; hsa-let-7i-5p; hsa-miR-760; hsa-miR-1287-5p; hsa-miR-146a-5p; hsa-miR-548d-3p; hsa-miR-16-5p; hsa-miR-20a-5p; hsa-miR-24-3p; hsa-miR-92b-3p; hsa-miR-181a-5p; hsa-miR-93-5p; hsa-miR-181b-5p; hsa-miR-124-3p; hsa-miR-21-5p; hsa-miR-223-3p; hsa-miR-29a-3p; hsa-miR-26a-5p; hsa-miR-19a-3p; hsa-miR-15a-5p; hsa-miR-148a-3p; hsa-miR-940; hsa-miR-19b-3p; hsa-miR-25-3p; hsa-miR-17-5p; hsa-miR-18a-5p; hsa-miR-10a-5p; hsa-miR-15b-5p; hsa-miR-148b-3p; hsa-miR-149-5p; hsa-miR-23a-3p; hsa-miR-26b-3p; hsa-miR-92a-3p; hsa-miR-615-3p; hsa-miR-30b-5p; hsa-miR-204-5p; hsa-miR-1226-3p; hsa-miR-331-

					3p; hsa-miR-197-3p; hsa-miR-211-5p; hsa-miR-30c-5p; hsa-miR-296-5p; hsa-miR-328-3p
Gene Ontology (miRWalk)	GO0030983 mismatched dna binding	enriched	0.0409681	11	hsa-miR-29c-3p; hsa-miR-155-5p; hsa-miR-324-3p; hsa-miR-1-3p; hsa-miR-106b-5p; hsa-miR-16-5p; hsa-miR-92b-3p; hsa-miR-21-5p; hsa-miR-26a-5p; hsa-miR-15a-5p; hsa-miR-615-3p
Gene Ontology (miRWalk)	GO0031103 axon regeneration	enriched	0.0409681	26	hsa-miR-29c-3p; hsa-miR-155-5p; hsa-let-7a-5p; hsa-miR-1-3p; hsa-miR-26b-5p; hsa-miR-103a-3p; hsa-miR-451a; hsa-miR-16-5p; hsa-miR-20a-5p; hsa-miR-29b-3p; hsa-miR-365a-3p; hsa-miR-181a-5p; hsa-miR-181d-5p; hsa-miR-93-5p; hsa-miR-181b-5p; hsa-miR-21-5p; hsa-miR-29a-3p; hsa-miR-15a-5p; hsa-miR-148a-3p; hsa-miR-17-5p; hsa-miR-630; hsa-miR-15b-5p; hsa-miR-149-5p; hsa-miR-615-3p; hsa-miR-204-5p; hsa-miR-296-5p
Gene Ontology (miRWalk)	GO0032355 response to estradiol stimulus	enriched	0.0409681	48	hsa-miR-29c-3p; hsa-let-7c-5p; hsa-miR-155-5p; hsa-let-7a-5p; hsa-miR-324-3p; hsa-let-7f-5p; hsa-miR-30d-5p; hsa-miR-1-3p; hsa-miR-877-5p; hsa-miR-26b-5p; hsa-miR-320a; hsa-miR-103a-3p; hsa-miR-106b-5p; hsa-miR-18b-5p; hsa-let-7i-5p; hsa-miR-451a; hsa-miR-146a-5p; hsa-miR-107; hsa-miR-16-5p; hsa-miR-20a-5p; hsa-miR-24-3p; hsa-miR-29b-3p; hsa-miR-181a-5p; hsa-miR-93-5p; hsa-miR-124-3p; hsa-miR-21-5p; hsa-miR-26a-5p; hsa-miR-19a-3p; hsa-miR-1229-3p; hsa-miR-15a-5p; hsa-miR-20b-5p; hsa-miR-19b-3p; hsa-miR-25-3p; hsa-miR-17-5p; hsa-miR-18a-5p; hsa-miR-10a-5p; hsa-miR-15b-5p; hsa-miR-148b-3p; hsa-miR-149-5p; hsa-miR-92a-3p; hsa-miR-615-3p; hsa-miR-505-3p; hsa-miR-30b-5p; hsa-miR-204-5p; hsa-miR-337-3p; hsa-miR-1226-3p; hsa-miR-331-3p; hsa-miR-30c-5p
Gene Ontology (miRWalk)	GO0032469 endoplasmic reticulum calcium ion homeostasis	enriched	0.0409681	22	hsa-miR-29c-3p; hsa-miR-155-5p; hsa-let-7a-5p; hsa-miR-1-3p; hsa-miR-103a-3p; hsa-miR-451a; hsa-miR-16-5p; hsa-miR-20a-5p; hsa-miR-29b-3p; hsa-miR-365a-3p; hsa-miR-181a-5p; hsa-miR-181d-5p; hsa-miR-181b-5p; hsa-miR-21-5p; hsa-miR-29a-3p; hsa-miR-15a-5p; hsa-miR-148a-3p; hsa-miR-17-5p; hsa-miR-630; hsa-miR-15b-5p; hsa-miR-204-5p; hsa-miR-296-5p
Gene Ontology (miRWalk)	GO0032835 glomerulus development	enriched	0.0409681	24	hsa-miR-29c-3p; hsa-let-7a-5p; hsa-miR-1-3p; hsa-miR-26b-5p; hsa-miR-320a; hsa-miR-103a-3p; hsa-miR-451a; hsa-miR-16-5p; hsa-miR-20a-5p; hsa-miR-29b-3p; hsa-miR-365a-3p; hsa-miR-181a-5p; hsa-miR-181d-5p; hsa-miR-181b-5p; hsa-miR-21-5p; hsa-miR-29a-3p; hsa-miR-15a-5p; hsa-miR-148a-3p; hsa-miR-17-5p; hsa-miR-18a-5p; hsa-miR-630; hsa-miR-15b-5p; hsa-miR-204-5p; hsa-miR-296-5p
Gene Ontology (miRWalk)	GO0033689 negative regulation of osteoblast proliferation	enriched	0.0409681	26	hsa-miR-29c-3p; hsa-miR-155-5p; hsa-let-7a-5p; hsa-miR-1-3p; hsa-miR-877-5p; hsa-miR-103a-3p; hsa-miR-451a; hsa-miR-16-5p; hsa-miR-20a-5p; hsa-miR-29b-3p; hsa-miR-365a-3p; hsa-miR-181a-5p; hsa-miR-181d-5p; hsa-miR-181b-5p; hsa-miR-124-3p; hsa-miR-21-5p; hsa-miR-29a-3p; hsa-miR-15a-5p; hsa-miR-148a-3p; hsa-miR-17-5p; hsa-miR-18a-5p; hsa-miR-630; hsa-miR-15b-5p; hsa-miR-204-5p; hsa-miR-296-5p; hsa-miR-328-3p

Gene Ontology (miRWalk)	GO0042476 odontogenesis	enriched	0.0409681	26	hsa-miR-29c-3p; hsa-let-7c-5p; hsa-let-7a-5p; hsa-let-7f-5p; hsa-miR-1-3p; hsa-miR-26b-5p; hsa-miR-320a; hsa-let-7g-5p; hsa-miR-103a-3p; hsa-miR-760; hsa-miR-146a-5p; hsa-miR-16-5p; hsa-miR-24-3p; hsa-miR-93-5p; hsa-miR-124-3p; hsa-miR-21-5p; hsa-miR-29a-3p; hsa-miR-148a-3p; hsa-miR-10a-5p; hsa-miR-148b-3p; hsa-miR-615-3p; hsa-miR-505-3p; hsa-miR-30b-5p; hsa-miR-346; hsa-miR-30c-5p; hsa-miR-328-3p
Gene Ontology (miRWalk)	GO0042692 muscle cell differentiation	enriched	0.0409681	26	hsa-miR-29c-3p; hsa-let-7c-5p; hsa-miR-155-5p; hsa-let-7a-5p; hsa-miR-1-3p; hsa-miR-26b-5p; hsa-miR-320a; hsa-miR-106b-5p; hsa-miR-16-5p; hsa-miR-20a-5p; hsa-miR-24-3p; hsa-miR-29b-3p; hsa-miR-93-5p; hsa-miR-124-3p; hsa-miR-21-5p; hsa-miR-223-3p; hsa-miR-29a-3p; hsa-miR-19b-3p; hsa-miR-17-5p; hsa-miR-148b-3p; hsa-miR-149-5p; hsa-miR-92a-3p; hsa-miR-615-3p; hsa-miR-204-5p; hsa-miR-1226-3p; hsa-miR-331-3p
Gene Ontology (miRWalk)	GO0043524 negative regulation of neuron apoptotic process	enriched	0.0409681	65	hsa-miR-29c-3p; hsa-let-7c-5p; hsa-miR-155-5p; hsa-let-7a-5p; hsa-miR-584-5p; hsa-miR-324-3p; hsa-miR-193a-5p; hsa-miR-1-3p; hsa-miR-26b-5p; hsa-miR-320a; hsa-let-7g-5p; hsa-miR-103a-3p; hsa-miR-199b-5p; hsa-miR-106b-5p; hsa-miR-760; hsa-miR-451a; hsa-miR-146a-5p; hsa-miR-425-3p; hsa-miR-107; hsa-miR-16-5p; hsa-miR-20a-5p; hsa-miR-24-3p; hsa-miR-92b-3p; hsa-miR-29b-3p; hsa-miR-671-5p; hsa-miR-365a-3p; hsa-miR-181a-5p; hsa-miR-181d-5p; hsa-miR-93-5p; hsa-miR-181b-5p; hsa-miR-124-3p; hsa-miR-21-5p; hsa-miR-223-3p; hsa-miR-29a-3p; hsa-miR-26a-5p; hsa-miR-19a-3p; hsa-miR-1229-3p; hsa-miR-15a-5p; hsa-miR-20b-5p; hsa-miR-148a-3p; hsa-miR-320c; hsa-miR-19b-3p; hsa-miR-25-3p; hsa-miR-17-5p; hsa-miR-18a-5p; hsa-miR-630; hsa-miR-10a-5p; hsa-miR-15b-5p; hsa-miR-148b-3p; hsa-miR-149-5p; hsa-miR-23a-3p; hsa-miR-504-5p; hsa-miR-92a-3p; hsa-miR-802; hsa-miR-615-3p; hsa-miR-1260b; hsa-miR-30b-5p; hsa-miR-204-5p; hsa-miR-331-3p; hsa-miR-197-3p; hsa-miR-346; hsa-miR-30c-5p; hsa-miR-296-5p; hsa-miR-328-3p; hsa-miR-1296-5p
Gene Ontology (miRWalk)	GO0051149 positive regulation of muscle cell differentiation	enriched	0.0409681	28	hsa-miR-29c-3p; hsa-let-7c-5p; hsa-miR-155-5p; hsa-let-7a-5p; hsa-miR-1-3p; hsa-miR-877-5p; hsa-miR-26b-5p; hsa-miR-320a; hsa-miR-106b-5p; hsa-miR-20a-5p; hsa-miR-24-3p; hsa-miR-29b-3p; hsa-miR-93-5p; hsa-miR-181b-5p; hsa-miR-124-3p; hsa-miR-21-5p; hsa-miR-223-3p; hsa-miR-29a-3p; hsa-miR-25-3p; hsa-miR-17-5p; hsa-miR-148b-3p; hsa-miR-149-5p; hsa-miR-92a-3p; hsa-miR-615-3p; hsa-miR-204-5p; hsa-miR-1226-3p; hsa-miR-331-3p; hsa-miR-197-3p
Gene Ontology (miRWalk)	GO0051924 regulation of calcium ion transport	enriched	0.0409681	24	hsa-miR-29c-3p; hsa-miR-155-5p; hsa-let-7a-5p; hsa-miR-1-3p; hsa-miR-26b-5p; hsa-miR-103a-3p; hsa-miR-451a; hsa-miR-16-5p; hsa-miR-20a-5p; hsa-miR-29b-3p; hsa-miR-365a-3p; hsa-miR-181a-5p; hsa-miR-181d-5p; hsa-miR-181b-5p; hsa-miR-21-5p; hsa-miR-29a-3p; hsa-miR-15a-5p; hsa-miR-148a-3p; hsa-miR-17-5p; hsa-miR-630; hsa-miR-15b-5p; hsa-miR-204-5p; hsa-miR-331-3p; hsa-miR-296-5p
Gene Ontology (miRWalk)	GO0070531 brca1 a complex	enriched	0.0409681	11	hsa-miR-26b-5p; hsa-miR-146a-5p; hsa-miR-107; hsa-miR-16-5p; hsa-miR-24-3p; hsa-miR-181a-5p; hsa-miR-124-3p; hsa-miR-21-5p; hsa-miR-99b-3p; hsa-miR-15a-5p; hsa-miR-92a-3p
Gene Ontology (miRWalk)	GO0016525 negative regulation of angiogenesis	enriched	0.0411334	28	hsa-miR-29c-3p; hsa-miR-155-5p; hsa-let-7a-5p; hsa-miR-584-5p; hsa-miR-1-3p; hsa-miR-26b-5p; hsa-miR-320a; hsa-miR-146a-5p; hsa-miR-16-5p; hsa-miR-20a-5p; hsa-miR-24-3p; hsa-miR-93-5p; hsa-miR-124-3p; hsa-miR-21-5p; hsa-miR-223-3p; hsa-miR-29a-3p; hsa-miR-26a-5p; hsa-miR-19a-3p; hsa-miR-1229-3p; hsa-miR-19b-3p; hsa-miR-17-5p; hsa-miR-10a-5p; hsa-miR-148b-3p; hsa-miR-516a-3p; hsa-miR-92a-3p; hsa-miR-615-3p; hsa-miR-346; hsa-miR-744-3p

Gene Ontology (miRWalk)	GO0043124 negative regulation of i kappab kinase nf kappab cascade	enriched	0.0411334	28	hsa-let-7c-5p; hsa-miR-324-3p; hsa-miR-1-3p; hsa-miR-877-5p; hsa-miR-26b-5p; hsa-miR-18b-5p; hsa-miR-146a-5p; hsa-miR-142-3p; hsa-miR-16-5p; hsa-miR-24-3p; hsa-miR-29b-3p; hsa-miR-181a-5p; hsa-miR-181b-5p; hsa-miR-124-3p; hsa-miR-21-5p; hsa-miR-29a-3p; hsa-miR-26a-5p; hsa-miR-19a-3p; hsa-miR-1229-3p; hsa-miR-15a-5p; hsa-miR-20b-5p; hsa-miR-19b-3p; hsa-miR-18a-5p; hsa-miR-149-5p; hsa-miR-92a-3p; hsa-miR-615-3p; hsa-miR-331-3p; hsa-miR-328-3p
Gene Ontology (miRWalk)	GO0043312 neutrophil degranulation	enriched	0.0411334	5	hsa-miR-155-5p; hsa-miR-1-3p; hsa-miR-26b-5p; hsa-miR-16-5p; hsa-miR-93-5p
Pathways (miRWalk)	hsa04520 Adherens junction	enriched	0.0411403	67	hsa-miR-29c-3p; hsa-let-7c-5p; hsa-miR-155-5p; hsa-let-7a-5p; hsa-miR-324-3p; hsa-miR-1-3p; hsa-miR-877-5p; hsa-miR-26b-5p; hsa-miR-320a; hsa-miR-103a-3p; hsa-miR-1227-3p; hsa-miR-199b-5p; hsa-miR-106b-5p; hsa-miR-760; hsa-miR-146a-5p; hsa-miR-1295a; hsa-miR-548d-3p; hsa-miR-142-3p; hsa-miR-16-5p; hsa-miR-20a-5p; hsa-miR-24-3p; hsa-miR-92b-3p; hsa-miR-29b-3p; hsa-miR-181a-5p; hsa-miR-483-5p; hsa-miR-181d-5p; hsa-miR-93-5p; hsa-miR-181b-5p; hsa-miR-124-3p; hsa-miR-21-5p; hsa-miR-223-3p; hsa-miR-29a-3p; hsa-miR-26a-5p; hsa-miR-99b-3p; hsa-miR-19a-3p; hsa-miR-1229-3p; hsa-miR-15a-5p; hsa-miR-26a-2-3p; hsa-miR-940; hsa-miR-320c; hsa-miR-1224-5p; hsa-miR-19b-3p; hsa-miR-25-3p; hsa-miR-766-3p; hsa-miR-17-5p; hsa-miR-18a-5p; hsa-miR-630; hsa-miR-10a-5p; hsa-miR-15b-5p; hsa-miR-148b-3p; hsa-miR-149-5p; hsa-miR-23a-3p; hsa-miR-92a-3p; hsa-miR-615-3p; hsa-miR-483-3p; hsa-miR-1260b; hsa-miR-130b-5p; hsa-miR-204-5p; hsa-miR-337-3p; hsa-miR-1226-3p; hsa-miR-331-3p; hsa-miR-1914-5p; hsa-miR-197-3p; hsa-miR-346; hsa-miR-34b-3p; hsa-miR-30c-5p; hsa-miR-328-3p
Gene Ontology (miRWalk)	GO0001816 cytokine production	enriched	0.0413646	9	hsa-miR-155-5p; hsa-let-7a-5p; hsa-miR-1-3p; hsa-miR-26b-5p; hsa-miR-760; hsa-miR-184; hsa-miR-92b-3p; hsa-miR-20b-5p; hsa-miR-148b-3p
Gene Ontology (miRWalk)	GO0035025 positive regulation of rho protein signal transduction	enriched	0.0413646	9	hsa-miR-155-5p; hsa-let-7a-5p; hsa-miR-1-3p; hsa-miR-26b-5p; hsa-miR-142-3p; hsa-miR-16-5p; hsa-miR-92b-3p; hsa-miR-92a-3p; hsa-miR-30c-5p
Gene Ontology (miRWalk)	GO0055007 cardiac muscle cell differentiation	enriched	0.0413743	17	hsa-let-7a-5p; hsa-miR-1-3p; hsa-miR-26b-5p; hsa-miR-320a; hsa-miR-106b-5p; hsa-miR-16-5p; hsa-miR-20a-5p; hsa-miR-92b-3p; hsa-miR-181a-5p; hsa-miR-181b-5p; hsa-miR-124-3p; hsa-miR-26a-5p; hsa-miR-1229-3p; hsa-miR-25-3p; hsa-miR-149-5p; hsa-miR-615-3p; hsa-miR-30c-5p
Pathways (miRWalk)	hsa04310 Wnt signaling pathway	enriched	0.0415724	67	hsa-miR-29c-3p; hsa-let-7c-5p; hsa-miR-155-5p; hsa-let-7a-5p; hsa-miR-584-5p; hsa-miR-324-3p; hsa-let-7f-5p; hsa-miR-30d-5p; hsa-miR-1-3p; hsa-miR-877-5p; hsa-miR-26b-5p; hsa-miR-320a; hsa-let-7g-5p; hsa-miR-103a-3p; hsa-miR-106b-5p; hsa-miR-760; hsa-miR-451a; hsa-miR-184; hsa-miR-146a-5p; hsa-miR-142-3p; hsa-miR-16-5p; hsa-miR-20a-5p; hsa-miR-24-3p; hsa-miR-92b-3p; hsa-miR-29b-3p; hsa-miR-365a-3p; hsa-miR-181a-5p; hsa-miR-181d-5p; hsa-miR-93-5p; hsa-miR-181b-5p; hsa-miR-124-3p; hsa-miR-21-5p; hsa-miR-29a-3p; hsa-miR-26a-5p; hsa-miR-19a-3p; hsa-miR-15a-5p; hsa-miR-449c-5p; hsa-miR-148a-3p; hsa-miR-940; hsa-miR-320c; hsa-miR-19b-3p; hsa-miR-25-3p; hsa-miR-17-5p; hsa-miR-18a-5p; hsa-miR-10a-5p; hsa-miR-15b-5p; hsa-miR-148b-3p; hsa-miR-149-5p; hsa-miR-23a-3p; hsa-miR-504-5p; hsa-miR-92a-3p; hsa-miR-615-3p; hsa-miR-505-3p; hsa-miR-483-3p; hsa-miR-1260b; hsa-miR-130b-5p; hsa-miR-204-5p; hsa-miR-337-3p; hsa-miR-1226-3p; hsa-miR-331-3p; hsa-miR-1914-5p; hsa-miR-197-3p; hsa-miR-34b-3p; hsa-miR-30c-5p; hsa-miR-605-5p; hsa-miR-296-5p; hsa-miR-1296-5p

Pathways (miRWalk)	hsa05215 Prostate cancer	enriched	0.0415724	74	hsa-miR-29c-3p; hsa-let-7c-5p; hsa-miR-155-5p; hsa-let-7a-5p; hsa-miR-324-3p; hsa-let-7f-5p; hsa-miR-30d-5p; hsa-miR-1-3p; hsa-miR-26b-5p; hsa-miR-320a; hsa-let-7g-5p; hsa-miR-103a-3p; hsa-miR-199b-5p; hsa-miR-106b-5p; hsa-miR-18b-5p; hsa-miR-760; hsa-miR-451a; hsa-miR-184; hsa-miR-146a-5p; hsa-miR-548d-3p; hsa-miR-107; hsa-miR-16-5p; hsa-miR-20a-5p; hsa-miR-24-3p; hsa-miR-92b-3p; hsa-miR-29b-3p; hsa-miR-365a-3p; hsa-miR-181a-5p; hsa-miR-483-5p; hsa-miR-181d-5p; hsa-miR-93-5p; hsa-miR-181b-5p; hsa-miR-124-3p; hsa-miR-21-5p; hsa-miR-223-3p; hsa-miR-29a-3p; hsa-miR-26a-5p; hsa-miR-19a-3p; hsa-miR-1229-3p; hsa-miR-15a-5p; hsa-miR-520a-3p; hsa-miR-20b-5p; hsa-miR-148a-3p; hsa-miR-19b-3p; hsa-miR-494-3p; hsa-miR-25-3p; hsa-miR-766-3p; hsa-miR-17-5p; hsa-miR-18a-5p; hsa-miR-630; hsa-miR-572; hsa-miR-10a-5p; hsa-miR-15b-5p; hsa-miR-148b-3p; hsa-miR-149-5p; hsa-miR-23a-3p; hsa-miR-504-5p; hsa-miR-92a-3p; hsa-miR-299-5p; hsa-miR-615-3p; hsa-miR-1260b; hsa-miR-30b-5p; hsa-miR-130b-5p; hsa-miR-204-5p; hsa-miR-1226-3p; hsa-miR-331-3p; hsa-miR-346; hsa-miR-34b-3p; hsa-miR-211-5p; hsa-miR-30c-5p; hsa-miR-605-5p; hsa-miR-296-5p; hsa-miR-1296-5p; hsa-miR-885-5p
Gene Ontology (miRWalk)	GO0016575 histone deacetylation	enriched	0.0416363	27	hsa-miR-29c-3p; hsa-let-7c-5p; hsa-let-7a-5p; hsa-miR-1-3p; hsa-miR-26b-5p; hsa-miR-320a; hsa-miR-103a-3p; hsa-miR-142-3p; hsa-miR-16-5p; hsa-miR-24-3p; hsa-miR-92b-3p; hsa-miR-29b-3p; hsa-miR-671-5p; hsa-miR-181a-5p; hsa-miR-93-5p; hsa-miR-181b-5p; hsa-miR-124-3p; hsa-miR-17-5p; hsa-miR-148b-3p; hsa-miR-149-5p; hsa-miR-23a-3p; hsa-miR-92a-3p; hsa-miR-615-3p; hsa-miR-30b-5p; hsa-miR-331-3p; hsa-miR-30c-5p; hsa-miR-328-3p
Diseases (published studies about miRNA profiles from peripheral blood)	COPD upregulated	depleted	0.0416943	19	hsa-miR-875-3p; hsa-miR-498; hsa-miR-1246; hsa-miR-184; hsa-miR-181d-5p; hsa-miR-1267; hsa-miR-576-5p; hsa-miR-23a-3p; hsa-miR-1249-3p; hsa-miR-516a-3p; hsa-miR-574-3p; hsa-miR-130b-5p; hsa-miR-204-5p; hsa-let-7e-3p; hsa-miR-331-3p; hsa-miR-197-3p; hsa-miR-1909-5p; hsa-let-7d-3p; hsa-miR-885-5p
Diseases (published studies about miRNA profiles from peripheral blood)	sarcoidosis downregulated	enriched	0.0416943	35	hsa-miR-155-5p; hsa-let-7a-5p; hsa-let-7f-5p; hsa-miR-26b-5p; hsa-let-7g-5p; hsa-miR-103a-3p; hsa-miR-106b-5p; hsa-miR-18b-5p; hsa-let-7i-5p; hsa-miR-1295a; hsa-miR-107; hsa-miR-16-5p; hsa-miR-525-5p; hsa-miR-20a-5p; hsa-miR-24-3p; hsa-miR-33b-3p; hsa-miR-93-5p; hsa-miR-595; hsa-miR-21-5p; hsa-miR-26a-5p; hsa-miR-1914-3p; hsa-miR-15a-5p; hsa-miR-20b-5p; hsa-miR-148a-3p; hsa-miR-940; hsa-miR-1202; hsa-miR-1228-3p; hsa-miR-17-5p; hsa-miR-634; hsa-miR-18a-5p; hsa-miR-15b-5p; hsa-miR-148b-3p; hsa-let-7g-3p; hsa-miR-631; hsa-miR-566
Gene Ontology (miRWalk)	GO0000018 regulation of dna recombination	enriched	0.0417141	8	hsa-let-7a-5p; hsa-miR-26b-5p; hsa-miR-320a; hsa-miR-103a-3p; hsa-miR-16-5p; hsa-miR-93-5p; hsa-miR-181b-5p; hsa-miR-92a-3p
Gene Ontology (miRWalk)	GO0002237 response to molecule of bacterial origin	enriched	0.0417141	11	hsa-miR-155-5p; hsa-miR-1-3p; hsa-miR-26b-5p; hsa-miR-146a-5p; hsa-miR-24-3p; hsa-miR-93-5p; hsa-miR-124-3p; hsa-miR-21-5p; hsa-miR-29a-3p; hsa-miR-17-5p; hsa-miR-204-5p
Gene Ontology (miRWalk)	GO0002634 regulation of germinal center formation	enriched	0.0417141	11	hsa-let-7c-5p; hsa-miR-155-5p; hsa-miR-877-5p; hsa-miR-26b-5p; hsa-miR-24-3p; hsa-miR-124-3p; hsa-miR-21-5p; hsa-miR-223-3p; hsa-miR-29a-3p; hsa-miR-92a-3p; hsa-miR-30b-5p

Gene Ontology (miRWalk)	GO0005125 cytokine activity	enriched	0.0417141	61	hsa-miR-29c-3p; hsa-miR-155-5p; hsa-let-7a-5p; hsa-miR-324-3p; hsa-let-7f-5p; hsa-miR-1-3p; hsa-miR-877-5p; hsa-miR-26b-5p; hsa-miR-320a; hsa-let-7g-5p; hsa-miR-103a-3p; hsa-miR-1227-3p; hsa-miR-106b-5p; hsa-let-7i-5p; hsa-miR-760; hsa-miR-451a; hsa-miR-146a-5p; hsa-miR-142-3p; hsa-miR-107; hsa-miR-16-5p; hsa-miR-20a-5p; hsa-miR-24-3p; hsa-miR-92b-3p; hsa-miR-29b-3p; hsa-miR-671-5p; hsa-miR-365a-3p; hsa-miR-181a-5p; hsa-miR-181d-5p; hsa-miR-93-5p; hsa-miR-124-3p; hsa-miR-21-5p; hsa-miR-223-3p; hsa-miR-26a-5p; hsa-miR-134-5p; hsa-miR-1229-3p; hsa-miR-15a-5p; hsa-miR-20b-5p; hsa-miR-148a-3p; hsa-miR-520g-3p; hsa-miR-17-5p; hsa-miR-18a-5p; hsa-miR-15b-5p; hsa-miR-148b-3p; hsa-miR-149-5p; hsa-miR-504-5p; hsa-miR-92a-3p; hsa-miR-299-5p; hsa-miR-615-3p; hsa-miR-505-3p; hsa-miR-30b-5p; hsa-miR-204-5p; hsa-miR-1226-3p; hsa-miR-331-3p; hsa-miR-454-5p; hsa-miR-197-3p; hsa-miR-346; hsa-miR-34b-3p; hsa-miR-211-5p; hsa-miR-30c-5p; hsa-miR-328-3p; hsa-miR-1296-5p
Gene Ontology (miRWalk)	GO0009617 response to bacterium	enriched	0.0417141	16	hsa-miR-29c-3p; hsa-miR-155-5p; hsa-let-7a-5p; hsa-miR-1-3p; hsa-miR-26b-5p; hsa-miR-146a-5p; hsa-miR-16-5p; hsa-miR-24-3p; hsa-miR-124-3p; hsa-miR-21-5p; hsa-miR-29a-3p; hsa-miR-26a-5p; hsa-miR-15a-5p; hsa-miR-17-5p; hsa-miR-92a-3p; hsa-miR-505-3p
Gene Ontology (miRWalk)	GO0031643 positive regulation of myelination	enriched	0.0417141	11	hsa-miR-29c-3p; hsa-let-7c-5p; hsa-let-7a-5p; hsa-miR-320a; hsa-miR-103a-3p; hsa-miR-107; hsa-miR-16-5p; hsa-miR-154-5p; hsa-miR-29a-3p; hsa-miR-18a-5p; hsa-miR-130b-5p
Gene Ontology (miRWalk)	GO0042787 protein ubiquitination involved in ubiquitin dependent protein catabolic process	enriched	0.0417141	39	hsa-miR-29c-3p; hsa-let-7c-5p; hsa-miR-155-5p; hsa-let-7a-5p; hsa-miR-324-3p; hsa-miR-1-3p; hsa-miR-877-5p; hsa-miR-26b-5p; hsa-miR-320a; hsa-let-7g-5p; hsa-miR-103a-3p; hsa-miR-106b-5p; hsa-miR-18b-5p; hsa-miR-760; hsa-miR-16-5p; hsa-miR-24-3p; hsa-miR-181a-5p; hsa-miR-93-5p; hsa-miR-181b-5p; hsa-miR-124-3p; hsa-miR-21-5p; hsa-miR-29a-3p; hsa-miR-26a-5p; hsa-miR-19a-3p; hsa-miR-15a-5p; hsa-miR-20b-5p; hsa-miR-19b-3p; hsa-miR-25-3p; hsa-miR-17-5p; hsa-miR-10a-5p; hsa-miR-15b-5p; hsa-miR-149-5p; hsa-miR-504-5p; hsa-miR-92a-3p; hsa-miR-615-3p; hsa-miR-331-3p; hsa-miR-30c-5p; hsa-miR-605-5p; hsa-miR-1296-5p
Pathways (miRWalk)	WP241 One Carbon Metabolism	enriched	0.0417856	28	hsa-miR-29c-3p; hsa-miR-155-5p; hsa-let-7a-5p; hsa-miR-30d-5p; hsa-miR-1-3p; hsa-miR-877-5p; hsa-miR-26b-5p; hsa-miR-320a; hsa-miR-103a-3p; hsa-miR-106b-5p; hsa-miR-16-5p; hsa-miR-24-3p; hsa-miR-29b-3p; hsa-miR-93-5p; hsa-miR-124-3p; hsa-miR-29a-3p; hsa-miR-26a-5p; hsa-miR-148a-3p; hsa-miR-940; hsa-miR-19b-3p; hsa-miR-10a-5p; hsa-miR-15b-5p; hsa-miR-148b-3p; hsa-miR-149-5p; hsa-miR-92a-3p; hsa-miR-615-3p; hsa-miR-1260b; hsa-miR-30b-5p
Pathways (miRWalk)	WP262 EBV LMP1 signaling	enriched	0.0417856	28	hsa-miR-155-5p; hsa-let-7a-5p; hsa-miR-324-3p; hsa-miR-1-3p; hsa-miR-877-5p; hsa-miR-26b-5p; hsa-miR-320a; hsa-miR-103a-3p; hsa-miR-760; hsa-miR-146a-5p; hsa-miR-16-5p; hsa-miR-24-3p; hsa-miR-92b-3p; hsa-miR-124-3p; hsa-miR-21-5p; hsa-miR-223-3p; hsa-miR-26a-5p; hsa-miR-15a-5p; hsa-miR-25-3p; hsa-miR-766-3p; hsa-miR-17-5p; hsa-miR-10a-5p; hsa-miR-15b-5p; hsa-miR-92a-3p; hsa-miR-204-5p; hsa-miR-1226-3p; hsa-miR-30c-5p; hsa-miR-328-3p
Gene Ontology (miRWalk)	GO0000228 nuclear chromosome	enriched	0.0418176	30	hsa-miR-29c-3p; hsa-let-7c-5p; hsa-miR-155-5p; hsa-let-7a-5p; hsa-miR-324-3p; hsa-let-7f-5p; hsa-miR-1-3p; hsa-miR-26b-5p; hsa-let-7g-5p; hsa-miR-103a-3p; hsa-miR-106b-5p; hsa-miR-760; hsa-miR-16-5p; hsa-miR-24-3p; hsa-miR-92b-3p; hsa-miR-93-5p; hsa-miR-124-3p; hsa-miR-21-5p; hsa-miR-26a-5p; hsa-miR-1229-3p; hsa-miR-15a-5p; hsa-miR-3620-3p; hsa-miR-10a-5p; hsa-miR-149-5p;

					hsa-miR-615-3p; hsa-miR-1260b; hsa-miR-1226-3p; hsa-miR-331-3p; hsa-miR-30c-5p; hsa-miR-328-3p
Gene Ontology (miRWalk)	GO0003281 ventricular septum development	enriched	0.0418176	15	hsa-miR-155-5p; hsa-miR-1-3p; hsa-miR-26b-5p; hsa-miR-320a; hsa-miR-103a-3p; hsa-miR-199b-5p; hsa-miR-760; hsa-miR-142-3p; hsa-miR-16-5p; hsa-miR-93-5p; hsa-miR-124-3p; hsa-miR-19b-3p; hsa-miR-23a-3p; hsa-miR-92a-3p; hsa-miR-615-3p
Gene Ontology (miRWalk)	GO0008047 enzyme activator activity	enriched	0.0418176	30	hsa-miR-29c-3p; hsa-miR-155-5p; hsa-let-7a-5p; hsa-miR-324-3p; hsa-miR-30d-5p; hsa-miR-1-3p; hsa-miR-26b-5p; hsa-miR-320a; hsa-miR-16-5p; hsa-miR-20a-5p; hsa-miR-24-3p; hsa-miR-29b-3p; hsa-miR-671-5p; hsa-miR-181a-5p; hsa-miR-93-5p; hsa-miR-124-3p; hsa-miR-21-5p; hsa-miR-26a-5p; hsa-miR-19a-3p; hsa-miR-1229-3p; hsa-miR-19b-3p; hsa-miR-17-5p; hsa-miR-149-5p; hsa-miR-23a-3p; hsa-miR-92a-3p; hsa-miR-615-3p; hsa-miR-30b-5p; hsa-miR-1226-3p; hsa-miR-331-3p; hsa-miR-1296-5p
Gene Ontology (miRWalk)	GO0010467 gene expression	enriched	0.0421102	107	hsa-miR-29c-3p; hsa-miR-22-5p; hsa-let-7c-5p; hsa-miR-155-5p; hsa-miR-193b-5p; hsa-let-7a-5p; hsa-miR-324-3p; hsa-let-7f-5p; hsa-miR-193a-5p; hsa-miR-30d-5p; hsa-miR-1-3p; hsa-miR-877-5p; hsa-miR-26b-5p; hsa-miR-320a; hsa-let-7g-5p; hsa-miR-103a-3p; hsa-miR-1250-5p; hsa-miR-1227-3p; hsa-miR-199b-5p; hsa-miR-106b-5p; hsa-miR-18b-5p; hsa-let-7i-5p; hsa-miR-3188; hsa-miR-760; hsa-miR-1287-5p; hsa-miR-451a; hsa-miR-146a-5p; hsa-miR-1295a; hsa-miR-142-3p; hsa-miR-425-3p; hsa-miR-107; hsa-miR-16-5p; hsa-miR-20a-5p; hsa-miR-24-3p; hsa-miR-92b-3p; hsa-miR-422a; hsa-miR-765; hsa-miR-29b-3p; hsa-miR-154-5p; hsa-miR-671-5p; hsa-miR-365a-3p; hsa-miR-374a-3p; hsa-miR-181a-5p; hsa-miR-483-5p; hsa-miR-181d-5p; hsa-miR-93-5p; hsa-miR-181b-5p; hsa-miR-124-3p; hsa-miR-21-5p; hsa-miR-223-3p; hsa-miR-29a-3p; hsa-miR-26a-5p; hsa-miR-99b-3p; hsa-miR-19a-3p; hsa-miR-1229-3p; hsa-miR-188-5p; hsa-miR-15a-5p; hsa-miR-449c-5p; hsa-miR-1268a; hsa-miR-20b-5p; hsa-miR-148a-3p; hsa-miR-940; hsa-miR-320c; hsa-miR-613; hsa-miR-636; hsa-miR-19b-3p; hsa-miR-1228-3p; hsa-miR-25-3p; hsa-miR-766-3p; hsa-miR-17-5p; hsa-miR-576-5p; hsa-miR-18a-5p; hsa-miR-630; hsa-miR-10a-5p; hsa-miR-15b-5p; hsa-miR-148b-3p; hsa-miR-149-5p; hsa-miR-3605-3p; hsa-miR-663a; hsa-let-7f-1-3p; hsa-miR-23a-3p; hsa-miR-504-5p; hsa-miR-92a-3p; hsa-miR-3679-3p; hsa-miR-1225-5p; hsa-miR-1913; hsa-miR-615-3p; hsa-miR-185-3p; hsa-miR-1237-3p; hsa-miR-505-3p; hsa-miR-483-3p; hsa-miR-1260b; hsa-miR-30b-5p; hsa-miR-130b-5p; hsa-miR-204-5p; hsa-miR-1226-3p; hsa-miR-331-3p; hsa-miR-1914-5p; hsa-miR-1236-3p; hsa-miR-197-3p; hsa-miR-346; hsa-miR-34b-3p; hsa-miR-30c-5p; hsa-miR-605-5p; hsa-miR-328-3p; hsa-miR-1296-5p; hsa-miR-196b-3p
Gene Ontology (miRWalk)	GO0016239 positive regulation of macroautophagy	enriched	0.0422984	25	hsa-let-7c-5p; hsa-miR-155-5p; hsa-let-7a-5p; hsa-miR-324-3p; hsa-let-7f-5p; hsa-miR-877-5p; hsa-miR-26b-5p; hsa-miR-320a; hsa-miR-18b-5p; hsa-miR-16-5p; hsa-miR-20a-5p; hsa-miR-92b-3p; hsa-miR-181a-5p; hsa-miR-93-5p; hsa-miR-181b-5p; hsa-miR-124-3p; hsa-miR-1229-3p; hsa-miR-320c; hsa-miR-17-5p; hsa-miR-18a-5p; hsa-miR-15b-5p; hsa-miR-149-5p; hsa-miR-92a-3p; hsa-miR-615-3p; hsa-miR-331-3p

Pathways (miRWalk)	P00004 Alzheimer disease presenilin pathway	enriched	0.0423733	53	hsa-miR-29c-3p; hsa-let-7c-5p; hsa-miR-155-5p; hsa-let-7a-5p; hsa-miR-324-3p; hsa-miR-1-3p; hsa-miR-877-5p; hsa-miR-26b-5p; hsa-miR-320a; hsa-miR-103a-3p; hsa-miR-1227-3p; hsa-miR-106b-5p; hsa-miR-451a; hsa-miR-146a-5p; hsa-miR-1295a; hsa-miR-107; hsa-miR-16-5p; hsa-miR-20a-5p; hsa-miR-24-3p; hsa-miR-29b-3p; hsa-miR-181a-5p; hsa-miR-93-5p; hsa-miR-124-3p; hsa-miR-21-5p; hsa-miR-29a-3p; hsa-miR-26a-5p; hsa-miR-99b-3p; hsa-miR-19a-3p; hsa-miR-181c-3p; hsa-miR-15a-5p; hsa-miR-940; hsa-miR-3620-3p; hsa-miR-19b-3p; hsa-miR-25-3p; hsa-miR-766-3p; hsa-miR-17-5p; hsa-miR-18a-5p; hsa-miR-10a-5p; hsa-miR-15b-5p; hsa-miR-148b-3p; hsa-miR-149-5p; hsa-miR-92a-3p; hsa-miR-615-3p; hsa-miR-1260b; hsa-miR-130b-5p; hsa-miR-204-5p; hsa-miR-1226-3p; hsa-miR-331-3p; hsa-miR-197-3p; hsa-miR-346; hsa-miR-34b-3p; hsa-miR-30c-5p; hsa-miR-328-3p
Pathways (miRWalk)	hsa01100 Metabolic pathways	enriched	0.0423733	86	hsa-miR-29c-3p; hsa-let-7c-5p; hsa-miR-155-5p; hsa-let-7a-5p; hsa-miR-324-3p; hsa-let-7f-5p; hsa-miR-30d-5p; hsa-miR-1-3p; hsa-miR-877-5p; hsa-miR-26b-5p; hsa-miR-320a; hsa-let-7g-5p; hsa-miR-103a-3p; hsa-miR-106b-5p; hsa-miR-18b-5p; hsa-let-7i-5p; hsa-miR-550a-5p; hsa-miR-760; hsa-miR-184; hsa-miR-146a-5p; hsa-miR-142-3p; hsa-miR-425-3p; hsa-miR-107; hsa-miR-16-5p; hsa-miR-20a-5p; hsa-miR-24-3p; hsa-miR-92b-3p; hsa-miR-422a; hsa-miR-29b-3p; hsa-miR-671-5p; hsa-miR-939-5p; hsa-miR-365a-3p; hsa-miR-374a-3p; hsa-miR-181a-5p; hsa-miR-181d-5p; hsa-miR-93-5p; hsa-miR-181b-5p; hsa-miR-124-3p; hsa-miR-21-5p; hsa-miR-29a-3p; hsa-miR-26a-5p; hsa-miR-99b-3p; hsa-miR-19a-3p; hsa-miR-1229-3p; hsa-miR-1914-3p; hsa-miR-15a-5p; hsa-miR-449c-5p; hsa-miR-1268a; hsa-miR-186-3p; hsa-miR-520a-3p; hsa-miR-148a-3p; hsa-miR-940; hsa-miR-320c; hsa-miR-19b-3p; hsa-miR-25-3p; hsa-miR-766-3p; hsa-miR-17-5p; hsa-miR-18a-5p; hsa-miR-10a-5p; hsa-miR-15b-5p; hsa-miR-762; hsa-miR-148b-3p; hsa-miR-149-5p; hsa-let-7f-1-3p; hsa-miR-23a-3p; hsa-miR-504-5p; hsa-miR-92a-3p; hsa-miR-937-3p; hsa-miR-1225-5p; hsa-miR-615-3p; hsa-miR-505-3p; hsa-miR-629-3p; hsa-miR-1260b; hsa-miR-30b-5p; hsa-miR-130b-5p; hsa-miR-204-5p; hsa-miR-1226-3p; hsa-miR-331-3p; hsa-miR-197-3p; hsa-miR-346; hsa-miR-744-3p; hsa-miR-1909-5p; hsa-miR-30c-5p; hsa-miR-4326; hsa-miR-328-3p; hsa-miR-1296-5p
Gene Ontology (miRWalk)	GO0000187 activation of mapk activity	enriched	0.0424207	51	hsa-let-7c-5p; hsa-miR-155-5p; hsa-let-7a-5p; hsa-miR-324-3p; hsa-let-7f-5p; hsa-miR-193a-5p; hsa-miR-30d-5p; hsa-miR-1-3p; hsa-miR-877-5p; hsa-miR-26b-5p; hsa-miR-320a; hsa-miR-103a-3p; hsa-let-7i-5p; hsa-miR-760; hsa-miR-1287-5p; hsa-miR-146a-5p; hsa-miR-16-5p; hsa-miR-20a-5p; hsa-miR-24-3p; hsa-miR-92b-3p; hsa-miR-29b-3p; hsa-miR-181a-5p; hsa-miR-483-5p; hsa-miR-93-5p; hsa-miR-181b-5p; hsa-miR-124-3p; hsa-miR-21-5p; hsa-miR-29a-3p; hsa-miR-26a-5p; hsa-miR-1229-3p; hsa-miR-15a-5p; hsa-miR-19b-3p; hsa-miR-766-3p; hsa-miR-17-5p; hsa-miR-18a-5p; hsa-miR-10a-5p; hsa-miR-15b-5p; hsa-miR-148b-3p; hsa-miR-663a; hsa-miR-23a-3p; hsa-miR-92a-3p; hsa-miR-615-3p; hsa-miR-1260b; hsa-miR-30b-5p; hsa-miR-130b-5p; hsa-miR-204-5p; hsa-miR-1226-3p; hsa-miR-197-3p; hsa-miR-34b-3p; hsa-miR-30c-5p; hsa-miR-328-3p
Gene Ontology (miRWalk)	GO0004518 nuclease activity	enriched	0.0424207	8	hsa-let-7f-5p; hsa-miR-1-3p; hsa-miR-26b-5p; hsa-miR-760; hsa-miR-16-5p; hsa-miR-93-5p; hsa-miR-124-3p; hsa-miR-1260a

Gene Ontology (miRWalk)	GO0005506 iron ion binding	enriched	0.0424207	42	hsa-miR-155-5p; hsa-let-7a-5p; hsa-miR-324-3p; hsa-let-7f-5p; hsa-miR-1-3p; hsa-miR-877-5p; hsa-miR-26b-5p; hsa-miR-320a; hsa-miR-103a-3p; hsa-let-7i-5p; hsa-miR-107; hsa-miR-16-5p; hsa-miR-20a-5p; hsa-miR-24-3p; hsa-miR-92b-3p; hsa-miR-422a; hsa-miR-29b-3p; hsa-miR-671-5p; hsa-miR-181a-5p; hsa-miR-93-5p; hsa-miR-124-3p; hsa-miR-21-5p; hsa-miR-29a-3p; hsa-miR-26a-5p; hsa-miR-19a-3p; hsa-miR-1229-3p; hsa-miR-148a-3p; hsa-miR-320c; hsa-miR-19b-3p; hsa-miR-17-5p; hsa-miR-10a-5p; hsa-miR-15b-5p; hsa-miR-148b-3p; hsa-miR-149-5p; hsa-miR-23a-3p; hsa-miR-92a-3p; hsa-miR-615-3p; hsa-miR-1260b; hsa-miR-331-3p; hsa-miR-197-3p; hsa-miR-30c-5p; hsa-miR-328-3p
Gene Ontology (miRWalk)	GO0006614 srp dependent cotranslational protein targeting to membrane	enriched	0.0424207	71	hsa-miR-22-5p; hsa-let-7c-5p; hsa-miR-155-5p; hsa-miR-193b-5p; hsa-let-7a-5p; hsa-miR-324-3p; hsa-miR-193a-5p; hsa-miR-30d-5p; hsa-miR-1-3p; hsa-miR-877-5p; hsa-miR-26b-5p; hsa-miR-320a; hsa-let-7g-5p; hsa-miR-103a-3p; hsa-miR-1250-5p; hsa-miR-1227-3p; hsa-miR-106b-5p; hsa-let-7i-5p; hsa-miR-3188; hsa-miR-760; hsa-miR-107; hsa-miR-16-5p; hsa-miR-20a-5p; hsa-miR-24-3p; hsa-miR-92b-3p; hsa-miR-422a; hsa-miR-29b-3p; hsa-miR-671-5p; hsa-miR-365a-3p; hsa-miR-181a-5p; hsa-miR-181d-5p; hsa-miR-93-5p; hsa-miR-181b-5p; hsa-miR-124-3p; hsa-miR-21-5p; hsa-miR-29a-3p; hsa-miR-26a-5p; hsa-miR-99b-3p; hsa-miR-1229-3p; hsa-miR-148a-3p; hsa-miR-940; hsa-miR-636; hsa-miR-25-3p; hsa-miR-766-3p; hsa-miR-17-5p; hsa-miR-576-5p; hsa-miR-18a-5p; hsa-miR-10a-5p; hsa-miR-15b-5p; hsa-miR-148b-3p; hsa-miR-149-5p; hsa-miR-3605-3p; hsa-miR-23a-3p; hsa-miR-504-5p; hsa-miR-92a-3p; hsa-miR-615-3p; hsa-miR-1237-3p; hsa-miR-505-3p; hsa-miR-1260b; hsa-miR-30b-5p; hsa-miR-130b-5p; hsa-miR-204-5p; hsa-miR-1226-3p; hsa-miR-331-3p; hsa-miR-1914-5p; hsa-miR-197-3p; hsa-miR-346; hsa-miR-30c-5p; hsa-miR-328-3p; hsa-miR-1296-5p; hsa-miR-196b-3p
Gene Ontology (miRWalk)	GO0006744 ubiquinone biosynthetic process	enriched	0.0424207	8	hsa-miR-1-3p; hsa-miR-26b-5p; hsa-miR-320a; hsa-miR-106b-5p; hsa-miR-16-5p; hsa-miR-93-5p; hsa-miR-124-3p; hsa-miR-148b-3p
Gene Ontology (miRWalk)	GO0008203 cholesterol metabolic process	enriched	0.0424207	35	hsa-let-7c-5p; hsa-miR-155-5p; hsa-let-7a-5p; hsa-miR-324-3p; hsa-miR-193a-5p; hsa-miR-1-3p; hsa-miR-877-5p; hsa-miR-26b-5p; hsa-miR-320a; hsa-miR-106b-5p; hsa-miR-146a-5p; hsa-miR-16-5p; hsa-miR-24-3p; hsa-miR-548d-5p; hsa-miR-92b-3p; hsa-miR-365a-3p; hsa-miR-93-5p; hsa-miR-181b-5p; hsa-miR-124-3p; hsa-miR-21-5p; hsa-miR-26a-5p; hsa-miR-19a-3p; hsa-miR-19b-3p; hsa-miR-25-3p; hsa-miR-10a-5p; hsa-miR-148b-3p; hsa-miR-149-5p; hsa-miR-26b-3p; hsa-miR-92a-3p; hsa-miR-615-3p; hsa-miR-1260b; hsa-miR-30b-5p; hsa-miR-1226-3p; hsa-miR-331-3p; hsa-miR-346
Gene Ontology (miRWalk)	GO0010039 response to iron ion	enriched	0.0424207	35	hsa-miR-29c-3p; hsa-miR-155-5p; hsa-let-7a-5p; hsa-let-7f-5p; hsa-miR-1-3p; hsa-miR-26b-5p; hsa-miR-103a-3p; hsa-miR-106b-5p; hsa-miR-451a; hsa-miR-16-5p; hsa-miR-20a-5p; hsa-miR-24-3p; hsa-miR-29b-3p; hsa-miR-365a-3p; hsa-miR-181a-5p; hsa-miR-181d-5p; hsa-miR-93-5p; hsa-miR-181b-5p; hsa-miR-124-3p; hsa-miR-21-5p; hsa-miR-29a-3p; hsa-miR-19a-3p; hsa-miR-15a-5p; hsa-miR-148a-3p; hsa-miR-17-5p; hsa-miR-630; hsa-miR-15b-5p; hsa-miR-148b-3p; hsa-miR-92a-3p; hsa-miR-937-3p; hsa-miR-204-5p; hsa-miR-331-3p; hsa-miR-34b-3p; hsa-miR-30c-5p; hsa-miR-296-5p

Gene Ontology (miRWalk)	GO0016702 oxidoreductase activity acting on single donors with incorporation of molecular oxygen incorporation of two atoms of oxygen	enriched	0.0424207	40	hsa-miR-29c-3p; hsa-let-7c-5p; hsa-miR-155-5p; hsa-let-7a-5p; hsa-miR-324-3p; hsa-miR-1-3p; hsa-miR-877-5p; hsa-miR-26b-5p; hsa-miR-320a; hsa-let-7i-5p; hsa-miR-550a-5p; hsa-miR-760; hsa-miR-425-3p; hsa-miR-16-5p; hsa-miR-20a-5p; hsa-miR-92b-3p; hsa-miR-29b-3p; hsa-miR-181a-5p; hsa-miR-93-5p; hsa-miR-124-3p; hsa-miR-21-5p; hsa-miR-29a-3p; hsa-miR-26a-5p; hsa-miR-99b-3p; hsa-miR-1229-3p; hsa-miR-320c; hsa-miR-19b-3p; hsa-miR-766-3p; hsa-miR-17-5p; hsa-miR-148b-3p; hsa-miR-149-5p; hsa-miR-23a-3p; hsa-miR-92a-3p; hsa-miR-615-3p; hsa-miR-505-3p; hsa-miR-1260b; hsa-miR-1226-3p; hsa-miR-331-3p; hsa-miR-605-5p; hsa-miR-328-3p
Gene Ontology (miRWalk)	GO0030335 positive regulation of cell migration	enriched	0.0424207	54	hsa-miR-29c-3p; hsa-let-7c-5p; hsa-miR-155-5p; hsa-let-7a-5p; hsa-miR-324-3p; hsa-let-7f-5p; hsa-miR-1-3p; hsa-miR-877-5p; hsa-miR-26b-5p; hsa-miR-320a; hsa-miR-103a-3p; hsa-miR-106b-5p; hsa-miR-760; hsa-miR-146a-5p; hsa-miR-142-3p; hsa-miR-425-3p; hsa-miR-107; hsa-miR-16-5p; hsa-miR-20a-5p; hsa-miR-24-3p; hsa-miR-29b-3p; hsa-miR-181a-5p; hsa-miR-93-5p; hsa-miR-181b-5p; hsa-miR-124-3p; hsa-miR-21-5p; hsa-miR-223-3p; hsa-miR-29a-3p; hsa-miR-26a-5p; hsa-miR-134-5p; hsa-miR-19a-3p; hsa-miR-15a-5p; hsa-miR-20b-5p; hsa-miR-26a-2-3p; hsa-miR-148a-3p; hsa-miR-19b-3p; hsa-miR-520g-3p; hsa-miR-25-3p; hsa-miR-17-5p; hsa-miR-18a-5p; hsa-miR-630; hsa-miR-10a-5p; hsa-miR-15b-5p; hsa-miR-148b-3p; hsa-miR-149-5p; hsa-miR-504-5p; hsa-miR-92a-3p; hsa-miR-615-3p; hsa-miR-505-3p; hsa-miR-30b-5p; hsa-miR-204-5p; hsa-miR-1226-3p; hsa-miR-34b-3p; hsa-miR-30c-5p
Gene Ontology (miRWalk)	GO0032227 negative regulation of synaptic transmission dopaminergic	enriched	0.0424207	8	hsa-let-7c-5p; hsa-let-7a-5p; hsa-miR-26b-5p; hsa-miR-320a; hsa-miR-16-5p; hsa-miR-181a-5p; hsa-miR-124-3p; hsa-miR-17-5p
Gene Ontology (miRWalk)	GO0035810 positive regulation of urine volume	enriched	0.0424207	8	hsa-miR-155-5p; hsa-let-7a-5p; hsa-miR-1-3p; hsa-miR-26b-5p; hsa-miR-320a; hsa-miR-16-5p; hsa-miR-124-3p; hsa-miR-204-5p
Gene Ontology (miRWalk)	GO0040008 regulation of growth	enriched	0.0424207	40	hsa-miR-29c-3p; hsa-let-7c-5p; hsa-miR-155-5p; hsa-let-7a-5p; hsa-miR-324-3p; hsa-let-7f-5p; hsa-miR-1-3p; hsa-miR-26b-5p; hsa-let-7g-5p; hsa-miR-103a-3p; hsa-miR-106b-5p; hsa-let-7i-5p; hsa-miR-760; hsa-miR-142-3p; hsa-miR-16-5p; hsa-miR-20a-5p; hsa-miR-24-3p; hsa-miR-92b-3p; hsa-miR-181a-5p; hsa-miR-93-5p; hsa-miR-124-3p; hsa-miR-21-5p; hsa-miR-26a-5p; hsa-miR-19a-3p; hsa-miR-1229-3p; hsa-miR-19b-3p; hsa-miR-25-3p; hsa-miR-766-3p; hsa-miR-17-5p; hsa-miR-18a-5p; hsa-miR-10a-5p; hsa-miR-148b-3p; hsa-miR-149-5p; hsa-miR-92a-3p; hsa-miR-615-3p; hsa-miR-30b-5p; hsa-miR-204-5p; hsa-miR-331-3p; hsa-miR-197-3p; hsa-miR-30c-5p
Gene Ontology (miRWalk)	GO0070652 haus complex	enriched	0.0424207	8	hsa-miR-26b-5p; hsa-miR-320a; hsa-miR-103a-3p; hsa-miR-16-5p; hsa-miR-20a-5p; hsa-miR-93-5p; hsa-miR-124-3p; hsa-miR-92a-3p
Gene Ontology (miRWalk)	GO0071222 cellular response to lipopolysaccharide	enriched	0.0424207	35	hsa-let-7c-5p; hsa-miR-155-5p; hsa-miR-1-3p; hsa-miR-26b-5p; hsa-miR-320a; hsa-miR-103a-3p; hsa-miR-199b-5p; hsa-let-7i-5p; hsa-miR-146a-5p; hsa-miR-142-3p; hsa-miR-16-5p; hsa-miR-20a-5p; hsa-miR-24-3p; hsa-miR-29b-3p; hsa-miR-181a-5p; hsa-miR-93-5p; hsa-miR-124-3p; hsa-miR-21-5p; hsa-miR-223-3p; hsa-miR-29a-3p; hsa-miR-26a-5p; hsa-miR-19a-3p; hsa-miR-15a-5p; hsa-miR-613; hsa-miR-19b-3p; hsa-miR-25-3p; hsa-miR-17-5p; hsa-miR-15b-5p; hsa-miR-23a-3p; hsa-miR-92a-3p; hsa-miR-615-3p; hsa-miR-204-5p; hsa-miR-1226-3p; hsa-miR-30c-5p; hsa-miR-1296-5p
Gene Ontology (miRWalk)	GO2001022 positive regulation of response to dna damage stimulus	enriched	0.0424207	18	hsa-miR-22-5p; hsa-let-7c-5p; hsa-let-7a-5p; hsa-let-7f-5p; hsa-miR-26b-5p; hsa-miR-320a; hsa-let-7g-5p; hsa-miR-760; hsa-miR-451a; hsa-miR-20a-5p; hsa-miR-24-3p; hsa-miR-21-5p; hsa-miR-26a-5p; hsa-miR-449c-5p; hsa-miR-17-5p; hsa-miR-92a-3p; hsa-miR-615-3p; hsa-miR-34b-3p

Gene Ontology (miRWalk)	GO0007611 learning or memory	enriched	0.0424923	36	hsa-let-7c-5p; hsa-miR-1-3p; hsa-miR-877-5p; hsa-miR-26b-5p; hsa-miR-103a-3p; hsa-miR-106b-5p; hsa-miR-760; hsa-miR-142-3p; hsa-miR-107; hsa-miR-16-5p; hsa-miR-20a-5p; hsa-miR-24-3p; hsa-miR-29b-3p; hsa-miR-671-5p; hsa-miR-93-5p; hsa-miR-181b-5p; hsa-miR-124-3p; hsa-miR-21-5p; hsa-miR-223-3p; hsa-miR-29a-3p; hsa-miR-26a-5p; hsa-miR-19a-3p; hsa-miR-1229-3p; hsa-miR-19b-3p; hsa-miR-494-3p; hsa-miR-17-5p; hsa-miR-18a-5p; hsa-miR-15b-5p; hsa-miR-148b-3p; hsa-miR-149-5p; hsa-miR-23a-3p; hsa-miR-92a-3p; hsa-miR-505-3p; hsa-miR-331-3p; hsa-miR-346; hsa-miR-1296-5p
Gene Ontology (miRWalk)	GO0016874 ligase activity	enriched	0.0424923	36	hsa-miR-29c-3p; hsa-let-7c-5p; hsa-miR-155-5p; hsa-let-7a-5p; hsa-miR-324-3p; hsa-miR-1-3p; hsa-miR-877-5p; hsa-miR-26b-5p; hsa-miR-320a; hsa-let-7g-5p; hsa-miR-103a-3p; hsa-miR-146a-5p; hsa-miR-142-3p; hsa-miR-16-5p; hsa-miR-20a-5p; hsa-miR-24-3p; hsa-miR-92b-3p; hsa-miR-181a-5p; hsa-miR-93-5p; hsa-miR-124-3p; hsa-miR-21-5p; hsa-miR-26a-5p; hsa-miR-1229-3p; hsa-miR-148a-3p; hsa-miR-17-5p; hsa-miR-18a-5p; hsa-miR-10a-5p; hsa-miR-15b-5p; hsa-miR-149-5p; hsa-miR-92a-3p; hsa-miR-615-3p; hsa-miR-574-3p; hsa-miR-130b-5p; hsa-miR-331-3p; hsa-miR-197-3p; hsa-miR-328-3p
Gene Ontology (miRWalk)	GO0042995 cell projection	enriched	0.0424923	36	hsa-let-7c-5p; hsa-miR-155-5p; hsa-let-7a-5p; hsa-miR-324-3p; hsa-miR-1-3p; hsa-miR-26b-5p; hsa-miR-103a-3p; hsa-miR-106b-5p; hsa-miR-146a-5p; hsa-miR-107; hsa-miR-16-5p; hsa-miR-20a-5p; hsa-miR-24-3p; hsa-miR-92b-3p; hsa-miR-29b-3p; hsa-miR-93-5p; hsa-miR-181b-5p; hsa-miR-124-3p; hsa-miR-21-5p; hsa-miR-29a-3p; hsa-miR-26a-5p; hsa-miR-19a-3p; hsa-miR-1229-3p; hsa-miR-19b-3p; hsa-miR-494-3p; hsa-miR-17-5p; hsa-miR-18a-5p; hsa-miR-10a-5p; hsa-miR-148b-3p; hsa-miR-149-5p; hsa-miR-23a-3p; hsa-miR-92a-3p; hsa-miR-299-5p; hsa-miR-615-3p; hsa-miR-505-3p; hsa-miR-331-3p
Gene Ontology (miRWalk)	GO0043234 protein complex	enriched	0.0424923	72	hsa-miR-29c-3p; hsa-miR-22-5p; hsa-let-7c-5p; hsa-miR-155-5p; hsa-let-7a-5p; hsa-miR-324-3p; hsa-let-7f-5p; hsa-miR-30d-5p; hsa-miR-1-3p; hsa-miR-877-5p; hsa-miR-26b-5p; hsa-miR-320a; hsa-let-7g-5p; hsa-miR-103a-3p; hsa-miR-199b-5p; hsa-miR-106b-5p; hsa-miR-18b-5p; hsa-let-7i-5p; hsa-miR-760; hsa-miR-451a; hsa-miR-146a-5p; hsa-miR-142-3p; hsa-miR-425-3p; hsa-miR-107; hsa-miR-16-5p; hsa-miR-20a-5p; hsa-miR-24-3p; hsa-miR-92b-3p; hsa-miR-671-5p; hsa-miR-181a-5p; hsa-miR-93-5p; hsa-miR-181b-5p; hsa-miR-124-3p; hsa-miR-595; hsa-miR-21-5p; hsa-miR-223-3p; hsa-miR-26a-5p; hsa-miR-99b-3p; hsa-miR-19a-3p; hsa-miR-1229-3p; hsa-miR-15a-5p; hsa-miR-449c-5p; hsa-miR-940; hsa-miR-19b-3p; hsa-miR-25-3p; hsa-miR-766-3p; hsa-miR-17-5p; hsa-miR-18a-5p; hsa-miR-10a-5p; hsa-miR-15b-5p; hsa-miR-148b-3p; hsa-miR-149-5p; hsa-miR-23a-3p; hsa-miR-504-5p; hsa-miR-92a-3p; hsa-miR-802; hsa-miR-615-3p; hsa-miR-521; hsa-miR-505-3p; hsa-miR-483-3p; hsa-miR-1260b; hsa-miR-30b-5p; hsa-miR-130b-5p; hsa-let-7g-3p; hsa-miR-204-5p; hsa-miR-1226-3p; hsa-miR-331-3p; hsa-miR-197-3p; hsa-miR-34b-3p; hsa-miR-30c-5p; hsa-miR-605-5p; hsa-miR-1296-5p
Gene Ontology (miRWalk)	GO0035176 social behavior	enriched	0.0426362	33	hsa-miR-155-5p; hsa-let-7a-5p; hsa-miR-1-3p; hsa-miR-26b-5p; hsa-let-7g-5p; hsa-miR-103a-3p; hsa-miR-106b-5p; hsa-miR-425-3p; hsa-miR-107; hsa-miR-16-5p; hsa-miR-20a-5p; hsa-miR-29b-3p; hsa-miR-181a-5p; hsa-miR-181d-5p; hsa-miR-93-5p; hsa-miR-181b-5p; hsa-miR-124-3p; hsa-miR-21-5p; hsa-miR-29a-3p; hsa-miR-26a-5p; hsa-miR-19a-3p; hsa-miR-19b-3p; hsa-miR-494-3p; hsa-miR-17-5p; hsa-miR-18a-5p; hsa-miR-10a-5p; hsa-miR-148b-3p; hsa-miR-23a-3p; hsa-miR-92a-3p; hsa-miR-802; hsa-miR-615-3p; hsa-miR-204-5p; hsa-miR-331-3p

Gene Ontology (miRWalk)	GO0006695 cholesterol biosynthetic process	enriched	0.0428301	29	hsa-miR-29c-3p; hsa-miR-155-5p; hsa-let-7a-5p; hsa-miR-324-3p; hsa-miR-1-3p; hsa-miR-26b-5p; hsa-miR-103a-3p; hsa-miR-106b-5p; hsa-miR-760; hsa-miR-142-3p; hsa-miR-107; hsa-miR-20a-5p; hsa-miR-24-3p; hsa-miR-92b-3p; hsa-miR-29b-3p; hsa-miR-181a-5p; hsa-miR-124-3p; hsa-miR-26a-5p; hsa-miR-19a-3p; hsa-miR-148a-3p; hsa-miR-19b-3p; hsa-miR-18a-5p; hsa-miR-10a-5p; hsa-miR-15b-5p; hsa-miR-148b-3p; hsa-miR-149-5p; hsa-miR-92a-3p; hsa-miR-615-3p; hsa-miR-1260b
Gene Ontology (miRWalk)	GO0006869 lipid transport	enriched	0.0428301	29	hsa-miR-155-5p; hsa-miR-324-3p; hsa-miR-1-3p; hsa-miR-877-5p; hsa-miR-26b-5p; hsa-miR-103a-3p; hsa-miR-1227-3p; hsa-miR-146a-5p; hsa-miR-16-5p; hsa-miR-548d-5p; hsa-miR-92b-3p; hsa-miR-29b-3p; hsa-miR-365a-3p; hsa-miR-181a-5p; hsa-miR-93-5p; hsa-miR-181b-5p; hsa-miR-124-3p; hsa-miR-21-5p; hsa-miR-19a-3p; hsa-miR-19b-3p; hsa-miR-148b-3p; hsa-miR-149-5p; hsa-miR-23a-3p; hsa-miR-92a-3p; hsa-miR-615-3p; hsa-miR-505-3p; hsa-miR-1260b; hsa-miR-30c-5p; hsa-miR-1296-5p
Gene Ontology (miRWalk)	GO0016591 dna directed rna polymerase ii holoenzyme	enriched	0.0428395	17	hsa-let-7c-5p; hsa-miR-155-5p; hsa-miR-324-3p; hsa-miR-1-3p; hsa-miR-26b-5p; hsa-miR-320a; hsa-miR-103a-3p; hsa-miR-16-5p; hsa-miR-20a-5p; hsa-miR-24-3p; hsa-miR-181b-5p; hsa-miR-124-3p; hsa-miR-17-5p; hsa-miR-10a-5p; hsa-miR-148b-3p; hsa-miR-92a-3p; hsa-miR-331-3p
Gene Ontology (miRWalk)	GO0030511 positive regulation of transforming growth factor beta receptor signaling pathway	enriched	0.0429631	27	hsa-miR-155-5p; hsa-let-7a-5p; hsa-miR-1-3p; hsa-miR-26b-5p; hsa-miR-320a; hsa-miR-103a-3p; hsa-miR-760; hsa-miR-146a-5p; hsa-miR-16-5p; hsa-miR-20a-5p; hsa-miR-24-3p; hsa-miR-92b-3p; hsa-miR-181a-5p; hsa-miR-93-5p; hsa-miR-124-3p; hsa-miR-21-5p; hsa-miR-26a-5p; hsa-miR-19a-3p; hsa-miR-320c; hsa-miR-19b-3p; hsa-miR-25-3p; hsa-miR-17-5p; hsa-miR-18a-5p; hsa-miR-149-5p; hsa-miR-92a-3p; hsa-miR-615-3p; hsa-miR-483-3p
Pathways (miRWalk)	WP500 Glycogen Metabolism	enriched	0.0433831	26	hsa-let-7c-5p; hsa-miR-155-5p; hsa-let-7a-5p; hsa-miR-1-3p; hsa-miR-26b-5p; hsa-miR-320a; hsa-miR-103a-3p; hsa-miR-106b-5p; hsa-miR-142-3p; hsa-miR-16-5p; hsa-miR-20a-5p; hsa-miR-24-3p; hsa-miR-365a-3p; hsa-miR-93-5p; hsa-miR-124-3p; hsa-miR-26a-5p; hsa-miR-15a-5p; hsa-miR-25-3p; hsa-miR-17-5p; hsa-miR-10a-5p; hsa-miR-148b-3p; hsa-miR-149-5p; hsa-miR-92a-3p; hsa-miR-615-3p; hsa-miR-1914-5p; hsa-miR-197-3p
Gene Ontology (miRWalk)	GO0042310 vasoconstriction	enriched	0.043649	5	hsa-miR-155-5p; hsa-miR-1-3p; hsa-miR-26b-5p; hsa-miR-103a-3p; hsa-miR-124-3p
Gene Ontology (miRWalk)	GO0007517 muscle organ development	enriched	0.0439363	39	hsa-miR-29c-3p; hsa-let-7c-5p; hsa-miR-155-5p; hsa-let-7a-5p; hsa-miR-324-3p; hsa-let-7f-5p; hsa-miR-30d-5p; hsa-miR-1-3p; hsa-miR-877-5p; hsa-miR-26b-5p; hsa-miR-320a; hsa-miR-103a-3p; hsa-miR-106b-5p; hsa-miR-16-5p; hsa-miR-20a-5p; hsa-miR-92b-3p; hsa-miR-181a-5p; hsa-miR-93-5p; hsa-miR-181b-5p; hsa-miR-124-3p; hsa-miR-21-5p; hsa-miR-223-3p; hsa-miR-29a-3p; hsa-miR-1229-3p; hsa-miR-940; hsa-miR-19b-3p; hsa-miR-25-3p; hsa-miR-17-5p; hsa-miR-18a-5p; hsa-miR-15b-5p; hsa-miR-148b-3p; hsa-miR-149-5p; hsa-miR-92a-3p; hsa-miR-615-3p; hsa-miR-505-3p; hsa-miR-204-5p; hsa-miR-1226-3p; hsa-miR-331-3p; hsa-miR-211-5p
Gene Ontology (miRWalk)	GO0001957 intramembranous ossification	enriched	0.0440397	5	hsa-miR-29c-3p; hsa-miR-103a-3p; hsa-miR-16-5p; hsa-miR-29b-3p; hsa-miR-124-3p
Gene Ontology (miRWalk)	GO0005179 hormone activity	enriched	0.0440397	18	hsa-miR-155-5p; hsa-let-7a-5p; hsa-let-7f-5p; hsa-miR-1-3p; hsa-miR-877-5p; hsa-miR-26b-5p; hsa-miR-142-3p; hsa-miR-16-5p; hsa-miR-92b-3p; hsa-miR-181a-5p; hsa-miR-93-5p; hsa-miR-124-3p; hsa-miR-26a-5p; hsa-miR-10a-5p; hsa-miR-148b-3p; hsa-miR-1260b; hsa-miR-30b-5p; hsa-miR-30c-5p
Gene Ontology (miRWalk)	GO0005319 lipid transporter activity	enriched	0.0440397	5	hsa-miR-1-3p; hsa-miR-26b-5p; hsa-miR-16-5p; hsa-miR-181a-5p; hsa-miR-124-3p

Gene Ontology (miRWalk)	GO0005525 gtp binding	enriched	0.0440397	70	hsa-miR-29c-3p; hsa-miR-22-5p; hsa-let-7c-5p; hsa-miR-155-5p; hsa-let-7a-5p; hsa-miR-324-3p; hsa-let-7f-5p; hsa-miR-30d-5p; hsa-miR-1-3p; hsa-miR-877-5p; hsa-miR-26b-5p; hsa-miR-320a; hsa-let-7g-5p; hsa-miR-103a-3p; hsa-miR-1250-5p; hsa-miR-106b-5p; hsa-miR-760; hsa-miR-451a; hsa-miR-146a-5p; hsa-miR-142-3p; hsa-miR-107; hsa-miR-16-5p; hsa-miR-20a-5p; hsa-miR-24-3p; hsa-miR-92b-3p; hsa-miR-29b-3p; hsa-miR-365a-3p; hsa-miR-181a-5p; hsa-miR-181d-5p; hsa-miR-93-5p; hsa-miR-181b-5p; hsa-miR-124-3p; hsa-miR-21-5p; hsa-miR-223-3p; hsa-miR-29a-3p; hsa-miR-26a-5p; hsa-miR-99b-3p; hsa-miR-19a-3p; hsa-miR-1229-3p; hsa-miR-15a-5p; hsa-miR-148a-3p; hsa-miR-940; hsa-miR-320c; hsa-miR-19b-3p; hsa-miR-1228-3p; hsa-miR-25-3p; hsa-miR-17-5p; hsa-miR-18a-5p; hsa-miR-10a-5p; hsa-miR-15b-5p; hsa-miR-148b-3p; hsa-miR-149-5p; hsa-miR-23a-3p; hsa-miR-92a-3p; hsa-miR-615-3p; hsa-miR-505-3p; hsa-miR-1260b; hsa-miR-30b-5p; hsa-miR-130b-5p; hsa-miR-204-5p; hsa-miR-337-3p; hsa-miR-1226-3p; hsa-miR-331-3p; hsa-miR-197-3p; hsa-miR-211-5p; hsa-miR-30c-5p; hsa-miR-605-5p; hsa-miR-4326; hsa-miR-328-3p; hsa-miR-1296-5p
Gene Ontology (miRWalk)	GO0006725 cellular aromatic compound metabolic process	enriched	0.0440397	5	hsa-miR-155-5p; hsa-miR-1-3p; hsa-miR-16-5p; hsa-miR-24-3p; hsa-miR-124-3p
Gene Ontology (miRWalk)	GO0006796 phosphate containing compound metabolic process	enriched	0.0440397	13	hsa-miR-155-5p; hsa-let-7a-5p; hsa-miR-1-3p; hsa-miR-26b-5p; hsa-miR-103a-3p; hsa-miR-16-5p; hsa-miR-24-3p; hsa-miR-365a-3p; hsa-miR-124-3p; hsa-miR-21-5p; hsa-miR-10a-5p; hsa-miR-15b-5p; hsa-miR-92a-3p
Gene Ontology (miRWalk)	GO0015014 heparan sulfate proteoglycan biosynthetic process polysaccharide chain biosynthetic process	enriched	0.0440397	5	hsa-miR-1-3p; hsa-miR-26b-5p; hsa-miR-320a; hsa-miR-16-5p; hsa-miR-124-3p
Gene Ontology (miRWalk)	GO0030010 establishment of cell polarity	enriched	0.0440397	18	hsa-let-7c-5p; hsa-miR-155-5p; hsa-miR-1-3p; hsa-miR-26b-5p; hsa-miR-320a; hsa-miR-103a-3p; hsa-miR-106b-5p; hsa-miR-16-5p; hsa-miR-20a-5p; hsa-miR-93-5p; hsa-miR-124-3p; hsa-miR-21-5p; hsa-miR-26a-5p; hsa-miR-19b-3p; hsa-miR-17-5p; hsa-miR-10a-5p; hsa-miR-92a-3p; hsa-miR-130b-5p
Gene Ontology (miRWalk)	GO0032964 collagen biosynthetic process	enriched	0.0440397	5	hsa-miR-29c-3p; hsa-miR-1-3p; hsa-miR-26b-5p; hsa-miR-29b-3p; hsa-miR-124-3p
Gene Ontology (miRWalk)	GO0033235 positive regulation of protein sumoylation	enriched	0.0440397	13	hsa-miR-155-5p; hsa-miR-1-3p; hsa-miR-320a; hsa-let-7g-5p; hsa-miR-107; hsa-miR-16-5p; hsa-miR-24-3p; hsa-miR-29b-3p; hsa-miR-124-3p; hsa-miR-21-5p; hsa-miR-10a-5p; hsa-miR-615-3p; hsa-miR-30c-5p
Gene Ontology (miRWalk)	GO0042622 photoreceptor outer segment membrane	enriched	0.0440397	5	hsa-miR-1-3p; hsa-miR-26b-5p; hsa-miR-142-3p; hsa-miR-93-5p; hsa-miR-124-3p
Gene Ontology (miRWalk)	GO0043589 skin morphogenesis	enriched	0.0440397	13	hsa-miR-29c-3p; hsa-miR-155-5p; hsa-miR-324-3p; hsa-miR-1-3p; hsa-miR-26b-5p; hsa-miR-320a; hsa-let-7g-5p; hsa-miR-29b-3p; hsa-miR-124-3p; hsa-miR-21-5p; hsa-miR-18a-5p; hsa-miR-92a-3p; hsa-miR-331-3p
Gene Ontology (miRWalk)	GO0045070 positive regulation of viral genome replication	enriched	0.0440397	13	hsa-miR-155-5p; hsa-miR-324-3p; hsa-miR-1-3p; hsa-miR-26b-5p; hsa-miR-320a; hsa-miR-146a-5p; hsa-miR-16-5p; hsa-miR-181a-5p; hsa-miR-93-5p; hsa-miR-21-5p; hsa-miR-615-3p; hsa-miR-1226-3p; hsa-miR-331-3p

Gene Ontology (miRWalk)	GO0048268 clathrin coat assembly	enriched	0.0440397	13	hsa-miR-155-5p; hsa-miR-1-3p; hsa-miR-26b-5p; hsa-miR-106b-5p; hsa-let-7i-5p; hsa-miR-16-5p; hsa-miR-181d-5p; hsa-miR-93-5p; hsa-miR-124-3p; hsa-miR-21-5p; hsa-miR-19b-3p; hsa-miR-92a-3p; hsa-miR-615-3p
Gene Ontology (miRWalk)	GO0050327 testosterone 17 beta dehydrogenase nadposi activity	enriched	0.0440397	5	hsa-miR-155-5p; hsa-miR-1-3p; hsa-miR-26b-5p; hsa-miR-16-5p; hsa-miR-124-3p
Gene Ontology (miRWalk)	GO0051575 5 deoxyribose 5 phosphate lyase activity	enriched	0.0440397	18	hsa-let-7c-5p; hsa-let-7a-5p; hsa-miR-324-3p; hsa-let-7f-5p; hsa-miR-877-5p; hsa-miR-26b-5p; hsa-let-7g-5p; hsa-miR-760; hsa-miR-16-5p; hsa-miR-671-5p; hsa-miR-181b-5p; hsa-miR-124-3p; hsa-miR-26a-5p; hsa-miR-766-3p; hsa-miR-615-3p; hsa-miR-505-3p; hsa-miR-331-3p; hsa-miR-30c-5p
Gene Ontology (miRWalk)	GO0060137 maternal process involved in parturition	enriched	0.0440397	5	hsa-miR-155-5p; hsa-miR-1-3p; hsa-miR-26b-5p; hsa-miR-24-3p; hsa-miR-124-3p
Gene Ontology (miRWalk)	GO0060766 negative regulation of androgen receptor signaling pathway	enriched	0.0440397	13	hsa-let-7c-5p; hsa-miR-155-5p; hsa-miR-26b-5p; hsa-miR-16-5p; hsa-miR-24-3p; hsa-miR-671-5p; hsa-miR-181a-5p; hsa-miR-181b-5p; hsa-miR-124-3p; hsa-miR-21-5p; hsa-miR-92a-3p; hsa-miR-615-3p; hsa-miR-331-3p
Pathways (miRWalk)	P00037 Ionotropic glutamate receptor pathway	enriched	0.0441407	11	hsa-miR-155-5p; hsa-let-7a-5p; hsa-miR-1-3p; hsa-miR-26b-5p; hsa-miR-103a-3p; hsa-miR-16-5p; hsa-miR-181b-5p; hsa-miR-124-3p; hsa-miR-19b-3p; hsa-miR-505-3p; hsa-miR-331-3p
Pathways (miRWalk)	hsa00740 Riboflavin metabolism	enriched	0.0441407	11	hsa-miR-1-3p; hsa-miR-877-5p; hsa-miR-320a; hsa-miR-103a-3p; hsa-miR-106b-5p; hsa-miR-16-5p; hsa-miR-181a-5p; hsa-miR-124-3p; hsa-miR-25-3p; hsa-miR-15b-5p; hsa-miR-197-3p
Pathways (miRWalk)	WP183 Proteasome Degradation	enriched	0.0441536	49	hsa-miR-29c-3p; hsa-let-7c-5p; hsa-miR-155-5p; hsa-let-7a-5p; hsa-miR-324-3p; hsa-miR-30d-5p; hsa-miR-1-3p; hsa-miR-877-5p; hsa-miR-26b-5p; hsa-miR-320a; hsa-miR-103a-3p; hsa-miR-106b-5p; hsa-miR-760; hsa-miR-1287-5p; hsa-miR-142-3p; hsa-miR-107; hsa-miR-16-5p; hsa-miR-20a-5p; hsa-miR-24-3p; hsa-miR-92b-3p; hsa-miR-29b-3p; hsa-miR-181a-5p; hsa-miR-93-5p; hsa-miR-181b-5p; hsa-miR-124-3p; hsa-miR-26a-5p; hsa-miR-1229-3p; hsa-miR-188-5p; hsa-miR-15a-5p; hsa-miR-148a-3p; hsa-miR-940; hsa-miR-19b-3p; hsa-miR-25-3p; hsa-miR-18a-5p; hsa-miR-10a-5p; hsa-miR-15b-5p; hsa-miR-148b-3p; hsa-miR-149-5p; hsa-miR-23a-3p; hsa-miR-92a-3p; hsa-miR-615-3p; hsa-miR-185-3p; hsa-miR-1226-3p; hsa-miR-331-3p; hsa-miR-1236-3p; hsa-miR-197-3p; hsa-miR-346; hsa-miR-30c-5p; hsa-miR-328-3p
Gene Ontology (miRWalk)	GO0051091 positive regulation of sequence specific dna binding transcription factor activity	enriched	0.0442145	50	hsa-miR-155-5p; hsa-let-7a-5p; hsa-miR-324-3p; hsa-miR-1-3p; hsa-miR-26b-5p; hsa-miR-320a; hsa-miR-103a-3p; hsa-miR-106b-5p; hsa-miR-18b-5p; hsa-miR-760; hsa-miR-451a; hsa-miR-146a-5p; hsa-miR-425-3p; hsa-miR-107; hsa-miR-16-5p; hsa-miR-20a-5p; hsa-miR-24-3p; hsa-miR-92b-3p; hsa-miR-29b-3p; hsa-miR-365a-3p; hsa-miR-181a-5p; hsa-miR-181d-5p; hsa-miR-93-5p; hsa-miR-181b-5p; hsa-miR-124-3p; hsa-miR-21-5p; hsa-miR-29a-3p; hsa-miR-26a-5p; hsa-miR-19a-3p; hsa-miR-188-5p; hsa-miR-15a-5p; hsa-miR-20b-5p; hsa-miR-940; hsa-miR-19b-3p; hsa-miR-494-3p; hsa-miR-25-3p; hsa-miR-17-5p; hsa-miR-18a-5p; hsa-miR-15b-5p; hsa-miR-149-5p; hsa-miR-23a-3p; hsa-miR-92a-3p; hsa-miR-615-3p; hsa-miR-505-3p; hsa-miR-130b-5p; hsa-miR-204-5p; hsa-miR-331-3p; hsa-miR-454-5p; hsa-miR-197-3p; hsa-miR-30c-5p

Pathways (miRWalk)	hsa04210 Apoptosis	enriched	0.0444031	55	hsa-miR-29c-3p; hsa-let-7c-5p; hsa-miR-155-5p; hsa-let-7a-5p; hsa-miR-324-3p; hsa-miR-30d-5p; hsa-miR-1-3p; hsa-miR-26b-5p; hsa-miR-320a; hsa-let-7g-5p; hsa-miR-103a-3p; hsa-miR-106b-5p; hsa-miR-451a; hsa-miR-184; hsa-miR-146a-5p; hsa-miR-16-5p; hsa-miR-20a-5p; hsa-miR-24-3p; hsa-miR-29b-3p; hsa-miR-365a-3p; hsa-miR-181a-5p; hsa-miR-181d-5p; hsa-miR-93-5p; hsa-miR-181b-5p; hsa-miR-124-3p; hsa-miR-21-5p; hsa-miR-223-3p; hsa-miR-29a-3p; hsa-miR-26a-5p; hsa-miR-15a-5p; hsa-miR-148a-3p; hsa-miR-940; hsa-miR-320c; hsa-miR-19b-3p; hsa-miR-1228-3p; hsa-miR-25-3p; hsa-miR-17-5p; hsa-miR-18a-5p; hsa-miR-630; hsa-miR-15b-5p; hsa-miR-148b-3p; hsa-miR-149-5p; hsa-miR-504-5p; hsa-miR-92a-3p; hsa-miR-1260b; hsa-miR-204-5p; hsa-miR-1226-3p; hsa-miR-331-3p; hsa-miR-197-3p; hsa-miR-346; hsa-miR-30c-5p; hsa-miR-605-5p; hsa-miR-296-5p; hsa-miR-328-3p; hsa-miR-1296-5p
Gene Ontology (miRWalk)	GO0000014 single stranded dna specific endodeoxyribonuclease activity	enriched	0.0447474	2	hsa-miR-324-3p; hsa-miR-1-3p
Gene Ontology (miRWalk)	GO0005786 signal recognition particle endoplasmic reticulum targeting	enriched	0.0447474	6	hsa-miR-26b-5p; hsa-miR-320a; hsa-miR-16-5p; hsa-miR-92b-3p; hsa-miR-365a-3p; hsa-miR-1229-3p
Gene Ontology (miRWalk)	GO0006474 n terminal protein amino acid acetylation	enriched	0.0447474	6	hsa-miR-155-5p; hsa-miR-26b-5p; hsa-miR-103a-3p; hsa-miR-16-5p; hsa-miR-124-3p; hsa-miR-1229-3p
Gene Ontology (miRWalk)	GO0006548 histidine catabolic process	enriched	0.0447474	2	hsa-miR-155-5p; hsa-miR-1-3p
Gene Ontology (miRWalk)	GO0015057 thrombin receptor activity	enriched	0.0447474	2	hsa-let-7a-5p; hsa-miR-1-3p
Gene Ontology (miRWalk)	GO0019556 histidine catabolic process to glutamate and formamide	enriched	0.0447474	2	hsa-miR-155-5p; hsa-miR-1-3p
Gene Ontology (miRWalk)	GO0019557 histidine catabolic process to glutamate and formate	enriched	0.0447474	2	hsa-miR-155-5p; hsa-miR-1-3p
Gene Ontology (miRWalk)	GO0032825 positive regulation of natural killer cell differentiation	enriched	0.0447474	6	hsa-miR-155-5p; hsa-miR-324-3p; hsa-miR-1-3p; hsa-miR-199b-5p; hsa-miR-124-3p; hsa-miR-1229-3p
Gene Ontology (miRWalk)	GO0032873 negative regulation of stress activated mapk cascade	enriched	0.0447474	19	hsa-let-7c-5p; hsa-let-7a-5p; hsa-let-7f-5p; hsa-miR-26b-5p; hsa-miR-320a; hsa-let-7g-5p; hsa-miR-451a; hsa-miR-20a-5p; hsa-miR-24-3p; hsa-miR-21-5p; hsa-miR-223-3p; hsa-miR-26a-5p; hsa-miR-134-5p; hsa-miR-15a-5p; hsa-miR-449c-5p; hsa-miR-17-5p; hsa-miR-149-5p; hsa-miR-92a-3p; hsa-miR-34b-3p
Gene Ontology (miRWalk)	GO0045806 negative regulation of endocytosis	enriched	0.0447474	6	hsa-miR-155-5p; hsa-miR-1-3p; hsa-miR-16-5p; hsa-miR-24-3p; hsa-miR-124-3p; hsa-miR-1229-3p
Gene Ontology (miRWalk)	GO0060501 positive regulation of epithelial cell proliferation involved in lung morphogenesis	enriched	0.0447474	6	hsa-miR-155-5p; hsa-miR-1-3p; hsa-miR-320a; hsa-miR-20a-5p; hsa-miR-181a-5p; hsa-miR-1229-3p

Gene Ontology (miRWalk)	GO2000778 positive regulation of interleukin 6 secretion	enriched	0.0447474	2	hsa-let-7a-5p; hsa-miR-1-3p
Pathways (miRWalk)	hsa00564 Glycerophospholipid metabolism	enriched	0.0447711	26	hsa-miR-155-5p; hsa-miR-1-3p; hsa-miR-877-5p; hsa-miR-26b-5p; hsa-miR-320a; hsa-miR-103a-3p; hsa-miR-106b-5p; hsa-miR-142-3p; hsa-miR-16-5p; hsa-miR-24-3p; hsa-miR-92b-3p; hsa-miR-181a-5p; hsa-miR-124-3p; hsa-miR-21-5p; hsa-miR-26a-5p; hsa-miR-1229-3p; hsa-miR-15a-5p; hsa-miR-449c-5p; hsa-miR-10a-5p; hsa-miR-15b-5p; hsa-miR-149-5p; hsa-miR-615-3p; hsa-miR-1260b; hsa-miR-30b-5p; hsa-miR-1226-3p; hsa-miR-331-3p
Pathways (miRWalk)	P00033 Insulin IGF pathway protein kinase B signaling cascade	enriched	0.0458228	47	hsa-let-7c-5p; hsa-miR-155-5p; hsa-let-7a-5p; hsa-miR-877-5p; hsa-miR-26b-5p; hsa-miR-320a; hsa-miR-103a-3p; hsa-miR-106b-5p; hsa-miR-18b-5p; hsa-let-7i-5p; hsa-miR-451a; hsa-miR-184; hsa-miR-107; hsa-miR-16-5p; hsa-miR-20a-5p; hsa-miR-24-3p; hsa-miR-29b-3p; hsa-miR-671-5p; hsa-miR-93-5p; hsa-miR-181b-5p; hsa-miR-124-3p; hsa-miR-21-5p; hsa-miR-223-3p; hsa-miR-29a-3p; hsa-miR-26a-5p; hsa-miR-19a-3p; hsa-miR-148a-3p; hsa-miR-19b-3p; hsa-miR-494-3p; hsa-miR-25-3p; hsa-miR-17-5p; hsa-miR-18a-5p; hsa-miR-10a-5p; hsa-miR-15b-5p; hsa-miR-148b-3p; hsa-miR-149-5p; hsa-miR-23a-3p; hsa-miR-504-5p; hsa-miR-92a-3p; hsa-miR-615-3p; hsa-miR-1226-3p; hsa-miR-331-3p; hsa-miR-197-3p; hsa-miR-346; hsa-miR-30c-5p; hsa-miR-605-5p; hsa-miR-1296-5p
Pathways (miRWalk)	WP47 Hedgehog Signaling Pathway	enriched	0.0458228	13	hsa-miR-155-5p; hsa-miR-324-3p; hsa-miR-1-3p; hsa-miR-877-5p; hsa-miR-26b-5p; hsa-miR-142-3p; hsa-miR-92b-3p; hsa-miR-93-5p; hsa-miR-124-3p; hsa-miR-494-3p; hsa-miR-148b-3p; hsa-miR-92a-3p; hsa-miR-331-3p
Pathways (miRWalk)	hsa00604 Glycosphingolipid biosynthesis ganglio series	enriched	0.0458228	8	hsa-miR-155-5p; hsa-miR-324-3p; hsa-let-7f-5p; hsa-miR-26b-5p; hsa-miR-760; hsa-miR-148b-3p; hsa-miR-615-3p; hsa-miR-204-5p
Gene Ontology (miRWalk)	GO0006605 protein targeting	enriched	0.0461022	20	hsa-let-7c-5p; hsa-miR-155-5p; hsa-miR-324-3p; hsa-let-7f-5p; hsa-miR-1-3p; hsa-miR-26b-5p; hsa-miR-320a; hsa-miR-16-5p; hsa-miR-24-3p; hsa-miR-92b-3p; hsa-miR-181a-5p; hsa-miR-93-5p; hsa-miR-124-3p; hsa-miR-26a-5p; hsa-miR-25-3p; hsa-miR-149-5p; hsa-miR-92a-3p; hsa-miR-615-3p; hsa-miR-1910-5p; hsa-miR-328-3p
Gene Ontology (miRWalk)	GO0035267 nua4 histone acetyltransferase complex	enriched	0.0461022	30	hsa-miR-29c-3p; hsa-let-7c-5p; hsa-let-7a-5p; hsa-miR-324-3p; hsa-miR-1-3p; hsa-miR-26b-5p; hsa-miR-320a; hsa-miR-103a-3p; hsa-miR-106b-5p; hsa-let-7i-5p; hsa-miR-760; hsa-miR-1295a; hsa-miR-142-3p; hsa-miR-16-5p; hsa-miR-24-3p; hsa-miR-92b-3p; hsa-miR-181b-5p; hsa-miR-1229-3p; hsa-miR-19b-3p; hsa-miR-25-3p; hsa-miR-766-3p; hsa-miR-17-5p; hsa-miR-18a-5p; hsa-miR-10a-5p; hsa-miR-148b-3p; hsa-miR-23a-3p; hsa-miR-92a-3p; hsa-miR-615-3p; hsa-miR-331-3p; hsa-miR-197-3p
Gene Ontology (miRWalk)	GO0060325 face morphogenesis	enriched	0.0461022	20	hsa-miR-29c-3p; hsa-miR-155-5p; hsa-let-7a-5p; hsa-miR-1-3p; hsa-miR-26b-5p; hsa-miR-16-5p; hsa-miR-24-3p; hsa-miR-92b-3p; hsa-miR-29b-3p; hsa-miR-93-5p; hsa-miR-124-3p; hsa-miR-21-5p; hsa-miR-29a-3p; hsa-miR-26a-5p; hsa-miR-940; hsa-miR-10a-5p; hsa-miR-149-5p; hsa-miR-92a-3p; hsa-miR-30b-5p; hsa-miR-1226-3p
Gene Ontology (miRWalk)	GO0070742 c2h2 zinc finger domain binding	enriched	0.0461022	20	hsa-let-7c-5p; hsa-let-7a-5p; hsa-miR-324-3p; hsa-let-7f-5p; hsa-miR-877-5p; hsa-miR-26b-5p; hsa-miR-320a; hsa-let-7g-5p; hsa-miR-760; hsa-miR-16-5p; hsa-miR-92b-3p; hsa-miR-671-5p; hsa-miR-181b-5p; hsa-miR-26a-5p; hsa-miR-15a-5p; hsa-miR-25-3p; hsa-miR-504-5p; hsa-miR-92a-3p; hsa-miR-615-3p; hsa-miR-331-3p

Gene Ontology (miRWalk)	GO0001503 ossification	enriched	0.0461224	47	hsa-miR-29c-3p; hsa-miR-155-5p; hsa-let-7a-5p; hsa-let-7f-5p; hsa-miR-30d-5p; hsa-miR-1-3p; hsa-miR-877-5p; hsa-miR-26b-5p; hsa-miR-320a; hsa-miR-103a-3p; hsa-miR-451a; hsa-miR-146a-5p; hsa-miR-142-3p; hsa-miR-16-5p; hsa-miR-20a-5p; hsa-miR-24-3p; hsa-miR-29b-3p; hsa-miR-365a-3p; hsa-miR-181a-5p; hsa-miR-181d-5p; hsa-miR-93-5p; hsa-miR-181b-5p; hsa-miR-124-3p; hsa-miR-21-5p; hsa-miR-223-3p; hsa-miR-29a-3p; hsa-miR-26a-5p; hsa-miR-15a-5p; hsa-miR-148a-3p; hsa-miR-940; hsa-miR-25-3p; hsa-miR-17-5p; hsa-miR-18a-5p; hsa-miR-630; hsa-miR-10a-5p; hsa-miR-15b-5p; hsa-miR-148b-3p; hsa-miR-149-5p; hsa-miR-92a-3p; hsa-miR-505-3p; hsa-miR-1260b; hsa-miR-30b-5p; hsa-miR-204-5p; hsa-miR-454-5p; hsa-miR-1236-3p; hsa-miR-30c-5p; hsa-miR-296-5p
Gene Ontology (miRWalk)	GO0001836 release of cytochrome c from mitochondria	enriched	0.0461224	47	hsa-miR-29c-3p; hsa-let-7c-5p; hsa-miR-155-5p; hsa-let-7a-5p; hsa-let-7f-5p; hsa-miR-193a-5p; hsa-miR-30d-5p; hsa-miR-1-3p; hsa-miR-26b-5p; hsa-miR-320a; hsa-let-7g-5p; hsa-miR-103a-3p; hsa-miR-106b-5p; hsa-miR-451a; hsa-miR-16-5p; hsa-miR-20a-5p; hsa-miR-24-3p; hsa-miR-92b-3p; hsa-miR-29b-3p; hsa-miR-365a-3p; hsa-miR-181a-5p; hsa-miR-181d-5p; hsa-miR-93-5p; hsa-miR-181b-5p; hsa-miR-21-5p; hsa-miR-29a-3p; hsa-miR-26a-5p; hsa-miR-15a-5p; hsa-miR-449c-5p; hsa-miR-148a-3p; hsa-miR-25-3p; hsa-miR-17-5p; hsa-miR-18a-5p; hsa-miR-630; hsa-miR-15b-5p; hsa-miR-148b-3p; hsa-miR-149-5p; hsa-miR-504-5p; hsa-miR-92a-3p; hsa-miR-615-3p; hsa-miR-505-3p; hsa-miR-483-3p; hsa-miR-204-5p; hsa-miR-331-3p; hsa-miR-34b-3p; hsa-miR-605-5p; hsa-miR-296-5p
Gene Ontology (miRWalk)	GO0019900 kinase binding	enriched	0.0462123	32	hsa-miR-155-5p; hsa-miR-324-3p; hsa-miR-1-3p; hsa-miR-877-5p; hsa-miR-26b-5p; hsa-miR-320a; hsa-miR-103a-3p; hsa-miR-106b-5p; hsa-miR-451a; hsa-miR-146a-5p; hsa-miR-142-3p; hsa-miR-16-5p; hsa-miR-20a-5p; hsa-miR-24-3p; hsa-miR-181a-5p; hsa-miR-93-5p; hsa-miR-124-3p; hsa-miR-21-5p; hsa-miR-29a-3p; hsa-miR-26a-5p; hsa-miR-15a-5p; hsa-miR-1224-5p; hsa-miR-17-5p; hsa-miR-15b-5p; hsa-miR-149-5p; hsa-miR-504-5p; hsa-miR-92a-3p; hsa-miR-615-3p; hsa-miR-204-5p; hsa-miR-1226-3p; hsa-miR-331-3p; hsa-miR-30c-5p
Gene Ontology (miRWalk)	GO0045786 negative regulation of cell cycle	enriched	0.0462123	32	hsa-miR-29c-3p; hsa-miR-155-5p; hsa-let-7a-5p; hsa-miR-1-3p; hsa-miR-877-5p; hsa-miR-26b-5p; hsa-miR-320a; hsa-miR-103a-3p; hsa-miR-107; hsa-miR-16-5p; hsa-miR-24-3p; hsa-miR-29b-3p; hsa-miR-671-5p; hsa-miR-93-5p; hsa-miR-181b-5p; hsa-miR-124-3p; hsa-miR-21-5p; hsa-miR-223-3p; hsa-miR-29a-3p; hsa-miR-26a-5p; hsa-miR-15a-5p; hsa-miR-766-3p; hsa-miR-17-5p; hsa-miR-10a-5p; hsa-miR-148b-3p; hsa-miR-149-5p; hsa-miR-92a-3p; hsa-miR-615-3p; hsa-miR-505-3p; hsa-miR-197-3p; hsa-miR-34b-3p; hsa-miR-30c-5p
Pathways (miRWalk)	hsa04260 Cardiac muscle contraction	enriched	0.046393	37	hsa-let-7c-5p; hsa-miR-155-5p; hsa-let-7a-5p; hsa-miR-324-3p; hsa-let-7f-5p; hsa-miR-30d-5p; hsa-miR-1-3p; hsa-miR-26b-5p; hsa-miR-320a; hsa-miR-103a-3p; hsa-miR-106b-5p; hsa-miR-18b-5p; hsa-miR-760; hsa-miR-142-3p; hsa-miR-16-5p; hsa-miR-20a-5p; hsa-miR-671-5p; hsa-miR-93-5p; hsa-miR-124-3p; hsa-miR-21-5p; hsa-miR-26a-5p; hsa-miR-148a-3p; hsa-miR-940; hsa-miR-25-3p; hsa-miR-17-5p; hsa-miR-18a-5p; hsa-miR-10a-5p; hsa-miR-15b-5p; hsa-miR-148b-3p; hsa-miR-92a-3p; hsa-miR-615-3p; hsa-miR-130b-5p; hsa-miR-1226-3p; hsa-miR-331-3p; hsa-miR-197-3p; hsa-miR-30c-5p; hsa-miR-1296-5p
Pathways (miRWalk)	hsa00410 beta Alanine metabolism	enriched	0.0464827	16	hsa-miR-155-5p; hsa-let-7a-5p; hsa-miR-324-3p; hsa-let-7f-5p; hsa-miR-1-3p; hsa-miR-26b-5p; hsa-miR-320a; hsa-miR-16-5p; hsa-miR-124-3p; hsa-miR-15b-5p; hsa-miR-149-5p; hsa-miR-92a-3p; hsa-miR-615-3p; hsa-miR-130b-5p; hsa-miR-1226-3p; hsa-miR-328-3p

Gene Ontology (miRWalk)	GO0008209 androgen metabolic process	enriched	0.0465755	11	hsa-miR-155-5p; hsa-let-7f-5p; hsa-miR-1-3p; hsa-miR-26b-5p; hsa-miR-16-5p; hsa-miR-93-5p; hsa-miR-124-3p; hsa-miR-21-5p; hsa-miR-26a-5p; hsa-miR-19b-3p; hsa-miR-1226-3p
Pathways (miRWalk)	WP98 Prostaglandin Synthesis and Regulation	enriched	0.0466162	24	hsa-let-7c-5p; hsa-miR-155-5p; hsa-let-7a-5p; hsa-miR-1-3p; hsa-miR-26b-5p; hsa-miR-320a; hsa-miR-550a-5p; hsa-miR-760; hsa-miR-16-5p; hsa-miR-92b-3p; hsa-miR-181a-5p; hsa-miR-124-3p; hsa-miR-21-5p; hsa-miR-26a-5p; hsa-miR-99b-3p; hsa-miR-449c-5p; hsa-miR-320c; hsa-miR-766-3p; hsa-miR-17-5p; hsa-miR-10a-5p; hsa-miR-148b-3p; hsa-miR-149-5p; hsa-miR-92a-3p; hsa-miR-615-3p
Pathways (miRWalk)	hsa00910 Nitrogen metabolism	enriched	0.0466162	24	hsa-miR-155-5p; hsa-let-7a-5p; hsa-let-7f-5p; hsa-miR-1-3p; hsa-miR-877-5p; hsa-miR-26b-5p; hsa-miR-320a; hsa-miR-103a-3p; hsa-miR-142-3p; hsa-miR-16-5p; hsa-miR-24-3p; hsa-miR-124-3p; hsa-miR-29a-3p; hsa-miR-26a-5p; hsa-miR-99b-3p; hsa-miR-19b-3p; hsa-miR-17-5p; hsa-miR-18a-5p; hsa-miR-149-5p; hsa-miR-23a-3p; hsa-miR-92a-3p; hsa-miR-937-3p; hsa-miR-615-3p; hsa-miR-1226-3p
Gene Ontology (miRWalk)	GO0001190 rna polymerase ii transcription factor binding transcription factor activity involved in positive regulation of transcription	enriched	0.0467008	22	hsa-miR-29c-3p; hsa-miR-155-5p; hsa-miR-26b-5p; hsa-miR-103a-3p; hsa-miR-106b-5p; hsa-miR-142-3p; hsa-miR-107; hsa-miR-16-5p; hsa-miR-24-3p; hsa-miR-181a-5p; hsa-miR-93-5p; hsa-miR-124-3p; hsa-miR-21-5p; hsa-miR-223-3p; hsa-miR-26a-5p; hsa-miR-15a-5p; hsa-miR-494-3p; hsa-miR-25-3p; hsa-miR-149-5p; hsa-miR-615-3p; hsa-miR-30b-5p; hsa-miR-34b-3p
Gene Ontology (miRWalk)	GO0001656 metanephros development	enriched	0.0467008	35	hsa-miR-29c-3p; hsa-miR-155-5p; hsa-let-7a-5p; hsa-miR-324-3p; hsa-miR-30d-5p; hsa-miR-1-3p; hsa-miR-877-5p; hsa-miR-26b-5p; hsa-miR-103a-3p; hsa-miR-451a; hsa-miR-16-5p; hsa-miR-20a-5p; hsa-miR-29b-3p; hsa-miR-365a-3p; hsa-miR-181a-5p; hsa-miR-181d-5p; hsa-miR-93-5p; hsa-miR-181b-5p; hsa-miR-124-3p; hsa-miR-21-5p; hsa-miR-29a-3p; hsa-miR-15a-5p; hsa-miR-148a-3p; hsa-miR-940; hsa-miR-25-3p; hsa-miR-17-5p; hsa-miR-630; hsa-miR-15b-5p; hsa-miR-148b-3p; hsa-miR-92a-3p; hsa-miR-615-3p; hsa-miR-30b-5p; hsa-miR-204-5p; hsa-miR-1236-3p; hsa-miR-296-5p
Gene Ontology (miRWalk)	GO0001671 atpase activator activity	enriched	0.0467008	8	hsa-miR-155-5p; hsa-miR-1-3p; hsa-miR-26b-5p; hsa-miR-106b-5p; hsa-miR-16-5p; hsa-miR-124-3p; hsa-miR-21-5p; hsa-miR-615-3p
Gene Ontology (miRWalk)	GO0005109 frizzled binding	enriched	0.0467008	22	hsa-miR-29c-3p; hsa-miR-155-5p; hsa-miR-324-3p; hsa-let-7f-5p; hsa-miR-26b-5p; hsa-miR-320a; hsa-miR-103a-3p; hsa-miR-106b-5p; hsa-miR-760; hsa-miR-16-5p; hsa-miR-181a-5p; hsa-miR-93-5p; hsa-miR-124-3p; hsa-miR-21-5p; hsa-miR-26a-5p; hsa-miR-20b-5p; hsa-miR-940; hsa-miR-10a-5p; hsa-miR-15b-5p; hsa-miR-148b-3p; hsa-miR-92a-3p; hsa-miR-615-3p
Gene Ontology (miRWalk)	GO0006491 n glycan processing	enriched	0.0467008	8	hsa-miR-155-5p; hsa-miR-26b-5p; hsa-miR-320a; hsa-miR-142-3p; hsa-miR-16-5p; hsa-miR-124-3p; hsa-miR-21-5p; hsa-miR-149-5p
Gene Ontology (miRWalk)	GO0015030 cajal body	enriched	0.0467008	29	hsa-miR-29c-3p; hsa-miR-155-5p; hsa-let-7a-5p; hsa-miR-324-3p; hsa-let-7f-5p; hsa-miR-1-3p; hsa-miR-877-5p; hsa-miR-26b-5p; hsa-miR-320a; hsa-miR-103a-3p; hsa-miR-146a-5p; hsa-miR-16-5p; hsa-miR-20a-5p; hsa-miR-365a-3p; hsa-miR-93-5p; hsa-miR-181b-5p; hsa-miR-124-3p; hsa-miR-21-5p; hsa-miR-1229-3p; hsa-miR-25-3p; hsa-miR-17-5p; hsa-miR-10a-5p; hsa-miR-15b-5p; hsa-miR-504-5p; hsa-miR-92a-3p; hsa-miR-615-3p; hsa-miR-331-3p; hsa-miR-1236-3p; hsa-miR-885-5p
Gene Ontology (miRWalk)	GO0017111 nucleoside triphosphatase activity	enriched	0.0467008	22	hsa-miR-155-5p; hsa-let-7a-5p; hsa-miR-1-3p; hsa-miR-26b-5p; hsa-miR-320a; hsa-miR-106b-5p; hsa-miR-142-3p; hsa-miR-16-5p; hsa-miR-24-3p; hsa-miR-92b-3p; hsa-miR-93-5p; hsa-miR-124-3p; hsa-miR-21-5p; hsa-miR-29a-3p; hsa-miR-26a-5p; hsa-miR-25-3p; hsa-miR-149-5p; hsa-miR-23a-3p; hsa-miR-92a-3p; hsa-miR-615-3p; hsa-miR-331-3p; hsa-miR-346

Gene Ontology (miRWalk)	GO0032609 interferon gamma production	enriched	0.0467008	8	hsa-miR-1-3p; hsa-miR-26b-5p; hsa-miR-106b-5p; hsa-let-7i-5p; hsa-miR-146a-5p; hsa-miR-93-5p; hsa-miR-21-5p; hsa-miR-148b-3p
Gene Ontology (miRWalk)	GO0034101 erythrocyte homeostasis	enriched	0.0467008	15	hsa-let-7c-5p; hsa-miR-155-5p; hsa-let-7a-5p; hsa-miR-30d-5p; hsa-miR-1-3p; hsa-miR-26b-5p; hsa-miR-320a; hsa-miR-199b-5p; hsa-miR-16-5p; hsa-miR-124-3p; hsa-miR-26a-5p; hsa-miR-148a-3p; hsa-miR-148b-3p; hsa-miR-92a-3p; hsa-miR-615-3p
Gene Ontology (miRWalk)	GO0035326 enhancer binding	enriched	0.0467008	8	hsa-let-7a-5p; hsa-let-7f-5p; hsa-miR-1-3p; hsa-miR-16-5p; hsa-miR-29b-3p; hsa-miR-124-3p; hsa-miR-21-5p; hsa-miR-149-5p
Gene Ontology (miRWalk)	GO0045120 pronucleus	enriched	0.0467008	22	hsa-miR-155-5p; hsa-let-7a-5p; hsa-miR-30d-5p; hsa-miR-1-3p; hsa-miR-877-5p; hsa-miR-26b-5p; hsa-miR-320a; hsa-miR-106b-5p; hsa-miR-16-5p; hsa-miR-92b-3p; hsa-miR-29b-3p; hsa-miR-93-5p; hsa-miR-181b-5p; hsa-miR-124-3p; hsa-miR-26a-5p; hsa-miR-3620-3p; hsa-miR-25-3p; hsa-miR-615-3p; hsa-miR-1226-3p; hsa-miR-331-3p; hsa-miR-30c-5p; hsa-miR-328-3p
Gene Ontology (miRWalk)	GO0046902 regulation of mitochondrial membrane permeability	enriched	0.0467008	35	hsa-miR-29c-3p; hsa-let-7c-5p; hsa-let-7a-5p; hsa-miR-30d-5p; hsa-miR-1-3p; hsa-miR-26b-5p; hsa-miR-320a; hsa-let-7g-5p; hsa-miR-103a-3p; hsa-miR-106b-5p; hsa-miR-451a; hsa-miR-16-5p; hsa-miR-20a-5p; hsa-miR-29b-3p; hsa-miR-365a-3p; hsa-miR-181a-5p; hsa-miR-181d-5p; hsa-miR-181b-5p; hsa-miR-124-3p; hsa-miR-21-5p; hsa-miR-29a-3p; hsa-miR-15a-5p; hsa-miR-148a-3p; hsa-miR-25-3p; hsa-miR-17-5p; hsa-miR-630; hsa-miR-15b-5p; hsa-miR-149-5p; hsa-miR-504-5p; hsa-miR-92a-3p; hsa-miR-615-3p; hsa-miR-204-5p; hsa-miR-331-3p; hsa-miR-605-5p; hsa-miR-296-5p
Gene Ontology (miRWalk)	GO0048863 stem cell differentiation	enriched	0.0467008	22	hsa-let-7c-5p; hsa-miR-155-5p; hsa-let-7a-5p; hsa-let-7f-5p; hsa-miR-877-5p; hsa-miR-26b-5p; hsa-miR-320a; hsa-let-7g-5p; hsa-miR-106b-5p; hsa-miR-760; hsa-miR-16-5p; hsa-miR-24-3p; hsa-miR-93-5p; hsa-miR-21-5p; hsa-miR-26a-5p; hsa-miR-940; hsa-miR-19b-3p; hsa-miR-17-5p; hsa-miR-92a-3p; hsa-miR-615-3p; hsa-miR-204-5p; hsa-miR-1236-3p
Gene Ontology (miRWalk)	GO0051593 response to folic acid	enriched	0.0467008	30	hsa-miR-29c-3p; hsa-let-7a-5p; hsa-miR-1-3p; hsa-miR-26b-5p; hsa-miR-320a; hsa-miR-103a-3p; hsa-miR-18b-5p; hsa-miR-451a; hsa-miR-16-5p; hsa-miR-20a-5p; hsa-miR-29b-3p; hsa-miR-365a-3p; hsa-miR-181a-5p; hsa-miR-181d-5p; hsa-miR-181b-5p; hsa-miR-124-3p; hsa-miR-21-5p; hsa-miR-29a-3p; hsa-miR-26a-5p; hsa-miR-15a-5p; hsa-miR-148a-3p; hsa-miR-940; hsa-miR-17-5p; hsa-miR-630; hsa-miR-15b-5p; hsa-miR-149-5p; hsa-miR-92a-3p; hsa-miR-615-3p; hsa-miR-204-5p; hsa-miR-296-5p
Gene Ontology (miRWalk)	GO0051721 protein phosphatase 2a binding	enriched	0.0467008	35	hsa-miR-29c-3p; hsa-miR-155-5p; hsa-let-7a-5p; hsa-miR-30d-5p; hsa-miR-1-3p; hsa-miR-320a; hsa-miR-103a-3p; hsa-miR-106b-5p; hsa-miR-451a; hsa-miR-16-5p; hsa-miR-20a-5p; hsa-miR-29b-3p; hsa-miR-365a-3p; hsa-miR-181a-5p; hsa-miR-181d-5p; hsa-miR-93-5p; hsa-miR-181b-5p; hsa-miR-124-3p; hsa-miR-21-5p; hsa-miR-223-3p; hsa-miR-29a-3p; hsa-miR-15a-5p; hsa-miR-148a-3p; hsa-miR-25-3p; hsa-miR-17-5p; hsa-miR-630; hsa-miR-15b-5p; hsa-miR-504-5p; hsa-miR-92a-3p; hsa-miR-130b-5p; hsa-miR-204-5p; hsa-miR-331-3p; hsa-miR-197-3p; hsa-miR-605-5p; hsa-miR-296-5p
Gene Ontology (miRWalk)	GO0009083 branched chain family amino acid catabolic process	enriched	0.046743	15	hsa-miR-29c-3p; hsa-miR-155-5p; hsa-let-7a-5p; hsa-miR-1-3p; hsa-miR-26b-5p; hsa-miR-320a; hsa-miR-103a-3p; hsa-miR-16-5p; hsa-miR-92b-3p; hsa-miR-124-3p; hsa-miR-21-5p; hsa-miR-10a-5p; hsa-miR-92a-3p; hsa-miR-615-3p; hsa-miR-1260b
Gene Ontology (miRWalk)	GO0016572 histone phosphorylation	enriched	0.046743	16	hsa-let-7c-5p; hsa-miR-155-5p; hsa-let-7a-5p; hsa-let-7f-5p; hsa-miR-26b-5p; hsa-miR-103a-3p; hsa-miR-16-5p; hsa-miR-20a-5p; hsa-miR-93-5p; hsa-miR-124-3p; hsa-miR-21-5p; hsa-miR-19a-3p; hsa-miR-148a-3p; hsa-miR-17-5p; hsa-miR-30b-5p; hsa-miR-885-5p

Gene Ontology (miRWalk)	GO0030122 ap 2 adaptor complex	enriched	0.046743	15	hsa-miR-155-5p; hsa-let-7a-5p; hsa-miR-324-3p; hsa-miR-1-3p; hsa-miR-103a-3p; hsa-miR-146a-5p; hsa-miR-16-5p; hsa-miR-92b-3p; hsa-miR-93-5p; hsa-miR-124-3p; hsa-miR-21-5p; hsa-miR-92a-3p; hsa-miR-615-3p; hsa-miR-30b-5p; hsa-miR-331-3p
Gene Ontology (miRWalk)	GO0040037 negative regulation of fibroblast growth factor receptor signaling pathway	enriched	0.046743	15	hsa-miR-29c-3p; hsa-miR-155-5p; hsa-let-7a-5p; hsa-miR-324-3p; hsa-miR-1-3p; hsa-miR-26b-5p; hsa-miR-16-5p; hsa-miR-20a-5p; hsa-miR-181a-5p; hsa-miR-124-3p; hsa-miR-21-5p; hsa-miR-148a-3p; hsa-miR-17-5p; hsa-miR-516a-3p; hsa-miR-92a-3p
Gene Ontology (miRWalk)	GO0045335 phagocytic vesicle	enriched	0.046743	16	hsa-miR-155-5p; hsa-miR-324-3p; hsa-miR-1-3p; hsa-miR-26b-5p; hsa-miR-320a; hsa-miR-106b-5p; hsa-miR-142-3p; hsa-miR-24-3p; hsa-miR-93-5p; hsa-miR-124-3p; hsa-miR-26a-5p; hsa-miR-19a-3p; hsa-miR-25-3p; hsa-miR-18a-5p; hsa-miR-92a-3p; hsa-miR-615-3p
Gene Ontology (miRWalk)	GO0045948 positive regulation of translational initiation	enriched	0.046743	15	hsa-miR-155-5p; hsa-miR-1-3p; hsa-miR-877-5p; hsa-miR-26b-5p; hsa-miR-103a-3p; hsa-miR-760; hsa-miR-16-5p; hsa-miR-92b-3p; hsa-miR-181a-5p; hsa-miR-124-3p; hsa-miR-21-5p; hsa-miR-148b-3p; hsa-miR-629-3p; hsa-miR-130b-5p; hsa-miR-1296-5p
Gene Ontology (miRWalk)	GO0005615 extracellular space	enriched	0.0468237	75	hsa-miR-29c-3p; hsa-let-7c-5p; hsa-miR-155-5p; hsa-let-7a-5p; hsa-miR-324-3p; hsa-let-7f-5p; hsa-miR-30d-5p; hsa-miR-1-3p; hsa-miR-877-5p; hsa-miR-26b-5p; hsa-miR-320a; hsa-let-7g-5p; hsa-miR-103a-3p; hsa-miR-199b-5p; hsa-miR-106b-5p; hsa-let-7i-5p; hsa-miR-760; hsa-miR-451a; hsa-miR-146a-5p; hsa-miR-142-3p; hsa-miR-107; hsa-miR-16-5p; hsa-miR-20a-5p; hsa-miR-24-3p; hsa-miR-92b-3p; hsa-miR-29b-3p; hsa-miR-671-5p; hsa-miR-365a-3p; hsa-miR-181a-5p; hsa-miR-181d-5p; hsa-miR-93-5p; hsa-miR-181b-5p; hsa-miR-124-3p; hsa-miR-21-5p; hsa-miR-223-3p; hsa-miR-29a-3p; hsa-miR-26a-5p; hsa-miR-99b-3p; hsa-miR-134-5p; hsa-miR-19a-3p; hsa-miR-1229-3p; hsa-miR-15a-5p; hsa-miR-20b-5p; hsa-miR-148a-3p; hsa-miR-19b-3p; hsa-miR-520g-3p; hsa-miR-25-3p; hsa-miR-17-5p; hsa-miR-18a-5p; hsa-miR-10a-5p; hsa-miR-15b-5p; hsa-miR-148b-3p; hsa-miR-149-5p; hsa-miR-663a; hsa-miR-23a-3p; hsa-miR-504-5p; hsa-miR-26b-3p; hsa-miR-516a-3p; hsa-miR-92a-3p; hsa-miR-299-5p; hsa-miR-615-3p; hsa-miR-505-3p; hsa-miR-1260b; hsa-miR-30b-5p; hsa-miR-204-5p; hsa-miR-1226-3p; hsa-miR-331-3p; hsa-miR-454-5p; hsa-miR-197-3p; hsa-miR-346; hsa-miR-34b-3p; hsa-miR-211-5p; hsa-miR-30c-5p; hsa-miR-328-3p; hsa-miR-1296-5p
Gene Ontology (miRWalk)	GO0006468 protein phosphorylation	enriched	0.0468237	75	hsa-miR-29c-3p; hsa-let-7c-5p; hsa-miR-155-5p; hsa-let-7a-5p; hsa-miR-584-5p; hsa-miR-324-3p; hsa-miR-30d-5p; hsa-miR-1-3p; hsa-miR-877-5p; hsa-miR-26b-5p; hsa-miR-320a; hsa-let-7g-5p; hsa-miR-103a-3p; hsa-miR-199b-5p; hsa-miR-106b-5p; hsa-miR-550a-5p; hsa-miR-760; hsa-miR-451a; hsa-miR-146a-5p; hsa-miR-548d-3p; hsa-miR-142-3p; hsa-miR-425-3p; hsa-miR-107; hsa-miR-16-5p; hsa-miR-20a-5p; hsa-miR-24-3p; hsa-miR-92b-3p; hsa-miR-29b-3p; hsa-miR-365a-3p; hsa-miR-181a-5p; hsa-miR-483-5p; hsa-miR-93-5p; hsa-miR-181b-5p; hsa-miR-124-3p; hsa-miR-21-5p; hsa-miR-223-3p; hsa-miR-29a-3p; hsa-miR-26a-5p; hsa-miR-99b-3p; hsa-miR-19a-3p; hsa-miR-1229-3p; hsa-miR-15a-5p; hsa-miR-1268a; hsa-miR-20b-5p; hsa-miR-26a-2-3p; hsa-miR-148a-3p; hsa-miR-940; hsa-miR-320c; hsa-miR-3620-3p; hsa-miR-19b-3p; hsa-miR-25-3p; hsa-miR-766-3p; hsa-miR-17-5p; hsa-miR-18a-5p; hsa-miR-10a-5p; hsa-miR-15b-5p; hsa-miR-148b-3p; hsa-miR-149-5p; hsa-miR-23a-3p; hsa-miR-92a-3p; hsa-miR-615-3p; hsa-miR-505-3p; hsa-miR-1260b; hsa-miR-30b-5p; hsa-miR-204-5p; hsa-miR-337-3p; hsa-miR-1226-3p; hsa-miR-331-3p; hsa-miR-197-3p; hsa-miR-346; hsa-miR-34b-3p; hsa-miR-30c-5p; hsa-miR-296-5p; hsa-miR-328-3p; hsa-miR-1296-5p

Gene Ontology (miRWalk)	GO0004674 protein serine threonine kinase activity	enriched	0.0470175	66	hsa-miR-29c-3p; hsa-let-7c-5p; hsa-miR-155-5p; hsa-let-7a-5p; hsa-miR-584-5p; hsa-miR-324-3p; hsa-let-7f-5p; hsa-miR-1-3p; hsa-miR-877-5p; hsa-miR-26b-5p; hsa-miR-320a; hsa-miR-103a-3p; hsa-miR-199b-5p; hsa-miR-106b-5p; hsa-miR-550a-5p; hsa-miR-760; hsa-miR-451a; hsa-miR-184; hsa-miR-146a-5p; hsa-miR-142-3p; hsa-miR-425-3p; hsa-miR-107; hsa-miR-16-5p; hsa-miR-20a-5p; hsa-miR-24-3p; hsa-miR-92b-3p; hsa-miR-29b-3p; hsa-miR-365a-3p; hsa-miR-181a-5p; hsa-miR-93-5p; hsa-miR-181b-5p; hsa-miR-124-3p; hsa-miR-21-5p; hsa-miR-29a-3p; hsa-miR-26a-5p; hsa-miR-19a-3p; hsa-miR-1229-3p; hsa-miR-15a-5p; hsa-miR-20b-5p; hsa-miR-148a-3p; hsa-miR-940; hsa-miR-320c; hsa-miR-19b-3p; hsa-miR-766-3p; hsa-miR-17-5p; hsa-miR-18a-5p; hsa-miR-10a-5p; hsa-miR-15b-5p; hsa-miR-148b-3p; hsa-miR-149-5p; hsa-miR-92a-3p; hsa-miR-615-3p; hsa-miR-505-3p; hsa-miR-1260b; hsa-miR-30b-5p; hsa-miR-130b-5p; hsa-miR-204-5p; hsa-miR-337-3p; hsa-miR-1226-3p; hsa-miR-331-3p; hsa-miR-197-3p; hsa-miR-346; hsa-miR-30c-5p; hsa-miR-296-5p; hsa-miR-328-3p; hsa-miR-1296-5p
Gene Ontology (miRWalk)	GO0004842 ubiquitin protein ligase activity	enriched	0.0470175	66	hsa-miR-29c-3p; hsa-let-7c-5p; hsa-miR-155-5p; hsa-let-7a-5p; hsa-miR-324-3p; hsa-let-7f-5p; hsa-miR-30d-5p; hsa-miR-1-3p; hsa-miR-877-5p; hsa-miR-26b-5p; hsa-miR-320a; hsa-let-7g-5p; hsa-miR-103a-3p; hsa-miR-1227-3p; hsa-miR-106b-5p; hsa-miR-18b-5p; hsa-miR-760; hsa-miR-451a; hsa-miR-146a-5p; hsa-miR-142-3p; hsa-miR-16-5p; hsa-miR-20a-5p; hsa-miR-24-3p; hsa-miR-92b-3p; hsa-miR-671-5p; hsa-miR-181a-5p; hsa-miR-181d-5p; hsa-miR-93-5p; hsa-miR-181b-5p; hsa-miR-124-3p; hsa-miR-4284; hsa-miR-21-5p; hsa-miR-29a-3p; hsa-miR-26a-5p; hsa-miR-99b-3p; hsa-miR-19a-3p; hsa-miR-1229-3p; hsa-miR-188-5p; hsa-miR-15a-5p; hsa-miR-20b-5p; hsa-miR-19b-3p; hsa-miR-25-3p; hsa-miR-17-5p; hsa-miR-18a-5p; hsa-miR-10a-5p; hsa-miR-15b-5p; hsa-miR-148b-3p; hsa-miR-149-5p; hsa-miR-504-5p; hsa-miR-26b-3p; hsa-miR-92a-3p; hsa-miR-615-3p; hsa-miR-521; hsa-miR-505-3p; hsa-miR-1260b; hsa-miR-30b-5p; hsa-miR-130b-5p; hsa-miR-204-5p; hsa-miR-1226-3p; hsa-miR-331-3p; hsa-miR-197-3p; hsa-miR-346; hsa-miR-30c-5p; hsa-miR-605-5p; hsa-miR-328-3p; hsa-miR-1296-5p
Gene Ontology (miRWalk)	GO0043407 negative regulation of map kinase activity	enriched	0.0470175	24	hsa-miR-29c-3p; hsa-let-7c-5p; hsa-miR-155-5p; hsa-miR-324-3p; hsa-miR-1-3p; hsa-miR-877-5p; hsa-miR-26b-5p; hsa-miR-103a-3p; hsa-miR-760; hsa-miR-184; hsa-miR-16-5p; hsa-miR-671-5p; hsa-miR-181a-5p; hsa-miR-124-3p; hsa-miR-21-5p; hsa-miR-26a-5p; hsa-miR-148a-3p; hsa-miR-1224-5p; hsa-miR-19b-3p; hsa-miR-92a-3p; hsa-miR-615-3p; hsa-miR-204-5p; hsa-miR-1914-5p; hsa-miR-328-3p
Gene Ontology (miRWalk)	GO0006730 one carbon metabolic process	enriched	0.0470251	21	hsa-miR-29c-3p; hsa-miR-155-5p; hsa-let-7a-5p; hsa-miR-324-3p; hsa-miR-30d-5p; hsa-miR-1-3p; hsa-miR-26b-5p; hsa-miR-103a-3p; hsa-miR-106b-5p; hsa-miR-142-3p; hsa-miR-16-5p; hsa-miR-124-3p; hsa-miR-26a-5p; hsa-miR-99b-3p; hsa-miR-1229-3p; hsa-miR-940; hsa-miR-19b-3p; hsa-miR-18a-5p; hsa-miR-149-5p; hsa-miR-92a-3p; hsa-miR-615-3p

Gene Ontology (miRWalk)	GO0030424 axon	enriched	0.0470251	58	hsa-miR-29c-3p; hsa-let-7c-5p; hsa-miR-155-5p; hsa-let-7a-5p; hsa-let-7f-5p; hsa-miR-30d-5p; hsa-miR-1-3p; hsa-miR-877-5p; hsa-miR-26b-5p; hsa-miR-320a; hsa-let-7g-5p; hsa-miR-103a-3p; hsa-miR-106b-5p; hsa-miR-760; hsa-miR-451a; hsa-miR-320d; hsa-miR-146a-5p; hsa-miR-1295a; hsa-miR-142-3p; hsa-miR-16-5p; hsa-miR-20a-5p; hsa-miR-24-3p; hsa-miR-92b-3p; hsa-miR-29b-3p; hsa-miR-181a-5p; hsa-miR-93-5p; hsa-miR-181b-5p; hsa-miR-124-3p; hsa-miR-21-5p; hsa-miR-29a-3p; hsa-miR-26a-5p; hsa-miR-99b-3p; hsa-miR-1229-3p; hsa-miR-15a-5p; hsa-miR-449c-5p; hsa-miR-148a-3p; hsa-miR-3620-3p; hsa-miR-25-3p; hsa-miR-17-5p; hsa-miR-18a-5p; hsa-miR-10a-5p; hsa-miR-15b-5p; hsa-miR-148b-3p; hsa-miR-3605-3p; hsa-miR-23a-3p; hsa-miR-504-5p; hsa-miR-92a-3p; hsa-miR-615-3p; hsa-miR-505-3p; hsa-miR-1260b; hsa-miR-30b-5p; hsa-miR-130b-5p; hsa-miR-1226-3p; hsa-miR-331-3p; hsa-miR-197-3p; hsa-miR-34b-3p; hsa-miR-30c-5p; hsa-miR-328-3p
Gene Ontology (miRWalk)	GO0045668 negative regulation of osteoblast differentiation	enriched	0.0470251	26	hsa-miR-29c-3p; hsa-miR-155-5p; hsa-let-7a-5p; hsa-miR-1-3p; hsa-miR-26b-5p; hsa-miR-103a-3p; hsa-miR-107; hsa-miR-16-5p; hsa-miR-24-3p; hsa-miR-92b-3p; hsa-miR-29b-3p; hsa-miR-181a-5p; hsa-miR-181b-5p; hsa-miR-124-3p; hsa-miR-21-5p; hsa-miR-29a-3p; hsa-miR-26a-5p; hsa-miR-25-3p; hsa-miR-17-5p; hsa-miR-18a-5p; hsa-miR-10a-5p; hsa-miR-149-5p; hsa-miR-92a-3p; hsa-miR-615-3p; hsa-miR-331-3p; hsa-miR-34b-3p
Gene Ontology (miRWalk)	GO0048839 inner ear development	enriched	0.0470251	21	hsa-miR-29c-3p; hsa-miR-155-5p; hsa-miR-324-3p; hsa-miR-193a-5p; hsa-miR-1-3p; hsa-miR-26b-5p; hsa-miR-760; hsa-miR-142-3p; hsa-miR-24-3p; hsa-miR-29b-3p; hsa-miR-181a-5p; hsa-miR-124-3p; hsa-miR-21-5p; hsa-miR-29a-3p; hsa-miR-1229-3p; hsa-miR-10a-5p; hsa-miR-615-3p; hsa-miR-1260b; hsa-miR-1226-3p; hsa-miR-34b-3p; hsa-miR-296-5p
Gene Ontology (miRWalk)	GO0016032 viral reproduction	enriched	0.0470253	82	hsa-miR-29c-3p; hsa-miR-22-5p; hsa-let-7c-5p; hsa-miR-155-5p; hsa-miR-193b-5p; hsa-let-7a-5p; hsa-miR-324-3p; hsa-let-7f-5p; hsa-miR-193a-5p; hsa-miR-30d-5p; hsa-miR-1-3p; hsa-miR-877-5p; hsa-miR-26b-5p; hsa-miR-320a; hsa-let-7g-5p; hsa-miR-103a-3p; hsa-miR-1250-5p; hsa-miR-1227-3p; hsa-miR-106b-5p; hsa-miR-3188; hsa-miR-760; hsa-miR-1287-5p; hsa-miR-146a-5p; hsa-miR-142-3p; hsa-miR-107; hsa-miR-16-5p; hsa-miR-20a-5p; hsa-miR-24-3p; hsa-miR-92b-3p; hsa-miR-422a; hsa-miR-29b-3p; hsa-miR-671-5p; hsa-miR-181a-5p; hsa-miR-181d-5p; hsa-miR-93-5p; hsa-miR-181b-5p; hsa-miR-124-3p; hsa-miR-595; hsa-miR-21-5p; hsa-miR-29a-3p; hsa-miR-26a-5p; hsa-miR-99b-3p; hsa-miR-19a-3p; hsa-miR-1229-3p; hsa-miR-15a-5p; hsa-miR-1268a; hsa-miR-148a-3p; hsa-miR-940; hsa-miR-1224-5p; hsa-miR-636; hsa-miR-19b-3p; hsa-miR-25-3p; hsa-miR-766-3p; hsa-miR-17-5p; hsa-miR-576-5p; hsa-miR-18a-5p; hsa-miR-10a-5p; hsa-miR-15b-5p; hsa-miR-148b-3p; hsa-miR-149-5p; hsa-miR-3605-3p; hsa-miR-23a-3p; hsa-miR-504-5p; hsa-miR-92a-3p; hsa-miR-615-3p; hsa-miR-185-3p; hsa-miR-1237-3p; hsa-miR-505-3p; hsa-miR-1260b; hsa-miR-30b-5p; hsa-miR-130b-5p; hsa-miR-204-5p; hsa-miR-1226-3p; hsa-miR-331-3p; hsa-miR-1914-5p; hsa-miR-1236-3p; hsa-miR-197-3p; hsa-miR-346; hsa-miR-30c-5p; hsa-miR-328-3p; hsa-miR-1296-5p; hsa-miR-196b-3p
Gene Ontology (miRWalk)	GO0002009 morphogenesis of an epithelium	enriched	0.0470863	11	hsa-miR-29c-3p; hsa-miR-1-3p; hsa-miR-26b-5p; hsa-miR-320a; hsa-miR-16-5p; hsa-miR-124-3p; hsa-miR-21-5p; hsa-miR-26a-5p; hsa-miR-99b-3p; hsa-miR-615-3p; hsa-miR-130b-5p

Gene Ontology (miRWalk)	GO0042326 negative regulation of phosphorylation	enriched	0.0471055	32	hsa-let-7c-5p; hsa-miR-155-5p; hsa-let-7a-5p; hsa-let-7f-5p; hsa-miR-1-3p; hsa-miR-877-5p; hsa-miR-26b-5p; hsa-miR-320a; hsa-let-7g-5p; hsa-miR-106b-5p; hsa-miR-146a-5p; hsa-miR-16-5p; hsa-miR-20a-5p; hsa-miR-24-3p; hsa-miR-92b-3p; hsa-miR-181a-5p; hsa-miR-93-5p; hsa-miR-181b-5p; hsa-miR-124-3p; hsa-miR-1229-3p; hsa-miR-520a-3p; hsa-miR-20b-5p; hsa-miR-25-3p; hsa-miR-17-5p; hsa-miR-572; hsa-miR-148b-3p; hsa-miR-92a-3p; hsa-miR-299-5p; hsa-miR-615-3p; hsa-miR-1260b; hsa-miR-331-3p; hsa-miR-296-5p
Gene Ontology (miRWalk)	GO0051056 regulation of small gtpase mediated signal transduction	enriched	0.0472185	45	hsa-miR-29c-3p; hsa-let-7c-5p; hsa-miR-155-5p; hsa-let-7a-5p; hsa-miR-324-3p; hsa-miR-1-3p; hsa-miR-877-5p; hsa-miR-26b-5p; hsa-miR-320a; hsa-miR-103a-3p; hsa-miR-106b-5p; hsa-miR-760; hsa-miR-142-3p; hsa-miR-16-5p; hsa-miR-20a-5p; hsa-miR-24-3p; hsa-miR-92b-3p; hsa-miR-29b-3p; hsa-miR-181a-5p; hsa-miR-93-5p; hsa-miR-181b-5p; hsa-miR-124-3p; hsa-miR-21-5p; hsa-miR-223-3p; hsa-miR-29a-3p; hsa-miR-26a-5p; hsa-miR-15a-5p; hsa-miR-940; hsa-miR-320c; hsa-miR-19b-3p; hsa-miR-25-3p; hsa-miR-17-5p; hsa-miR-18a-5p; hsa-miR-10a-5p; hsa-miR-148b-3p; hsa-miR-149-5p; hsa-miR-92a-3p; hsa-miR-615-3p; hsa-miR-204-5p; hsa-miR-331-3p; hsa-miR-1914-5p; hsa-miR-197-3p; hsa-miR-30c-5p; hsa-miR-4326; hsa-miR-328-3p
Gene Ontology (miRWalk)	GO0006655 phosphatidylglycerol biosynthetic process	enriched	0.0473732	5	hsa-miR-30d-5p; hsa-miR-877-5p; hsa-miR-320a; hsa-miR-16-5p; hsa-miR-21-5p
Gene Ontology (miRWalk)	GO0007023 post chaperonin tubulin folding pathway	enriched	0.0473732	5	hsa-miR-155-5p; hsa-miR-1-3p; hsa-miR-877-5p; hsa-miR-93-5p; hsa-miR-21-5p
Gene Ontology (miRWalk)	GO0007567 parturition	enriched	0.0473732	5	hsa-miR-1-3p; hsa-miR-142-3p; hsa-miR-181a-5p; hsa-miR-124-3p; hsa-miR-21-5p
Gene Ontology (miRWalk)	GO0009103 lipopolysaccharide biosynthetic process	enriched	0.0473732	5	hsa-miR-155-5p; hsa-miR-320a; hsa-miR-24-3p; hsa-miR-124-3p; hsa-miR-21-5p
Gene Ontology (miRWalk)	GO0010633 negative regulation of epithelial cell migration	enriched	0.0473732	5	hsa-miR-155-5p; hsa-miR-1-3p; hsa-miR-26b-5p; hsa-miR-181a-5p; hsa-miR-21-5p
Gene Ontology (miRWalk)	GO0045178 basal part of cell	enriched	0.0473732	5	hsa-miR-1-3p; hsa-miR-103a-3p; hsa-miR-16-5p; hsa-miR-24-3p; hsa-miR-21-5p
Gene Ontology (miRWalk)	GO0045359 positive regulation of interferon beta biosynthetic process	enriched	0.0473732	5	hsa-miR-1-3p; hsa-let-7i-5p; hsa-miR-146a-5p; hsa-miR-124-3p; hsa-miR-21-5p
Gene Ontology (miRWalk)	GO2000648 positive regulation of stem cell proliferation	enriched	0.0473732	9	hsa-let-7c-5p; hsa-miR-155-5p; hsa-let-7a-5p; hsa-let-7f-5p; hsa-let-7g-5p; hsa-miR-760; hsa-miR-26a-5p; hsa-miR-148b-3p; hsa-miR-1226-3p
Diseases (miRWalk)	Lymphoma	enriched	0.0477929	15	hsa-miR-29c-3p; hsa-miR-155-5p; hsa-let-7a-5p; hsa-let-7f-5p; hsa-miR-16-5p; hsa-miR-20a-5p; hsa-miR-29b-3p; hsa-miR-181a-5p; hsa-miR-21-5p; hsa-miR-29a-3p; hsa-miR-26a-5p; hsa-miR-15a-5p; hsa-miR-17-5p; hsa-miR-30b-5p; hsa-miR-34b-3p
Organs (miRWalk)	Myocardium	enriched	0.0477936	2	hsa-miR-155-5p; hsa-miR-1-3p

Pathways (miRWalk)	P00019 Endothelin signaling pathway	enriched	0.0480086	45	hsa-miR-29c-3p; hsa-let-7c-5p; hsa-miR-155-5p; hsa-let-7a-5p; hsa-miR-324-3p; hsa-miR-1-3p; hsa-miR-877-5p; hsa-miR-26b-5p; hsa-miR-320a; hsa-miR-103a-3p; hsa-let-7i-5p; hsa-miR-451a; hsa-miR-184; hsa-miR-142-3p; hsa-miR-16-5p; hsa-miR-24-3p; hsa-miR-92b-3p; hsa-miR-365a-3p; hsa-miR-181a-5p; hsa-miR-483-5p; hsa-miR-93-5p; hsa-miR-124-3p; hsa-miR-21-5p; hsa-miR-29a-3p; hsa-miR-15a-5p; hsa-miR-449c-5p; hsa-miR-19b-3p; hsa-miR-25-3p; hsa-miR-766-3p; hsa-miR-17-5p; hsa-miR-10a-5p; hsa-miR-15b-5p; hsa-miR-148b-3p; hsa-miR-149-5p; hsa-miR-92a-3p; hsa-miR-615-3p; hsa-miR-505-3p; hsa-miR-1260b; hsa-miR-204-5p; hsa-miR-1226-3p; hsa-miR-331-3p; hsa-miR-197-3p; hsa-miR-346; hsa-miR-30c-5p; hsa-miR-1296-5p
Pathways (miRWalk)	WP1403 AMPK signaling	enriched	0.0480086	55	hsa-miR-29c-3p; hsa-miR-155-5p; hsa-let-7a-5p; hsa-miR-324-3p; hsa-let-7f-5p; hsa-miR-30d-5p; hsa-miR-1-3p; hsa-miR-877-5p; hsa-miR-26b-5p; hsa-miR-320a; hsa-miR-103a-3p; hsa-miR-106b-5p; hsa-miR-451a; hsa-miR-184; hsa-miR-146a-5p; hsa-miR-16-5p; hsa-miR-20a-5p; hsa-miR-24-3p; hsa-miR-92b-3p; hsa-miR-765; hsa-miR-29b-3p; hsa-miR-93-5p; hsa-miR-181b-5p; hsa-miR-124-3p; hsa-miR-21-5p; hsa-miR-223-3p; hsa-miR-29a-3p; hsa-miR-26a-5p; hsa-miR-99b-3p; hsa-miR-1229-3p; hsa-miR-15a-5p; hsa-miR-1268a; hsa-miR-520a-3p; hsa-miR-20b-5p; hsa-miR-19b-3p; hsa-miR-25-3p; hsa-miR-766-3p; hsa-miR-17-5p; hsa-miR-18a-5p; hsa-miR-572; hsa-miR-10a-5p; hsa-miR-148b-3p; hsa-miR-149-5p; hsa-miR-504-5p; hsa-miR-92a-3p; hsa-miR-299-5p; hsa-miR-615-3p; hsa-miR-1260b; hsa-miR-130b-5p; hsa-miR-331-3p; hsa-miR-197-3p; hsa-miR-346; hsa-miR-30c-5p; hsa-miR-605-5p; hsa-miR-1296-5p
Pathways (miRWalk)	WP311 Synthesis and Degradation of Ketone Bodies	enriched	0.0480086	6	hsa-miR-155-5p; hsa-miR-1-3p; hsa-miR-26b-5p; hsa-miR-106b-5p; hsa-miR-21-5p; hsa-miR-1260b
Pathways (miRWalk)	WP581 EPO Receptor Signaling	enriched	0.0480086	29	hsa-miR-155-5p; hsa-miR-324-3p; hsa-miR-26b-5p; hsa-miR-320a; hsa-let-7i-5p; hsa-miR-451a; hsa-miR-146a-5p; hsa-miR-16-5p; hsa-miR-20a-5p; hsa-miR-92b-3p; hsa-miR-181a-5p; hsa-miR-483-5p; hsa-miR-93-5p; hsa-miR-124-3p; hsa-miR-21-5p; hsa-miR-26a-5p; hsa-miR-19a-3p; hsa-miR-1229-3p; hsa-miR-20b-5p; hsa-miR-148a-3p; hsa-miR-19b-3p; hsa-miR-766-3p; hsa-miR-15b-5p; hsa-miR-148b-3p; hsa-miR-92a-3p; hsa-miR-615-3p; hsa-miR-30b-5p; hsa-miR-337-3p; hsa-miR-30c-5p
Gene Ontology (miRWalk)	GO0030659 cytoplasmic vesicle membrane	enriched	0.0480436	44	hsa-miR-29c-3p; hsa-let-7c-5p; hsa-miR-155-5p; hsa-let-7a-5p; hsa-miR-324-3p; hsa-miR-30d-5p; hsa-miR-1-3p; hsa-miR-877-5p; hsa-miR-26b-5p; hsa-miR-320a; hsa-let-7g-5p; hsa-miR-103a-3p; hsa-miR-106b-5p; hsa-miR-142-3p; hsa-miR-107; hsa-miR-16-5p; hsa-miR-20a-5p; hsa-miR-92b-3p; hsa-miR-29b-3p; hsa-miR-181a-5p; hsa-miR-93-5p; hsa-miR-124-3p; hsa-miR-21-5p; hsa-miR-29a-3p; hsa-miR-26a-5p; hsa-miR-19a-3p; hsa-miR-15a-5p; hsa-miR-148a-3p; hsa-miR-19b-3p; hsa-miR-25-3p; hsa-miR-17-5p; hsa-miR-18a-5p; hsa-miR-15b-5p; hsa-miR-148b-3p; hsa-miR-23a-3p; hsa-miR-92a-3p; hsa-miR-615-3p; hsa-miR-30b-5p; hsa-miR-204-5p; hsa-miR-331-3p; hsa-miR-1914-5p; hsa-miR-30c-5p; hsa-miR-328-3p; hsa-miR-1296-5p
Gene Ontology (miRWalk)	GO0034613 cellular protein localization	enriched	0.0480436	33	hsa-miR-29c-3p; hsa-let-7c-5p; hsa-miR-155-5p; hsa-let-7a-5p; hsa-miR-324-3p; hsa-miR-30d-5p; hsa-miR-1-3p; hsa-miR-26b-5p; hsa-miR-103a-3p; hsa-miR-106b-5p; hsa-miR-16-5p; hsa-miR-20a-5p; hsa-miR-24-3p; hsa-miR-92b-3p; hsa-miR-29b-3p; hsa-miR-181a-5p; hsa-miR-93-5p; hsa-miR-124-3p; hsa-miR-21-5p; hsa-miR-29a-3p; hsa-miR-15a-5p; hsa-miR-25-3p; hsa-miR-766-3p; hsa-miR-10a-5p; hsa-miR-148b-3p; hsa-miR-149-5p; hsa-miR-504-5p; hsa-miR-92a-3p; hsa-miR-615-3p; hsa-miR-30b-5p; hsa-miR-331-3p; hsa-miR-30c-5p; hsa-miR-605-5p

Gene Ontology (miRWalk)	GO0016049 cell growth	enriched	0.0483509	44	hsa-miR-29c-3p; hsa-let-7c-5p; hsa-miR-155-5p; hsa-let-7a-5p; hsa-let-7f-5p; hsa-miR-1-3p; hsa-miR-877-5p; hsa-miR-26b-5p; hsa-miR-103a-3p; hsa-miR-106b-5p; hsa-miR-760; hsa-miR-451a; hsa-miR-107; hsa-miR-16-5p; hsa-miR-20a-5p; hsa-miR-24-3p; hsa-miR-29b-3p; hsa-miR-365a-3p; hsa-miR-181a-5p; hsa-miR-181d-5p; hsa-miR-93-5p; hsa-miR-181b-5p; hsa-miR-124-3p; hsa-miR-21-5p; hsa-miR-29a-3p; hsa-miR-1229-3p; hsa-miR-15a-5p; hsa-miR-148a-3p; hsa-miR-25-3p; hsa-miR-17-5p; hsa-miR-18a-5p; hsa-miR-630; hsa-miR-10a-5p; hsa-miR-15b-5p; hsa-miR-149-5p; hsa-miR-23a-3p; hsa-miR-92a-3p; hsa-miR-615-3p; hsa-miR-1260b; hsa-miR-130b-5p; hsa-miR-204-5p; hsa-miR-331-3p; hsa-miR-197-3p; hsa-miR-296-5p
Gene Ontology (miRWalk)	GO0007257 activation of jun kinase activity	enriched	0.048467	22	hsa-miR-155-5p; hsa-miR-1-3p; hsa-miR-26b-5p; hsa-miR-320a; hsa-miR-425-3p; hsa-miR-16-5p; hsa-miR-24-3p; hsa-miR-92b-3p; hsa-miR-181a-5p; hsa-miR-181d-5p; hsa-miR-181b-5p; hsa-miR-124-3p; hsa-miR-21-5p; hsa-miR-26a-5p; hsa-miR-99b-3p; hsa-miR-320c; hsa-miR-17-5p; hsa-miR-18a-5p; hsa-miR-148b-3p; hsa-miR-92a-3p; hsa-miR-615-3p; hsa-miR-331-3p
Gene Ontology (miRWalk)	GO0009615 response to virus	enriched	0.048467	54	hsa-miR-29c-3p; hsa-let-7c-5p; hsa-miR-155-5p; hsa-let-7a-5p; hsa-miR-324-3p; hsa-let-7f-5p; hsa-miR-30d-5p; hsa-miR-1-3p; hsa-miR-877-5p; hsa-miR-26b-5p; hsa-miR-320a; hsa-let-7g-5p; hsa-miR-103a-3p; hsa-miR-106b-5p; hsa-miR-760; hsa-miR-146a-5p; hsa-miR-107; hsa-miR-16-5p; hsa-miR-24-3p; hsa-miR-92b-3p; hsa-miR-29b-3p; hsa-miR-671-5p; hsa-miR-181a-5p; hsa-miR-181d-5p; hsa-miR-181b-5p; hsa-miR-124-3p; hsa-miR-21-5p; hsa-miR-223-3p; hsa-miR-29a-3p; hsa-miR-26a-5p; hsa-miR-1229-3p; hsa-miR-15a-5p; hsa-miR-320c; hsa-miR-25-3p; hsa-miR-17-5p; hsa-miR-10a-5p; hsa-miR-15b-5p; hsa-miR-148b-3p; hsa-miR-149-5p; hsa-miR-23a-3p; hsa-miR-92a-3p; hsa-miR-615-3p; hsa-miR-1260a; hsa-miR-629-3p; hsa-miR-1260b; hsa-miR-130b-5p; hsa-miR-204-5p; hsa-miR-1226-3p; hsa-miR-331-3p; hsa-miR-1914-5p; hsa-miR-197-3p; hsa-miR-34b-3p; hsa-miR-30c-5p; hsa-miR-1296-5p
Gene Ontology (miRWalk)	GO0035102 prc1 complex	enriched	0.048467	16	hsa-miR-155-5p; hsa-miR-1-3p; hsa-miR-26b-5p; hsa-let-7g-5p; hsa-miR-103a-3p; hsa-miR-16-5p; hsa-miR-20a-5p; hsa-miR-24-3p; hsa-miR-93-5p; hsa-miR-181b-5p; hsa-miR-124-3p; hsa-miR-1229-3p; hsa-miR-15a-5p; hsa-miR-766-3p; hsa-miR-92a-3p; hsa-miR-328-3p
Gene Ontology (miRWalk)	GO0045987 positive regulation of smooth muscle contraction	enriched	0.048467	22	hsa-let-7c-5p; hsa-miR-155-5p; hsa-let-7a-5p; hsa-miR-324-3p; hsa-miR-1-3p; hsa-miR-26b-5p; hsa-miR-320a; hsa-miR-550a-5p; hsa-miR-760; hsa-miR-16-5p; hsa-miR-92b-3p; hsa-miR-181a-5p; hsa-miR-124-3p; hsa-miR-26a-5p; hsa-miR-99b-3p; hsa-miR-320c; hsa-miR-766-3p; hsa-miR-17-5p; hsa-miR-149-5p; hsa-miR-92a-3p; hsa-miR-615-3p; hsa-miR-331-3p
Gene Ontology (miRWalk)	GO0001669 acrosomal vesicle	enriched	0.048722	17	hsa-miR-155-5p; hsa-let-7a-5p; hsa-miR-1-3p; hsa-miR-26b-5p; hsa-miR-320a; hsa-miR-103a-3p; hsa-miR-142-3p; hsa-miR-16-5p; hsa-miR-24-3p; hsa-miR-181a-5p; hsa-miR-124-3p; hsa-miR-21-5p; hsa-miR-25-3p; hsa-miR-18a-5p; hsa-miR-149-5p; hsa-miR-615-3p; hsa-miR-34b-3p
Gene Ontology (miRWalk)	GO0034612 response to tumor necrosis factor	enriched	0.048722	17	hsa-miR-155-5p; hsa-let-7a-5p; hsa-miR-30d-5p; hsa-miR-1-3p; hsa-miR-877-5p; hsa-miR-26b-5p; hsa-miR-103a-3p; hsa-miR-451a; hsa-miR-16-5p; hsa-miR-24-3p; hsa-miR-124-3p; hsa-miR-21-5p; hsa-miR-17-5p; hsa-miR-18a-5p; hsa-miR-10a-5p; hsa-miR-23a-3p; hsa-miR-92a-3p
Gene Ontology (miRWalk)	GO0001942 hair follicle development	enriched	0.0491425	21	hsa-miR-29c-3p; hsa-let-7c-5p; hsa-miR-155-5p; hsa-let-7a-5p; hsa-miR-1-3p; hsa-miR-26b-5p; hsa-miR-320a; hsa-miR-103a-3p; hsa-miR-106b-5p; hsa-miR-146a-5p; hsa-miR-16-5p; hsa-miR-24-3p; hsa-miR-21-5p; hsa-miR-29a-3p; hsa-miR-15a-5p; hsa-miR-148a-3p; hsa-miR-10a-5p; hsa-miR-148b-3p; hsa-miR-92a-3p; hsa-miR-615-3p; hsa-miR-197-3p

Gene Ontology (miRWalk)	GO0007173 epidermal growth factor receptor signaling pathway	enriched	0.0491425	73	hsa-miR-29c-3p; hsa-let-7c-5p; hsa-miR-155-5p; hsa-let-7a-5p; hsa-miR-324-3p; hsa-miR-1-3p; hsa-miR-877-5p; hsa-miR-26b-5p; hsa-miR-320a; hsa-let-7g-5p; hsa-miR-103a-3p; hsa-miR-199b-5p; hsa-miR-106b-5p; hsa-miR-18b-5p; hsa-let-7i-5p; hsa-miR-760; hsa-miR-1287-5p; hsa-miR-451a; hsa-miR-146a-5p; hsa-miR-548d-3p; hsa-miR-142-3p; hsa-miR-107; hsa-miR-16-5p; hsa-miR-20a-5p; hsa-miR-24-3p; hsa-miR-92b-3p; hsa-miR-29b-3p; hsa-miR-365a-3p; hsa-miR-181a-5p; hsa-miR-483-5p; hsa-miR-181d-5p; hsa-miR-93-5p; hsa-miR-181b-5p; hsa-miR-124-3p; hsa-miR-21-5p; hsa-miR-223-3p; hsa-miR-29a-3p; hsa-miR-26a-5p; hsa-miR-19a-3p; hsa-miR-1229-3p; hsa-miR-1914-3p; hsa-miR-15a-5p; hsa-miR-148a-3p; hsa-miR-19b-3p; hsa-miR-494-3p; hsa-miR-25-3p; hsa-miR-766-3p; hsa-miR-17-5p; hsa-miR-18a-5p; hsa-miR-10a-5p; hsa-miR-15b-5p; hsa-miR-148b-3p; hsa-miR-149-5p; hsa-miR-663a; hsa-miR-23a-3p; hsa-miR-504-5p; hsa-miR-92a-3p; hsa-miR-615-3p; hsa-miR-505-3p; hsa-miR-1260b; hsa-miR-30b-5p; hsa-miR-130b-5p; hsa-miR-204-5p; hsa-miR-1226-3p; hsa-miR-331-3p; hsa-miR-1914-5p; hsa-miR-197-3p; hsa-miR-744-3p; hsa-miR-30c-5p; hsa-miR-605-5p; hsa-miR-296-5p; hsa-miR-4326; hsa-miR-1296-5p
Gene Ontology (miRWalk)	GO0007411 axon guidance	enriched	0.0491425	73	hsa-miR-29c-3p; hsa-let-7c-5p; hsa-miR-155-5p; hsa-let-7a-5p; hsa-miR-584-5p; hsa-miR-324-3p; hsa-let-7f-5p; hsa-miR-1-3p; hsa-miR-877-5p; hsa-miR-26b-5p; hsa-miR-320a; hsa-let-7g-5p; hsa-miR-103a-3p; hsa-miR-1227-3p; hsa-miR-199b-5p; hsa-miR-106b-5p; hsa-miR-18b-5p; hsa-miR-760; hsa-miR-146a-5p; hsa-miR-1295a; hsa-miR-548d-3p; hsa-miR-142-3p; hsa-miR-16-5p; hsa-miR-20a-5p; hsa-miR-24-3p; hsa-miR-92b-3p; hsa-miR-29b-3p; hsa-miR-181a-5p; hsa-miR-483-5p; hsa-miR-181d-5p; hsa-miR-93-5p; hsa-miR-181b-5p; hsa-miR-124-3p; hsa-miR-21-5p; hsa-miR-223-3p; hsa-miR-29a-3p; hsa-miR-26a-5p; hsa-miR-99b-3p; hsa-miR-19a-3p; hsa-miR-1229-3p; hsa-miR-1914-3p; hsa-miR-15a-5p; hsa-miR-148a-3p; hsa-miR-320c; hsa-miR-19b-3p; hsa-miR-25-3p; hsa-miR-766-3p; hsa-miR-17-5p; hsa-miR-18a-5p; hsa-miR-10a-5p; hsa-miR-15b-5p; hsa-miR-148b-3p; hsa-miR-149-5p; hsa-miR-663a; hsa-miR-23a-3p; hsa-miR-92a-3p; hsa-miR-615-3p; hsa-miR-505-3p; hsa-miR-30b-5p; hsa-miR-130b-5p; hsa-miR-1910-5p; hsa-miR-204-5p; hsa-miR-337-3p; hsa-miR-1226-3p; hsa-miR-331-3p; hsa-miR-197-3p; hsa-miR-346; hsa-miR-744-3p; hsa-miR-34b-3p; hsa-miR-30c-5p; hsa-miR-4326; hsa-miR-328-3p; hsa-miR-1296-5p
Gene Ontology (miRWalk)	GO0035970 peptidyl threonine dephosphorylation	enriched	0.0491425	8	hsa-miR-29c-3p; hsa-miR-155-5p; hsa-miR-26b-5p; hsa-miR-106b-5p; hsa-miR-16-5p; hsa-miR-24-3p; hsa-miR-29a-3p; hsa-miR-10a-5p
Gene Ontology (miRWalk)	GO0043125 erbb 3 class receptor binding	enriched	0.0491425	8	hsa-miR-155-5p; hsa-let-7a-5p; hsa-miR-199b-5p; hsa-miR-548d-3p; hsa-miR-124-3p; hsa-miR-21-5p; hsa-miR-29a-3p; hsa-miR-331-3p
Gene Ontology (miRWalk)	GO0005852 eukaryotic translation initiation factor 3 complex	enriched	0.0492721	24	hsa-let-7c-5p; hsa-miR-155-5p; hsa-let-7a-5p; hsa-miR-324-3p; hsa-let-7f-5p; hsa-miR-26b-5p; hsa-miR-320a; hsa-miR-106b-5p; hsa-miR-16-5p; hsa-miR-92b-3p; hsa-miR-181a-5p; hsa-miR-181b-5p; hsa-miR-124-3p; hsa-miR-21-5p; hsa-miR-29a-3p; hsa-miR-99b-3p; hsa-miR-320c; hsa-miR-19b-3p; hsa-miR-149-5p; hsa-miR-92a-3p; hsa-miR-615-3p; hsa-miR-629-3p; hsa-miR-130b-5p; hsa-miR-1226-3p

Gene Ontology (miRWalk)	GO0006928 cellular component movement	enriched	0.0492721	54	hsa-let-7c-5p; hsa-miR-155-5p; hsa-let-7a-5p; hsa-miR-324-3p; hsa-let-7f-5p; hsa-miR-1-3p; hsa-miR-877-5p; hsa-miR-26b-5p; hsa-miR-320a; hsa-let-7g-5p; hsa-miR-103a-3p; hsa-miR-1227-3p; hsa-miR-106b-5p; hsa-let-7i-5p; hsa-miR-760; hsa-miR-146a-5p; hsa-miR-1295a; hsa-miR-142-3p; hsa-miR-107; hsa-miR-16-5p; hsa-miR-20a-5p; hsa-miR-24-3p; hsa-miR-92b-3p; hsa-miR-29b-3p; hsa-miR-365a-3p; hsa-miR-181a-5p; hsa-miR-93-5p; hsa-miR-124-3p; hsa-miR-21-5p; hsa-miR-99b-3p; hsa-miR-1229-3p; hsa-miR-15a-5p; hsa-miR-20b-5p; hsa-miR-940; hsa-miR-320c; hsa-miR-1224-5p; hsa-miR-19b-3p; hsa-miR-25-3p; hsa-miR-17-5p; hsa-miR-10a-5p; hsa-miR-15b-5p; hsa-miR-149-5p; hsa-miR-3605-3p; hsa-miR-92a-3p; hsa-miR-615-3p; hsa-miR-505-3p; hsa-miR-30b-5p; hsa-miR-130b-5p; hsa-miR-204-5p; hsa-miR-337-3p; hsa-miR-1226-3p; hsa-miR-331-3p; hsa-miR-197-3p; hsa-miR-30c-5p
Gene Ontology (miRWalk)	GO0007435 salivary gland morphogenesis	enriched	0.0492721	14	hsa-miR-155-5p; hsa-let-7a-5p; hsa-miR-324-3p; hsa-miR-1-3p; hsa-miR-146a-5p; hsa-miR-16-5p; hsa-miR-24-3p; hsa-miR-124-3p; hsa-miR-21-5p; hsa-miR-29a-3p; hsa-miR-1229-3p; hsa-miR-10a-5p; hsa-miR-615-3p; hsa-miR-331-3p
Gene Ontology (miRWalk)	GO0045739 positive regulation of dna repair	enriched	0.0492721	28	hsa-let-7c-5p; hsa-miR-155-5p; hsa-let-7a-5p; hsa-miR-1-3p; hsa-miR-877-5p; hsa-miR-26b-5p; hsa-miR-320a; hsa-miR-146a-5p; hsa-miR-107; hsa-miR-16-5p; hsa-miR-24-3p; hsa-miR-92b-3p; hsa-miR-181a-5p; hsa-miR-181d-5p; hsa-miR-181b-5p; hsa-miR-124-3p; hsa-miR-21-5p; hsa-miR-99b-3p; hsa-miR-15a-5p; hsa-miR-17-5p; hsa-miR-148b-3p; hsa-miR-149-5p; hsa-miR-92a-3p; hsa-miR-521; hsa-miR-505-3p; hsa-miR-30b-5p; hsa-miR-331-3p; hsa-miR-328-3p
Gene Ontology (miRWalk)	GO0060136 embryonic process involved in female pregnancy	enriched	0.0492721	15	hsa-miR-155-5p; hsa-let-7a-5p; hsa-let-7f-5p; hsa-miR-1-3p; hsa-miR-26b-5p; hsa-miR-103a-3p; hsa-miR-16-5p; hsa-miR-29b-3p; hsa-miR-124-3p; hsa-miR-21-5p; hsa-miR-223-3p; hsa-miR-149-5p; hsa-miR-663a; hsa-miR-1260b; hsa-miR-1226-3p
Gene Ontology (miRWalk)	GO0042803 protein homodimerization activity	enriched	0.0493823	80	hsa-miR-29c-3p; hsa-let-7c-5p; hsa-miR-155-5p; hsa-let-7a-5p; hsa-miR-324-3p; hsa-let-7f-5p; hsa-miR-193a-5p; hsa-miR-30d-5p; hsa-miR-1-3p; hsa-miR-498; hsa-miR-877-5p; hsa-miR-26b-5p; hsa-miR-320a; hsa-miR-103a-3p; hsa-miR-1227-3p; hsa-miR-199b-5p; hsa-miR-106b-5p; hsa-miR-760; hsa-miR-451a; hsa-miR-146a-5p; hsa-miR-142-3p; hsa-miR-425-3p; hsa-miR-107; hsa-miR-16-5p; hsa-miR-20a-5p; hsa-miR-24-3p; hsa-miR-92b-3p; hsa-miR-765; hsa-miR-29b-3p; hsa-miR-671-5p; hsa-miR-365a-3p; hsa-miR-181a-5p; hsa-miR-181d-5p; hsa-miR-93-5p; hsa-miR-181b-5p; hsa-miR-124-3p; hsa-miR-21-5p; hsa-miR-223-3p; hsa-miR-29a-3p; hsa-miR-26a-5p; hsa-miR-134-5p; hsa-miR-19a-3p; hsa-miR-1229-3p; hsa-miR-15a-5p; hsa-miR-20b-5p; hsa-miR-148a-3p; hsa-miR-940; hsa-miR-320c; hsa-miR-19b-3p; hsa-miR-520g-3p; hsa-miR-1228-3p; hsa-miR-25-3p; hsa-miR-766-3p; hsa-miR-17-5p; hsa-miR-18a-5p; hsa-miR-630; hsa-miR-10a-5p; hsa-miR-15b-5p; hsa-miR-148b-3p; hsa-miR-149-5p; hsa-miR-23a-3p; hsa-miR-504-5p; hsa-miR-92a-3p; hsa-miR-937-3p; hsa-miR-615-3p; hsa-miR-505-3p; hsa-miR-483-3p; hsa-miR-1260b; hsa-miR-30b-5p; hsa-miR-204-5p; hsa-miR-1226-3p; hsa-miR-331-3p; hsa-miR-197-3p; hsa-miR-346; hsa-miR-744-3p; hsa-miR-34b-3p; hsa-miR-30c-5p; hsa-miR-296-5p; hsa-miR-328-3p; hsa-miR-1296-5p

Gene Ontology (miRWalk)	GO0007160 cell matrix adhesion	enriched	0.0494144	42	hsa-miR-29c-3p; hsa-let-7c-5p; hsa-miR-155-5p; hsa-let-7a-5p; hsa-miR-324-3p; hsa-let-7f-5p; hsa-miR-1-3p; hsa-miR-26b-5p; hsa-miR-320a; hsa-miR-103a-3p; hsa-miR-106b-5p; hsa-miR-146a-5p; hsa-miR-142-3p; hsa-miR-16-5p; hsa-miR-24-3p; hsa-miR-92b-3p; hsa-miR-29b-3p; hsa-miR-181a-5p; hsa-miR-181d-5p; hsa-miR-93-5p; hsa-miR-124-3p; hsa-miR-21-5p; hsa-miR-29a-3p; hsa-miR-26a-5p; hsa-miR-19a-3p; hsa-miR-320c; hsa-miR-19b-3p; hsa-miR-494-3p; hsa-miR-25-3p; hsa-miR-17-5p; hsa-miR-18a-5p; hsa-miR-15b-5p; hsa-miR-148b-3p; hsa-miR-23a-3p; hsa-miR-92a-3p; hsa-miR-615-3p; hsa-miR-130b-5p; hsa-miR-204-5p; hsa-miR-1226-3p; hsa-miR-331-3p; hsa-miR-30c-5p; hsa-miR-328-3p
Gene Ontology (miRWalk)	GO0051290 protein heterotetramerization	enriched	0.0494386	15	hsa-miR-155-5p; hsa-let-7a-5p; hsa-miR-1-3p; hsa-miR-26b-5p; hsa-miR-320a; hsa-let-7g-5p; hsa-miR-106b-5p; hsa-let-7i-5p; hsa-miR-24-3p; hsa-miR-124-3p; hsa-miR-26a-5p; hsa-miR-148b-3p; hsa-miR-92a-3p; hsa-miR-615-3p; hsa-miR-30c-5p
Pathways (miRWalk)	P00057 Wnt signaling pathway	enriched	0.0494549	75	hsa-miR-29c-3p; hsa-let-7c-5p; hsa-miR-155-5p; hsa-let-7a-5p; hsa-miR-324-3p; hsa-let-7f-5p; hsa-miR-30d-5p; hsa-miR-1-3p; hsa-miR-877-5p; hsa-miR-26b-5p; hsa-miR-320a; hsa-let-7g-5p; hsa-miR-103a-3p; hsa-miR-106b-5p; hsa-let-7i-5p; hsa-miR-760; hsa-miR-451a; hsa-miR-184; hsa-miR-146a-5p; hsa-miR-142-3p; hsa-miR-107; hsa-miR-16-5p; hsa-miR-20a-5p; hsa-miR-24-3p; hsa-miR-92b-3p; hsa-miR-29b-3p; hsa-miR-671-5p; hsa-miR-365a-3p; hsa-miR-181a-5p; hsa-miR-181d-5p; hsa-miR-93-5p; hsa-miR-181b-5p; hsa-miR-124-3p; hsa-miR-21-5p; hsa-miR-223-3p; hsa-miR-29a-3p; hsa-miR-26a-5p; hsa-miR-19a-3p; hsa-miR-1229-3p; hsa-miR-15a-5p; hsa-miR-449c-5p; hsa-miR-148a-3p; hsa-miR-940; hsa-miR-320c; hsa-miR-19b-3p; hsa-miR-25-3p; hsa-miR-17-5p; hsa-miR-18a-5p; hsa-miR-10a-5p; hsa-miR-15b-5p; hsa-miR-148b-3p; hsa-miR-149-5p; hsa-miR-23a-3p; hsa-miR-504-5p; hsa-miR-92a-3p; hsa-miR-615-3p; hsa-miR-505-3p; hsa-miR-483-3p; hsa-miR-1260b; hsa-miR-30b-5p; hsa-miR-130b-5p; hsa-miR-204-5p; hsa-miR-181a-2-3p; hsa-miR-337-3p; hsa-miR-1226-3p; hsa-miR-331-3p; hsa-miR-1914-5p; hsa-miR-197-3p; hsa-miR-744-3p; hsa-miR-34b-3p; hsa-miR-211-5p; hsa-miR-30c-5p; hsa-miR-605-5p; hsa-miR-296-5p; hsa-miR-328-3p
Pathways (miRWalk)	hsa04722 Neurotrophin signaling pathway	enriched	0.0494549	75	hsa-miR-29c-3p; hsa-let-7c-5p; hsa-miR-155-5p; hsa-miR-193b-5p; hsa-let-7a-5p; hsa-let-7f-5p; hsa-miR-193a-5p; hsa-miR-30d-5p; hsa-miR-1-3p; hsa-miR-877-5p; hsa-miR-26b-5p; hsa-miR-320a; hsa-let-7g-5p; hsa-miR-103a-3p; hsa-miR-106b-5p; hsa-miR-760; hsa-miR-451a; hsa-miR-184; hsa-miR-146a-5p; hsa-miR-142-3p; hsa-miR-107; hsa-miR-16-5p; hsa-miR-20a-5p; hsa-miR-24-3p; hsa-miR-92b-3p; hsa-miR-765; hsa-miR-29b-3p; hsa-miR-365a-3p; hsa-miR-181a-5p; hsa-miR-483-5p; hsa-miR-181d-5p; hsa-miR-93-5p; hsa-miR-181b-5p; hsa-miR-124-3p; hsa-miR-21-5p; hsa-miR-29a-3p; hsa-miR-26a-5p; hsa-miR-19a-3p; hsa-miR-1229-3p; hsa-miR-1914-3p; hsa-miR-15a-5p; hsa-miR-148a-3p; hsa-miR-320c; hsa-miR-25-3p; hsa-miR-766-3p; hsa-miR-17-5p; hsa-miR-18a-5p; hsa-miR-630; hsa-miR-10a-5p; hsa-miR-129-1-3p; hsa-miR-15b-5p; hsa-miR-148b-3p; hsa-miR-149-5p; hsa-miR-23a-3p; hsa-miR-504-5p; hsa-miR-92a-3p; hsa-miR-615-3p; hsa-miR-1260b; hsa-miR-30b-5p; hsa-miR-130b-5p; hsa-miR-1910-5p; hsa-let-7g-3p; hsa-miR-204-5p; hsa-miR-337-3p; hsa-miR-1226-3p; hsa-miR-331-3p; hsa-miR-197-3p; hsa-miR-346; hsa-miR-744-3p; hsa-miR-30c-5p; hsa-miR-605-5p; hsa-miR-296-5p; hsa-miR-328-3p; hsa-miR-1296-5p; hsa-miR-485-3p
Gene Ontology (miRWalk)	GO0001103 rna polymerase ii repressing transcription factor binding	enriched	0.0495802	19	hsa-miR-155-5p; hsa-let-7a-5p; hsa-let-7f-5p; hsa-miR-1-3p; hsa-miR-26b-5p; hsa-miR-16-5p; hsa-miR-20a-5p; hsa-miR-92b-3p; hsa-miR-29b-3p; hsa-miR-181a-5p; hsa-miR-181b-5p; hsa-miR-124-3p; hsa-miR-21-5p; hsa-miR-18a-5p;

					hsa-miR-149-5p; hsa-miR-92a-3p; hsa-miR-615-3p; hsa-miR-331-3p; hsa-miR-328-3p
Gene Ontology (miRWalk)	GO0019894 kinesin binding	enriched	0.0495802	19	hsa-let-7c-5p; hsa-miR-155-5p; hsa-let-7a-5p; hsa-miR-1-3p; hsa-miR-26b-5p; hsa-miR-320a; hsa-miR-106b-5p; hsa-miR-1295a; hsa-miR-16-5p; hsa-miR-24-3p; hsa-miR-93-5p; hsa-miR-124-3p; hsa-miR-21-5p; hsa-miR-191-3p; hsa-miR-19b-3p; hsa-miR-92a-3p; hsa-miR-331-3p; hsa-miR-346; hsa-miR-30c-5p
Gene Ontology (miRWalk)	GO0006979 response to oxidative stress	enriched	0.049669	47	hsa-let-7c-5p; hsa-miR-155-5p; hsa-let-7a-5p; hsa-miR-1-3p; hsa-miR-877-5p; hsa-miR-26b-5p; hsa-miR-320a; hsa-miR-103a-3p; hsa-miR-106b-5p; hsa-let-7i-5p; hsa-miR-550a-5p; hsa-miR-760; hsa-miR-451a; hsa-miR-146a-5p; hsa-miR-142-3p; hsa-miR-16-5p; hsa-miR-24-3p; hsa-miR-92b-3p; hsa-miR-181a-5p; hsa-miR-181d-5p; hsa-miR-93-5p; hsa-miR-181b-5p; hsa-miR-124-3p; hsa-miR-21-5p; hsa-miR-26a-5p; hsa-miR-99b-3p; hsa-miR-134-5p; hsa-miR-1229-3p; hsa-miR-15a-5p; hsa-miR-148a-3p; hsa-miR-940; hsa-miR-320c; hsa-miR-766-3p; hsa-miR-17-5p; hsa-miR-18a-5p; hsa-miR-10a-5p; hsa-miR-15b-5p; hsa-miR-148b-3p; hsa-miR-149-5p; hsa-miR-504-5p; hsa-miR-92a-3p; hsa-miR-615-3p; hsa-miR-521; hsa-miR-1260b; hsa-miR-1226-3p; hsa-miR-331-3p; hsa-miR-30c-5p
Gene Ontology (miRWalk)	GO0051225 spindle assembly	enriched	0.0499086	31	hsa-miR-29c-3p; hsa-let-7c-5p; hsa-let-7a-5p; hsa-miR-324-3p; hsa-miR-1-3p; hsa-miR-877-5p; hsa-miR-26b-5p; hsa-miR-320a; hsa-miR-103a-3p; hsa-miR-106b-5p; hsa-miR-107; hsa-miR-16-5p; hsa-miR-20a-5p; hsa-miR-24-3p; hsa-miR-154-5p; hsa-miR-365a-3p; hsa-miR-93-5p; hsa-miR-124-3p; hsa-miR-29a-3p; hsa-miR-15a-5p; hsa-miR-320c; hsa-miR-25-3p; hsa-miR-17-5p; hsa-miR-18a-5p; hsa-miR-10a-5p; hsa-miR-149-5p; hsa-miR-92a-3p; hsa-miR-615-3p; hsa-miR-505-3p; hsa-miR-1226-3p; hsa-miR-331-3p
Gene Ontology (miRWalk)	GO0031047 gene silencing by rna	enriched	0.0499909	23	hsa-miR-155-5p; hsa-let-7a-5p; hsa-miR-30d-5p; hsa-miR-1-3p; hsa-miR-26b-5p; hsa-miR-320a; hsa-miR-103a-3p; hsa-miR-760; hsa-miR-425-3p; hsa-miR-16-5p; hsa-miR-24-3p; hsa-miR-92b-3p; hsa-miR-93-5p; hsa-miR-124-3p; hsa-miR-21-5p; hsa-miR-1229-3p; hsa-miR-19b-3p; hsa-miR-18a-5p; hsa-miR-505-3p; hsa-miR-30b-5p; hsa-miR-1226-3p; hsa-miR-197-3p; hsa-miR-30c-5p

Supplemental Table 4: MiRNA target pathways

**miRNA**	**Target_Gene**	**Evidence**		**miRNA**	**Target_Gene**	**Evidence**		**miRNA**	**Target_Gene**	**Evidence**
hsa-miR-29c-3p	SIRT1	strong		hsa-miR-22-5p	EPHA4	weak		hsa-let-7c-5p	HUWE1	weak
hsa-let-7c-5p	TRIM71	strong		hsa-miR-22-5p	DUSP1	weak		hsa-let-7c-5p	RPS24	weak
hsa-let-7c-5p	DICER1	strong		hsa-miR-22-5p	CEBPB	weak		hsa-let-7c-5p	IRF2BP2	weak
hsa-let-7c-5p	CEBPB	strong		hsa-miR-22-5p	GRPEL2	weak		hsa-let-7c-5p	TMTC3	weak
hsa-miR-29c-3p	IRF2BP2	weak		hsa-miR-22-5p	TMCO1	weak		hsa-let-7c-5p	GRPEL2	weak
hsa-miR-29c-3p	CDV3	weak		hsa-miR-22-5p	CAND1	weak		hsa-let-7c-5p	MDM4	weak
hsa-miR-29c-3p	CCNT2	weak		hsa-miR-22-5p	UBXN2A	weak		hsa-let-7c-5p	NCOA3	weak
hsa-miR-29c-3p	DICER1	weak		hsa-miR-22-5p	CPA4	weak		hsa-let-7c-5p	NAA30	weak

hsa-miR-29c-3p	TMTC3	weak		hsa-miR-22-5p	RPS24	weak		hsa-let-7c-5p	CDV3	weak
hsa-miR-29c-3p	HDGF	weak		hsa-miR-6511b-5p	RABL2A	weak		hsa-let-7c-5p	MXD1	weak
hsa-miR-29c-3p	HUWE1	weak		hsa-miR-6511b-5p	RABL2B	weak		hsa-let-7c-5p	ARID3A	weak
hsa-miR-29c-3p	GOLGA7	weak		hsa-miR-6511b-5p	ZNF385A	weak		hsa-let-7c-5p	FXN	weak
hsa-miR-29c-3p	MXD1	weak		hsa-miR-6511b-5p	VCL	weak		hsa-let-7c-5p	RABL2A	weak
hsa-miR-29c-3p	TUBB2A	weak		hsa-miR-6511b-5p	SEC22C	weak		hsa-let-7c-5p	RABL2B	weak
hsa-miR-29c-3p	RAB40C	weak		hsa-miR-6511b-5p	HDGF	weak		hsa-let-7c-5p	NOM1	weak
hsa-miR-29c-3p	CAND1	weak		hsa-miR-6511b-5p	TRIM72	weak		hsa-let-7c-5p	YAE1D1	weak
hsa-miR-29c-3p	MDM2	weak		hsa-miR-6511b-5p	UBXN2A	weak		hsa-let-7c-5p	VCL	weak
hsa-miR-29c-3p	ZBTB5	weak		hsa-miR-6511b-5p	GOSR2	weak		hsa-let-7c-5p	RAB40C	weak
hsa-miR-29c-3p	CBX6	weak		hsa-miR-6511b-5p	ICOSLG	weak		hsa-let-7c-5p	PLXND1	weak
hsa-miR-29c-3p	YAE1D1	weak		hsa-miR-6757-5p	ARID3A	weak		hsa-let-7c-5p	SMCR7L	weak
hsa-miR-29c-3p	TRIM72	weak		hsa-miR-6757-5p	TMED10	weak		hsa-let-7c-5p	ICOSLG	weak
hsa-miR-4486	PLXND1	weak		hsa-miR-6757-5p	SOD2	weak		hsa-let-7c-5p	TUBB2A	weak
hsa-miR-4486	NOM1	weak		hsa-miR-6757-5p	GOLGA7	weak		hsa-let-7c-5p	ZBTB5	weak
hsa-miR-4486	PLEKHA3	weak		hsa-miR-6812-5p	NCOA3	weak		hsa-let-7c-5p	PMAIP1	weak
hsa-miR-4486	GOSR2	weak		hsa-miR-6812-5p	TMED10	weak		hsa-let-7c-5p	PLCG2	weak
hsa-miR-4486	ASB6	weak		hsa-miR-6812-5p	SEC22C	weak		hsa-let-7c-5p	C9orf156	weak
hsa-miR-4486	FBXL20	weak		hsa-miR-6812-5p	MDM4	weak		hsa-let-7c-5p	DNA2	weak
hsa-miR-4486	ZNF460	weak		hsa-miR-6812-5p	HDGF	weak		hsa-let-7c-5p	SOD2	weak
hsa-miR-4486	NUPL2	weak		hsa-miR-6812-5p	ASB6	weak		hsa-let-7c-5p	FBXL20	weak
hsa-miR-4486	MDM2	weak		hsa-miR-6812-5p	PLCG2	weak		hsa-let-7c-5p	EPHA4	weak
hsa-miR-4486	TMCO1	weak		hsa-miR-6812-5p	ZNF385A	weak		hsa-let-7c-5p	DUSP1	weak
hsa-miR-4486	C9orf156	weak		hsa-miR-6812-5p	TRIM71	weak		hsa-let-7c-5p	CCNT2	weak
hsa-miR-4486	NAA30	weak		hsa-miR-6812-5p	ATXN2	weak		hsa-let-7c-5p	ZNF460	weak
hsa-miR-4486	DNA2	weak		hsa-miR-6812-5p	NUPL2	weak		hsa-let-7c-5p	PLEKHA3	weak
hsa-miR-4486	CHST6	weak		hsa-miR-6812-5p	FXN	weak		hsa-let-7c-5p	CPA4	weak
hsa-miR-22-5p	SMCR7L	weak		hsa-let-7c-5p	SIRT1	weak		hsa-let-7c-5p	ATXN2	weak
hsa-miR-22-5p	PMAIP1	weak		hsa-let-7c-5p	CBX6	weak				
hsa-miR-22-5p	CCNT2	weak		hsa-let-7c-5p	CHST6	weak				
